# Core Structure–Activity Relationship Studies of 5,7,20-*O*-Trimethylsilybins in Prostate Cancer Cell Models

**DOI:** 10.3390/ph16040531

**Published:** 2023-04-02

**Authors:** Sitong Wu, Guanglin Chen, Eva Y. Chen, Leyla S. Farshidpour, Qiang Zhang, Guangdi Wang, Qiao-Hong Chen

**Affiliations:** 1 Department of Chemistry and Biochemistry, California State University, Fresno, 2555 E. San Ramon Avenue, M/S SB70, Fresno, CA 93740, USA; 2 Department of Chemistry and RCMI Cancer Research Center, Xavier University of Louisiana, 1 Drexel Drive, New Orleans, LA 70125, USA

**Keywords:** silybin, flavonolignan, 5,7,20-*O*-trimethylsilybin, prostate cancer, androgen receptor

## Abstract

Silibinin, also known as silybin, is isolated from milk thistle (*Silybum marianum*). Silibinin has been demonstrated to be a good lead compound due to its potential to prevent and treat prostate cancer. Its moderate potency and poor pharmacokinetic profile hindered it from moving forward to therapeutic use. Our research group has been working on optimizing silibinin for the potential treatment of castration-resistant prostate cancer. Our previous studies established 5,7,20-*O*-trimethylsilybins as promising lead compounds as they can selectively suppress androgen receptor (AR)-positive LNCaP cell proliferation. Encouraged by the promising data, the present study aims to investigate the relationships between the core structure of 5,7,20-*O*-trimethylsilybin and their antiproliferative activities towards AR-positive (LNCaP) and AR-negative prostate cancer cell lines (PC-3 and DU145). The structure–activity relationships among the four different core structures (including flavanonol-type flavonolignan (silibinin), flavone-type flavonolignan (hydnocarpin D), chalcone-type flavonolignan, and taxifolin (a flavonolignan precursor) indicate that 5,7,20-*O*-trimethylsilybins are the most promising scaffold to selectively suppress AR-positive LNCaP prostate cancer cell proliferation. Further investigation on the antiproliferative potency of their optically enriched versions of the most promising 5,7,20-*O*-trimethylsilybins led to the conclusion that (10*R*,11*R*) derivatives (silybin A series) are more potent than (10*S*,11*S*) derivatives (silybin B series) in suppressing AR positive LNCaP cell proliferation.

## 1. Introduction

**Figure 1:**
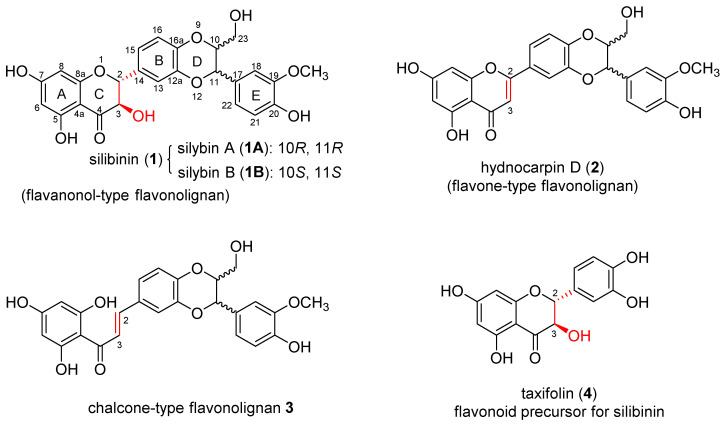
Structures of silibinin, hydnocarpin D, chalcone-type flavonolignan, and taxifolin.

**Figure 2:**
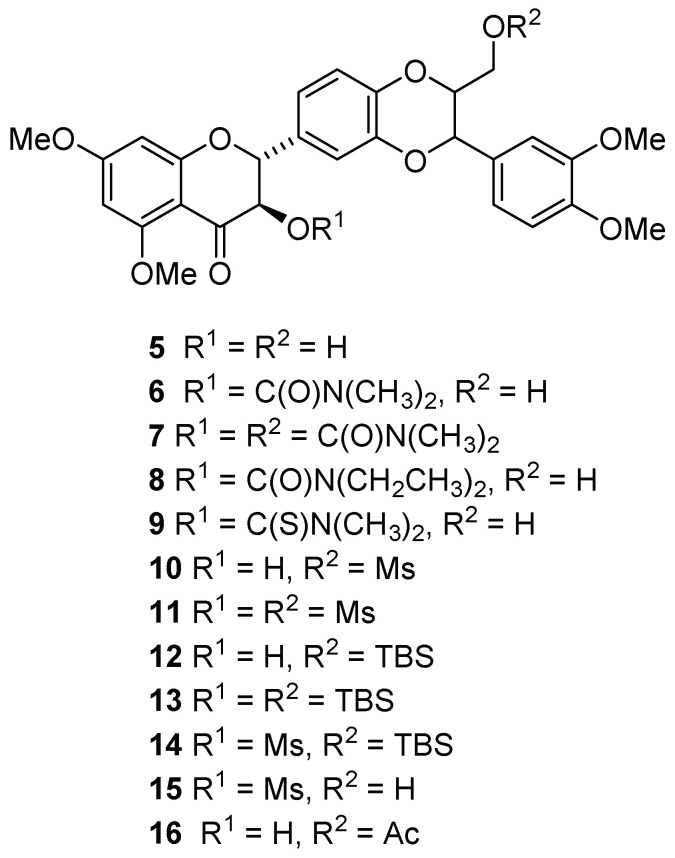
Chemical structures of 5,7,20-*O*-trimethylsilybins. Ms = methanesulfonyl. TBS = *tert*-butyl(dimethyl)silyl. Ac = acetyl.

Silibinin (**1**, also known as silybin, Figure 1) is the first naturally occurring flavonolignan and the major medicinally active chemical component in silymarin, a crude extract of milk thistle (*Silybum marianum* L. Gaertner, Asteraceae) [1]. Silibinin is a diastereomeric mixture of (2*R*,3*R*,10*R*,11*R*) silybin A (**1A**) and (2*R*,3*R*,10*S*,11*S*) silybin B (**1B**), which can be separated by chemoenzymatic resolution using Novozyme 435 as a biocatalyst [2]. The medicinal merit of silymarin has been exploited and taken advantage of by human beings since ancient times. The lasting and extensive research led to the conclusion that silymarin, silibinin, and the derivatives possess a diverse set of biological activities spanning from the well-known hepatoprotective activity to the current anti-COVID-19 activity, which have been elegantly summarized by Křen and Valentová [3]. Silibinin caught our attention due to its potential and promise in treating prostate cancer [4], as demonstrated by the in vitro [5] and in vivo [6,7] experimental data. Equally important, the mechanism of action relates to the androgen receptor (AR)-signaling axis [4] that holds the foremost impetus for the progression of castration-resistant prostate cancer, the lethal version of prostate cancer [8]. Specifically, silymarin, silibinin (**1**), silybin A (**1A**), and silybin B (**1B**) have been reported to reduce the secretion of prostate-specific antigens in AR-positive LNCaP prostate cancer cells [5,9,10,11]. Isosilybin A is a regioisomer of silybin A (**1A**) and has been demonstrated to decrease the AR concentration in three AR-positive cell models (22RV1, LAPC4, and LNCaP) [12]. Isosilybin B is a regioisomer of silybin B (**1B**) and has been reported to downregulate both nuclear and cytoplasmic AR, to inhibit AR nuclear localization, and to promote proteasome-dependent AR degradation through activating Akt [13]. Additionally, the structure scaffold of silibinin (**1**) and derivatives is distinguished from that of all currently marketed AR antagonists, making it possible to become a novel group of AR modulators for prostate cancer. We have been working on optimizing silibinin to improve its potency and selectivity toward AR-positive prostate cancer cells. Our previous studies revealed that: (1) the silibinin scaffold exhibits higher selectivity towards AR-positive LNCaP prostate cancer cells than the 2,3-dehydrosilybin scaffold [14], (2) modifying the alcoholic 23-OH of 3,5,7,20-*O*-tetramethyl-2,3-dehydrosilybin or alcoholic 3-OH and/or 23-OH of 5,7,20-*O*-trimethylsilybin (**5**) led to good selectivity in suppressing LNCaP prostate cancer cell line [15,16]. Among them, 5,7,20-*O*-trimethylsilybin (**5**) and four 3-*O*-carbamoyl-5,7,20-*O*-trimethylsilybins **(6**–**9**) (Figure 2 and Table 1) emerge as very promising lead compounds since they can selectively suppress AR-positive LNCaP cell proliferation. The IC_50_ values of these five 5,7,20-*O*-trimethylsilybins against the LNCaP cells fall into the range of 0.11–0.83 μM, which exhibit up to 660 times greater in vitro antiproliferative potency than silibinin [16]. Encouraged by the promising data, the present study aims to investigate (1) the relationship between four core structures and their antiproliferative activities towards both AR-positive (LNCaP) and AR-null (PC-3 and DU-145) prostate cancer cell lines. The four core structures include silibinin (**1**, flavanonol-type flavonolignan), hydnocarpin D (**2**, flavone-type flavonolignan), chalcone-type flavonolignan **3**, and taxifolin (**4**, flavonoid precursor of silibinin) (Figure 1); and (2) the association between the diastereomeric 5,7,20-*O*-trimethylsilybins and structures and their antiproliferative activities toward both AR-positive (LNCaP and 22Rv1) and AR-null (PC-3 and DU-145) prostate cancer cell lines.

## 2. Results and Discussion

### 2.1. Synthesis and Antiproliferative Evaluation of 5,7,20-O-Trimethylsilybins

**Scheme 1:**
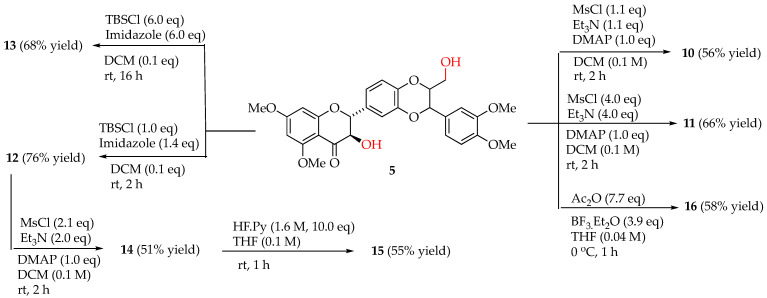
Synthesis of 5,7,20-*O*-trimethylsilybins **10**–**16**. For chemical structures of **10**–**16**, refer to Figure 2.

The promising potency and selectivity of the five 5,7,20-*O*-trimethylsilybins (**5**–**9**) in the AR-positive LNCaP cell model [16] motivated us to delve deeper into this group of derivatives. Seven additional derivatives of 5,7,20-*O*-trimethylsilybin (**10**–**16**) (Figure 2) were prepared from 5,7,20-*O*-trimethylsilybin (**5**) according to the procedures summarized in Scheme 1. Specifically, derivatives **10** and **11** were prepared by mesylating **5** with mesyl chloride, while **12** and **13** were synthesized by treating **5** with TBSCl mediated by imidazole. The mesylation of **12** gave **14**, and the latter was converted to **15** by removing the TBS group. Derivate **16** was made by acetylating **5** with acetic anhydride mediated by borontrifluoride etherate. These derivatives were evaluated by WST-1 cell proliferation for their in vitro antiproliferative potency toward AR-positive LNCaP cells. As illustrated in Scheme 1, Two AR-negative prostate cancer lines (PC-3 and DU145 cells) were used as a comparison to assess the antiproliferative selectivity of AR-positive cells over AR-negative ones. As illustrated in Table 1, four out of seven newly prepared derivatives (**10**, **11**, **15**, **16**) are established as new additions to the promising lead 5,7,20-*O*-trimethylsilybins that can selectively and potentially suppress AR-positive LNCaP cell proliferation with IC_50_ values in the range of 0.51–0.75 μM.

### 2.2. Synthesis, Structural Characterization, and Antiproliferation Evaluation of 5,7,20-O-Trimethylhydnocarpin Ds ***17***–***22***

**Figure 3:**
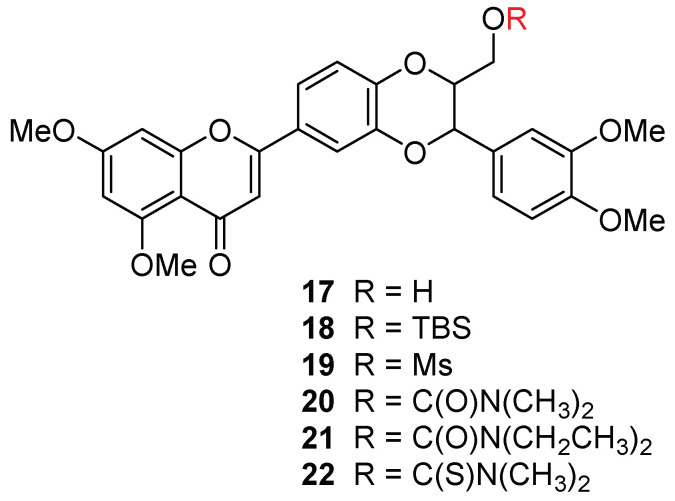
Chemical structures of 5,7,20-*O*-trimethylhydnocarpins.

**Scheme 2:**
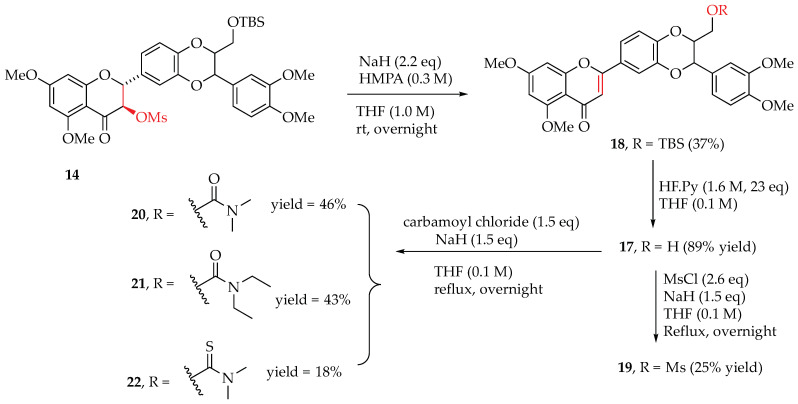
Synthesis of 5,7,20-*O*-trimethylhydnocarpin Ds **17**–**22**.

Hydnocarpin D (**2**, Figure 1) is a luteolin-subtype flavonolignan and was isolated from the flowering plant Hydnocarpus wightiana in extremely limited amounts as a racemic mixture devoid of optical activity [17,18]. The in vitro anti-prostate cancer potency of hydnocarpin D has only been evaluated in the AR-negative DU145 cell line with an IC_50_ value of 4.9 µM [19]. No other anti-prostate cancer activity has been explored for hydnocarpin D or other luteolin-subtype flavonolignans. To compare the antiproliferative potency of 5,7,20-*O*-trimethylsilybins (taxifolin-subtype flavonolignans) with that of 5,7-*O*-trimethylhydnocarpin Ds (luetolin-subtype flavonolignans), 5,7,20-*O*-trimethylhydnocarbin-D (**17**) (Figure 3 and Scheme 2) was first synthesized from 5,7,20-*O*-trimethylsilybin (**5**) through a four-step transformation. To eliminate the secondary aliphatic 3-OH, the primary aliphatic 23-OH was first protected as TBS ether in **12**. A mesyl group was then incorporated into the 3-OH in **14** to yield a good leaving group by reacting with methanesulfonyl chloride (MsCl) mediated by triethylamine and DMAP. The elimination of **14** using sodium hydride as a base and HMPA as a solvent gave 23-*O*-TBS-5,7,20-trimethylhydnocarpin D (**18**). The removal of TBS afforded 5,7,20-*O*-trimethylhydnocarpin D (**17**), which was converted to its mesylate (**19**) and 23-*O*-(thio)carbamoyl derivatives (**20–22**) (Figure 3 and Scheme 2).

Structurally, hydnocarpin D (**2**) differs from silybinin in ring C due to the absence of 3-OH and the presence of a double bond between C-2 and C-3. The chemical structure of 5,7,20-*O*-trimethylhydnocarpin D (**17**) was characterized by its NMR and HRMS. The ^1^H NMR signal at 4.92 ppm for H-2 of 5,7,20-*O*-trimethylsilybin (**5**) disappeared, and the H-3 signal was downshifted from 4.42 ppm for 5,7,20-*O*-trimethylsilybin (**5**) to 6.61 ppm for 5,7,20-*O*-trimethylhydnocarpin D (**17**), indicating the formation of a C2–C3 double bond on ring C for 5,7,20-*O*-trimethylhydnocarpin D. The chemical structure of 5,7,20-*O*-trimethylhydnocarpin D (**17**) was further confirmed by comparing its ^1^H and ^13^C NMR data with those reported for hydnocarpin D (**2**) [18].

Six 5,7,20-*O*-trimethylhydnocarpin D derivatives (**17**–**22**) were evaluated for their antiproliferative activity towards AR-positive (LNCaP) and AR-negative (PC-3 and DU145) prostate cancer cell lines by WST-1 cell proliferation assay. As demonstrated in Table 2, 5,7,20-*O*-trimethylhydnocarpin D (**17**) is the only representative from this group to possess the capability in inhibiting AR-positive LNCaP cell proliferation with the IC_50_ value of 2.84 µM. AR-negative PC-3 and DU145 cells do not respond to the treatment of 5,7,20-*O*-trimethylhydnocarpin D (**17**) up to 25 μM, indicating the selectivity of AR-positive cells over AR-negative ones. However, 5,7,20-*O*-trimethylhydnocarpin D (**17**) is 7-fold less potent than 5,7,20-*O*-trimethylsilibinin (**5**), suggesting the double bond at C-2 and C-3 is not beneficial for the antiproliferative activity in AR-positive LNCaP cells. Additionally, the chemical modification of 23-OH led to the attenuation of antiproliferative activity because all tested 23-*O*-substituted 5,7,20-*O*-trimethylhydnocarpin Ds (**18**–**22**) did not exhibit any capability in suppressing LNCaP cell proliferation up to 25 μM.

### 2.3. Synthesis, Structure Characterization, and Antiproliferative Evaluation of Chalcone-Type Flavonolignans

**Figure 4:**
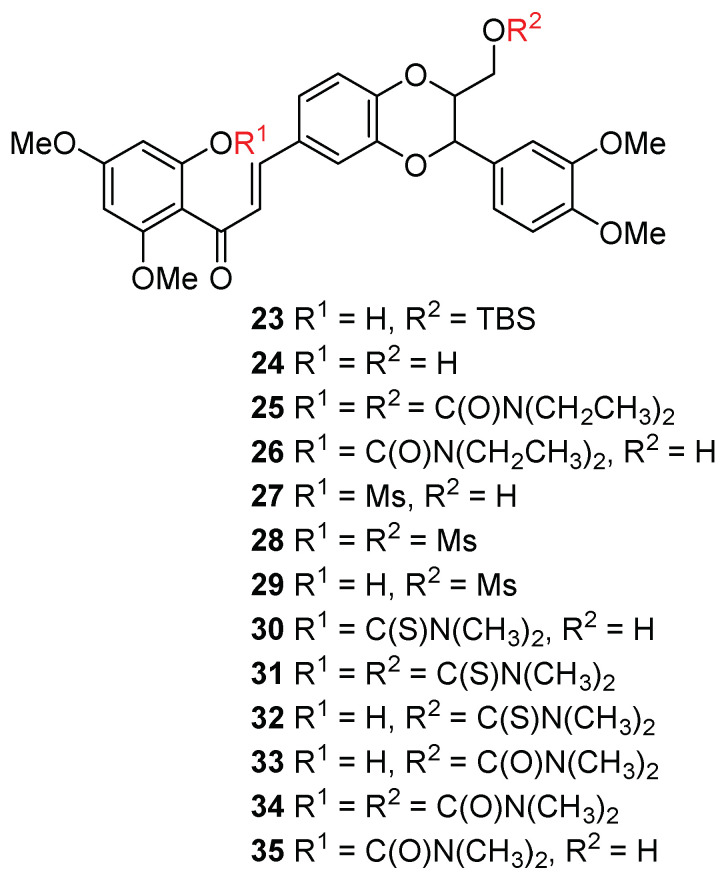
Chemical structures of chalcone-type flavonolignans.

**Scheme 3:**
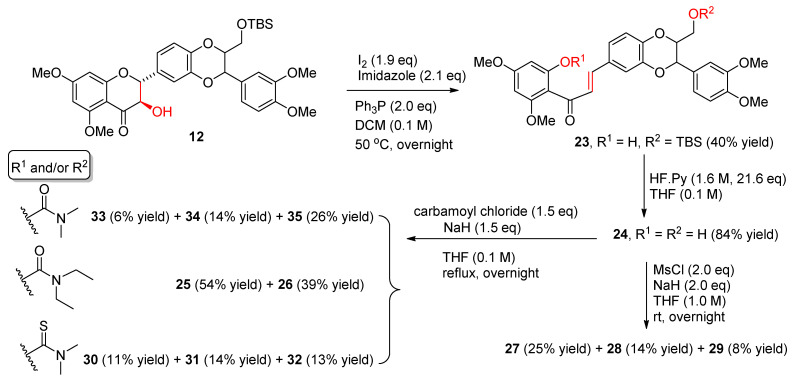
Synthesis of chalcone-type flavonolignans **23**–**35**. For chemical structures of **24**–**35**, refer to Figure 4.

So far, only one chalcone-type flavonolignan was prepared from silibinin (**1**) [20], and none were isolated from nature. Chalcone has been evidenced as a privileged scaffold in the field of drug design and drug discovery due to its robust medicinal properties [21]. Certain natural or synthetic chalcones have been revealed to have appreciable in vitro antiproliferative activity in prostate cancer cell models at sub-micromolar to micromolar concentration [22,23,24,25,26,27,28,29]. The in vivo antitumor efficacy of some chalcones has also been confirmed by the animal experimental data [26,28]. Some chalcones have been demonstrated to boost prostate cancer cell apoptosis mediated by TRAIL (tumor necrosis factor-related apoptosis-inducing ligand) [30]. The anti-prostate cancer activity is associated with different mechanisms of action, including inhibition of 5*α*-reductase, androgen receptor translocation, and sexual hormone synthesis [31]. It is thus intriguing to investigate the capability of chalcone-type flavonolignans in suppressing prostate cancer cell proliferation, especially in comparison with that of 5,7,20-*O*-trimethylsilibins. As shown in Figure 4 and Scheme 3, a chalcone-type flavonolignan (**23**) was synthesized from 23-*O*-TBS-5,7,20-*O*-trimethylsilybin (**12**) through metal-free iodine-mediated deoxygenation of 3-OH followed by opening ring C (breaking O-1 and C-2 bond) by treating with triphenylphosphine (PPh_3_), imidazole, and iodine [20]. Removing the TBS group in **23** gave chalcone-type flavonolignan **24** with one phenolic hydroxyl at C-8a and aliphatic hydroxyl at C-23. At this point, the chemical manipulation of 8a-OH and 23-OH furnished an additional eleven chalcone-type flavonolignans (Figure 4 and Scheme 3).

Chalcone-type flavonolignans are structurally distinguished from silibinin and hydnocarpin D because of the trans α,β-unsaturated ketone derived from the ring C opening. The chemical structure of chalcone-type flavonolignan **23** was elucidated based on its NMR and HRMS. The downshifts of H-3 at 4.42 ppm and H-2 at 4.92 ppm to 7.79 ppm (d, *J* = 15.9 Hz) and 7.71 ppm (d, *J* = 15.9 Hz) imply the existence of the characteristic trans α,β-unsaturated ketone, suggesting the formation of the chalcone scaffold.

Thirteen chalcone-type flavonolignans were synthesized for the evaluation of their antiproliferative activities towards AR-positive (LNCaP) and AR-negative (PC-3 and DU-145) prostate cancer cell lines by WST-1 cell proliferation assay (Table 3). Four chalcone-type flavonolignans (**24**, **29**, **30**, **33**) can preferentially inhibit AR-positive LNCaP cell proliferation with IC_50_ values falling into a range of 2.99–8.48 µM. Similar to 5,7,20-*O*-trimethylhydnocarpin D (**17**), these chalcone-type flavonolignans are 7–21 times less potent than 5,7,20-*O*-trimethylsilybin (**5**) in LNCaP cell models. Another four chalcone-type flavonolignans (**26**, **27**, **34**, **35**) lost selectivity, but possess a moderate inhibitory potency toward both AR-positive (LNCaP) and AR-negative (PC3 and DU145) cells with IC_50_ values of 2.60–8.97 µM.

### 2.4. Antiproliferative Evaluation of Taxifolin Derivatives

**Figure 5:**
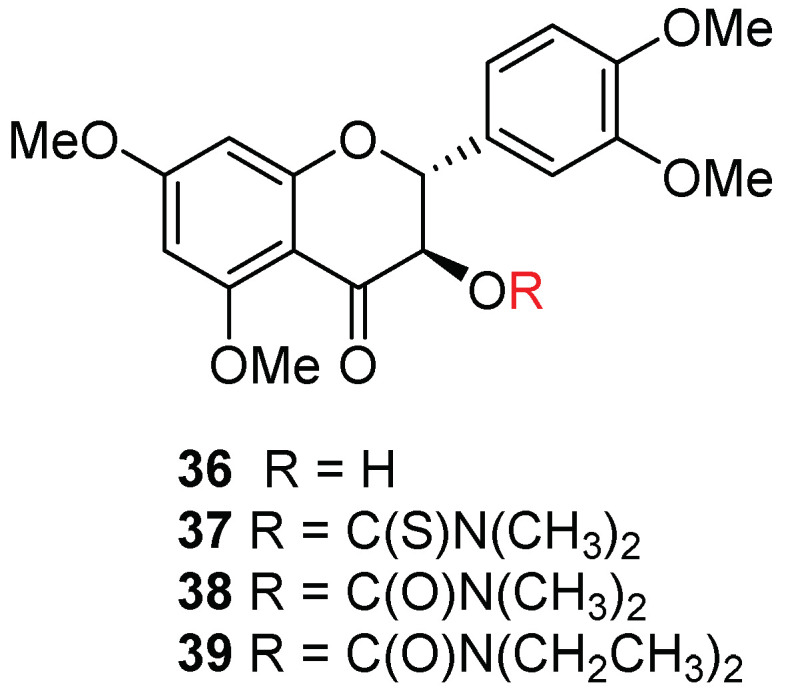
Chemical structures of taxifolins.

**Scheme 4:**
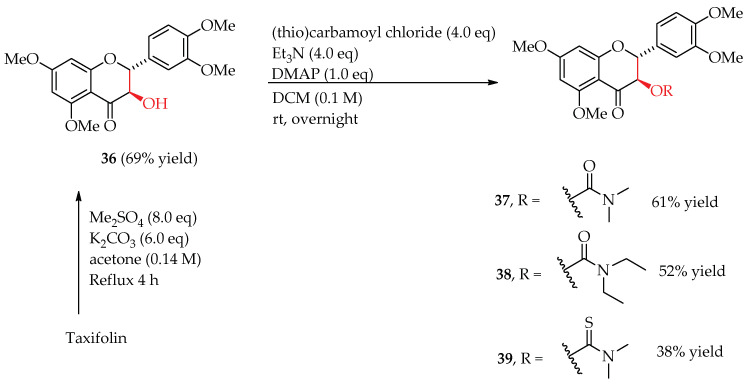
Synthesis of 3-*O*-carbamoyltaxifolins **36**–**39** [16].

Taxifolin (Figure 1) is the biogenetical flavonoid precursor for silybin A and silybin B [32]. The AR-positive LNCaP prostate cancer cells were reported to possess a similar sensitivity to taxifolin and silibinin [5]. It is thus interesting to evaluate the antiproliferative activity of trimethyltaxifolin (**36**) and its 3-*O*-(thio)carbamoyl derivatives (**37**–**39**) (Figure 5) on AR-positive and AR-negative prostate cancer cell models. The four taxifolin derivatives were synthesized according to the procedures reported in our earlier publication (Scheme 4) [16]. As summarized in Table 4, all four derivatives (**36**–**39**) cannot suppress cell proliferation in either AR-positive or AR-negative prostate cancer cell models up to 25 µM, suggesting the lignan portion is imperative to the impressive antiproliferitive potency of silibinin derivatives in the AR-positive LNCaP cell model. This is also in agreement with the notation that flavonolignans are generally more potent than their respective flavonoid precursor in prostate cancer cell models [14].

### 2.5. Synthesis and Antiproliferative Evaluation of Optically Enriched 5,7,20-O-Trimethylsilybins

**Figure 6:**
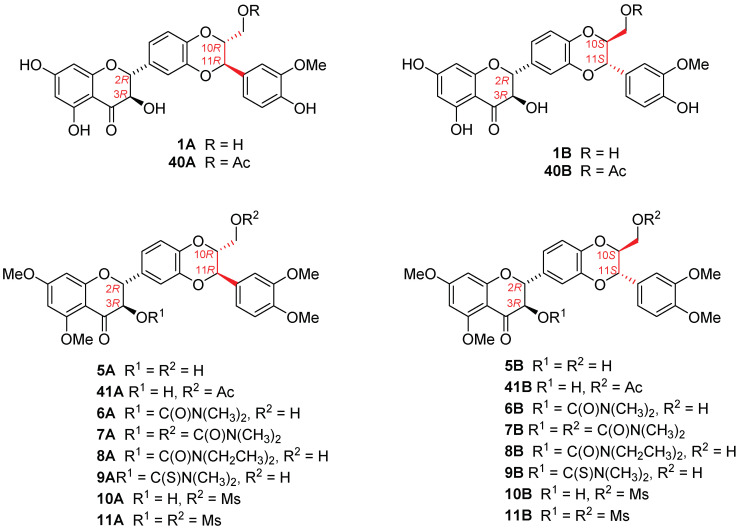
Chemical structures of optically enriched silybins.

**Scheme 5:**
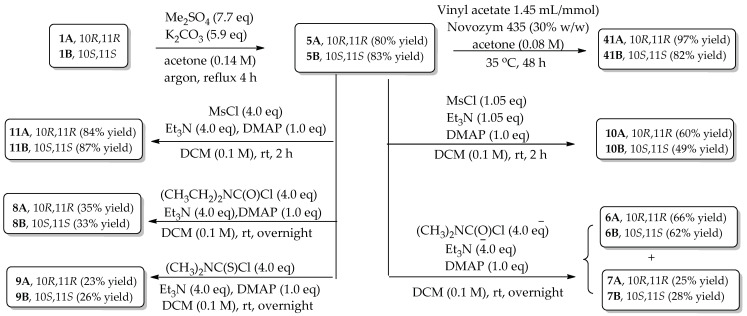
Synthesis of optically enriched 5,7,20-*O*-trimethylsilybins. Chemical structures for the compounds included in this scheme, refer to Figure 6.

The different diastereomeric isomers of silibinin and derivatives have been evidenced to have different biological effects [33,34]. It is thus necessary to further evaluate the antiproliferative potency of the optically pure version for optimal derivatives. The fact that 5,7,20-*O*-trimethylsilybin (**5**) and its derivatives (**6**–**11**, **16**) possess the optimal antiproliferative potency and selectivity in the AR-positive LNCaP cells spurred us to further investigate the antiproliferative potency and selectivity of optically enriched 5,7,20-*O*-trimethylsilybin A (**5A**) and B (**5B**) and their derivatives. Silybin A (**1A**), silybin B (**1B**), 23-*O*-acetylsilybin A (**40A**), and 23-*O*-acetylsilybin B (**40B**) were prepared from diastereomeric silibinin employing the selective transesterification of silibinin (**1**) and stereoselective alcoholysis of 23-*O*-acetylsilybin (**40**) based on the reported procedure [2,35]. Novozym 435 was used as a biocatalyst to discriminate the diastereoisomers. The optically enriched versions of derivatives **6**–**11** were synthesized from the optically enriched 5,7,20-O-trimethysilybin A (**5A**) and B (**5B**) as illustrated in Figure 6 and Scheme 5.

**Figure 7:**
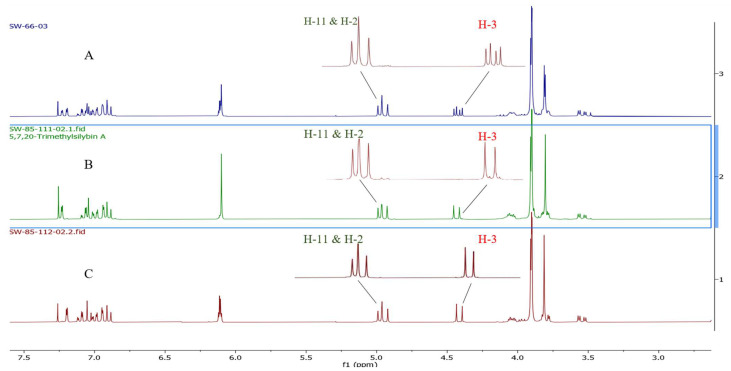
^1^H NMR of 5,7,20-*O*-trimethylsilybin. (**A**) ^1^H NMR spectrum of diastereomeric mixture of 5,7,20-*O*-trimethylsilybin (**5**). (**B**) ^1^H NMR spectrum of optical pure 5,7,20-*O*-trimethylsilybin A (**5A**). (**C**) ^1^H NMR spectrum of optical pure 5,7,20-*O*-trimethylsilybin B (**5B**).

**Figure 8:**
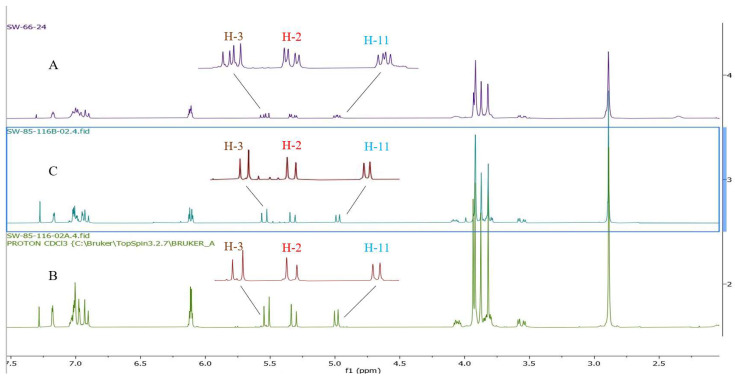
^1^H NMR spectra of 3-*O*-dimethylcarbamoyl-5,7,20-*O*-trimethylsilybin. (**A**) ^1^H NMR spectrum of diastereomeric mixture of 3-*O*-dimethylcarbamoyl-5,7,20-*O*-trimethylsilybin (**6**). (**B**) ^1^H NMR spectrum of optical pure 3-*O*-dimethylcarbamoyl-5,7,20-*O*-trimethylsilybin A (**6A**). (**C**) ^1^H NMR spectrum of optical pure 3-*O*-dimethylcarbamoyl-5,7,20-*O*-trimethylsilybin B (**6B**).

**Figure 9:**
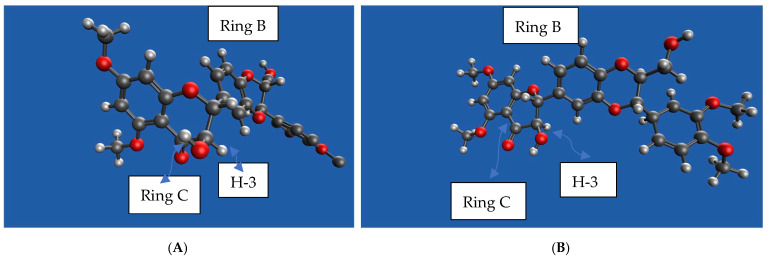
(**A**,**B**) 3D models for **5A** and **5B**. The models were created with IQmol. Red ball represents oxygen (O); black, carbon (C); grey, hygrogen (H).

To confirm the optical purity of silybin A (**1A**) and B (**1B**) isolated from diastereomeric silibinin (**1**), the specific rotation values were measured in acetone and compared to the reported values (Table 5) [35]. Our experimental specific rotation value for silybin A (**1A**) is [*α*]_D_^22^ = +15.14 (*c* 0.7, acetone) and for silybin B (**1B**) is [*α*]_D_^22^ = +2.28 (*c* 1.97, acetone); by comparing the data reported by previous studies, Silybin A (**1A**) and B (**1B**) were confirmed to be separated successfully (Table 5). Optically enriched 5,7,20-*O*-trimethylsilybin A (**5A**) and B (**5B**) were first prepared by the methylation of optically enriched silybin A (**1A**) and silybin B (**1B**) with dimethyl sulfate mediated by potassium carbonate. Fourteen optically enriched derivatives were synthesized for either 5,7,20-*O*-trimethylsilybin A (**5A**) or 5,7,20-*O*-trimethylsilybin B (**5B**). We found the signal for H-3 can serve as a signature signal for (10*R*,11*R*) 5,7,20-*O*-trimethylsilybin A or (10*R*,11*R*) 5,7,20-*O*-trimethylsilybin B. Specifically, the ^1^H NMR signal for H-3 at 4.44 ppm is a pair of doublet signals on the spectrum of diastereomeric 5,7,20-*O*-trimethylsilybin (**5**) (Figure 7), while it is a single doublet on the spectrum of either (10*R*,11*R*) 5,7,20-*O*-trimethylsilybin A (**5A**) or (10*S*,11*S*) 5,7,20-*O*-trimethylsilybin B (**5B**) (Figure 7). The H-3 assignment was supported by the following critical correlation peaks in the set of 2D NMR spectra for **5** (refer to Figures S169–S171 in the Supporting Information): (1) the COSY correlation peaks between H-2 and H-3, and between H-11 and H-10; and (2) the HMBC correlations from H-3 to C-14, and from H-11 to C-10, C-18, C-17, and C-22. Similarly, the featuring ^1^H NMR signals for H-3, H-2, and H-11 at 5.50, 5.28, and 4.94 ppm are pairs of doublets on the spectrum of the diastereomeric mixture of 3-*O*-dimethylcarbamoyl-5,7,20-*O*-trimethylsilybin (**6**, Figure 8), whereas these signals are single doublets on the ^1^H NMR spectrum of (10*R*,11*R*) 3-*O*-dimethylcarbamoyl-5,7,20-*O*-trimethylsilybin A (**6A**) or (10*S*,11*S*) 3-*O*-dimethylcarbamoyl-5,7,20-*O*-trimethylsilybin B (**6B**, Figure 8). The H-3 signal assignment of **6** has been confirmed in our previous research according to the critical HMBC correlations from H-3 to H-4, carbonyl carbon from the dimethyl carbamoyl group, and C-14 [16]. The fact that (10*R*,11*R*) derivatives (5,7,20-*O*-trimethylsilybin A series) and their (10*S*,11*S*) counter partners have slightly different chemical shifts at H-3 is probably caused by the different conformation of ring C and different orientations of H-3 about the aromatic ring B (Figure 9).

The antiproliferative potency and selectivity of ten pairs of diastereomers were evaluated on AR-positive (LNCaP and 22Rv1) and AR-negative (PC-3 and DU145) human prostate cancer cell lines. The IC_50_ values are summarized in Table 6 and reveal that (10*R*,11*R*) derivatives (silybin A series) possess a significantly greater antiproliferative potency and higher selectivity than (10*S*,11S) derivatives (silybin B series) towards LNCaP prostate cancer cell lines. Derivatives **8A** and **41A** were identified as the optimal derivatives with an IC_50_ value of 0.07 μM in the LNCaP cell model. However, these derivatives cannot suppress 22Rv1 prostate cancer cell proliferation up to a 10 μM concentration, revealing that the derivatives of 5,7,20-*O*-trimethylsilybin very likely bind to the ligand-binding domain on AR to exhibit antiproliferative activity in LNCaP cells.

## 3. Materials and Methods

### 3.1. General Procedures

HRMS were obtained on a Thermo Scientific Q-Exactive mass spectrometer with electrospray ionization (ESI). NMR spectra were obtained on a Bruker Fourier 300 spectrometer in CDCl_3_. The chemical shifts are given in ppm referenced to the respective solvent peak, and coupling constants are reported in Hz. All reagents and solvents were purchased from commercial sources and were used without further purification. Silica gel column chromatography was performed using silica gel (32–63 µm). Preparative thin-layer chromatography (PTLC) separations were carried out on thin layer chromatography plates loaded with silica gel 60 GF254 (EMD Millipore Corporation, MA, USA). 5,7,20-*O*-Trimethylsilybin (**5**, HPLC purity 96.3%) was synthesized from silibinin (>98%, purchased from Fischer Scientific, Waltham, MA, USA) using the procedure previously described by us [36]. The synthesis and physical data of compounds **36**–**39** have been included in our recent publication [16]. Silybin A (**1A**) {[*α*]_D_ +14.6 (*c* 0.70, acetone); HRMS (ESI): *m*/*z* calculated for C_25_H_23_O_10_ [M + H]^+^: 483.1292. Found: 483.1294}, silybin B (**1B**) {[*α*]_D_ +2.3 (*c* 2.0, acetone); HRMS (ESI): *m*/*z* calculated for C_25_H_23_O_10_ [M + H]^+^: 483.1292. Found: 483.1293}, 23-*O*-acetylsilybin A (**40A**) {[*α*]_D_ +34.00 (*c* 0.7, acetone); HRMS (ESI): *m*/*z* calculated for C_27_H_25_O_11_ [M + H]^+^: 525.1397. Found: 525.1414}, and 23-*O*-acetylsilybin B (**40B**) {[*α*]_D_ −13.62 (*c* 1.0, acetone); HRMS (ESI): *m*/*z* calculated for C_27_H_25_O_11_ [M + H]^+^: 525.1397. Found: 529.1398} were prepared from purchased silibinin (**1**) employing the selective transesterification of silibinin (**1**) and stereoselective alcoholysis of 23-*O*-acetylsilybin (**40**) based on the reported procedure [2,35]. The HPLC purity analyses were performed on an Agilent Hewlett-Packard 1100 series HPLC DAD system using a 5 µM C18 reversed phase column (4.6 × 250 mm) and a diode array detector. Solvent A is methanol and solvent B is 5% methanol in DI water. All testing samples were run 30 min of 35–100% A in B, with 20 min gradient. The flow rate is 1 mL/min.

### 3.2. Synthesis of 23-O-Methanesulfonyl-5,7,20-O-trimethylsilybins (***10***)

To the solution of 5,7,20-*O*-trimethylsilybin (**5**, 200 mg, 0.38 mmol) in DCM (3.8 mL) were sequentially added triethylamine (0.06 mL, 0.42 mmol), DMAP (46 mg, 0.38 mmol), and methanesulfonyl chloride (0.03 mL, 0.42 mmol). The reaction mixture was stirred at room temperature for 2 h under argon prior to being quenched with a saturated ammonium chloride solution (20 mL). The subsequent mixture was extracted with ethyl acetate (10 mL × 3), and the combined extracts were dried over anhydrous sodium sulfate and concentrated. The crude product was subjected to PTLC purification eluting with purification eluting with DCM:MeOH (95:5, *v*/*v*) to furnish the desired product as a light-yellow foam in 56% yield. ^1^H NMR (300 MHz, CDCl_3_) *δ* 7.20 (dd, *J* = 10.5, 2.1 Hz, 1H, Ar-H), 7.13–6.89 (overlapped, 5H, Ar-H), 6.09 (s, 1H, Ar-H), 6.08 (s, 1H, Ar-H), 4.92 (d, *J* = 12.0 Hz, 1H, H-11), 4.91 (d, *J* = 8.4 Hz, 1H, H-2), 4.42 (dd, *J* = 11.7, 2.4 Hz, 1H, H-23), 4.38 (dd, *J* = 11.7, 4.8 Hz, H-23), 4.22 (d, *J* = 8.4 Hz, 1H, H-3), 4.13–4.06 (m, 1H, H-10), 3.89(s, 3H, OCH_3_), 3.88 (s, 6H, 2 × OCH_3_), 3.80 (3.79) (s, 3H, OCH_3_), 3.06 (s, 3H, SO_2_CH_3_). ^13^C NMR (75 MHz, CDCl_3_) *δ* 109.62, 167.03, 164.81, 162.13, 149.97, 149.55, 143.60 (143.54), 143.34 (143.29), 130.50 (130.46), 127.44, 121.53 (121.17), 120.14, 117.06, 116.61, 111.41, 110.02, 102.86, 93.74 (93.64), 93.24, 82.70, 75.61, 72.65, 72.60, 68.13, 56.29, 56.06, 55.82, 55.68, 37.7. HRMS (ESI) *m*/*z* calculated for C_29_H_31_O_12_S [M + H]^+^: 603.1537. Found: 603.1536. IR (film) *ν*_max_ 3446, 2836, 1670, 1594, 1559, 1518, 1505 cm^−1^. HPLC purity 97.6%.

### 3.3. Synthesis of 3,23-O-Dimethanesulfonyl-5,7,20-O-trimethylsilybins (***11***)

Triethylamine (0.21 mL, 1.51 mmol), methanesulfonyl chloride (0.12 mL, 1.52 mmol), and DMAP (46 mg, 0.38 mmol) were sequentially added to the solution of 5,7,20-*O*-trimethylsilibinin (**5**, 200 mg, 0.38 mmol) in DCM (3.8 mL). The reaction was allowed to proceed with stirring at room temperature under argon for 2 h before being quenched with a saturated ammonium chloride solution (20 mL). The subsequent mixture was extracted with ethyl acetate (10 mL × 3), the combined extracts were dried over anhydrous sodium sulfate, and the organic solvent was removed in vacuo. The crude product was purified via PTLC using DCM:MeOH (9:1, *v*/*v*) as an eluent to provide the desired compound as a white foam in 66% yield. ^1^H NMR (300 MHz, CDCl_3_) *δ* 7.16 (dd, *J* = 8.1, 1.8 Hz, 1H, Ar-H), 7.08–6.89 (m, 5H, Ar-H), 6.13 (s, 1H, Ar-H), 6.12 (s, 1H, Ar-H), 5.32 (dd, *J* = 11.4, 5.7 Hz, 1H, H-23), 5.23 (d, *J* = 11.7 Hz, 1H, H-3), 4.93 (4.92) (d, *J* = 8.1 Hz, 1H, H-11), 4.40 (d, *J* = 11.4 Hz, 1H, H-23), 4.29–4.24 (m, 1H, H-10), 4.11 (4.09) (d, *J* = 11.7 Hz, 1H, H-2), 3.90 (s, 6H, 2 × OCH_3_), 3.89 (s, 3H, OCH_3_), 3.81 (s, 3H, OCH_3_), 3.24 (3.23) (s, 3H, SO_2_CH_3_), 3.07 (s, 3H, SO_2_CH_3_). ^13^C NMR (75 MHz, CDCl_3_) *δ* 184.55, 167.25, 164.37, 162.81, 150.06, 149.64, 143.82 (143.78), 143.72, 128.71 (128.63), 127.42, 121.56 (121.32), 120.24, 117.51 (117.31), 117.12 (116.70), 111.43, 109.98, 104.13 (104.10), 93.93, 93.85, 80.88, 80.17 (80.05), 75.79, 75.69, 68.20, 56.52, 56.14, 56.05, 55.99, 39.83, 37.86. HRMS (ESI) *m*/*z* calculated for C_30_H_33_O_14_S_2_ [M + H]^+^: 681.1312. Found: 681.1335. IR (film) *ν*_max_: 2938, 2840, 1670, 1571, 1508 cm^−1^. HPLC purity 95.0%.

### 3.4. Synthesis of 23-O-TBS-5,7,20-O-Trimethylsilybin (***12***)

Imidazole (36 mg, 0.53 mmol) and TBSCl (57 mg, 0.38 mmol) were sequentially added to the solution of 5,7,20-*O*-trimethylsilibinin (**5**, 200 mg, 0.38 mmol) in DCM (3.8 mL), and the reaction was allowed to proceed at room temperature for 2 h prior to being quenched with a saturated ammonium chloride solution (20 mL). The resulting mixture was extracted with ethyl acetate (10 mL × 3). The organic layers were combined, dried over anhydrous sodium sulfate, and concentrated in vacuo. PTLC purification of the crude product eluting with DCM:MeOH (97:3, *v*/*v*) produced the desired product as a white foam in 76% yield. ^1^H NMR (300 MHz, CDCl_3_) *δ* 7.20 (dd, *J* = 10.8, 1.8 Hz, 1H, Ar-H), 7.11–6.97 (overlapped, 4H, Ar-H), 6.89 (d, *J* = 8.1 Hz, 1H, Ar-H), 6.11 (d, *J* = 2.1 Hz, 1H, Ar-H), 6.10 (br.s, 1H, Ar-H), 5.02 (d, *J* = 8.1 Hz, 1H, H-11), 4.94 (d, *J* = 12.0 Hz, H-2), 4.45 (4.43) (d, *J* = 12.0 Hz, H-3), 3.96 (ddd, *J* = 8.1, 5.1, 2.4 Hz, 1H, H-10), 3.90 (s, 3H, OCH_3_), 3.89 (s, 3H, OCH_3_), 3.88 (s, 3H, OCH_3_), 3.87–3.80 (overlapped, 1H, H-23), 3.80 (3.79) (s, 3H, OCH_3_), 3.55 (dd, *J* = 11.7, 2.4 Hz, 1H, H-23), 0.90 (s, 9H, TBS), 0.07 (s, 3H, TBS), 0.06 (s, 3H, TBS). ^13^C NMR (75 MHz, CDCl3) *δ* 190.97, 167.12, 165.06, 162.24, 149.46, 149.20, 144.76, 143.98, 129.40 (129.35), 129.22, 121.32 (120.86), 120.19, 117.22, 116.67, 111.18, 110.45, 103.02, 93.79, 93.43, 83.12, 78.67, 76.17, 72.64, 62.40, 56.04 (3 × C), 55.92, 25.94, 18.41, −4.95, −5.34. HRMS (ESI): *m*/*z* calculated for C_34_H_43_O_10_Si [M + H]^+^: 639.2626. Found: 639.2623. IR (film) ν_max_: 3446, 2951, 2929, 2882, 2854, 1675, 1606, 1573, 1540, 1507 cm^−1^. HPLC purity 95.1%.

### 3.5. Synthesis of Synthesis of 3,23-O-diTBS-5,7,20-O-Trimethylsilybin (***13***)

Imidazole (118 mg, 1.74 mmol) and TBSCl (260 mg, 1.73 mmol) were sequentially added to the solution of 5,7,20-*O*-trimethylsilibinin (**5**, 151 mg, 0.29 mmol) in DCM (2.9 mL). After stirring at room temperature overnight, the reaction was quenched by adding a saturated ammonium chloride solution (20 mL). The resulting mixture was extracted with ethyl acetate (10 mL × 3), and the organic layers were combined, dried over anhydrous sodium sulfate, and concentrated under a reduced vacuum. The crude product was subjected to PTLC purification eluting with DCM:MeOH (95:5, *v*/*v*) to produce the desired product as a grey foam in 68% yield. ^1^H NMR (300 MHz, CDCl_3_) *δ* 7.09 (dd, *J* = 7.5, 2.1 Hz, 1H, Ar-H), 7.03–6.93 (overlapped, 4H, Ar-H), 6.89 (dd, *J* = 8.4, 1.5 Hz, 1H, Ar-H), 6.09 (d, *J* = 2.4 Hz, 1H, Ar-H), 6.08 (br.s, 1H, Ar-H), 5.01 (5.00) (d, *J* = 11.1 Hz, 1H, H-2), 5.00 (4.98) (d, *J* = 8.1 Hz, H-11), 4.31 (4.27) (d, *J* = 11.1 Hz, 1H, H-3), 3.98–3.90 (m, 1H, H-10), 3.88 (s, 6H, 2 × OCH_3_), 3.87 (s, 3H, OCH_3_), 3.88–3.80 (overlapped, 1H, H-23), 3.78 (3.77) (s, 3H, OCH_3_), 3.57 (dd, *J* = 11.7, 3.0 Hz, 1H, H-23), 0.90 (0.89) (s, 9H, TBS), 0.65 (0.64) (s, 9H, TBS), 0.15 (s, 3H, TBS), 0.08 (0.07) (s, 3H, TBS), 0.07 (0.06) (s, 3H, TBS), −0.23 (−0.26) (s, 3H, TBS). ^13^C NMR (75 MHz, CDCl_3_) *δ* 190.10, 165.89, 164.45, 162.36, 149.37 (149.35), 149.07, 144.16 (144.13), 143.72 (143.61), 130.60 (130.49), 129.22 (129.20), 121.12 (120.90), 119.98, 116.80, 116.73 (116.34), 111.13, 110.45 (110.34), 104.38, 93.32 (2 × C), 83.60 (83.50), 78.62, 76.09 (75.94), 75.94 (75.86), 62.31, 56.26, 55.99, 55.93, 55.56, 25.87, 25.59, 18.30, −4.01, −5.03, −5.35, −6.07 (−6.16). HRMS (ESI): *m*/*z* calculated for C_40_H_57_O_10_Si_2_ [M + H]^+^: 753.3491. Found:753.3489. IR (film) *ν*_max_: 2951, 2927, 2883, 2854, 1692, 1607, 1574, 1507 cm^−1^. HPLC purity 94.9% (two very close signals were observed).

### 3.6. Synthesis of 3-O-Mesyl-23-O-TBS-5,7,20-O-trimethylsilybin (***14***)

To the solution of **12** (161 mg, 0.25 mmol) in DCM (2.5 mL) were sequentially added triethylamine (0.07 mL, 0.50 mmol), methanesulfonyl chloride (0.04 mL, 0.52 mmol), and DMAP (31 mg, 0.25 mmol). The reaction mixture was stirred at room temperature under argon for 2 h before adding a saturated ammonium chloride solution (20 mL) to quench the reaction. The subsequent mixture was extracted with ethyl acetate (10 mL × 3). The ethyl acetate extracts were combined, dried over anhydrous sodium sulfate, and concentrated. PTLC purification of the crude product eluting with hexane:EtOAc (1:1, *v*/*v*) afforded the desired product as a light-yellow foam in 51% yield. ^1^H NMR (300 MHz, CDCl_3_) *δ* 7.16 (7.10) (br.s, 1H, Ar-H), 7.04–6.97 (overlapped, 4H, Ar-H), 6.89 (d, *J* = 8.1 Hz, 1H, Ar-H), 6.14 (br.s, 1H, Ar-H), 6.13 (br.s, 1H, Ar-H), 5.37 (5.34) (d, *J* = 11.1 Hz, 1H, H-3), 5.25 (5.24) (d, *J* = 11.1 Hz, 1H, H-2), 5.01 (5.00) (d, *J* = 7.8 Hz, 1H, H-11), 4.03–3.90 (m, 1H, H-10), 3.90 (s, 9H, 3 × OCH_3_), 3.98–3.86 (overlapped, 1H, H-23), 3.81 (s, 3H, OCH_3_), 3.56 (dd, *J* = 11.7, 2.7 Hz, 1H, H-23), 3.19 (s, 3H, SO_2_CH_3_), 0.89 (s, 9H, TBS), 0.06 (s, 6H, TBS). ^13^C NMR (75 MHz, CDCl_3_) *δ* 184.54, 167.18, 164.41, 162.80, 149.50, 149.23, 144.97, 144.18 (144.06), 129.11, 127.64 (127.55), 121.25 (120.70), 120.26, 117.56 (117.27), 116.98 (116.27), 111.19, 110.46, 104.20, 93.93 (2 × C), 81.10, 80.20 (80.06), 78.70, 76.23, 62.41, 56.42, 56.09, 56.05, 55.92. HRMS (ESI): *m*/*z* calculated for C_35_H_45_O_12_SSi [M + H]^+^: 717.2401. Found: 717.2398. IR (film) *ν*_max_: 2930, 2855, 1688, 1608, 1572, 1508 cm^−1^. HPLC purity 99.0%.

### 3.7. Synthesis of 3-O-Mesyl-5,7,20-O-Trimethylsilybin (***15***)

HF·Py (1.4 mL, 1.6 M) was added dropwise into the solution of **14** (71.6 mg, 0.1 mmol) in THF (1 mL, 0.1M) in a 5 mL plastic reaction vial at 0 °C. The reaction mixture was stirred for 1 h and then transferred into a saturated sodium bicarbonate solution (20 mL). The resulting mixture was extracted with ethyl acetate (10 mL × 3). The organic layers were combined, dried over anhydrous sodium sulfate, and concentrated in vacuo to produce the alcohol as a yellow foam in 55% yield. ^1^H NMR (300 MHz, CDCl_3_) *δ* 7.16 (7.14) (br.s, 1H, Ar-H), 7.03–6.97 (overlapped, 3H, Ar-H), 6.94 (br.s, 1H, Ar-H), 6.89 (d, *J* = 8.4 Hz, 1H, Ar-H), 6.13 (br.s, 1H, Ar-H), 6.12 (s, 1H, Ar-H), 5.35 (5.33) (d, *J* = 11.1 Hz, H-3), 5.23 (d, *J* = 11.1 Hz, H-2), 4.98 (4.96) (d, *J* = 8.4 Hz, 1H, H-11), 4.10–4.03 (m, 1H, H-10), 3.90 (s, 6H, 2 × OCH_3_), 3.89 (s, 3H, OCH_3_), 3.82 (3.81) (s, 3H, OCH_3_), 3.82–3.77 (overlapped, 1H, H-23), 3.53 (dd, *J* = 12.3, 3.9 Hz, 1H, H-23), 3.22 (3.21) (s, 3H, SO_2_CH_3_). ^13^C NMR (75 MHz, CDCl_3_) *δ* 184.51, 167.19, 164.36, 162.77, 149.72, 149.47, 144.39, 144.08, 128.52, 128.23 (128.15), 121.14, 120.28, 117.51 (117.34), 116.83 (116.58), 111.29, 110.13, 104.14, 93.91 (2 × C), 80.96, 80.16 (80.05), 78.39, 76.33, 61.73, 56.41, 56.06 (2 × C), 55.90, 39.72. HRMS (ESI): *m*/*z* calculated for C_29_H_31_O_12_S [M + H]^+^: 603.1537. Found: 603.1536. IR (film) *ν*_max_: 3502, 2933, 1685, 1609, 1571, 1540, 1508 cm^−1^. HPLC purity 97.9% (two very close signals were observed).

### 3.8. Synthesis of 23-O-Acetyl-5,7,20-O-Trimethylsilybin (***16***)

To a solution of 5 (200 mg, 0.38 mmol) in THF (9.5 mL) at 0 °C was added acetic anhydride (0.3 mL, 2.91 mmol) followed by boron trifluoride etherate (0.2 mL, 1.48 mmol). The reaction mixture was stirred at 0 °C for 1 h prior to being quenched with a saturated NaHCO_3_ solution (50 mL). The resulting mixture was extracted with EtOAc (50 mL × 3), the combined extracts were dried over anhydrous Na_2_SO_4,_ and concentrated. The crude product was subjected to PTLC purification eluting with CHCl_3_:acetone:formic acid (90:10:1, *v*/*v*/*v*) to provide the desired product as a white foam in 58% yield. ^1^H NMR (300 MHz, CDCl_3_) *δ* 7.22 (7.20) (d, *J* = 2.1 Hz, 1H, Ar-H), 7.10–7.03 (overlapped, 2H, Ar-H), 6.95–6.88 (overlapped, 3H, Ar-H), 6.11 (d, *J* = 1.5 Hz, 1H, Ar-H), 6.10 (br.s, 1H, Ar-H), 4.95 (d, *J* = 12.3 Hz, 1H, H-2), 4.91 (d, *J* = 8.4 Hz, 1H, H-11), 4.43 (4.41) (d, *J* = 12.3 Hz, 1H, H-3), 4.31 (dd, *J* = 11.7, 2.7 Hz, 1H, H-23), 4.29–4.22 (m, 1H, H-10), 3.98–3.91 (overlapped, 1H, H-23), 3.92 (s, 3H, OCH_3_), 3.90 (s, 6H, 2 × OCH_3_), 3.82 (3.81) (s, 3H, OCH_3_). ^13^C NMR (75 MHz, CDCl_3_) *δ* 190.90, 170.65, 167.17, 165.03, 162.26, 149.95, 149.63, 143.96 (143.92), 143.82, 130.11 (130.07), 128.22, 121.49 (121.22), 120.29, 117.56 (117.46), 116.55, 111.36, 109.94, 103.01, 93.79, 93.43, 82.98, 76.47, 72.83, 72,70, 63.10, 56.50, 56.14, 56.07, 55.94, 20.85. HRMS (ESI): *m*/*z* calculated for C_30_H_31_O_11_ [M + H]^+^: 567.1867. Found: 567.1872. IR (film) ν_max_: 3447, 2938, 1741, 1677, 1608, 1261, 1237, 1216 cm^−1^. HPLC purity 96.0%.

### 3.9. Synthesis of 23-O-TBS-5,7,20-O-Trimethylhydnocarpin D (***18***)

To a solution of **14** (746 mg, 1.0 mmol, 1.0 eq) in THF (1.04 mL) and HMPA (1.04 mL) was added a suspension of NaH (92 mg, 2.3 mmol, 2.2 eq) in HMPA (2.29 mL) at 0 °C via a long needle under argon. The reaction was allowed to proceed with stirring at room temperature overnight under argon prior to being quenched with diethyl either (20 mL). The subsequent mixture was filtered through a Celite pad rinsing with DCM (20 mL). The crude product was subjected to two repetitive PTLC purifications eluting with EtOAc and DCM:MeOH (95:5, *v*/*v*), respectively, to afford the desired product in 37% as a slight yellow solid. ^1^H NMR (300 MHz, CDCl_3_) *δ* 7.64 (d, *J* = 2.1 Hz, 1H, Ar-H), 7.30 (dd, *J* = 8.4 Hz, 1H, Ar-H), 7.03 (dd, *J* = 8.4, 2.1 Hz, 1H, Ar-H), 6.98 (d, *J* = 1.5 Hz, 1H, Ar-H), 6.97 (d, *J* = 8.4 Hz, 1H, Ar-H), 6.68 (s, 1H, H-3), 6.34 (d, *J* = 1.8 Hz, 1H, Ar-H), 6.08 (d, *J* = 1.8 Hz, 1H, Ar-H), 5.01 (d, *J* = 8.1 Hz, 1H, H-11), 4.06–4.01 (m, 1H, H-10), 3.91 (s, 3H, OCH_3_), 3.90 (s, 3H, OCH_3_), 3.88–3.84 (overlapped, 1H, H-23), 3.85 (s, 3H, OCH_3_), 3.55 (dd, *J* = 11.7, 2.7 Hz, 1H, H-23), 0.88 (s, 9H, TBS), 0.06 (s, 3H, TBS), 0.05 (s, 3H, TBS). ^13^C NMR (75 MHz, CDCl_3_) *δ* 180.47, 168.72, 168.69, 159.18, 149.47, 149.11, 146.89, 145.20, 143.84, 128.79, 125.76, 125.67, 120.11, 119.35, 117.16, 111.15, 110.84, 110.40, 105.23, 93.95 (93.87), 89.11 (89.04), 78.88 (78.76), 76.11, 62.21, 56.06, 55.97 (2 × C), 55.81, 25.82 (25,77), 18.25, −5.15 (−5.22), −5.42 (−5.50). HRMS (ESI): *m*/*z* calculated for C_34_H_41_O_9_Si [M + H]^+^: 621.2520. Found: 621.2530. IR (film) *ν*_max_: 2928, 2854, 1694, 1654, 1591, 1504 cm^−1^. HPLC purity 95.4%.

### 3.10. Synthesis of 5,7,20-O-Trimethylhydnocarpin D (***17***)

To a solution of **18** (237 mg, 0.38 mmol, 1.0 eq) in THF (3.8 mL) at 0 °C was added dropwise HF·Py (1.6 M, 5.4 mL) via a needle. The reaction solution was stirred for 3 h at room temperature before being transferred into a separatory funnel with saturated sodium bicarbonate (50 mL), which was extracted with ethyl acetate (15 mL × 3). The extracts were dried over anhydrous sodium sulfate and concentrated. The crude product was purified by PTLC eluting with hexane:ethyl acetate (3:4, *v*/*v*) to furnish the alcohol in 89% as a yellow solid. ^1^H NMR (300 MHz, CDCl_3_) *δ* 7.58 (d, *J* = 1.8 Hz, 1H, Ar-H), 7.24 (dd, *J* = 8.4, 1.5 Hz, 1H, Ar-H), 6.98 (dd, *J* = 8.1, 1.8 Hz, 1H, Ar-H), 6.93 (d, *J* = 8.4 Hz, 1H, Ar-H), 6.93 (d, *J* = 2.1 Hz, 1H, Ar-H), 6.86 (d, *J* = 8.4 Hz, 1H, Ar-H), 6.61 (s, 1H, H-3), 6.34 (d, *J* = 1.8 Hz, 1H, H-6), 6.05 (d, *J* = 1.8 Hz, 1H, H-8), 4.97 (d, *J* = 8.4 Hz, 1H, H-11), 4.08–4.04 (m, 1H, H-10), 3.87 (s, 3H, OCH_3_), 3.85 (s, 3H, OCH_3_), 3.84 (s, 3H, OCH_3_), 3.82 (s, 3H, OCH_3_), 3.75 (dd, *J* = 12.3, 2.7 Hz, 1H, H-23), 3.48 (dd, *J* = 12.6, 3.9 Hz, 1H, H-23). ^13^C NMR (75 MHz, CDCl_3_ + DMSO-d_6_ (*v*/*v* 10:1)) *δ* 180.56, 168.83, 168.79, 159.24, 149.62, 149.32, 146.97, 144.87, 143.88, 128.53, 126.06, 125.62, 120.21, 119.45, 117.32, 111.27, 110.80, 110.18, 105.24, 93.96, 89.17, 78.73, 76.18, 61.23, 55.97 (4 × C). HRMS (ESI): *m*/*z* calculated for C_28_H_27_O_9_ [M + H]^+^: 507.1655. Found: 507.1670. IR (film) *ν*_max_: 3392, 2929, 1690, 1649, 1593, 1505 cm^−1^. HPLC purity 93.3%.

### 3.11. Synthesis of 23-O-Mesyl-5,7,20-O-trimethylhydnocarpin D (***19***)

**Method 1**: Potassium *tert*-butoxide (0.22 mL, 1 M in butanol, 0.22 mmol) was added to the solution of **11** (99 mg, 0.15 mmol) in THF (1.5 mL) at 0 °C. The reaction solution was stirred overnight at 60 °C prior to being quenched with brine (20 mL). The subsequent mixture was extracted with EtOAc (10 mL × 3). The organic layers were combined, dried over anhydrous sodium sulfate, and concentrated in vacuo. The crude product was subjected to PTLC purification eluting with EtOAc to furnish the desired product as a yellow syrup in 5% yield.

**Method 2**: NaH (60% in mineral oil, 6.2 mg, 0.15 mmol) was added into the solution of **17** (52 mg, 0.10 mmol) in THF (1.0 mL) at 0 °C. The mixture was stirred for 30 min before adding methanesulfonyl chloride (0.02 mL, 0.26 mmol). The reaction was then refluxed overnight before adding water (20 mL) to quench the reaction. The subsequent mixture was extracted with EtOAc (10 mL × 3), the organic layers were dried over anhydrous sodium sulfate, and the organic solvents were removed in vacuo. The crude product was purified twice via PTLC eluting with DCM: MeOH (95:5, *v*/*v*) and pure EtOAc, respectively, to produce the desired product as a yellow foam in 25% yield. ^1^H NMR (300 MHz, CDCl_3_) *δ* 7.67 (d, *J* = 2.1 Hz, 1H, Ar-H), 7.35 (dd, *J* = 8.7, 2.1 Hz, 1H, Ar-H), 7.05–6.93 (overlapped, 4H, Ar-H), 6.69 (s, 1H, H-3), 6.37 (d, *J* = 1.5 Hz, 1H, H-6), 6.12 (d, *J* = 1.8 Hz, 1H, H-8), 4.96 (d, *J* = 8.4 Hz, 1H, H-11), 4.44 (dd, *J* = 11.6, 2.4 Hz, 1H, H-23), 4.35–4.30 (m, 1H, H-10), 4.13 (dd, *J* = 11.6, 3.9 Hz, 1H, H-23), 3.95 (s, 3H, OCH_3_), 3.93 (s, 3H, OCH_3_), 3.92 (s, 3H, OCH_3_), 3.89 (s, 3H, OCH_3_), 3.09 (s, 3H, SO_2_CH_3_). ^13^C NMR (75 MHz, CDCl_3_) *δ* 180.69, 169.01, 169.02, 159.50, 150.26, 149.78, 147.36, 143.97, 143.74, 127.34, 126.92, 126.06, 120.30, 119.82, 117.37, 111.61, 110.52, 110.14, 105.43, 94.19, 89.32, 76.07, 75.78, 68.10, 56.35, 56.23, 56.20, 56.18, 37.87. HRMS (ESI): *m*/*z* calculated for C_29_H_29_O_11_S [M + H]^+^: 585.1431. Found: 585.1430. IR (film) *ν*_max_: 2937, 2839, 1693, 1653, 1592, 1505 cm^−1^. HPLC purity 92.1%.

### 3.12. Synthesis of 23-O-(N,N-Dimethylcarbamoyl)-5,7,20-O-trimethylhydnocarpin D (***20***)

**Method 1**: To the solution of **17** (122 mg, 0.24 mmol) in THF (2.4 mL), NaH (60%, 15 mg, 0.36 mmol) at 0 °C was added, and the reaction mixture was stirred for 30 min before adding dimethylcarbamoyl chloride (0.033 mL, 0.36 mmol). The reaction mixture was then refluxed overnight under argon before adding water (20 mL) to quench the reaction. The subsequent mixture was extracted with EtOAc (10 mL × 3), the EtOAc extracts were dried over anhydrous sodium sulfate, and the organic solvents were removed in vacuo. The crude product was subjected to a three-time PTLC purification eluting with EtOAc:hexane (7:3, *v*/*v*), DCM:MeOH (95:5, *v*/*v*) and pure EtOAc, respectively, to provide the desired product as a yellow foam in 46%.

**Method 2**: Triethylamine (0.027 mL, 0.20 mmol), dimethylcarbamyl chloride (0.018 mL, 0.20 mmol), and DMAP (12 mg, 0.10 mmol) were added to the solution of **17** (50 mg, 0.10 mmol) in DCM (1.0 mL), and the reaction solution was refluxed overnight before being quenched with saturated ammonium chloride (20 mL). The resulting mixture was then extracted with ethyl acetate (10 mL × 3), the combined extracts were dried over anhydrous sodium sulfate, and the organic solvents were removed in vacuo. The crude product was sequentially purified via PTLC twice eluting with DCM:MeOH (97:3, *v*/*v*) and pure EtOAc, respectively, to produce **20** as a yellow foam in 8% yield. ^1^H NMR (300 MHz, CDCl_3_) *δ* 7.67 (d, *J* = 2.1 Hz, 1H, Ar-H), 7.34 (dd, *J* = 8.7, 2.1 Hz, 1H, Ar-H), 7.02 (d, *J* = 8.4 Hz, 1H, Ar-H), 6.98 (dd, *J* = 8.4, 1.8 Hz, 1H, Ar-H), 6.93 (d, *J* = 1.8 Hz, 1H, Ar-H), 6.91 (d, *J* = 8.1 Hz, 1H, Ar-H), 6.71 (s, 1H, H-3), 6.38 (d, *J* = 1.8 Hz, 1H, H-6), 6.12 (d, *J* = 1.8 Hz, H-8), 4.93 (d, *J* = 7.8 Hz, 1H, H-11), 4.39–4.31 (overlapped, 2H, H_2_-23), 4.02–3.98 (m, 1H, H-10), 3.95 (s, 3H, OCH_3_), 3.91 (s, 6H, OCH_3_), 3.89 (s, 3H, OCH_3_), 2.89 (s, 6H, *N*(CH_3_)_2_). ^13^C NMR (75 MHz, CDCl_3_) *δ* 180.75, 169.03, 168.95, 159.48, 155.89, 150.05, 149.66, 147.23, 144.69, 143.89, 128.18, 126.44, 126.03 (125.97), 120.35, 119.70 (119.61), 117.60, 111.51, 110.87, 110.10, 105.51, 94.20 (94.12), 89.33 (89.26), 76.93, 76.40, 63.99, 56.33, 56.29 (2 × C), 56.18, 35.65 (2 × C). HRMS (ESI): *m*/*z* calculated for C_31_H_32_NO_10_ [M + H] ^+^: 578.2026. Found: 578.2026. IR (film) *ν*_max_: 2935, 1701, 1651, 1605, 1505 cm^−1^. HPLC purity 91.1%.

### 3.13. Synthesis of 23-O-(N,N-Diethylcarbamoyl)-5,7,20-O-trimethylhydnocarpin D (***21***)

NaH (60% in mineral oil, 6 mg, 0.15 mmol) was added to the solution of **17** (49 mg, 0.10 mmol) in THF (1.0 mL) at 0 °C, and the suspension was for 30 min before adding diethylcarbamoyl chloride (0.02 mL, 0.15 mmol). The reaction mixture was refluxed overnight under argon prior to being quenched with DI water (20 mL). The resulting mixture was extracted with EtOAc (10 mL × 3), the combined organic layers were dried over anhydrous sodium sulfate, and the organic solvents were removed in vacuo. The crude product was subjected to two sequential PTLC purification eluting with DCM:MeOH (95:5, *v*/*v*) and pure EtOAc, respectively, to produce the desired product as a yellow foam in 43% yield. ^1^H NMR (300 MHz, CDCl_3_) *δ* 7.66 (d, *J* = 1.8 Hz, 1H, Ar-H), 7.33 (dd, *J* = 8.4, 1.8 Hz, 1H, Ar-H), 7.01 (d, *J* = 8.4 Hz, 1H, Ar-H), 76.97–6.90 (overlapped, 3H, Ar-H), 6.70 (s, 1H, H-3), 6.37 (d, *J* = 1.8 Hz, 1H, H-6), 6.11 (d, *J* = 1.8 Hz, 1H, H-8), 4.91 (d, *J* = 7.5 Hz, 1H, H-11), 4.37–4.28 (overlapped, 2H, H_2_-23), 4.03–3.97 (m, 1H, H-10), 3.94 (s, 3H, OCH_3_), 3.90 (s, 6H, OCH_3_), 3.88 (s, 3H, OCH_3_), 3.35–3.25 (overlapped, 4H, *N*(*CH_2_*CH_3_)_2_), 1.13 (t, *J* = 6.9 Hz, 6H, *N*(CH_2_*CH_3_*)_2_). ^13^C NMR (75 MHz, CDCl_3_) *δ* 180.70, 168.97, 168.91, 159.42, 155.27, 150.00, 149.63, 147.18, 144.70, 143.85, 128.14, 126.35, 120.32, 119.68, 119.31, 117.52, 111.46, 110.81, 110.05, 105.45, 94.18, 89.32 (89.20), 76.74, 76.47, 63.29, 56.42, 56.20, 56.17, 55.94, 14.24, 13.55 (13.50). HRMS (ESI): *m*/*z* calculated for C_33_H_36_NO_10_ [M + H]^+^: 606.2339. Found: 606.2339. IR (film) *ν*_max_: 2969, 1697, 1655, 1593, 1505 cm^−1^. HPLC purity 94.2%.

### 3.14. Synthesis of 23-O-(N,N-Dimethylthiocarbamoyl)-5,7,20-O-trimethylhydnocarpin D (***22***)

NaH (60% in mineral oil, 6 mg, 0.15 mmol) was added to the solution of **17** (50 mg, 0.10 mmol) in THF (1.0 mL) at 0 °C, and the suspension was stirred for 30 min before adding dimethylthiocarbamoyl chloride (18 mg, 0.15 mmol). The reaction was allowed to proceed with refluxing overnight under argon prior to being quenched with DI water (20 mL). The subsequent mixture was extracted with EtOAc (10 mL × 3), the organic extracts were combined and dried over with sodium sulfate, and the organic solvents were removed under vacuum. The crude product was sequentially purified twice via PTLC. The first PTLC used EtOAc:hexane (7:3, *v*/*v*) as eluent, while the second PTLC developed twice sequentially eluting with DCM:MeOH (95:5, *v*/*v*) and hexane:ethyl acetate (4:1, *v*/*v*) to furnish the desired thiocarbonate as a yellow foam in 18% yield. ^1^H NMR (300 MHz, CDCl_3_) *δ* 7.67 (d, *J* = 2.1 Hz, 1H, Ar-H), 7.34 (dd, *J* = 8.7, 2.1 Hz, 1H, Ar-H), 7.02–6.90 (overlapped, 4H, Ar-H), 6.70 (s, 1H, H-3), 6.37 (d, *J* = 1.8 Hz, 1H, H-6), 6.12 (d, *J* = 1.8 Hz, 1H, H-8), 4.93 (d, *J* = 7.8 Hz, 1H, H-11), 4.69 (dd, *J* = 11.9, 3.0 Hz, 1H, H-23), 4.48–4.43 (m, 1H, H-10), 4.36 (dd, *J* = 11.9, 4.5 Hz, 1H, H-23), 3.94 (s, 3H, OCH_3_), 3.91 (s, 3H, OCH_3_), 3.90 (s, 3H, OCH_3_), 3.89 (s, 3H, OCH_3_), 3.34 (s, 3H, *N*CH_3_), 3.08 (s, 3H, *N*CH_3_). ^13^C NMR (75 MHz, CDCl_3_) *δ* 187.50, 180.69, 168.98, 168.94, 159.44, 150.08, 149.68, 147.21, 144.52, 143.81, 127.97, 126.47, 125.96, 120.35, 119.67 (119.60), 117.51, 111.48, 110.79, 110.02, 105.44, 94.17 (94.08), 89.31 (89.25), 76.89, 76.10, 69.50, 56.37, 56.26, 56.17, 56.08, 43.00, 37.96. HRMS (ESI): *m*/*z* calculated for C_31_H_32_NO_9_S [M + H]^+^: 594.1798. Found: 594.1799. IR (film) *ν*_max_: 2938, 1696, 1655, 1593, 1505 cm^−1^. HPLC purity 91.5%.

### 3.15. Synthesis of Chalcone-Type Flavonolignan ***23***

Iodine (220 mg, 0.87 mmol) was added to the solution of imidazole (71 mg, 1.0 mmol) and triphenylphosphine (250 mg, 0.95 mmol) in DCM (3.8 mL) at 0 °C, and the reaction mixture was stirred for 10 min before adding the solution of **12** (302 mg, 0.47 mmol) in DCM (0.95 mL). The reaction was allowed to proceed with heating at 50 °C overnight prior to being quenched with a saturated sodium thiosulfate solution (20 mL). The subsequent mixture was extracted with ethyl acetate (10 mL × 3), the organic extracts were combined and dried over anhydrous sodium sulfate, and the organic solvents were removed in vacuo. The crude product was subjected to PTLC purification eluting with hexane:ethyl acetate (1:1, *v*/*v*) to produce chalcone **23** as a yellow oil in 43% yield. ^1^H NMR (300 MHz, CDCl_3_) *δ* 7.78(d, *J* = 15.6 Hz, 1H, H-2), 7.72 (d, *J* = 15.6 Hz, 1H, H-3), 77.26 (d, *J* = 2.1 Hz, 1H, Ar-H), 7.15 (dd, *J* = 8.4, 1.8 Hz, 1H, Ar-H), 7.02 (dd, *J* = 8.1, 1.8 Hz, 1H, Ar-H), 6.98 (d, *J* = 2.1 Hz, 1H, Ar-H), 6.97 (d, J = 8.4 Hz, 1H, Ar-H), 6.92 (d, *J* = 8.1 Hz, 1H, Ar-H), 6.09 (d, *J* = 2.4 Hz, 1H, Ar-H), 5.94 (d, *J* = 2.4 Hz, 1H, Ar-H), 5.02 (d, *J* = 7.8 Hz, 1H, H-11), 4.04 (ddd, *J* = 8.1, 3.0, 2.7 Hz, 1H, H-10), 3.91 (s, 3H, OCH_3_), 3.90 (s, 3H, OCH_3_), 3.89 (s, 3H, OCH_3_), 3.86–3.80 (overlapped, 1H, H-23), 3.82 (s, 3H, OCH_3_), 3.57 (dd, *J* = 12.0, 3.0 Hz, 1H, H-23), 0.90 (s, 9H, TBS), 0.07 (s, 3H, TBS), 0.06 (s, 3H, TBS). ^13^C NMR (75 MHz, CDCl_3_) *δ* 192.61, 168.44, 166.13, 162.56, 149.61, 149.24, 146.01, 144.10, 142.60, 129.03, 128.88, 125.61, 123.27, 120.21, 117.40, 116.50, 111.26, 110.47, 106.43, 93.84, 91.30, 78.93, 76.27, 62.33, 56.03, 55.97, 55.68, 55.62, 25.93, 18.40, −5.03, −5.30. HRMS (ESI): *m*/*z* calculated for C_34_H_43_O_9_Si [M + H]^+^: 623.2677. Found: 623.2675. IR (film) *ν*_max_: 2952, 2927, 2853, 1617, 1581, 1559, 1518, 1505 cm^−1^. HPLC purity 96.4% (two very close signals were observed).

### 3.16. Synthesis of Chalcone-Type Flavonolignan ***24***

HF·Py (1.6 M, 1.35 mL) was added dropwise to the solution of **23** (60 mg, 0.10 mmol) in THF (1.0 mL) at 0 °C. The reaction solution was stirred for 3 h at room temperature, and a saturated sodium bicarbonate solution (50 mL) was used to quench the reaction. The subsequent mixture was extracted with ethyl acetate (15 mL × 3), and the combined extracts were dried over anhydrous sodium sulfate and concentrated. PTLC purification of the crude product, using hexane: ethyl acetate (3:4, *v*/*v*) as eluent, produced chalcone **24** as a yellow solid in 84% yield. ^1^H NMR (300 MHz, CDCl_3_) *δ* 7.79 (d, *J* = 15.6 Hz, 1H, H-3), 7.72 (d, *J* = 15.6 Hz, 1H, H-2), 7.27 (d, *J* = 2.1 Hz, 1H, Ar-H), 7.17 (dd, *J* = 8.4, 1.8 Hz, 1H, Ar-H), 7.03 (dd, *J* = 8.1, 1.8 Hz, 1H, Ar-H), 6.99 (d, *J* = 8.1 Hz, 1H, Ar-H), 6.96 (d, *J* = 1.8 Hz, Ar-H), 6.93 (d, *J* = 8.4 Hz, 1H, Ar-H), 6.10 (d, *J* = 2.4 Hz, 1H, Ar-H), 5.95 (d, *J* = 2.4 Hz, 1H, Ar-H), 5.00 (d, *J* = 8.4 Hz, 1H, H-11), 4.14–4.09 (m, 1H, H-10), 3.93 (s, 3H, OCH_3_), 3.92 (s, 3H, OCH_3_), 3.91 (s, 3H, OCH_3_), 3.83 (s, 3H, OCH_3_), 3.83 (dd, *J* = 12.8, 3.0 Hz, 1H, H-23), 3.56 (dd, *J* = 12.8, 3.9 Hz, 1H, H-23). ^13^C NMR (75 MHz, CDCl_3_) *δ* 192.58, 168.43, 166.20, 162.57, 149.89, 149.55, 145.38, 144.10, 142.35, 129.52, 128.35, 125.93, 123.16, 120.29, 117.49, 116.64, 111.42, 110.20, 106.41, 93.89, 91.31, 78.64, 76.39, 61.69, 56.12, 56.09, 56.01, 55.66. HRMS (ESI): *m*/*z* calculated for C_28_H_29_O_9_ [M + H]^+^: 509.1812. Found: 509.1806. IR (film) *ν*_max_: 3502, 2926, 2852, 1621, 1581, 1558, 1518, 1504 cm^−1^. HPLC purity 98.1% (two very close signals were observed).

### 3.17. Synthesis of Chalcone-Type Flavonolignan ***25***

NaH (60% in mineral oil, 7 mg, 0.18 mmol) was added to the solution of **24** (45 mg, 0.09 mmol) in THF (0.09 mL) at 0 °C, and the reaction mixture was stirred for 30 min before adding diethylcarbamoyl chloride (0.02 mL, 0.18 mmol). The reaction was allowed to proceed with stirring overnight at room temperature before being quenched with DI water (20 mL). The resulting mixture was extracted with ethyl acetate (20 mL × 3), and the combined extracts were dried over anhydrous sodium sulfate and concentrated in vacuo. The crude product was subjected to three sequential PTLC purifications, eluting with DCM:MeOH (97:3, *v*/*v*), EtOAch:hexane (7:3, *v*/*v*) and EtOAc:hexane (3:2, *v*/*v*), respectively, to afford chalcone **25** as a yellow solid in 54% yield. ^1^H NMR (300 MHz, CDCl_3_) *δ* 7.36 (d, *J =* 15.9 Hz, 1H, H-2), 7.18 (d, *J =* 2.1 Hz, 1H, H-13), 7.08 (dd, *J =* 8.4, 2.1 Hz, 1H, H-15), 6.94 (d, *J* = 8.4 Hz, 1H, H-16), 6.94 (d, *J* = 7.8 Hz, 1H, H-20), 6.89 (d, *J* = 7.8 Hz, 1H, H-21), 6.88 (s, 1H, H-18), 6.82 (d, *J* = 15.9 Hz, 1H, H-3), 6.41 (d, *J* = 2.1 Hz, 1H, H-8), 6.36 (d, *J* = 2.1 Hz, 1H, H-6), 4.87 (d, *J* = 8.1 Hz, 1H, H-11), 4.32 (dd, *J* = 13.2, 3.3 Hz, 1H, H-23), 4.28 (dd, *J* = 8.1, 3.3 Hz, 1H, H-23), 4.02–3.91 (m, 1H, H-10), 3.89 (s, 3H, OCH_3_), 3.88 (s, 3H, OCH_3_), 3.82 (s, 3H, OCH_3_), 3.77 (s, 3H, OCH_3_), 3.33–3.20 (overlapped, 8H, 4 × *CH_2_*CH_3_), 1.11 (t, *J* = 7.2 Hz, 9H, 3 × CH_2_*CH_3_*), 1.02 (t, 3H, 1 × CH_2_*CH_3_*). ^13^C NMR (75 MHz, CDCl_3_) *δ* 192.64, 161.75, 158.67, 155.25, 153.39, 150.62, 149.95, 149.56, 145.41, 143.89, 128.77, 128.11, 126.99, 123.14, 120.19, 117.51, 116.97(116.79), 116.32, 111.44, 110.11, 100.38 (100.25), 96.45(96.32), 76.32, 75.48, 63.47, 56.19(56.16), 56.02(55.92), 55.82, 55.59, 42.31, 42.00, 14.17, 13.30. HRMS (ESI): *m*/*z* calculated for C_38_H_47_N_2_O_11_ [M + H]^+^: 707.3180. Found: 707.3179. IR (film) *ν*_max_: 2972, 2935, 2838, 1702, 1646, 1609, 1580, 1518, 1504 cm^−1^. HPLC purity 95.9% (two very close signals were observed).

### 3.18. Synthesis of Chalcone-Type Flavonolignan ***26***

**Method 1**: NaH (60% in mineral oil, 7 mg, 0.18 mmol) was added to the solution of **24** (45 mg, 0.09 mmol) in THF (0.09 mL) at 0 °C, and the reaction suspension was stirred for 30 min before adding diethylcarbamoyl chloride (0.02 mL, 0.18 mmol). The reaction was allowed to proceed with stirring at room temperature overnight prior to being quenched with DI water (20 mL). The subsequent mixture was extracted with ethyl acetate (20 mL × 3), the combined extracts were dried over anhydrous sodium sulfate, and the organic solvents were concentrated in vacuo. The crude product was purified via PTLC eluting with DCM:MeOH (95:5, *v*/*v*) to produce **26** as a yellow wax in 39% yield.

**Method 2**: Triethylamine (0.06 mL, 0.44 mmol), diethylcarbamyl chloride (0.06 mL, 0.44 mmol), and DMAP (14 mg, 0.11 mmol) were sequentially added to the solution of **24** (58 mg, 0.11 mmol) in DCM (1.1 mL). The reaction mixture was stirred at room temperature under argon for 4 h, and then saturated ammonium chloride (20 mL) was added to quench the reaction. The resulting mixture was extracted with ethyl acetate (10 mL × 3), the combined extracts were dried over anhydrous sodium sulfate, and the organic solvents were removed in vacuo. PTLC purification of the crude product, eluting with EtOAc:hexane (3:2, *v*/*v*), yielded **26** as a yellow wax in 32% yield. ^1^H NMR (300 MHz, CDCl_3_) *δ* 7.35 (d, *J* = 16.0 Hz, 1H, H-2), 7.18 (d, *J* = 1.8 Hz, 1H, Ar-H), 7.07 (dd, *J* = 8.4, 1.8 Hz, 1H, Ar-H), 6.99 (dd, *J* = 8.4, 1.8 Hz, 1H, Ar-H), 6.93 (d, *J* = 8.4 Hz, 1H, Ar-H), 6.92 (d, *J* = 1.8 Hz, 1H, Ar-H), 6.90 (d, *J* = 8.4 Hz, 1H, Ar-H), 6.82 (d, *J* = 16.0 Hz, 1H, H-3), 6.40 (d, *J* = 2.1, 1H, H-6), 6.35 (d, *J* = 2.1Hz, 1H, H-8), 4.95 (d, *J* = 8,1 Hz, 1H, H-11), 4.08–4.03 (m, 1H, H-10), 3.89 (s, 6H, 2 × OCH_3_), 3.82–3.75 (overlapped, 1H, H-23), 3.82 (s, 3H, OCH_3_), 3.76 (s, 3H, OCH_3_), 3.54 (dd, *J* = 12.3, 3.9 Hz, 1H, H-23), 3.30–3.19 (m, 4H, *N*(*CH_2_*CH_3_)_2_), 1.10 (t, *J* = 7.2 Hz, 3H, *N*CH_2_*CH_3_*), 1.02 (t, *J* = 7.2 Hz, 3H, *N*CH_2_*CH_3_*). ^13^C NMR (75 MHz, CDCl_3_) *δ* 192.69, 161.76, 158.69, 153.41, 150.62, 149.85, 149.52, 145.50, 144.76, 144.06, 128.85, 128.39, 127.02, 122.98, 120.22, 117.41, 116.97, 116.31, 111.39, 110.18, 100.33, 96.41, 78.68, 76.29, 61.68, 56.12, 55.74 (2 × C), 55.74, 42.31, 42.01, 14.18, 13.29. HRMS (ESI): *m*/*z* calculated for C_33_H_38_NO_10_ [M + H]^+^: 608.2496. Found: 608.2493. IR (film) *ν*_max_: 3446, 2959, 2925, 2839, 1716, 1636, 1608, 1577, 1517 cm^−1^. HPLC purity 95.6%.

### 3.19. Synthesis of Chalcone-Type Flavonolignan ***27***

NaH (60% in mineral oil, 7 mg, 0.18 mmol) was added to the solution of **24** (45 mg, 0.09 mmol) in THF (0.09 mL) at 0 °C. After stirring the reaction mixture for 30 min, methanesulfonyl chloride (0.02 mL, 0.18 mmol) was added. The reaction mixture was continued to stir overnight at room temperature, and then DI water (20 mL) was added to quench the reaction. The resulting mixture was extracted with ethyl acetate (20 mL × 3), the combined extracts were dried over anhydrous sodium sulfate, and the organic solvents were removed in vacuo. The crude product was sequentially subjected to twice PTLC purification eluting with DCM:MeOH (97:3, *v*/*v*) and EtOAc:hexane (7:3, *v*/*v*), respectively to produce **27** as a yellow solid in 33% yield. ^1^H NMR (300 MHz, CDCl_3_) *δ* 7.30 (d, *J* = 16.0 Hz, 1H, H-2), 7.19 (d, *J* = 2.1 Hz, 1H, Ar-H), 7.09 (dd, *J* = 8.4, 1.8 Hz, 1H, Ar-H), 7.01–6.89 (overlapped, 4H, Ar-H), 6.84 (d, *J* = 16.0 Hz, 1H, H-3), 6.65 (d, *J* = 2.1 Hz, 1H, H-8), 6.45 (d, *J* = 2.1 Hz, 1H, H-6), 4.97 (d, *J* = 8.4 Hz, 1H, H-11), 4.10–4.05 (m, 1H, H-10), 3.90 (s, 6H, 2 × OCH_3_), 3.85 (s, 3H, OCH_3_), 3.83–3.76 (overlapped, 1H, H-23), 3.78 (s, 3H, OCH_3_), 3.55 (dd, *J* = 12.6, 3.9 Hz, 1H, H-23), 3.12 (s, 3H, SO_2_CH_3_). ^13^C NMR (75 MHz, CDCl_3_) *δ* 191.76, 162.07, 162.01, 158.80, 149.84, 149.50, 147.80, 145.86, 145.46, 144.14, 128.39 (128.25), 126.69, 123.11, 120.23, 117.57, 117.10, 116.19, 111.37, 110.14, 99.88, 97.84, 78.69, 76.30, 61.62, 56.24, 56.09, 56.04, 55.89, 38.09. HRMS (ESI): *m*/*z* calculated for C_29_H_31_O_11_S [M + H]^+^: 587.1587. Found: 587.1587. IR (film) ν_max_: 3523, 2936, 2839, 1704, 1666, 1608, 1578, 1504 cm^−1^. HPLC purity 97.1% (two very close signals were observed).

### 3.20. Synthesis of Chalcone-Type Flavonolignan ***28*** and ***29***

NaH (60% in mineral oil, 11 mg, 0.28 mmol) was added to the solution of **24** (67 mg, 0.13 mmol) in THF (0.13 mL) at 0 °C, and the mixture was stirred for 30 min before adding methansulfonyl chloride (0.02 mL, 0.26 mmol). The reaction mixture was then stirred at room temperature overnight. DI water (20 mL) was then added to quench the reaction. The subsequent mixture was extracted with ethyl acetate (20 mL × 3), the combined extracts were dried over anhydrous sodium sulfate, and the organic solvents were removed in vacuo. The crude product was purified twice via PTLC using DCM:MeOH (95:5, *v*/*v*) and EtOAc:hexane (7:3, *v*/*v*) as eluent to produce chalcone-type flavonolignans **28** and **29**.

#### 3.20.1. Flavonolignan **28**

Yield, 14%; yellow wax. ^1^H NMR (300 MHz, CDCl_3_) *δ* 7.30 (d, *J* = 15.9 Hz, 1H, H-2), 7.20 (d, *J* = 2.1 Hz, 1H, H-13), 7.12 (dd, *J* = 8.7, 2.1 Hz, 1H, H-15), 6.99 (dd, *J* = 8.4, 2.1Hz, 1H, Ar-H), 6.95 (br.s, 1H, Ar-H), 6.94 (d, *J* = 8.4 Hz, 1H, Ar-H), 6.93 (d, *J* = 8.4 Hz, 1H, Ar-H), 6.85 (d, *J* = 15.9 Hz, H-3), 6.66 (d, *J* = 2.1 Hz, 1H, H-8), 6.45 (d, *J* = 2.1 Hz, 1H, H-6), 4.93 (d, *J* = 8.1Hz, 1H, H-11), 4.42 (dd, *J* = 11.4, 2.4 Hz, 1H, H-23), 4.31–4.26 (m, 1H, H-10), 4.11 (dd, *J* = 11.4, 3.6 Hz, 1H, H-23), 3.91 (s, 6H, 2 × OCH_3_), 3.85 (s, 3H, OCH_3_), 3.79 (s, 3H, OCH_3_), 3.12 (s, 3H, SO_2_CH_3_), 3.07 (s, 3H, SO_2_CH_3_). ^13^C NMR (75 MHz, CDCl_3_) *δ* 191.74, 162.11, 158.87, 150.23, 149.73, 147.87, 145.11 (2 × C), 143.91, 128.88, 127.20, 127.00, 123.38, 120.22, 117.57, 117.32, 116.20, 111.56, 110.08, 99.93, 97.92, 76.11, 75.71, 67.98, 56.29, 56.19, 56.11, 55.98, 38.15, 37.85, 14.33. HRMS (ESI): *m*/*z* calculated for C_30_H_33_O_13_S_2_ [M + H]^+^: 665.1363. Found: 665.1385. IR (film) ν_max_: 2958, 2924, 2853, 1646, 1609, 1580, 1519, 1505 cm^−1^. HPLC purity 95.4%.

#### 3.20.2. Flavonolignan **29**

Yield: 8%; yellow wax. ^1^H NMR (300 MHz, CDCl_3_) *δ* 7.78 (d, *J* = 15.6 Hz, 1H, H-2), 7.70 (d, *J* = 15.6 Hz, 1H, H-3), 7.26 (d, *J* = 2.4 Hz, H-13), 7.17 (dd, *J* = 8.4, 2.1 Hz, 1H, H-15), 7.03–6.92 (overlapped, 4H, Ar-H), 6.09 (d, *J* = 2.4 Hz, 1H, H-6), 5.95 (d, *J* = 2.4 Hz, 1H, H-8), 4.95 (d, *J* = 8.1 Hz, 1H, H-11), 4.43 (dd, *J* = 11.7, 2.7 Hz, 1H, H-23), 4.41 (ddd, *J* = 8.1, 3.6, 2.7 Hz, 1H, H-10), 4.11 (dd, *J* = 11.7, 3.6 Hz, 1H, H-23), 3.92 (s, 3H, OCH_3_), 3.91 (s, 3H, OCH_3_), 3.89 (s, 3H, OCH_3_), 3.83 (s, 3H, OCH_3_), 3.08 (s, 3H, SO_2_CH_3_). ^13^C NMR (75 MHz, CDCl_3_) *δ* 192.55, 168.48, 166.27, 162.58, 150.24, 149.73, 144.60, 143.85, 142.03, 129.97, 127.25, 126.32, 123.39, 120.26, 117.46, 116.82, 111.57, 110.12, 106.42, 93.90, 91.35, 76.04, 75.78, 68.07, 56.14 (2 × C), 56.06, 55.71, 37.84. HRMS (ESI): *m*/*z* calculated for C_29_H_31_O_11_S [M + H]^+^: 587.1587. Found: 587.1587. IR (film) ν_max_: 2940, 2910, 2837, 1582, 1561, 1519 cm^−1^. HPLC purity 98.8%.

### 3.21. Synthesis of Chalcone-Type Flavonolignans **30**, **31** and **32**

NaH (60% in mineral oil, 13 mg, 0.33 mmol) was added to a solution of **24** (84 mg, 0.16 mmol) in THF (0.16 mL) at 0 °C. The mixture was stirred for 30 min, to which was added dimethylthiocarbamoyl chloride (41 mg, 0.33 mmol). The reaction was allowed to proceed with stirring at room temperature overnight. DI water (30 mL) was added to quench the reaction, and the mixture was extracted with ethyl acetate (20 mL × 3). The organic layers were combined, dried over anhydrous sodium sulfate, and concentrated under a vacuum. The crude product was purified twice via PTLC using DCM:MeOH (95:5, *v*/*v*) and EtOAc:hexane (7:3, *v*/*v*) as eluent to furnish **30**, **31**, and **32**.

#### 3.21.1. Chalcone-Type Flavonolignan **30**

Yield, 11%; slight yellow foam. ^1^H NMR (300 MHz, CDCl_3_) *δ* 7.46 (d, *J* = 15.9 Hz, 1H, H-2), 7.19 (d, *J* = 2.1Hz, 1H, H-13), 7.11 (dd, *J* = 8.4, 1.8 Hz, 1H, H-15), 7.00 (dd, *J* = 8.4, 2.4 Hz, 1H, H-22), 6.95 (d, *J* = 8.4 Hz, 1H, Ar-H), 6.93 (d, *J* = 2.4 Hz, 1H, Ar-H), 6.91 (d, *J* = 8.4 Hz, 1H, Ar-H), 6.88 (d, *J* = 15.9 Hz, 1H, H-3), 6.40 (d, *J* = 2.1 Hz, 1H, H-6), 6.34 (d, *J* = 2.1 Hz, 1H, H-8), 4.96 (d, *J* = 8.1 Hz, 1H, H-11), 4.09–4.02 (m, 1H, H-10), 3.90 (s, 6H, 2 × OCH_3_), 3.83 (s, 3H, OCH_3_), 3.81–3.74 (overlapped, 1H, H-23), 3.79 (s, 3H, OCH_3_), 3.55 (dd, *J* = 12.6, 3.9 Hz, H-23), 3.31 (s, 3H, *N*CH_3_), 3.22 (s, 3H, *N*CH_3_). ^13^C NMR (75 MHz, CDCl_3_) *δ* 191.83, 186.37, 161.60, 158.87, 152.89, 149.84, 149.51, 145.45, 144.31, 144.05, 128.96, 128.34, 126.81, 122.94, 120.24, 117.46, 117.16 (2 × C), 111.35, 110.10, 101.53, 96.88, 78.63, 76.32, 61.72, 56.21, 56.14, 56.08, 55.81, 43.31, 39.01. HRMS (ESI): *m*/*z* calculated for C_31_H_34_NO_9_S [M + H]^+^: 596.1954. Found: 596.1950. IR (film) ν_max_: 3446, 2935, 2838, 1731, 1609, 1578, 1518, 1504 cm^−1^. HPLC purity 96.9%.

#### 3.21.2. Chalcone-Type Flavonolignan **31**

Yield, 14%^;^ slight yellow foam. ^1^H NMR (300 MHz, CDCl_3_) *δ* 7.45 (d, *J* = 15.9 Hz, 1H, H-2), 7.17 (d, *J* = 2.1 Hz, 1H, H-13), 7.11 (dd, *J* = 8.4, 2.1 Hz, 1H, H-15), 6.95–6.87 (overlapped, 4H, Ar-H), 6.87 (d, *J* = 15.9 Hz, 1H, H-3), 6.40 (d, *J* = 2.1 Hz, 1H, H-6), 6.33 (d, *J* = 2.1 Hz, 1H, H-8), 4.89 (d, *J* = 7.8 Hz, 1H, H-11), 4.66 (dd, *J* = 11.7, 3.0 Hz, 1H, H-23), 4.43–4.37 (m, 1H, H-10), 4.33 (dd, *J* = 11.7, 4.8 Hz, 1H, H-23), 3.89 (s, 6H, 2 × OCH_3_), 3.82 (s, 3H, OCH_3_), 3.78 (s, 3H, OCH_3_), 3.33 (s, 3H, *N*CH_3_), 3.30 (s, 3H, *N*CH_3_), 3.21 ((s, 3H, *N*CH_3_), 3.06 (s, 3H, *N*CH_3_). ^13^C NMR (75 MHz, CDCl_3_) δ 191.76, 187.45, 186.31, 161.57, 158.82, 152.85, 149.95, 149.55, 145.18, 143.80, 128.91, 127.88, 126.77, 122.96, 120.21, 117.53, 117.10, 111.38, 109.94, 101.56, 101.42, 100.08, 96.75, 76.02 (2 × C), 69.50, 56.20, 56.04, 55.93, 55.83, 43.28, 38.94, 37.92. HRMS (ESI): *m*/*z* calculated for C_34_H_39_N_2_O_9_S_2_ [M + H]^+^: 683.2097. Found: 683.2118. IR (film) ν_max_: 2938, 2837, 1641, 1610, 1578, 1518 cm^−1^. HPLC purity 97.3% (two very close signals were observed).

#### 3.21.3. Chalcone-Type Flavonolignan **32**

Yield, 13%; yellow wax. ^1^H NMR (300 MHz, CDCl_3_) *δ* 7.77 (d, *J* = 15.6 Hz, 1H, H-2), 7.70 (d, *J* = 15.6 Hz, 1H, H-3), 7.26 (d, *J* = 2.4 Hz, 1H, H-13), 7.16 (dd, *J* = 8.4, 2.1 Hz, 1H, H-15), 6.98 (d, *J* = 8.4 Hz, 1H, H-22), 6.94 (d, *J* = 1.8 Hz, 1H, H-18), 6.92–6.89 (overlapped, 2H, Ar-H), 6.09 (d, *J* = 2.4 Hz, 1H, H-6), 5.94 (d, *J* = 2.4 Hz, 1H, H-8), 4.91 (d, *J* = 8.1 Hz, 1H, H-11), 4.68 (dd, *J* = 12.0, 3.0 Hz, 1H, H-23), 4.47–4.41 (m, 1H, H-10), 4.34 (dd, *J* = 12. 0, 4.5 Hz, 1H, H-23), 3.90 (s, 6H, 2 × OCH_3_), 3.89 (s, 3H, OCH_3_), 3.82 (s, 3H, OCH_3_), 3.33 (s, 3H, *N*CH_3_), 3.07 (s, 3H, *N*CH_3_). ^13^C NMR (75 MHz, CDCl_3_) *δ* 192.58, 187.48, 168.46, 166.21, 162.57, 150.08, 149.65, 145.16, 143.92, 142.28, 129.56, 127.90, 126.00, 123.28, 120.33, 117.61, 116.66, 111.46, 110.02, 106.43, 93.88, 91.33, 76.90, 76.09, 69.49, 56.12 (3 × C), 55.69, 43.01, 37.94. HRMS (ESI): *m*/*z* calculated for C_31_H_34_NO_9_S [M + H]^+^: 596.1954. Found: 596.1953. IR (film) ν_max_: 2939, 1731, 1619, 1581, 1558, 1503 cm^−1^. HPLC purity 99.7%.

### 3.22. Synthesis of Chalcone-Type Flavonolignans **33**, **34**, and **35**

NaH (60% in mineral oil, 8.5 mg, 0.21 mmol) was added to the solution of **24** (54 mg, 0.11 mmol) in THF (0.1 mL) at 0 °C. The mixture was stirred for 30 min, to which dimethylcarbamoyl chloride was added (0.02 mL 0.21 mmol). The reaction was allowed to proceed with stirring at room temperature overnight before being quenched with DI water (30 mL). The resulting mixture was extracted with ethyl acetate (20 mL × 3). The combined organic layers were dried over anhydrous sodium sulfate. The crude compound was sequentially subjected to two PTLC purifications eluting with DCM:MeOH (95:5, *v*/*v*) and EtOAc:hexane (3:2, *v*/*v*) to afford flavonolignans **33**, **34**, and **35**.

#### 3.22.1. Chalcone-Type Flavonolignan **33**

Yield, 6%; light-yellow foam. ^1^H NMR (300 MHz, CDCl_3_) *δ* 7.79 (d, *J* = 15.6 Hz, 1H, H-2), 7.72 (d, *J* = 15.6 Hz, 1H, H-3), 7.26 (d, *J* = 2.4 Hz, Ar-H), 7.18 (dd, *J* = 8.4, 2.1 Hz, 1H, Ar-H), 7.04–6.90 (overlapped, 4H, Ar-H), 6.10 (d, *J* = 2.4 Hz, 1H, H-6), 5.95 (d, *J* = 2.4 Hz, 1H, H-8), 4.92 (d, *J* = 8.1 Hz, 1H, H-11), 4.39–4.30 (overlapped, 2H, H_2_-23), 4.01–3.96 (m, 1H, H-10), 3.91 (s, 9H, 3 × OCH_3_), 3.83 (s, 3H, OCH_3_), 2.89 (s, 6H, *N*(CH_3_)_2_).

^13^C NMR (75 MHz, CDCl_3_) *δ* 192.63, 168.49, 166.22, 162.60, 155.87, 150.04, 149.62, 145.33, 143.99, 142.37, 129.52, 128.11, 125.98, 123.26, 120.32, 117.68, 116.67, 111.49, 110.11, 106.47, 93.91, 91.36, 76.94, 76.39, 63.89, 56.09 (3 × C), 55.71, 36.66, 36.05. HRMS (ESI): *m*/*z* calculated for C_31_H_34_NO_10_ [M + H]^+^: 580.2183. Found: 580.2181. IR (film) *ν*_max_: 2931, 1707, 1620, 1561, 1518, 1504 cm^−1^. HPLC purity 97.7%.

#### 3.22.2. Chalcone-Type Flavonolignan **34**

Yield, 14%; light-yellow foam. ^1^H NMR (300 MHz, CDCl_3_) *δ* 7.35 (d, *J* = 16.0 Hz, 1H, H-2), 7.17 (d, *J* = 2.1 Hz, 1H, Ar-H), 7.08 (dd, *J* = 8.4, 2.1 Hz, 1H, Ar-H), 6.96–6.87 (overlapped, 4H, Ar-H), 6.83 (d, *J* = 16.0 Hz, 1H, H-3), 6.39 (d, *J* = 2.1 Hz, 1H, Ar-H), 6.35 (d, *J* = 2.1 Hz, 1H, Ar-H), 4.88 (d, *J* = 7.8 Hz, 1H, H-11), 4.35–4.25 (overlapped, 2H, H_2_-23), 3.99–3.93 (m, 1H, H-10), 3.88 (s, 3H, OCH_3_), 3.87 (s, 3H, OCH_3_), 3.81 (s, 3H, OCH_3_), 3.76 (s, 3H, OCH_3_), 2,91 (s, 3H, *N*CH_3_), 2.87 (s, 6H, *N*(CH_3_)_2_), 2.85 (s, 3H, *N*CH_3_). ^13^C NMR (75 MHz, CDCl_3_) *δ* 192.30, 161.83, 158.79, 155.80, 154.06, 150.69, 149.93, 149.51, 145.37, 144.32, 143.89, 128.75, 128.05, 126.94, 123.00, 120.19, 117.56, 116.81, 116.21, 111.40, 110.06, 100.39, 96.49, 77.37, 76.32, 63.81, 56.12, 56.06, 56.00, 55.68, 36.73, 36.49, 35.97. HRMS (ESI): *m*/*z* calculated for C_34_H_39_N_2_O_11_ [M + H]^+^: 651.2554. Found: 651.2551. IR (film) *ν*_max_: 2936, 2839, 1705, 1645, 1608, 1579, 1518, 1504 cm^−1^. HPLC purity 97.0%.

#### 3.22.3. Chalcone-Type Flavonolignan **35**

Yield, 26%; yellow wax. ^1^H NMR (300 MHz, CDCl_3_) *δ* 7.36 (d, *J* = 15.9 Hz, 1H, H-2), 7.19 (d, *J* = 2.1 Hz, 1H, H-13), 7.08 (dd, *J* = 8.4, 2.1 Hz, 1H, H-15), 6.99 (dd, *J* = 8.1, 1.8 Hz, 1H, H-22), 6.94 (d, *J* = 8.4 Hz, 1H, H-16), 6.93 (d, *J* = 1.8 Hz, 1H, H-18), 6.91 (d, *J* = 8.1 Hz, 1H, H-21), 6.83 (d, *J* = 15.9 Hz, 1H, H-3), 6.40 (d, *J* = 2.4 Hz, 1H, H-6), 6.36 (d, *J* = 2.1 Hz, 1H, H-8), 4.96 (d, *J* = 8.1 Hz, 1H, H-11), 4.10–4.04 (m, 1H, H-10), 3.90 (s, 6H, 2 × OCH_3_), 3.82 (s, 3H, OCH_3_), 3.79–3.73 (overlapped, 1H, H-23), 3.77 (s, 3H, OCH_3_), 3.54 (dd, *J* = 12.3, 3.9 Hz, 1H, H-23), 2.92 (s, 3H, *N*CH_3_), 2.86 (s, 3H, *N*CH_3_). ^13^C NMR (75 MHz, CDCl_3_) *δ* 192.4, 161.9, 158.9, 154.1, 150.7, 149.8, 149.5, 145.6, 144.5, 144.1, 128.8, 128.4, 127.0, 123.0, 120.2, 117.5, 116.9, 116.2, 111.4, 110.2, 100.4, 96.5, 78.7, 76.3, 61.7, 56.2, 56.12, 56.09, 55.7, 36.8, 36.6. HRMS (ESI): *m*/*z* calculated for C_31_H_34_NO_10_ [M + H]: 580.2183. Found: 580.2180. IR (film) ν_max_: 3437, 2924, 2852, 1723, 1608, 1578, 1517, 1504 cm^−1^. HPLC purity 95.5%.

### 3.23. Synthesis of Optically Enriched 5,7,20-O-Trimethylsilybin A and B (***5A*** and ***5B***)

Silybin A or silybin B (124 mg, 0.26 mmol) and potassium carbonate (213 mg, 1.54 mmol) were added to a reaction flask. After removing air from the reaction flask by vacuum, acetone (1.8 mL) was added through a syringe, and the mixture was heated at 75 ^o^C for 15 min. Dimethyl sulfate (0.2 mL, 2.0 mmol) was then added through a syringe, and the reaction was allowed to proceed with refluxing for 4 h before being quenched with saturated ammonium chloride (50 mL). The resulting mixture was extracted with EtOAc (30 mL × 3), and the extracts were dried over anhydrous sodium sulfate and concentrated. PTLC purification of the crude product using hexane:EtOAC (3:7) as eluent gave the optically pure 5,7,20-*O*-trimethylsilybin A or 5,7,20-*O*-trimethylsilybin B.

#### 3.23.1. 5,7,20-O-Trimethylsilybin A (**5A**)

Yield, 80%; white solid; [*α*]_D_ +2.0 (*c* 1.0, acetone). ^1^H NMR (300 MHz, CDCl_3_) *δ* 7.24 (d, *J* = 1.8 Hz, 1H, H-13), 7.08 (dd, *J* = 8.1, 1.8 Hz, 1H, H-15), 7.03 (d, *J* = 8.1 Hz, 1H, H-16), 7.00 (dd, *J* = 8.4, 2.1 Hz, 1H, H-22), 6.94 (d, *J* = 2.1 Hz, 1H, H-18), 6.90 (d, *J* = 8.4 Hz, 1H, H-21), 6.11 (s, 2H, H-6 and H-8), 4.98 (d, *J* = 8.4 Hz, 1H, H-11), 4.95 (d, *J* = 12.3 Hz, 1H, H-2), 4.44 (d, *J* = 12.3 Hz, 1H, H-3), 4.08–4.02 (m, 1H, H-10), 3.91 (s, 3H, OCH_3_), 3.97 (s, 3H, OCH_3_), 3.90 (s, 3H, OCH_3_), 3.81 (s, 3H, OCH_3_), 3.81 (dd, *J* = 12.6, 3.0 Hz, 1H, H-23), 3.55 (dd, *J* = 12.6, 4.2 Hz, 1H, H-23). ^13^C NMR (75 MHz, CDCl_3_) *δ* 190.93, 167.16, 165.02, 162.22, 149.72, 149.47, 144.16, 144.03, 130.00, 128.57, 121.39, 120.24, 117.28, 116.49, 111.25, 110.01, 102.97, 93.74, 93.41, 82.98, 78.32, 76.34, 72.68, 61.79, 56.36, 56.10 (2 × C), 55.87. HRMS (ESI): *m*/*z* calculated for C_28_H_29_O_10_ [M + H]^+^: 525.1761. Found: 525.1761. IR (film) *ν*_max_: 3467, 2938, 1674, 1609, 1573, 1509, 1463, 1262, 1110 cm^−1^. HPLC purity 95.4%.

#### 3.23.2. 5,7,20-*O*-Trimethylsilybin B (**5B**)

Yield, 83%; white solid; [*α*]_D_ −19.8 (*c* 1.0, acetone). ^1^H NMR (300 MHz, CDCl_3_) *δ* 7.20 (d, *J* = 2.1 Hz, 1H, H-13), 7.10 (dd, *J* = 8.4, 2.1 Hz, 1H, H-15), 7.04 (d, *J* = 8.4 Hz, 1H, H-16), 7.00 (dd, *J* = 8.1, 1.8 Hz, 1H, H-22), 6.94 (d, *J* = 1.8 Hz, 1H, H-18), 6.90 (d, *J* = 8.4 Hz, 1H, H-21), 6.12 (d, *J* = 2.4 Hz, 1H, H-6), 6.10 (d, *J* = 2.4 Hz, 1H, H-8), 4.98 (d, *J* = 8.4 Hz, 1H, H-11), 4.94 (d, *J* = 12.3 Hz, 1H, H-2), 4.41 (d, *J* = 12.3 Hz, 1H, H-3), 4.06–4.01 (m, 1H, H-10), 3.91 (s, 3H, OCH_3_), 3.90 (s, 3H, OCH_3_), 3.89 (s, 3H, OCH_3_), 3.81 (s, 3H, OCH_3_), 3.80 (dd, *J* = 12.3, 3.0 Hz, 1H, H-23), 3.54 (dd, *J* = 12.6, 4.2 Hz, 1H, H-23). ^13^C NMR (75 MHz, CDCl_3_) *δ* 190.87, 167.14, 165.00, 162.24, 149.73, 149.49, 144.10, 143.96, 130.06, 128.59, 120.98, 120.24, 117.40, 116.73, 111.28, 110.05, 102.99, 93.76, 93.41, 82.97, 78.37, 76.38, 72.78, 61.77, 56.34, 56.11, 56.08, 55.86. HRMS (ESI): *m*/*z* calculated for C_28_H_2 9_O_10_ [M + H]^+^: 525.1761. Found: 525.1763. IR (film) ν_max_: 3447, 2938, 1673, 1608, 1573, 1509, 1463, 1423, 1261 cm^−1^. HPLC purity 97.9%.

### 3.24. Synthesis of Optically Enriched 23-O-Acetyl-5,7,20-O-Trimethylsilybin A and B

Novozym 435 (421 mg, 30% *w*/*w*) and vinyl acetate (3.87 mL) was added to a solution of optically pure 5,7,20-*O*-trimethylsilybin A or 5,7,20-*O*-trimethylsilybin B (1.40 g, 2.67 mmol) in acetone (35 mL). The mixture was stirred at 35 °C for 48 h and then filtered off novozym 435. The filtrate was concentrated to produce a crude product, which was subjected to column chromatography for purification eluting with chloroform:acetone:formic acid (90:10:1, *v*/*v*/*v*) to furnish the optically pure 23-*O*-acetyl-5,7,20-*O*-trimethylsilybin A (**41A**) or 23-*O*-acetyl-5,7,20-*O*-trimethylsilybin B (**41B**).

#### 3.24.1. 23-*O*-Acetyl-5,7,20-*O*-Trimethylsilybin A (**41A**)

Yield, 97%; white foam; [*α*]_D_ +22.2 (*c* 1.28, acetone). ^1^H NMR (300 MHz, CDCl_3_) *δ* 7.20 (d, *J* = 1.8 Hz, 1H, H-13), 7.08 (dd, *J* = 8.7, 2.1 Hz, 1H, H-15), 7.03 (d, *J* = 8.1 Hz, 1H, H-16), 6.93–6.86 (overlapped, 3H, H-18, H-21 and H-22), 6.08 (s, 2H, H-6 and H-8), 4.92 (d, *J* = 12.0 Hz, 1H, H-2), 4.89 (d, *J* = 7.5 Hz, 1H, H-11), 4.40 (d, *J* = 12.0 Hz, 1H, H-3), 4.29 (dd, *J* = 12.0, 2.7 Hz, 1H, H-23), 4.26–4.21 (m, 1H, H-10), 3.93 (dd, *J* = 12.0, 4.5 Hz, 1H, H-23), 3.88 (s, 9H, 3 × OCH_3_), 3.79 (s, 3H, OCH_3_), 2.06 (s, 3H, OAc). ^13^C NMR (75 MHz, CDCl_3_) *δ* 190.76, 170.52, 167.02, 164.88, 162.11, 149.82, 149.48, 143.82, 143.70, 129.98, 128.11, 121.36, 120.19, 117.32, 116.52, 111.25, 109.86, 102.87, 93.71, 93.33, 82.84, 76.35, 75.66, 72.58, 62.97, 56.33, 56.07, 55.88, 55.67, 20.79. HRMS (ESI): *m*/*z* calculated for C_30_H_31_O_11_ [M + H]^+^: 567.1867. Found: 567.1869. IR (film) *ν*_max_: 3443, 2942, 2839, 1741, 1675, 1607, 1573, 1509, 1462, 1260, 1237, 1216 cm^−1^. HPLC purity 98.5%.

#### 3.24.2. 23-*O*-Acetyl-5,7,20-*O*-Trimethylsilybin B (**41B**)

Yield, 82%; white foam; [*α*]_D_ −17.0 (*c* 1.19, acetone). ^1^H NMR (300 MHz, CDCl_3_) *δ* 7.20 (d, *J* = 1.8 Hz, 1H, H-13), 7.11 (dd, *J* = 8.4, 2.1 Hz, 1H, H-15), 7.06 (d, *J* = 8.4 Hz, 1H, H-16), 6.95–6.87 (overlapped, 3H, H-18, H-21 and H-22), 6.12 (d, *J* = 2.0 Hz, 1H, H-6), 6.11 (d, *J* = 2.0 Hz, 1H, H-8), 4.95 (d, *J* = 12.3 Hz, 1H, H-2), 4.91 (d, *J* = 6.9 Hz, 1H, H-11), 4.41 (d, *J* = 12.3 Hz, 1H, H-3), 4.31 (dd, *J* = 12.0, 2.7 Hz, 1H, H-23), 4.2–4.22 (m, 1H, H-10), 3.99–3.91 (overlapped, 1H, H-23), 3.91 (s, 3H, OCH_3_), 3.90 (s, 6H, 2 × OCH_3_), 3.82 (s, 3H, OCH_3_), 2.08 (s, 3H, OAc). ^13^C NMR (75 MHz, CDCl_3_) *δ* 190.86, 170.62, 167.16, 165.01, 162.26, 149.96, 149.63, 143.92, 143.81, 130.11, 128.22, 121.22, 120.29, 117.54, 116.68, 111.36, 109.95, 103.01, 93.77, 93.44, 82.97, 76.50, 75.88, 72.81, 63.01, 56.39, 56.13, 56.07, 55.88, 20.84. HRMS (ESI): *m*/*z* calculated for C_30_H_31_O_11_ [M + H]^+^: 567.1867. Found: 567.1871. IR (film) *ν*_max_: 3448, 2942, 1741, 1676, 1608, 1573, 1509, 1262, 1237, 1216 cm^−1^. HPLC purity 98.3%.

### 3.25. Synthesis of Optically Enriched 23-O-Mesyl-5,7,20-O-Trimethylsilybin A and B

Triethylamine (15 μL, 0.1 mmol) and DMAP (12 mg, 0.095 mmol) were sequentially added to a solution of 5,7,20-*O*-trimethylsilybin A or 5,7,20-*O*-trimethylsilybin B (50 mg, 0.095 mmol) in DCM (0.95 mL). The mixture was stirred at room temperature for 10 min before adding methanesulfonyl chloride (8 μL, 0.10 mmol). The reaction was continued at room temperature for two additional hours prior to being quenched with saturated ammonium chloride solution (20 mL). The resulting mixture was extracted with EtOAc (20 mL × 3), and the extracts were dried over anhydrous sodium sulfate and concentrated. The crude product was subjected to PTLC purification eluting with DCM:MeOH (95:5, *v*/*v*) to yield 23-*O*-mesyl-5,7,20-*O*-trimethylsilybin A (**10A**) or 23-*O*-mesyl-5,7,20-*O*-trimethylsilybin B (**10B**).

#### 3.25.1. 23-*O*-Mesyl-5,7,20-*O*-Trimethylsilybin A (**10A**)

Yield, 60%; slight yellow foam; [*α*]_D_ +8.9 (*c* 0.18, acetone). ^1^H NMR (300 MHz, CDCl_3_) *δ* 7.25 (d, *J* = 2.1 Hz, 1H, H-13), 7.10 (dd, *J* = 8.4, 1.8 Hz, 1H, H-15), 7.03 (d, *J* = 8.1 Hz, 1H, H-16), 6.99 (dd, *J* = 8.4, 2.1 Hz, 1H, H-22), 6.93 (s, 1H, H-18), 6.92 (d, *J* = 8.1 Hz, 1H, H-21), 6.11 (s, 2H, H-6 and H-8), 4.95 (d, *J* = 12.0 Hz, 1H, H-2), 4.94 (d, *J* = 8.4 Hz, 1H, H-11, 4.44 (dd, *J* = 11.7, 2.4 Hz, 1H, H-23), 4.41 (d, *J* = 12.3 Hz, 1H, H-3), 4.27–4.22 (m, 1H, H-10), 4.11 (dd, *J* = 11.7, 3.6 Hz, 1H, H-23), 3.914 (s, 3H, OCH_3_), 3.911 (s, 3H, OCH_3_), 3.907 (s, 3H, OCH_3_), 3.81 (s, 3H, OCH_3_), 3.09 (s, 3H, SO_2_CH_3_). ^13^C NMR (75 MHz, CDCl_3_) *δ* 190.78, 167.17, 164.95, 162.24, 150.06, 149.64, 143.73, 143.47, 130.52, 127.49, 121.68, 120.21, 117.21, 116.66, 111.44, 109.97, 102.95, 93.76, 93.43, 82.85, 75.85, 75.68, 72.69, 68.28, 56.39, 56.11 (2 × C), 55.89, 37.87. HRMS (ESI): *m*/*z* calculated for C_29_H_31_O_12_S [M + H]^+^: 603.1537. Found: 603.1534. IR (film) *ν*_max_: 3446, 2937, 2840, 2367, 1674, 1607, 1573, 1509, 1460, 1423, 1353, 1261 cm^−1^. HPLC purity 95.2%.

#### 3.25.2. 23-*O*-Mesyl-5,7,20-*O*-Trimethylsilybin B (**10B**)

Yield, 49%; slight yellow foam; [*α*]_D_ −13.0 (*c* 0.63, acetone). ^1^H NMR (300 MHz, CDCl_3_) *δ* 7.22 (d, *J* = 2.1 Hz, 1H, H-13), 7.13 (dd, *J* = 8.4, 2.1 Hz, 1H, H-15), 7.05 (d, *J* = 8.4 Hz, 1H, H-16), 7.00 (dd, *J* = 8.1, 2.1 Hz, 1H, H-22), 6.94 (d, *J* = 2.1 Hz, 1H, H-18), 6.92 (d, *J* = 8.1 Hz, 1H, H-22), 6.12 (s, 2H, H-6 and H-8), 4.95 (d, *J* = 12.0 Hz, 1H, H-2), 4.94 (d, *J* = 9.0 Hz, 1H, H-11), 4.45 (dd, *J* = 11.7, 2.1 Hz, 1H, H-23), 4.40 (d, *J* = 12.0 Hz, 1H, H-3), 4.27–4.22 (m, 1H, H-10), 4.11 (dd, *J* = 11.7, 3.9 Hz, 1H, H-23), 3.918 (s, 3H, OCH_3_), 3.915 (s, 3H, OCH_3_), 3.910 (s, 3H, OCH_3_), 3.82 (s, 3H, OCH_3_), 3.09 (s, 3H, SO_2_CH_3_). ^13^C NMR (75 MHz, CDCl_3_) *δ* 190.78, 167.19, 164.97, 162.28, 150.09, 149.68, 143.69, 143.44, 130.58, 127.50, 121.30, 120.24, 117.37, 116.94, 111.46, 109.97, 102.99, 93.80, 93.46, 82.88, 75.91, 75.75, 72.82, 68.28, 56.20, 56.15, 56.13, 56.10, 37.92. HRMS (ESI): *m*/*z* calculated for C_29_H_31_O_12_S [M + H]^+^: 603.1537. Found: 603.1537. IR (film) *ν*_max_: 3446, 2936, 1674, 1608, 1573, 1509, 1463, 1423, 1353, 1262 cm^−1^. HPLC purity 95.5%.

### 3.26. Synthesis of Optically Enriched 3,23-O-Dimesyl-5,7,20-O-Trimethylsilybin A and B

Triethylamine (53 μL, 0.38 mmol) and DMAP (12 mg, 0.095 mmol) were sequentially added to a solution of 5,7,20-*O*-trimethylsilybin A or 5,7,20-*O*-trimethylsilybin B (50 mg, 0.095 mmol) in DCM (0.95 mL). The mixture was attired at room temperature for 10 min before adding methanesulfonyl chloride (29 μL, 0.38 mmoL), The reaction was stirred at room temperature for 2 h prior to being quenched with a saturated ammonium chloride solution (20 mL). The resulting mixture was extracted with EtOAc (20 mL × 3), the extracts were dried over anhydrous sodium sulfate, and the organic solvent was removed in vacuo. PTLC purification of the crude product eluting with DCM:MeOH (95:5, *v*/*v*) furnished 3,23-*O*-dimesyl-5,7,20-*O*-trimethylsilybin A (**11A**) or 3,23-*O*-dimesyl-5,7,20-*O*-trimethylsilybin B (**11B**).

#### 3.26.1. 3,23-*O*-Dimesyl-5,7,20-*O*-Trimethylsilybin A (**11A**)

Yield, 84%; white foam; [*α*]_D_ +54.3 (*c* 0.21, acetone). ^1^H NMR (300 MHz, CDCl_3_) *δ* 7.18 (d, *J* = 1.8 Hz, 1H, H-13), 7.07–6.93 (overlapped, 4H, H-15, H-16, H-18 and H-22), 6.90 (d, *J* = 8.4 Hz, 1H, H-21), 6.128 (s, 1H, H-6), 6.125 (s, 1H, H-8), 5.32 (d, *J* = 11.3 Hz, H-3), 5.24 (d, *J* = 11.3 Hz, 1H, H-2), 4.93 (d, *J* = 8.1 Hz, H-11), 4.41 (dd, *J* = 11.4, 2.4 Hz, 1H, H-23), 4.31–4.26 (m, 1H, H-10), 4.10 (dd, *J* = 11.7, 2.4 Hz, 1H, H-23), 3.91 (s, 6H, 2 × OCH_3_), 3.90 (s, 3H, OCH_3_), 3.82 (s, 3H, OCH_3_), 3.25 (s, 3H, SO_2_CH_3_), 3.08 (s, 3H, SO_2_CH_3_). ^13^C NMR (75 MHz, CDCl_3_) *δ* 184.51, 167.24, 164.36, 162.79, 150.05, 149.64, 143.81, 143.71, 128.71, 127.41, 121.55, 120.24, 117.30, 116.73, 111.42, 110.00, 104.09, 93.91 (2 × C), 80.87, 80.15, 75.80, 75.68, 68.17, 56.50, 56.19, 56.12, 56.01, 39.81, 37.84. HRMS (ESI): *m*/*z* calculated for C_30_H_33_O_14_S_2_ [M + H]^+^: 681.1312. Found: 681.1306. IR (film) *ν*_max_: 2939, 1689, 1608, 1572, 1509, 1463, 1356, 1263, 1174, 1161 cm^−1^. HPLC purity 96.0%.

#### 3.26.2. 3,23-*O*-Dimesyl-5,7,20-*O*-Trimethylsilybin B (**11B**)

Yield, 87%; white foam; [*α*]_D_ +27.3 (*c* 0.41, acetone). ^1^H NMR (300 MHz, CDCl_3_) *δ* 7.14 (d, *J* = 1.8 Hz, 1H, H-13), 7.06 (dd, *J* = 8.4, 2.1 Hz, 1H, H-15), 7.02 (d, *J* = 8.4 Hz, 1H, H-16), 6.98 (dd, *J* = 8.4, 2.1 Hz, 1H, H-22), 6.91 (br.s, 1H, H-18), 6.90 (d, *J* = 8.4 Hz, 1H, H-21), 6.12 (s, 2H, H-6 and H-8), 5.32 (d, *J* = 11.1 Hz, 1H, H-2), 5.22 (d, *J* = 11.1 Hz, 1H, H-3), 4.93 (d, *J* = 8.1 Hz, 1H, H-11), 4.40 (dd, *J* = 11.4, 2.4 Hz, 1H, H-23), 4.27–4.22 (m, 1H, H-10), 4.09 (dd, *J* = 11.7, 3.9 Hz, 1H, H-23), 3.899 (s, 3H, OCH_3_), 3.894 (s, 3H, OCH_3_), 3.888 (s, 3H, OCH_3_), 3.81 (s, 3H, OCH_3_), 3.23 (s, 3H, SO_2_CH_3_), 3.07 (s, 3H, SO_2_CH_3_). ^13^C NMR (75 MHz, CDCl_3_) *δ* 184.48, 167.21, 164.31, 162.78, 150.04, 149.61, 143.74, 143.69, 128.62, 127.42, 121.29, 120.21, 117.47, 117.04, 111.43, 110.02, 104.10, 93.95, 93.87, 80.84, 80.02, 75.79, 75.67, 68.10, 56.43, 56.09 (2 × C), 55.91, 39.76, 37.83. HRMS (ESI): *m*/*z* calculated for C_30_H_33_O_14_S_2_ [M + H]^+^: 681.1312. Found: 681.1310. IR (film) *ν*_max_: 2939, 1688, 1608, 1572, 1509, 1463, 1424, 1356, 1262, 1174, 1160 cm^−1^. HPLC purity 98.7%.

### 3.27. General Procedure for the Synthesis of Carbamoyled Derivatives of 5,7,20-O-Trimethylsilybin A and B

Triethylamine (53 µL, 0.38 mmol) followed by DMAP (12 mg, 0.095 mmol) were added to a solution of 5,7,20-*O*-trimethylsilybin A or 5,7,20-*O*-trimethylsilybin B (50 mg, 0.095 mmol) in DCM (0.95 mL), and the mixture was stirred for 10 min at room temperature before adding the respective (thio)carbamoyl chloride (0.38 mmol). The reaction was allowed to proceed with stirring at room temperature overnight under argon prior to being quenched with ammonium chloride (50 mL). The resulting suspension was extracted with ethyl acetate (30 mL × 3), the organic extracts were combined and dried over anhydrous sodium sulfate, and the organic solvents were removed under vacuo. PTLC purification of the crude product using DCM:MeOH (95:5, *v*/*v*) as eluent to yield the respective 3-carbomoyled derivative and 3,23-dicarbomoyled derivative. Summarized below are their physical and spectral data:

#### 3.27.1. 5,7,20-*O*-Trimethyl-3-*O*-(*N*,*N*-Dimethylcarbamoylsilybin A (**6A**)

Yield, 66%; white solid; [*α*]_D_ +41.34 (*c* 0.60, acetone). ^1^H NMR (300 MHz, CDCl_3_) *δ* 7.16 (d, *J* = 1.8 Hz, 1H, H-13), 7.03–6.95 (overlapped, 4H, H-15, H-16, H-18 and H-22), 6.90 (d, *J* = 8.1 Hz, 1H, H-21), 6.10 (d, *J* = 2.1 Hz, 1H, Ar-H), 6.09 (d, *J* = 2.1 Hz, 1H, Ar-H), 5.51 (d, *J* = 11.8 Hz, 1H, H-3), 5.30 (d, *J* = 11.8 Hz, 1H, H-2), 4.97 (d, *J* = 8.1 Hz, 1H, H-11), 4.06–4.01 (m, 1H, H-10), 3.92 (s, 3H, OCH_3_), 3.91 (s, 3H, OCH_3_), 3.86 (s, 3H, OCH_3_), 3.83–3.78 (overlapped, 1H, H-23), 3.78 (s, 3H, OCH_3_), 3.54 (dd, *J* = 12.6, 4.2 Hz, 1H, H-23), 2.87 (s, 6H, *N*(CH_3_)_2_). ^13^C NMR (75 MHz, CDCl_3_) *δ* 186.48, 166.49, 164.25, 162.47, 155.34, 149.73, 149.51, 144.02, 143.96, 129.71, 128.51, 120.89, 120.25, 117.09, 116.59, 111.22, 109.96, 104.32, 93.48 (2 × C), 81.05, 78.38, 76.37, 74.73, 61.79, 56.18 (2 × C), 56.14, 55.76, 36.85, 36.16. HRMS (ESI): *m*/*z* calculated for C_31_H_34_NO_11_ [M + H]^+^: 596.2132. Found: 596.2129. IR (film) *ν*_max_: 3502, 2935, 1693, 1609, 1573, 1509, 1457, 1423, 1395, 1264, 1159, 1110 cm^−1^. HPLC purity 96.0%.

#### 3.27.2. 5,7,20-*O*-Trimethyl-3-*O*-(*N*,*N*-Dimethylcarbamoyl-Silybin B (**6B**)

Yield, 62%; white foam; [*α*]_D_ +26.70 (*c* 1.19, acetone). ^1^H NMR (300 MHz, CDCl_3_) *δ* 7.15 (d, *J* = 1.8 Hz, 1H, H-13), 7.04–6.93 (overlapped, 4H, H-15, H-16, H-18 and H-22), 6.90 (d, *J* = 8.4 Hz, 1H, H-21), 6.11 (d, *J* = 2.4 Hz, 1H, H-6), 6.08 (d, *J* = 2.1 Hz, 1H, H-8), 5.53 (d, *J* = 11.8 Hz, 1H, H-3), 5.31 (d, *J* = 11.8 Hz, 1H, H-2), 4.96 (d, *J* = 8.4 Hz, 1H, H-11), 4.08–4.03 (m, 1H, H-10), 3.90 (s, 6H, 2 × OCH_3_), 3.86 (s, 3H, OCH_3_), 3.84–3.77 (overlapped, 1H, H-23), 3.80 (s, 3H, OCH_3_), 3.54 (dd, *J* = 12.3, 3.9 Hz, 1H, H-23), 2.87 (s, 6H, *N*(CH_3_)_2_). ^13^C NMR (75 MHz, CDCl_3_) *δ* 186.49, 166.47, 164.21, 162.46, 155.30, 149.73, 149.44, 144.05, 144.01, 129.56, 128.54, 121.04, 120.22, 117.17, 116.57, 111.28, 110.13, 104.35, 93.50 (2 × C), 80.95, 78.35, 76.29, 74.57, 61.78, 56.10 (3 × C), 55.77, 36.84, 36.16. HRMS (ESI): *m*/*z* calculated for C_31_H_34_NO_11_ [M + H]^+^: 596.2132. Found: 596.2133. IR (film) *ν*_max_: 3486, 2934, 1690, 1607, 1572, 1509, 1456, 1422, 1394, 1263, 1158, 1109 cm^−1^. HPLC purity 95.2%.

#### 3.27.3. 5,7,20-*O*-Trimethyl-3,23-*O*-di(*N*,*N*-Dimethylcarbamoyl)Silybin A (**7A**)

Yield, 25%; colorless syrup; [*α*]_D_ +55.59 (*c* 0.34, acetone). ^1^H NMR (300 MHz, CDCl_3_) *δ* 7.16 (br.s, 1H, H-13), 7.00–6.90 (overlapped, 4H, H-15, H-16, H-18 and H-22), 6.88 (d, *J* = 8.1 Hz, 1H, H-21), 6.09 (s, 2H, H-6 and H-8), 5.51 (d, *J* = 11.8 Hz, 1H, H-3), 5.30 (d, *J* = 11.8 Hz, 1H, H-2), 4.90 (d, *J* = 8.1 Hz, 1H, H-11), 4.34 (dd, *J* = 12.0, 3.3 Hz, 1H, H-23), 4.27–4.22 (m, 1H, H-10), 3.96 (dd, *J* = 12.0, 4.2 Hz, 1H, H-23), 3.90 (s, 3H, OCH_3_), 3.89 (s, 3H, OCH_3_), 3.86 (s, 3H, OCH_3_), 3.80 (s, 3H, OCH_3_), 2.88 (s, 12H, 2 × *N*(CH_3_)_2_). ^13^C NMR (75 MHz, CDCl_3_) *δ* 186.46, 166.49, 164.25, 162.48, 155.91, 155.36, 149.85, 149.56, 144.00, 143.84, 129.64, 128.30, 121.07, 120.22, 117.23, 116.40, 111.31, 109.94, 104.33, 93.51, 93.44, 81.05, 76.87, 76.21, 74.75, 63.95, 56.18, 56.09 (2 × C), 55.77, 36.84, 36.65, 36.16, 36.04. HRMS (ESI): *m*/*z* calculated for C_34_H_39_N_2_O_12_ [M + H]^+^: 667.2503. Found: 667.2499. IR (film) *ν*_max_: 2933, 1701, 1608, 1573, 1509, 1456, 1395, 1265, 1184, 1158 cm^−1^. HPLC purity 95.5%.

#### 3.27.4. 5,7,20-*O*-Trimethyl-3,23-*O*-di(*N*,*N*-Dimethylcarbamoyl)Silybin B (**7B**)

Yield, 28%; colorless syrup; [*α*]_D_ +0.97 (*c* 0.31, acetone). ^1^H NMR (300 MHz, CDCl_3_) *δ* 7.15 (br.s, 1H, H-13), 7.04–6.88 (overlapped, 5H, H-15, H-16, H-18, H-21 and H-22), 6.11 (d, *J* = 2.1 Hz, 1H, H-6), 6.09 (d, *J* = 2.1 Hz, 1H, H-8), 5.53 (d, *J* = 11.7 Hz, 1H, H-3), 5.31 (d, *J* = 11.7 Hz, 1H, H-2), 4.91 (d, *J* = 8.1 Hz, 1H, H-11), 4.34 (dd, *J* = 11.8, 3.3 Hz, 1H, H-23), 4.30–4.25 (m, 1H, H-10), 3.97 (dd, *J* = 11.8, 4.2 Hz, 1H, H-23), 3.90 (s, 3H, OCH_3_), 3.89 (s, 3H, OCH_3_), 3.86 (s, 3H, OCH_3_), 3.81 (s, 3H, OCH_3_), 2.88 (s, 12H, 2 × *N*(CH_3_)_2_). ^13^C NMR (75 MHz, CDCl_3_) *δ* 186.48, 166.50, 164.24, 162.50, 155.93, 155.34, 149.88, 149.51, 144.01, 143.89, 129.53, 128.33, 121.08, 120.24, 117.35, 116.53, 111.39, 110.11, 104.39, 93.47 (2 × C), 80.96, 77.37, 76.14, 74.63, 64.01, 56.08 (3 × C), 55.80, 36.86, 36.64, 36.16, 36.06. HRMS (ESI): *m*/*z* calculated for C_34_H_39_N_2_O_12_ [M + H]^+^: 667.2503. Found: 667.2501. IR (film) ν_max_: 2936, 1704, 1608, 1573, 1509, 1456, 1423, 1396, 1265, 1185, 1159, 1110 cm^−1^. HPLC purity 98.5%.

#### 3.27.5. 3-*O*-(*N*,*N*-Diethylcarbamoyl)-5,7,20-*O*-Trimethylsilybin A (**8A**)

Yield, 35%; slight yellow solid; [*α*]_D_ +35.7 (*c* 0.47, acetone). ^1^H NMR (300 MHz, CDCl_3_) *δ* 7.17 (br.s, 1H, H-13), 7.02–6.95 (overlapped, 4H, H-15, H-16, H-18 and H-22), 6.90 (d, *J* = 8.1 Hz, 1H, H-21), 6.10 (d, *J* = 2.1 Hz, 1H, H-6), 6.09 (d, *J* = 2.1 Hz, 1H, H-8), 5.55 (d, *J* = 11.9 Hz, 1H, H-3), 5.29 (d, *J* = 11.9 Hz, 1H, H-2), 4.96 (d, *J* = 8.4 Hz, 1H, H-11), 4.06–4.01 (m, 1H, H-10), 3.92 (s, 3H, OCH_3_), 3.90 (s, 3H, OCH_3_), 3.86 (s, 3H, OCH_3_), 3.86–3.76 (overlapped, 1H, H-23), 3.80 (s, 3H, OCH_3_), 3.62–3.49 (m, 1H, H-23), 3.30–3.10 (m, 4H, *N*(*CH_2_*CH_3_)_2_), 2.00 (br.s, 1H, OH), 1.15–0.95 (m, 6H, *N*(CH_2_*CH_3_*)_2_). ^13^C NMR (75 MHz, CDCl_3_) *δ* 186.48, 166.39, 164.22, 162.44, 154.54, 149.76, 149.54, 144.00, 143.96, 129.75, 128.57, 120.96, 120.25, 117.02, 116.60, 111.27, 110.04, 104.43, 93.48 (2 × C), 81.18, 78.39, 76.39, 74.40, 61.78, 56.15 (3 × C), 55.78, 42.30, 41.58, 13.92, 13.57. HRMS (ESI): *m*/*z* calculated for C_33_H_38_NO_11_ [M + H]^+^: 624.2445. Found: 624.2440. IR (film) *ν*_max_: 3481, 2935, 1689, 1609, 1573, 1509, 1462, 1423, 1274, 1239, 1216, 1159 cm^−1^. HPLC purity 98.5%.

#### 3.27.6. 3-*O*-(*N*,*N*-Diethylcarbamoyl)-5,7,20-*O*-Trimethylsilybin B (**8B**)

Yield, 33%; slight yellow solid; [*α*]_D_ +20.19 (*c* 0.53, acetone). ^1^H NMR (300 MHz, CDCl_3_) *δ* 7.14 (d, *J* = 1.8 Hz, 1H, H-13), 7.03–6.96 (overlapped, 3H, H-15, H-16 and H-22), 6.92 (d, *J* = 2.1 Hz, 1H, H-18), 6.89 (d, *J* = 8.1 Hz, 1H, H-21), 6.11 (d, *J* = 2.4 Hz, 1H, H-6), 6.09 (d, *J* = 2.4 Hz, 1H, H-8), 5.55 (d, *J* = 11.8 Hz, 1H, H-3), 5.30 (d, *J* = 11.8 Hz, 1H, H-2), 4.96 (d, *J* = 8.4 Hz, 1H, H-11), 4.06–4.01 (m, 1H, H-10), 3.90 (s, 6H, 2 × OCH_3_), 3.86 (s, 3H, OCH_3_), 3.81 (dd, *J* = 12.3, 2.7 Hz, 1H, H-23), 3.80 (s, 3H, OCH_3_), 3.54 (dd, *J* = 12.3, 3.9 Hz, 1H, H-23), 3.33–3.10 (m, 4H, *N*(*CH_2_*CH_3_)_2_), 1.06 (t, *J* = 7.2 Hz, 6H, *N*(CH_2_*CH_3_*)_2_). ^13^C NMR (75 MHz, CDCl_3_) *δ* 186.47, 166.37, 164.18, 162.43, 154.45, 149.72, 149.43, 143.99, 143.94, 129.61, 128.59, 120.95, 120.19, 117.14, 116.56, 111.31, 110.15, 104.44, 93.49 (2 × C), 81.07, 78.37, 76.26, 74.30, 61.74, 56.20, 56.08, 56.03, 55.76, 41.59 (2 × C), 13.48 (2 × C). HRMS (ESI): *m*/*z* calculated for C_33_H_38_NO_11_ [M + H]^+^: 624.2445. Found: 624.2441. IR (film) *ν*_max_: 3480, 2935, 1690, 1609, 1573, 1509, 1460, 1423, 1275, 1159 cm^−1^. HPLC purity 98.2%.

#### 3.27.7. 5,7,20-*O*-Trimethyl-3-*O*-(*N*,*N*-Dimethylthiocarbamoyl)Silybin A (**9A**)

Yield, 23%; slight yellow solid; [*α*]_D_ +20.7 (*c* 0.30, acetone). ^1^H NMR (300 MHz, CDCl_3_) *δ* 7.17 (d, *J* = 2.1 Hz, 1H, H-13), 7.06 (dd, *J* = 8.4, 2.1 Hz, 1H, H-15), 6.98 (d, *J* = 8.4 Hz, 2H, H-16 and H-22), 6.93 (d, *J* = 1.8 Hz, 1H, H-18), 6.90 (d, *J* = 8.1 Hz, 1H, H-21), 6.58 (d, *J* = 11.4 Hz, 1H, H-3), 6.10 (d, *J* = 2.4 Hz, 1H, Ar-H), 6.09 (d, *J* = 2.4 Hz, 1H, Ar-H), 5.36 (d, *J* = 11.4 Hz, 1H, H-2), 4.96 (d, *J* = 8.4 Hz, 1H, H-11), 4.08–4.03 (m, 1H, H-10), 4.08 (s, 3H, OCH_3_), 4.07 (s, 3H, OCH_3_), 4.06 (s, 3H, OCH_3_), 4.04 (s, 3H, OCH_3_), 4.05–4.03 (overlapped, 1H, H-23), 3.54 (dd, *J* = 12.6, 4.2 Hz, 1H, H-23), 3.33 (s, 3H, *N*CH_3_), 3.10 (s, 3H, *N*CH_3_). ^13^C NMR (75 MHz, CDCl_3_) *δ* 187.28 (2 × C), 166.40, 163.73, 162.30, 149.71, 149.41, 144.18, 144.01, 128.88, 128.49, 121.23, 120.22, 117.27, 116.89, 111.27, 110.12, 104.34, 93.48 (2 × C), 80.93, 79.43, 78.29, 76.26, 61.74, 56.22, 56.11, 56.03, 55.82, 43.39, 38.37. HRMS (ESI): *m*/*z* calculated for C_31_H_34_NO_10_S [M + H]^+^: 612.1904. Found: 612.1902. IR (film) *ν*_max_: 3484, 2937, 1689, 1609, 1573, 1509, 1158 cm^−1^. HPLC purity 97.1%.

#### 3.27.8. 5,7,20-*O*-Trimethyl-3-*O*-(*N*,*N*-Dimethylthiocarbamoyl)Silybin B (**9B**)

Yield, 26%; slight yellow solid; [*α*]_D_ +14.8 (*c* 0.29, acetone). ^1^H NMR (300 MHz, CDCl_3_) *δ* 7.15 (d, *J* = 1.8 Hz, 1H, H-13), 7.08 (dd, *J* = 8.4, 2.1 Hz, 1H, H-15), 7.02–6.97 (overlapped, 2H, H-16 and H-22), 6.94 (d, *J* = 1.8 Hz, 1H, H-18), 6.90 (d, *J* = 8.4 Hz, 1H, H-21), 6.56 (d, *J* = 11.6 Hz, 1H, H-3), 6.11 (d, *J* = 2.1 Hz, 1H, H-6), 6.09 (d, *J* = 2.1 Hz, 1H, H-8), 5.35 (d, *J* = 11.6 Hz, 1H, H-2), 4.96 (d, *J* = 8.4 Hz, 1H, H-11), 4.07–4.02 (m, 1H, H-10), 3.91 (s, 3H, OCH_3_), 3.90 (s, 3H, OCH_3_), 3.86 (s, 3H, OCH_3_), 3.80 (s, 3H, OCH_3_),3.80 (dd, *J* = 12.3, 2.7 Hz, 1H, H-23), 3.54 (dd, *J* = 12.3, 3.9 Hz, 1H, H-23), 3.33 (s, 3H, *N*CH_3_), 3.09 (s, 3H, *N*CH_3_). ^13^C NMR (75 MHz, CDCl_3_) *δ* 187.24 (2 × C), 166.38, 163.73, 162.29, 149.73, 149.48, 144.15, 143.92, 128.98, 128.47, 121.01, 120.25, 117.27, 117.00, 111.25, 110.02, 104.35, 93.48 (2 × C), 80.99, 78.36, 77.36, 76.35, 61.73, 56.20, 56.14, 56.08, 55.79, 43.40, 38.41. HRMS (ESI): *m*/*z* calculated for C_31_H_34_NO_10_S [M + H]^+^: 612.1904. Found: 612.1904. IR (film) ν_max_: 3503, 2937, 1689, 1573, 1509, 1463, 1158 cm^−1^. HPLC purity 100.0%.

### 3.28. Cell Culture

All prostate cancer cell lines were originally purchased from American Type Culture Collection (ATCC). The PC-3, LNCaP, and 22Rv1 prostate cancer cells were routinely cultured in RPMI-1640 medium supplemented with 10% FBS and 1% penicillin/streptomycin. The DU145 prostate cancer cell line was routinely cultured in Eagle’s Minimum Essential Medium (EMEM) supplemented with 10% FBS and 1% penicillin/streptomycin. Cultures were maintained in a high humidity environment supplemented with 5% carbon dioxide at a temperature of 37 °C.

### 3.29. WST-1 Cell Proliferation Assay

PC-3, DU145, and LNCaP cells were placed in 96-well plates at a density of 3200 each well in 200 µL of culture medium. The 22Rv1 cells were placed in 96-well plates at a density of 6400 each well in 200 µL of culture medium. The cells were cultured for 16 h and then treated with enzalutamide or silibinin (both as positive controls) or synthesized derivatives at five different doses for 3 days. Equal treatment volumes (200 µL) of DMSO (0.25%) in medium were used as vehicle control. The cells were further cultured in a CO_2_ incubator at 37 °C for three days. Ten µL of the premixed WST-1 cell proliferation reagent (Takara Bio USA, Inc., San Jose, CA, USA) was added to each well. After mixing gently for 1 min on an orbital shaker, the cells were cultured for an additional 3 h at 37 °C. The absorbance of each well was measured using a microplate reader (Synergy HT, BioTek) at a wavelength of 430 nm. The IC_50_ value is the concentration of each derivative that inhibits cell proliferation by 50% under the experimental conditions and is the average from triplicate determinations that were reproducible and statistically significant. For calculating each IC_50_ value, a linear proliferative inhibition was made based on at least five dosages for each compound.

## 4. Conclusions

**Figure 10:**
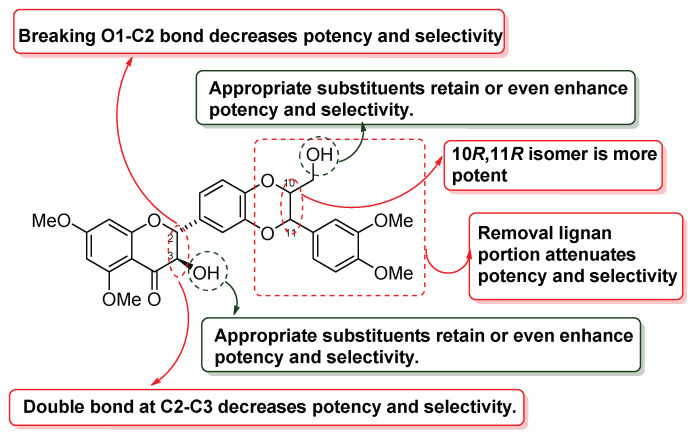
Structure-Activity Relationships of 5,7,20-*O*-trimethylsilibinins.

By comparing with 5,7,20-*O*-hydnocarpin Ds, chalcone-type flavonolignans, and taxifolin derivatives, 5,7,20-*O*-trimethylsilybin and its derivatives possess the highest potency and selectivity towards AR-positive LNCaP prostate cancer cell line. Our data indicate that 5,7,20-*O*-trimethylsilybins are the most promising scaffold for AR modulation among the four and that the appropriate modification of the alcoholic hydroxyl group at C-3 and/or C-23 can retain or even enhance the antiproliferative potency in the AR-positive LNCaP cell model. The most promising 5,7,20-*O*-trimethylsilybins were further studied on the antiproliferative potency of their optically pure versions. To this end, our data show that (10*R*,11*R*) derivatives (silybin A series) are more potent than (10*S*,11*S*) derivatives (silybin B series) in the AR-positive LNCaP cell model. The detailed structure–activity relationships among four core structures and diastereomers were illustrated in Figure 10. Two (10*R*,11*R*) derivatives **8A** and **41A** were established as the optimal lead compounds because they can selectively inhibit AR-positive LNCaP cell proliferation with IC_50_ value of 0.07 µM. The fact that **8A** and **41A** cannot suppress AR-null PC-3 and DU145 prostate cancer cell proliferation suggests their antiproliferative activity in the AR-positive LNCaP cell model may be associated with AR. Even though both LNCaP and 22Rv1 are AR-positive prostate cancer cell lines, they bear one critical difference. LNCaP possesses full-length AR that contains four domains: *N*-terminal domain, DNA-binding domain, *C*-terminal ligand binding domain (LBD), and the flexible hinge region. In contrast, 22Rv1 contains both full-length AR and LBD-truncated AR-V7. (10*R*,11*R*) Derivatives **8A** and **41A** cannot suppress 22Rv1 prostate cancer cell proliferation up to 10 μM concentration, suggesting that they are very likely binding to the ligand-binding domain on AR to exhibit antiproliferative activity in LNCaP cells. This assumption needs to be confirmed by further investigation on the optimal compounds **8A** and **41A**. Our findings warrant the further exploration of (10*R*,11*R*) 3-*O*-substituted-5,7,20-*O*-trimethylsilybin as a new scaffold of androgen receptor modulators.

## Figures and Tables

**Table 1:** Antiproliferative activity of 5,7,20-*O*-trimethylsilybins **5**–**16**.

Compound	IC_50_ (µM) ^1^		
	PC-3 ^2^	DU145 ^3^	LNCaP ^4^
**Silibinin (1)**	72.65 ± 3.15	93.34 ± 13.76	43.73 ± 10.90
**Enzalutamide**	>25	>25	40–60% inhibition at 25 µM
**5** ^5^	7.41 ± 1.30	22.41 ± 0.87	0.41 ± 0.08
**6** ^5^	12.49 ± 1.91	>25	0.83 ± 0.04
**7** ^5^	17.83 ± 1.09	>25	0.25 ± 0.04
**8** ^5^	> 25	>25	0.11 ± 0.03
**9**	> 25	>25	0.82 ± 0.03
**10**	6.45 ± 0.43	>25	0.51 ± 0.08
**11**	8.22 ± 1.33	14.05 ± 3.46	0.56 ± 0.06
**12**	>25	13.18 ± 0.92	24.52 ± 3.29
**13**	>25	13.55 ± 1.96	14.64 ± 3.65
**14**	>25	>25	17.92 ± 2.66
**15**	18.41 ± 1.49	>25	0.75 ± 0.17
**16**	4.50 ± 0.31	15.98 ± 2.45	0.60 ± 0.08

^1^ IC_50_ is the compound concentration effective in inhibiting 50% cell proliferation measured by WST-1 cell proliferation assay after 3-day exposure. The data were presented as the mean ± SD from n = 3. ^2^ Human androgen receptor-negative prostate cancer cell line derived from bone metastasis of prostate tumor. ^3^ Human androgen receptor-negative prostate cancer cell line derived from brain metastasis of prostate tumor. ^4^ Human androgen receptor-positive prostate cancer cell line derived from lymph node metastasis of prostate tumor. ^5^ These data have been reported in our previous paper [16].

**Table 2:** Antiproliferative activity of 5,7,20-*O*-trimethylhydnocarpins **17**–**22**.

Compd	IC_50_ (µM) ^1^		
	PC-3 ^2^	DU145 ^3^	LNCaP ^4^
**Silibinin**	72.65 ± 3.15	93.34 ± 13.76	43.74 ± 10.90
**Enzalutamide**	>25	>25	40–60% inhibition at 25 µM
**17**	>25	>25	2.84 ± 0.45
**18**	>25	>25	>25
**19**	>25	>25	>25
**20**	>25	>25	>25
**21**	>25	>25	>25
**22**	>25	>25	>25

^1^ IC_50_ is the compound concentration effective in inhibiting 50% cell proliferation measured by WST-1 cell proliferation assay after 3-day exposure. The data were presented as the mean ± SD from n = 3. ^2^ Human androgen receptor-negative prostate cancer cell line derived from bone metastasis of prostate tumor. ^3^ Human androgen receptor-negative prostate cancer cell line derived from brain metastasis of prostate tumor. ^4^ Human androgen receptor-positive prostate cancer cell line derived from lymph node metastasis of prostate tumor.

**Table 3:** Antiproliferative activity of chalcone-type flavonolignans.

Compd	IC_50_ (µM) ^1^		
	PC-3 ^2^	DU145 ^3^	LNCaP ^4^
**Silibinin**	72.65 ± 3.15	93.34 ± 13.76	43.74 ± 10.90
**Enzalutamide**	>25	>25	40–60% inhibition at 25 µM
**23**	>25	>25	>25
**24**	>25	7.93 ± 2.23	2.99 ± 0.25
**25**	>25	>25	>25
**26**	8.03 ± 0.32	8.97 ± 0.14	6.84 ± 0.11
**27**	5.67 ± 1.36	2.60 ± 0.33	3.63 ± 0.66
**28**	>25	>25	>25
**29**	>25	>25	8.48 ± 1.44
**30**	>25	>25	4.92 ± 0.72
**31**	>25	>25	>25
**32**	24.74 ± 0.62	13.30 ± 1.62	16.33 ± 1.32
**33**	>25	>25	3.29 ± 1.39
**34**	3.53 ± 0.31	3.28 ± 0.98	6.12 ± 0.11
**35**	4.95 ± 1.37	2.96 ± 0.23	5.66 ± 0.37

^1^ IC_50_ is the compound concentration effective in inhibiting 50% cell proliferation measured by WST-1 cell proliferation assay after 3-day exposure. The data were presented as the mean ± SD from n = 3. ^2^ Human androgen receptor-negative prostate cancer cell line derived from bone metastasis of prostate tumor. ^3^ Human androgen receptor-negative prostate cancer cell line derived from brain metastasis of prostate tumor. ^4^ Human androgen receptor-positive prostate cancer cell line derived from lymph node metastasis of prostate tumor.

**Table 4:** Antiproliferative activity of taxifolins.

Compound	IC_50_ (µM) ^1^		
	PC-3 ^2^	DU145 ^3^	LNCaP ^4^
**Silibinin**	72.65 ± 3.15	93.34 ± 13.76	43.73 ± 10.90
**Enzalutamide**	>25	>25	40–60% inhibition at 25 µM
**36**	>25	>25	>25
**37**	>25	>25	>25
**38**	>25	>25	>25
**39**	>25	>25	>25

^1^ IC_50_ is the compound concentration effective in inhibiting 50% cell proliferation measured by WST-1 cell proliferation assay after 3-day exposure. The data were presented as the mean ± SD from n = 3. ^2^ Human androgen receptor-negative prostate cancer cell line derived from bone metastasis of prostate tumor. ^3^ Human androgen receptor-negative prostate cancer cell line derived from brain metastasis of prostate tumor. ^4^ Human androgen receptor-positive prostate cancer cell line derived from lymph node metastasis of prostate tumor.

**Table 5:** Specific rotations of silybin A and B, and 23-*O*-acetylsilybin A and B.

Compd	Literature Reported [*α*]_D_	Our Measured [*α*]_D_
Silybin A (**1A**)	+16.33 (*c* 0.3, acetone) [35]	+15.14 (*c* 0.7, acetone)
Silybin B (**1B**)	+3.96 (*c* 0.3, acetone) [35]	+2.28 (*c* 2.0, acetone)
23-*O*-acetylsilybin (**40**)	+10.40 (*c* 0.3, acetone) [35]	+7.76 (*c* 0.8, acetone)
23-*O*-acetylsilybin A (**40A**)	+39.60 (*c* 0.3, acetone) [35]	+34.00 (*c* 0.7, acetone)
23-*O*-acetylsilybin B (**40B**)	−16.2 (*c* 0.3, acetone) [35]	−13.62 (*c* 1.0, acetone)

**Table 6:** Antiproliferative activity of optically enriched trimethylsilybins.

Compd	IC_50_ (µM) ^1^			
	PC-3 ^2^	DU145 ^3^	LNCaP ^4^	22Rv1 ^5^
**Enzalutamide**	>25	>25	40–60% inhibition at 25 µM	
**1A**	50.52 ± 7.74	98.97 ± 12.55	39.96 ± 2.70	92.54 ± 2.13
**1B**	29.41 ± 2.08	75.85 ± 4.48	33.52 ± 5.42	90.43 ± 8.00
**40A**	>10	>10	>10	>10
**40B**	>10	>10	>10	>10
**41A**	>10	>10	0.07 ± 0.02	>10
**41B**	>10	>10	1.84 ± 0.43	>10
**5A**	11.08 ± 2.71	>10	0.15 ± 0.06	>10
**5B**	2.71 ± 0.41	>10	1.77 ± 0.49	>10
**6A**	>10	>10	0.54 ± 0.05	>10
**6B**	>10	>10	3.87 ± 1.13	>10
**7A**	>10	>10	0.42 ± 0.14	>10
**7B**	>10	>10	1.96 ± 0.10	>10
**8A**	>10	>10	0.07 ± 0.01	>10
**8B**	>10	>10	3.00 ± 0.26	>10
**9A**	>10	>10	0.53 ± 0.21	>10
**9B**	>10	>10	2.77 ± 0.88	>10
**10A**	6.65 ± 0.24	>10	0.35 ± 0.08	>10
**10B**	1.58 ± 0.14	>10	1.26 ± 0.01	>10
**11A**	>10	>10	0.31 ± 0.04	>10
**11B**	>10	>10	10.55 ± 0.43	>10

^1^ IC_50_ is the compound concentration effective in inhibiting 50% cell proliferation measured by WST-1 cell proliferation assay after 3-day exposure. The data were presented as the mean ± SD from n = 3. ^2^ Human AR-negative prostate cancer cell line derived from bone metastasis of prostate tumor. ^3^ Human AR-negative prostate cancer cell line derived from brain metastasis of prostate tumor. ^4^ Human AR-positive prostate cancer cell line derived from lymph node metastasis of prostate tumor. ^5^ Human AR-positive prostate cancer cell line derived from a castration-resistant xenograft.

## Data Availability

All critical data have been included in the Supplementary Materials.

## Supplementary Materials

The following supporting information can be downloaded at: https://www.mdpi.com/article/10.3390/ph16040531/s1, Figures S1, S2, S5, S6, S9, S10, S13, S14, S17, S18, S21, S22, S25, S26, S29, S30, S33, S34, S37, S38, S41, S42, S45, S46, S49, S50, S53, S54, S57, S58, S61, S62, S65, S66, S69, S70, S73, S74, S77, S78, S81, S82, S85, S86, S89, S90, S93, S94, S97, S98, S101, S102, S105, S106, S109, S110, S113, S114, S117, S118, S121, S122, S125, S126, S129, S130, S133, S134, S137, S138, S141, S142, S145, S146, S149, S150, S153, S154, S157, S158, S161, S162, S165, S166, S169, S170, S171: NMR spectra; Figures S3, S7, S11, S15, S19, S23, S27, S31, S35, S39, S43, S47, S51, S55, S59, S63, S67, S71, S75, S79, S83, S87, S91, S95, S99, S103, S107, S111, S115, S119, S123, S127, S131, S135, S139, S143, S147, S151, S155, S159, S163, S167: High resolution mass spectra; Figures S4, S8, S12, S16, S20, S24, S28, S32, S36, S40, S44, S48, S52, S56, S60, S64, S68, S72, S76, S80, S84, S88, S92, S96, S100, S104, S108, S112, S116, S120, S124, S128, S132, S136, S140, S144, S148, S152, S156, S160, S164, S168: HPLC chromatograms.
