# Supplementary material for: Core Structure–Activity Relationship Studies of 5,7,20-O-Trimethylsilybins in Prostate Cancer Cell Models
Source: Pharmaceuticals (Basel). 2023 Apr 2;16(4):531. doi: 10.3390/ph16040531 (PMC10145751; doi:10.3390/ph16040531)

Electronic Supplementary Information

Core Structure-Activity Relationship Studies of 5,7,20-Trimethylsilybins in Prostate Cancer Cell Models

Sitong Wu [1], Guanglin Chen [1], Eva Y. Chen [1], Leyla S. Farshidpour [1], Qiang Zhang [2], Guangdi Wang [2] and Qiao-Hong Chen [1,*]

[1] Department of Chemistry and Biochemistry, California State University, Fresno, 2555 E. San Ramon Avenue, M/S SB70, Fresno, CA 93740, USA
[2] Department of Chemistry and RCMI Cancer Research Center, Xavier University of Louisiana, 1 Drexel Drive, New Orleans, LA 70125, USA
* Correspondence: qchen@csufresno.edu

List of Contents:

Figure S1: ^{1}H NMR spectrum of **10** in $CDCl_3$……S7
Figure S2: ^{13}C NMR spectrum of **10** in $CDCl_3$……S8
Figure S3: High resolution mass spectrum of **10**…… S9
Figure S4: HPLC chromatogram of **10**……S10
Figure S5: ^{1}H NMR spectrum of **11** in $CDCl_3$……S11
Figure S6: ^{13}C NMR spectrum of **11** in $CDCl_3$……S12
Figure S7: High resolution mass spectrum of **11**……S13
Figure S8: HPLC chromatogram of **11**……S14
Figure S9: ^{1}H NMR spectrum of **12** in $CDCl_3$……S15
Figure S10: ^{13}C NMR spectrum of **12** in $CDCl_3$……S16
Figure S11: High resolution mass spectrum of **12**……S17
Figure S12: HPLC chromatogram of **12**……S18
Figure S13: ^{1}H NMR spectrum of **13** in $CDCl_3$……S19
Figure S14: ^{13}C NMR spectrum of **13** in $CDCl_3$……S20
Figure S15: High resolution mass spectrum of **13**……S21
Figure S16: HPLC chromatogram of **13**……S22
Figure S17: ^{1}H NMR spectrum of **14** in $CDCl_3$……S23
Figure S18: ^{13}C NMR spectrum of **14** in $CDCl_3$……S24
Figure S19: High resolution mass spectrum of **14**……S25
Figure S20: HPLC chromatogram of **14**……S26

Figure S21: ^{1}H NMR spectrum of **15** in $CDCl_3$……………………………………………………………S27
Figure S22: ^{13}C NMR spectrum of **15** in $CDCl_3$……………………………………………………………S28
Figure S23: High resolution mass spectrum of **15**……………………………………………………...S29
Figure S24: HPLC chromatogram of **15**……………………………………………………………………S30
Figure S25: ^{1}H NMR spectrum of **16** in $CDCl_3$……………………………………………………………S31
Figure S26: ^{13}C NMR spectrum of **16** in $CDCl_3$……………………………………………………………S32
Figure S27: High resolution mass spectrum of **16**……………………………………………………...S33
Figure S28: HPLC chromatogram of **16**……………………………………………………………………S34
Figure S29: ^{1}H NMR spectrum of **17** in $CDCl_3$……………………………………………………………S35
Figure S30: ^{13}C NMR spectrum of **17** in $CDCl_3$/DMSO-d_6 (10:1, v/v)…..……………………………S36
Figure S31: High resolution mass spectrum of **17**………………………………………………………S37
Figure S32: HPLC chromatogram of **17**……………………………………………………………………S38
Figure S33: ^{1}H NMR spectrum of **18** in $CDCl_3$……………………..………………………………...S39
Figure S34: ^{13}C NMR spectrum of **18** in $CDCl_3$……………………………………………………………S40
Figure S35: High resolution mass spectrum of **18**………………………………………………………S41
Figure S36: HPLC chromatogram of **18**……………………………………………………………………..S42
Figure S37: ^{1}H NMR spectrum of **19** in $CDCl_3$……………………………………………………………S43
Figure S38: ^{13}C NMR spectrum of **19** in $CDCl_3$……………………………………………………………S44
Figure S39: High resolution mass spectrum of **19**………………………………………………………..S45
Figure S40: HPLC chromatogram of **19**……………………………………………………………………S46
Figure S41: ^{1}H NMR spectrum of **20** in $CDCl_3$……………………………………………………………S47
Figure S42: ^{13}C NMR spectrum of **20** in $CDCl_3$……………………………………………………………S48
Figure S43: High resolution mass spectrum of **20**………………………………………………………..S49
Figure S44: HPLC chromatogram of **20**……………………………………………………………………S50
Figure S45: ^{1}H NMR spectrum of **21** in $CDCl_3$……………………………………………………………S51
Figure S46: ^{13}C NMR spectrum of **21** in $CDCl_3$……………………………………………………………S52
Figure S47: High resolution mass spectrum of **21**………………………………………………………..S53
Figure S48: HPLC chromatogram of **21**……………………………………………………………………S54
Figure S49: ^{1}H NMR spectrum of **22** in $CDCl_3$……………………………………………………………..S55
Figure S50: ^{13}C NMR spectrum of **22** in $CDCl_3$……………………………………………………………S56
Figure S51: High resolution mass spectrum of **22**………………………………………………………S57
Figure S52: HPLC chromatogram of **22**……………………………………………………………………S58
Figure S53: ^{1}H NMR spectrum of **23** in $CDCl_3$……………………………………………………………..S59
Figure S54: ^{13}C NMR spectrum of **23** in $CDCl_3$……………………………………………………………S60
Figure S55: High resolution mass spectrum of **23**………………………………………………………S61
Figure S56: HPLC chromatogram of **23**……………………………………………………………………S62

Figure S57: ^{1}H NMR spectrum of **24** in $CDCl_3$…………S63
Figure S58: ^{13}C NMR spectrum of **24** in $CDCl_3$…………S64
Figure S59: High resolution mass spectrum of **24**…………S65
Figure S60: HPLC chromatogram of **24**…………S66
Figure S61: ^{1}H NMR spectrum of **25** in $CDCl_3$…………S67
Figure S62: ^{13}C NMR spectrum of **25** in $CDCl_3$…………S68
Figure S63: High resolution mass spectrum of **25**…………S69
Figure S64: HPLC chromatogram of **25**…………S70
Figure S65: ^{1}H NMR spectrum of **26** in $CDCl_3$…………S71
Figure S66: ^{13}C NMR spectrum of **26** in $CDCl_3$…………S72
Figure S67: High resolution mass spectrum of **26**…………S73
Figure S68: HPLC chromatogram of **26**…………S74
Figure S69: ^{1}H NMR spectrum of **27** in $CDCl_3$…………S75
Figure S70: ^{13}C NMR spectrum of **27** in $CDCl_3$…………S76
Figure S71: High resolution mass spectrum of **27**…………S77
Figure S72: HPLC chromatogram of **27**…………S78
Figure S73: ^{1}H NMR spectrum of **28** in $CDCl_3$…………S79
Figure S74: ^{13}C NMR spectrum of **28** in $CDCl_3$…………S80
Figure S75: High resolution mass spectrum of **28**…………S81
Figure S76: HPLC chromatogram of **28**…………S82
Figure S77: ^{1}H NMR spectrum of **29** in $CDCl_3$…………S83
Figure S78: ^{13}C NMR spectrum of **29** in $CDCl_3$…………S84
Figure S79: High resolution mass spectrum of **29**…………S85
Figure S80: HPLC chromatogram of **29**…………S86
Figure S81: ^{1}H NMR spectrum of **30** in $CDCl_3$…………S87
Figure S82: ^{13}C NMR spectrum of **30** in $CDCl_3$…………S88
Figure S83: High resolution mass spectrum of **30**…………S89
Figure S84: HPLC chromatogram of **30**…………S90
Figure S85: ^{1}H NMR spectrum of **31** in $CDCl_3$…………S91
Figure S86: ^{13}C NMR spectrum of **31** in $CDCl_3$…………S92
Figure S87: High resolution mass spectrum of **31**…………S93
Figure S88: HPLC chromatogram of **31**…………S94
Figure S89: ^{1}H NMR spectrum of **32** in $CDCl_3$…………S95
Figure S90: ^{13}C NMR spectrum of **32** in $CDCl_3$…………S96
Figure S91: High resolution mass spectrum of **32**…………S97
Figure S92: HPLC chromatogram of **32**…………S98

Figure S93: ^{1}H NMR spectrum of **33** in $CDCl_3$……………………………………………………….………S99
Figure S94: ^{13}C NMR spectrum of **33** in $CDCl_3$…………………………………………………...…….S100
Figure S95: High resolution mass spectrum of **33**……………………………………………...……S101
Figure S96: HPLC chromatogram of **33**……………………………………………………………..S102
Figure S97: ^{1}H NMR spectrum of **34** in $CDCl_3$…………………………………………………..……...S103
Figure S98: ^{13}C NMR spectrum of **34** in $CDCl_3$……………………………………………..…….…..S104
Figure S99: High resolution mass spectrum of **34**…………………………………………..………..S105
Figure S100: HPLC chromatogram of **34**……………………………………………………..………S106
Figure S101: ^{1}H NMR spectrum of **35** in $CDCl_3$……………………………………………….....……S107
Figure S102: ^{13}C NMR spectrum of **35** in $CDCl_3$……………………………………………………S108
Figure S103: High resolution mass spectrum of **35**…………………………………………………...S109
Figure S104: HPLC chromatogram of **35**……………………………………………………………S110
Figure S105: ^{1}H NMR spectrum of **5A** in $CDCl_3$……………………………………………………S111
Figure S106: ^{13}C NMR spectrum of **5A** in $CDCl_3$……………………………………………….……S112
Figure S107: High resolution mass spectrum of **5A**……………………………………..………...S113
Figure S108: HPLC chromatogram of **5A**……………………………………………...…………S114
Figure S109: ^{1}H NMR spectrum of **5B** in $CDCl_3$…………………………………………………...…S115
Figure S110: ^{13}C NMR spectrum of **5B** in $CDCl_3$………………………………………………...…S116
Figure S111: High resolution mass spectrum of **5B**……………………………………...…………S117
Figure S112: HPLC chromatogram of **5B**…………………………………………………..……S118
Figure S113: ^{1}H NMR spectrum of **41A** in $CDCl_3$………………………………………….…………S119
Figure S114: ^{13}C NMR spectrum of **41A** in $CDCl_3$……………………………………………………S120
Figure S115: High resolution mass spectrum of **41A**………………………………………………...S121
Figure S116: HPLC chromatogram of **41A**………………………………………………………….S122
Figure S117: ^{1}H NMR spectrum of **41B** in $CDCl_3$…………………………………………………..S123
Figure S118: ^{13}C NMR spectrum of **41B** in $CDCl_3$…………………………………………………….S124
Figure S119: High resolution mass spectrum of **41B**…………………………………………………S125
Figure S120: HPLC chromatogram of **41B**………………………………………………………….S126
Figure S121: ^{1}H NMR spectrum of **10A** in $CDCl_3$……………………………………………………….S127
Figure S122: ^{13}C NMR spectrum of **10A** in $CDCl_3$……………………………………………………S128
Figure S123: High resolution mass spectrum of **10A**…………………………………...……………S129
Figure S124: HPLC chromatogram of **10A**………………………………………………………….S130
Figure S125: ^{1}H NMR spectrum of **10B** in $CDCl_3$…………………………………………………..S131
Figure S126: ^{13}C NMR spectrum of **10B** in $CDCl_3$…………………………………………………….S132
Figure S127: High resolution mass spectrum of **10B**…………………………………………………S133
Figure S128: HPLC chromatogram of **10B**…………………………………………………………..S134

Figure S129: ^{1}H NMR spectrum of **11A** in $CDCl_3$……S135
Figure S130: ^{13}C NMR spectrum of **11A** in $CDCl_3$……S136
Figure S131: High resolution mass spectrum of **11A**……S137
Figure S132: HPLC chromatogram of **11A**……S138
Figure S133: ^{1}H NMR spectrum of **11B** in $CDCl_3$……S139
Figure S134: ^{13}C NMR spectrum of **11B** in $CDCl_3$……S140
Figure S135: High resolution mass spectrum of **11B**……S141
Figure S136: HPLC chromatogram of **11B**……S142
Figure S137: ^{1}H NMR spectrum of **6A** in $CDCl_3$……S143
Figure S138: ^{13}C NMR spectrum of **6A** in $CDCl_3$……S144
Figure S139: High resolution mass spectrum of **6A**……S145
Figure S140: HPLC chromatogram of **6A**……S146
Figure S141: ^{1}H NMR spectrum of **6B** in $CDCl_3$……S147
Figure S142: ^{13}C NMR spectrum of **6B** in $CDCl_3$……S148
Figure S143: High resolution mass spectrum of **6B**……S149
Figure S144: HPLC chromatogram of **6B**……S150
Figure S145: ^{1}H NMR spectrum of **7A** in $CDCl_3$……S151
Figure S146: ^{13}C NMR spectrum of **7A** in $CDCl_3$……S152
Figure S147: High resolution mass spectrum of **7A**……S153
Figure S148: HPLC chromatogram of **7A**……S154
Figure S149: ^{1}H NMR spectrum of **7B** in $CDCl_3$……S155
Figure S150: ^{13}C NMR spectrum of **7B** in $CDCl_3$……S156
Figure S151: High resolution mass spectrum of **7B**……S157
Figure S152: HPLC chromatogram of **7B**……S158
Figure S153: ^{1}H NMR spectrum of **8A** in $CDCl_3$……S159
Figure S154: ^{13}C NMR spectrum of **8A** in $CDCl_3$……S160
Figure S155: High resolution mass spectrum of **8A**……S161
Figure S156: HPLC chromatogram of **8A**……S162
Figure S157: ^{1}H NMR spectrum of **8B** in $CDCl_3$……S163
Figure S158: ^{13}C NMR spectrum of **8B** in $CDCl_3$……S164
Figure S159: High resolution mass spectrum of **8B**……S165
Figure S160: HPLC chromatogram of **8B**……S166
Figure S161: ^{1}H NMR spectrum of **9A** in $CDCl_3$……S167
Figure S162: ^{13}C NMR spectrum of **9A** in $CDCl_3$……S168
Figure S163: High resolution mass spectrum of **9A**……S169
Figure S164: HPLC chromatogram of **9A**……S170

Figure S165: ^{1}H NMR spectrum of **9B** in $CDCl_3$……………………………………………………………………S171
Figure S166: ^{13}C NMR spectrum of **9B** in $CDCl_3$……………………………………………………………………S172
Figure S167: High resolution mass spectrum of **9B**…………………………………………………………………...S173
Figure S168: HPLC chromatogram of **9B**……………………………………………………………………………S174
Figure S169: COSY spectrum of **5** in $CDCl_3$…………………………………………………………………………S175
Figure S170: HMQC spectrum of **5** in $CDCl_3$………………………………………………………………………..S176
Figure S171: HMBC spectrum of **5** in $CDCl_3$………………………………………………………………………..S177

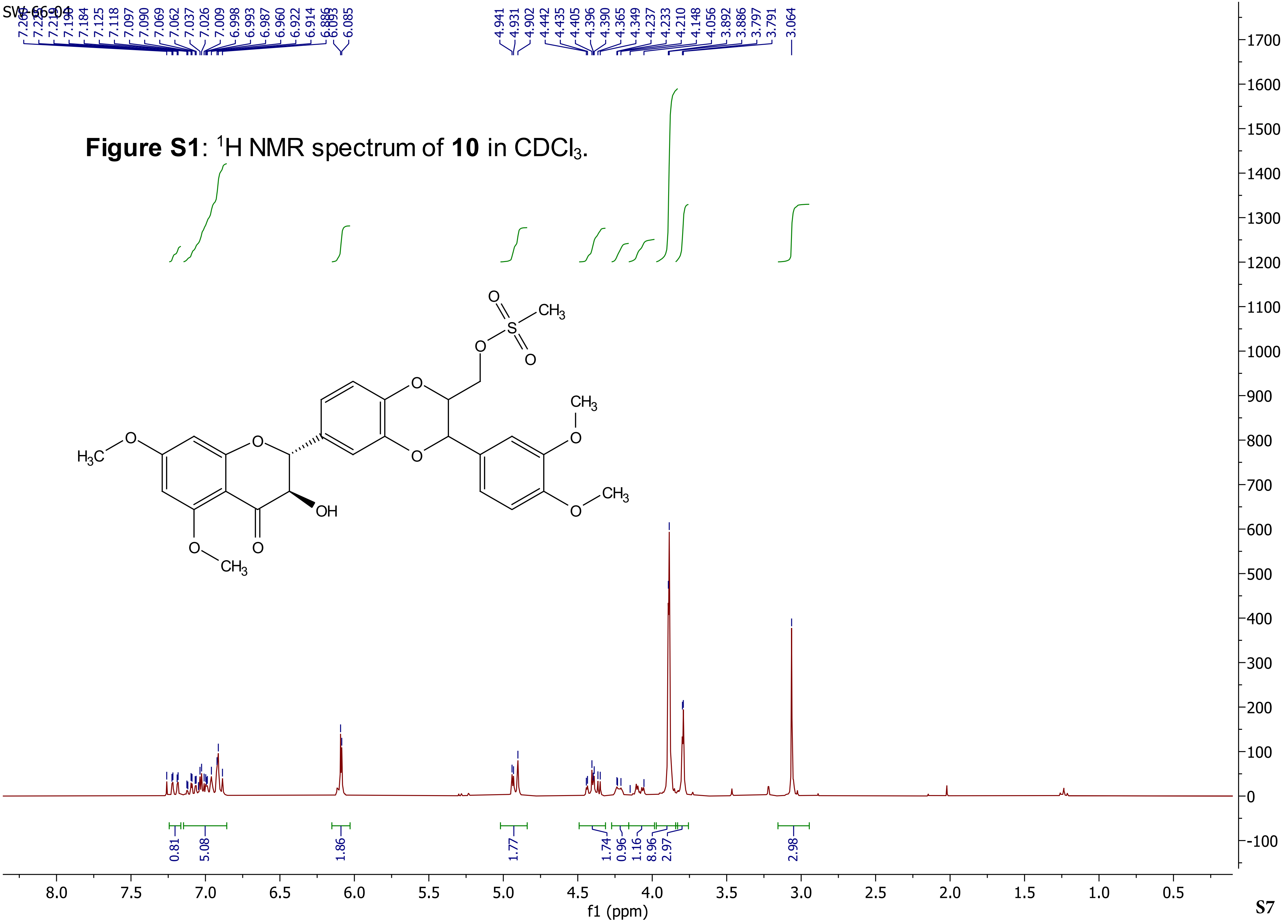

Figure S1: ^{1}H NMR spectrum of **10** in $CDCl_3$.

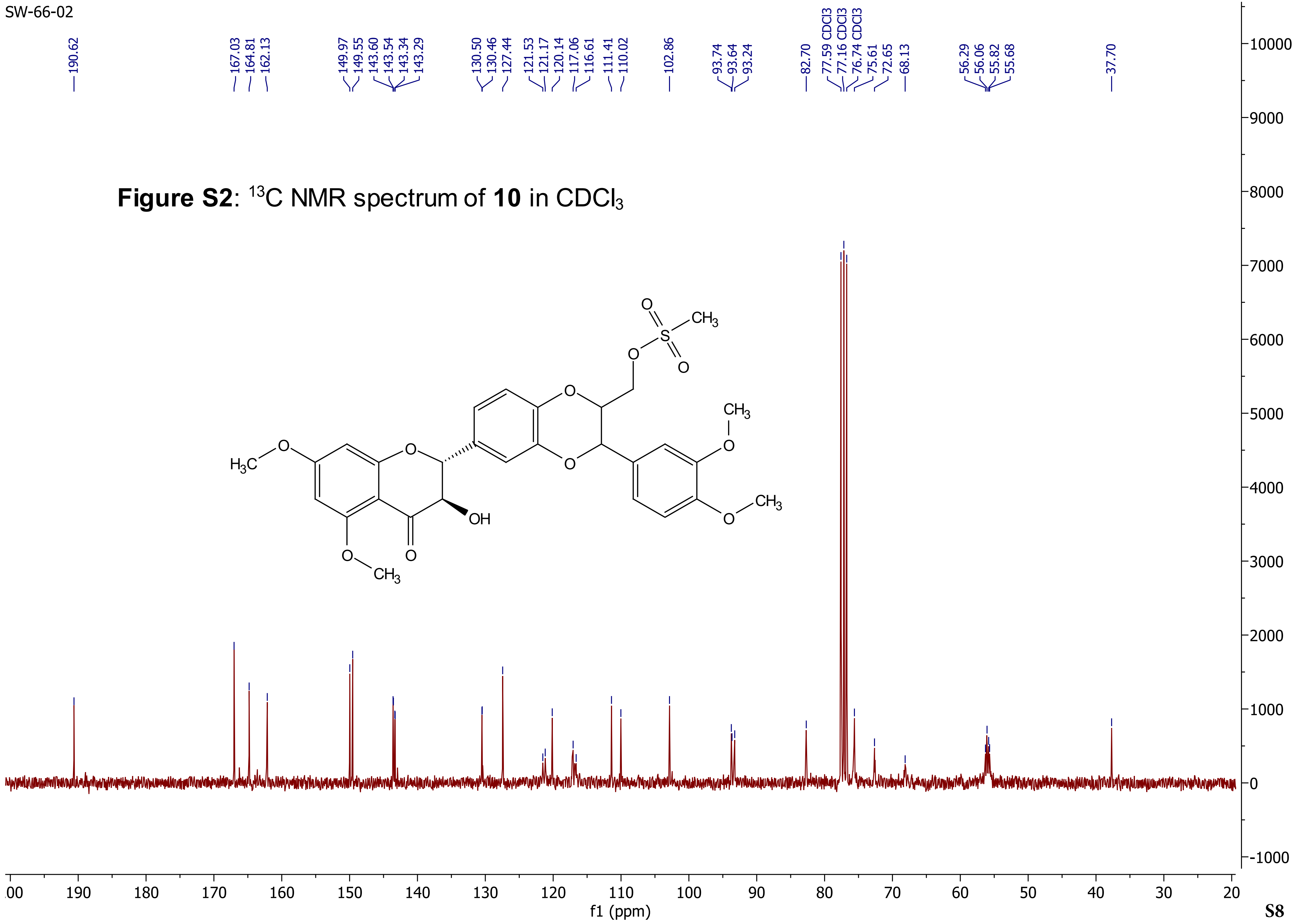

Figure S2: ^{13}C NMR spectrum of **10** in $CDCl_3$

SW-66-04	C29H30O12S	602.1459	603.1537	**603.1536**	**-0.0001**	**-0.10**

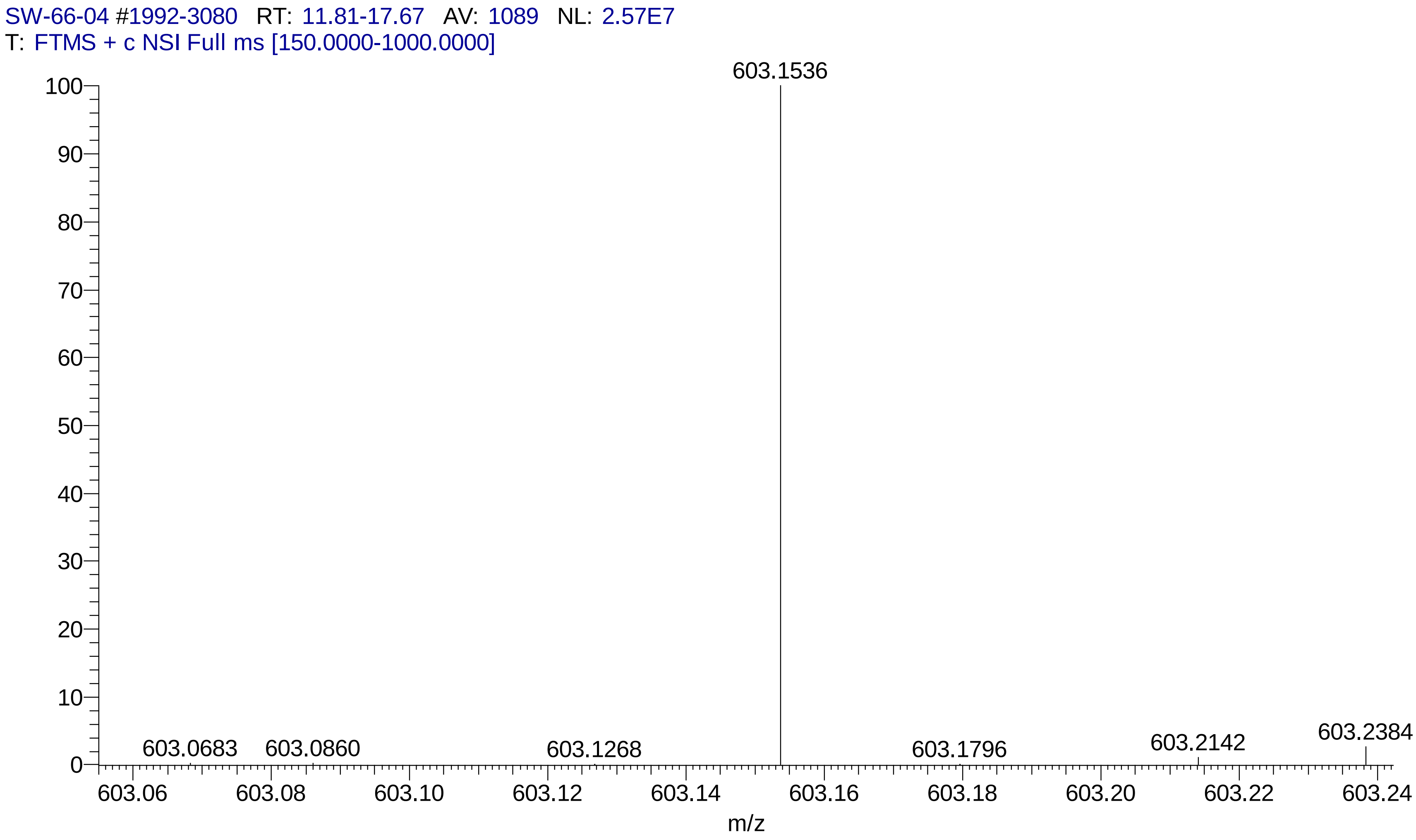

Figure S3: High resolution mass spectrum of **10**

===

Injection Date : 4/26/2022 3:56:09 PM
Sample Name : SW-66-04 Location : Vial 1
Acq. Operator :
Method : C:\HPCHEM\1\METHODS\JNP2015.M
Last changed : 4/24/2022 3:10:54 PM
(modified after loading)

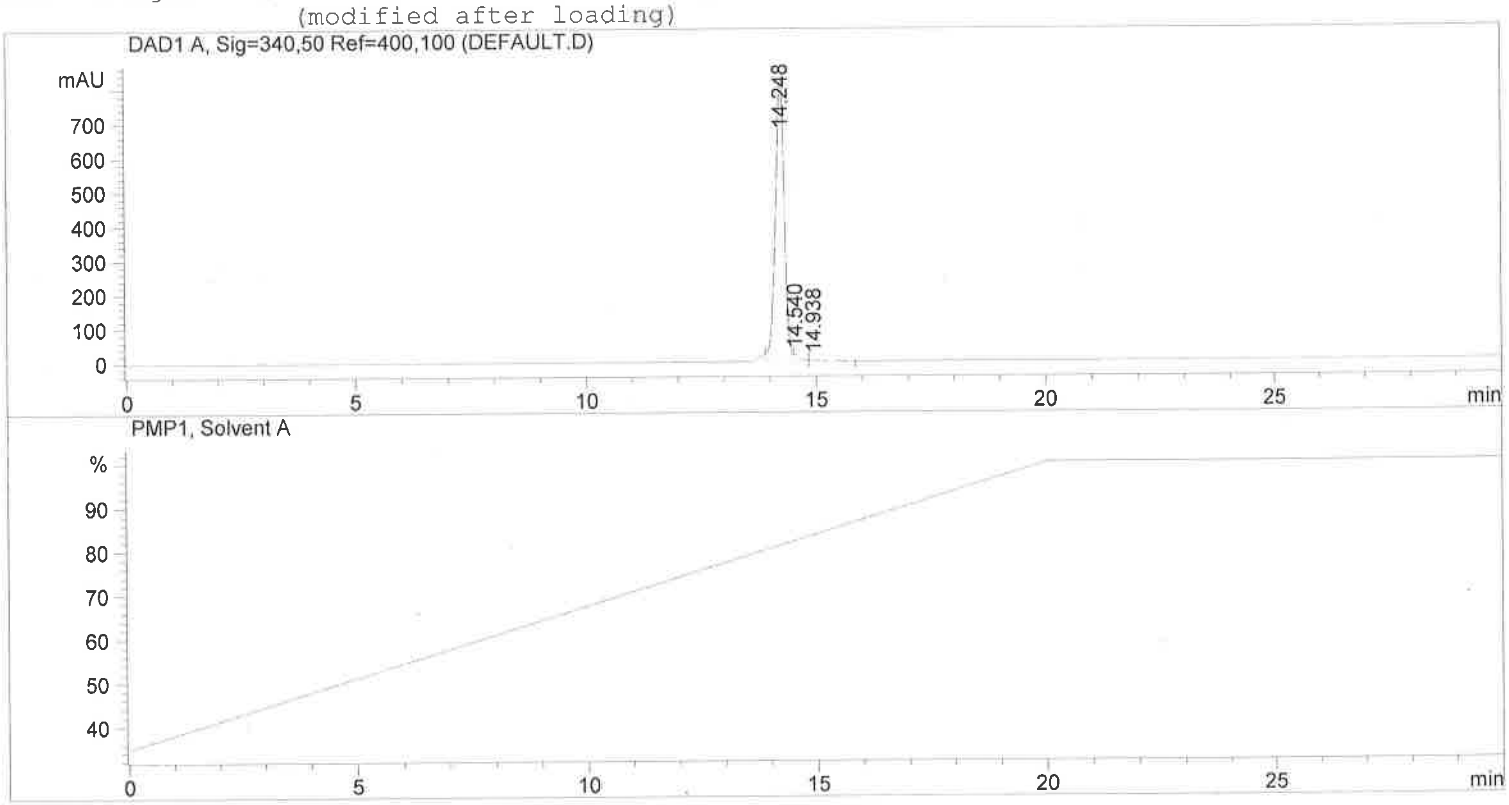

===
Area Percent Report
===

Sorted By : Signal
Multiplier : 1.0000
Dilution : 1.0000

Signal 1: DAD1 A, Sig=340,50 Ref=400,100

Peak #	RetTime [min]	Type	Width [min]	Area [mAU*s]	Height [mAU]	Area %
1	14.248	BV	0.1998	1.09255e4	828.84259	97.5757
2	14.540	VV	0.1418	198.29533	20.33183	1.7710
3	14.938	VB	0.1843	73.15618	5.75418	0.6534

Totals : 1.11970e4 854.92860

Results obtained with enhanced integrator!
===
*** End of Report ***

Figure S4: HPLC chromatogram of **10**

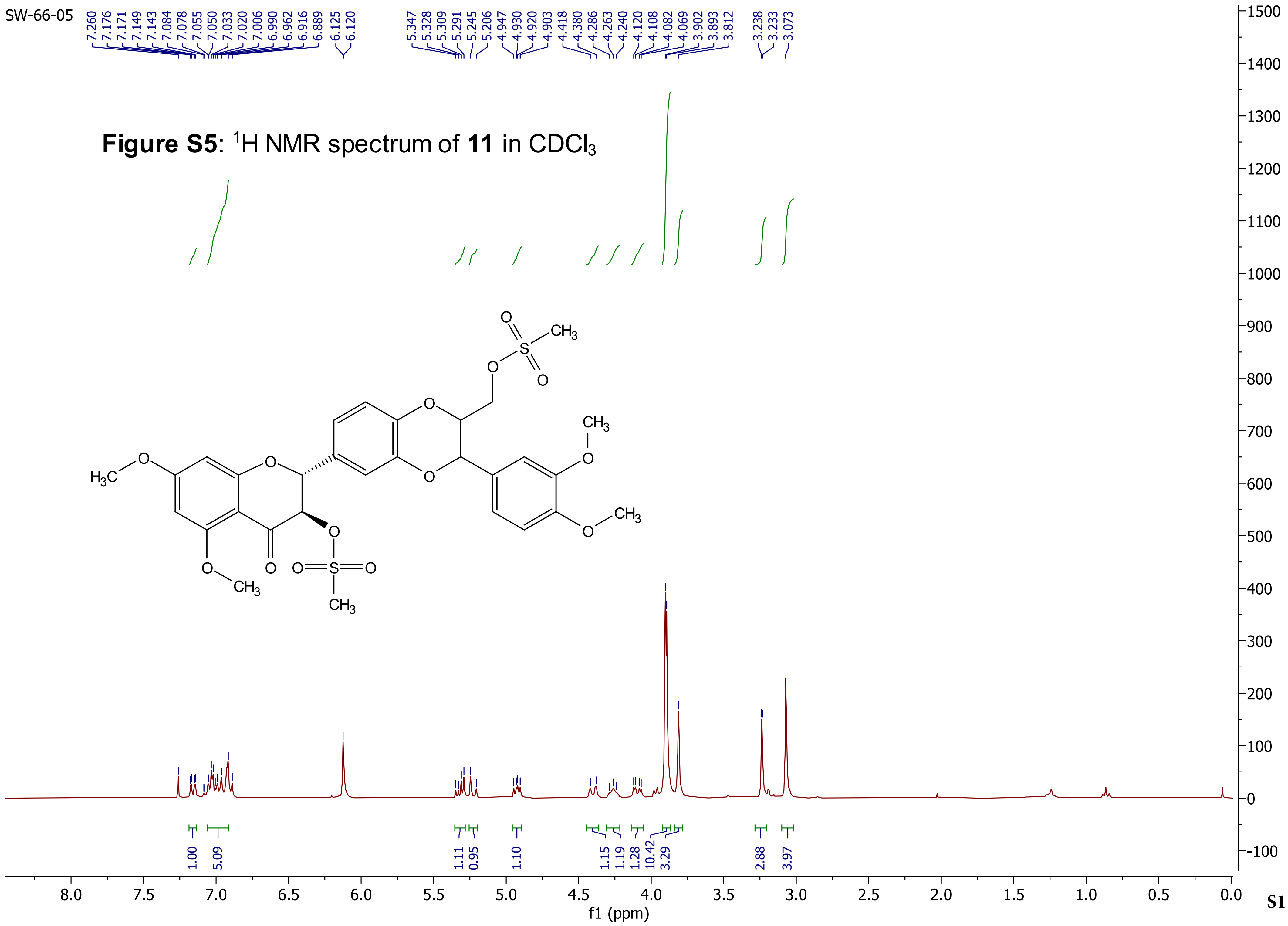

Figure S5: ^{1}H NMR spectrum of **11** in $CDCl_3$

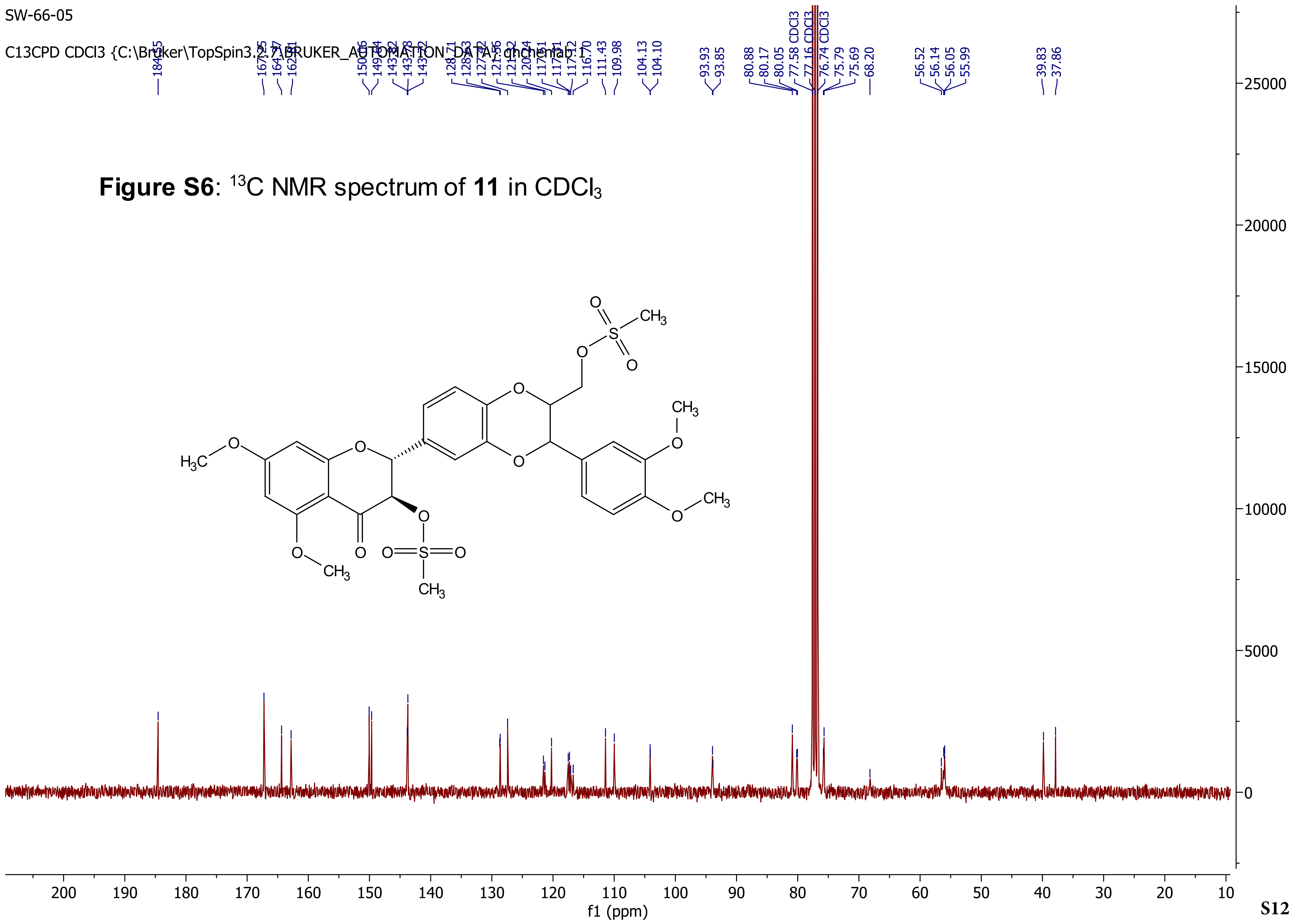

Figure S6: ^{13}C NMR spectrum of **11** in $CDCl_3$

SW-66-05	C30H32O14S2	680.1234	681.1312	**681.1335**	0.0023	3.35

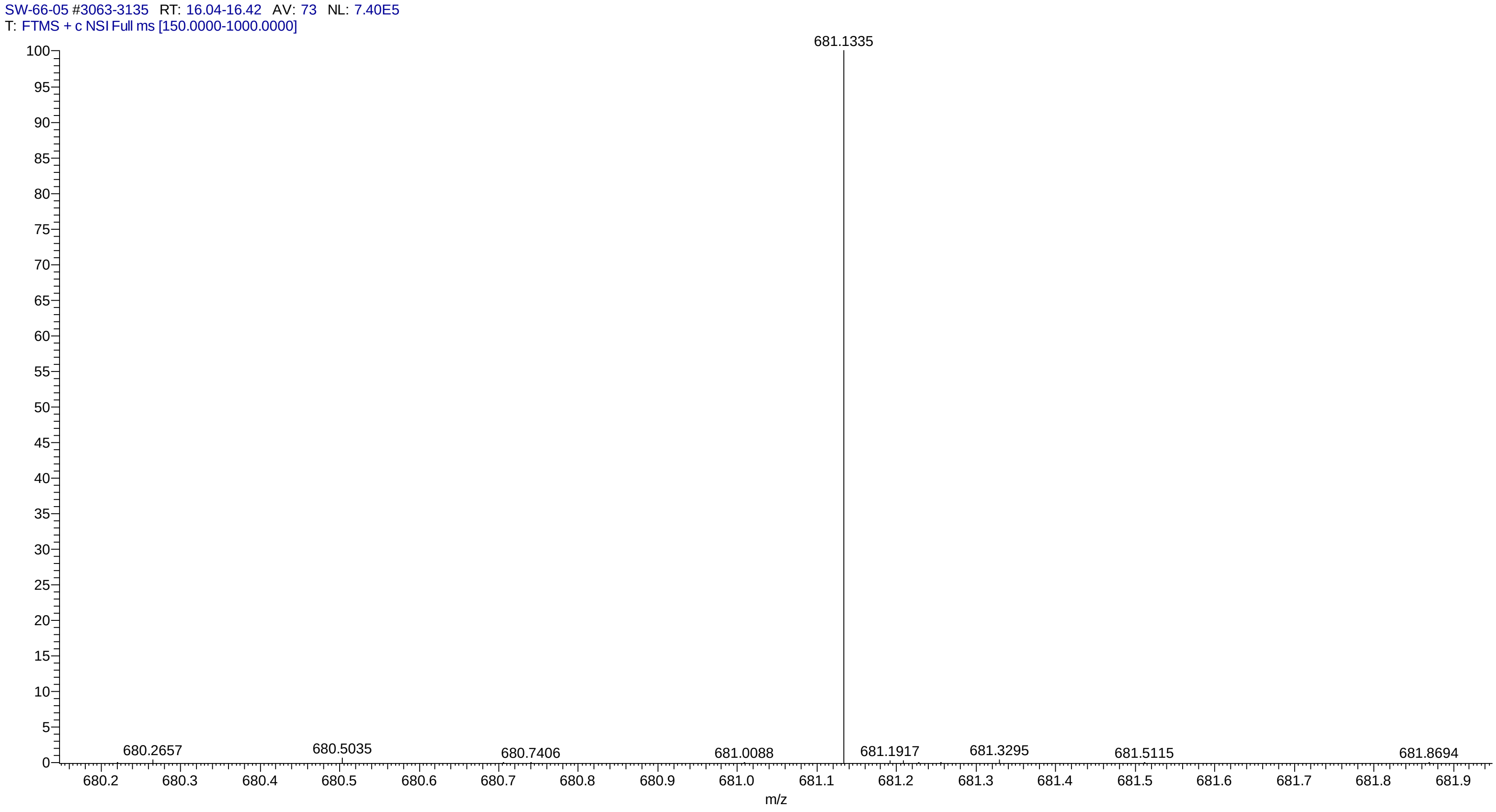

Figure S7: High resolution mass spectrum of **11**

===

Injection Date : 4/26/2022 12:51:40 PM
Sample Name : SW-66-05 Location : Vial 1
Acq. Operator :
Method : C:\HPCHEM\1\METHODS\JNP2015.M
Last changed : 4/24/2022 3:10:54 PM
(modified after loading)

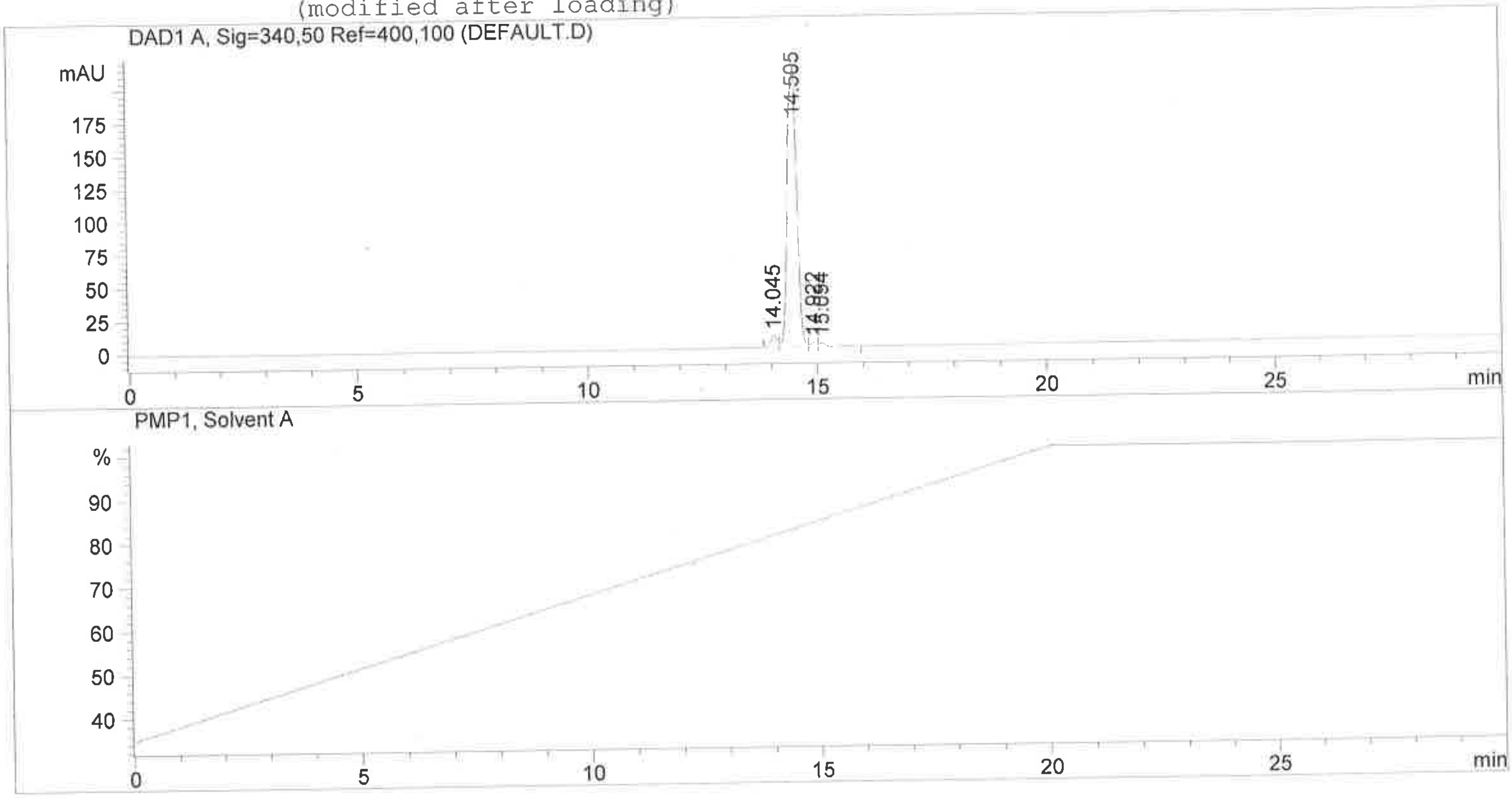

===

Area Percent Report

===

Sorted By : Signal
Multiplier : 1.0000
Dilution : 1.0000

Signal 1: DAD1 A, Sig=340,50 Ref=400,100

Peak #	RetTime [min]	Type	Width [min]	Area [mAU*s]	Height [mAU]	Area %
1	14.045	BV	0.1401	84.44093	9.11330	2.6230
2	14.505	VV	0.2311	3058.64844	213.51776	95.0097
3	14.922	VV	0.1380	34.50250	3.65813	1.0717
4	15.094	VP	0.1898	41.70987	3.08400	1.2956

Totals : 3219.30173 229.37320

Results obtained with enhanced integrator!

===

*** End of Report ***

Figure S8: HPLC chromatogram of **11**

SW-66-07

Figure S9: ^1H NMR spectrum of **12** in $CDCl_3$

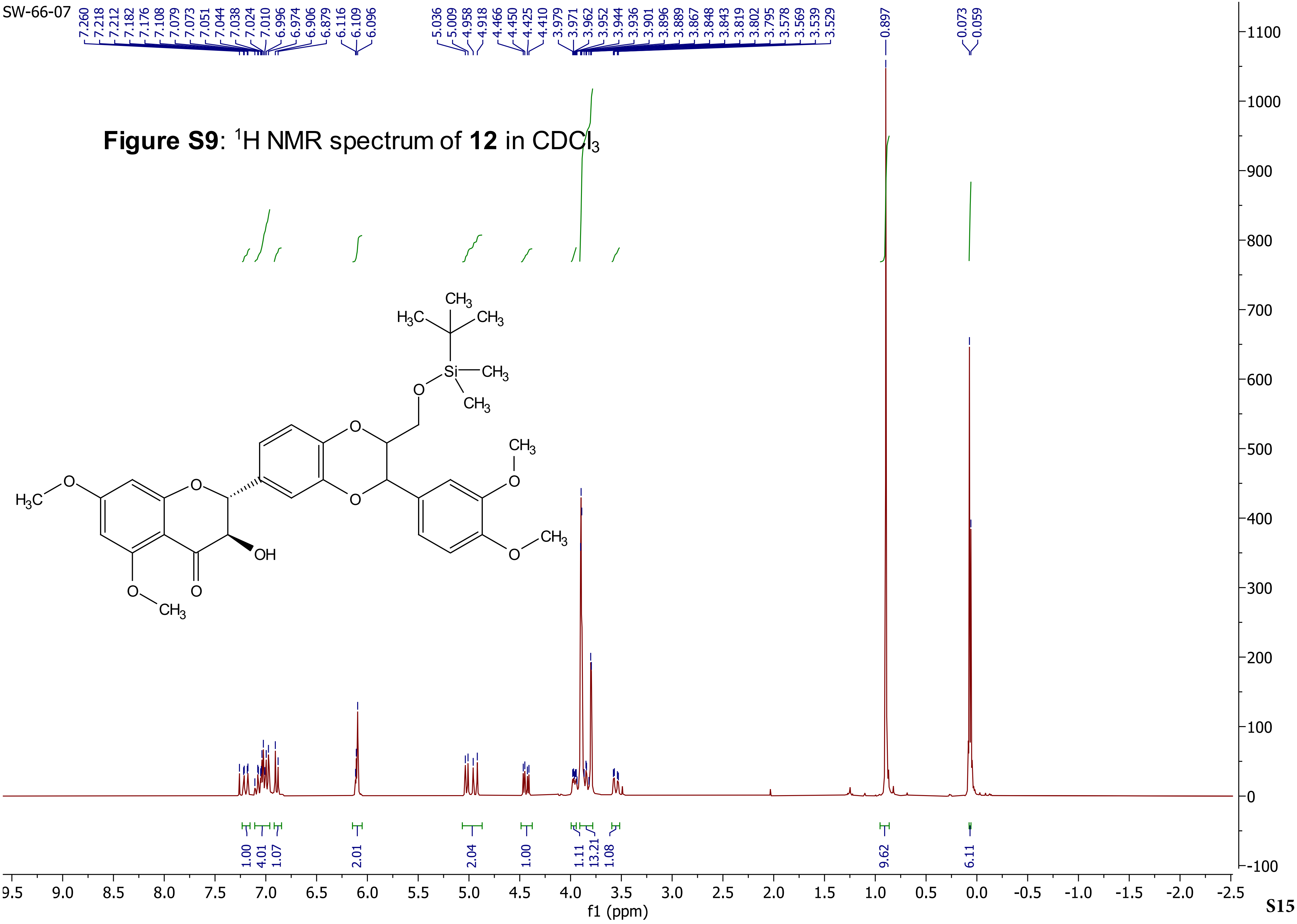

SW-66-07-01

Figure S10: ^{13}C NMR spectrum of **12** in $CDCl_3$

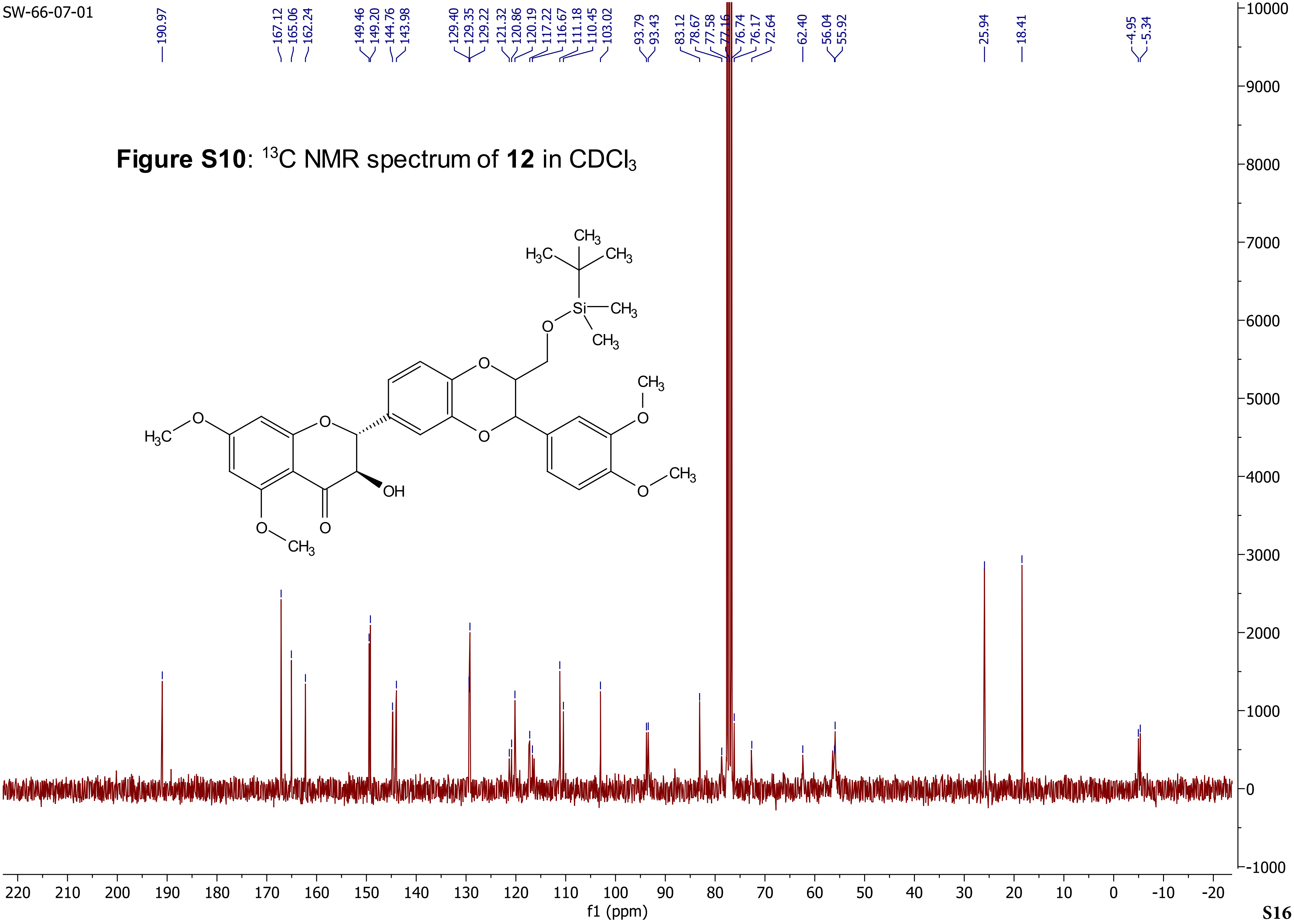

SW-66-07	C24H42O10Si	638.2548	639.2626	**639.2623**	**-0.0003**	**-0.44**

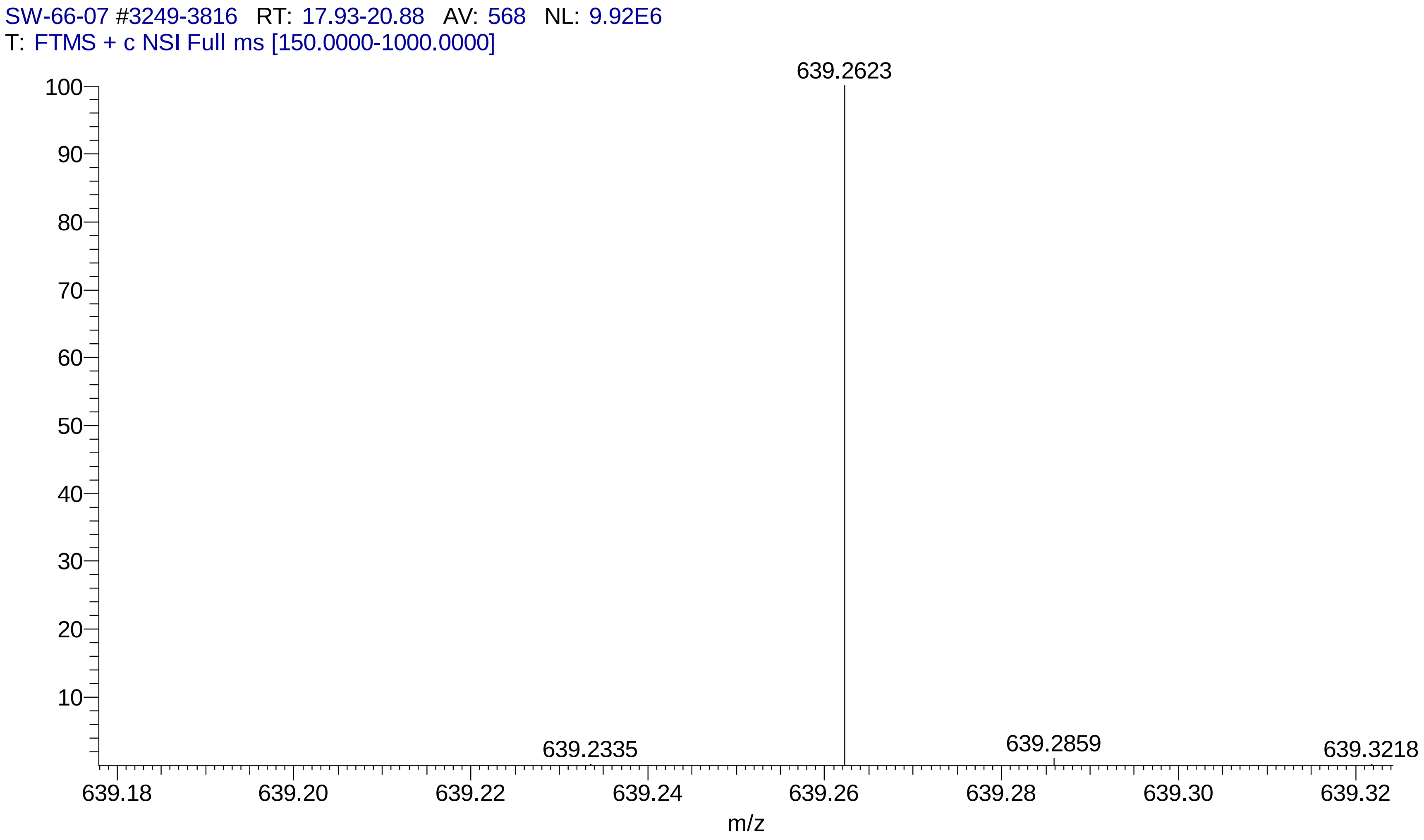

Figure S11: High resolution mass spectrum of **12**

===

Injection Date : 5/3/2022 10:17:13 AM
Sample Name : SW-66-07 Location : Vial 1
Acq. Operator :
Method : C:\HPCHEM\1\METHODS\JNP2015.M
Last changed : 4/30/2022 3:37:52 PM
(modified after loading)

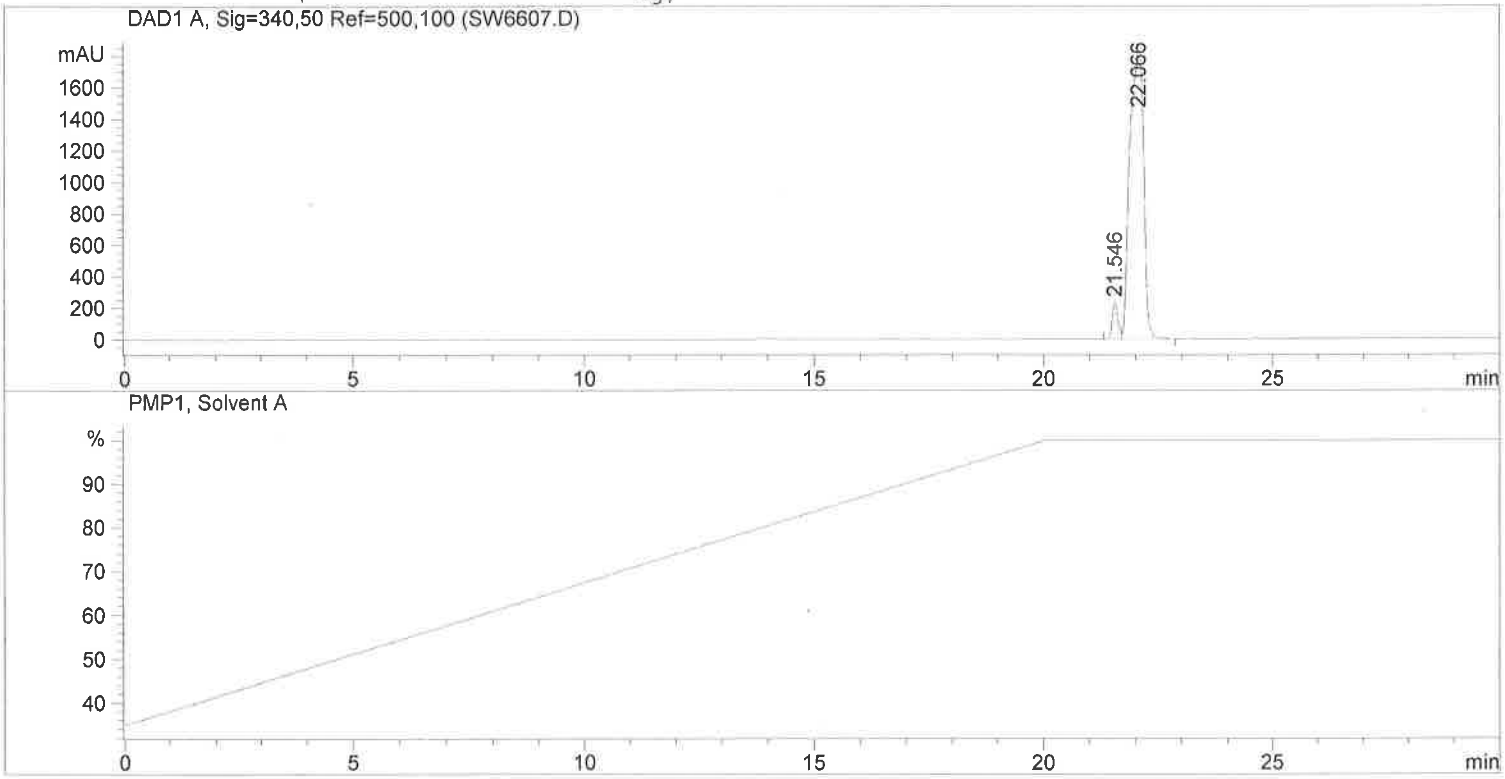

===

Area Percent Report

===

Sorted By : Signal
Multiplier : 1.0000
Dilution : 1.0000

Signal 1: DAD1 A, Sig=340,50 Ref=500,100

Peak #	RetTime [min]	Type	Width [min]	Area [mAU*s]	Height [mAU]	Area %
1	21.546	VV	0.1309	1945.58521	229.51608	4.8534
2	22.066	VV	0.3689	3.81414e4	1787.52332	95.1466

Totals : 4.00869e4 2017.03940

Results obtained with enhanced integrator!

===

*** End of Report ***

Figure S12: HPLC chrommatogram of **12**

SW-66-114

Figure S13: ^{1}H NMR spectrum of **13** in $CDCl_3$

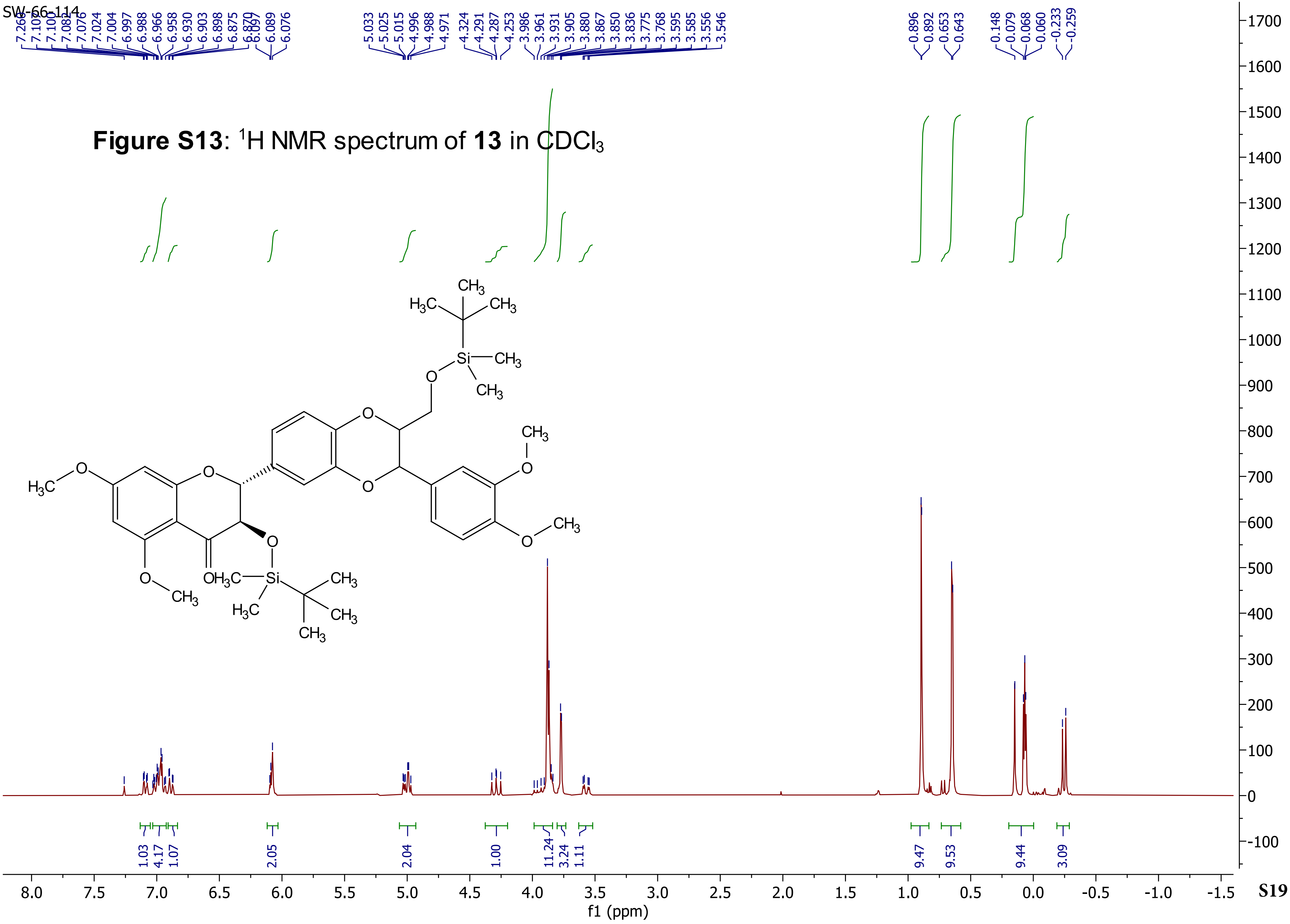

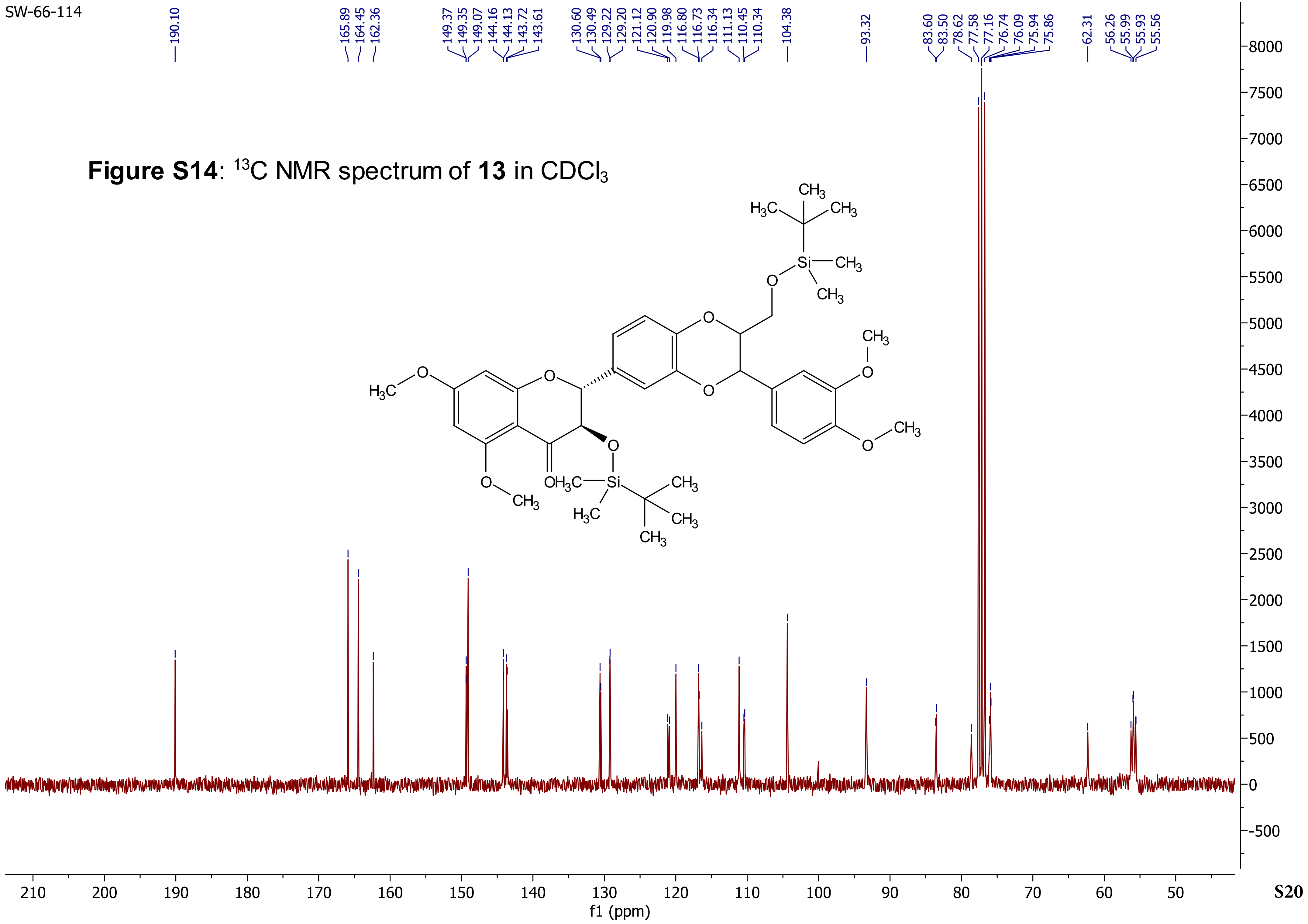

Figure S14: ^{13}C NMR spectrum of **13** in $CDCl_3$

Sample Name	Mol Fomula	MW	M+H	observed	delta	ppm
SW-66-08/114	C40H56O10Si2	752.3413	753.3491	753.3489	-0.0002	-0.21

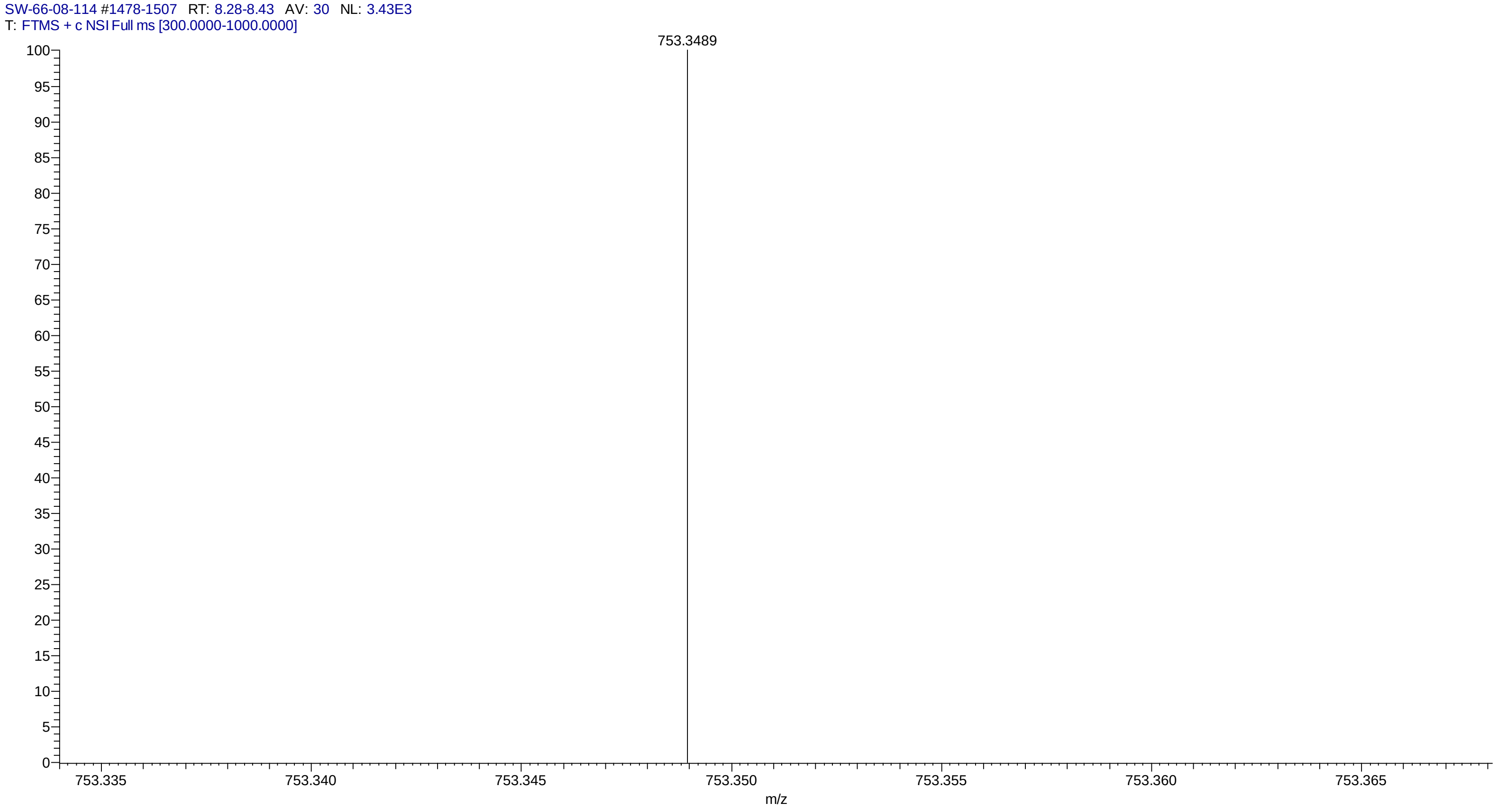

Figure S15: High resolution mass spectrum of **13**

Injection Date : 4/28/2022 1:30:24 PM
Sample Name : SW-66-08 Location : Vial 1
Acq. Operator :
Method : C:\HPCHEM\1\METHODS\JNP2015.M
Last changed : 4/28/2022 1:25:01 PM
(modified after loading)

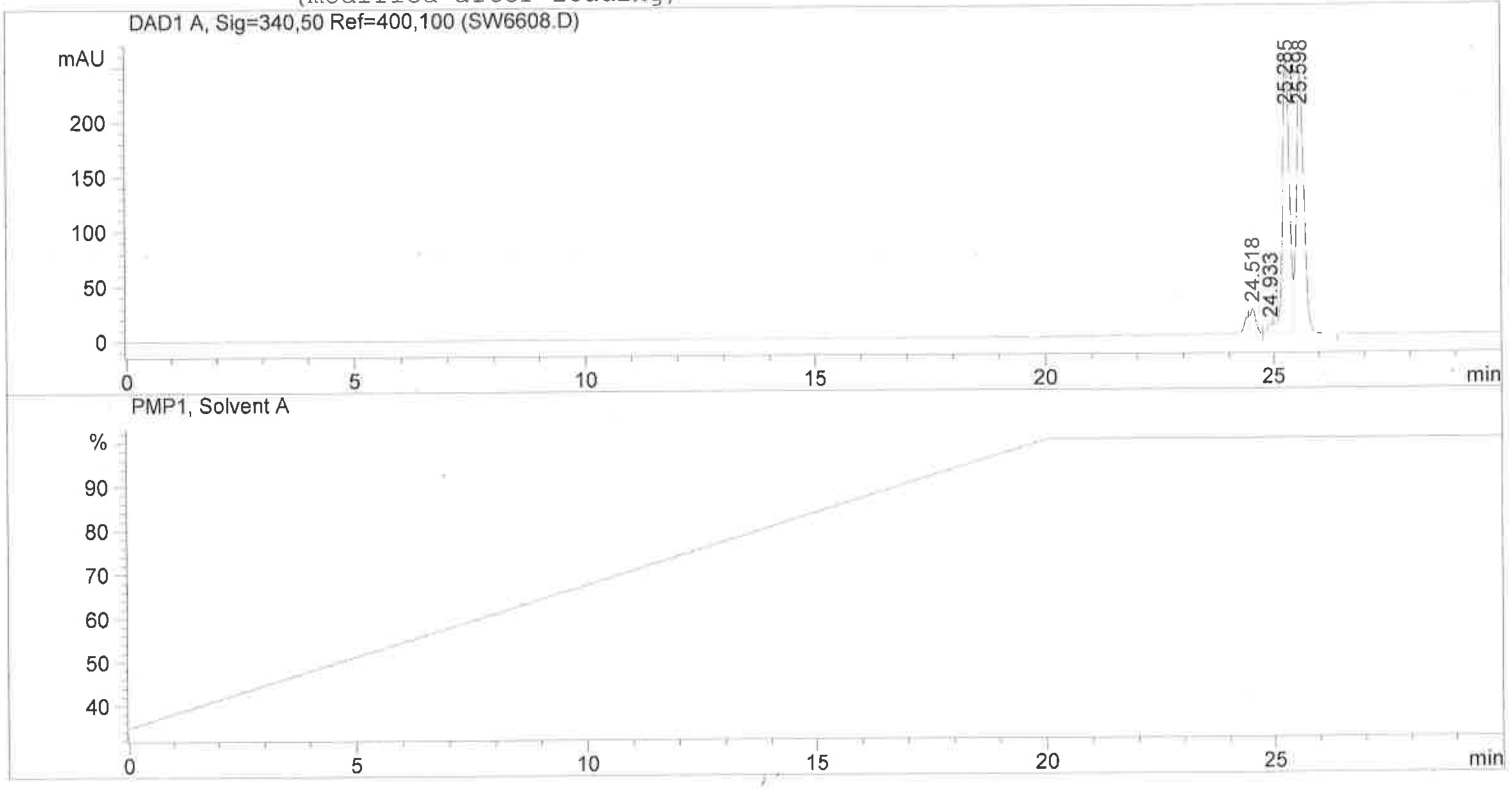

Area Percent Report

Sorted By : Signal
Multiplier : 1.0000
Dilution : 1.0000

Signal 1: DAD1 A, Sig=340,50 Ref=400,100

Peak #	RetTime [min]	Type	Width [min]	Area [mAU*s]	Height [mAU]	Area %
1	24.518	VP	0.1371	211.94373	23.08027	3.7549
2	24.933	VV	0.1226	73.86685	9.30888	1.3086
3	25.285	VV	0.1607	2685.11987	255.29753	47.5704
4	25.598	VB	0.1704	2673.58252	239.13531	47.3660

Totals : 5644.51297 526.82199

Results obtained with enhanced integrator!

*** End of Report ***

Figure S16: HPLC chromatogram of **13**

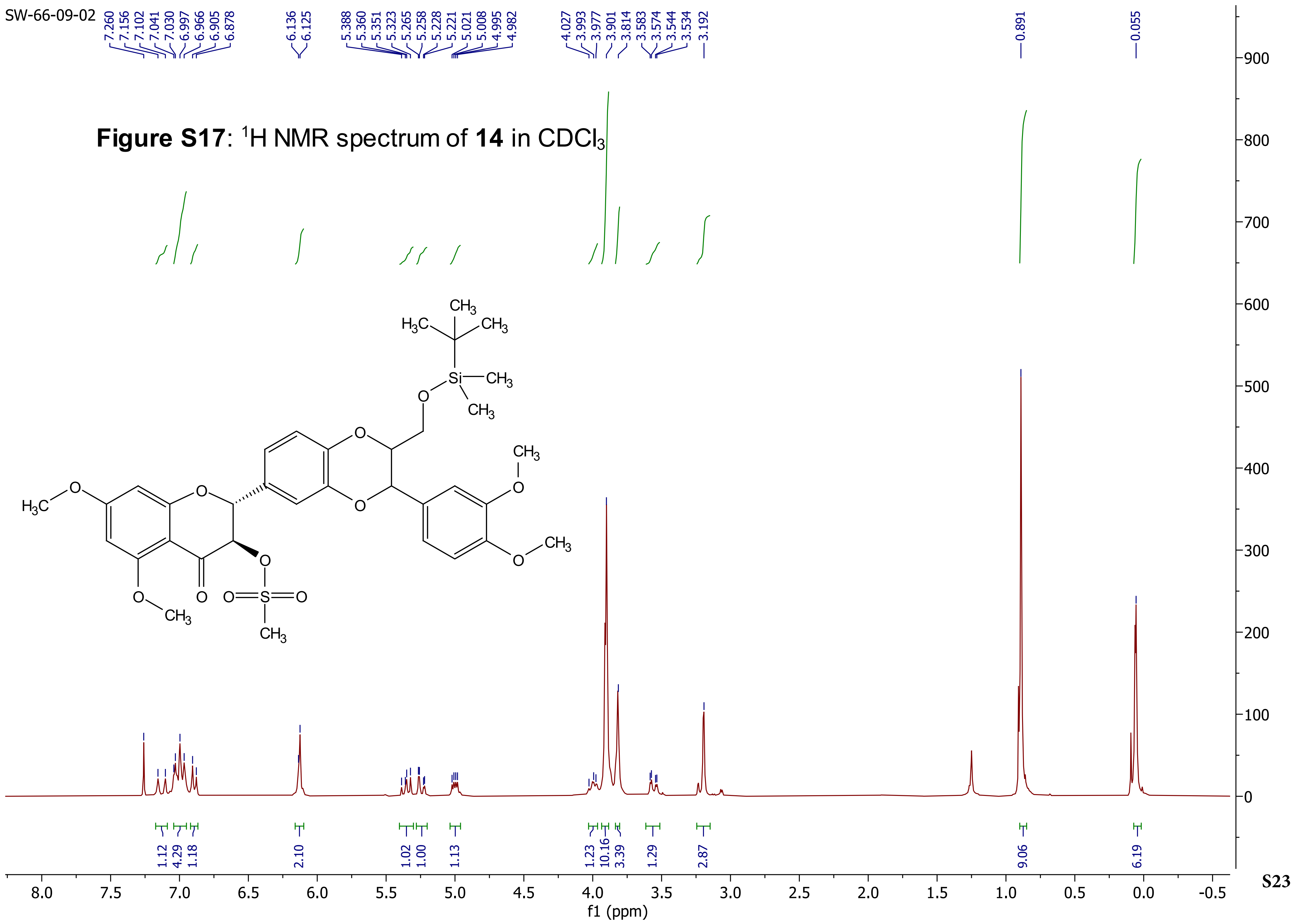

Figure S17: ^1H NMR spectrum of **14** in $CDCl_3$

SW-66-09-02

Figure S18: ^{13}C NMR spectrum of **14** in $CDCl_3$

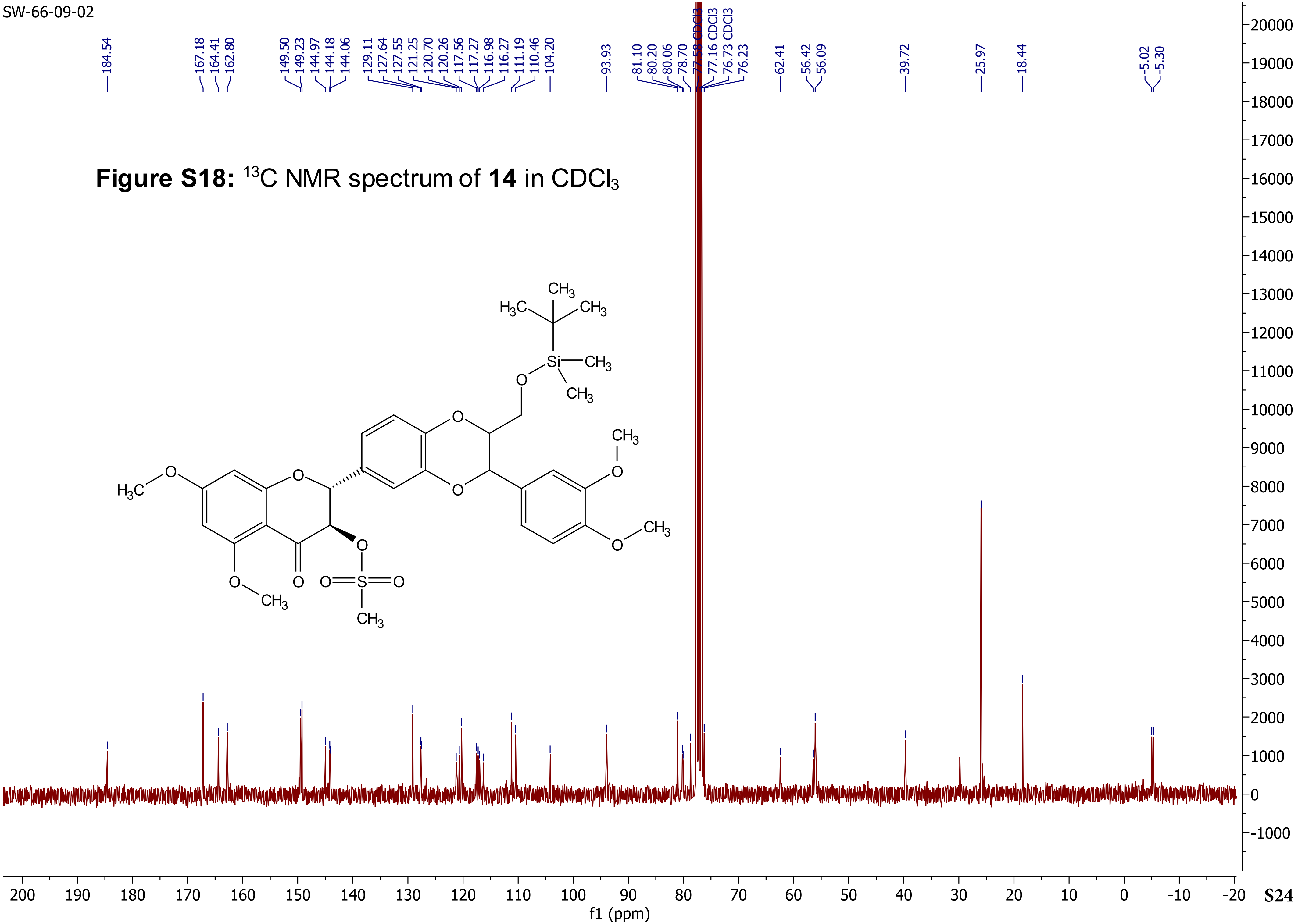

compds	Mol. Formula	Exact Mass	M+H	Observed	delta	ppm
SW-66-09	C35H44O12SSi	716.2323	717.2401	**717.2398**	-0.0003	-0.47

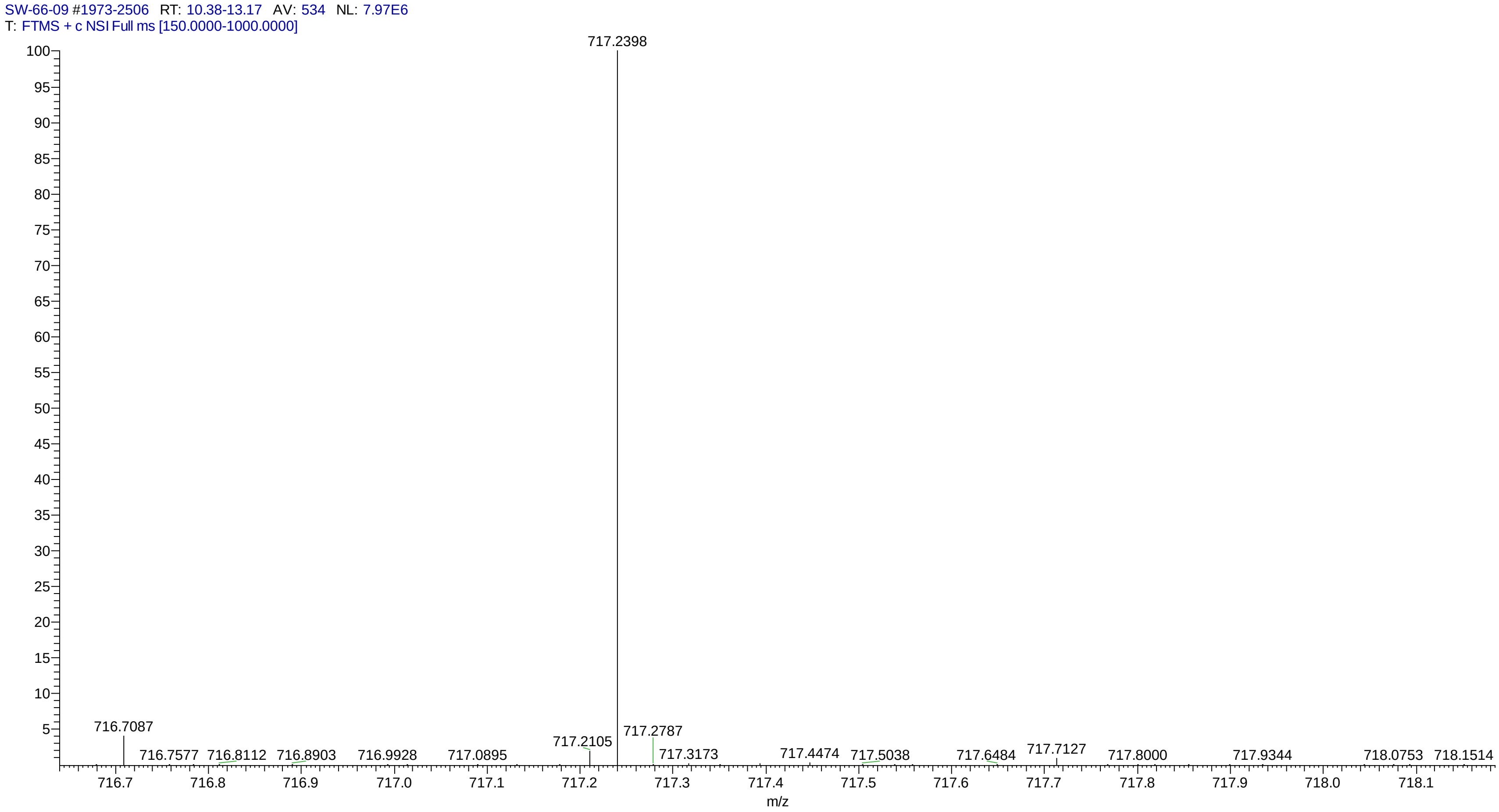

Figure S19: High resolution mass spectrum of **14**

===

Injection Date : 4/27/2022 12:22:44 PM
Sample Name : SW-66-09 Location : Vial 1
Acq. Operator :
Method : C:\HPCHEM\1\METHODS\JNP2015.M
Last changed : 4/24/2022 3:10:54 PM
(modified after loading)

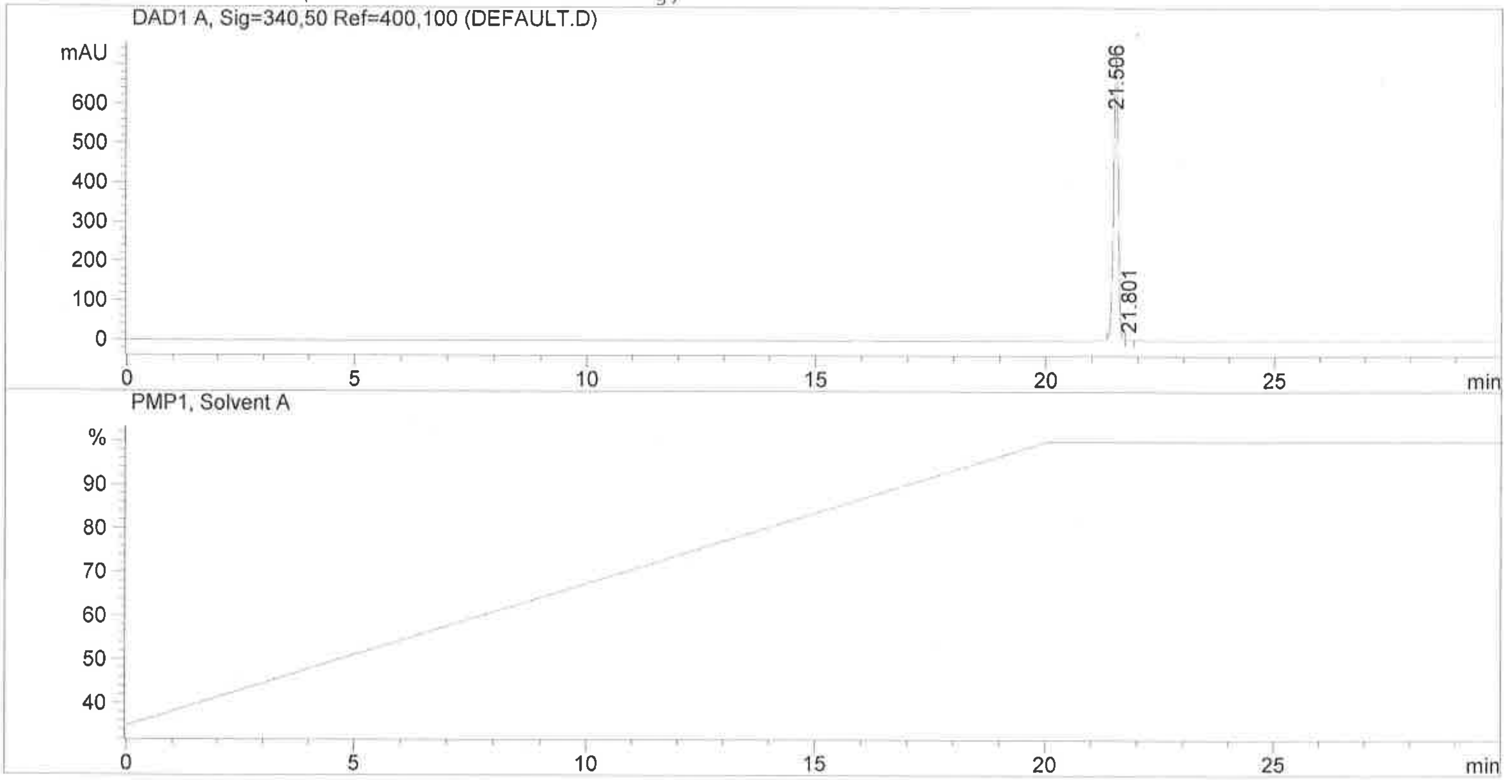

===

Area Percent Report

===

Sorted By : Signal
Multiplier : 1.0000
Dilution : 1.0000

Signal 1: DAD1 A, Sig=340,50 Ref=400,100

Peak #	RetTime [min]	Type	Width [min]	Area [mAU*s]	Height [mAU]	Area %
1	21.506	BV	0.1090	5121.35742	719.78973	98.9668
2	21.801	VV	0.1176	53.46510	6.81031	1.0332

Totals : 5174.82252 726.60004

Results obtained with enhanced integrator!

===

*** End of Report ***

Figure S20: HPLC chromatogram of **14**

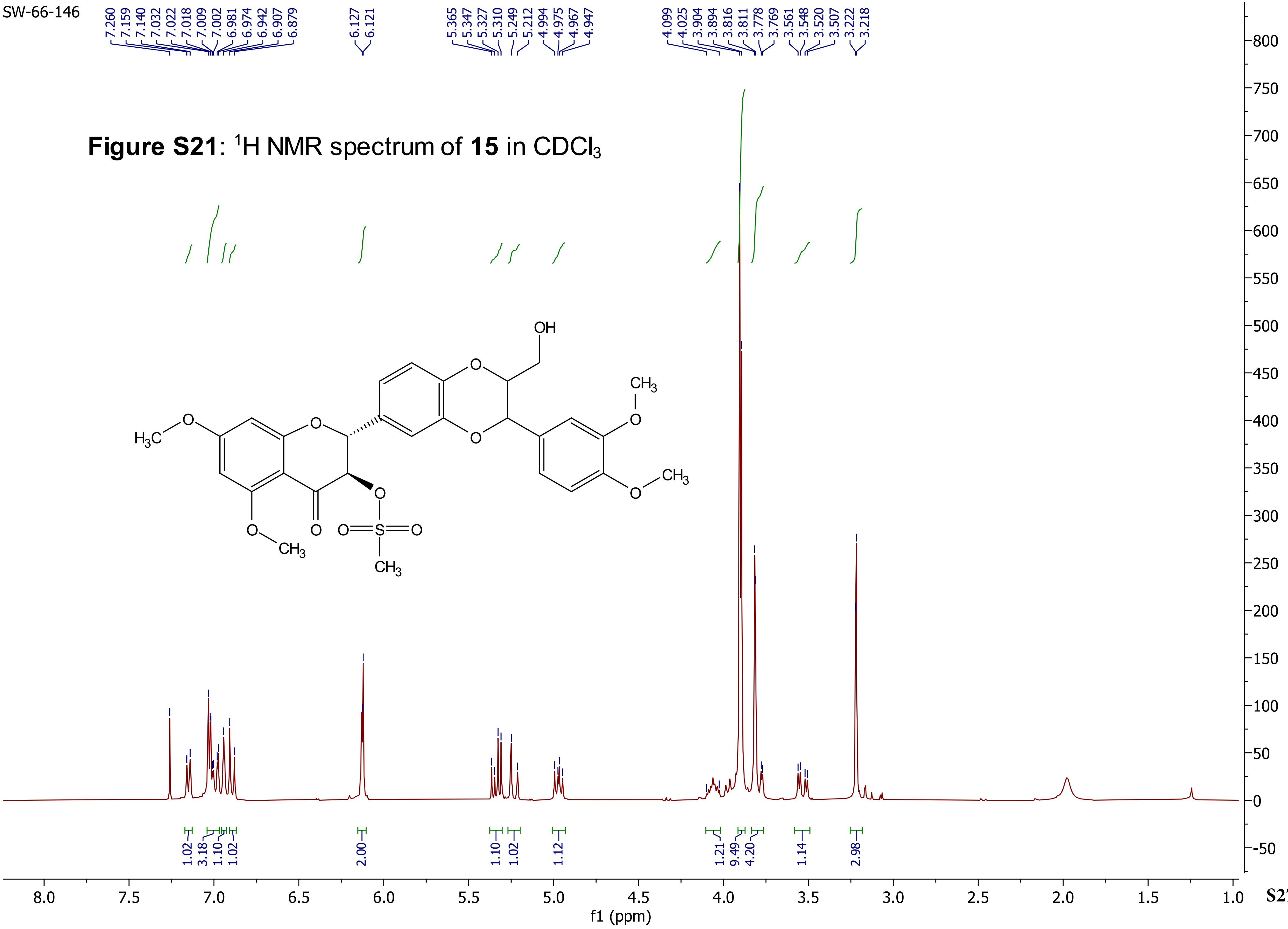

Figure S21: ^{1}H NMR spectrum of **15** in $CDCl_3$

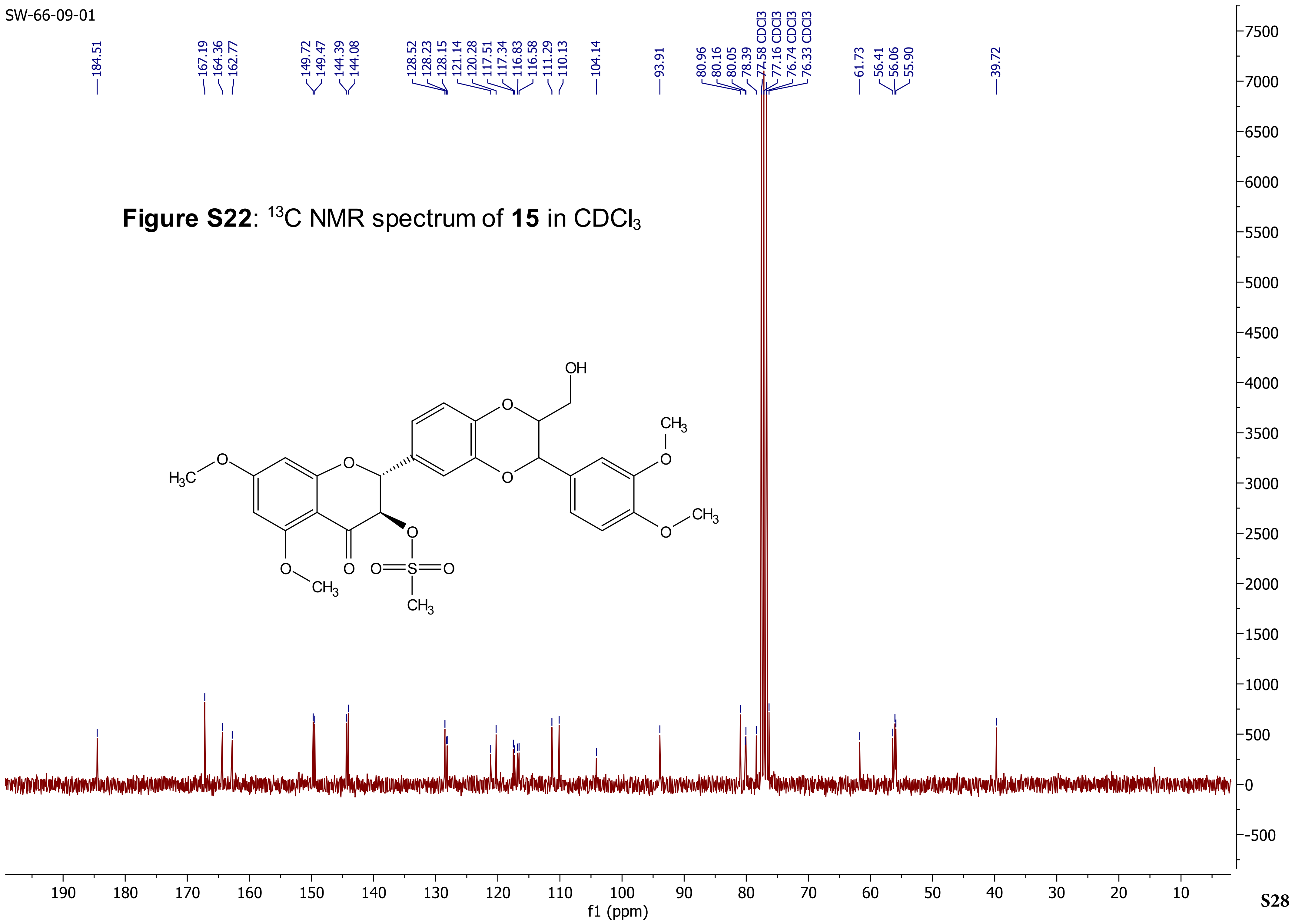

Figure S22: ^{13}C NMR spectrum of **15** in $CDCl_3$

SW-66-09-01	C29H30O12S	602.1459	603.1537	**603.1536**	-0.0001	-0.10

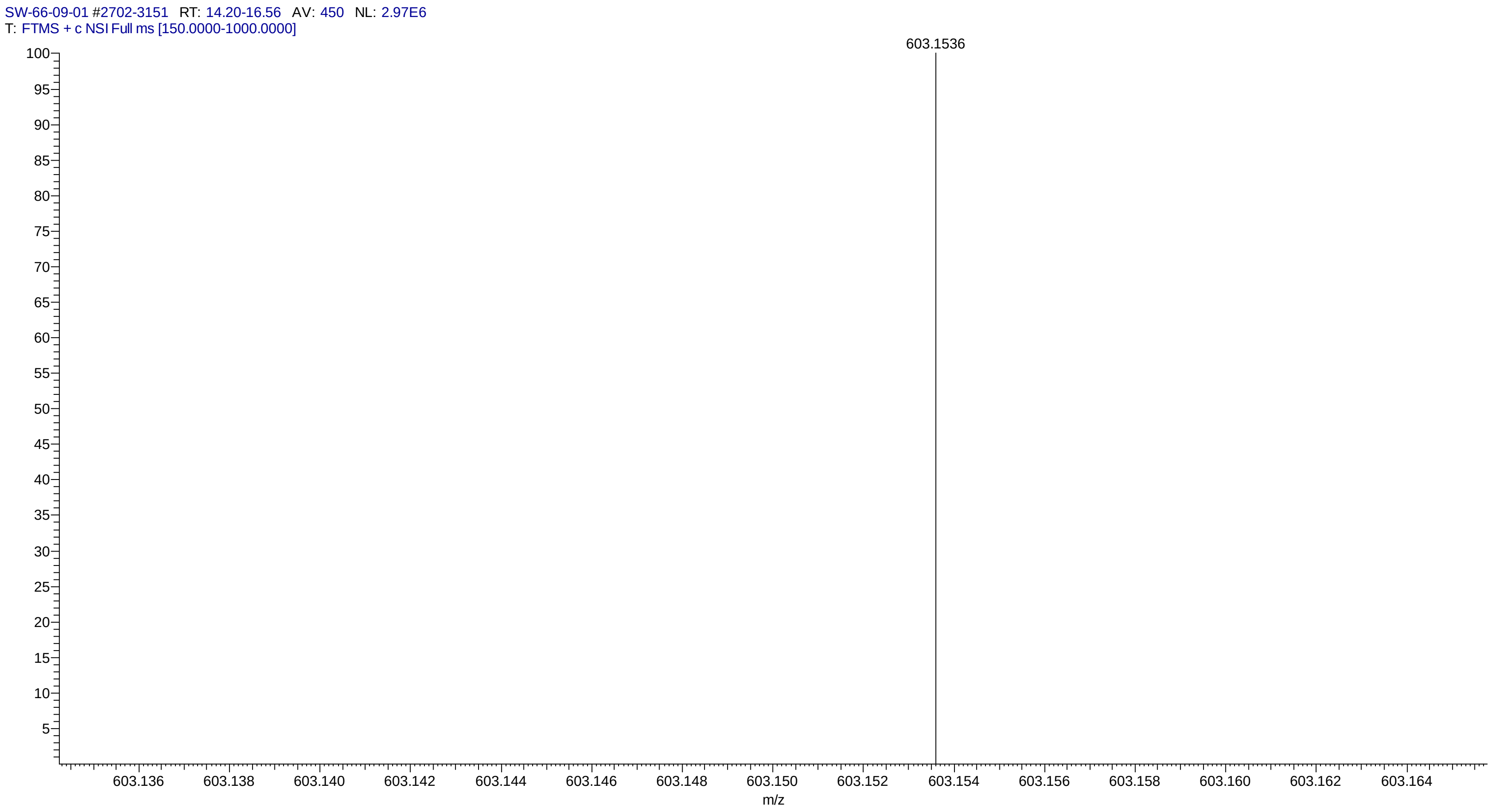

Figure S23: High resolution mass spectrum of **15**

===

Injection Date : 4/25/2022 5:17:26 PM
Sample Name : SW-66-09-01   Location : Vial 1
Acq. Operator :
Method : C:\HPCHEM\1\METHODS\JNP2015.M
Last changed : 4/24/2022 3:10:54 PM
(modified after loading)

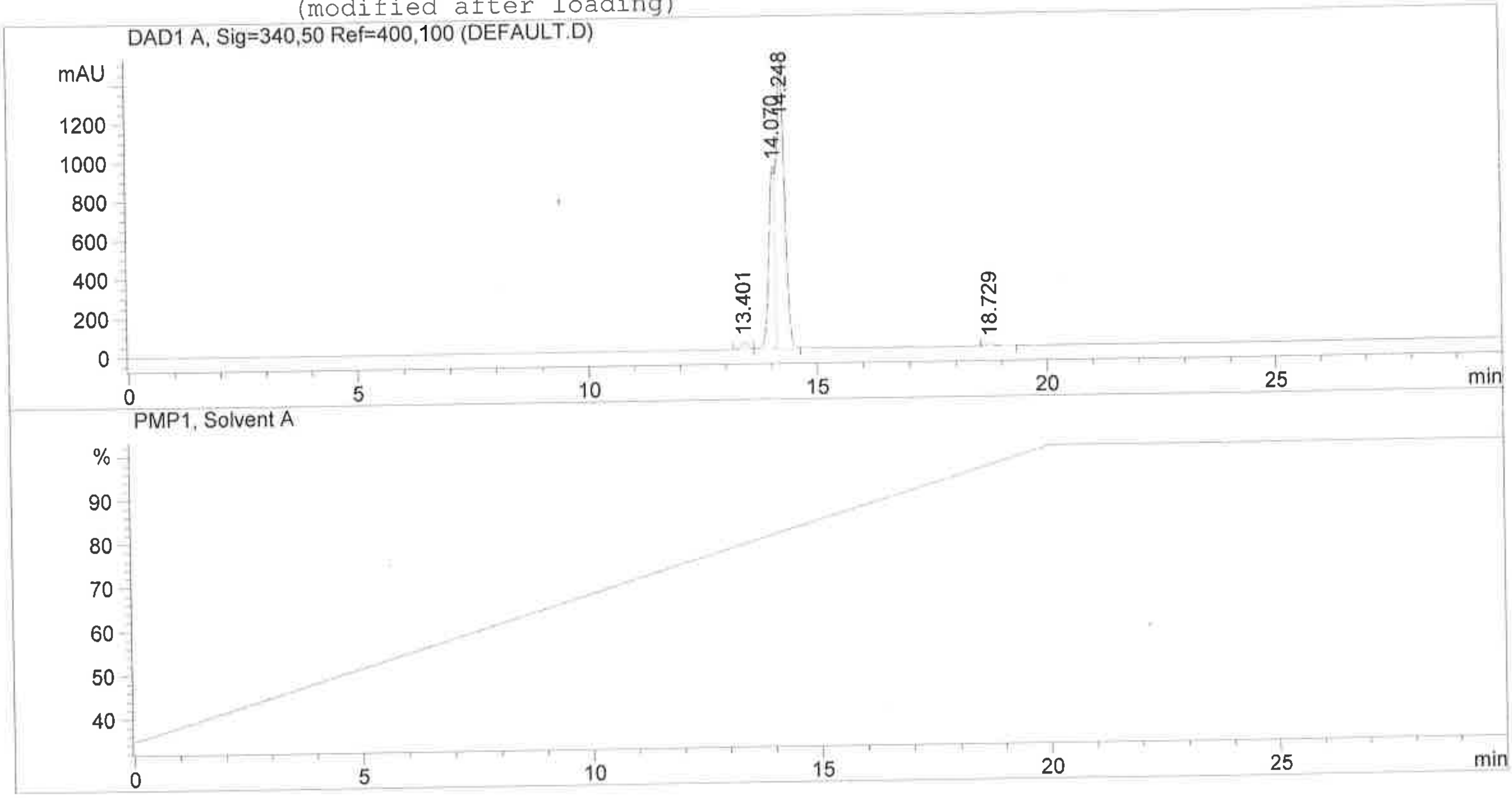

===

Area Percent Report

===

Sorted By : Signal
Multiplier : 1.0000
Dilution : 1.0000

Signal 1: DAD1 A, Sig=340,50 Ref=400,100

Peak #	RetTime [min]	Type	Width [min]	Area [mAU*s]	Height [mAU]	Area %
1	13.401	BV	0.1497	413.61719	40.99300	1.5172
2	14.070	VV	0.1430	8956.49023	941.62994	32.8529
3	14.248	VV	0.1821	1.77211e4	1455.16833	65.0018
4	18.729	BP	0.1426	171.25641	17.44182	0.6282

Totals : 2.72624e4 2455.23310

Results obtained with enhanced integrator!

===

*** End of Report ***

Figure S24: HPLC chromatogram of **15**

SW-85-87-02.2.fid

Figure S25: ^1H NMR spectrum of **16** in $CDCl_3$

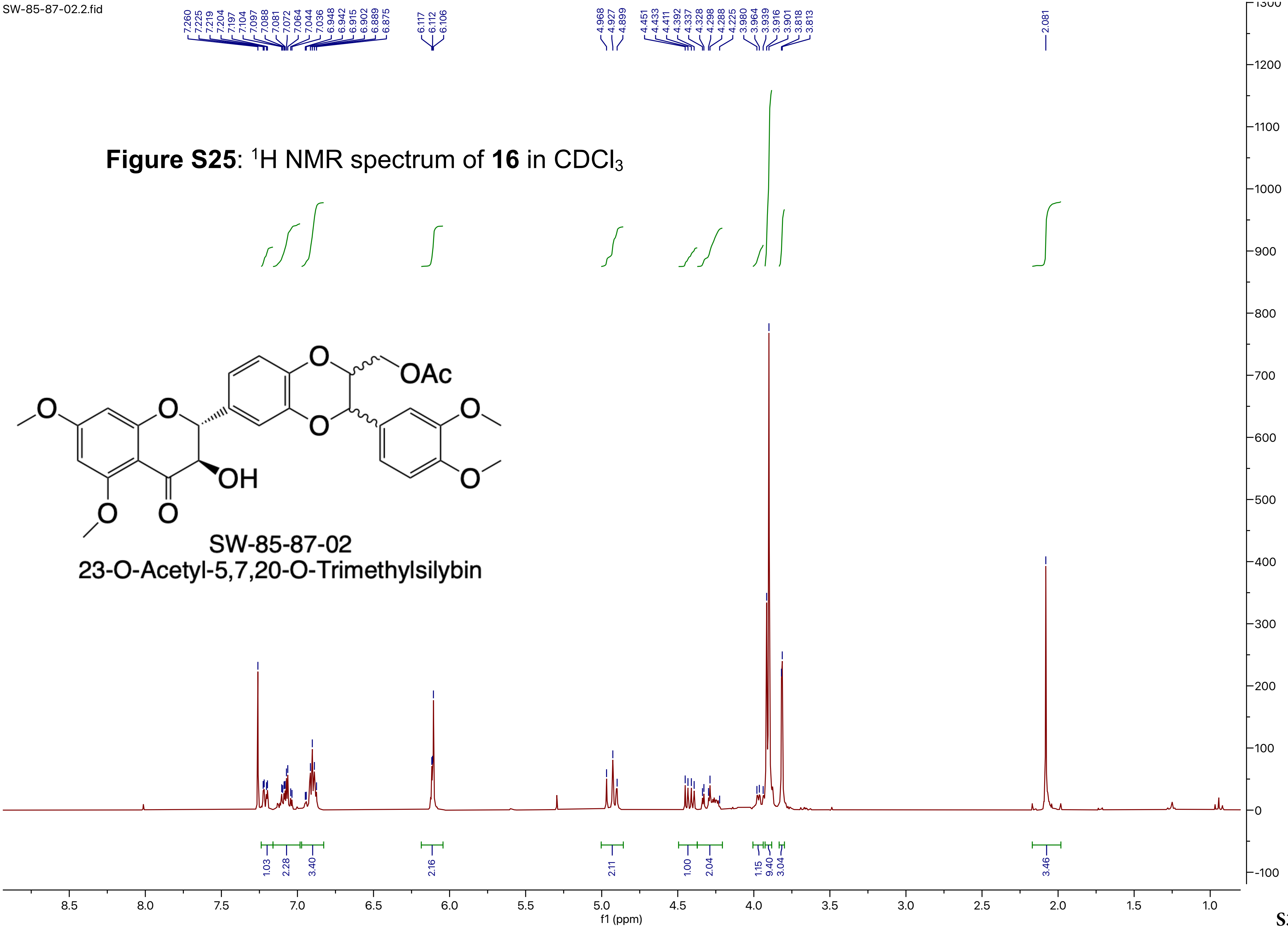

SW-85-87-02

23-O-Acetyl-5,7,20-O-Trimethylsilybin

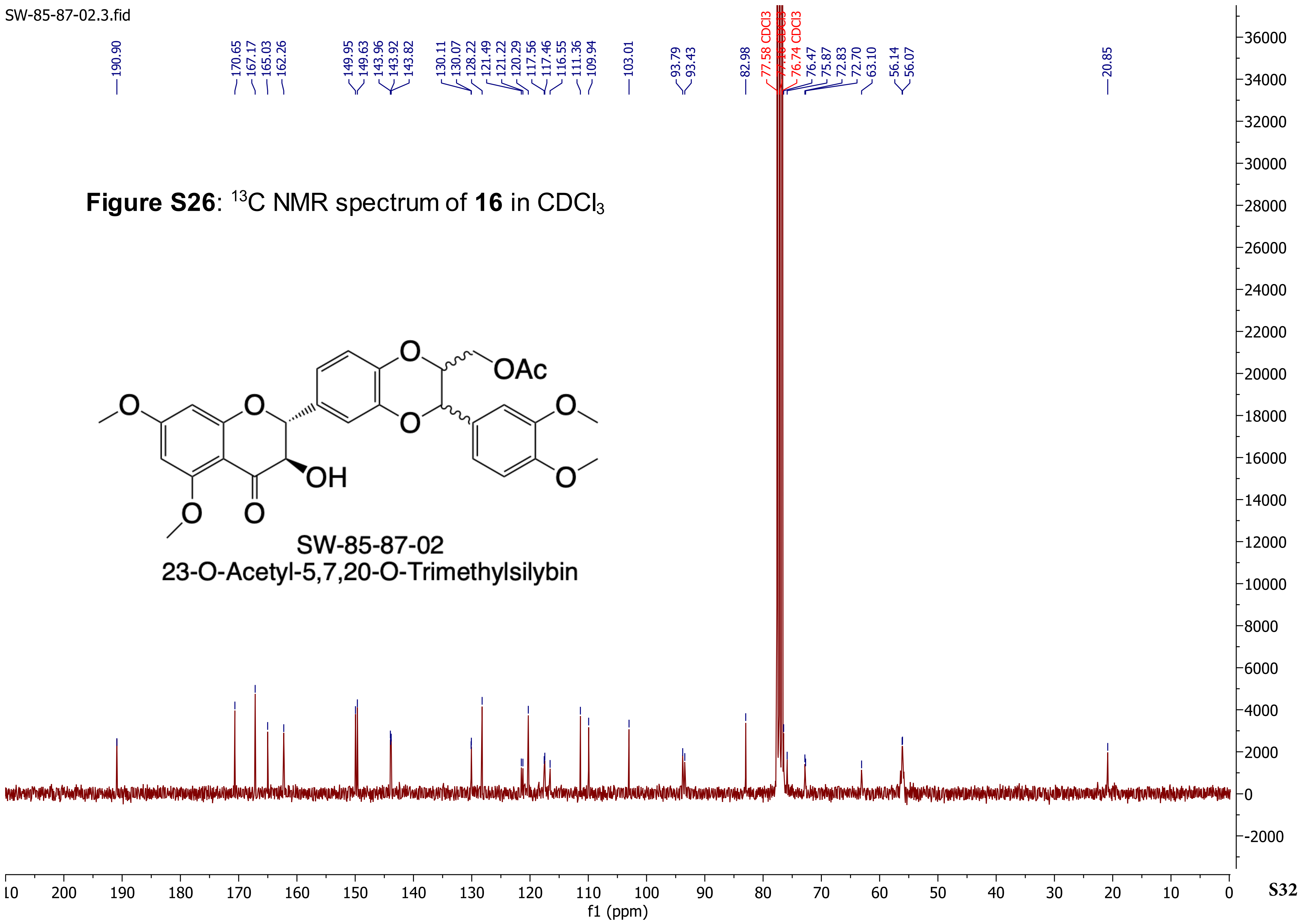

Figure S26: ^{13}C NMR spectrum of **16** in $CDCl_3$

Sample Name	Mol Fomula	MW	M+H	observed	delta	ppm
SW-85-107-01	C30H30O11	566.1789	567.1867	567.1872	0.0005	0.93

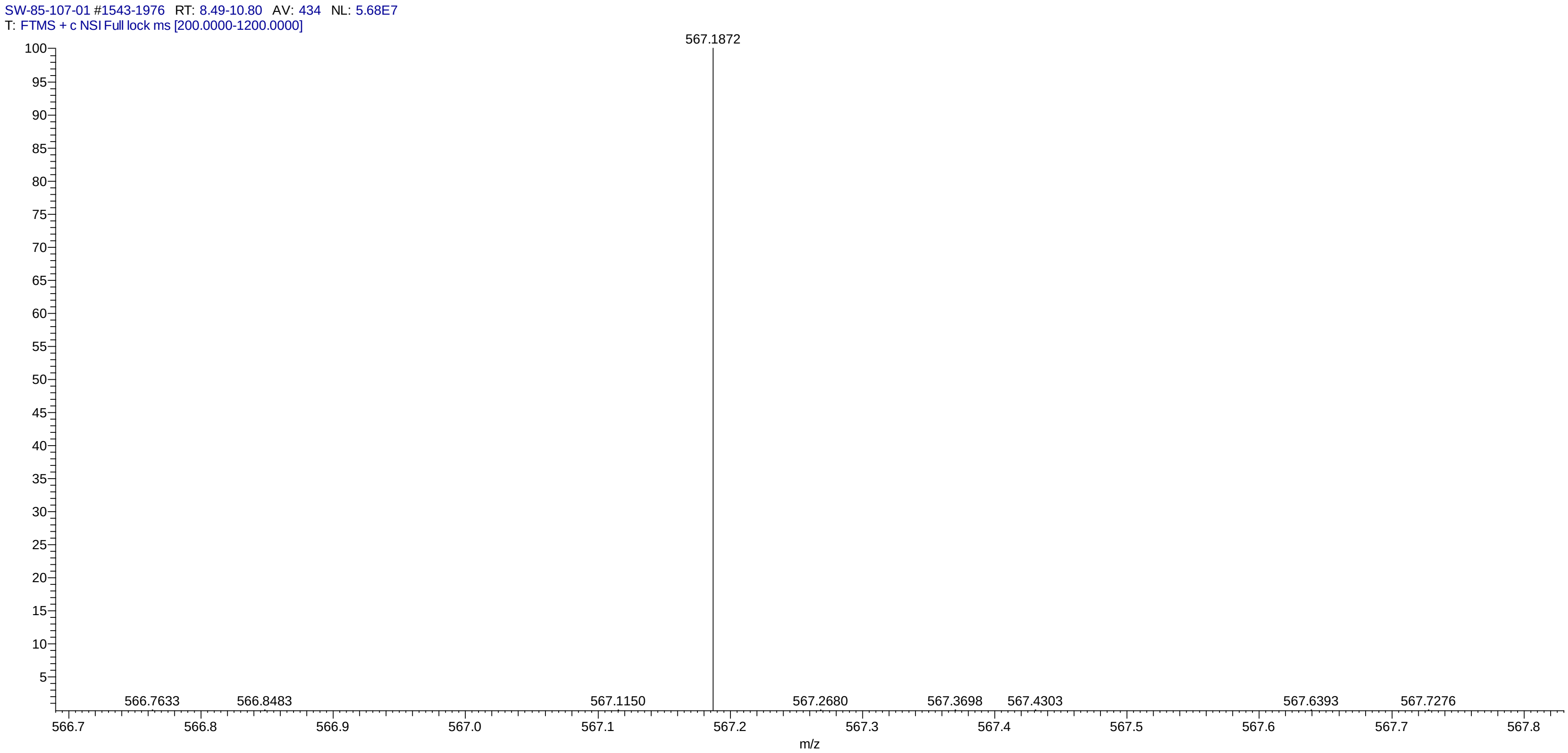

Figure S27: High resolution mass spectrum of **16**

Data File C:\HPCHEM\1\DATA\SW85107.D

Sample Name: SW-85-107

===

Injection Date : 5/1/2022 1:24:14 PM
Sample Name : SW-85-107 Location : Vial 1
Acq. Operator :
Method : C:\HPCHEM\1\METHODS\JNP2015.M
Last changed : 4/30/2022 3:37:52 PM
(modified after loading)

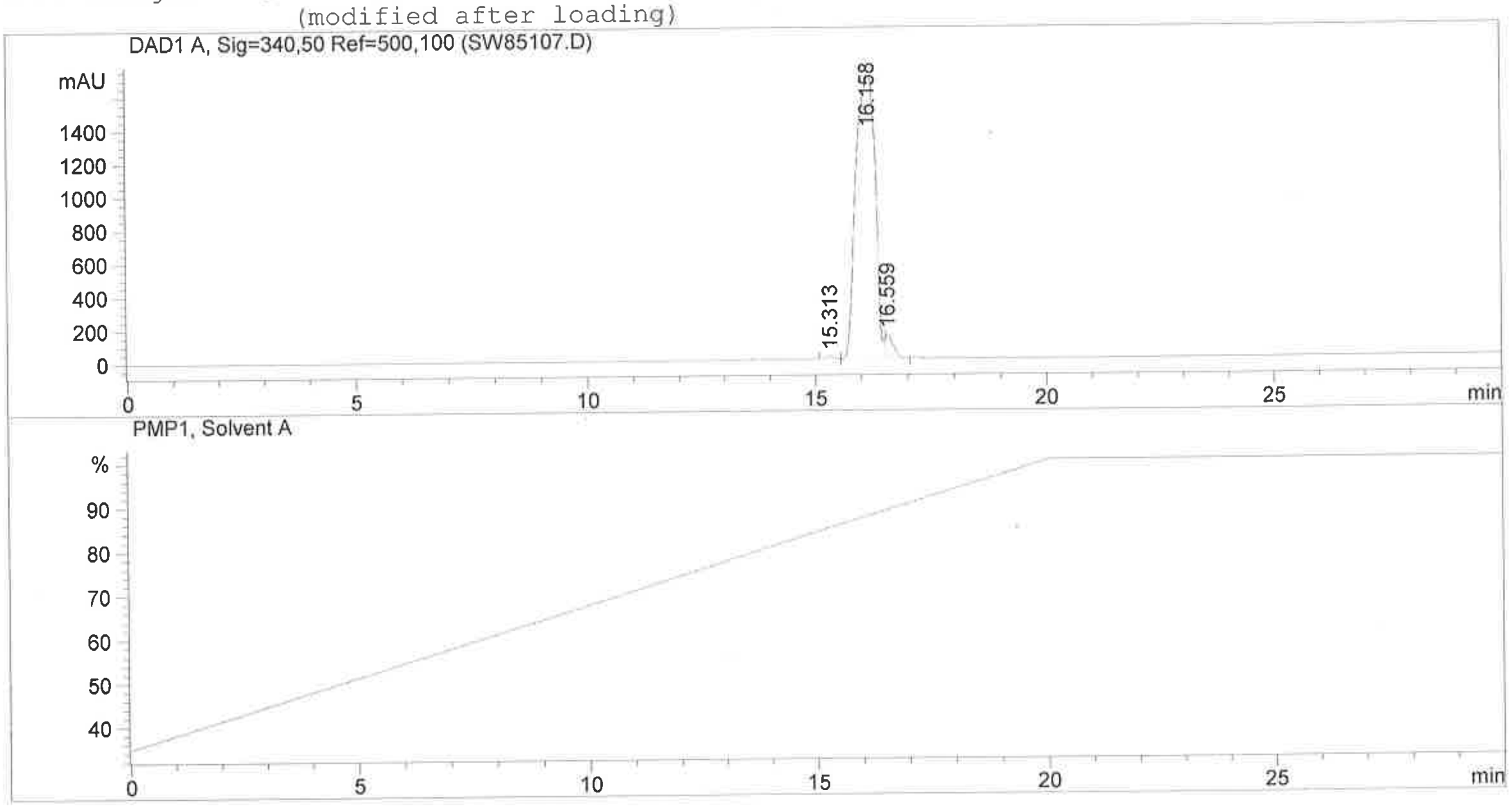

===
Area Percent Report
===

Sorted By : Signal
Multiplier : 1.0000
Dilution : 1.0000

Signal 1: DAD1 A, Sig=340,50 Ref=500,100

Peak #	RetTime [min]	Type	Width [min]	Area [mAU*s]	Height [mAU]	Area %
1	15.313	VV	0.1959	285.76041	21.94092	0.5619
2	16.158	VV	0.4881	4.88366e4	1695.28967	96.0268
3	16.559	VV	0.1587	1734.90137	150.23711	3.4113
Totals :				5.08572e4	1867.46770	

Results obtained with enhanced integrator!

===
*** End of Report ***

Figure S28: HPLC chromatogram of **16**

SW-66-154

Figure S29: ^{1}H NMR spectrum of **17** in $CDCl_3$

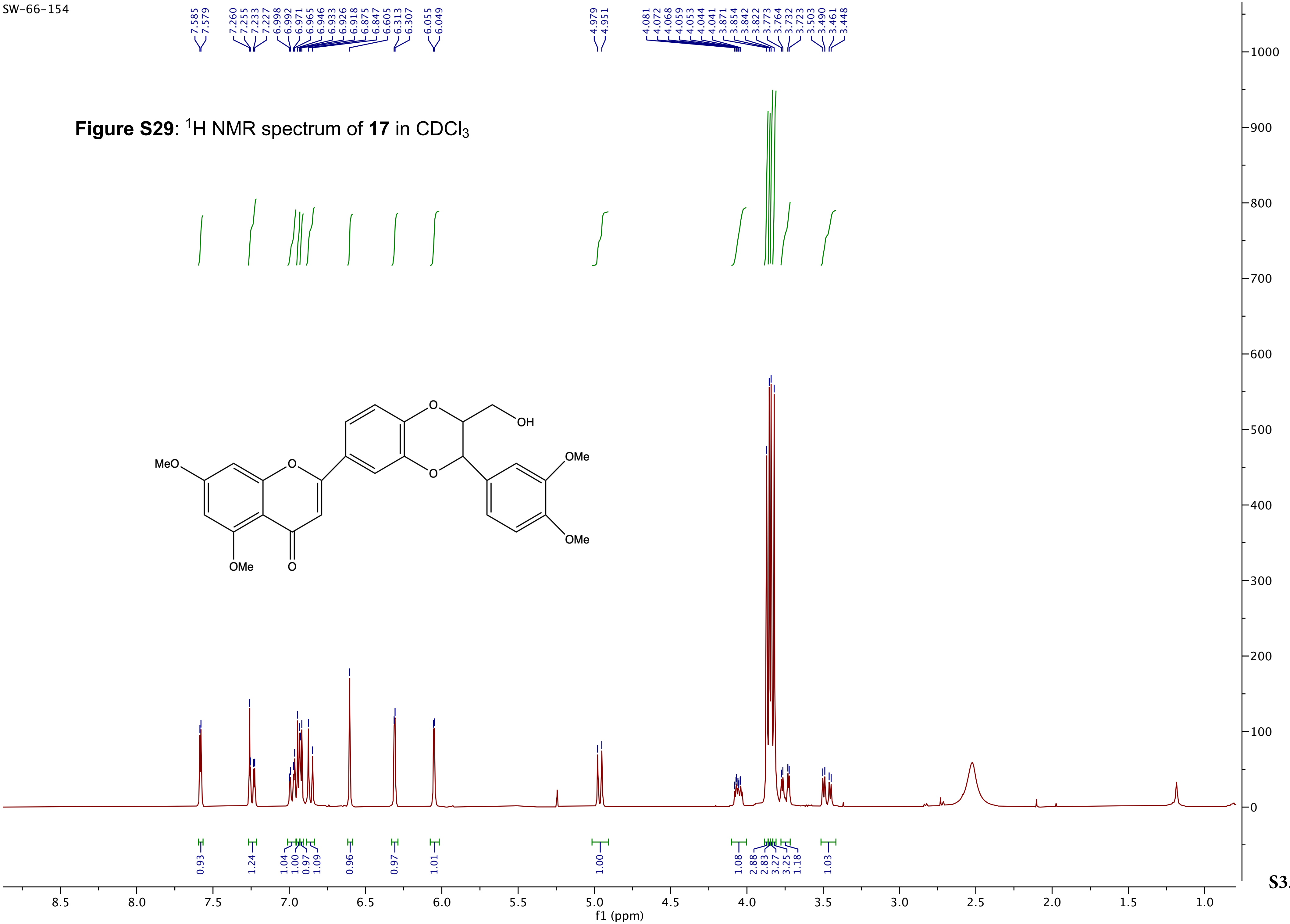

Figure S30: ^{13}C NMR spectrum of **17** in $CDCl_3$+ DMSO-d6 (10:1, v/v)

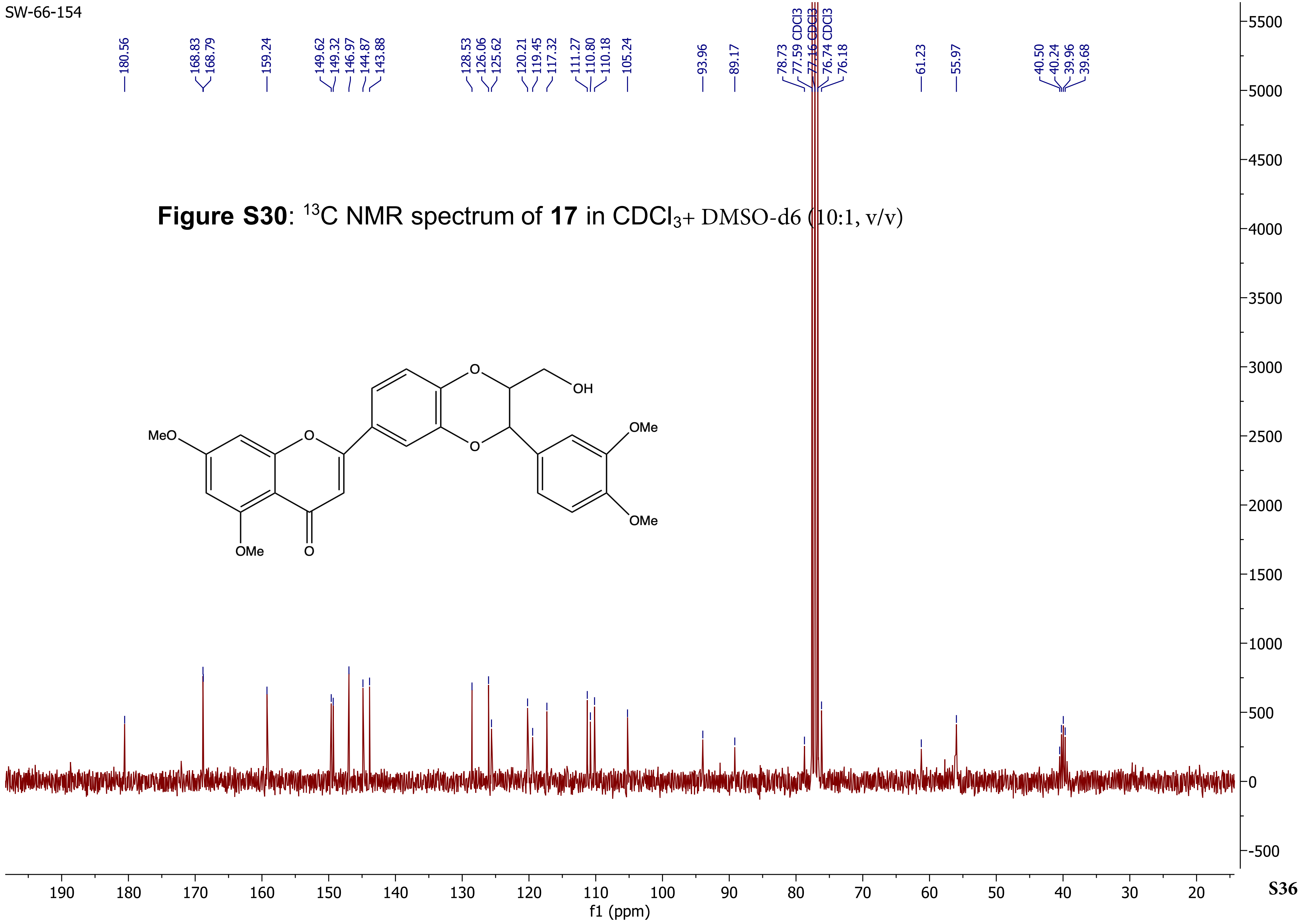

SW-66-154	C28H26O9	506.1577	507.1655	**507.1670**	0.0015	2.90

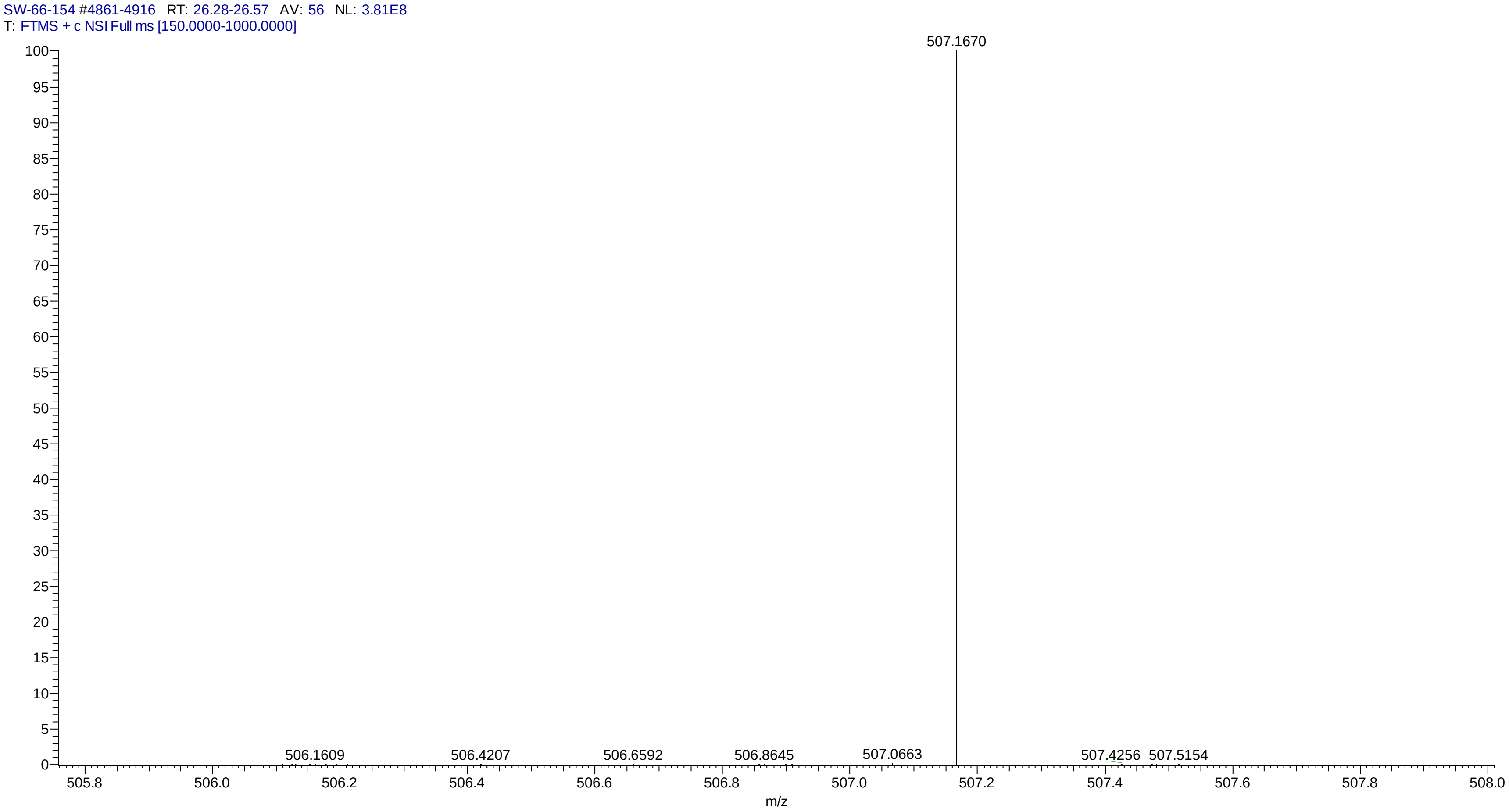

Figure S31: High resolution mass spectrum of **17**

===

Injection Date : 4/29/2022 3:24:31 PM
Sample Name : SW-66-154 Location : Vial 1
Acq. Operator :
Method : C:\HPCHEM\1\METHODS\JNP2015.M
Last changed : 4/29/2022 2:19:03 PM

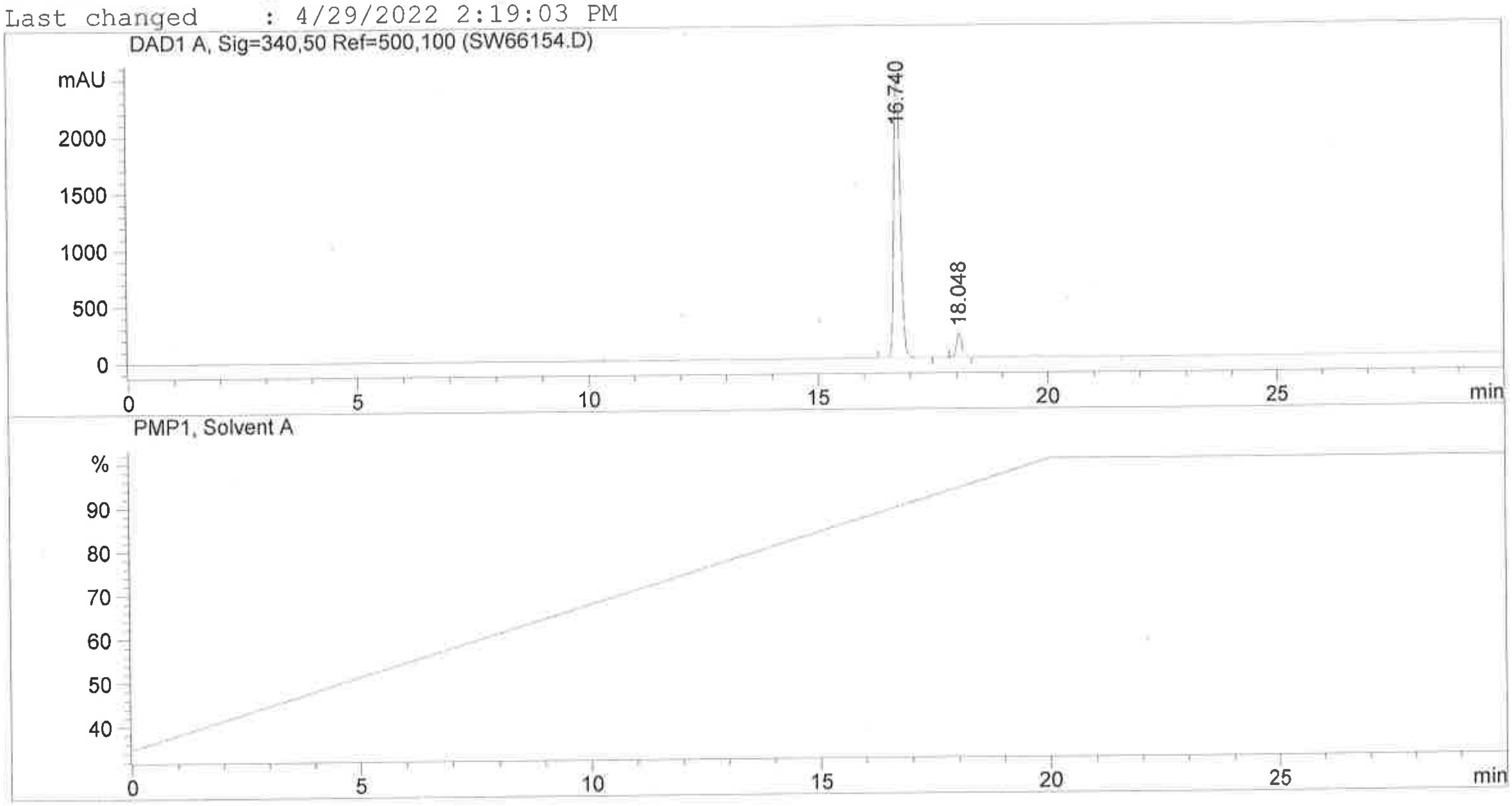

===

Area Percent Report

===

Sorted By : Signal
Multiplier : 1.0000
Dilution : 1.0000

Signal 1: DAD1 A, Sig=340,50 Ref=500,100

Peak #	RetTime [min]	Type	Width [min]	Area [mAU*s]	Height [mAU]	Area %
1	16.740	VV	0.1547	2.45456e4	2496.43213	93.3453
2	18.048	VV	0.1210	1749.89453	219.59286	6.6547

Totals : 2.62955e4 2716.02499

Results obtained with enhanced integrator!

===

*** End of Report ***

Figure S32: HPLC chromatogram of **17**

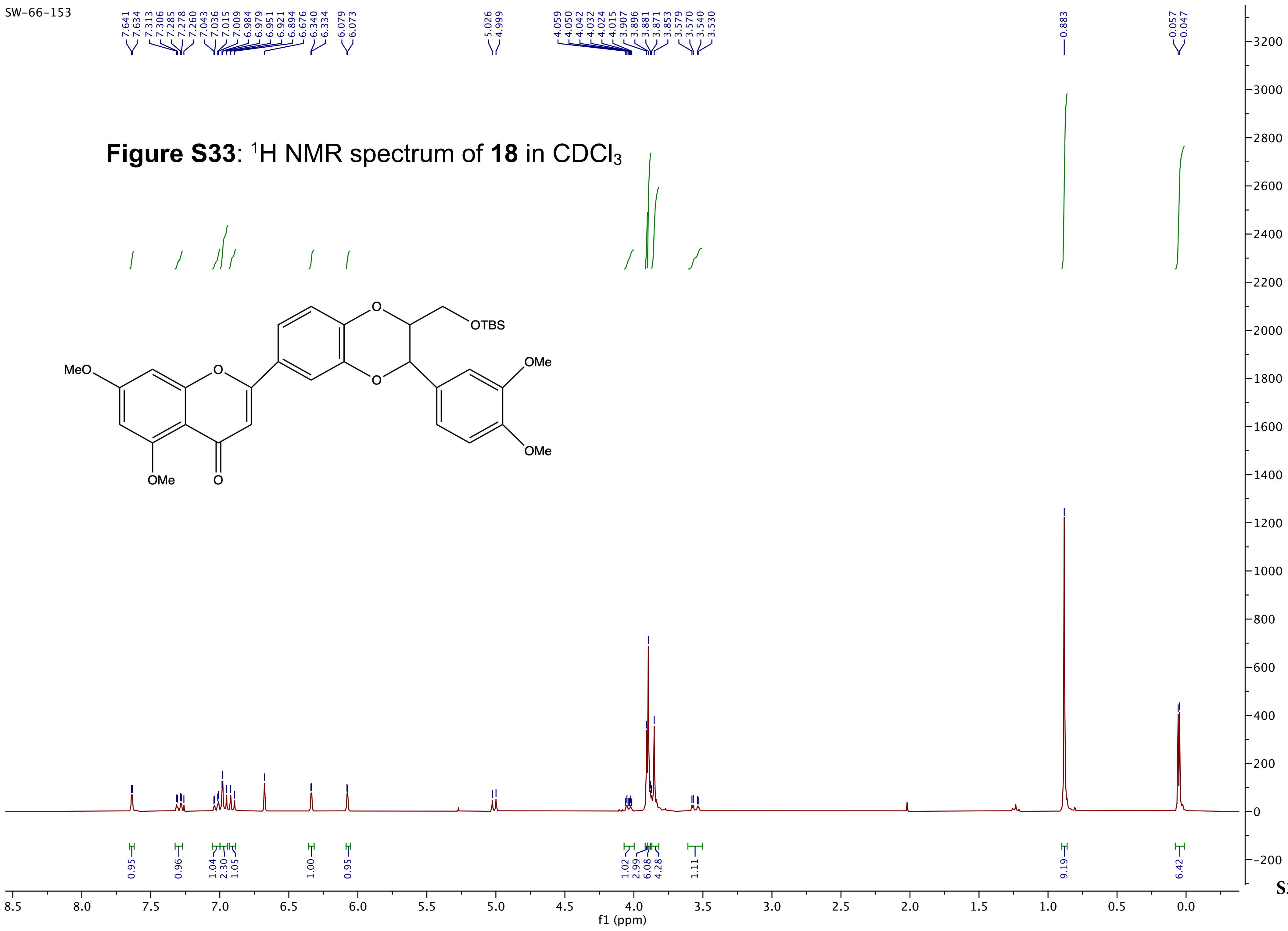

Figure S33: ^{1}H NMR spectrum of **18** in $CDCl_3$

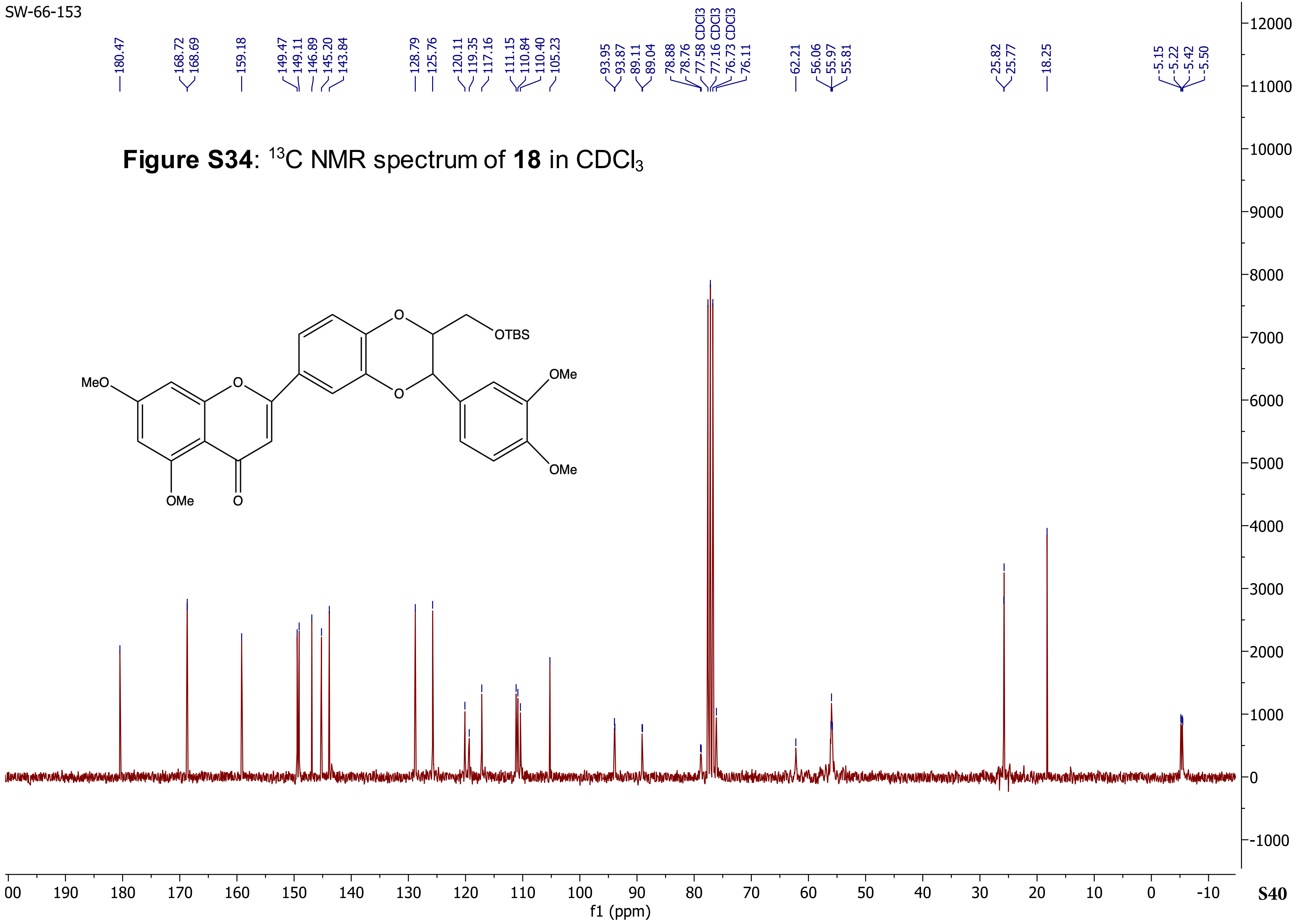

Figure S34: ^{13}C NMR spectrum of **18** in $CDCl_3$

SW-66-153	C34H40O9Si	620.2442	621.2520	**621.2530**	0.0010	1.59

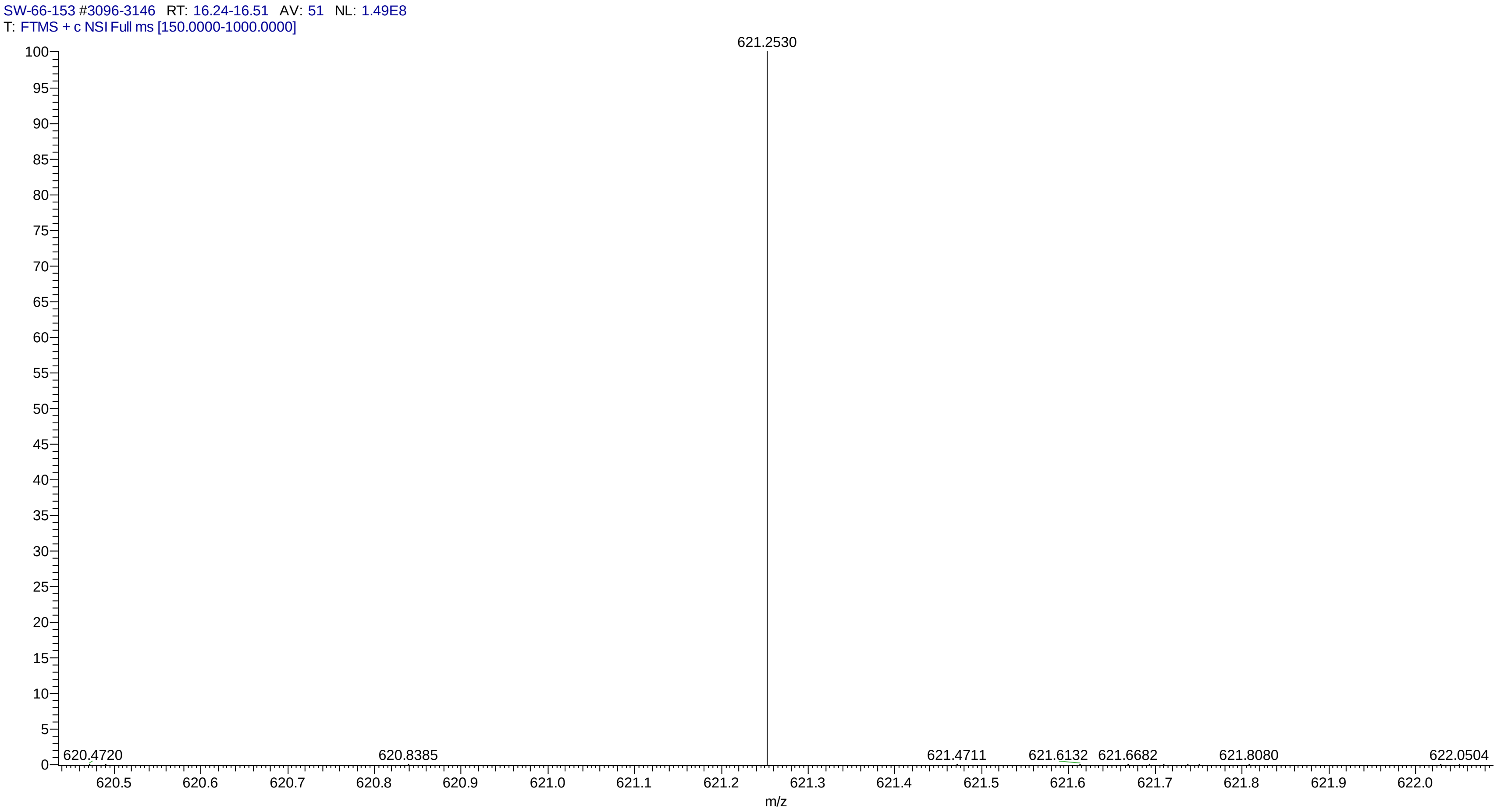

Figure S35: High resolution mass spectrum of **18**

```
=====================================================================
Injection Date  : 2/9/2023 11:58:06 AM
Sample Name     : SW-66-153                    Location : Vial 1
Acq. Operator   :
Method          : C:\HPCHEM\1\METHODS\JNP2015.M
Last changed    : 2/9/2023 9:06:13 AM
                  (modified after loading)
```

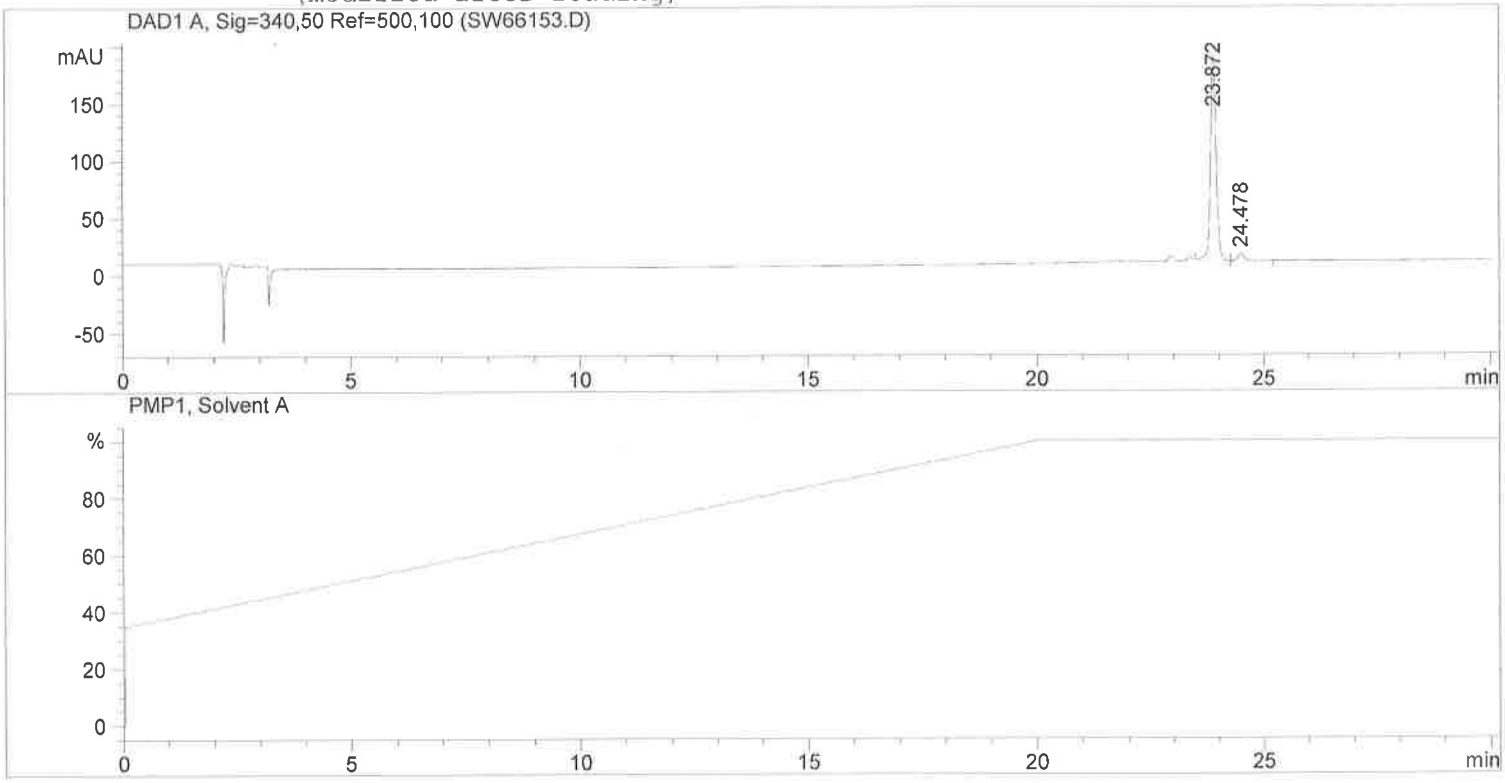


```
=====================================================================
                        Area Percent Report
=====================================================================

Sorted By             :      Signal
Multiplier            :      1.0000
Dilution              :      1.0000

Signal 1: DAD1 A, Sig=340,50 Ref=500,100
```

Peak #	RetTime [min]	Type	Width [min]	Area [mAU*s]	Height [mAU]	Area %
1	23.872	VV	0.1346	1577.18994	179.26302	95.4221
2	24.478	VP	0.1647	75.66637	6.65224	4.5779

Totals : 1652.85631 185.91526

Results obtained with enhanced integrator!

```
=====================================================================
                        *** End of Report ***
```

Figure S36: HPLC chromatogram of **18**

SW-66-148

Figure S37: ^{1}H NMR spectrum of **19** in $CDCl_3$

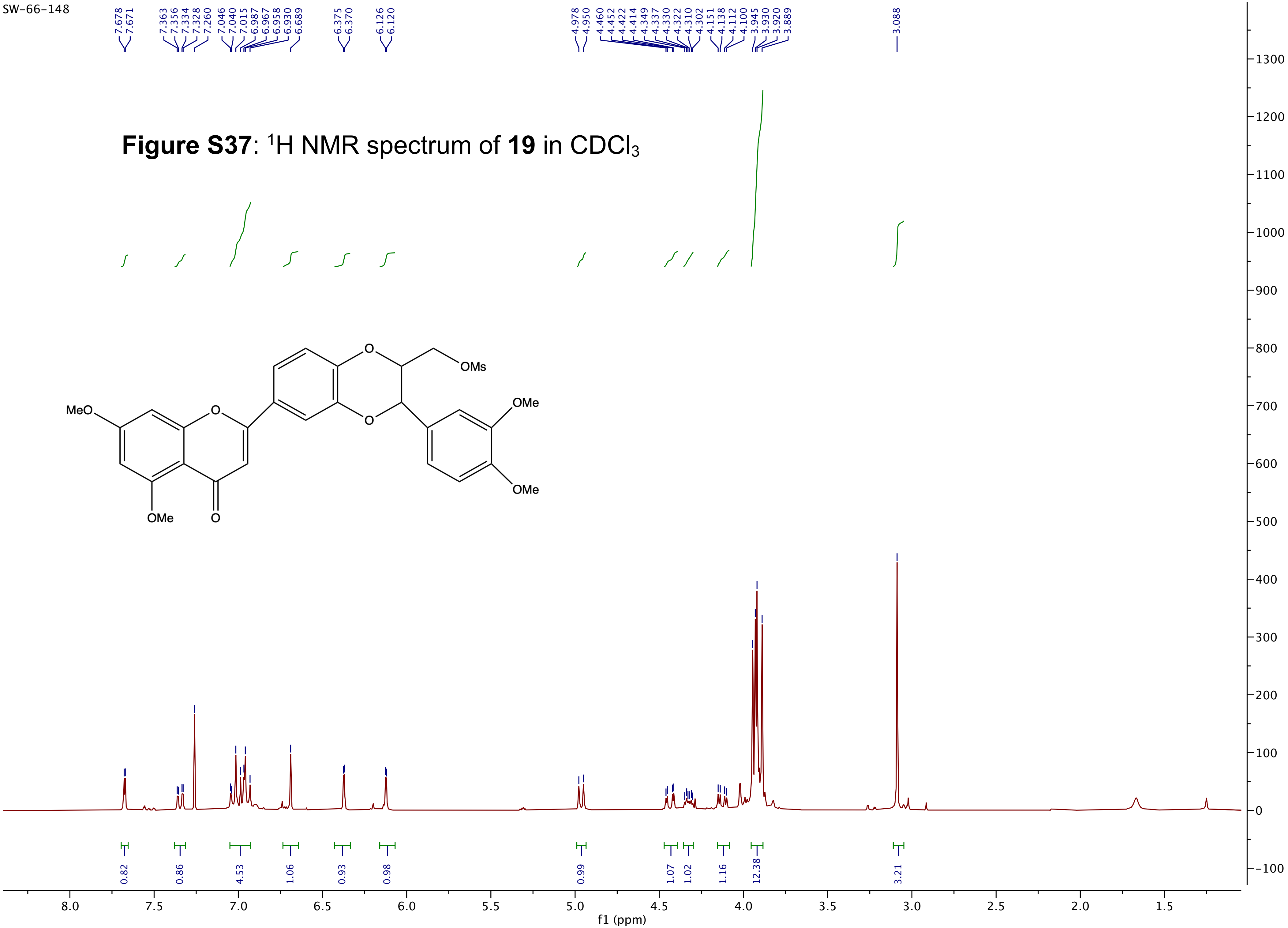

SW-66-148

Figure S38: ^{13}C NMR spectrum of **19** in $CDCl_3$

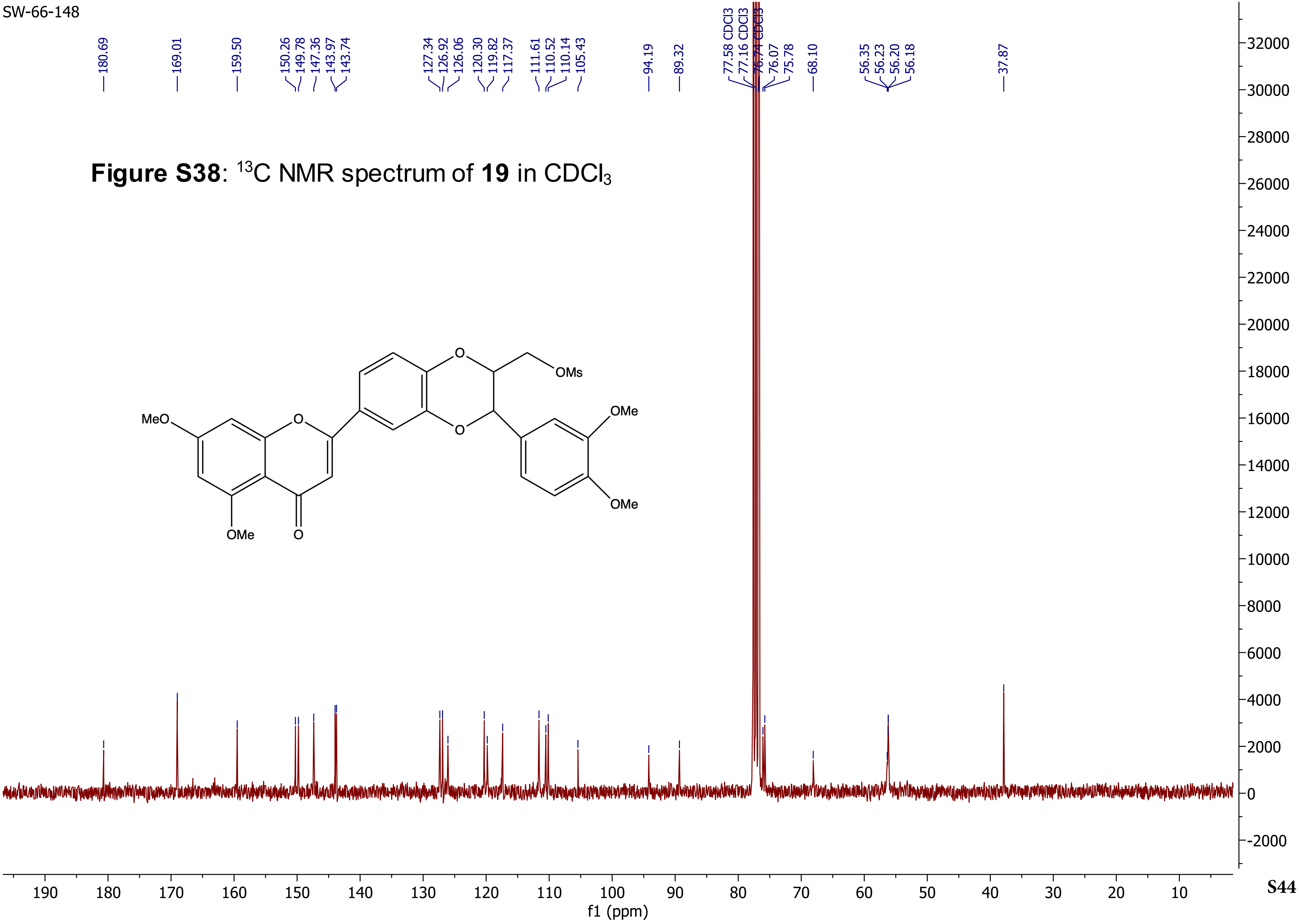

SW-66-148	C29H28O11S	584.1353	585.1431	**585.1430**	-0.0001	-0.15

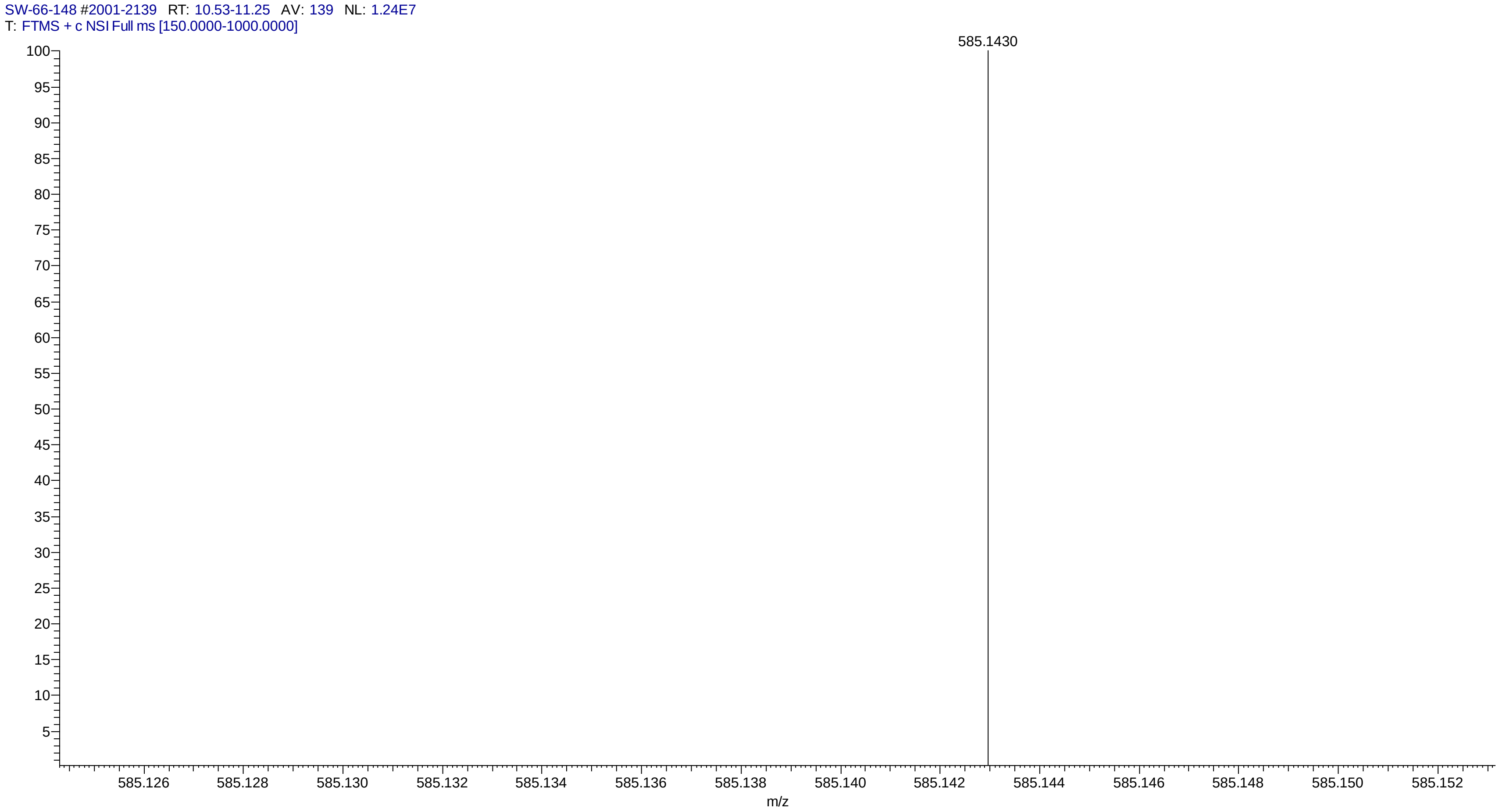

Figure S39: High resolution mass spectrum of **19**

```
=====================================================================
Injection Date  : 2/10/2023 11:09:26 AM
Sample Name     : SW-66-148                   Location : Vial 1
Acq. Operator   :
Method          : C:\HPCHEM\1\METHODS\JNP2015.M
Last changed    : 2/9/2023 3:27:35 PM
```

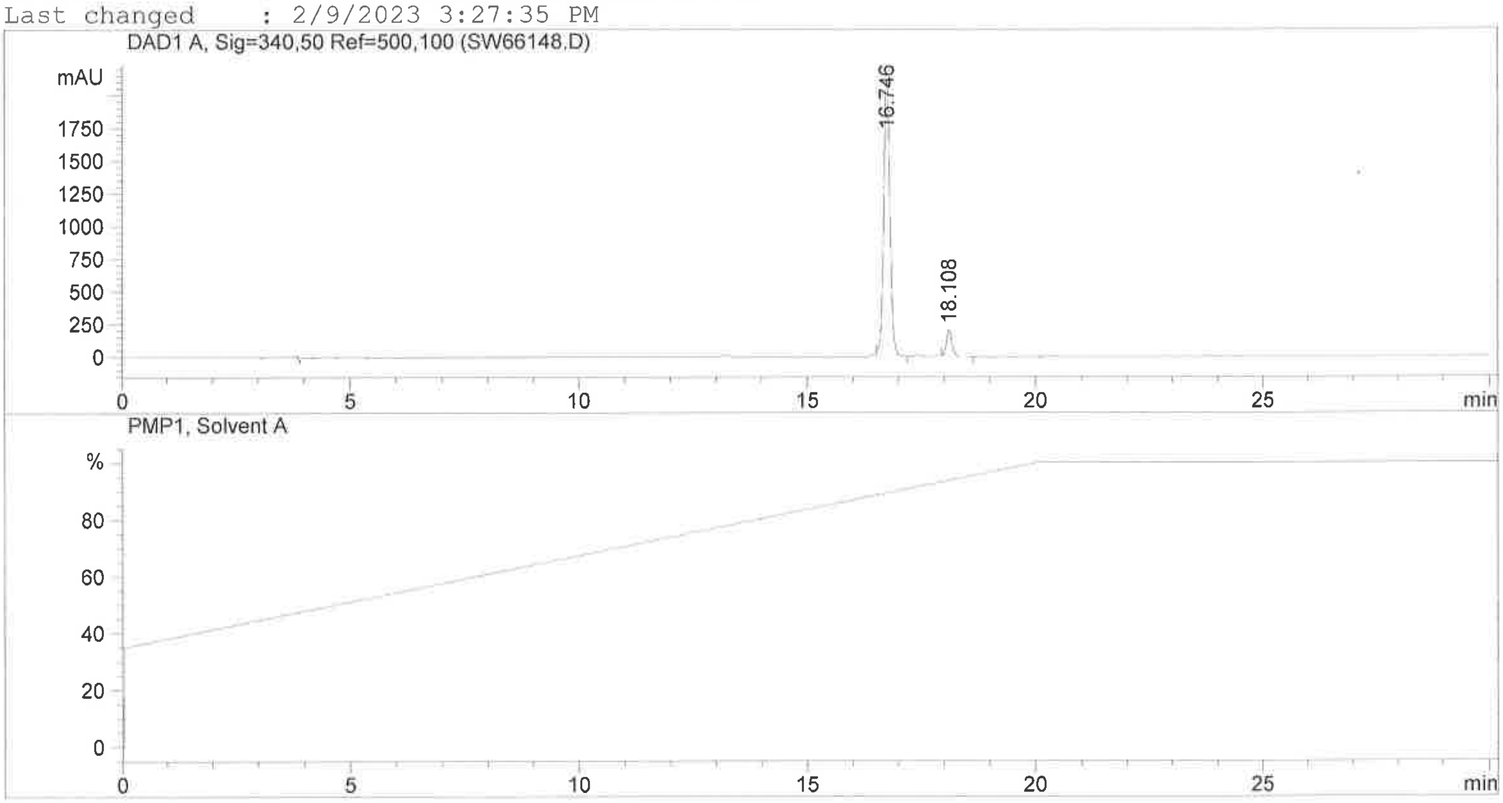


```
=====================================================================
                     Area Percent Report
=====================================================================

Sorted By             :      Signal
Multiplier            :      1.0000
Dilution              :      1.0000

Signal 1: DAD1 A, Sig=340,50 Ref=500,100
```

Peak #	RetTime [min]	Type	Width [min]	Area [mAU*s]	Height [mAU]	Area %
1	16.746	BV	0.1484	2.04009e4	2116.94556	92.1364
2	18.108	VP	0.1287	1741.16675	205.84135	7.8636
Totals :				2.21421e4	2322.78691	

Results obtained with enhanced integrator!

===

*** End of Report ***

Figure S40: HPLC chromatigram of **19**

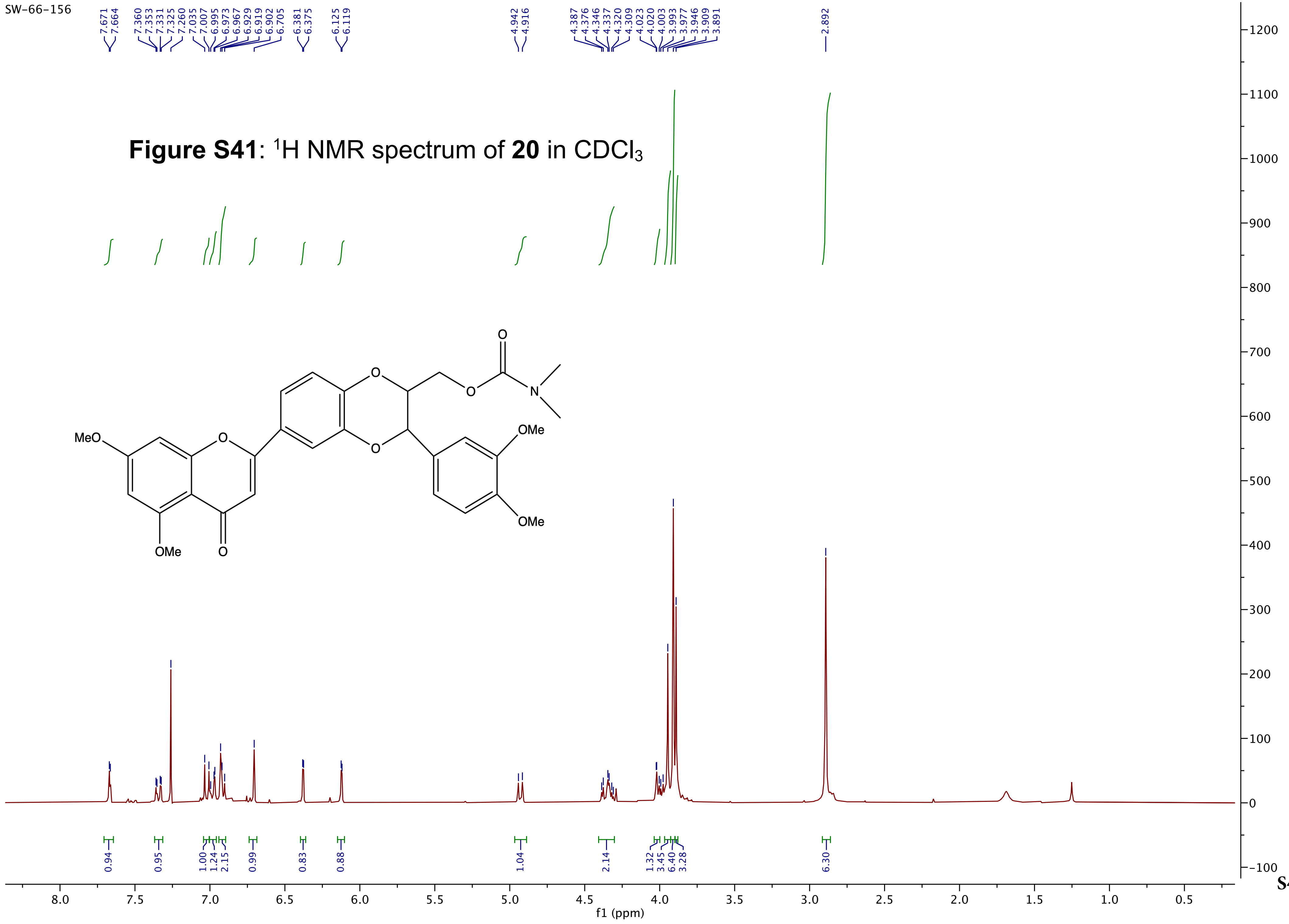

Figure S41: ^{1}H NMR spectrum of **20** in $CDCl_3$

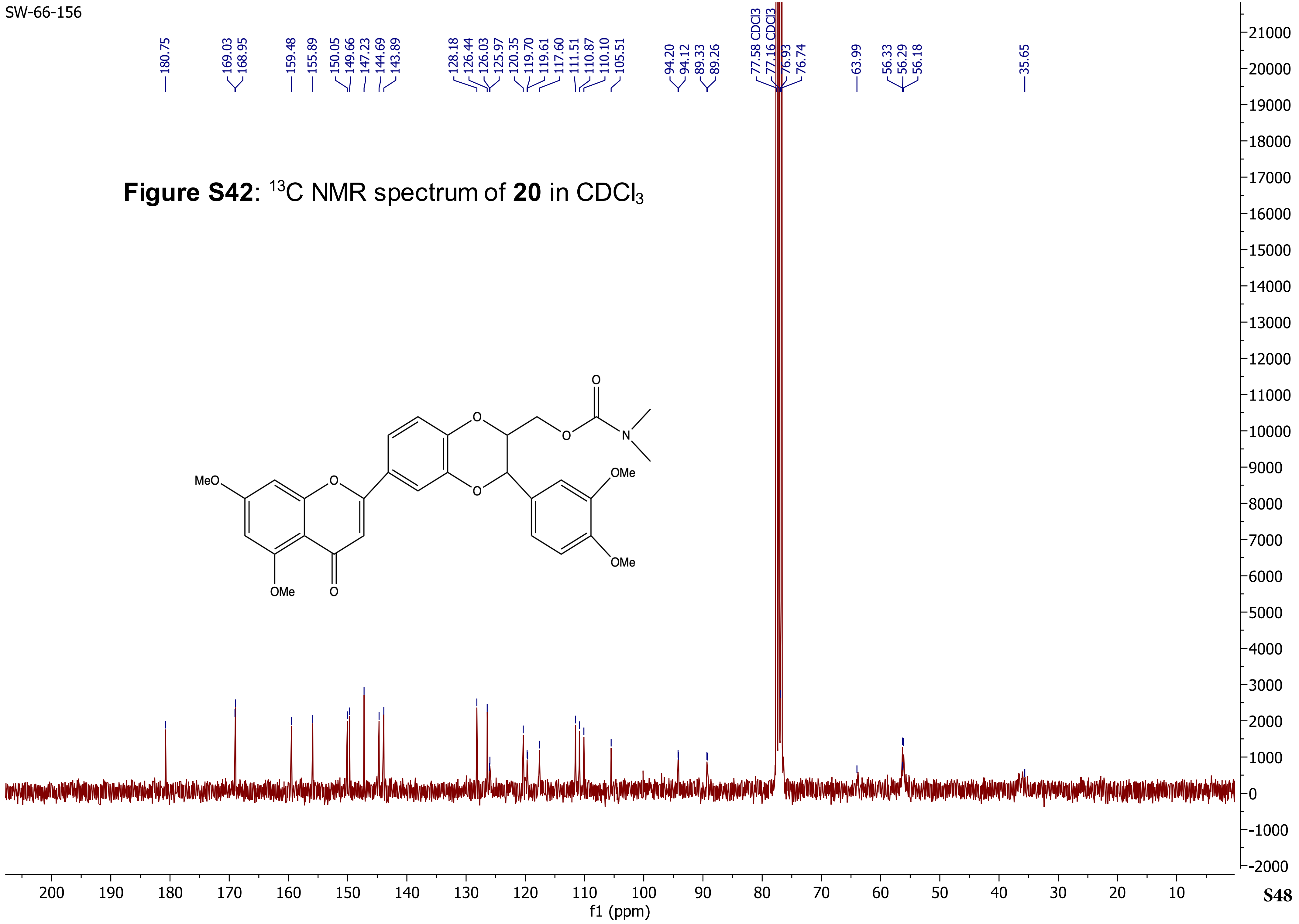

Figure S42: ^{13}C NMR spectrum of **20** in $CDCl_3$

Sample Name	Mol Fomula	MW	M+H	observed	delta	ppm
SW-66-156	C31H31NO10	577.1949	578.2027	578.2026	0.0000	-0.09

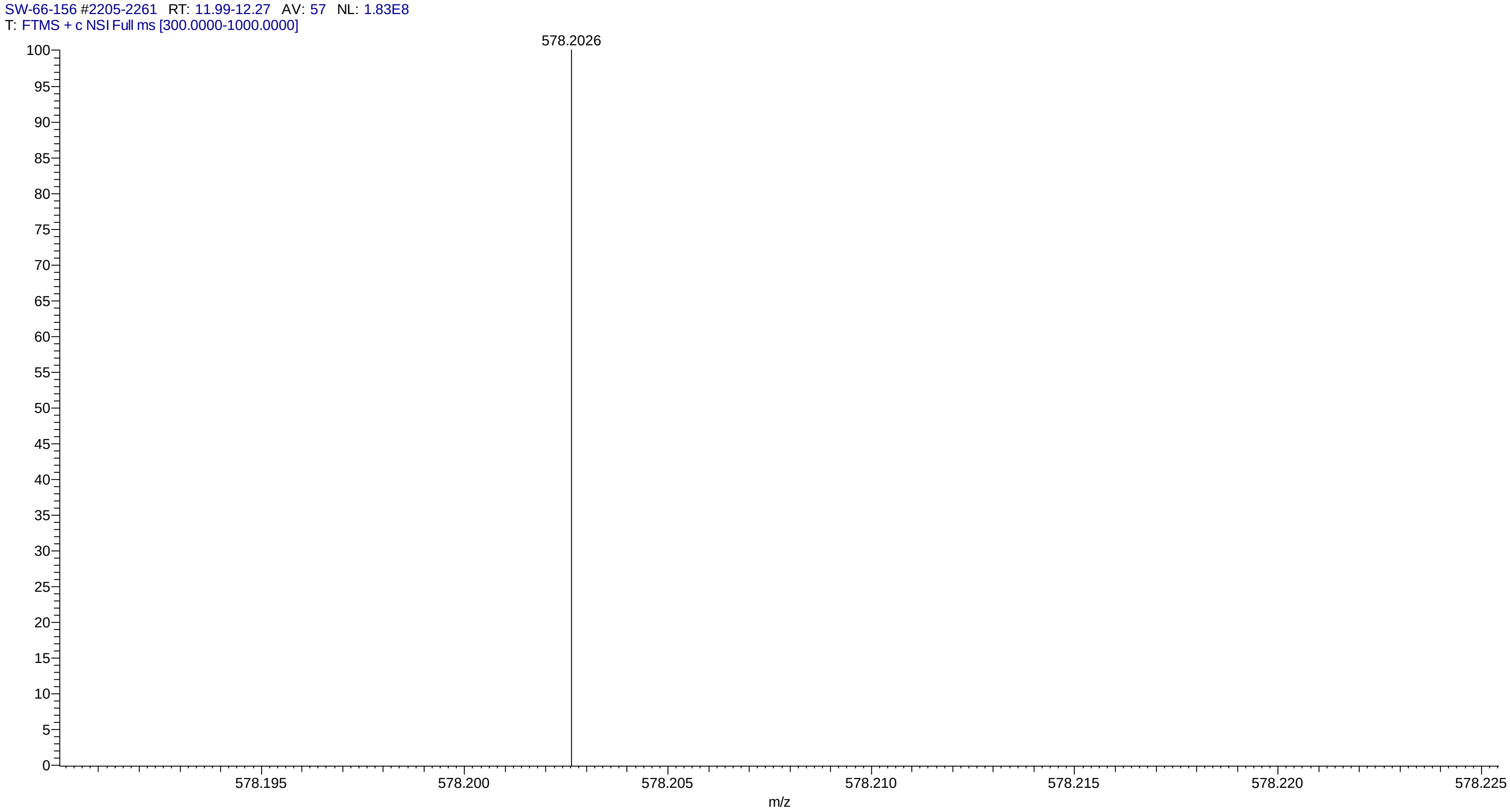

Figure S43: High resolution mass spectrum of **20**

===

Injection Date : 2/11/2023 1:12:49 PM
Sample Name : SW-66-~~156~~ 165 Location : Vial 1
Acq. Operator :
Method : C:\HPCHEM\1\METHODS\JNP2015.M
Last changed : 2/9/2023 3:27:35 PM

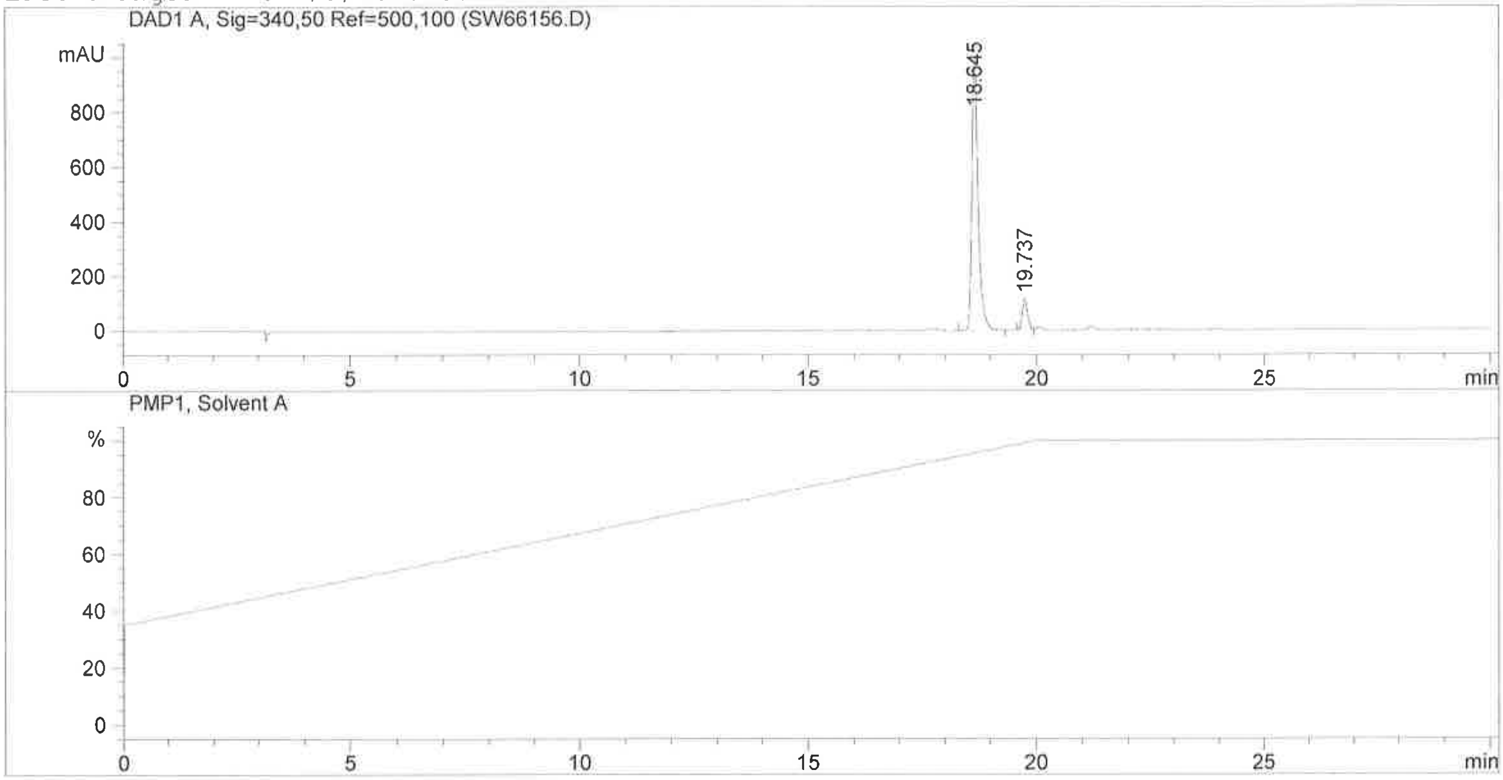

===

Area Percent Report

===

Sorted By : Signal
Multiplier : 1.0000
Dilution : 1.0000

Signal 1: DAD1 A, Sig=340,50 Ref=500,100

Peak #	RetTime [min]	Type	Width [min]	Area [mAU*s]	Height [mAU]	Area %
1	18.645	VB	0.1419	9441.60840	1002.03442	91.1191
2	19.737	PV	0.1216	920.22937	114.67751	8.8809

Totals : 1.03618e4 1116.71193

Results obtained with enhanced integrator!

===

*** End of Report ***

Figure S44: HPLC chromatogram of **20**

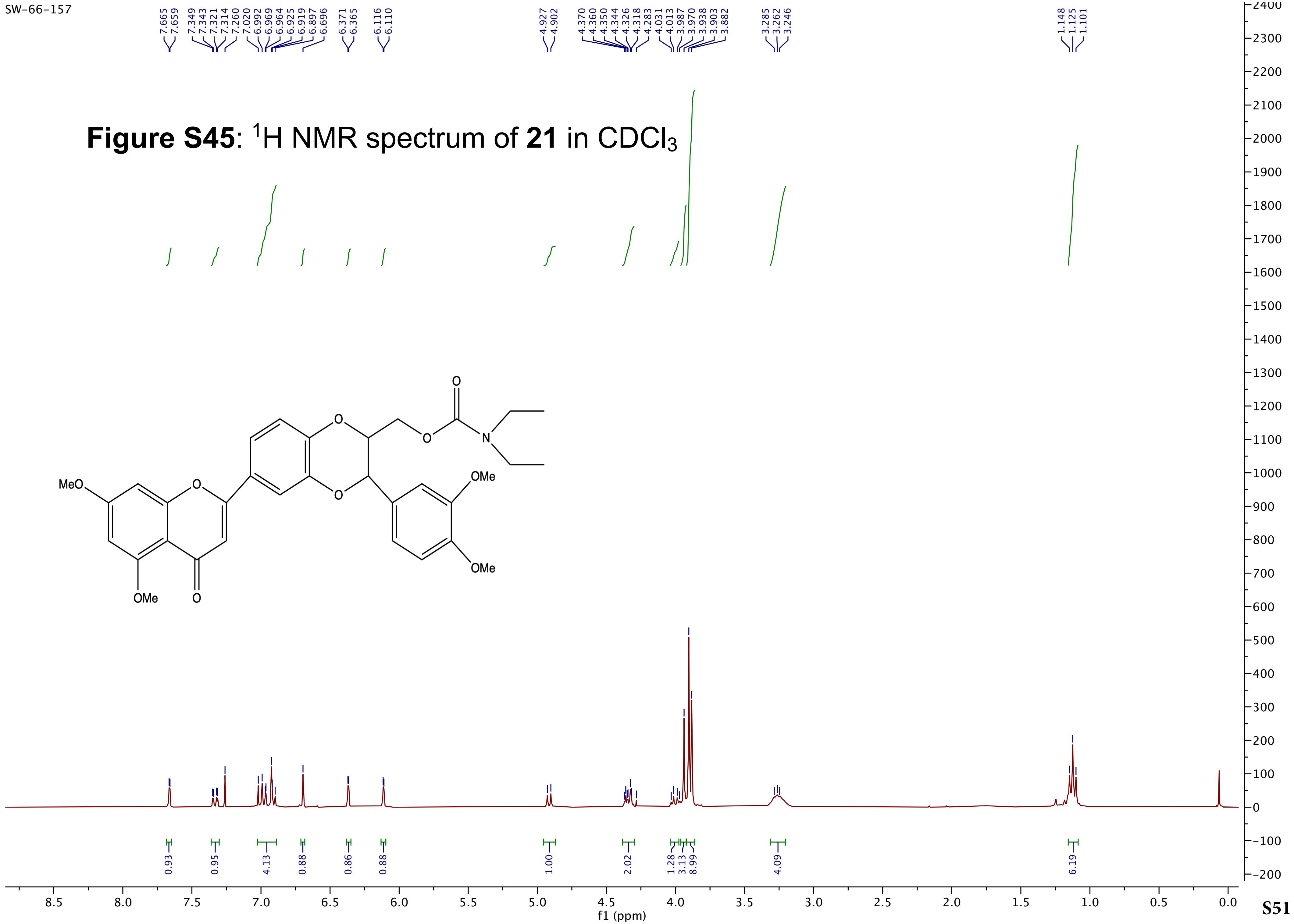

Figure S45: ^{1}H NMR spectrum of **21** in $CDCl_3$

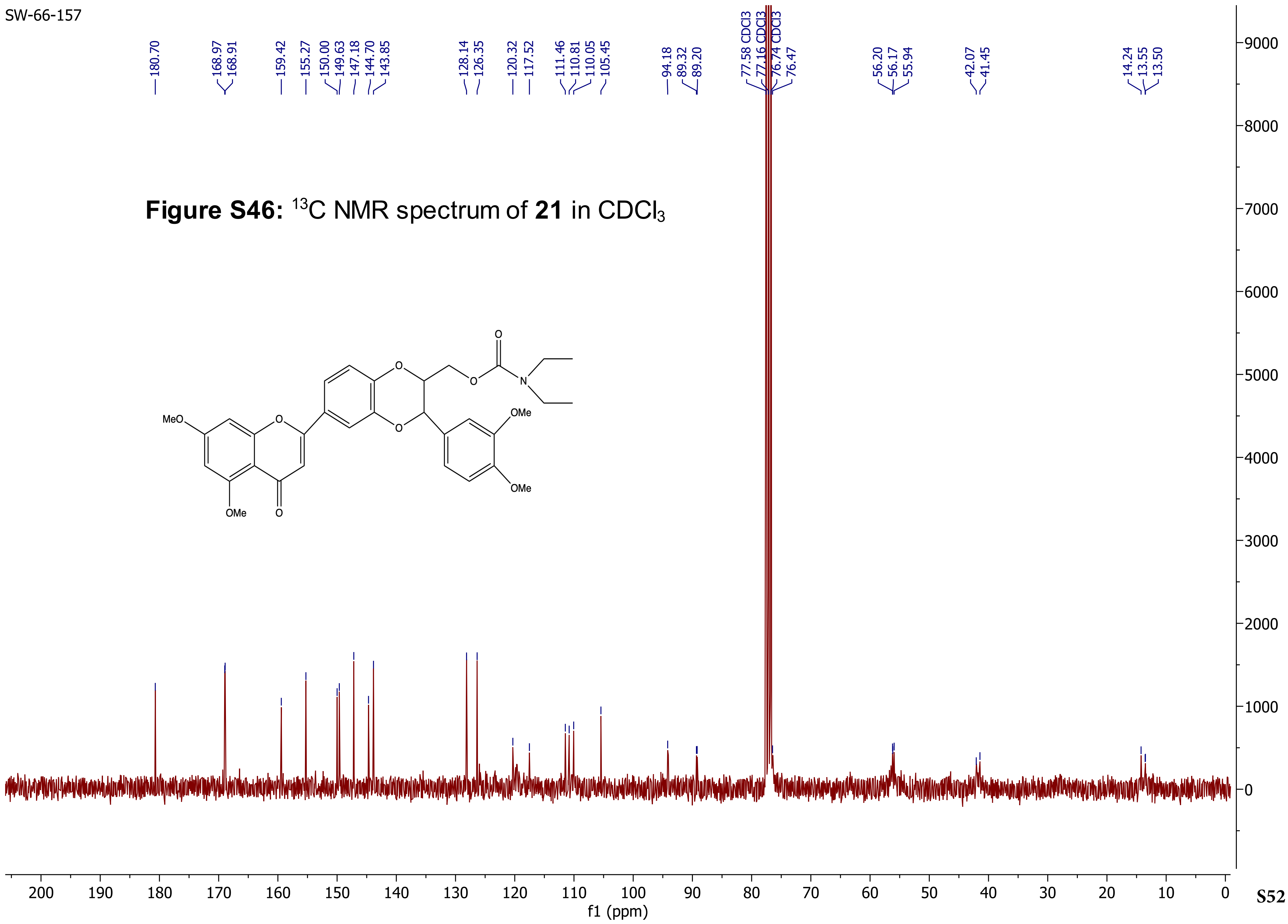

Figure S46: ^{13}C NMR spectrum of **21** in $CDCl_3$

Sample Name	Mol Fomula	MW	M+H	observed	delta	ppm
SW-66-157	C33H35NO10	605.2261	606.2339	606.2339	0.0000	0.00

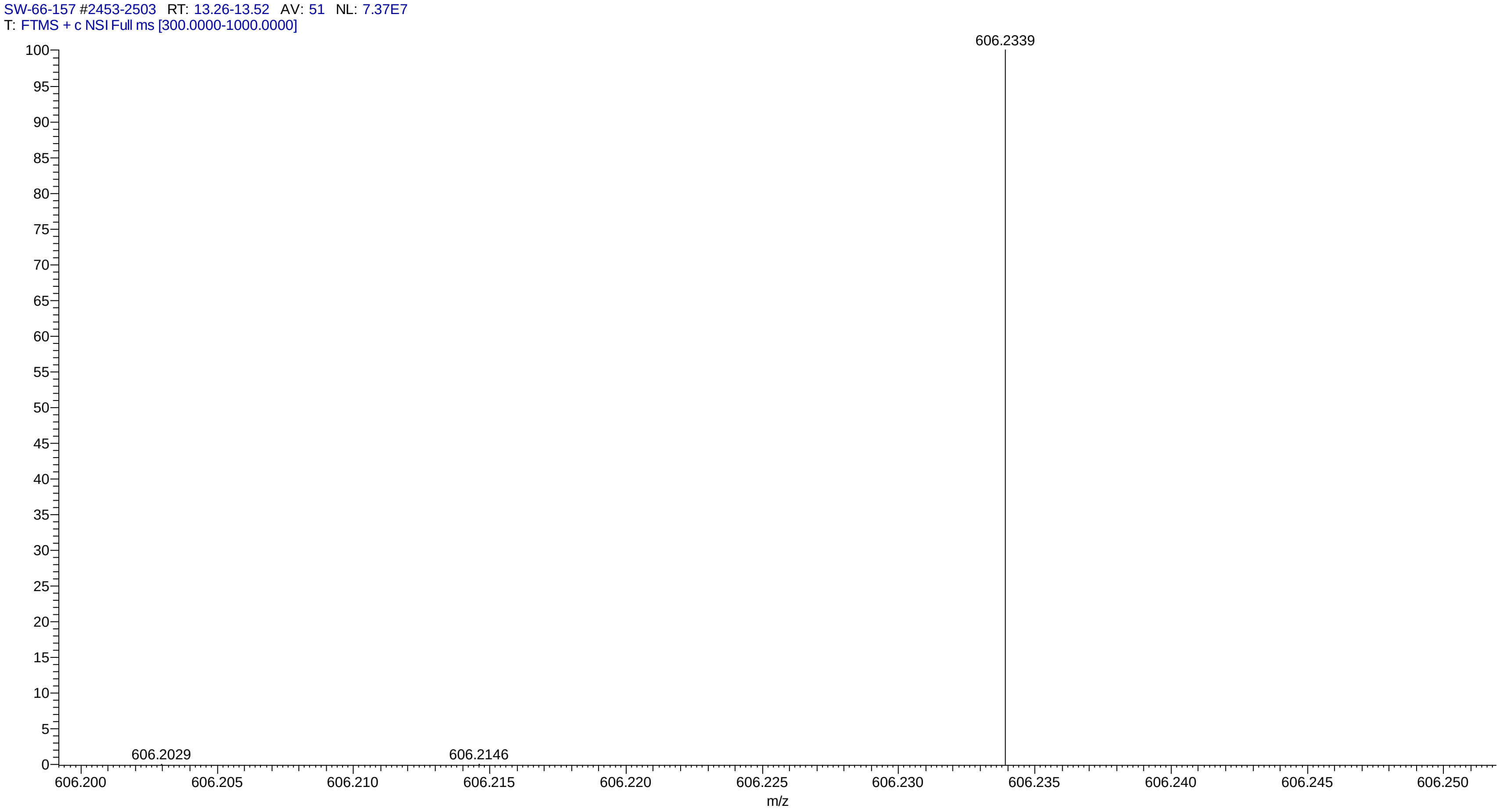

Figure S47: High resolution mass spectrum of **21**

===

Injection Date : 4/29/2022 1:24:59 PM
Sample Name : SW-66-157 Location : Vial 1
Acq. Operator :
Acq. Method : C:\HPCHEM\1\METHODS\JNP2015.M
Last changed : 4/29/2022 9:21:10 AM
(modified after loading)
Analysis Method : C:\HPCHEM\1\METHODS\JNP2015.M
Last changed : 4/29/2022 2:19:03 PM

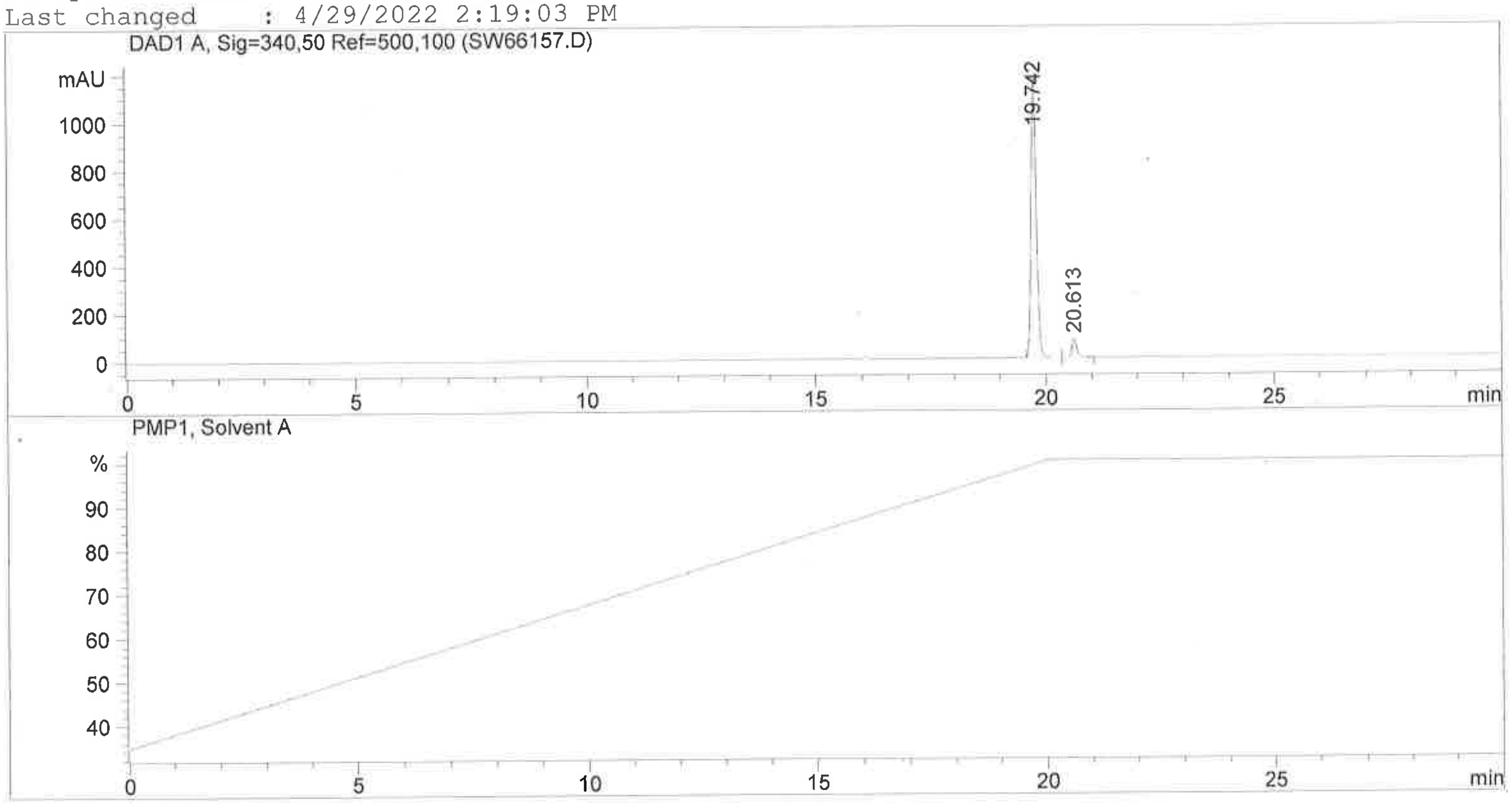

===
Area Percent Report
===

Sorted By : Signal
Multiplier : 1.0000
Dilution : 1.0000

Signal 1: DAD1 A, Sig=340,50 Ref=500,100

Peak #	RetTime [min]	Type	Width [min]	Area [mAU*s]	Height [mAU]	Area %
1	19.742	BV	0.1212	9231.31445	1180.81738	94.2247
2	20.613	VP	0.1146	565.81049	76.24522	5.7753

Totals : 9797.12494 1257.06260

Results obtained with enhanced integrator!
===
*** End of Report ***

Figure S48: HPLC chromatogram of **21**

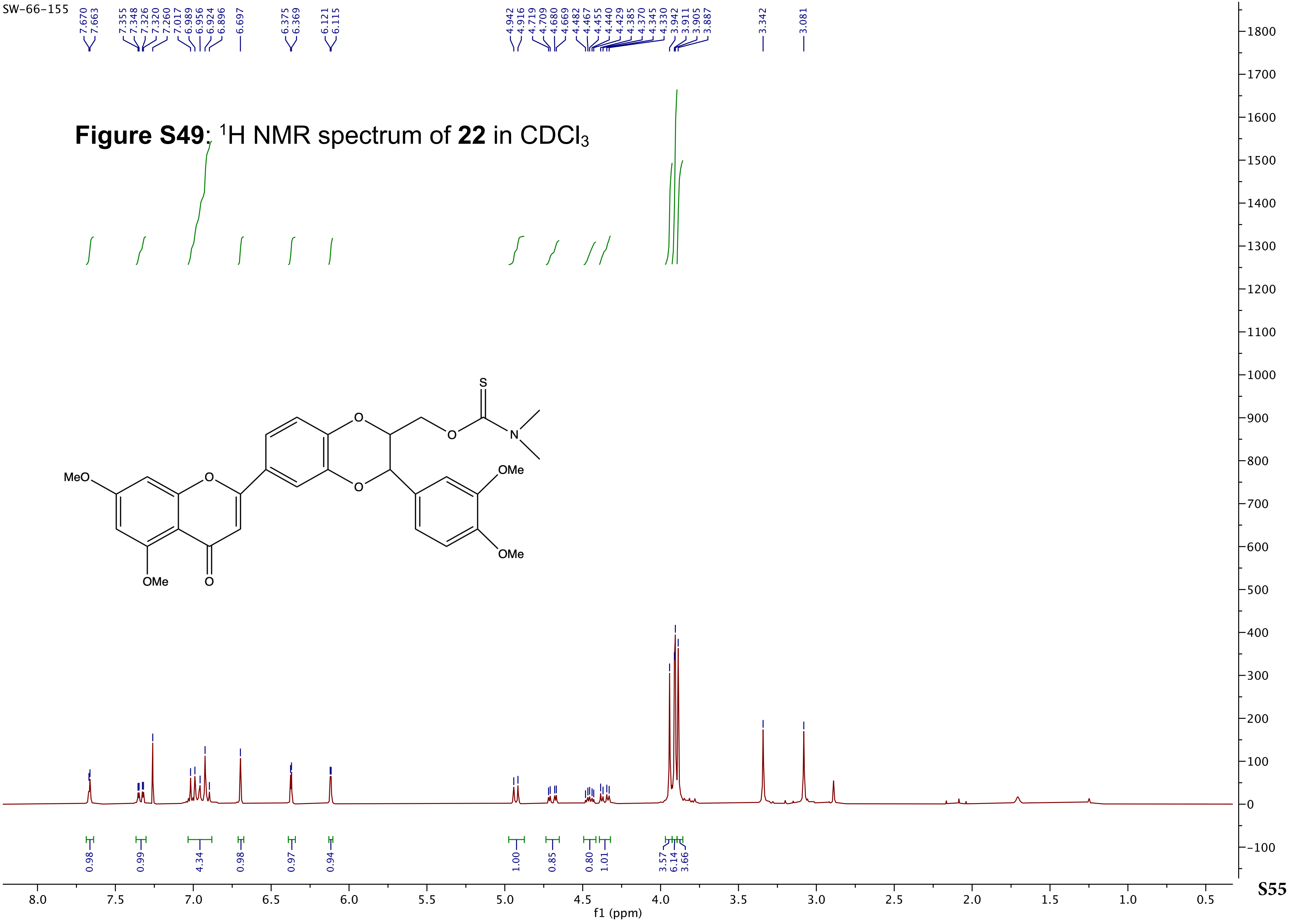

Figure S49: ^{1}H NMR spectrum of **22** in $CDCl_3$

SW-66-155

Figure S50: ^{13}C NMR spectrum of **22** in $CDCl_3$

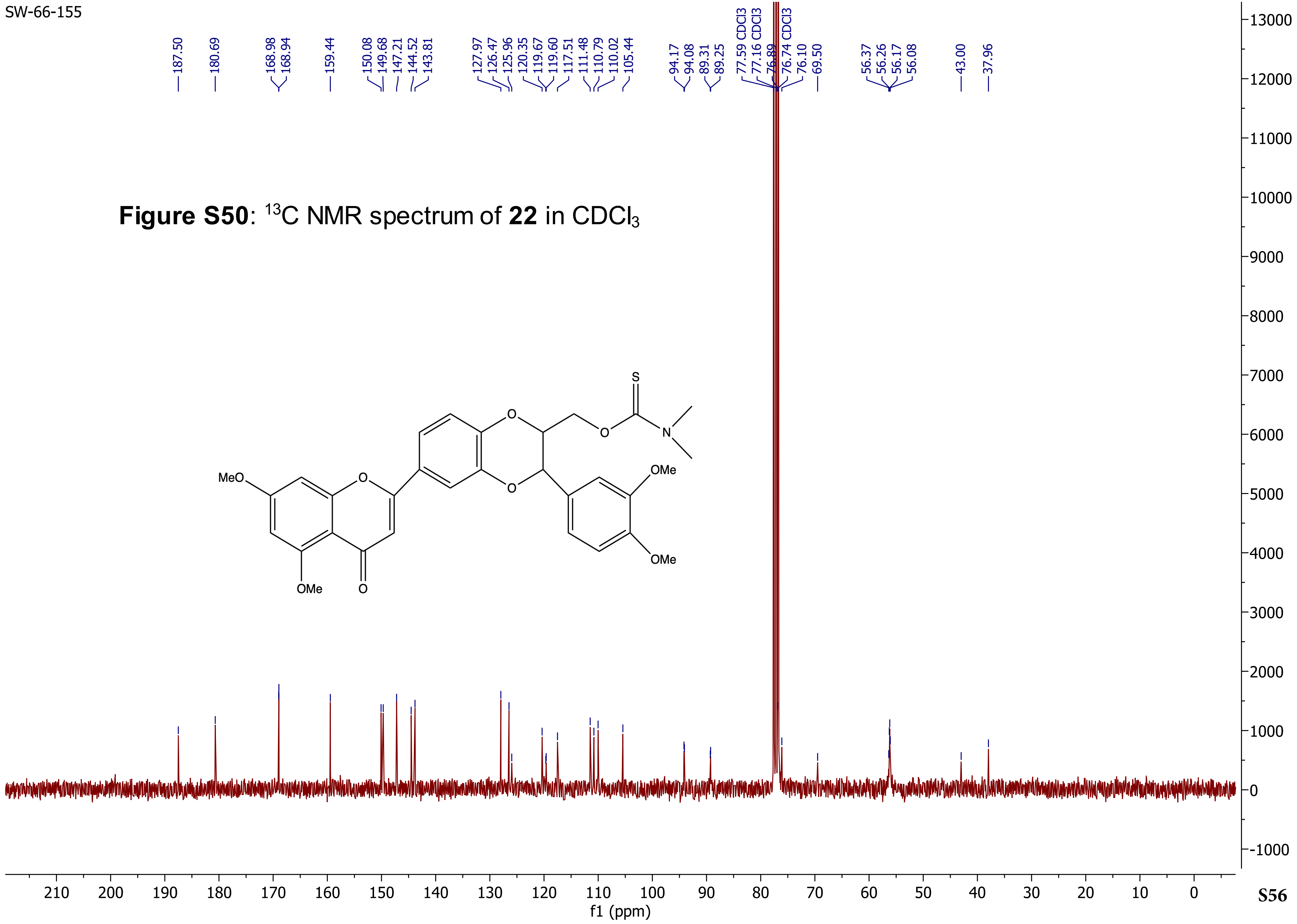

Sample Name	Mol Fomula	MW	M+H	observed	delta	ppm
SW-66-155	C31H31NO9S	593.1720	594.1798	594.1799	0.0001	0.17

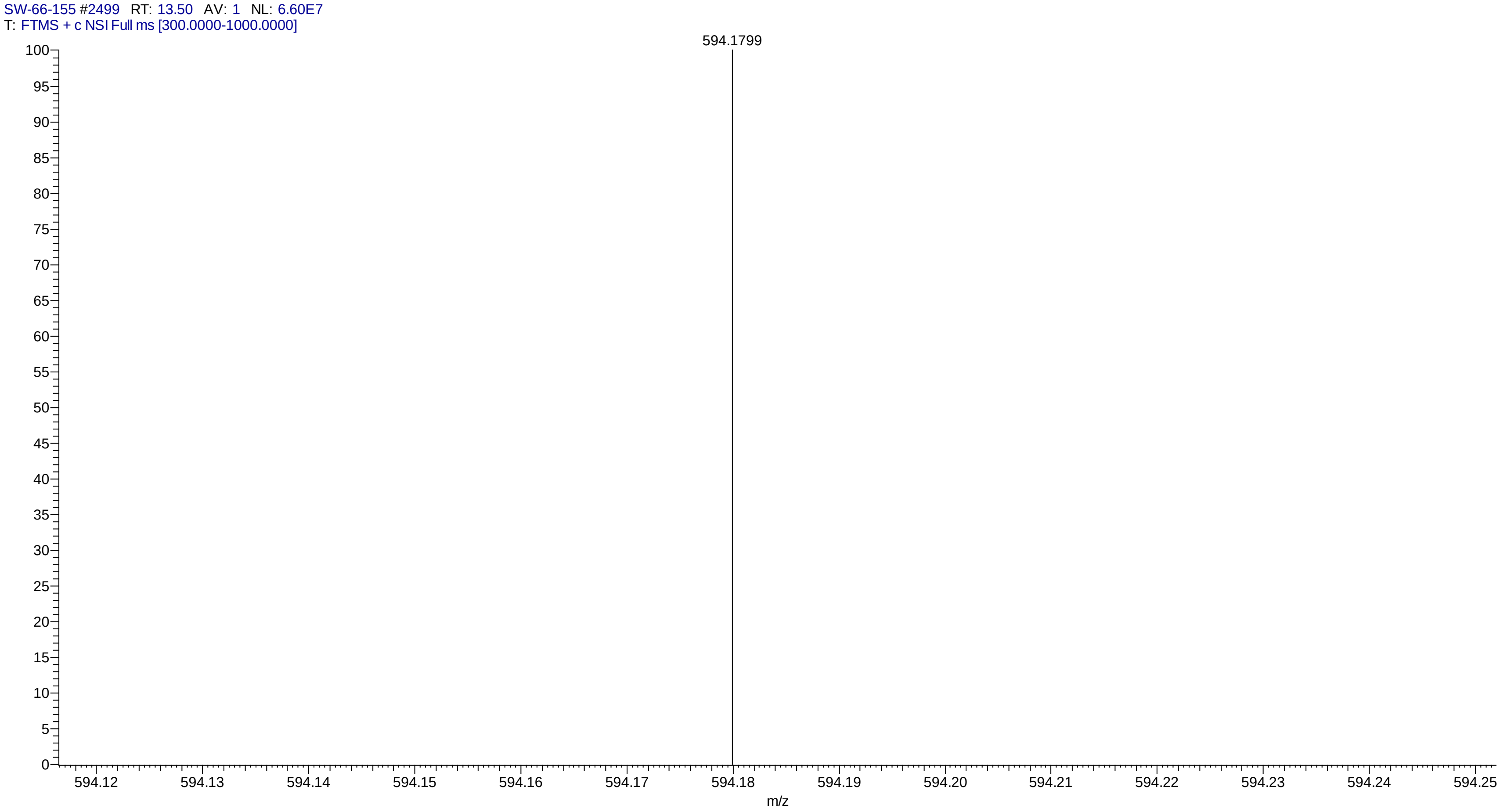

Figure S51: High resolution mass spectrum of **22**

===

Injection Date : 4/29/2022 5:14:59 PM
Sample Name : SW-66-155 Location : Vial 1
Acq. Operator :
Method : C:\HPCHEM\1\METHODS\JNP2015.M
Last changed : 4/29/2022 2:19:03 PM

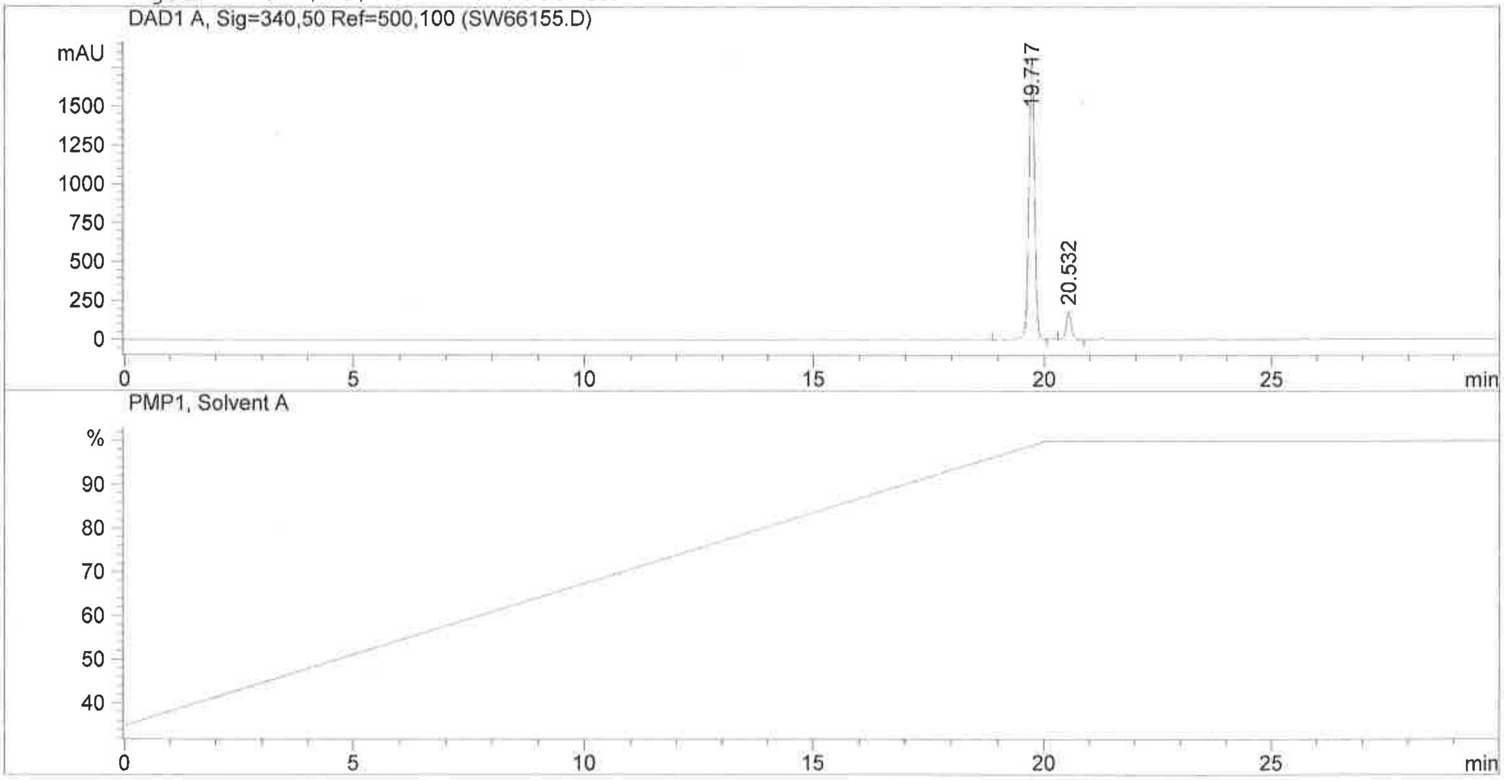

===

Area Percent Report

===

Sorted By : Signal
Multiplier : 1.0000
Dilution : 1.0000

Signal 1: DAD1 A, Sig=340,50 Ref=500,100

Peak #	RetTime [min]	Type	Width [min]	Area [mAU*s]	Height [mAU]	Area %
1	19.717	VV	0.1381	1.59404e4	1821.90735	91.4730
2	20.532	VV	0.1229	1485.94263	182.59920	8.5270

Totals : 1.74263e4 2004.50655

Results obtained with enhanced integrator!

===

*** End of Report ***

Figure S52: HPLC chromatogram of **22**

SW-66-12

Figure S53: ^{1}H NMR spectrum of **23** in $CDCl_3$

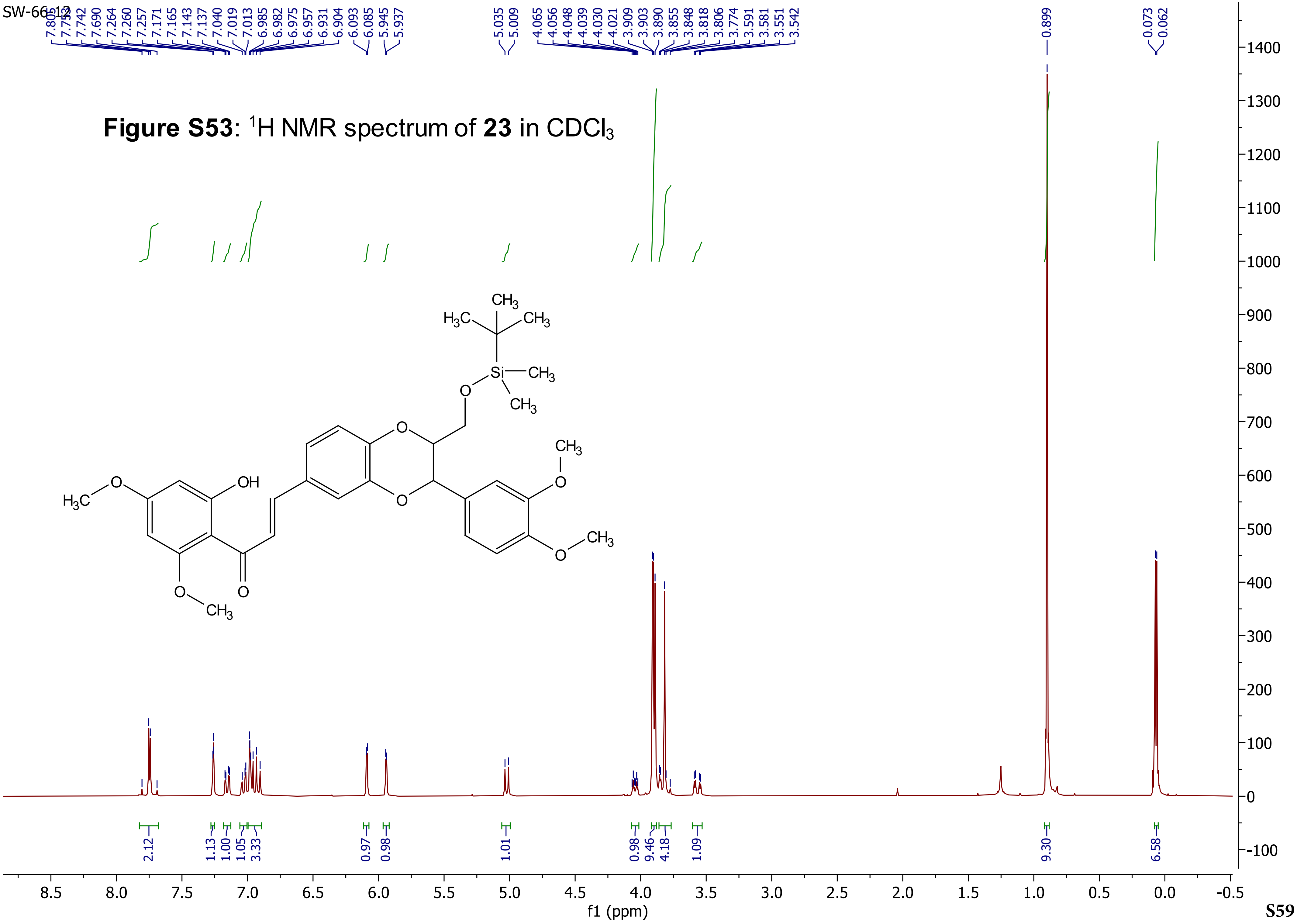

SW-66-12

Figure S54: ^{13}C NMR spectrum of **23** in $CDCl_3$

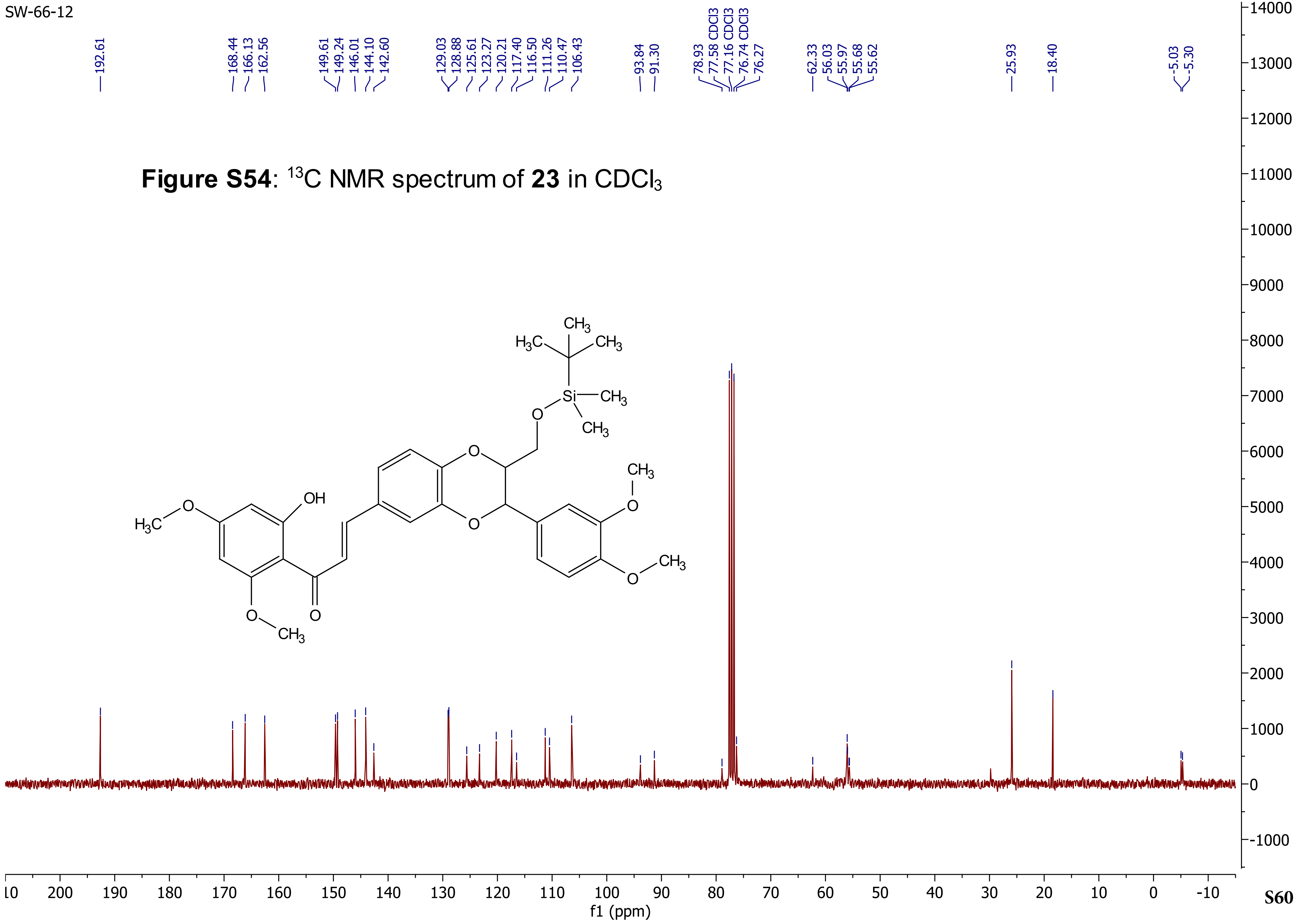

SW-66-110	C34H42O9Si	622.2599	623.2677	**623.2675**	-0.0002	-0.26

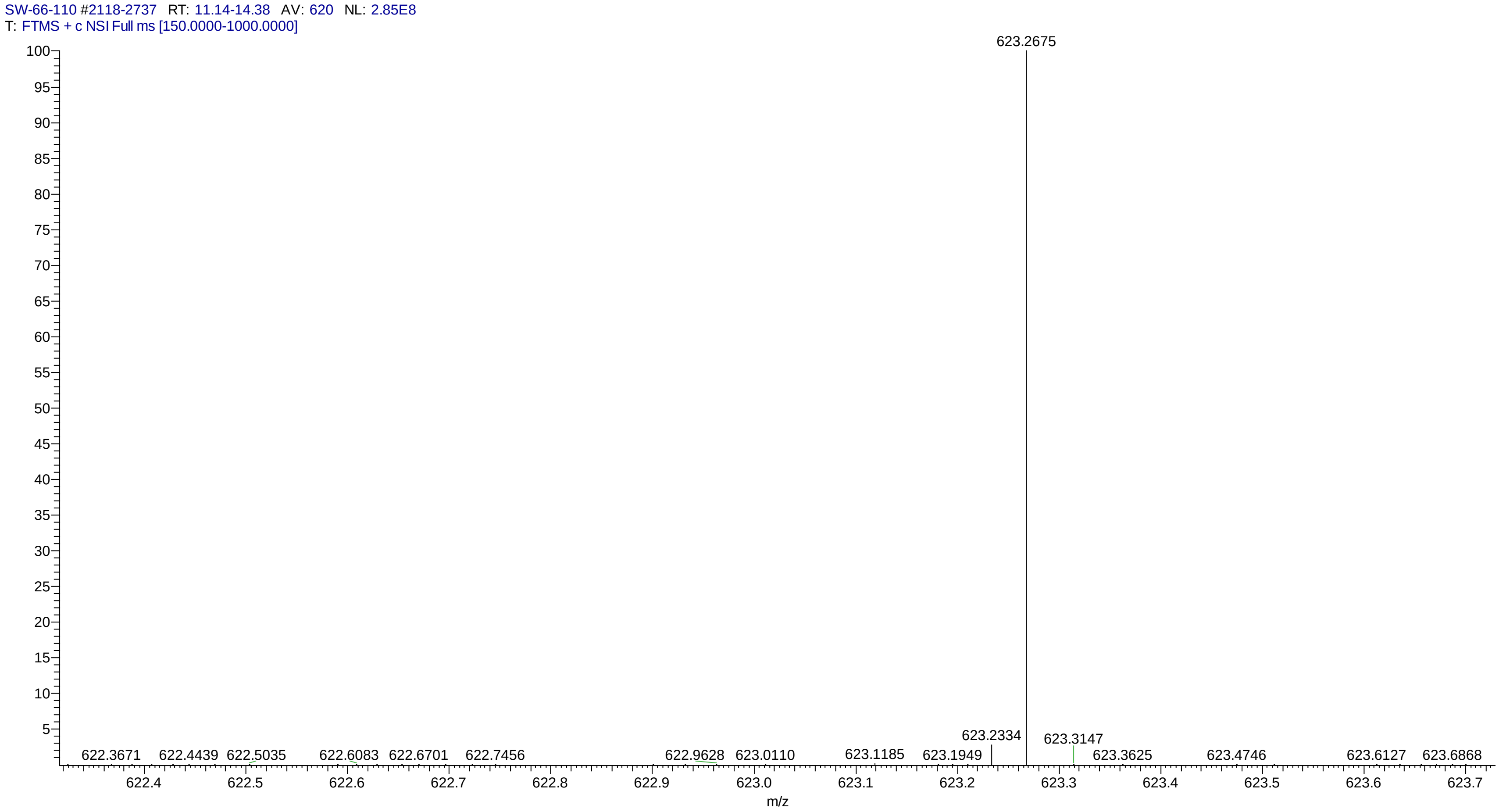

Figure S55: High resolution mass spectrum of **23**

===

Injection Date : 5/3/2022 1:40:30 PM
Sample Name : SW-66-12 Location : Vial 1
Acq. Operator :
Method : C:\HPCHEM\1\METHODS\JNP2015.M
Last changed : 4/30/2022 3:37:52 PM
(modified after loading)

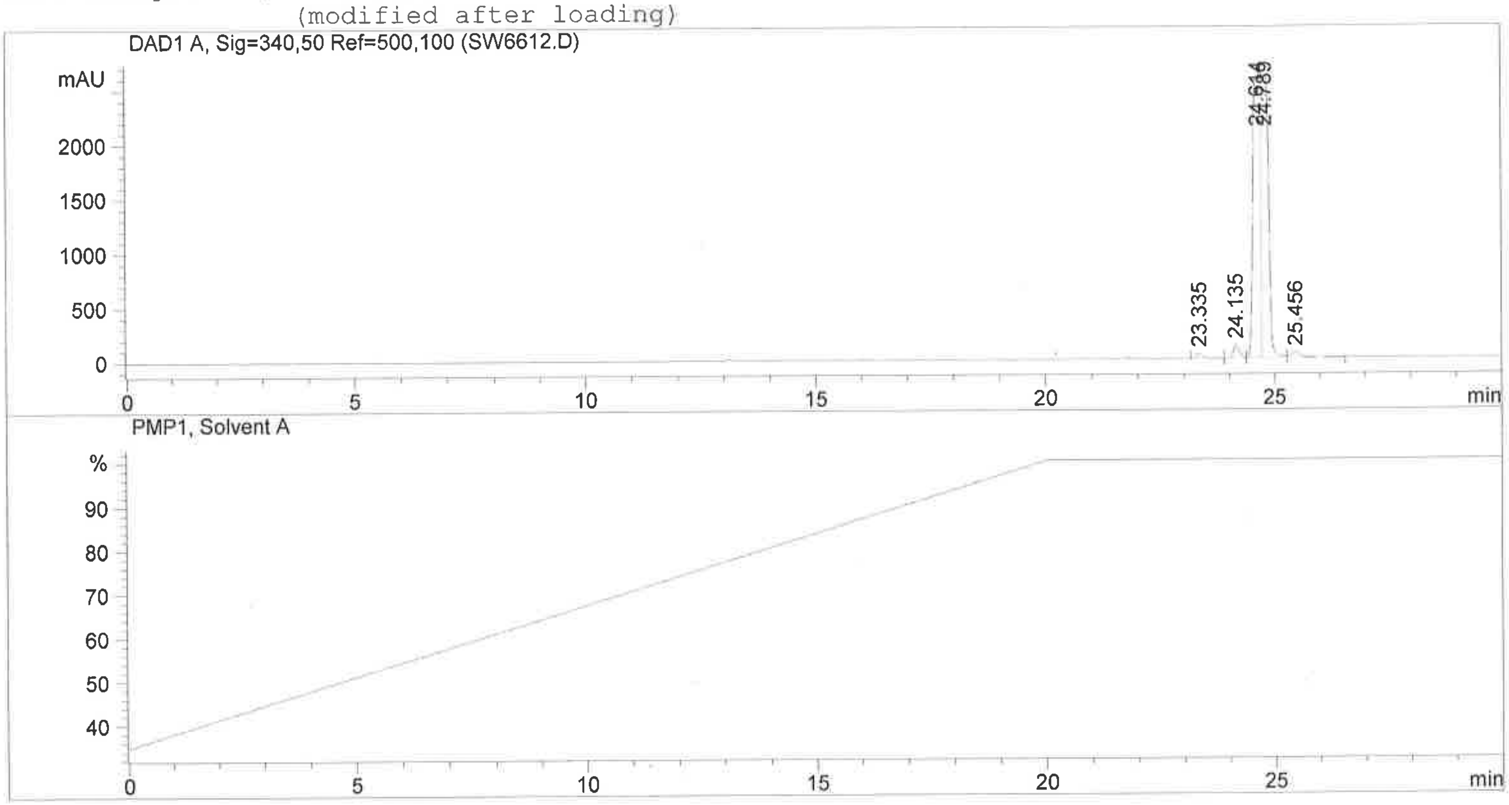

===

Area Percent Report

===

Sorted By : Signal
Multiplier : 1.0000
Dilution : 1.0000

Signal 1: DAD1 A, Sig=340,50 Ref=500,100

Peak #	RetTime [min]	Type	Width [min]	Area [mAU*s]	Height [mAU]	Area %
1	23.335	VP	0.1243	370.50369	46.81130	0.5992
2	24.135	VV	0.1358	1101.45093	123.75067	1.7812
3	24.614	VV	0.1830	3.09482e4	2597.71899	50.0486
4	24.789	VV	0.1606	2.86429e4	2598.86646	46.3206
5	25.456	VB	0.2137	773.15863	51.91675	1.2503

Totals : 6.18362e4 5419.06417

Results obtained with enhanced integrator!

===

*** End of Report ***

Figure S56: HPLC chromatogram of **23**

SW-66-14

Figure S57: ^{1}H NMR spectrum of **24** in $CDCl_3$

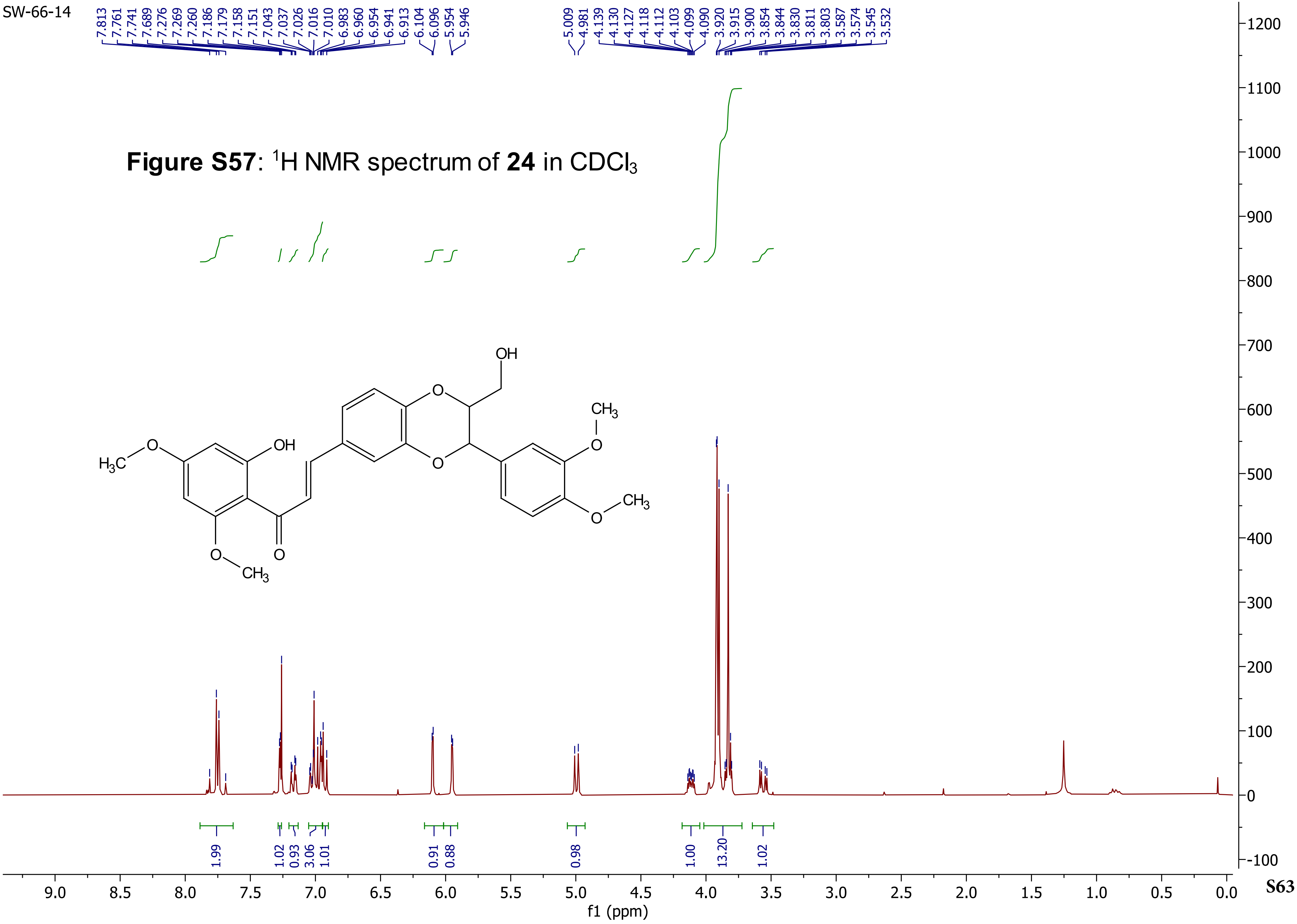

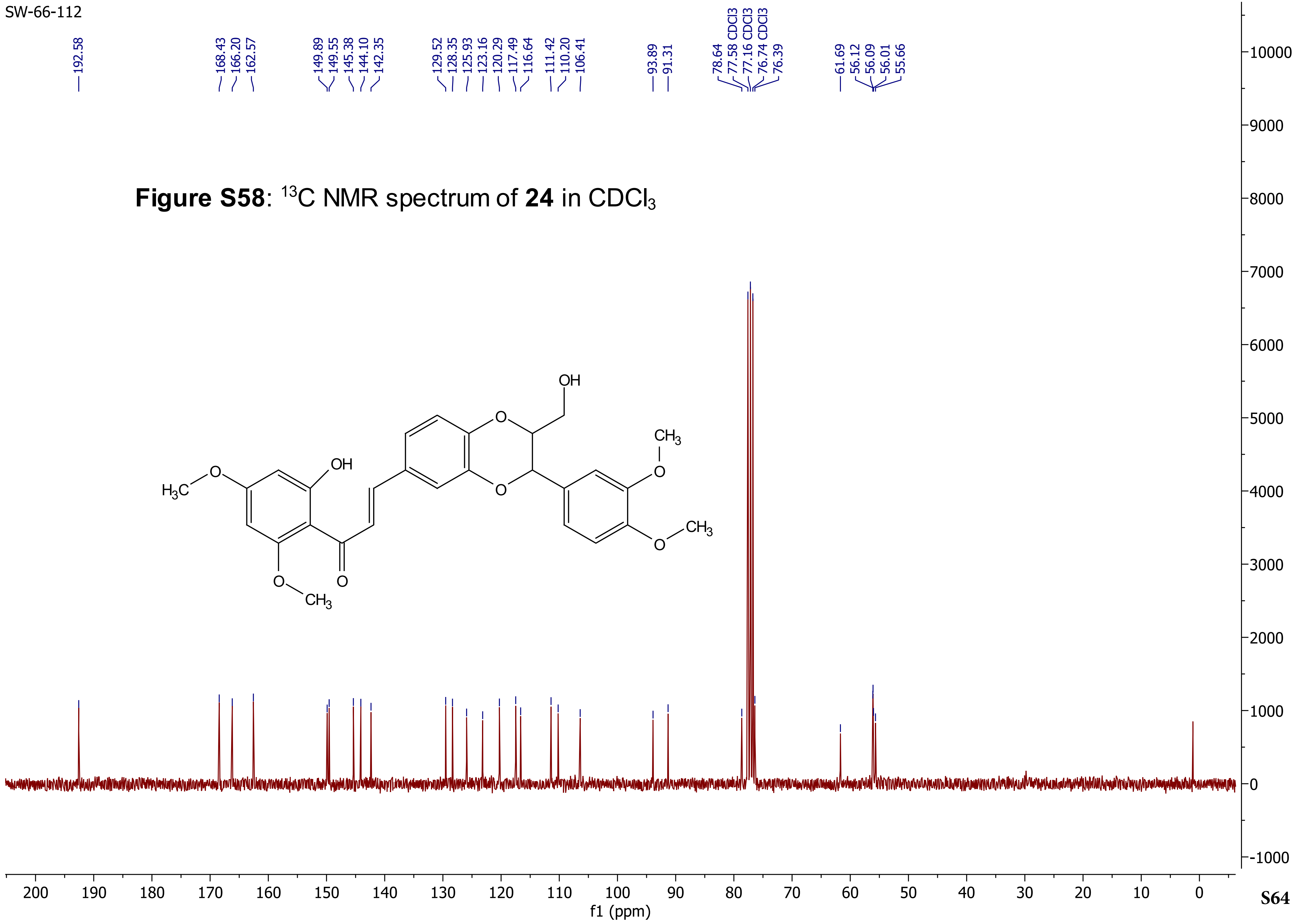

Figure S58: ^{13}C NMR spectrum of **24** in $CDCl_3$

SW-66-14	C28H28O9	508.1734	509.1812	**509.1806**	-0.0006	-1.14

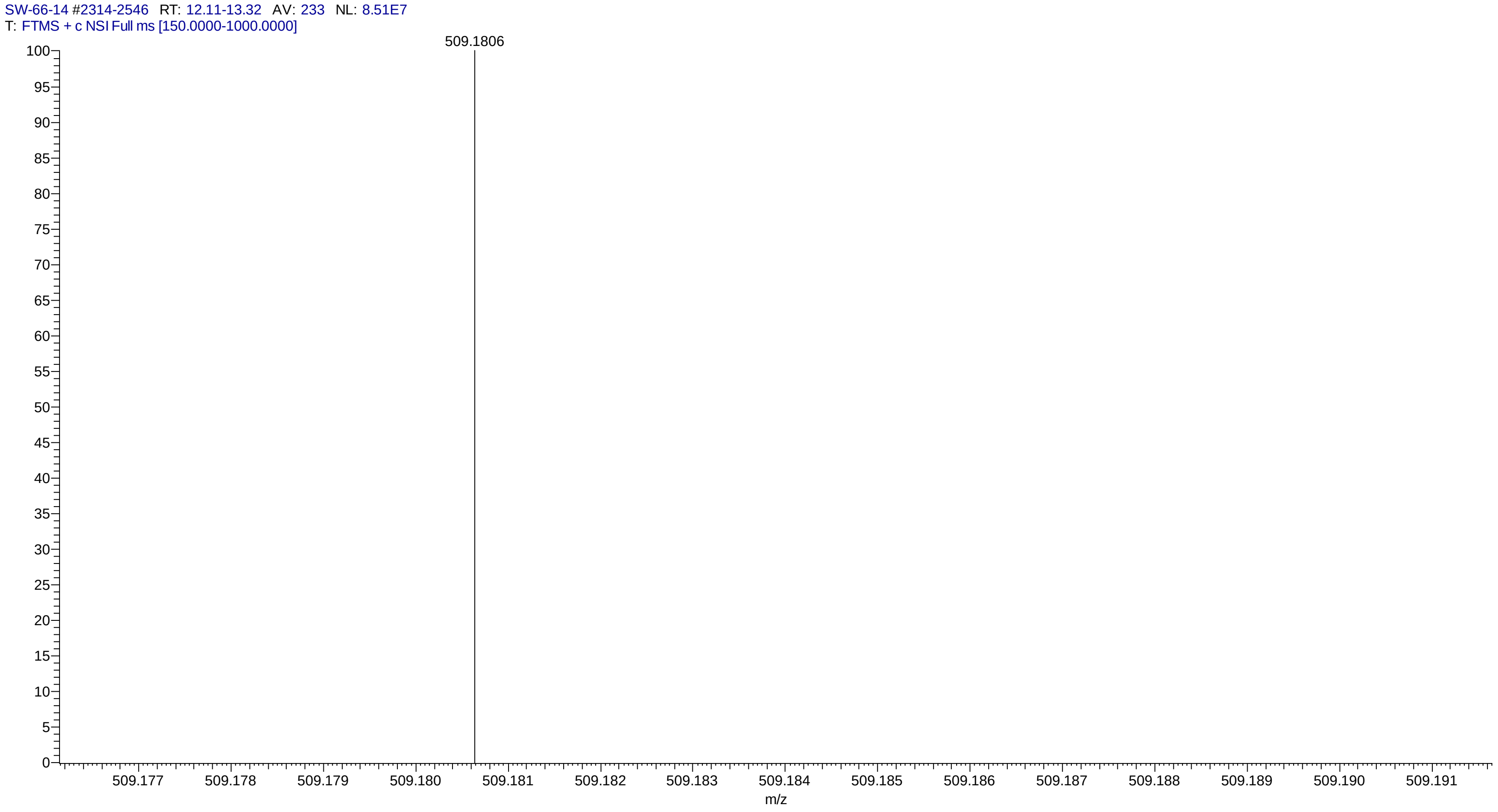

Figure S59: High resolution mass spectrum of **24**

Data File C:\HPCHEM\1\DATA\DEFAULT.D

Sample Name: SW-66-14

===

Injection Date : 4/25/2022 4:10:06 PM
Sample Name : SW-66-14
Location : Vial 1
Acq. Operator :
Method : C:\HPCHEM\1\METHODS\JNP2015.M
Last changed : 4/24/2022 3:10:54 PM
(modified after loading)

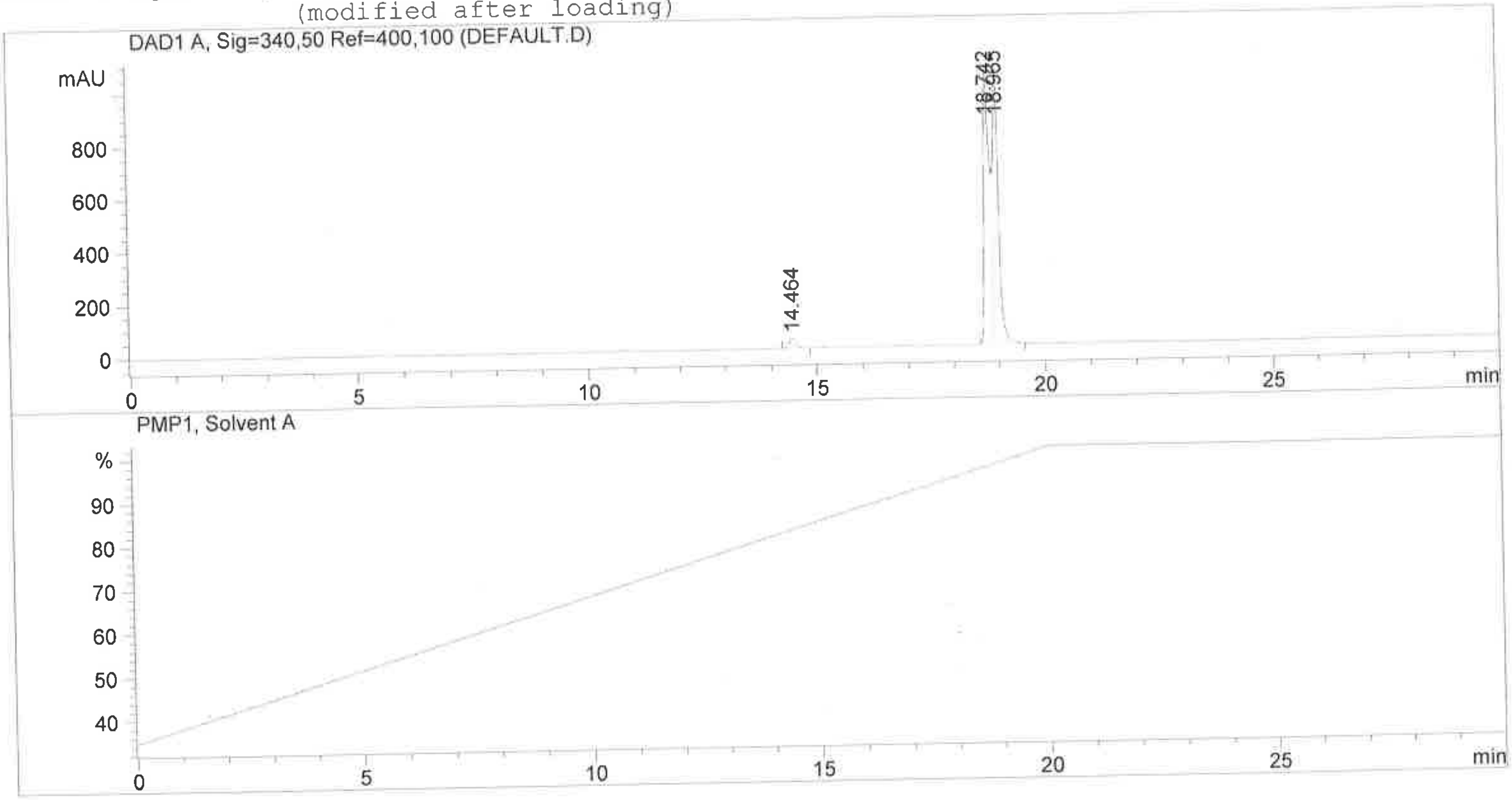

===

Area Percent Report

===

Sorted By : Signal
Multiplier : 1.0000
Dilution : 1.0000

Signal 1: DAD1 A, Sig=340,50 Ref=400,100

Peak #	RetTime [min]	Type	Width [min]	Area [mAU*s]	Height [mAU]	Area %
1	14.464	BV	0.1460	362.14001	37.04652	1.9489
2	18.742	BV	0.1224	9316.91309	1061.96533	50.1391
3	18.965	VB	0.1239	8903.08008	1040.01978	47.9120

Totals : 1.85821e4 2139.03162

Results obtained with enhanced integrator!

===

*** End of Report ***

Figure S60: HPLC chromatogram of **24**

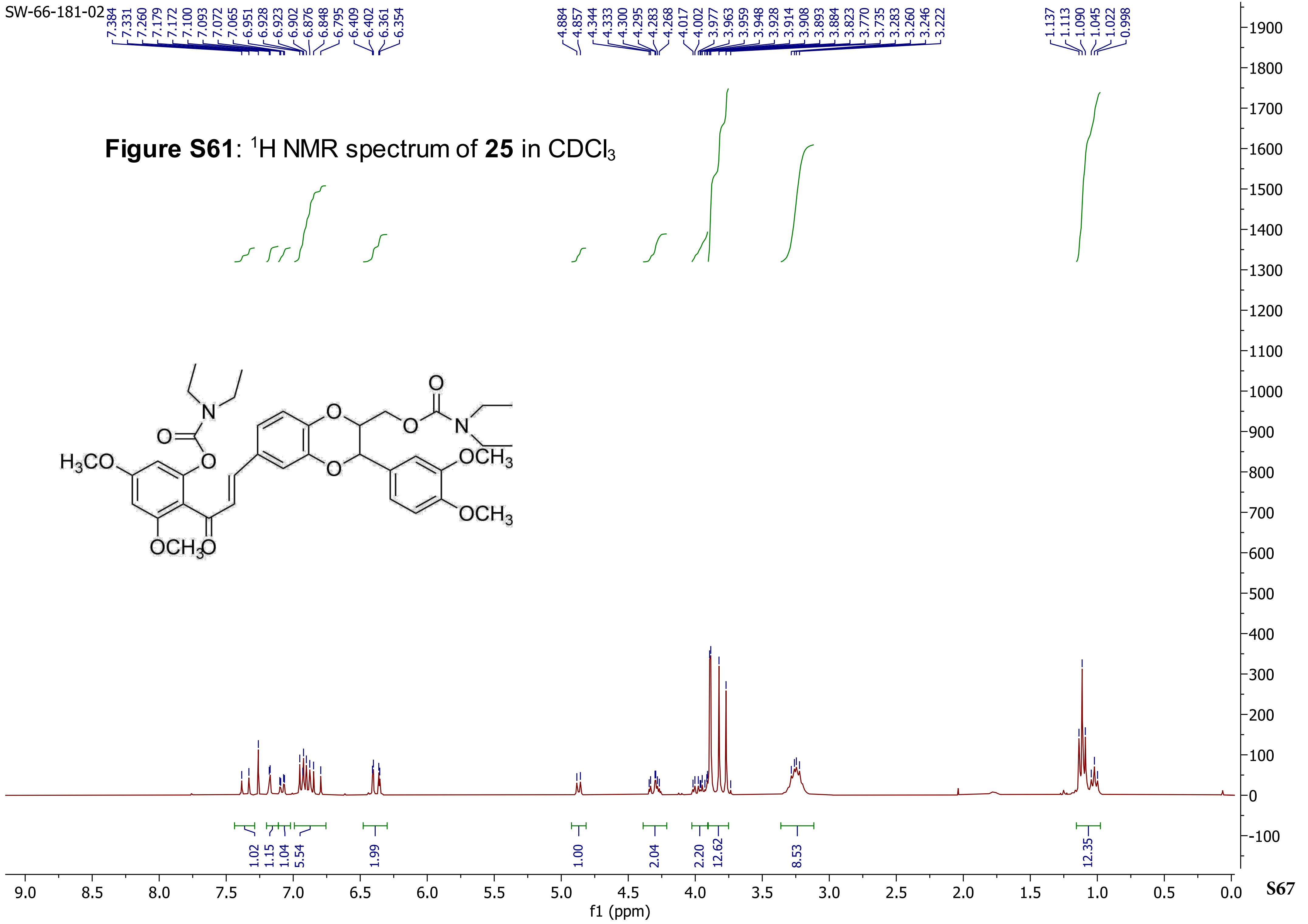

Figure S61: ^{1}H NMR spectrum of **25** in $CDCl_3$

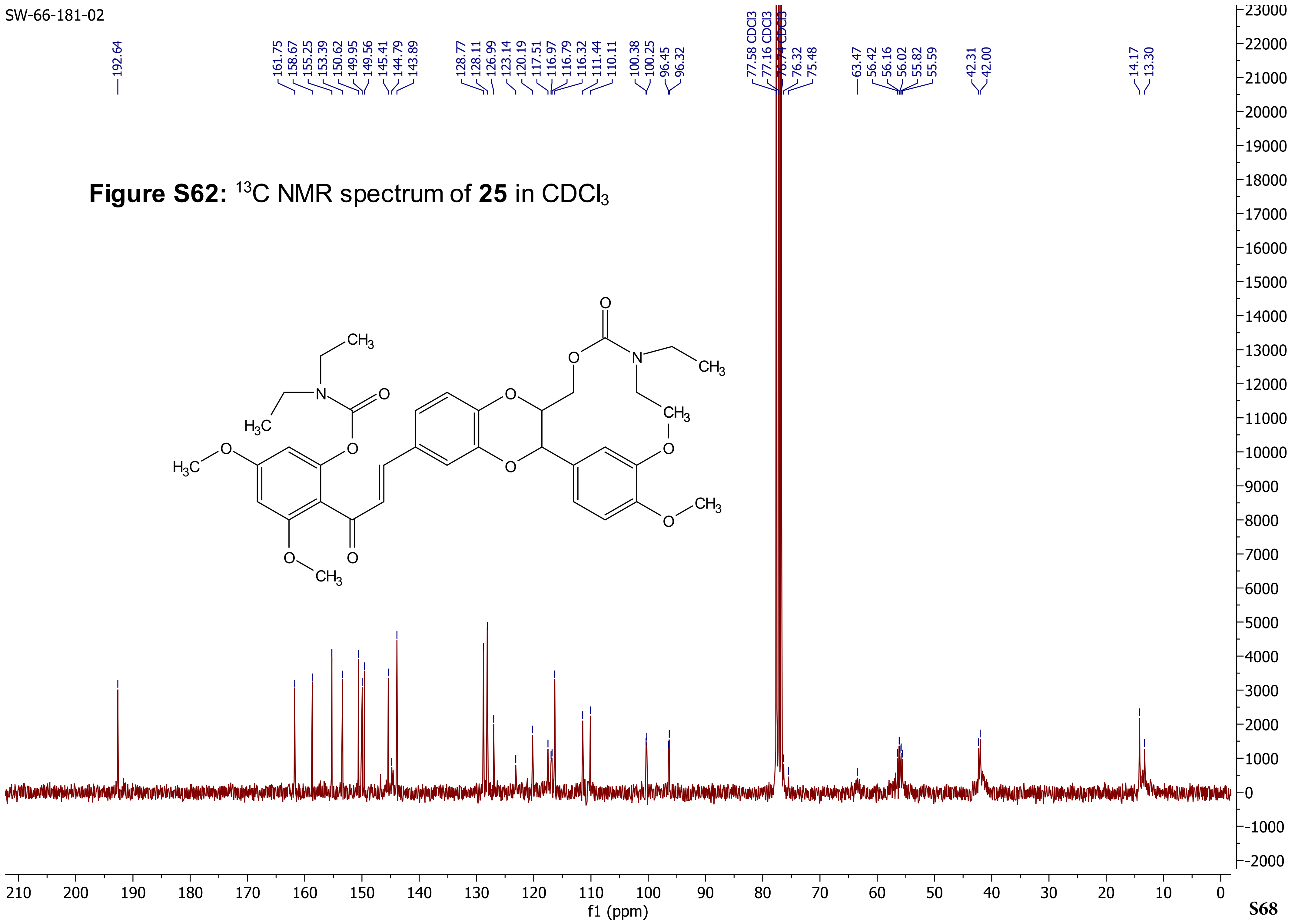

Figure S62: ^{13}C NMR spectrum of **25** in $CDCl_3$

Sample Name	Mol Fomula	MW	M+H	observed	delta	ppm
SW-66-181-02	C38H46N2O11	706.3102	707.3180	707.3179	-0.0001	-0.17

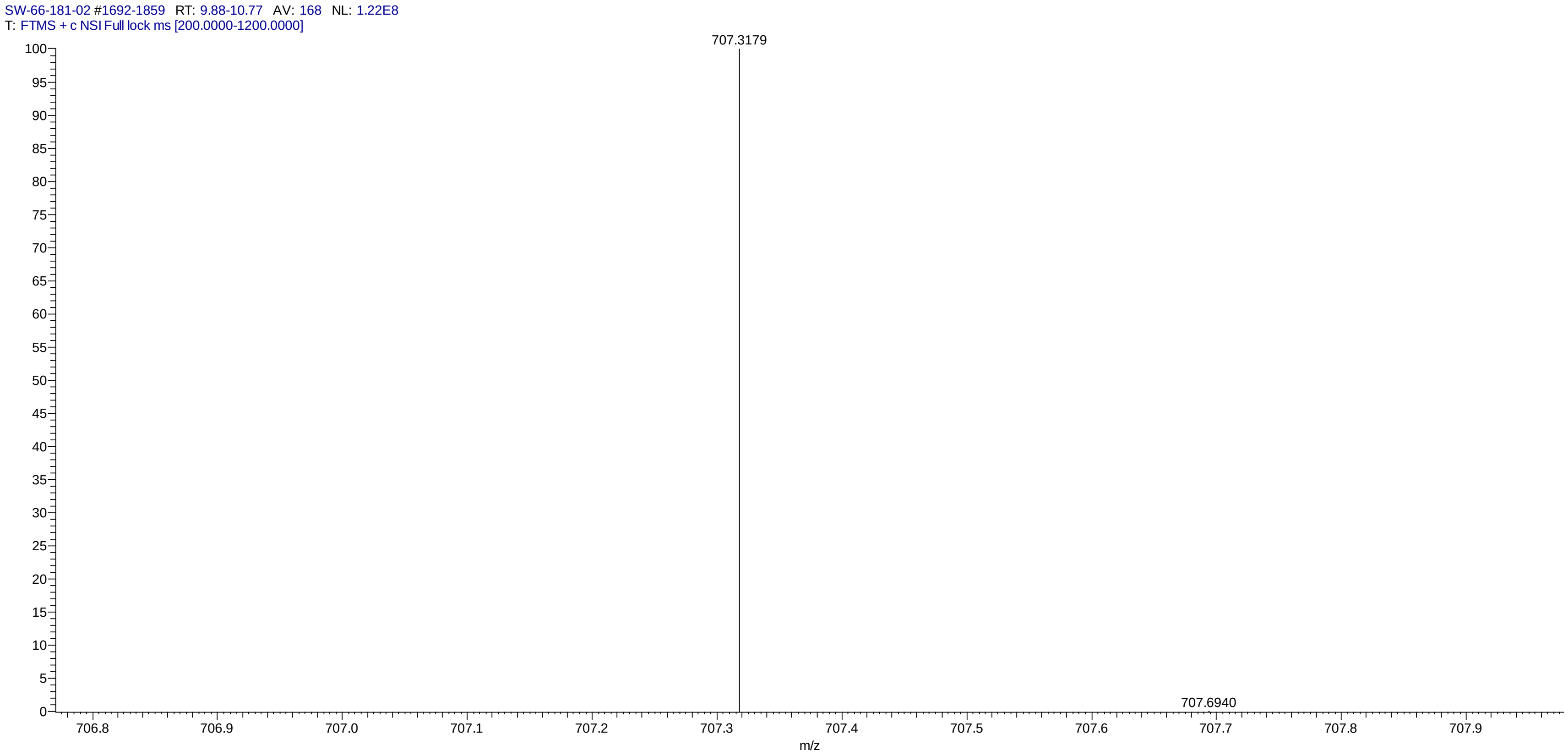

Figure S63: High resolution mass spectrum of **25**

===

Injection Date : 4/24/2022 5:28:43 PM
Sample Name : SW-85-181-02 Location : Vial 1
Acq. Operator :
Method : C:\HPCHEM\1\METHODS\JNP2015.M
Last changed : 4/24/2022 3:10:54 PM
(modified after loading)

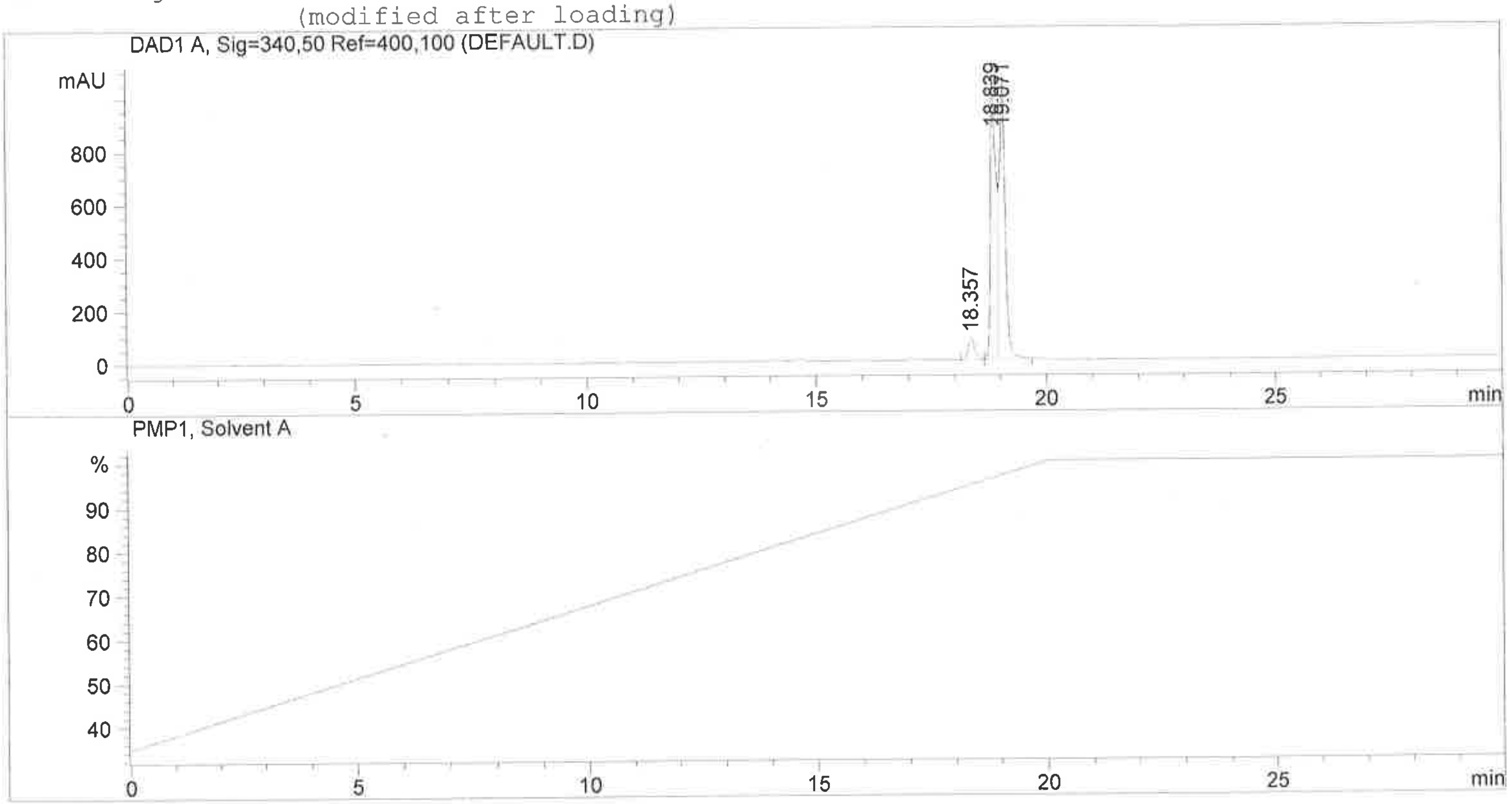

===

Area Percent Report

===

Sorted By : Signal
Multiplier : 1.0000
Dilution : 1.0000

Signal 1: DAD1 A, Sig=340,50 Ref=400,100

Peak #	RetTime [min]	Type	Width [min]	Area [mAU*s]	Height [mAU]	Area %
1	18.357	PP	0.1516	805.34631	82.72363	4.1372
2	18.839	VV	0.1201	9115.85254	1062.03345	46.8296
3	19.071	VB	0.1265	9544.79883	1065.28052	49.0332

Totals : 1.94660e4 2210.03760

Results obtained with enhanced integrator!

===

*** End of Report ***

Figure S64: HPLC chromatogram of **25**

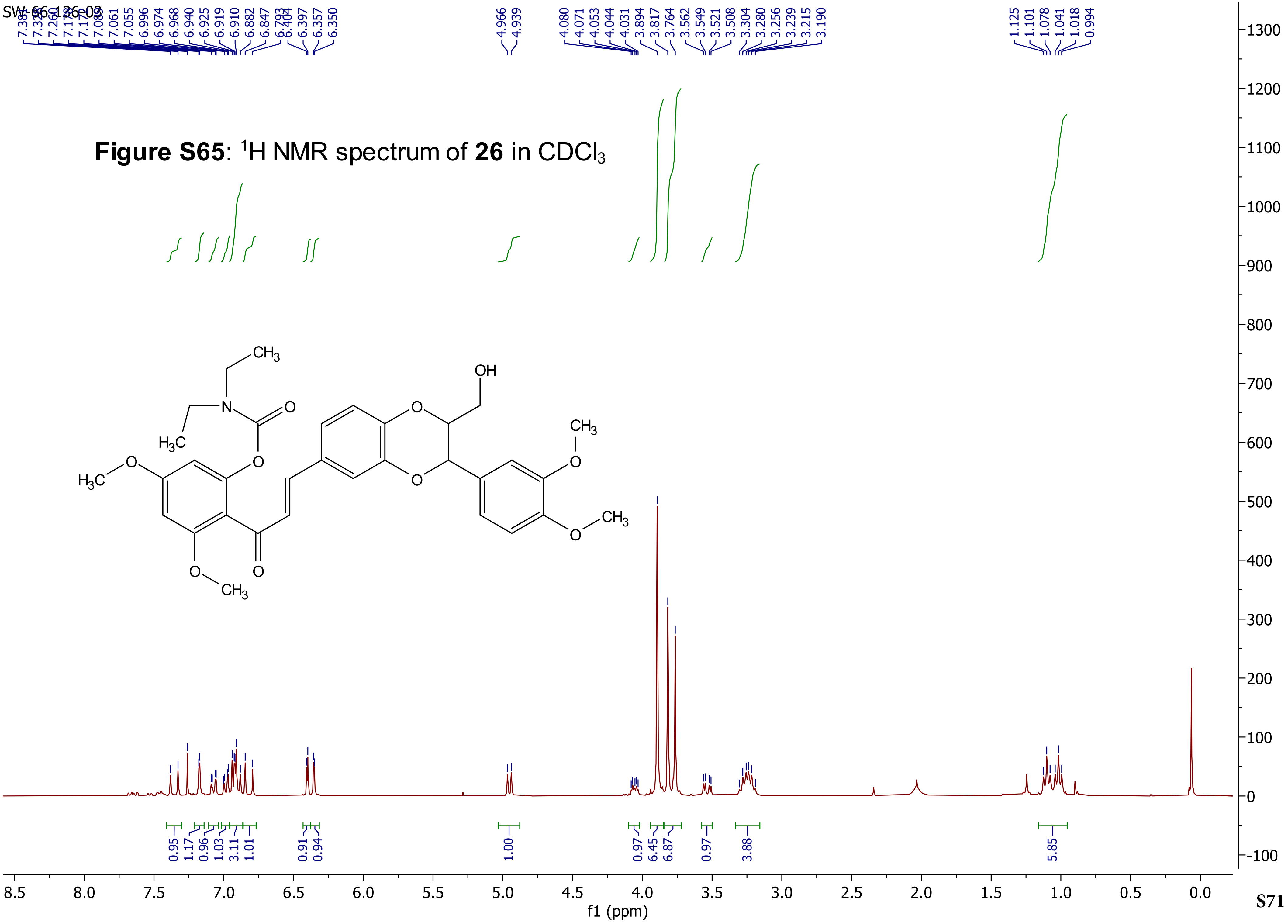

Figure S65: ^{1}H NMR spectrum of **26** in $CDCl_3$

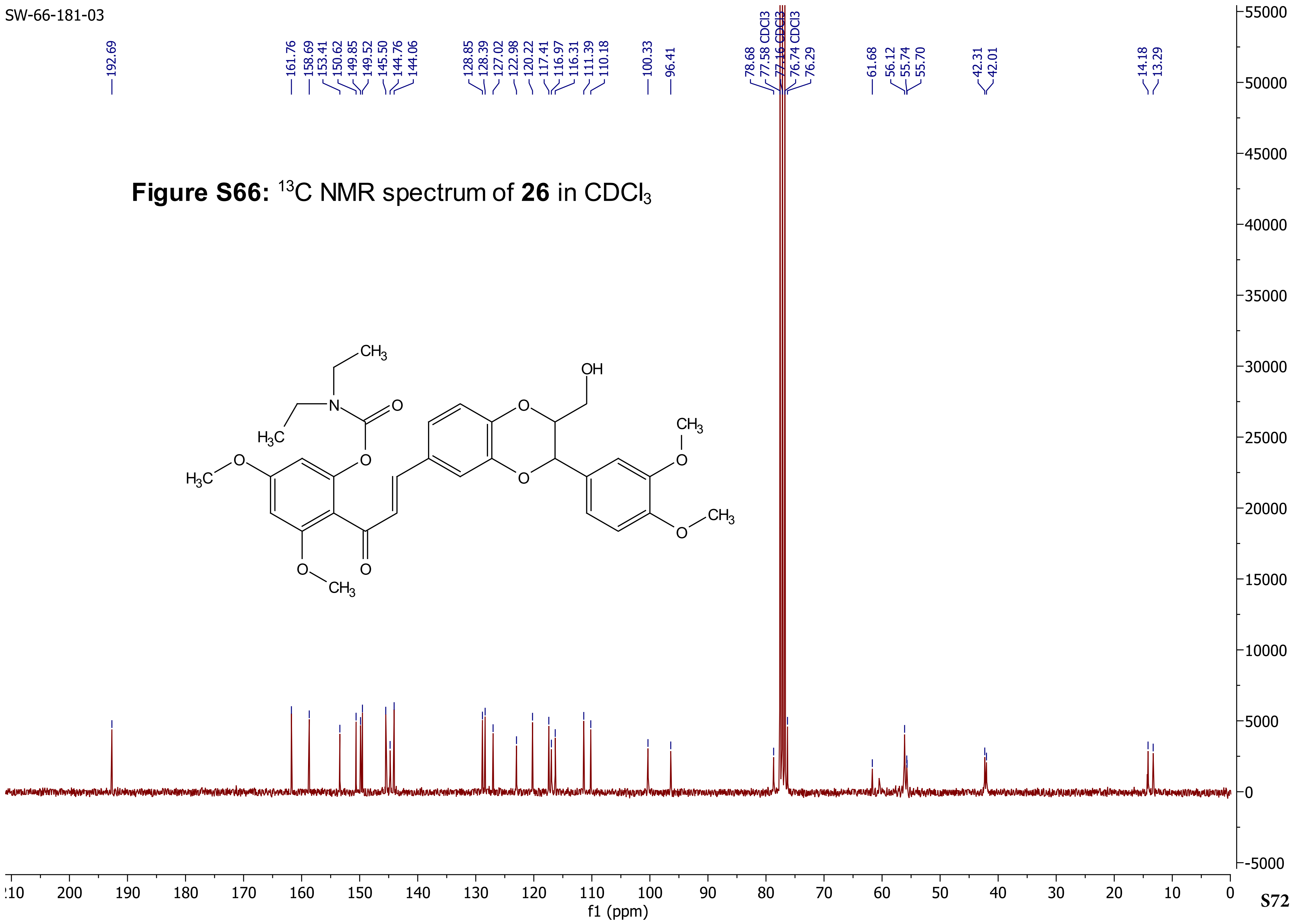

Figure S66: ^{13}C NMR spectrum of **26** in $CDCl_3$

SW-66-136-03	C33H37NO10	607.2418	608.2496	**608.2493**	-0.0003	-0.48

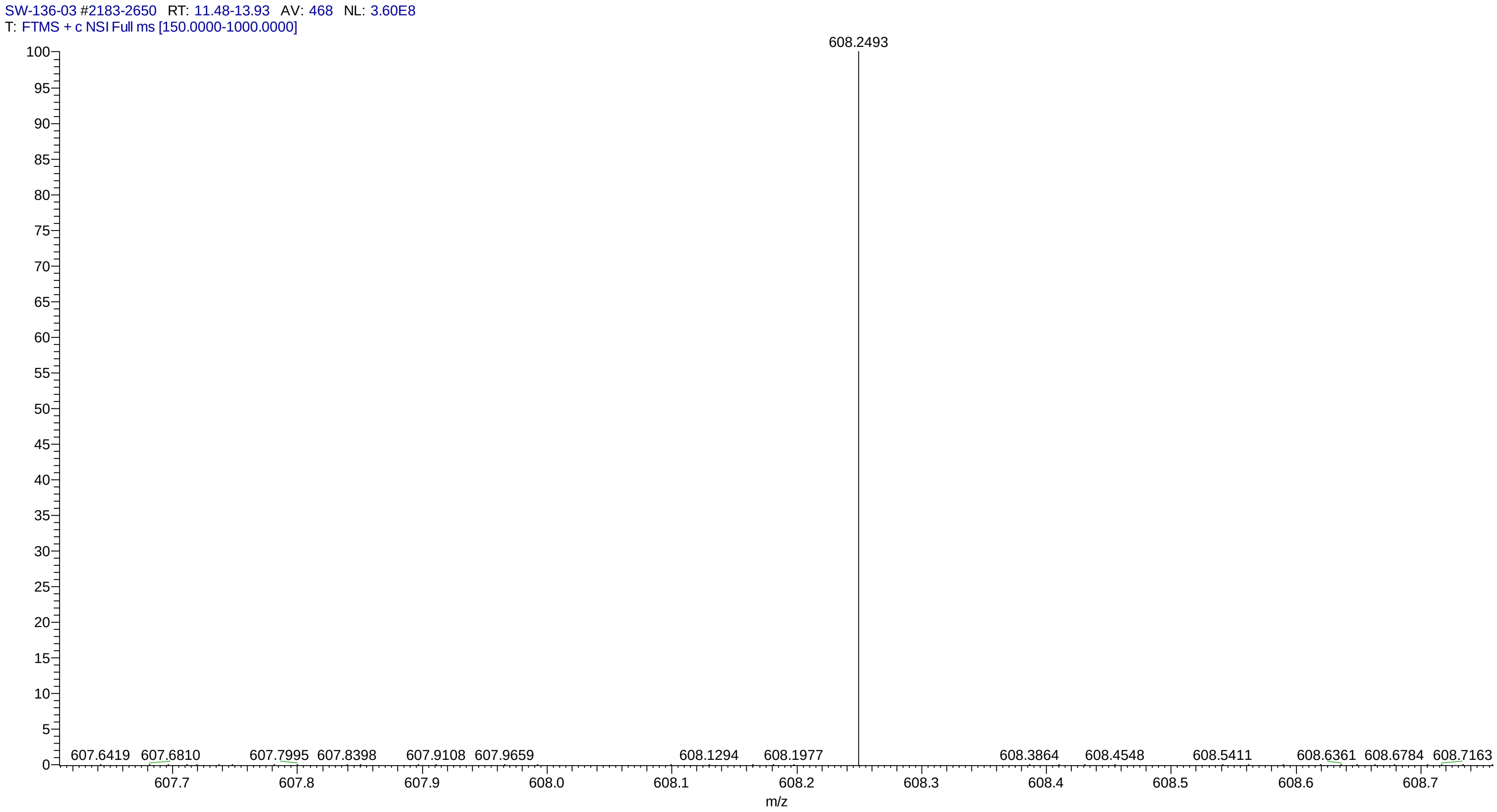

Figure S67: High resolution mass spectrum of **26**

```
=====================================================================
Injection Date   : 2/10/2023 1:47:32 PM
Sample Name      : SW-66-181-03                    Location : Vial 1
Acq. Operator    :
Method           : C:\HPCHEM\1\METHODS\JNP2015.M
Last changed     : 2/9/2023 3:27:35 PM
```

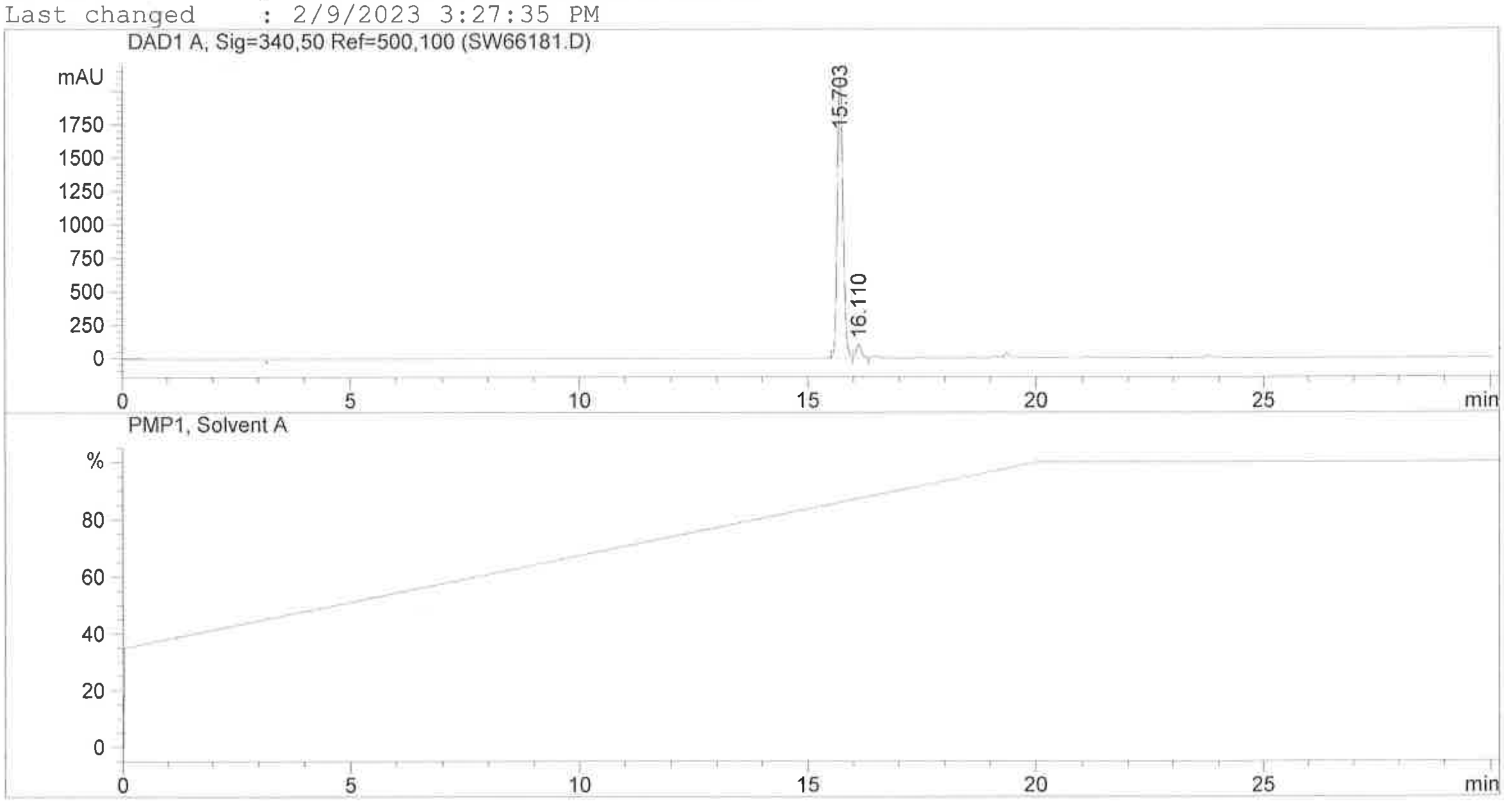


```
=====================================================================
                        Area Percent Report
=====================================================================

Sorted By             :      Signal
Multiplier            :      1.0000
Dilution              :      1.0000


Signal 1: DAD1 A, Sig=340,50 Ref=500,100

Peak RetTime Type  Width     Area       Height      Area
  #   [min]        [min]   [mAU*s]      [mAU]        %
----|-------|----|-------|----------|----------|--------|
   1  15.703 BV    0.1364 1.82694e4  2081.19800  95.5565
   2  16.110 VV    0.1265  849.54248  102.65541   4.4435

Totals :                  1.91190e4  2183.85341

 Results obtained with enhanced integrator!
=====================================================================
                         *** End of Report ***
```

Figure S68: HPLC chromatogram of **26**

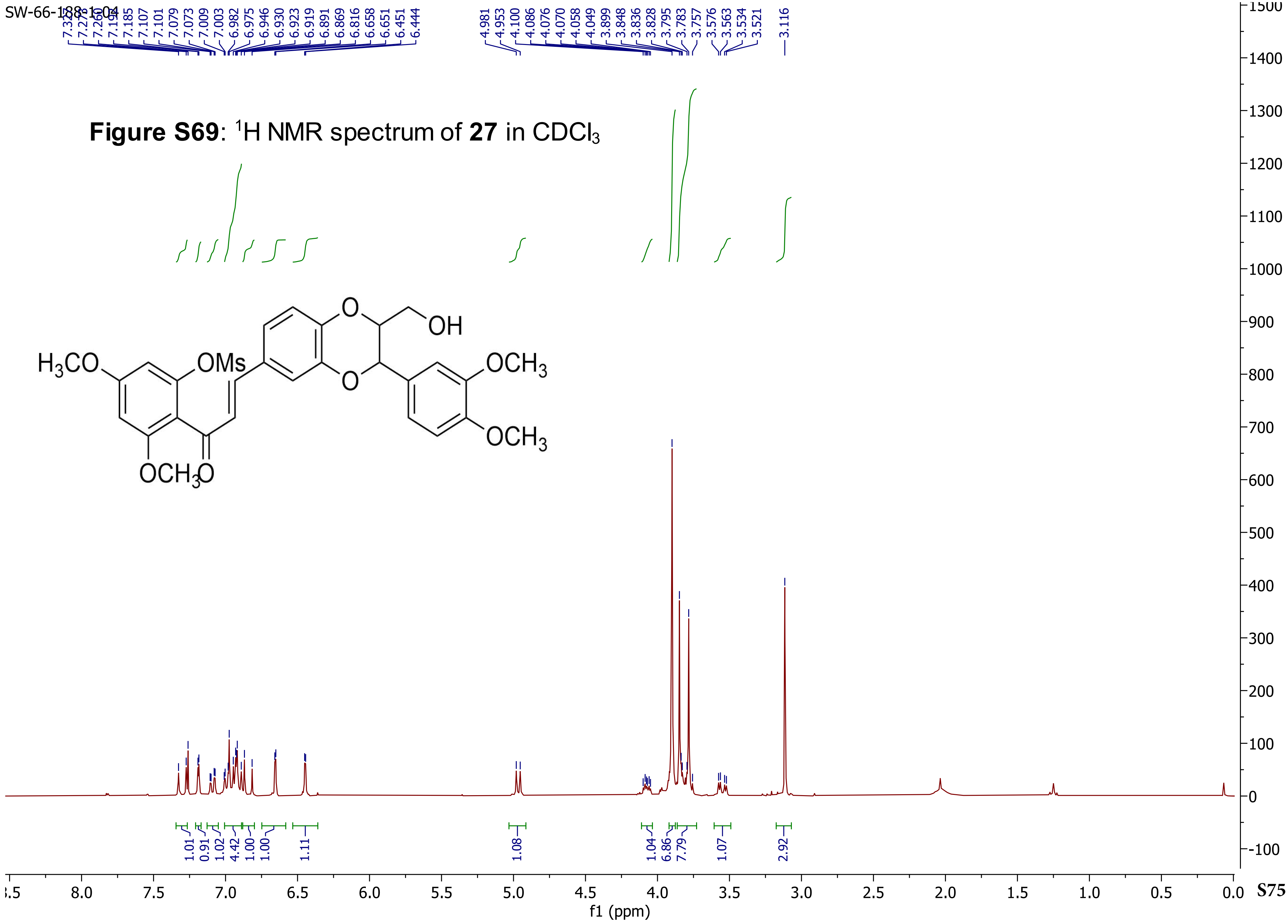

Figure S69: ^1H NMR spectrum of **27** in $CDCl_3$

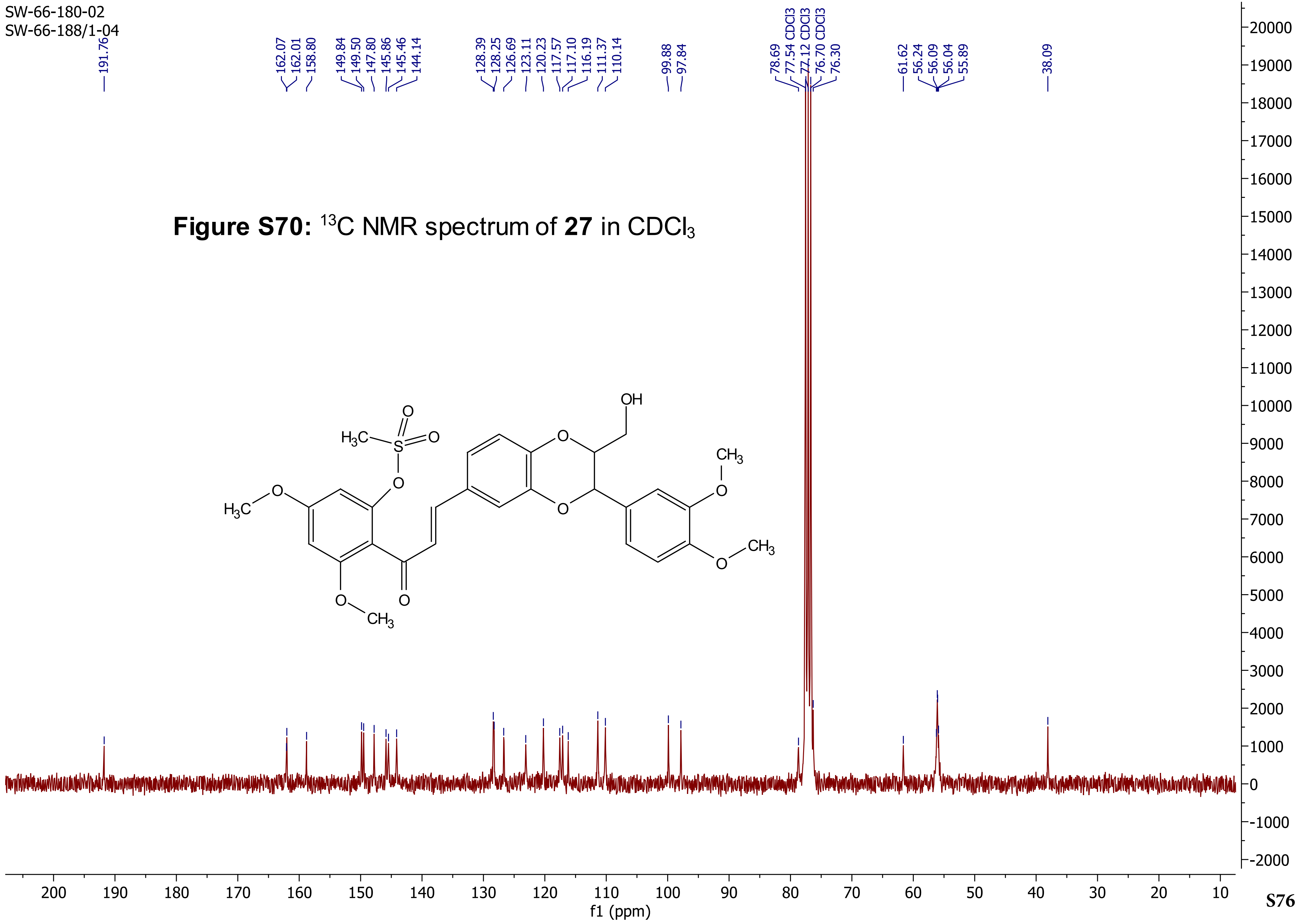

Figure S70: ^{13}C NMR spectrum of **27** in $CDCl_3$

Sample Name	Mol Fomula	MW	M+H	observed	delta	ppm
SW-66-180-02	C29H30O11S	586.1509	587.1587	587.1587	0.0000	0.00

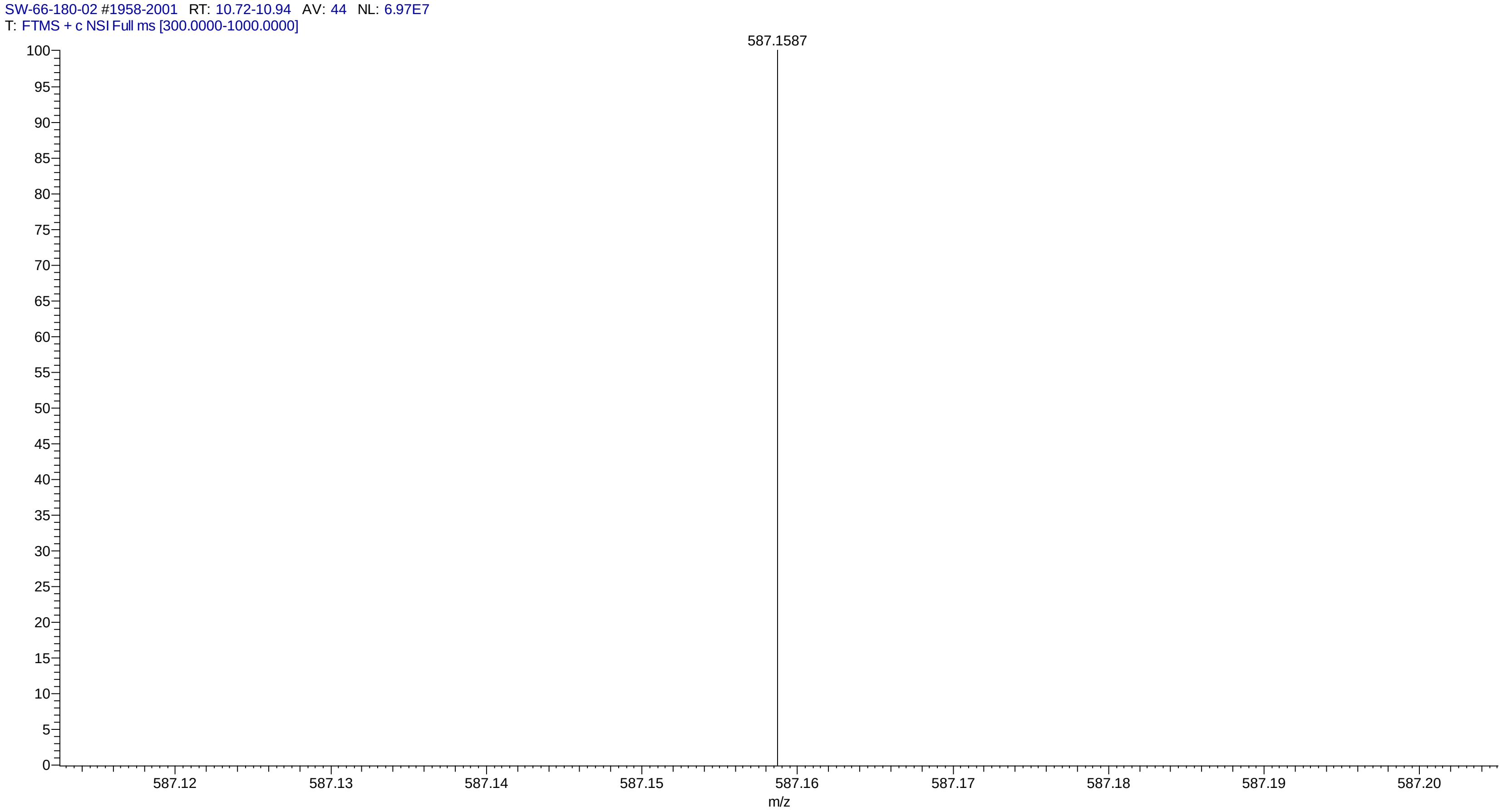

Figure S71: High resolution mass spectrum of **27**

Injection Date : 4/24/2022 1:16:42 PM
Sample Name : SW-85-180-02 Location : Vial 1
Acq. Operator :
Method : C:\HPCHEM\1\METHODS\JNP2015.M
Last changed : 4/22/2022 5:19:36 PM
(modified after loading)

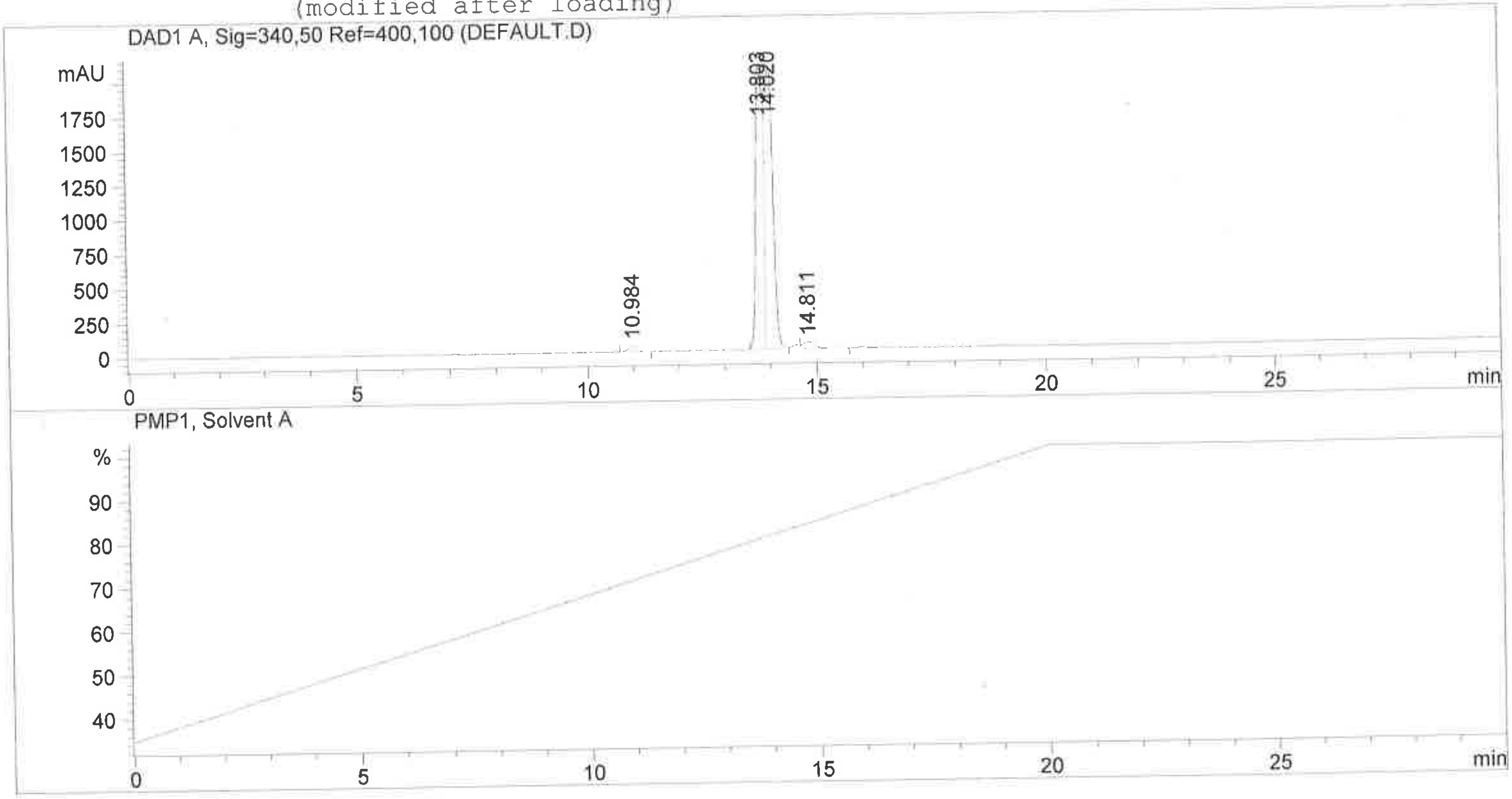

Area Percent Report

Sorted By : Signal
Multiplier : 1.0000
Dilution : 1.0000

Signal 1: DAD1 A, Sig=340,50 Ref=400,100

Peak #	RetTime [min]	Type	Width [min]	Area [mAU*s]	Height [mAU]	Area %
1	10.984	BP	0.1720	521.16962	44.70438	1.0442
2	13.803	BV	0.1639	2.41247e4	2071.67554	48.3376
3	14.020	VV	0.1736	2.43214e4	2061.74927	48.7318
4	14.811	VV	0.2767	941.45032	51.63195	1.8863

Totals : 4.99087e4 4229.76114

Results obtained with enhanced integrator!

*** End of Report ***

Figure S72: HPLC chromatogram of **27**

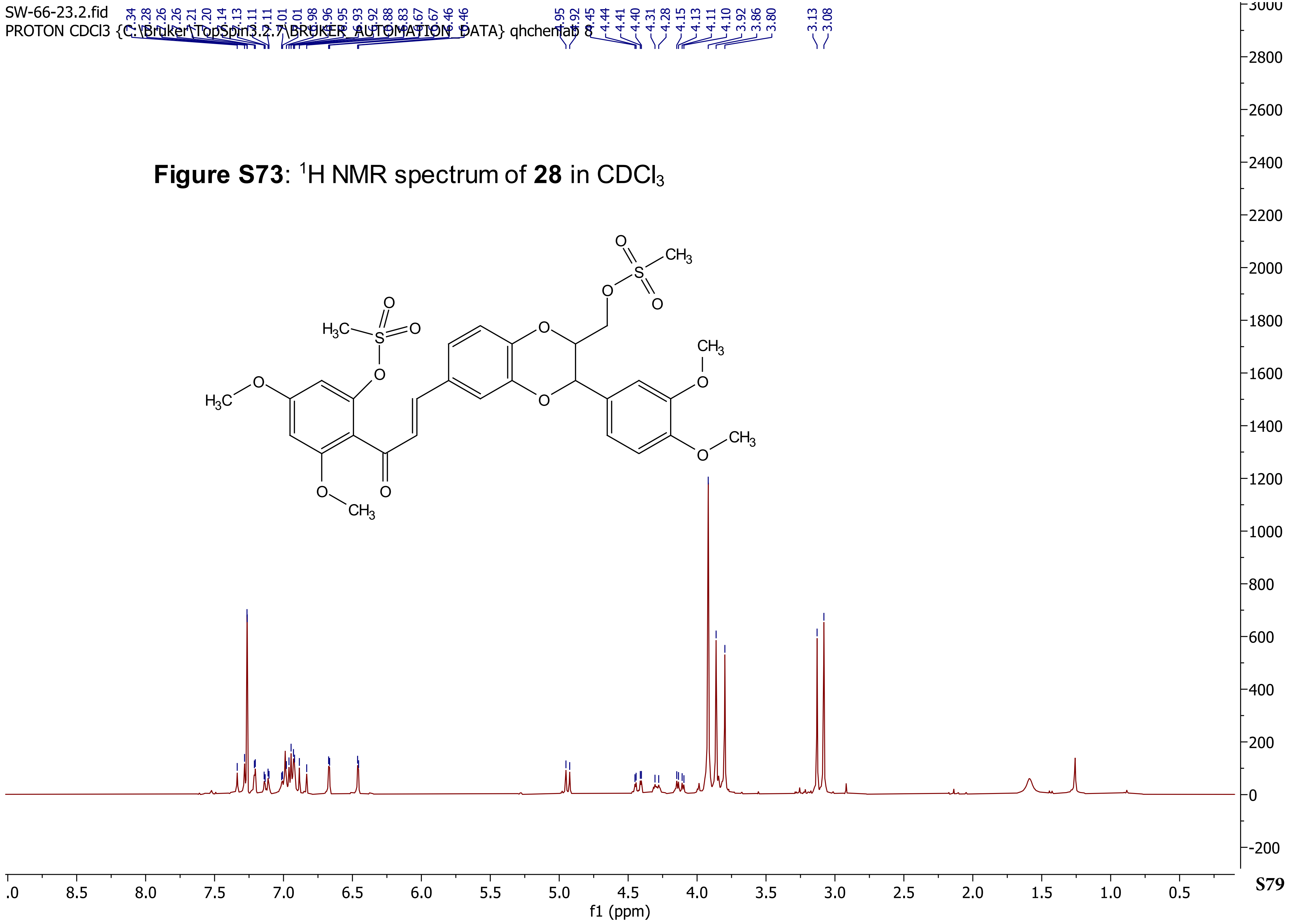

Figure S73: ^{1}H NMR spectrum of **28** in $CDCl_3$

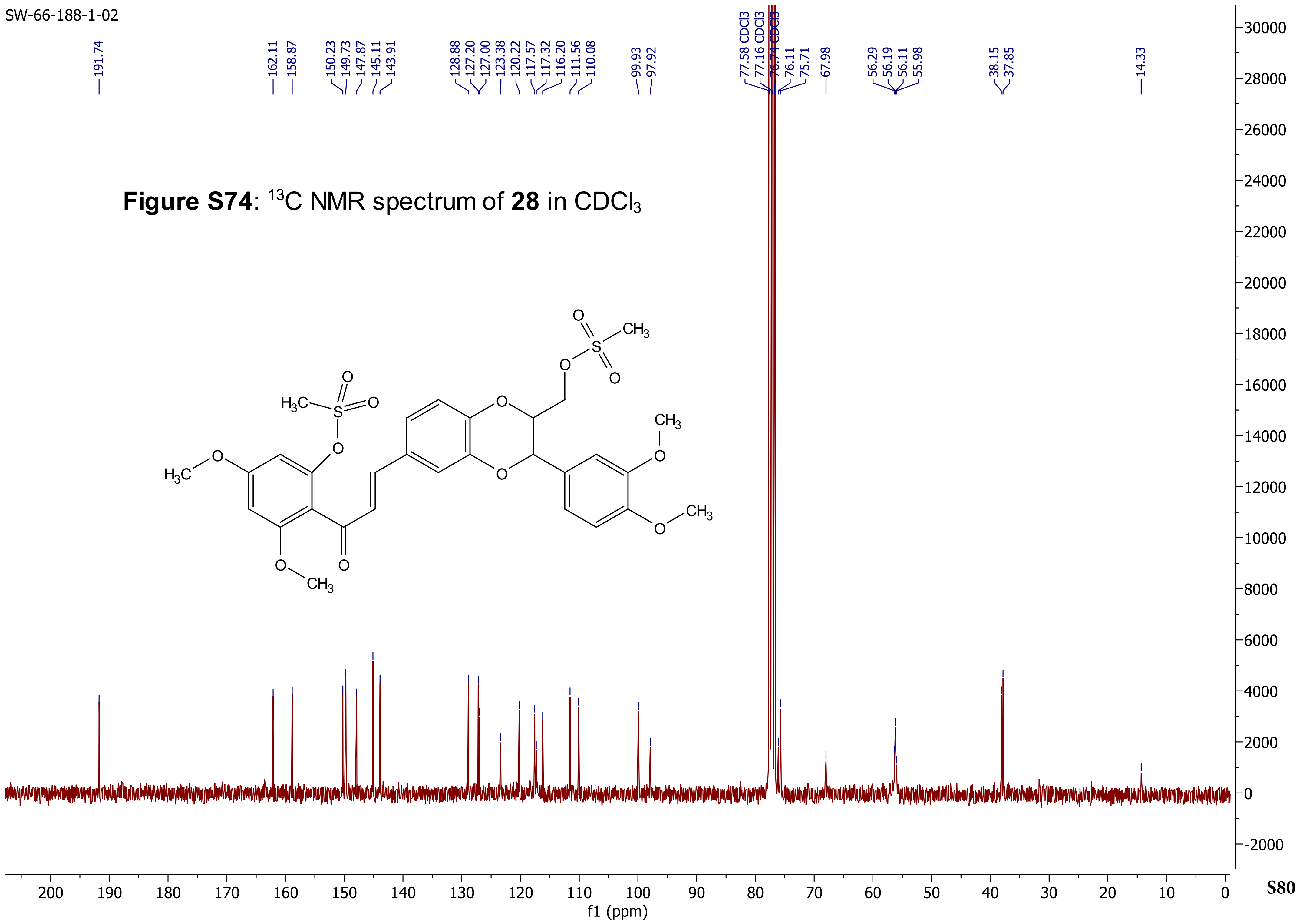

Figure S74: ^{13}C NMR spectrum of **28** in $CDCl_3$

SW-66-135-02	C30H32O13S2	664.1285	665.1363	**665.1385**	0.0022	3.31

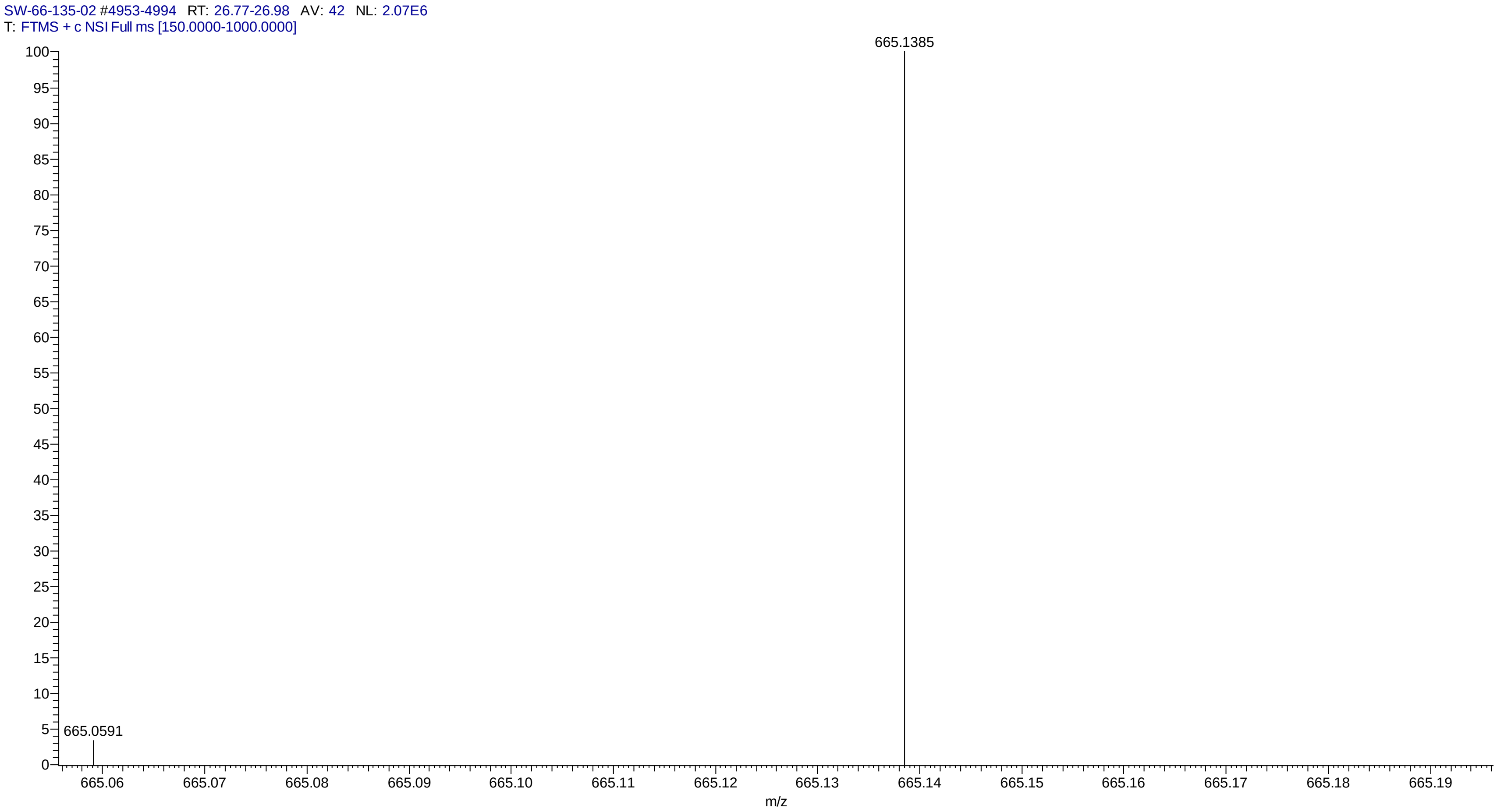

Figure S75: High resolution mass spectrum of **28**

```
=====================================================================
Injection Date  : 4/22/2022 11:25:52 AM
Sample Name     : SW-66-180-01                    Location : Vial 1
Acq. Operator   :
Acq. Method     : C:\HPCHEM\1\METHODS\JNP2015.M
Last changed    : 4/22/2022 10:36:46 AM
                  (modified after loading)
Analysis Method : C:\HPCHEM\1\METHODS\JNP2015.M
Last changed    : 4/22/2022 12:39:03 PM
                  (modified after loading)
```

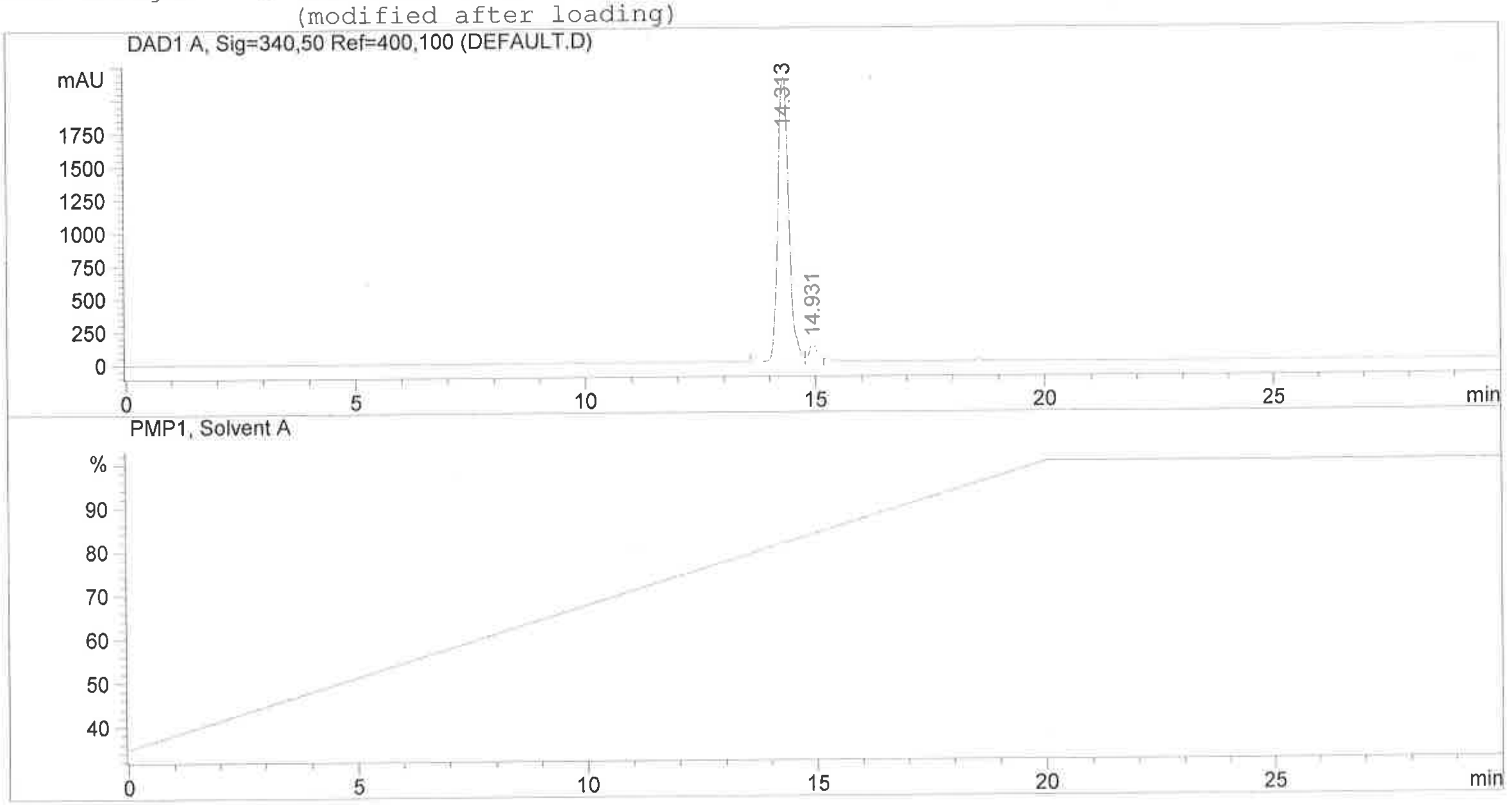


```
=====================================================================
                       Area Percent Report
=====================================================================

Sorted By             :      Signal
Multiplier            :      1.0000
Dilution              :      1.0000

Signal 1: DAD1 A, Sig=340,50 Ref=400,100
```

Peak #	RetTime [min]	Type	Width [min]	Area [mAU*s]	Height [mAU]	Area %
1	14.313	VV	0.2738	3.53433e4	2151.28345	95.4326
2	14.931	VV	0.1986	1691.51160	141.98376	4.5674

```
Totals :                    3.70348e4  2293.26721

 Results obtained with enhanced integrator!
=====================================================================
                         *** End of Report ***
```

Figure S76: HPLC chromatogram of **28**

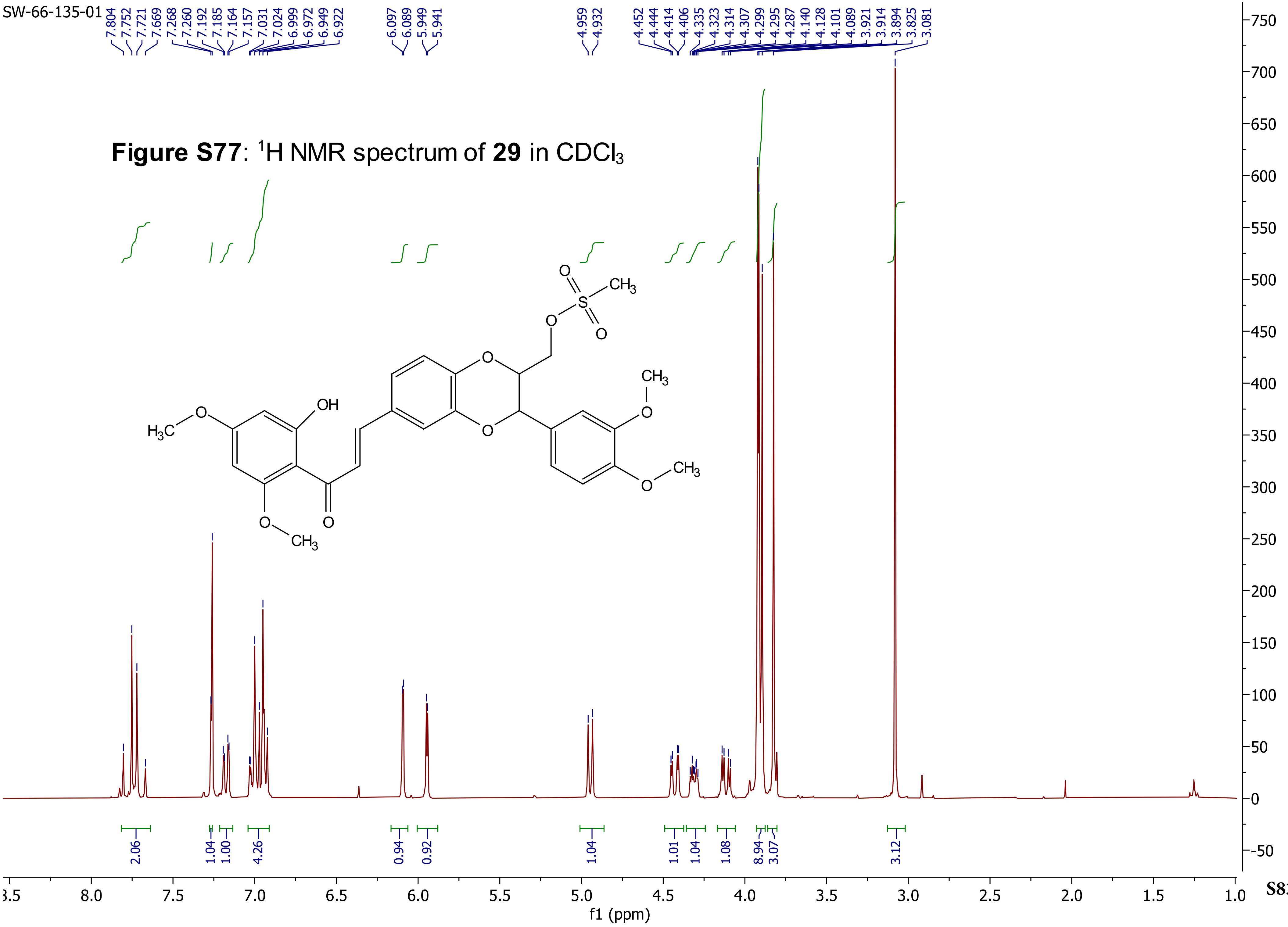

Figure S77: ^{1}H NMR spectrum of **29** in $CDCl_3$

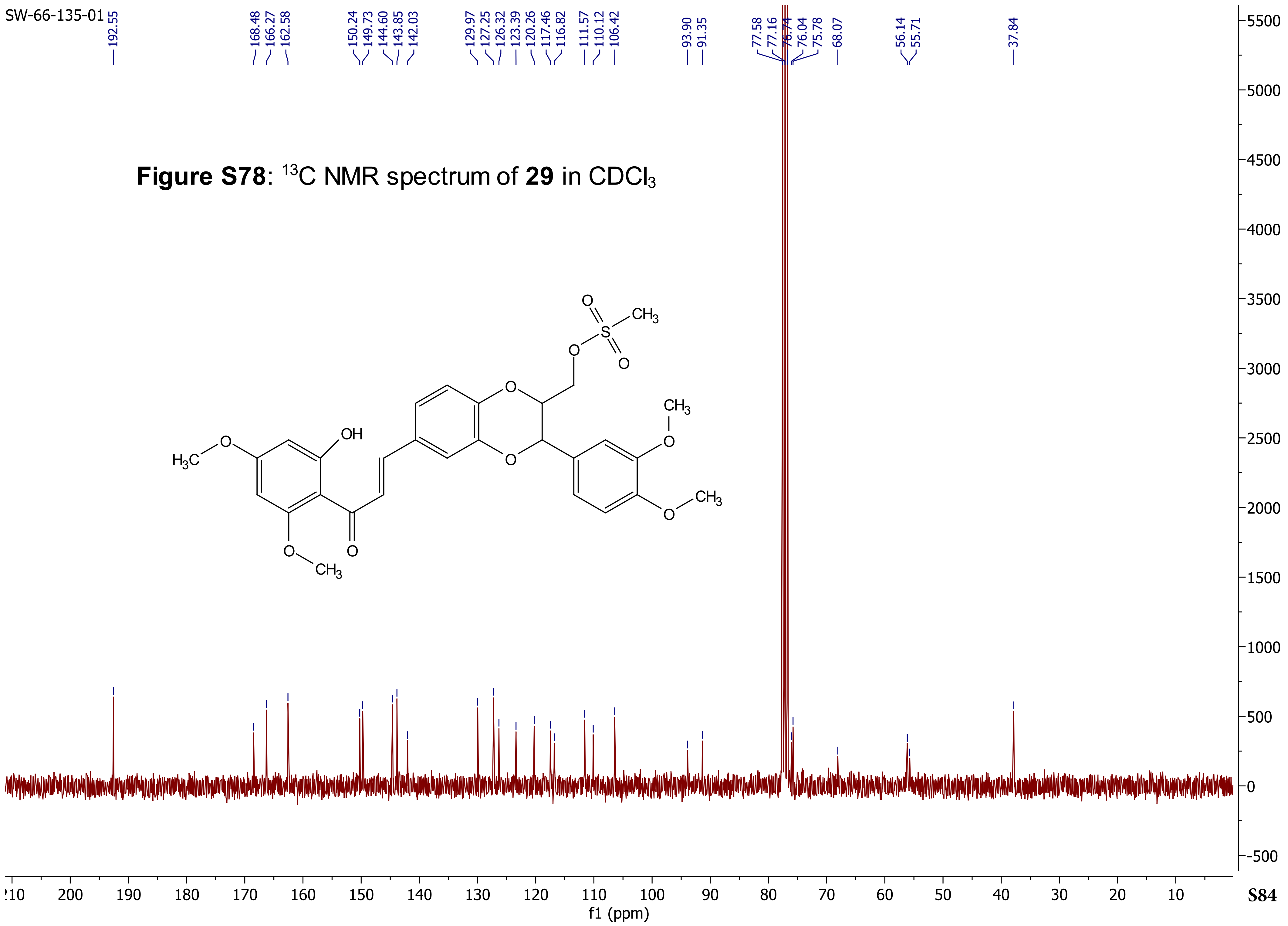

Figure S78: ^{13}C NMR spectrum of **29** in $CDCl_3$

SW-66-135-01	C29H30O11S	586.1509	587.1587	**587.1587**	0.0000	0.00

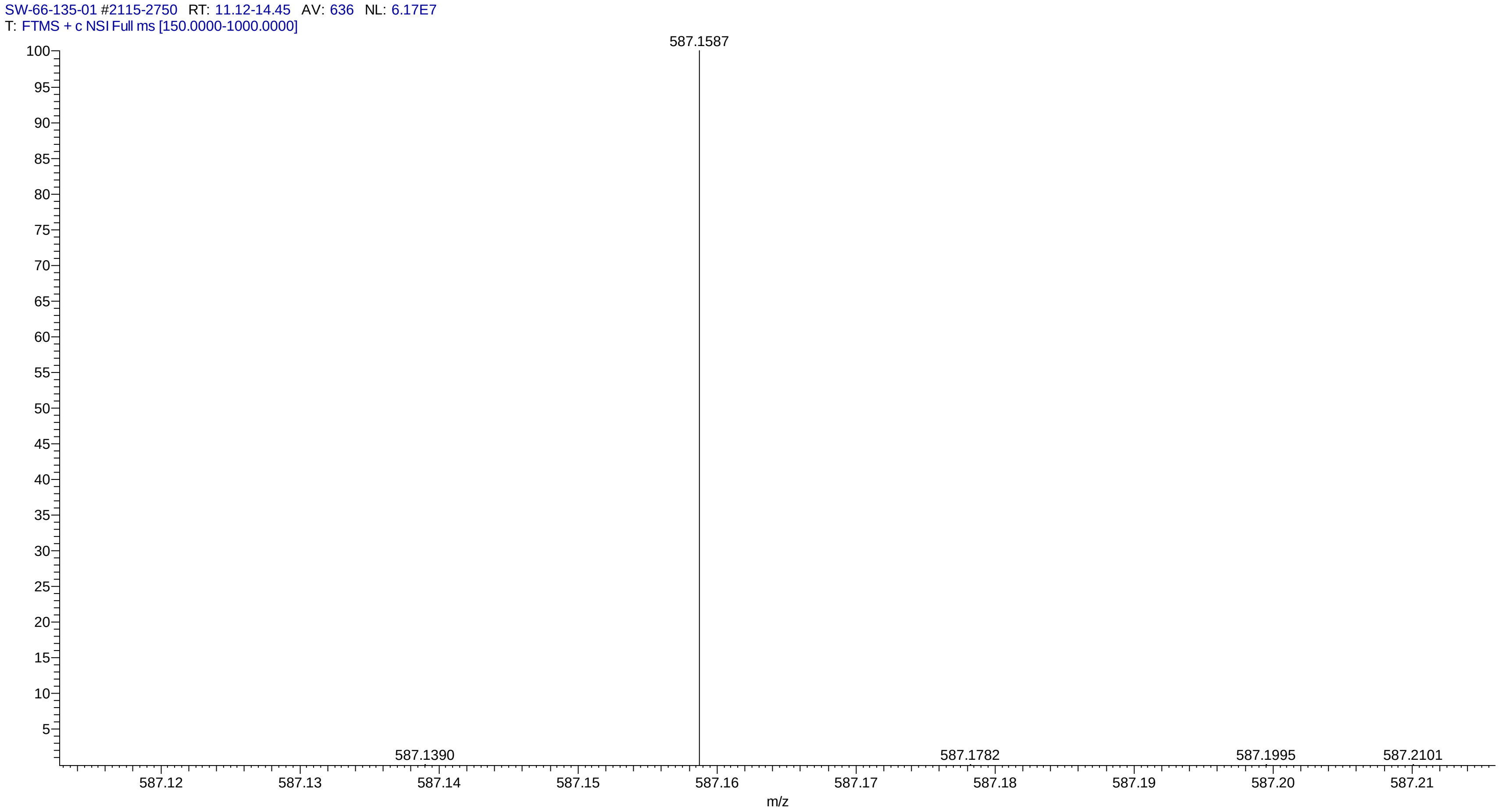

Figure S79: High resolution mass spectrum of **29**

```
=====================================================================
Injection Date  : 4/21/2022 3:29:34 PM
Sample Name     : SW-66-135-01                      Location : Vial 1
Acq. Operator   :
Acq. Method     : C:\HPCHEM\1\METHODS\JNP2015.M
Last changed    : 4/21/2022 3:19:41 PM
                  (modified after loading)
Analysis Method : C:\HPCHEM\1\METHODS\JNP2015.M
Last changed    : 4/21/2022 4:08:06 PM
                  (modified after loading)
```

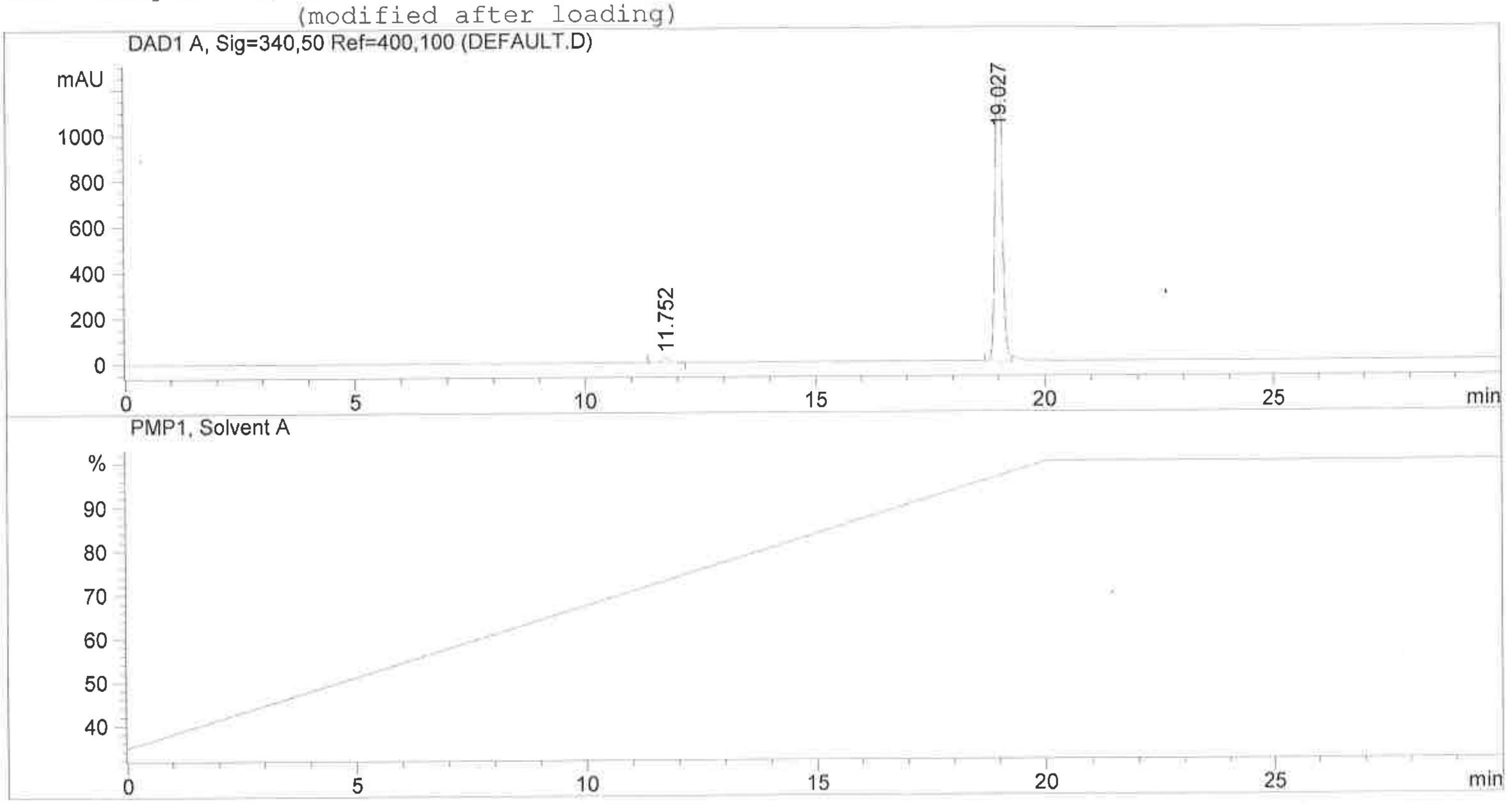


```
=====================================================================
                        Area Percent Report
=====================================================================

Sorted By             :      Signal
Multiplier            :      1.0000
Dilution              :      1.0000


Signal 1: DAD1 A, Sig=340,50 Ref=400,100

Peak RetTime Type  Width     Area      Height     Area
  #   [min]        [min]   [mAU*s]     [mAU]        %
----|-------|----|-------|----------|----------|--------|
   1  11.752 BB    0.1707  179.36371   17.51204   1.2375
   2  19.027 VV    0.1917 1.43146e4  1244.38928  98.7625

Totals :                  1.44940e4  1261.90132

 Results obtained with enhanced integrator!
=====================================================================
                        *** End of Report ***
```

Figure S80: HPLC chromatogram of **29**

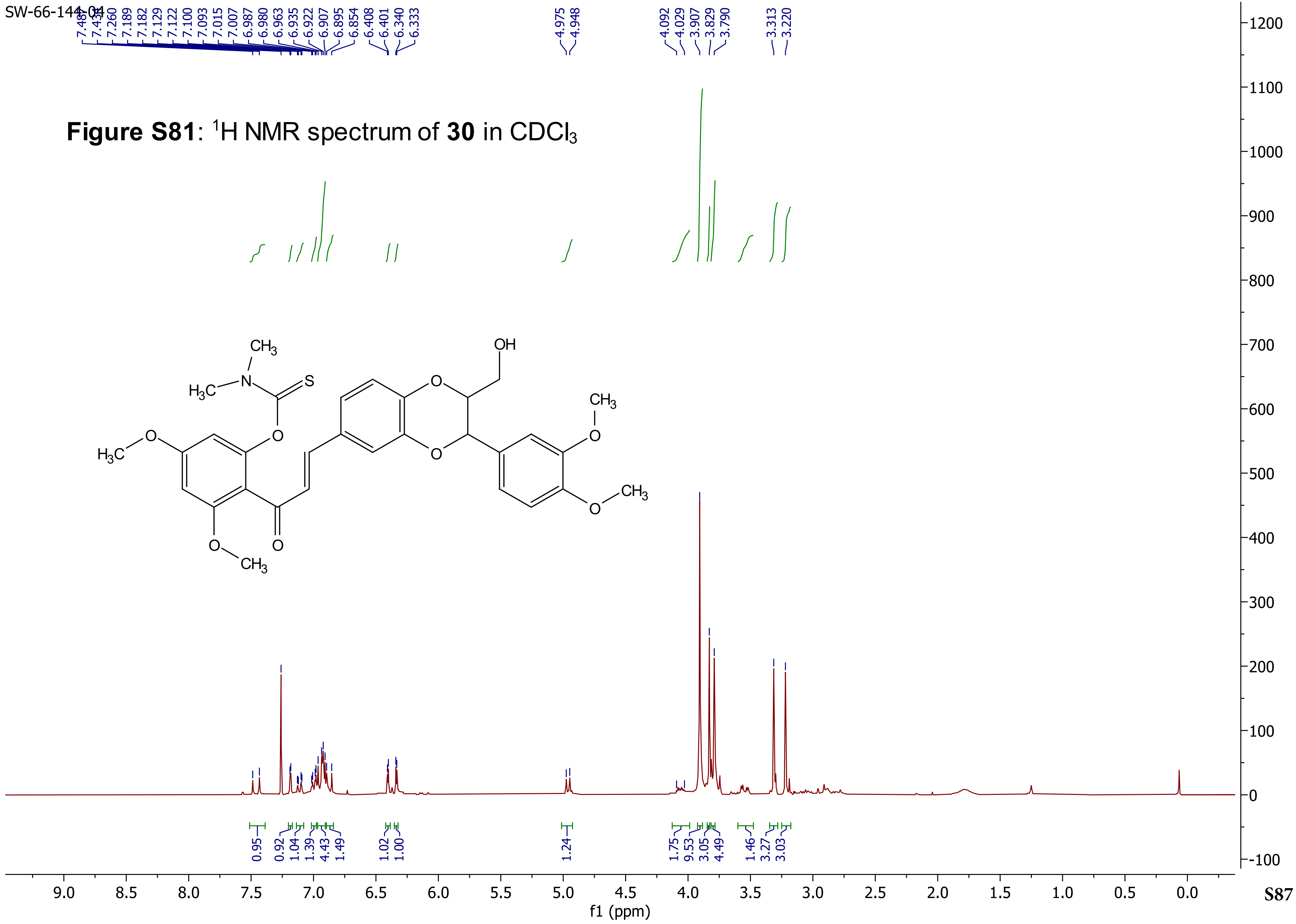

Figure S81: ^{1}H NMR spectrum of **30** in $CDCl_3$

SW-66-144-04

Figure S82: ^{13}C NMR spectrum of **30** in $CDCl_3$

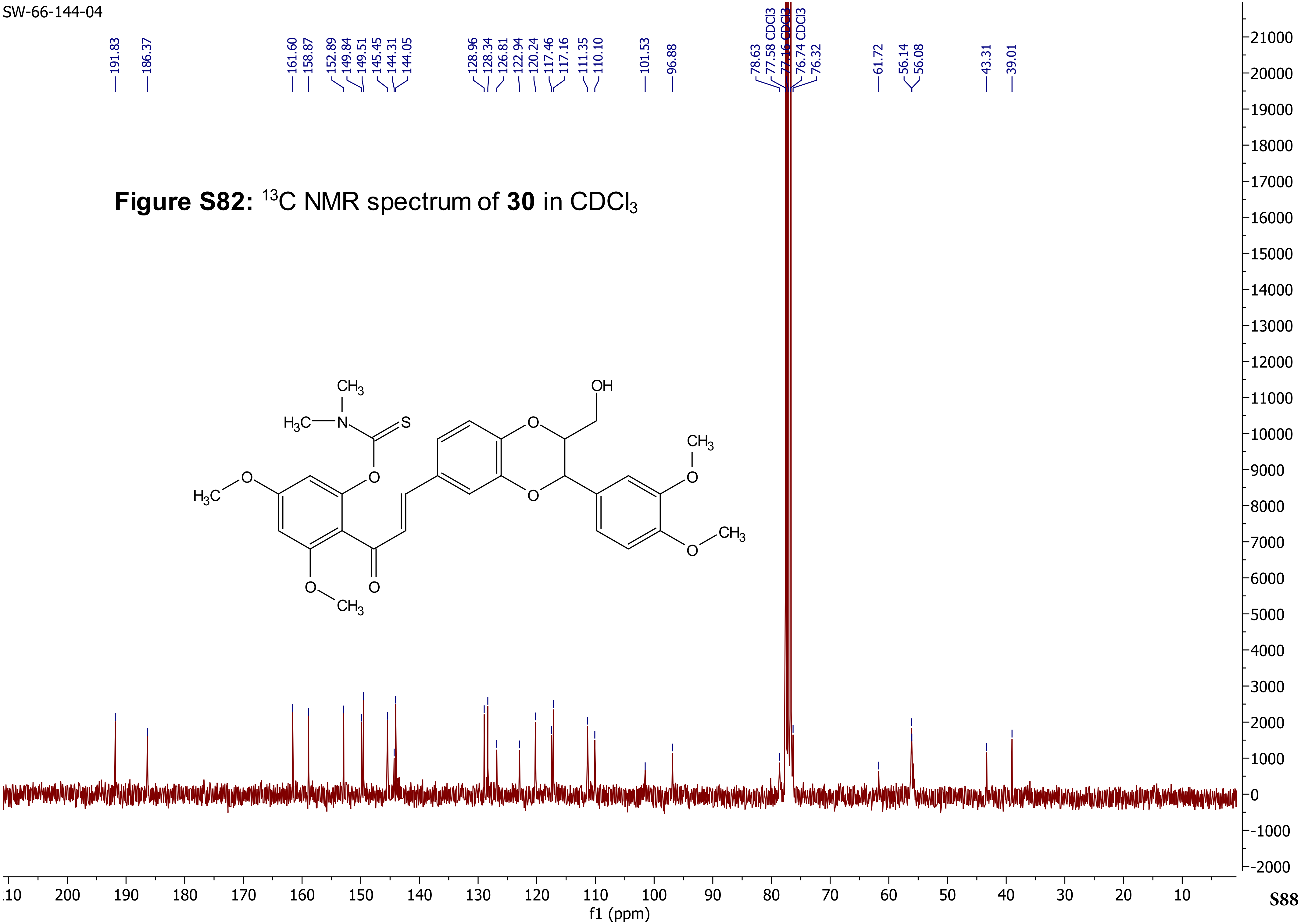

SW-66-15	C31H33NO9S	595.1876	596.1954	**596.1950**	-0.0004	-0.74

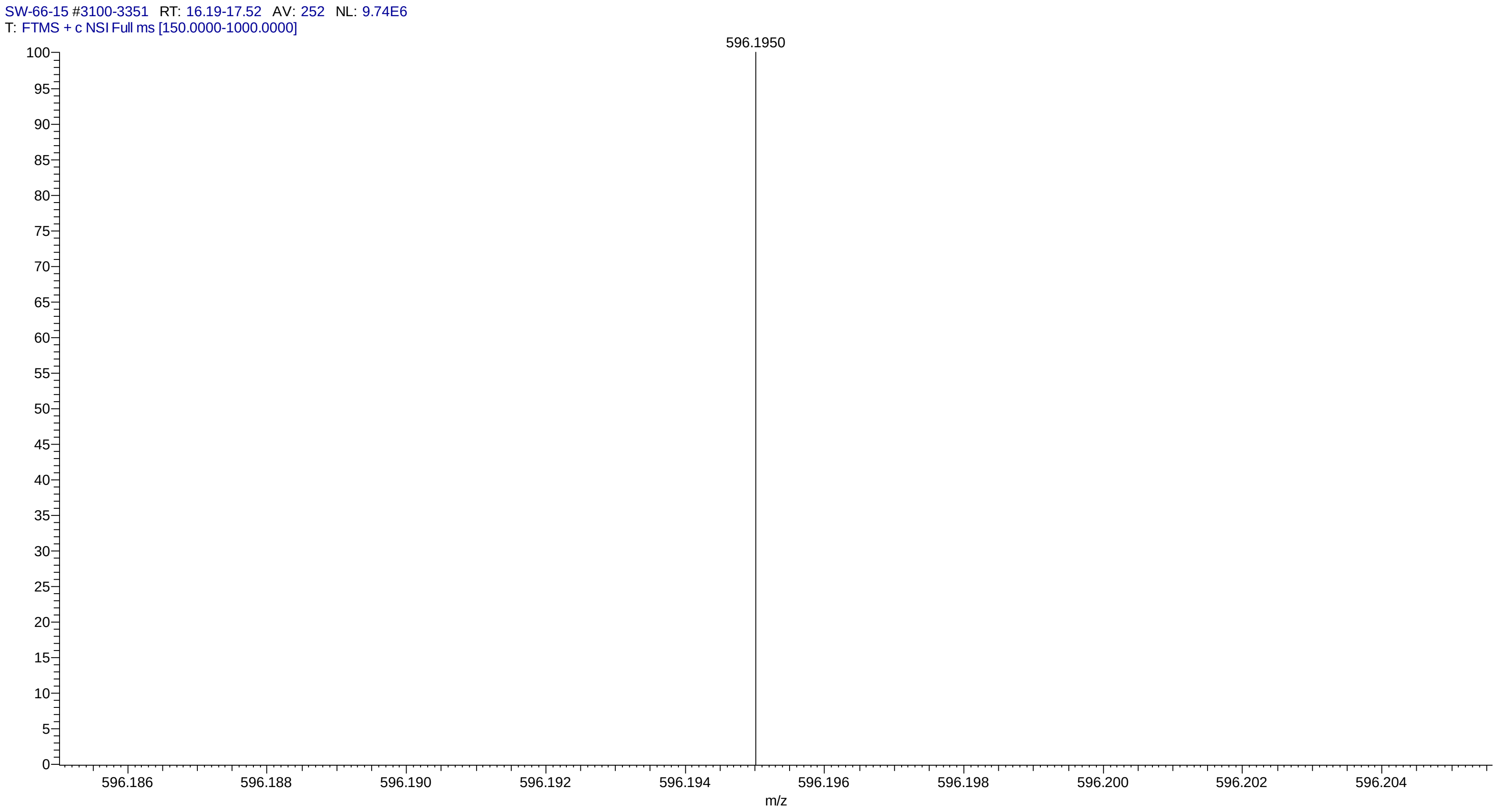

Figure S83: High resolution mass spectrum of **30**

===

Injection Date : 4/23/2022 1:08:04 PM
Sample Name : SW-66-144-04 Location : Vial 1
Acq. Operator :
Method : C:\HPCHEM\1\METHODS\JNP2015.M
Last changed : 4/22/2022 5:19:36 PM
(modified after loading)

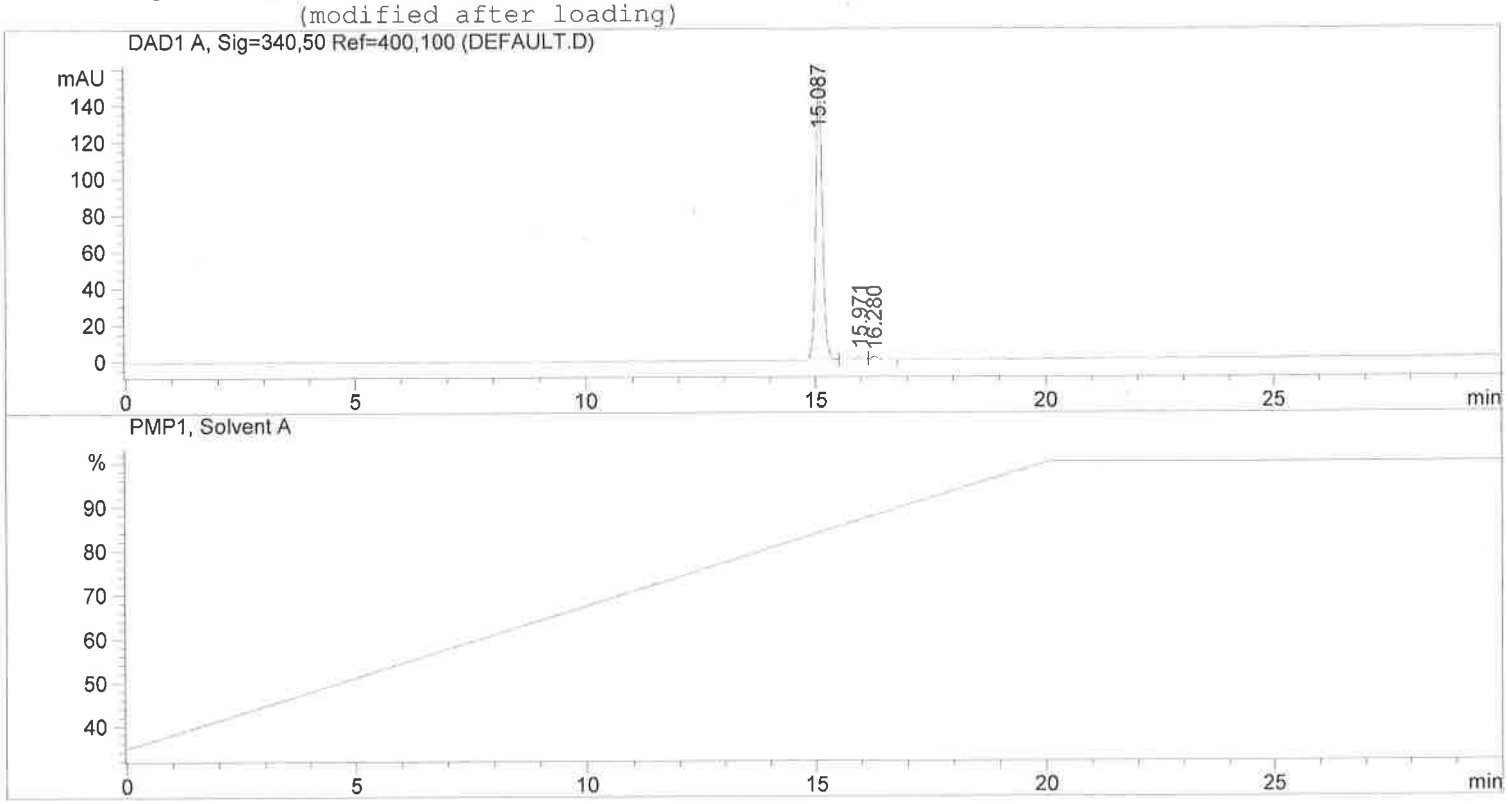

===
Area Percent Report
===

Sorted By : Signal
Multiplier : 1.0000
Dilution : 1.0000

Signal 1: DAD1 A, Sig=340,50 Ref=400,100

Peak #	RetTime [min]	Type	Width [min]	Area [mAU*s]	Height [mAU]	Area %
1	15.087	BV	0.1499	1531.83569	154.23935	96.8516
2	15.971	VV	0.1619	31.13817	2.84065	1.9687
3	16.280	VP	0.1552	18.65778	1.79653	1.1797

Totals : 1581.63164 158.87653

Results obtained with enhanced integrator!
===
*** End of Report ***

Figure S84: HPLC chromatogram of **30**

SW-66-144-02

Figure S85: ^1H NMR spectrum of **31** in $CDCl_3$

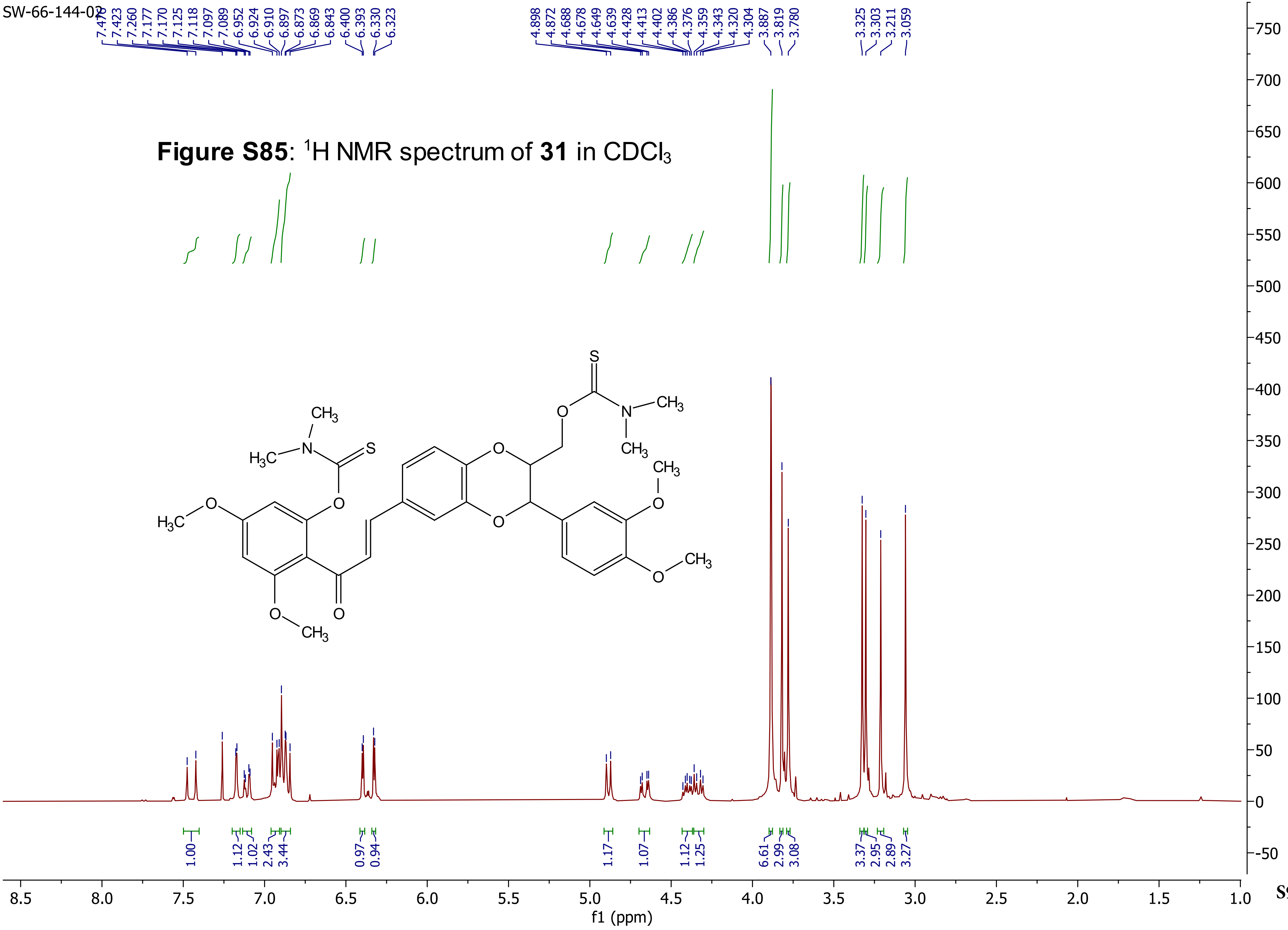

SW-66-144-02

Figure S86: ^{13}C NMR spectrum of **31** in $CDCl_3$

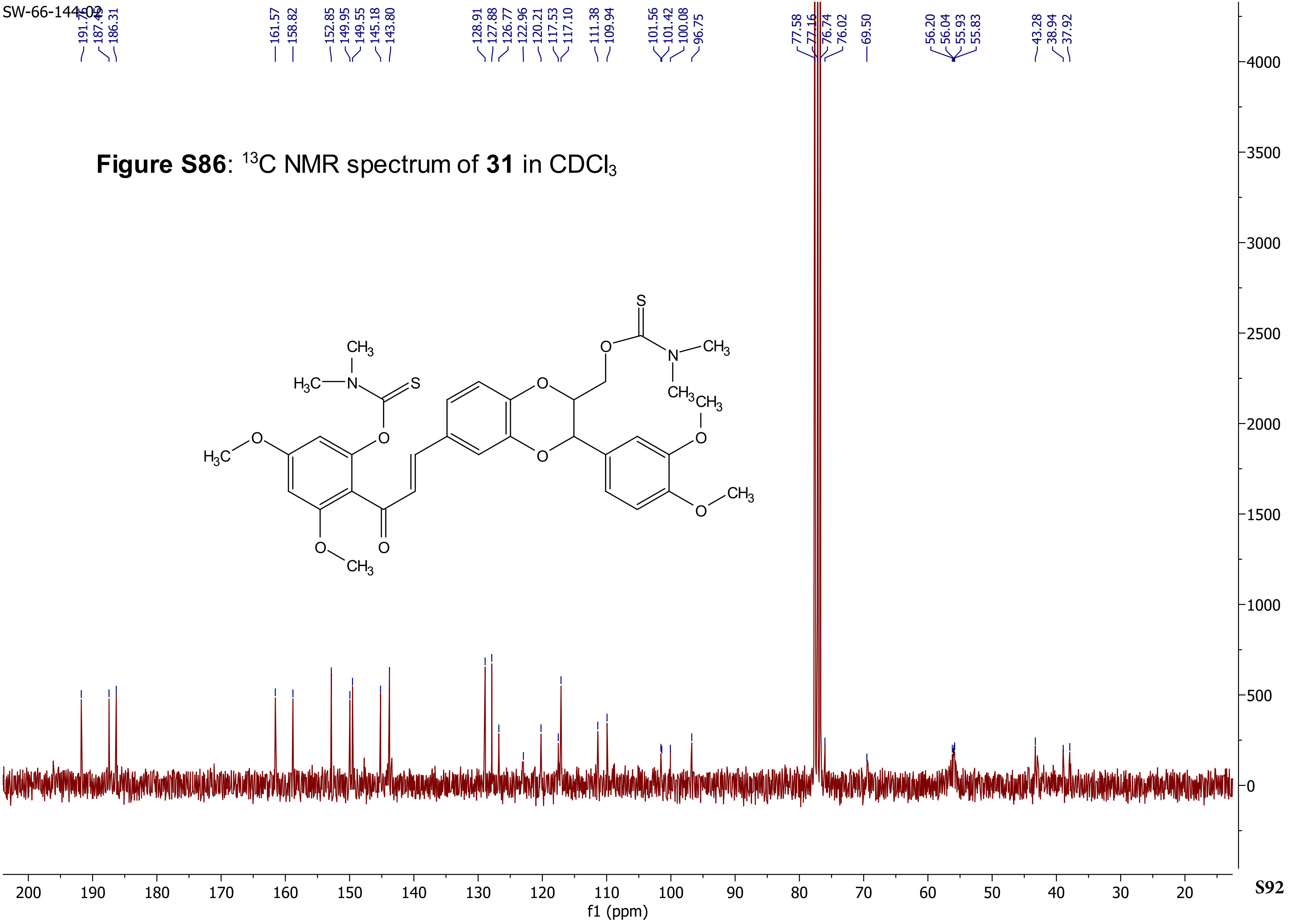

SW-66-144-02	C34H38N2O9S2	682.2019	683.2097	**683.2118**	0.0021	3.04

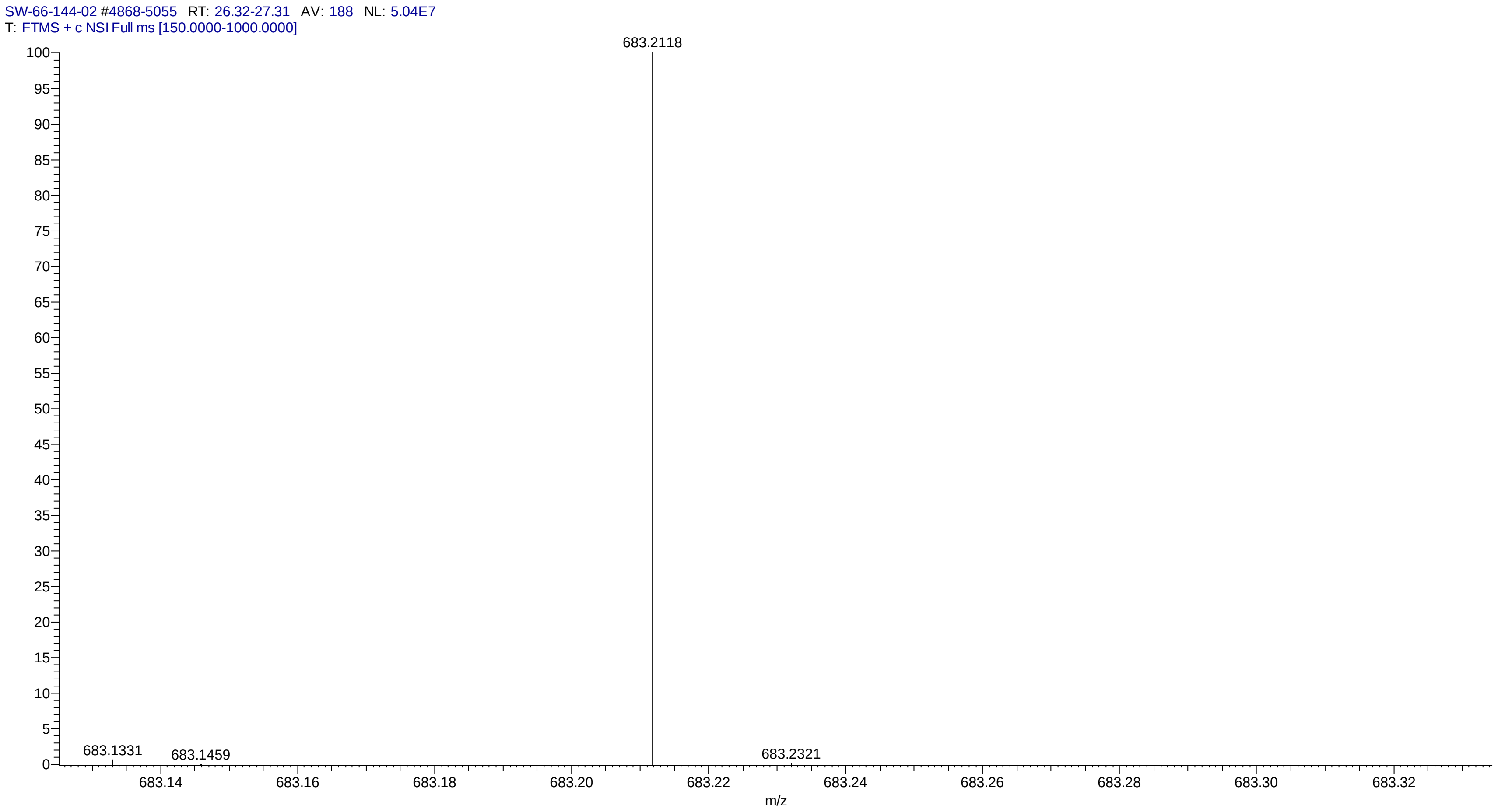

Figure S87: High resolution mass spectrum of **31**

===

Injection Date : 4/22/2022 5:19:42 PM
Sample Name : SW-66-144-02 Location : Vial 1
Acq. Operator :
Method : C:\HPCHEM\1\METHODS\JNP2015.M
Last changed : 4/22/2022 5:19:36 PM
(modified after loading)

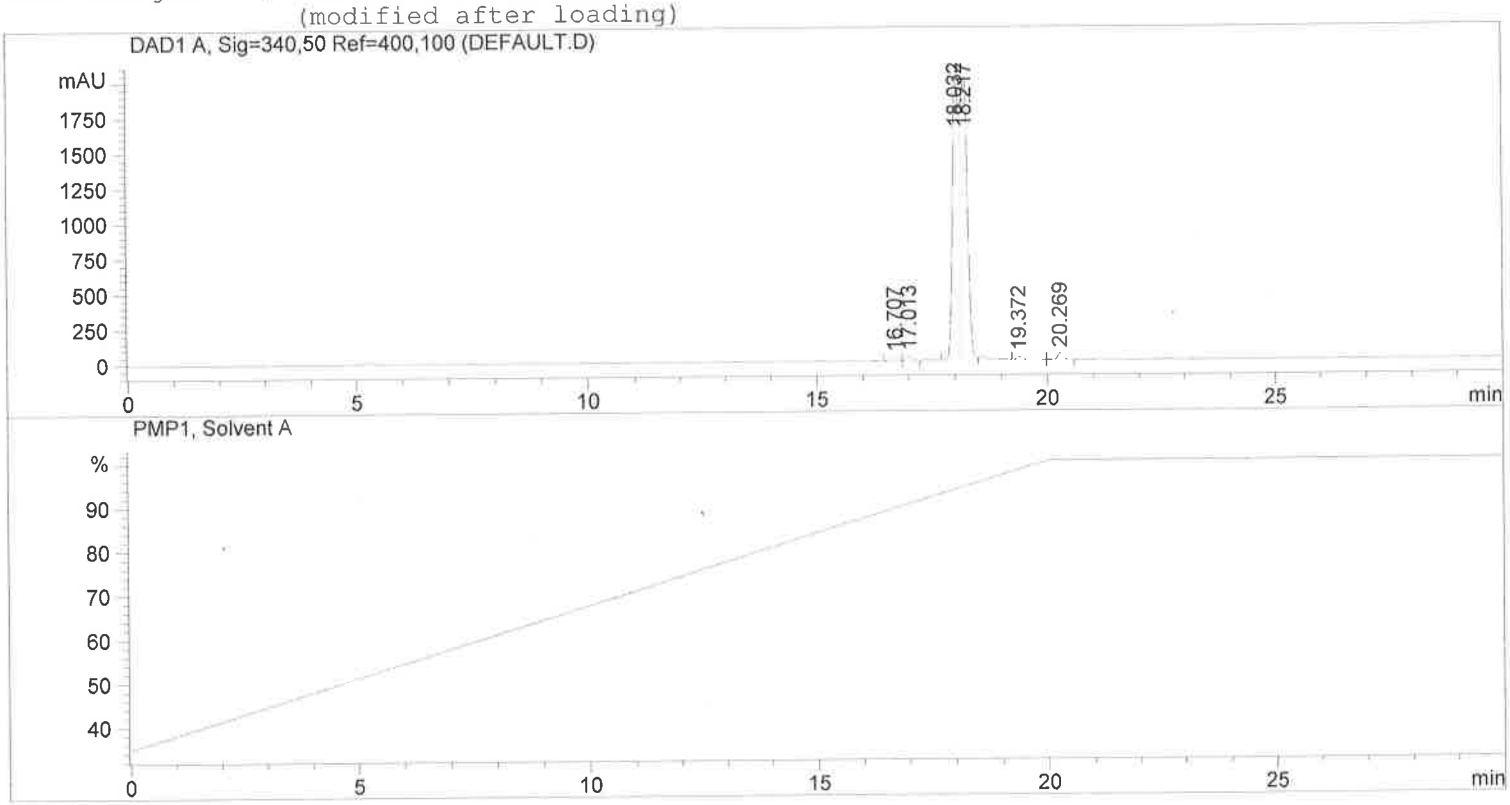

===

Area Percent Report

===

Sorted By : Signal
Multiplier : 1.0000
Dilution : 1.0000

Signal 1: DAD1 A, Sig=340,50 Ref=400,100

Peak #	RetTime [min]	Type	Width [min]	Area [mAU*s]	Height [mAU]	Area %
1	16.707	VV	0.1476	230.93034	24.14036	0.5562
2	17.013	VV	0.1470	310.72815	32.66385	0.7484
3	18.032	VV	0.1382	1.95639e4	1999.27563	47.1223
4	18.217	VV	0.1490	2.08223e4	2008.36292	50.1535
5	19.372	VV	0.1367	222.91071	23.91627	0.5369
6	20.269	VB	0.1201	366.44476	46.39542	0.8826
Totals :				4.15173e4	4134.75445	

Results obtained with enhanced integrator!

===

*** End of Report ***

Figure S88: HPLC chromatogram of **31**

SW-66-131-01

Figure S89: ^{1}H NMR Spectrum of **32** in $CDCl_3$

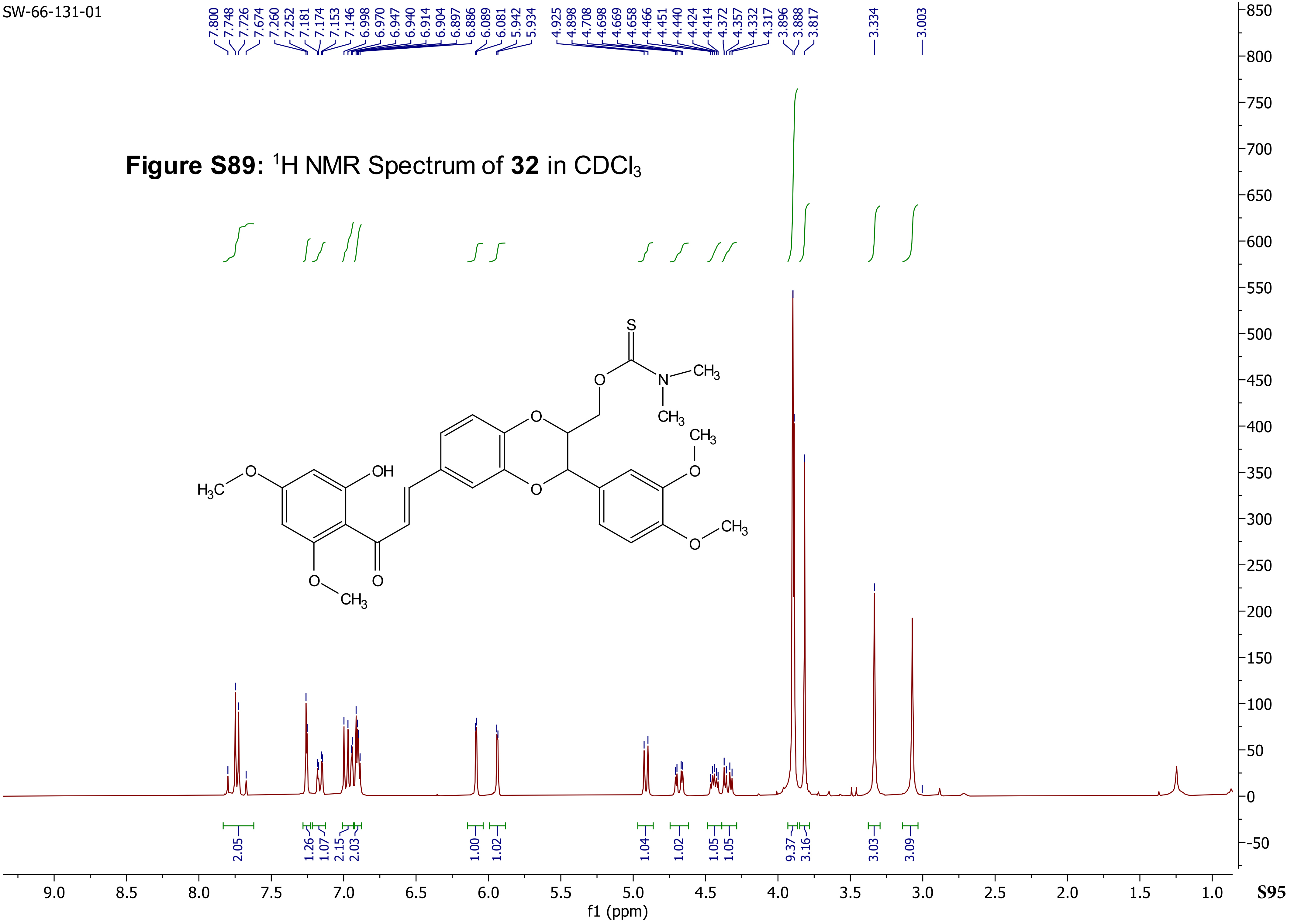

SW-66-131.1.01

Figure S90: ^{13}C NMR of **32** in $CDCl_3$

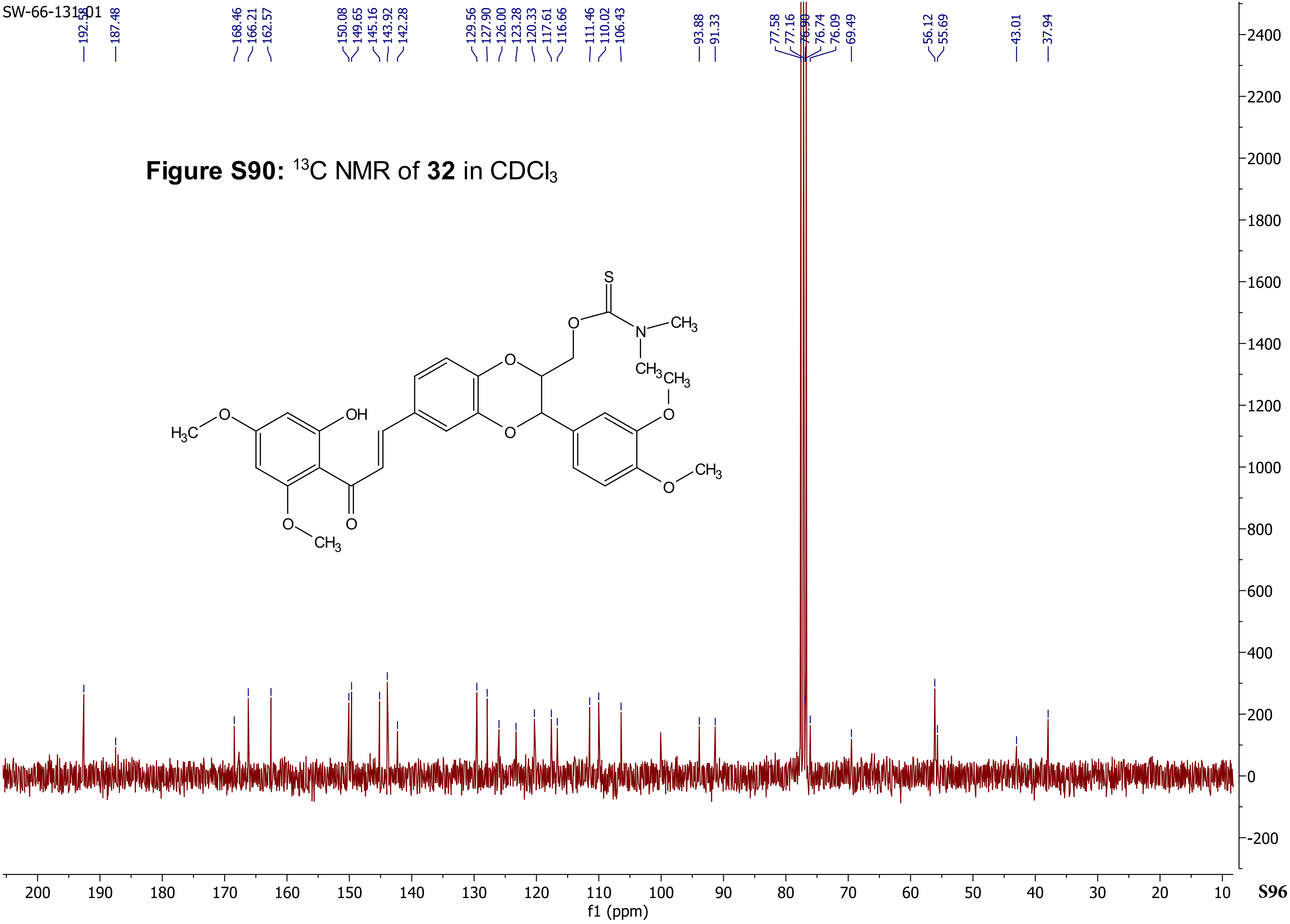

SW-66-131-01	C31H33NO9S	595.1876	596.1954	**596.1953**	-0.0001	-0.23

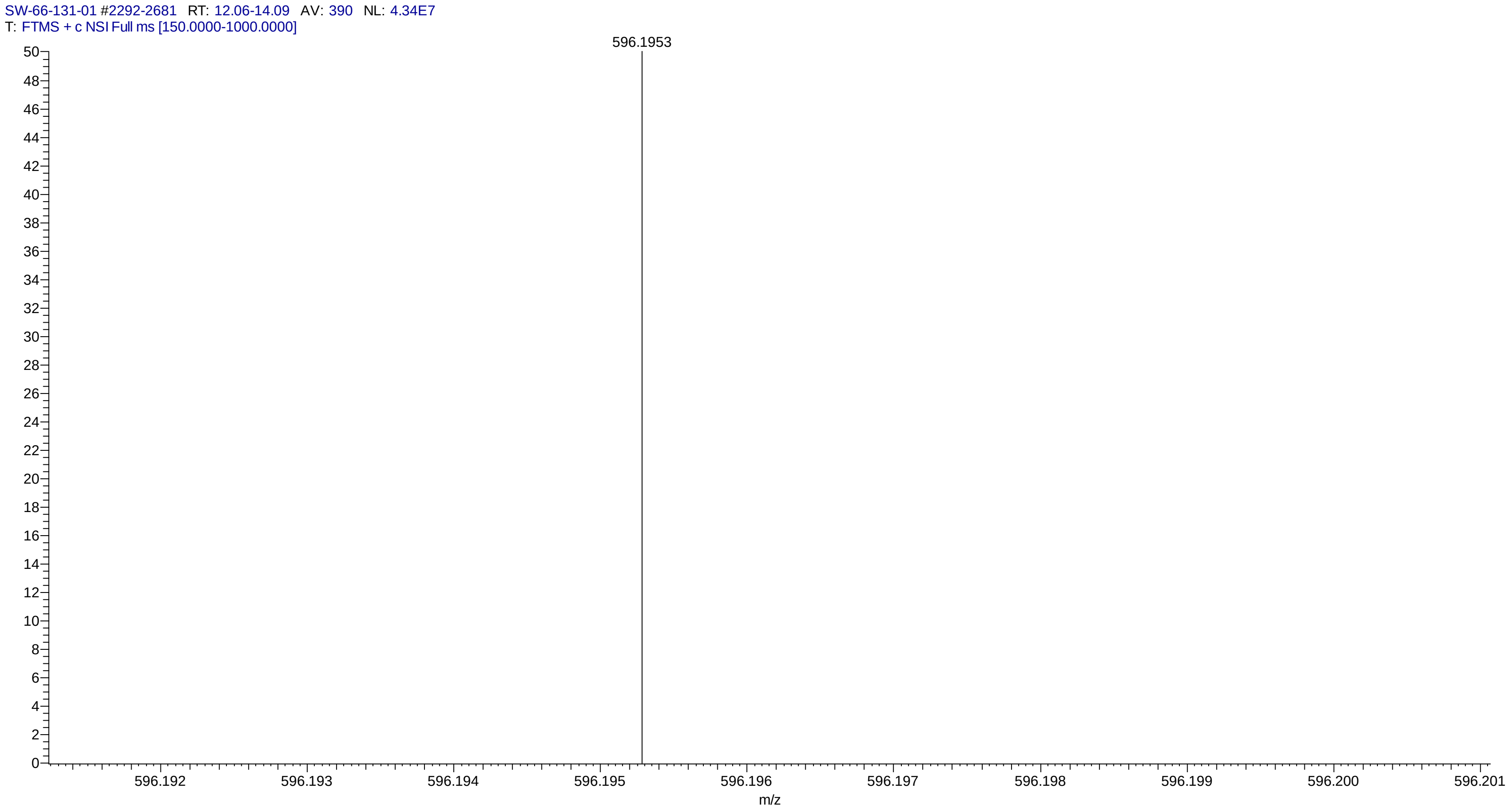

Figure S91: High resolution mass spectrum of **32**

===

Injection Date : 2/6/2023 12:29:49 PM
Sample Name : SW-66-144-01 Location : Vial 1
Acq. Operator :
Method : C:\HPCHEM\1\METHODS\JNP2015.M
Last changed : 1/31/2023 2:50:19 PM

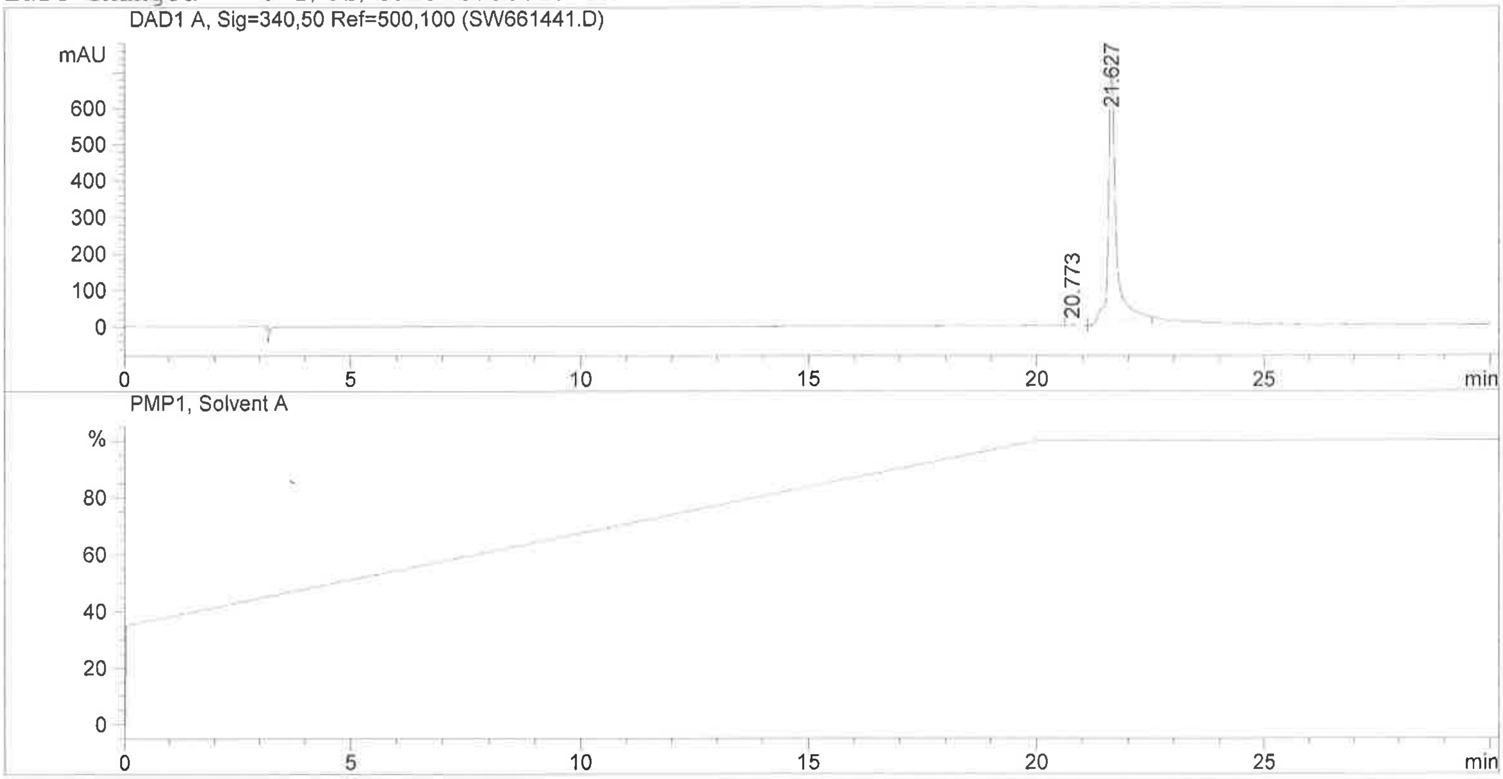

===

Area Percent Report

===

Sorted By : Signal
Multiplier : 1.0000
Dilution : 1.0000

Signal 1: DAD1 A, Sig=340,50 Ref=500,100

Peak #	RetTime [min]	Type	Width [min]	Area [mAU*s]	Height [mAU]	Area %
1	20.773	PP	0.1418	22.42879	2.29979	0.2767
2	21.627	VB	0.1575	8082.39355	727.97003	99.7233

Totals : 8104.82235 730.26982

Results obtained with enhanced integrator!

===

*** End of Report ***

Figure S92: HPLC chromatogram of **32**

SW-66-140-01

Figure S93: ^{1}H NMR spectrum of **33** in $CDCl_3$

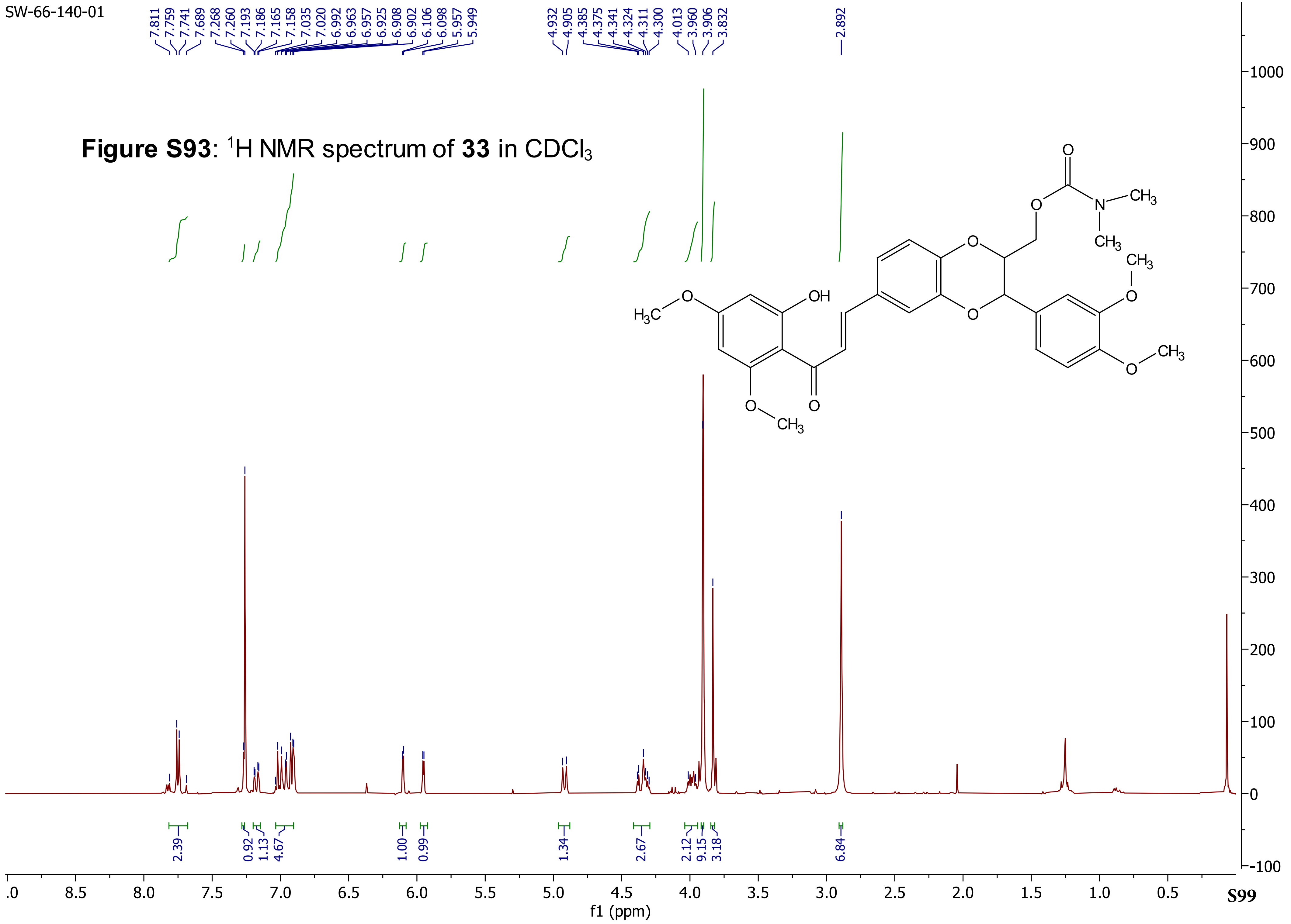

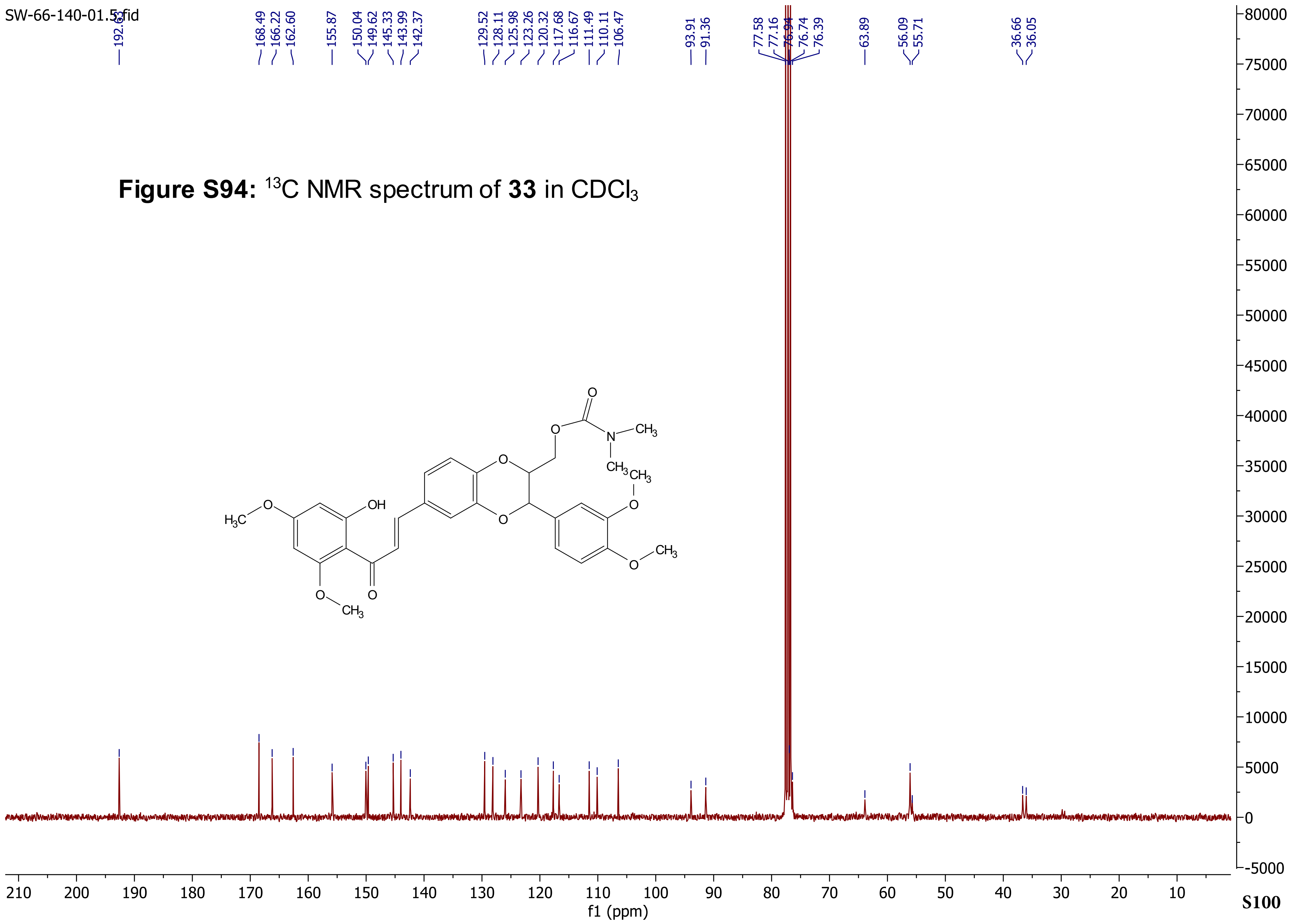

Figure S94: ^{13}C NMR spectrum of **33** in $CDCl_3$

SW-66-110	C34H42O9Si	622.2599	623.2677	**623.2675**	-0.0002	-0.26

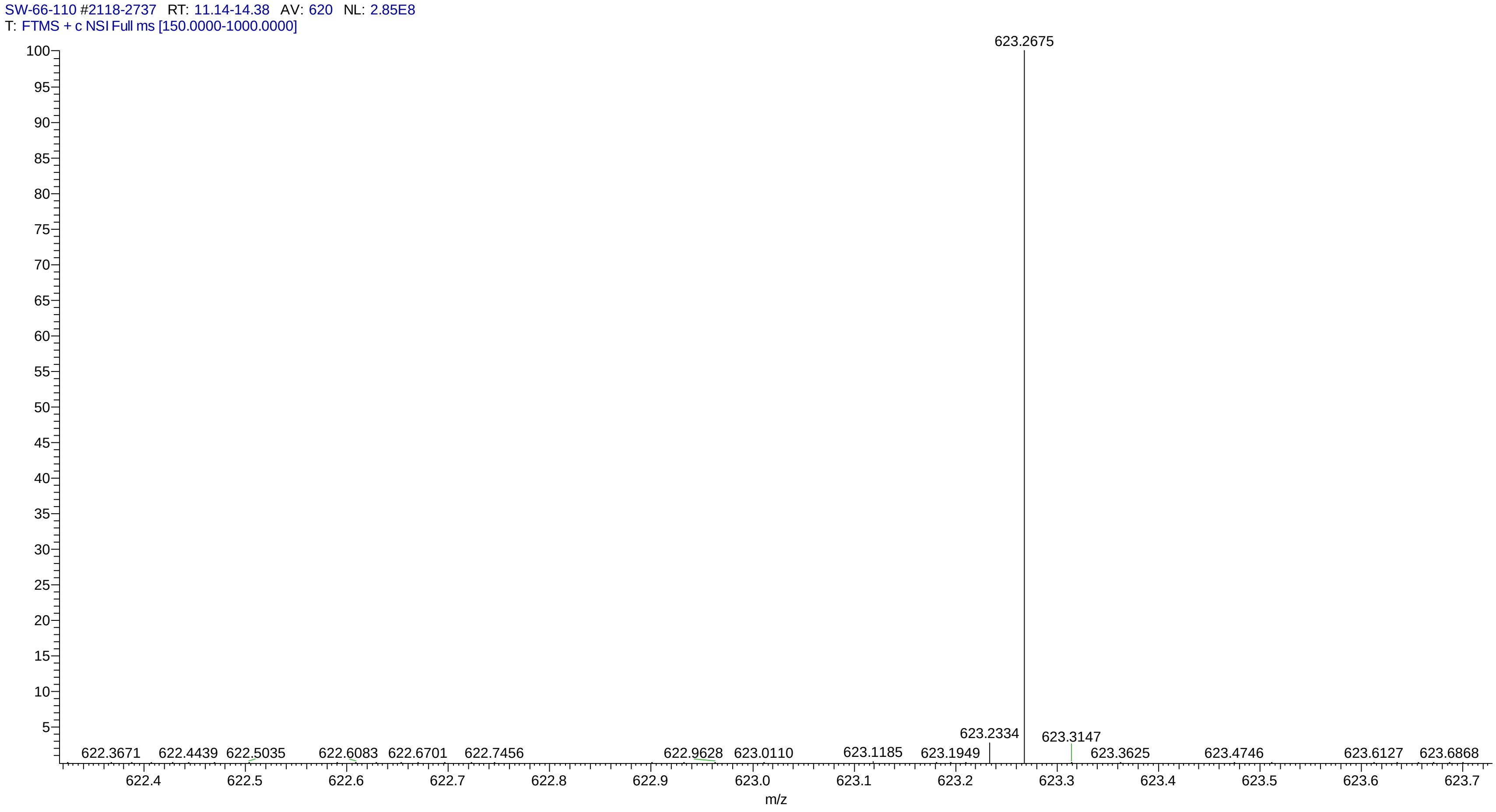

Figure S55: High resolution mass spectrum of **23**

```
=====================================================================
Injection Date  : 2/6/2023 11:14:57 AM
Sample Name     : SW-66-140-01                   Location : Vial 1
Acq. Operator   :
Method          : C:\HPCHEM\1\METHODS\JNP2015.M
Last changed    : 1/31/2023 2:50:19 PM
```

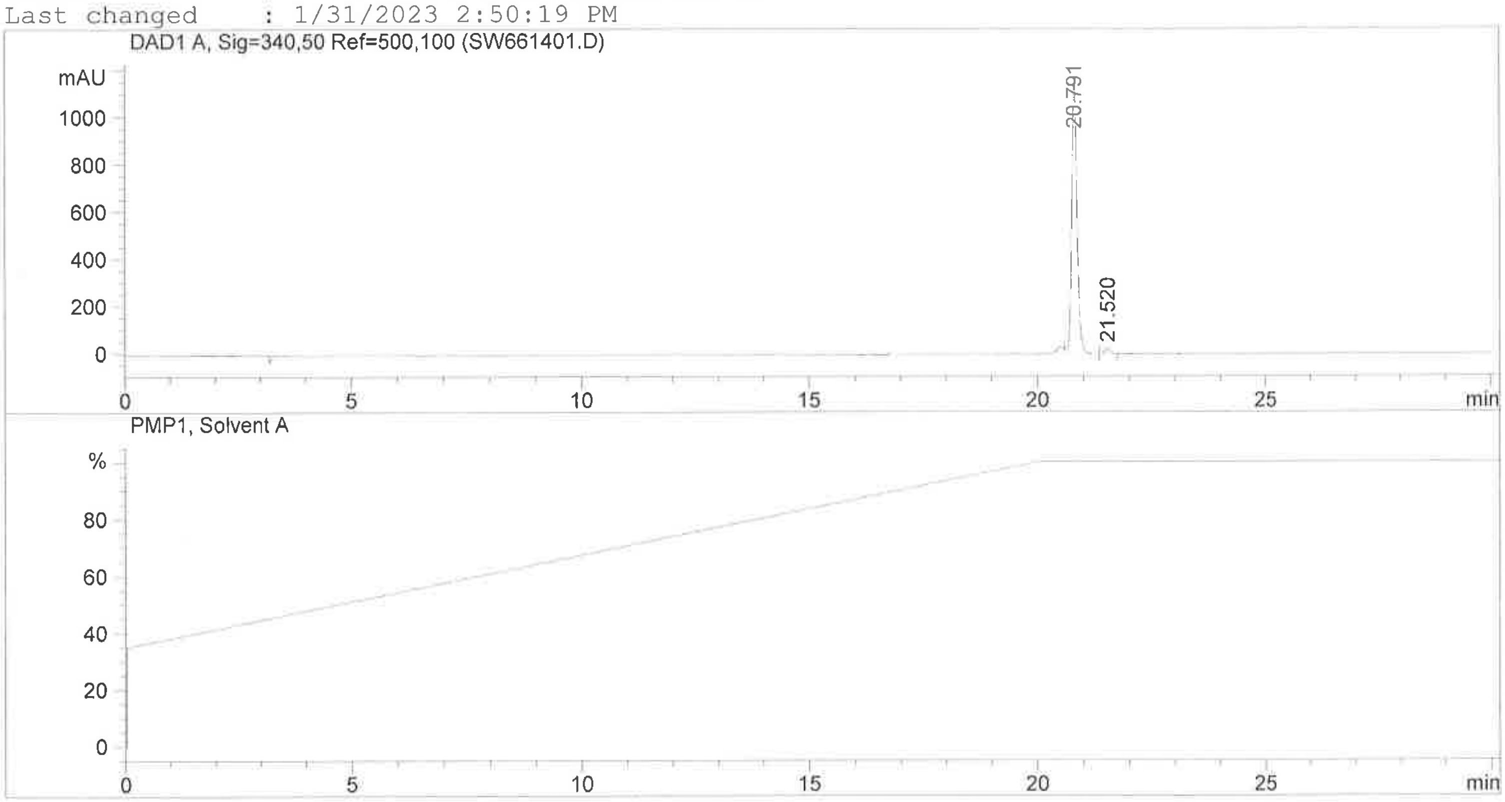


```
=====================================================================
                        Area Percent Report
=====================================================================

Sorted By             :      Signal
Multiplier            :      1.0000
Dilution              :      1.0000

Signal 1: DAD1 A, Sig=340,50 Ref=500,100
```

Peak #	RetTime [min]	Type	Width [min]	Area [mAU*s]	Height [mAU]	Area %
1	20.791	VV	0.1214	9306.10449	1162.17249	97.7461
2	21.520	VV	0.1277	214.58498	25.10204	2.2539

Totals :	9520.68947	1187.27452

Results obtained with enhanced integrator!

```
=====================================================================
                        *** End of Report ***
```

Figure S96: HPLC chromatogram of **33**

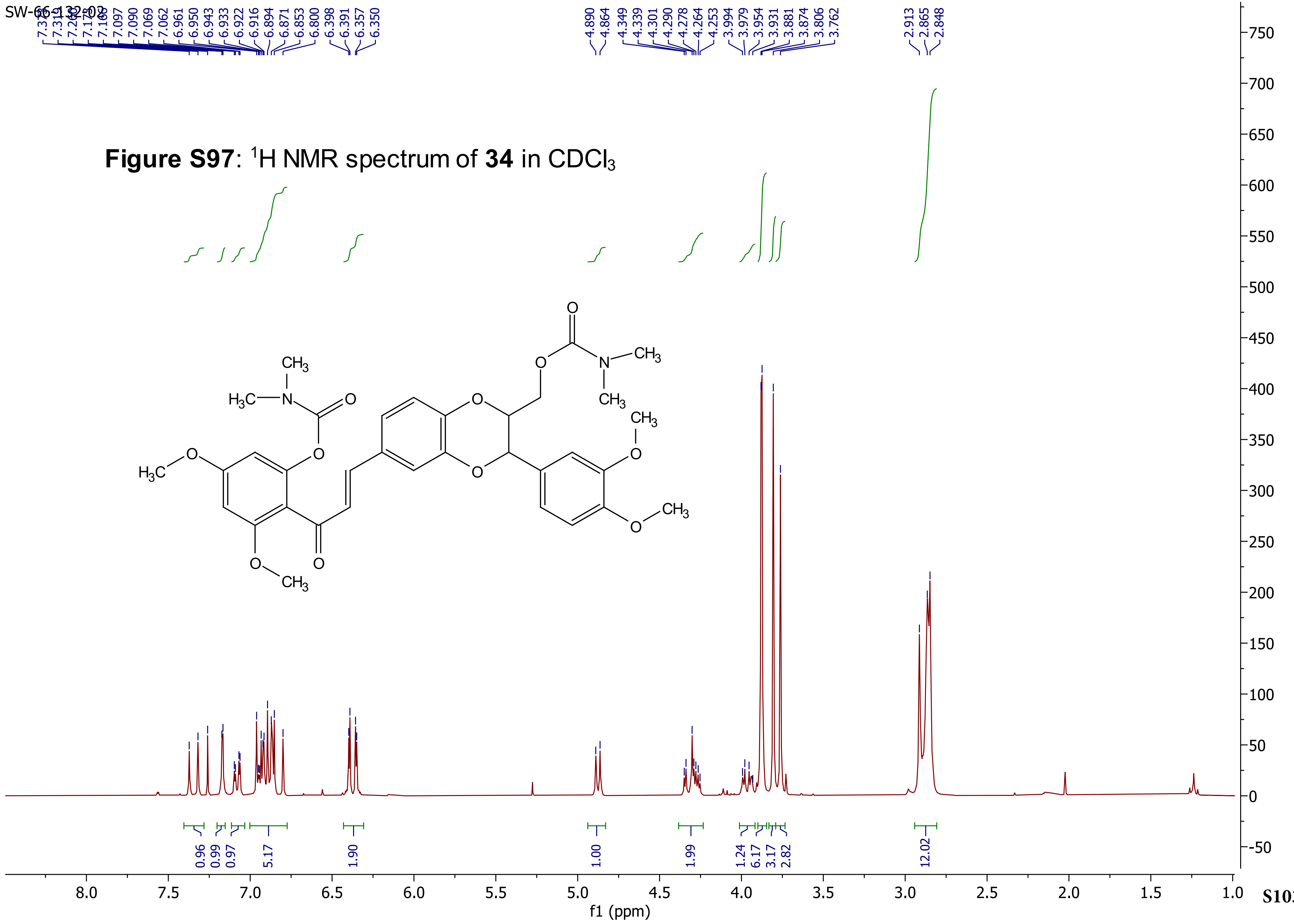

Figure S97: ^{1}H NMR spectrum of **34** in $CDCl_3$

SW-66-85-01

Figure S98: ^{13}C NMR spectrum of **34** in $CDCl_3$

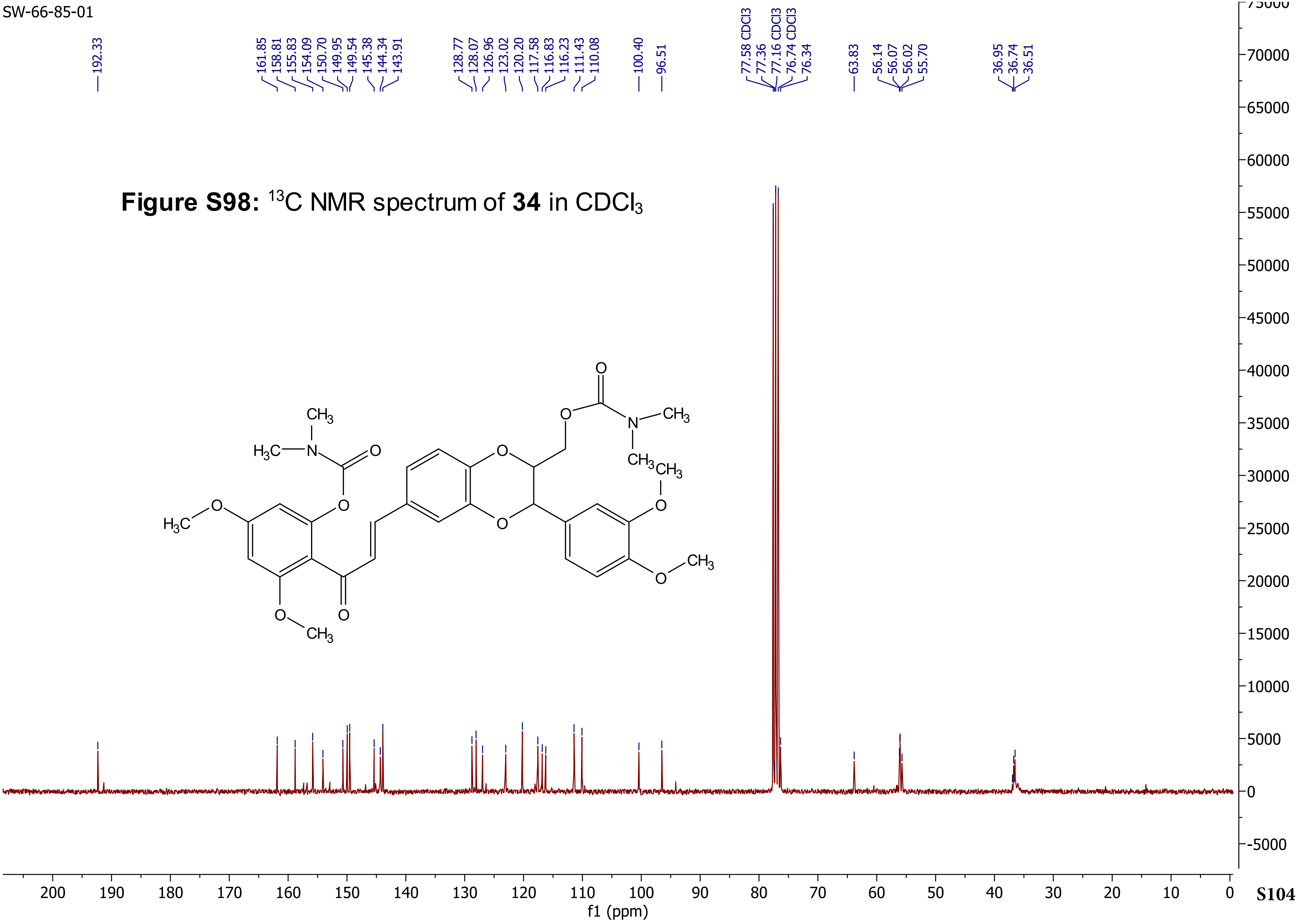

SW-66-132-02	C34H38N2O11	650.2476	651.2554	**651.2551**	-0.0003	-0.49

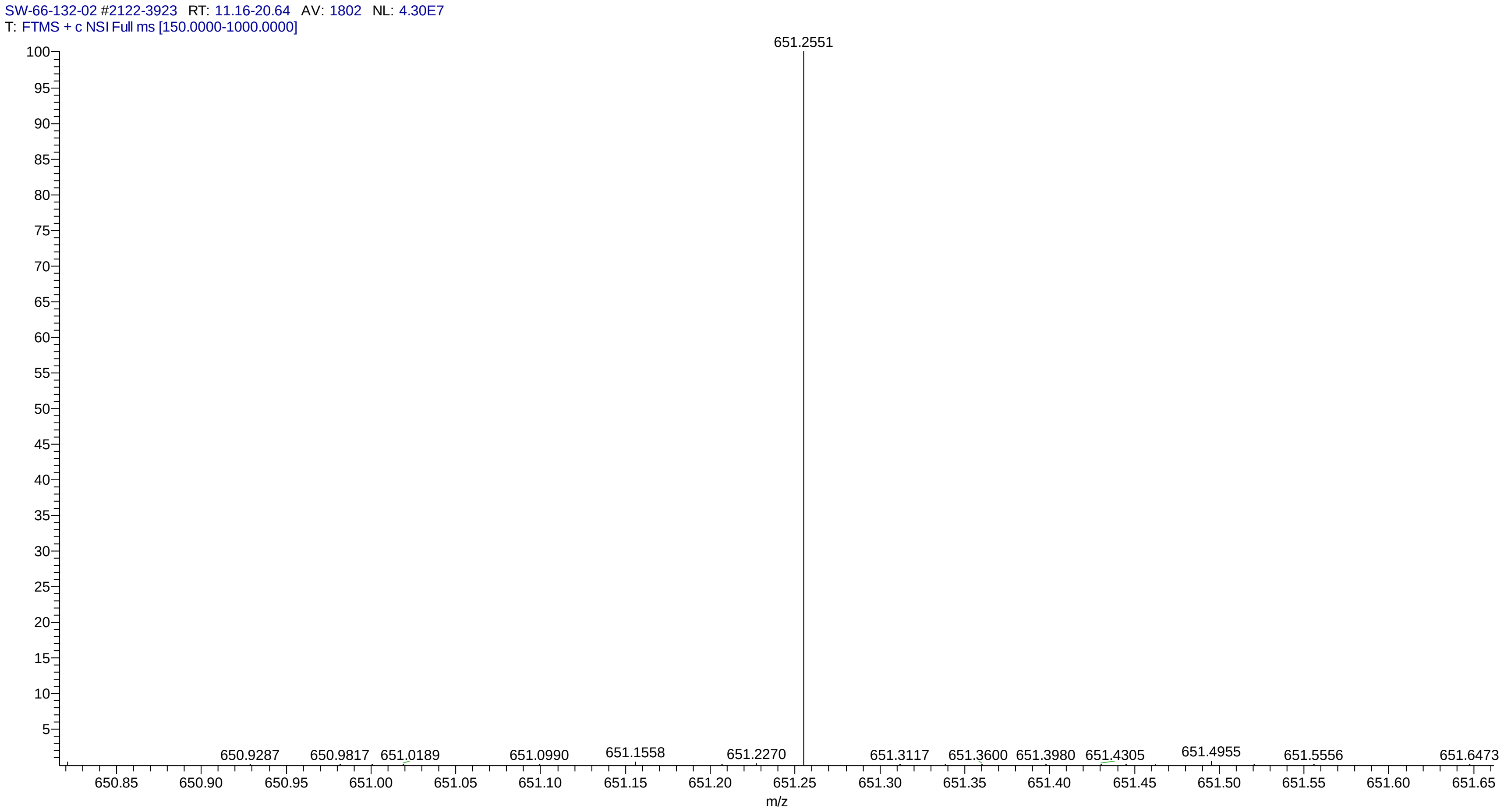

Figure S99: High resolution mass spectrum of **34**

===

Injection Date : 2/6/2023 1:47:08 PM
Sample Name : SW-66-140-03 Location : Vial 1
Acq. Operator :
Method : C:\HPCHEM\1\METHODS\JNP2015.M
Last changed : 1/31/2023 2:50:19 PM

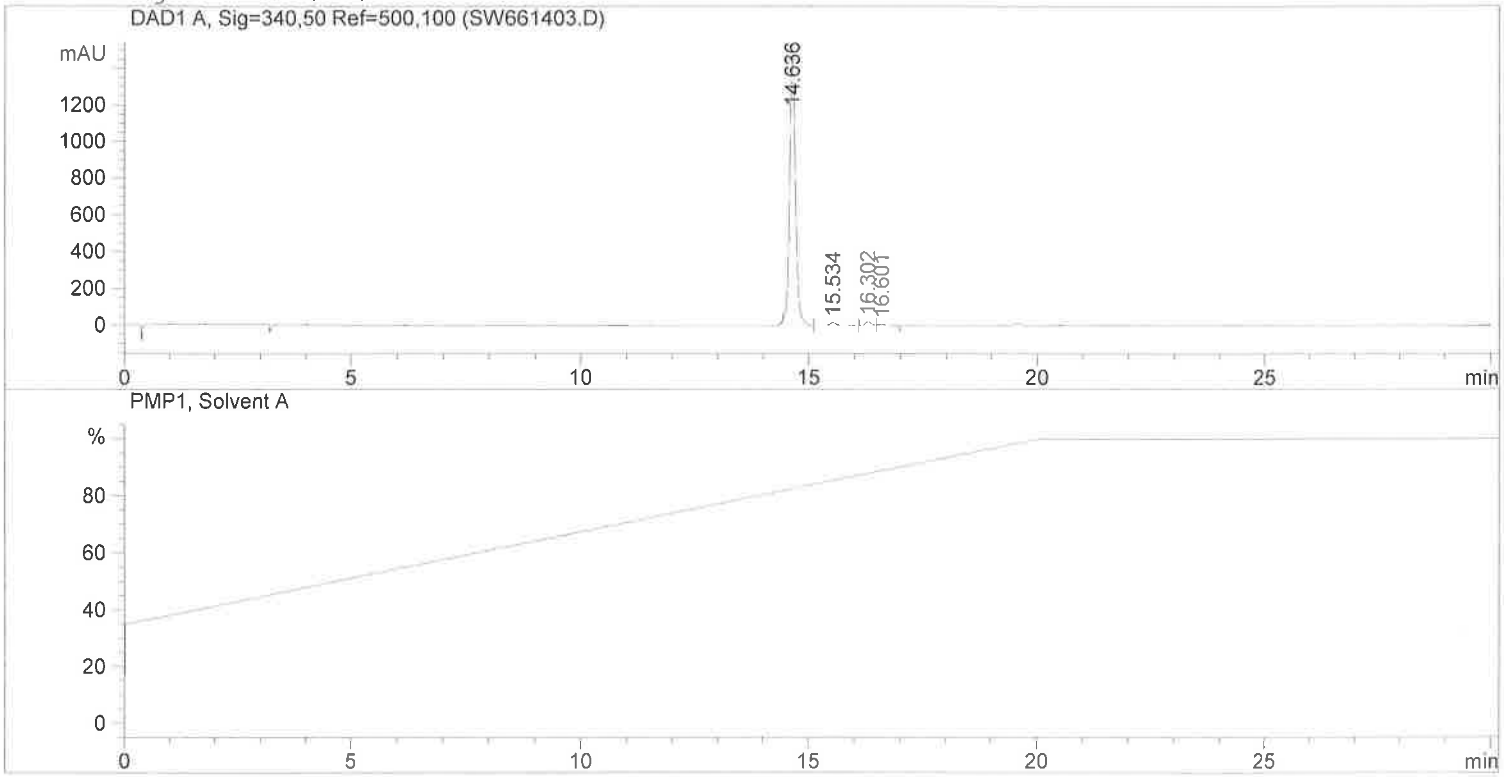

===

Area Percent Report

===

Sorted By : Signal
Multiplier : 1.0000
Dilution : 1.0000

Signal 1: DAD1 A, Sig=340,50 Ref=500,100

Peak #	RetTime [min]	Type	Width [min]	Area [mAU*s]	Height [mAU]	Area %
1	14.636	BV	0.1380	1.32796e4	1462.15613	96.9989
2	15.534	VP	0.1466	165.07674	16.81067	1.2058
3	16.302	VV	0.1337	181.16257	20.77712	1.3233
4	16.601	VP	0.1455	64.62059	6.64403	0.4720

Totals : 1.36904e4 1506.38795

Results obtained with enhanced integrator!

===

*** End of Report ***

Figure S100: HPLC chromatogram of **34**

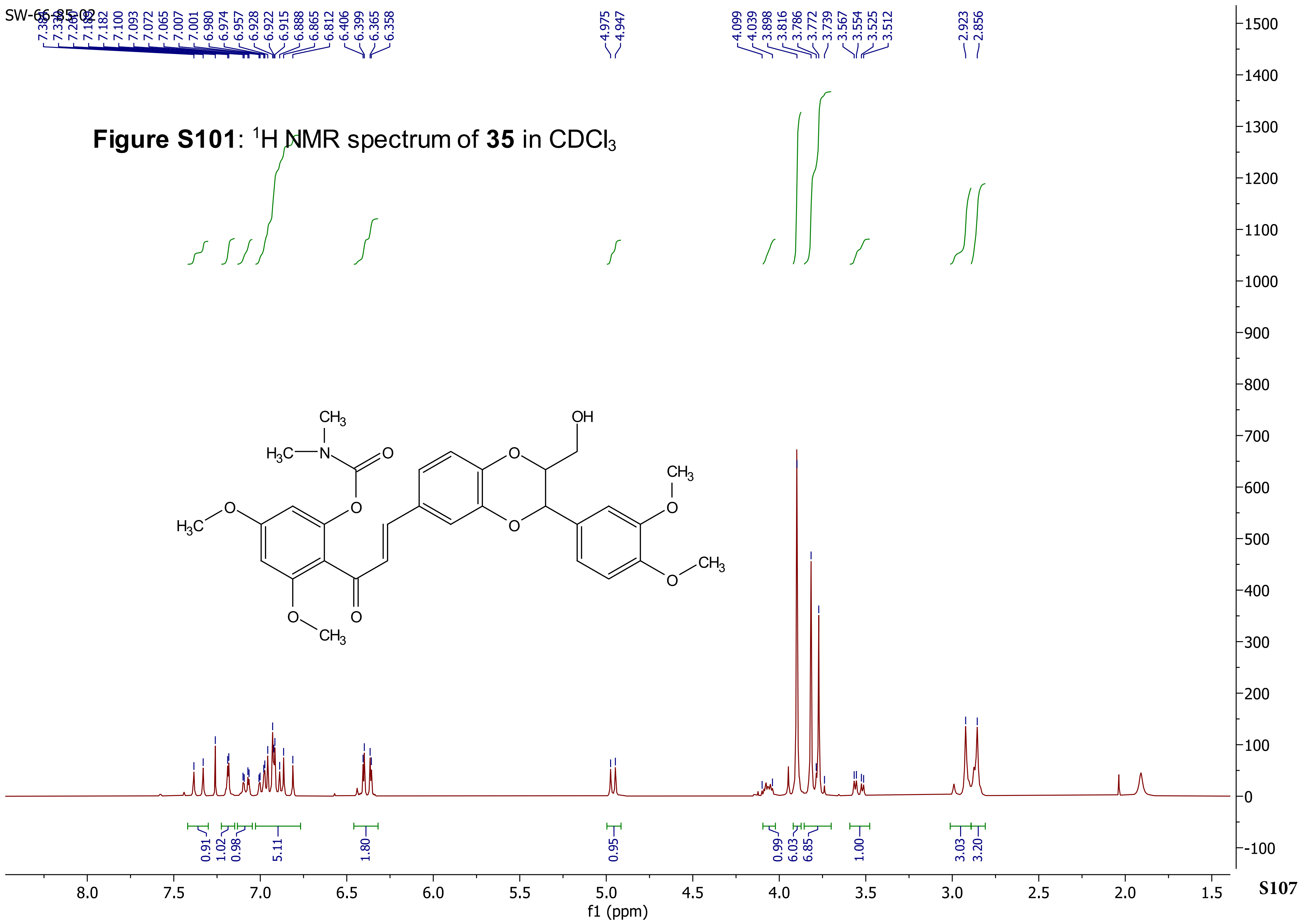

Figure S101: ^1H NMR spectrum of **35** in $CDCl_3$

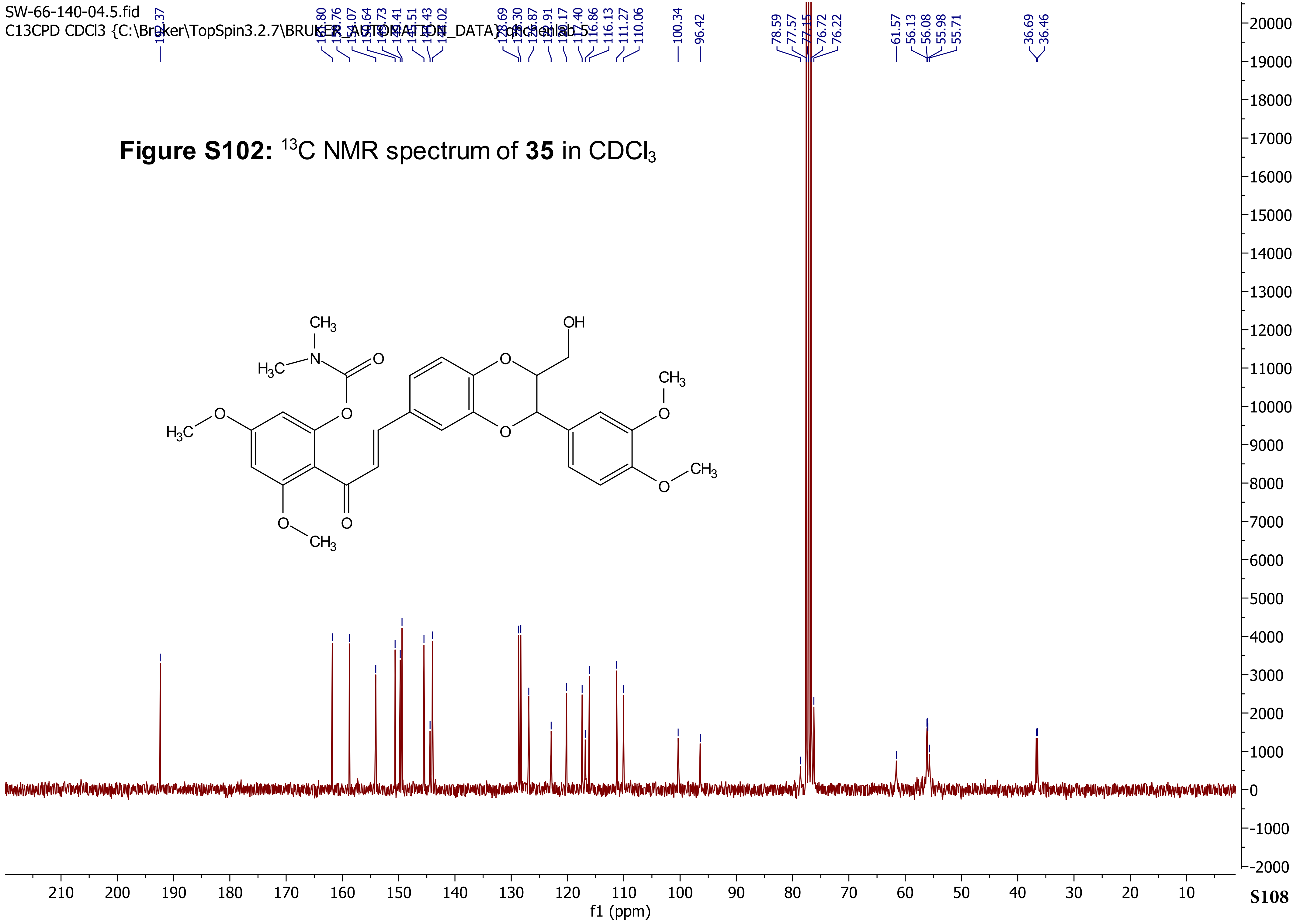

Figure S102: ^{13}C NMR spectrum of **35** in $CDCl_3$

SW-66-140-04	C31H33NO10	579.2105	580.2183	**580.2180**	-0.0003	-0.50

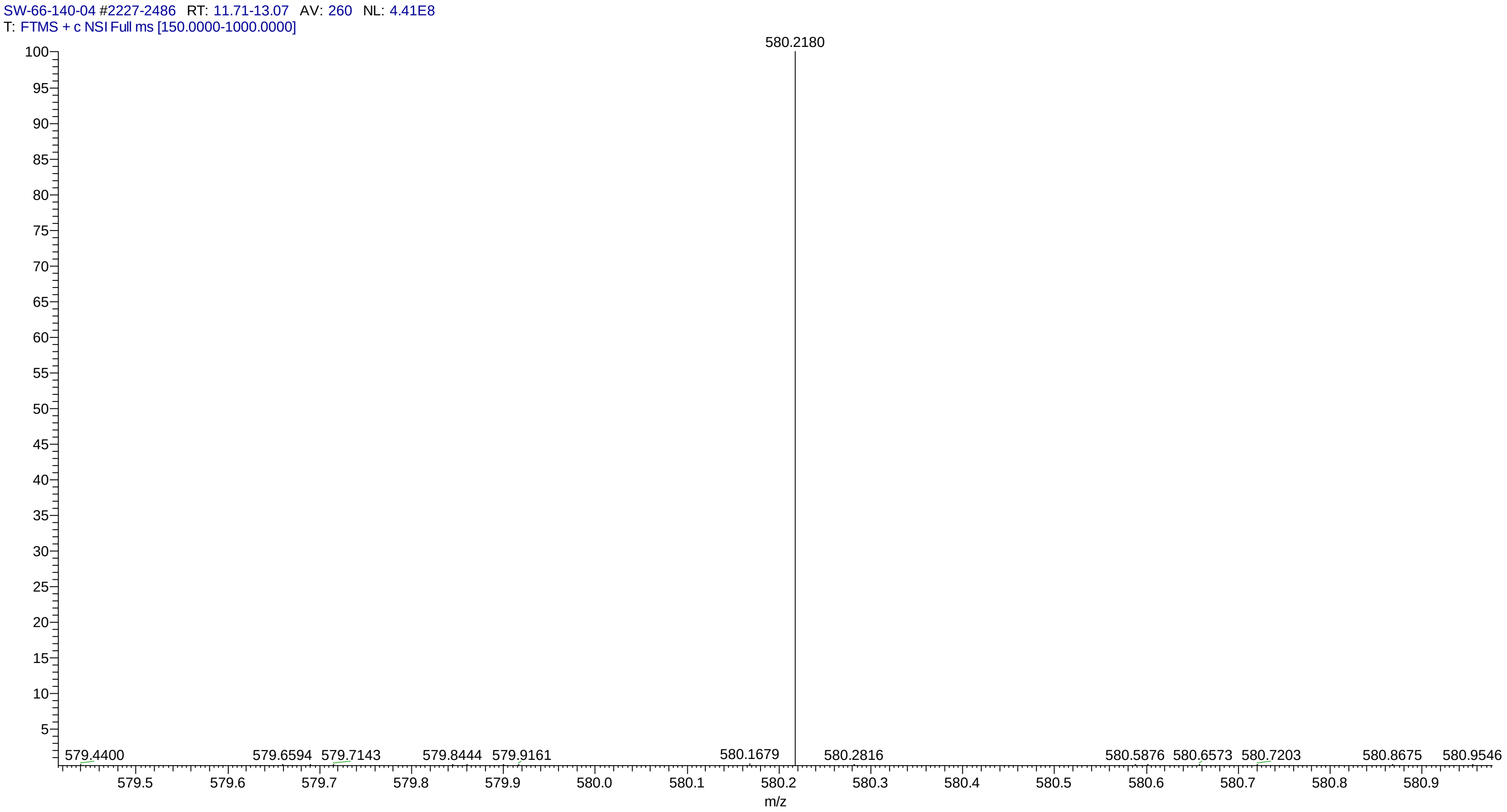

Figure S103: High resolution mass spectrum of **35**

===

Injection Date : 2/6/2023 3:19:26 PM
Sample Name : SW-66-140-04 Location : Vial 1
Acq. Operator :
Method : C:\HPCHEM\1\METHODS\JNP2015.M
Last changed : 1/31/2023 2:50:19 PM

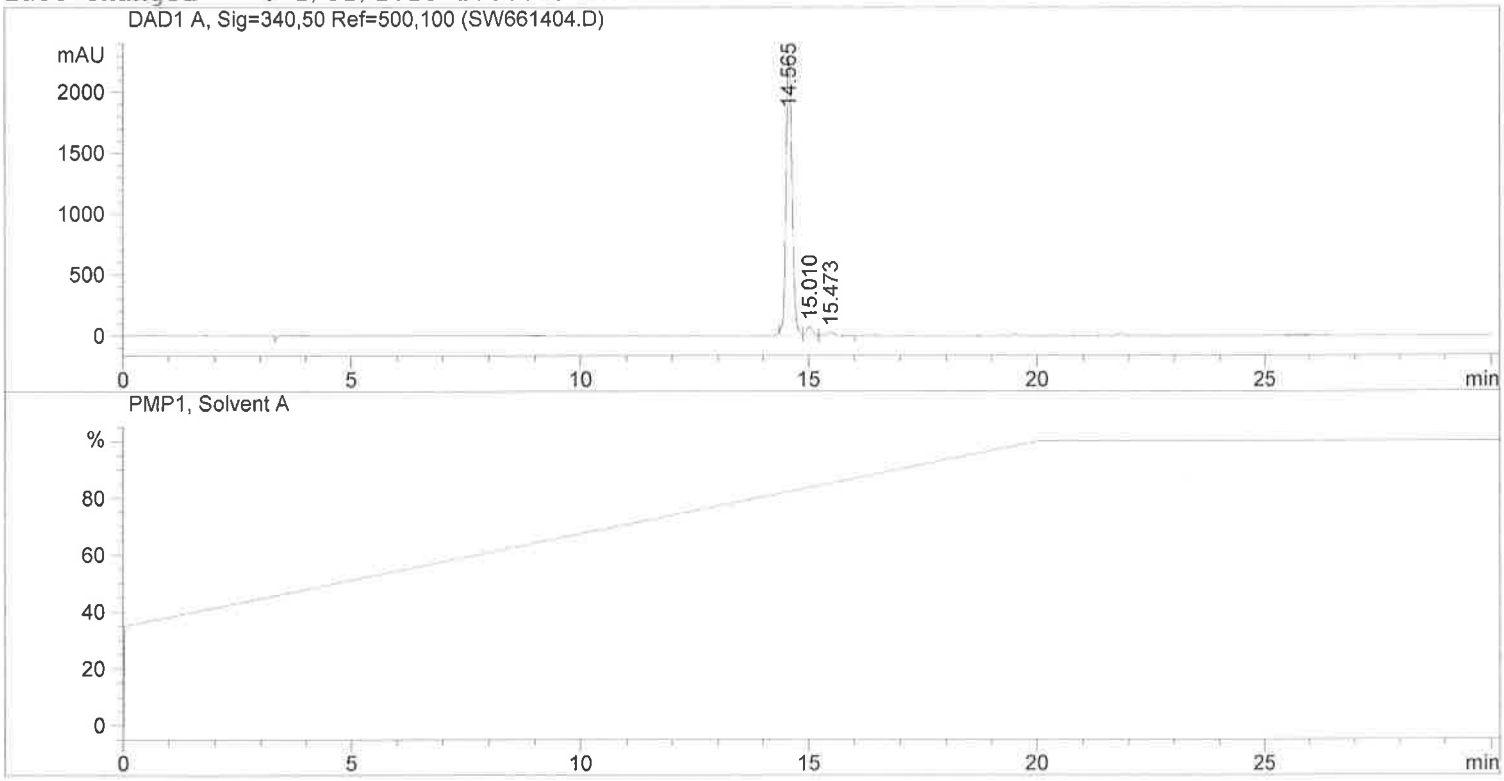

===

Area Percent Report

===

Sorted By : Signal
Multiplier : 1.0000
Dilution : 1.0000

Signal 1: DAD1 A, Sig=340,50 Ref=500,100

Peak #	RetTime [min]	Type	Width [min]	Area [mAU*s]	Height [mAU]	Area %
1	14.565	BV	0.1440	2.09682e4	2264.71973	95.4822
2	15.010	VV	0.1339	667.68225	76.45083	3.0404
3	15.473	VP	0.1675	324.44379	27.94491	1.4774

Totals : 2.19603e4 2369.11546

Results obtained with enhanced integrator!

===

*** End of Report ***

Figure S104: HPLC chromatogram of **35**

SW-85-111-02.1.fid
5,7,20-Trimethylsilybin A

Figure S105: ^{1}H NMR spectrum of **5A** in $CDCl_3$

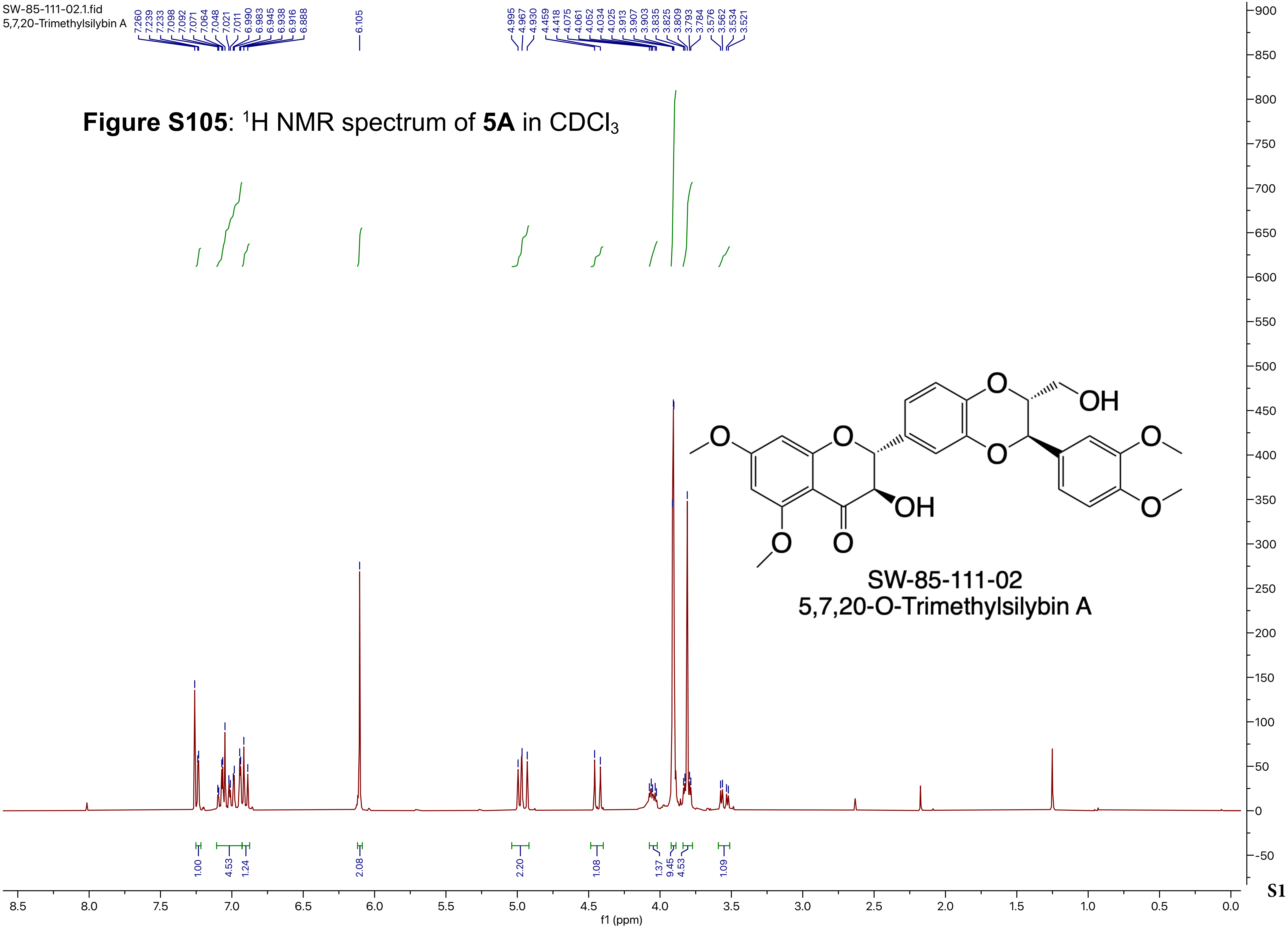

SW-85-111-02
5,7,20-O-Trimethylsilybin A

Figure S106: ^{13}C NMR spectrum of **5A** in $CDCl_3$

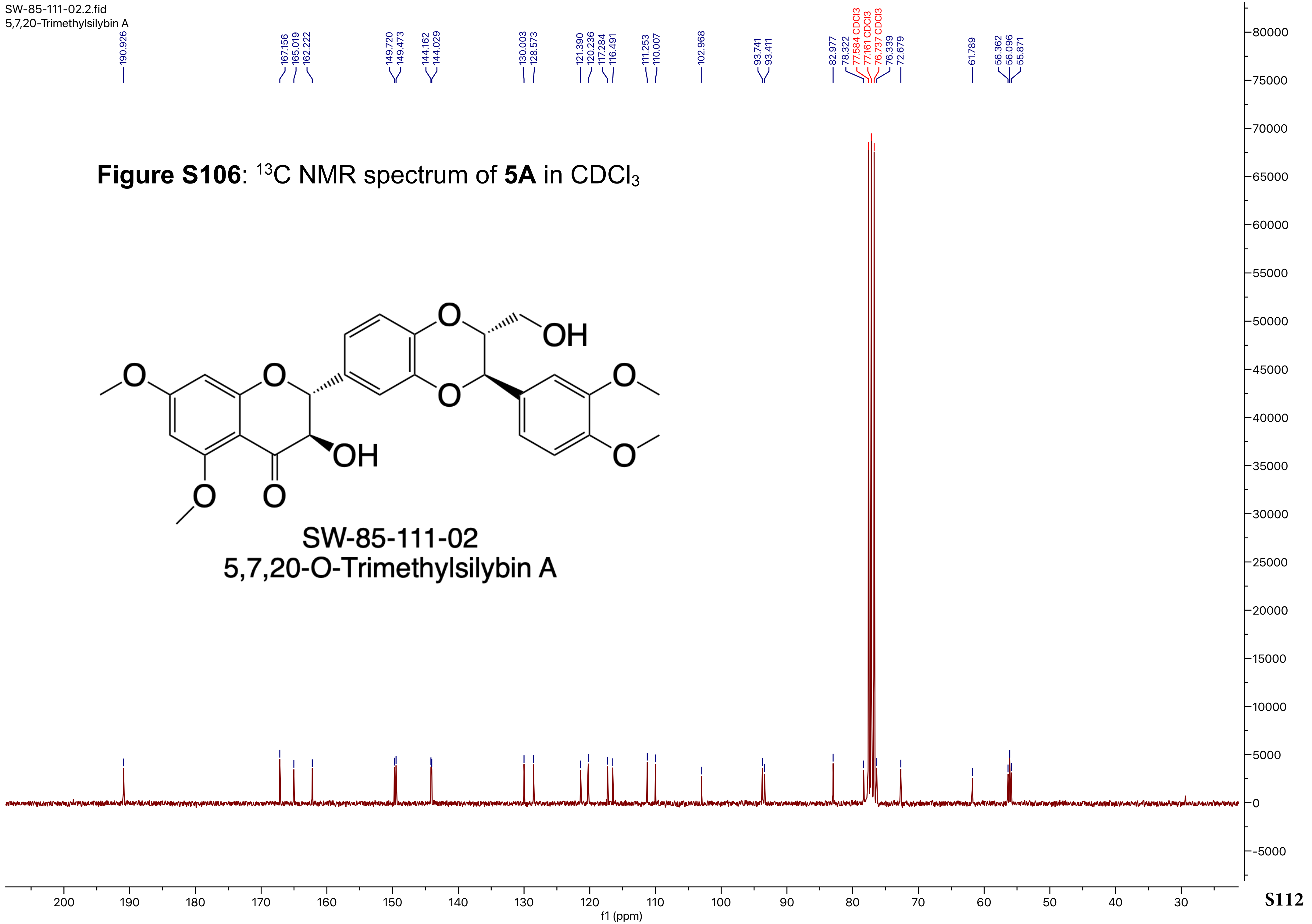

Sample Name	Mol Fomula	MW	M+H	observed	delta	ppm
SW-85-111-02	C28H28O10	524.1683	525.1761	525.1761	0.0000	0.00

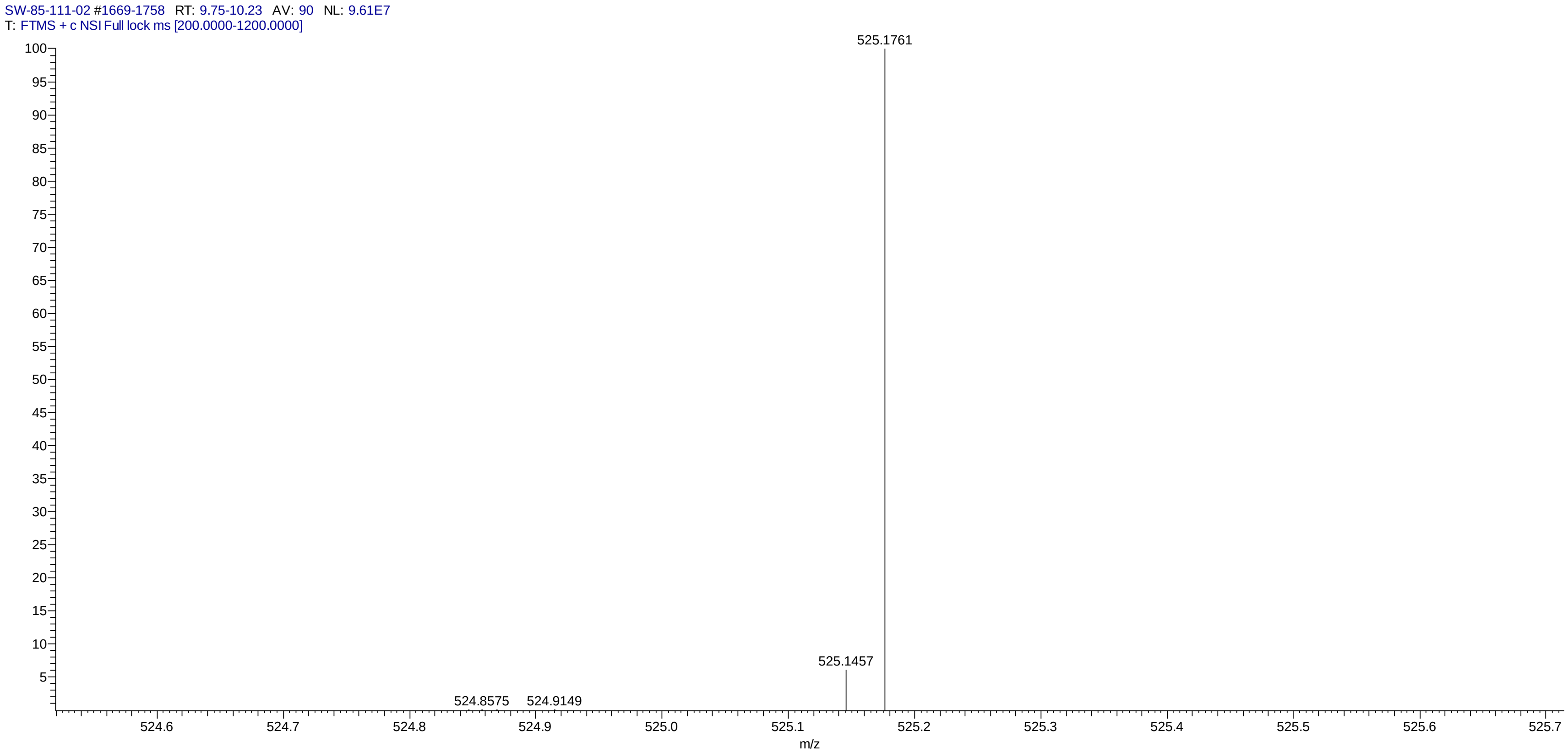

Figure S107: High resolution mass spectrum of **5A**

```
=====================================================================
Injection Date  : 4/20/2022 1:56:26 PM
Sample Name     : SW-85-126                    Location : Vial 1
Acq. Operator   :
Method          : C:\HPCHEM\1\METHODS\JNP2015.M
Last changed    : 4/19/2022 12:01:10 PM
                  (modified after loading)
```

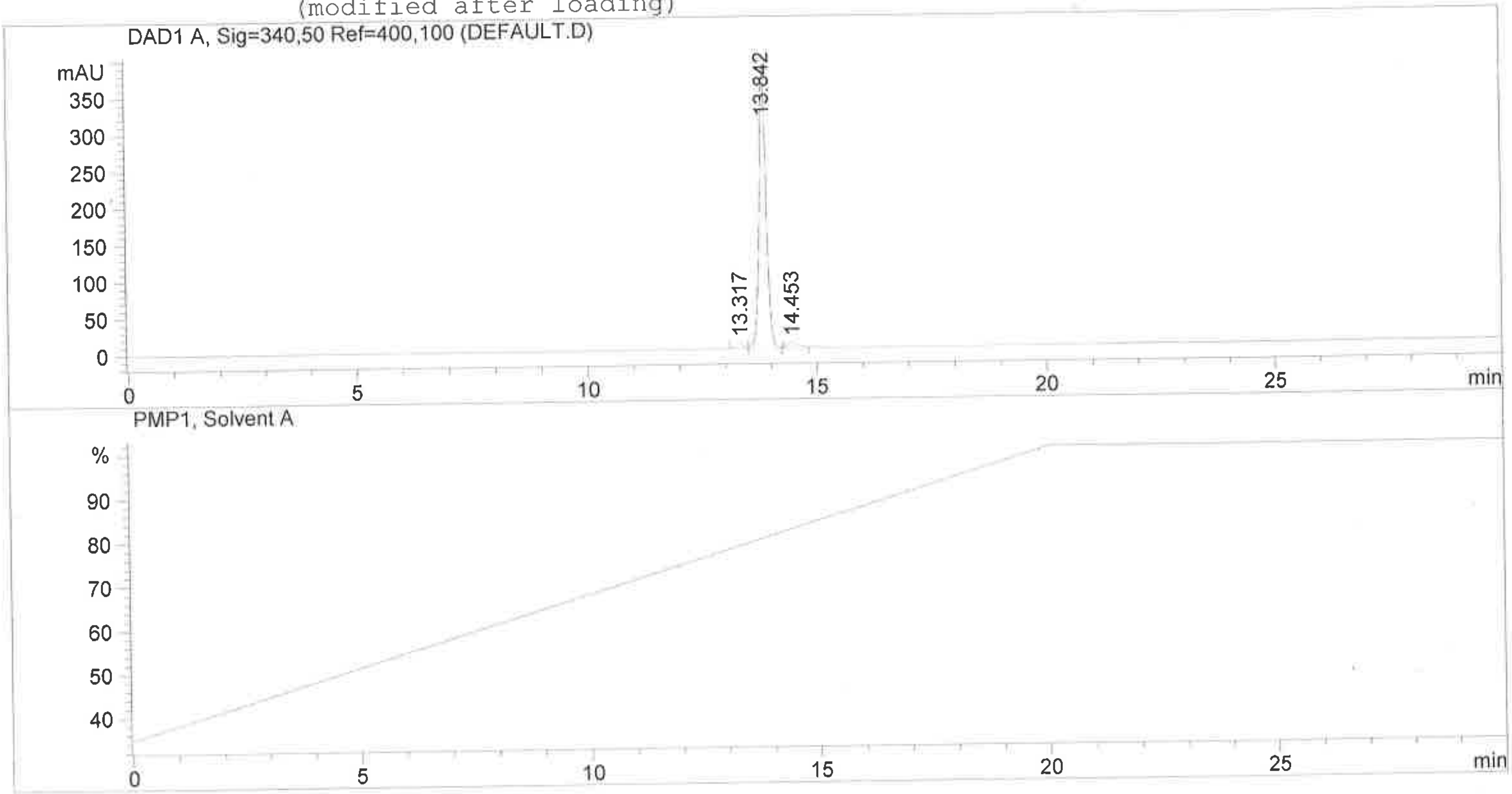


```
=====================================================================
                       Area Percent Report
=====================================================================

Sorted By             :      Signal
Multiplier            :      1.0000
Dilution              :      1.0000

Signal 1: DAD1 A, Sig=340,50 Ref=400,100
```

Peak #	RetTime [min]	Type	Width [min]	Area [mAU*s]	Height [mAU]	Area %
1	13.317	BV	0.1537	85.29691	8.31321	1.9459
2	13.842	VB	0.1607	4179.83057	384.95459	95.3551
3	14.453	BB	0.2026	118.30874	8.18441	2.6990
Totals :				4383.43621	401.45221	

```
 Results obtained with enhanced integrator!
=====================================================================
                         *** End of Report ***
```

Figure S108: HPLC chromatogram of **5A**

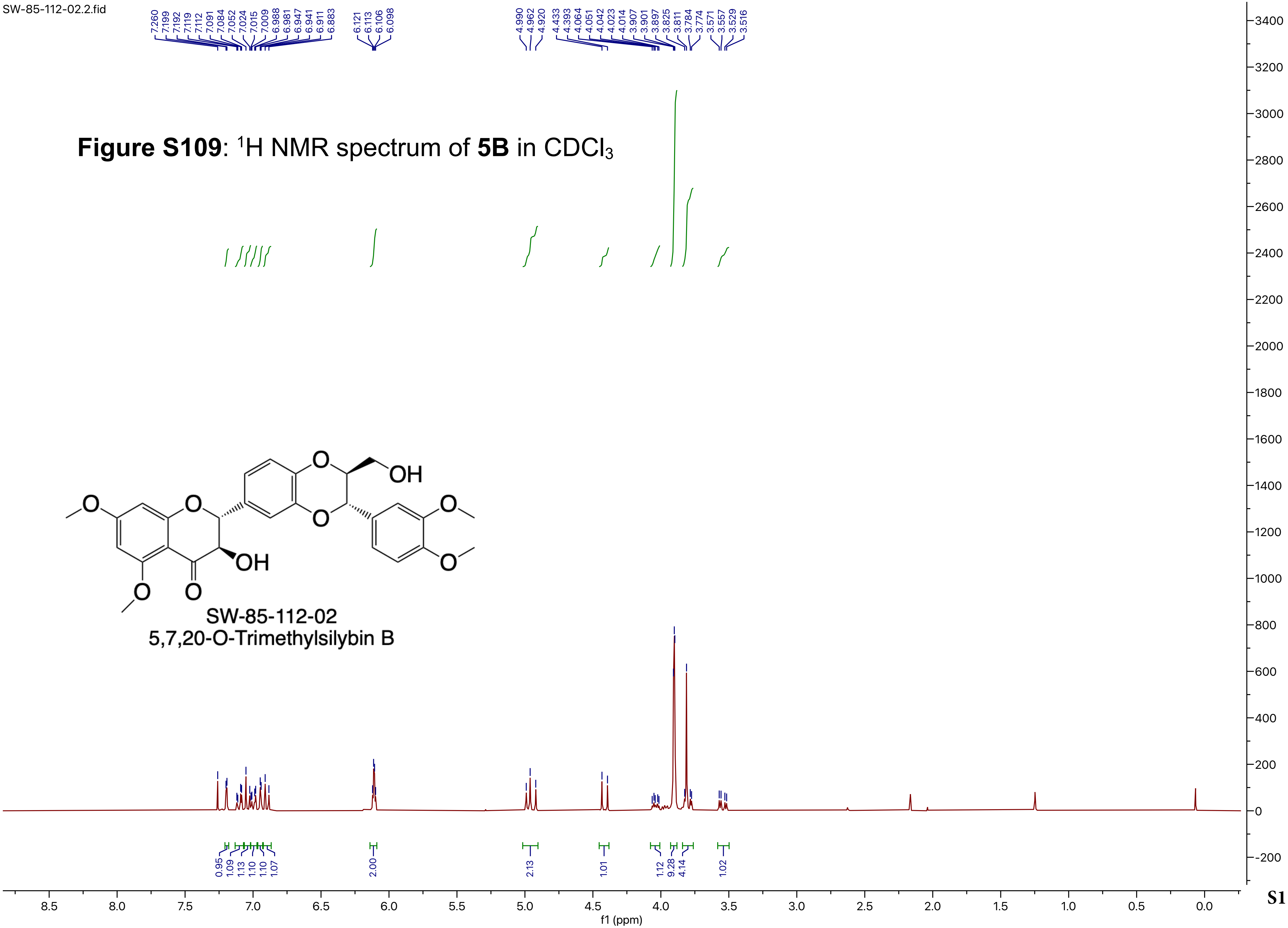

Figure S109: ^{1}H NMR spectrum of **5B** in $CDCl_3$

SW-85-112-02
5,7,20-O-Trimethylsilybin B

Figure S110: ^{13}C NMR spectrum of **5B** in $CDCl_3$

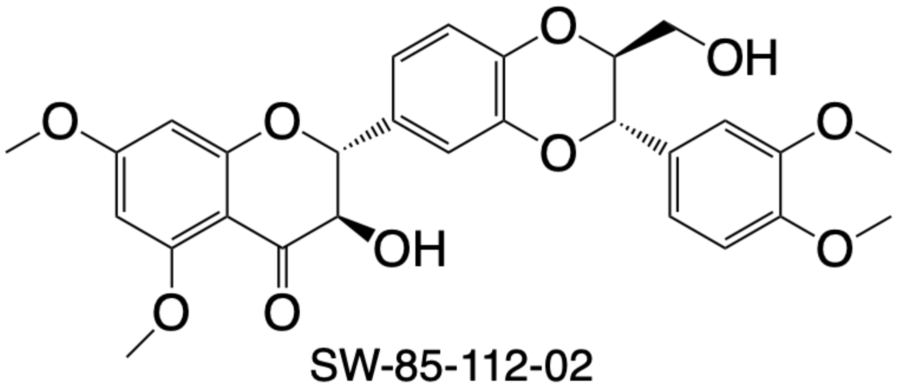

SW-85-112-02
5,7,20-O-Trimethylsilybin B

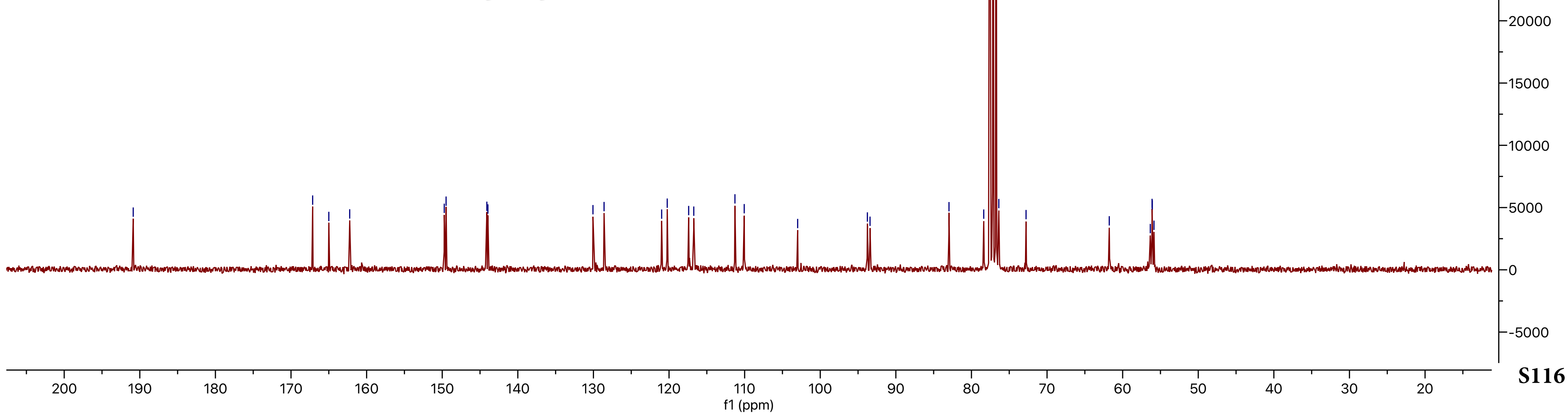

Sample Name	Mol Fomula	MW	M+H	observed	delta	ppm
SW-85-112-02	C28H28O10	524.1683	525.1761	525.1763	0.0002	0.38

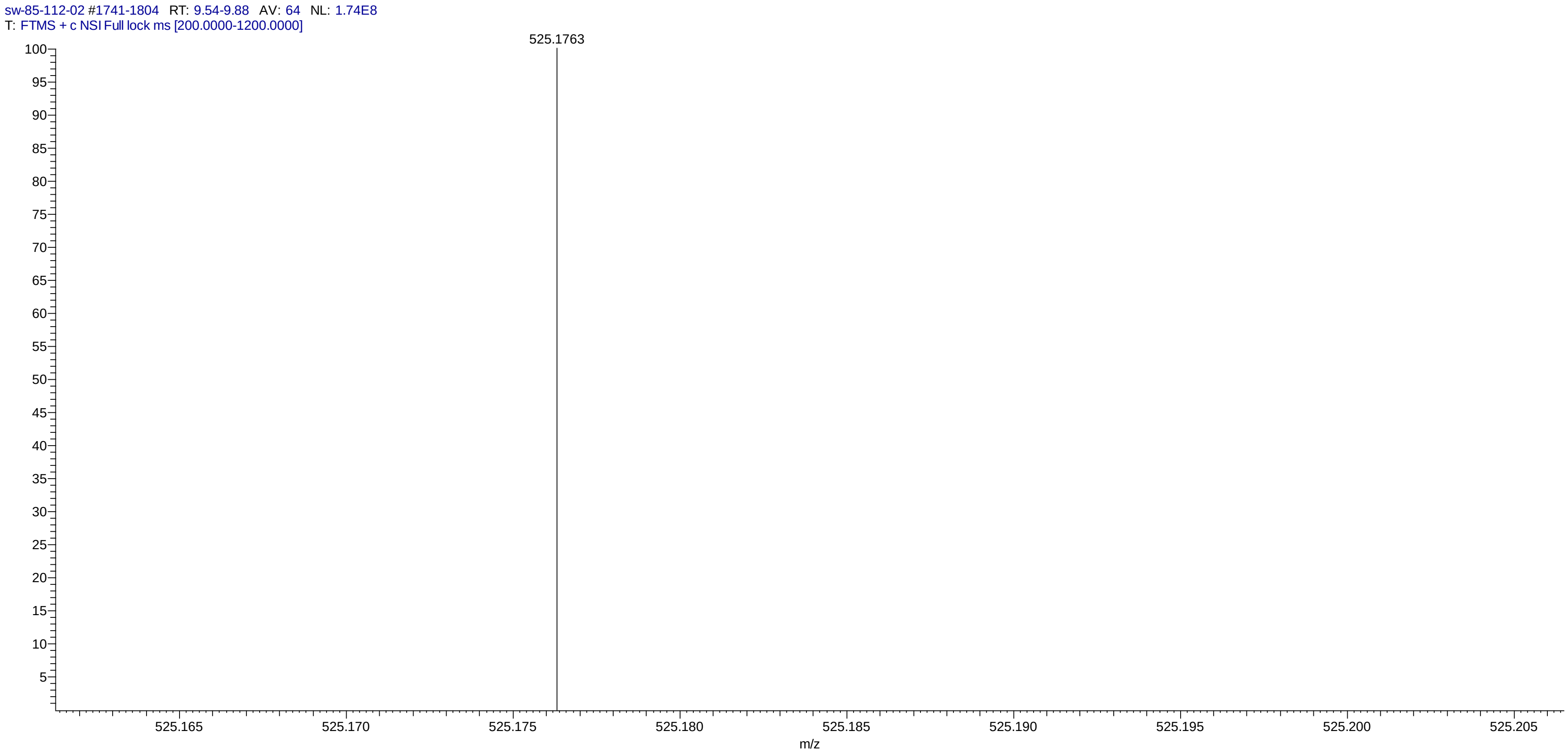

Figure S111: High resolution mass spectrum of **5B**

===

Injection Date : 4/20/2022 1:09:49 PM
Sample Name : SW-85-112-02 Location : Vial 1
Acq. Operator :
Method : C:\HPCHEM\1\METHODS\JNP2015.M
Last changed : 4/19/2022 12:01:10 PM
(modified after loading)

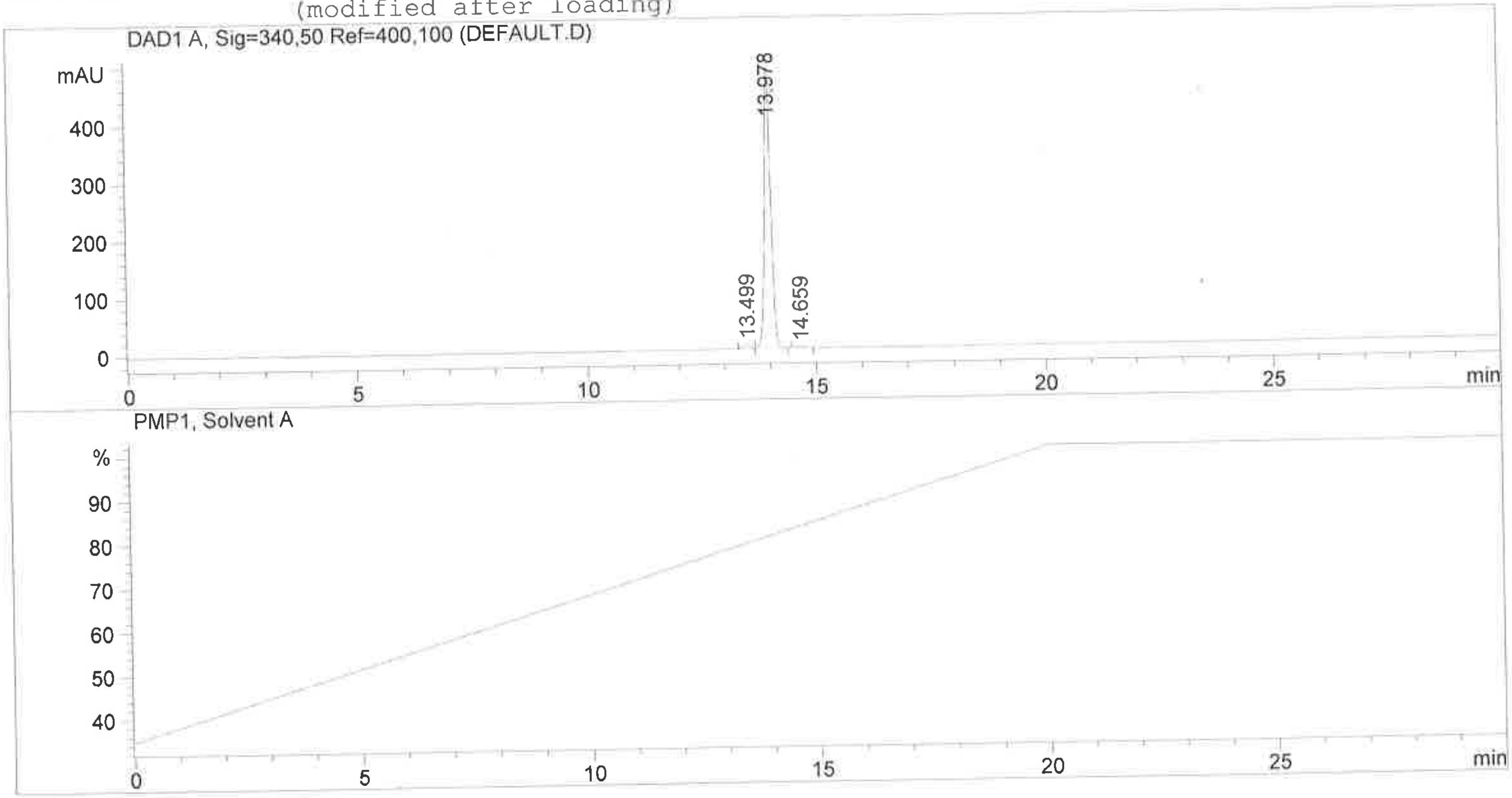

===

Area Percent Report

===

Sorted By : Signal
Multiplier : 1.0000
Dilution : 1.0000

Signal 1: DAD1 A, Sig=340,50 Ref=400,100

Peak #	RetTime [min]	Type	Width [min]	Area [mAU*s]	Height [mAU]	Area %
1	13.499	BV	0.1390	74.58830	8.13352	1.5132
2	13.978	VB	0.1488	4824.36230	490.18085	97.8718
3	14.659	BB	0.1732	30.31663	2.65632	0.6150
Totals :				4929.26724	500.97069	

Results obtained with enhanced integrator!

===

*** End of Report ***

Figures S112: HPLC chromatogram of **5B**

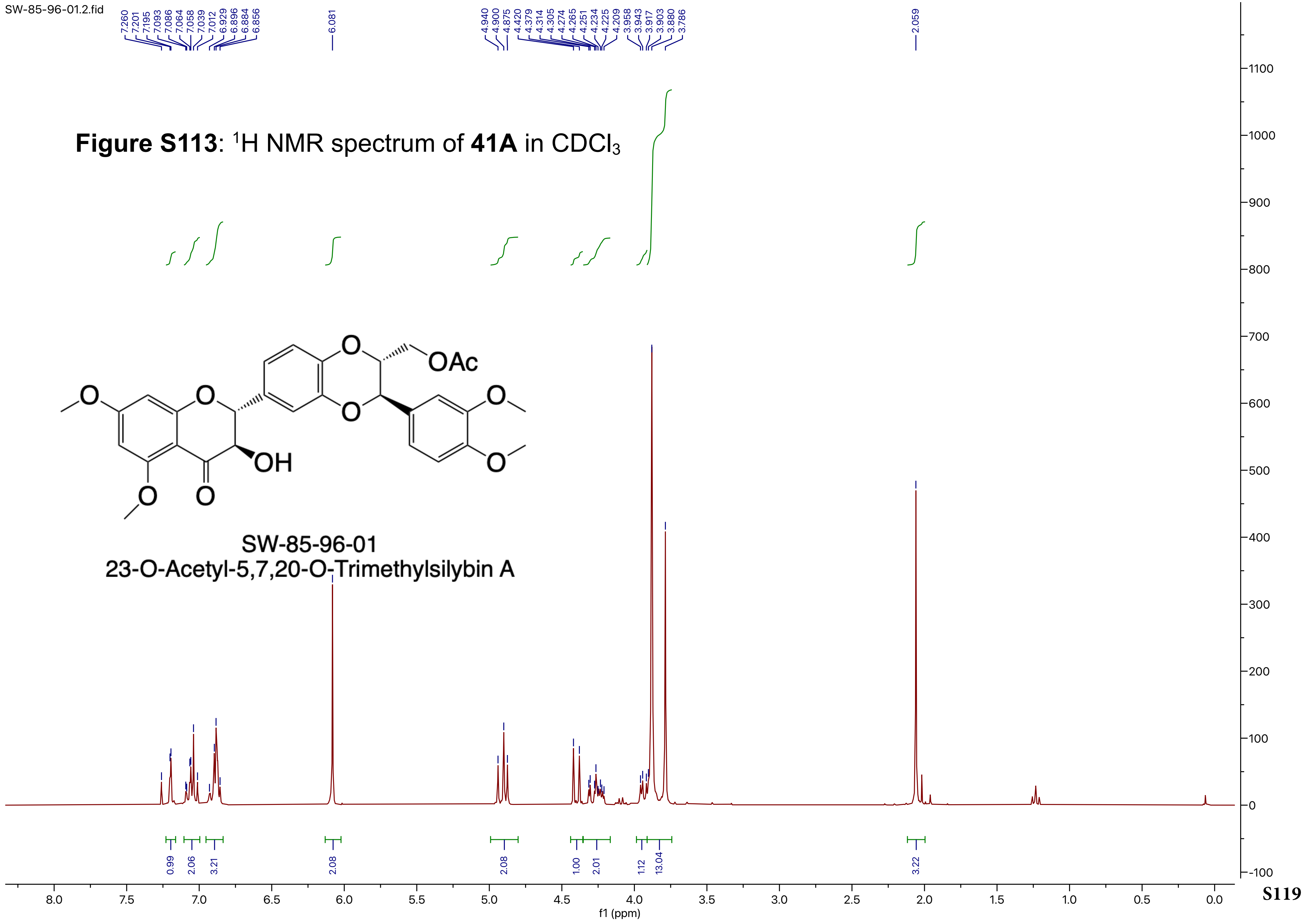

Figure S113: ^{1}H NMR spectrum of **41A** in $CDCl_3$

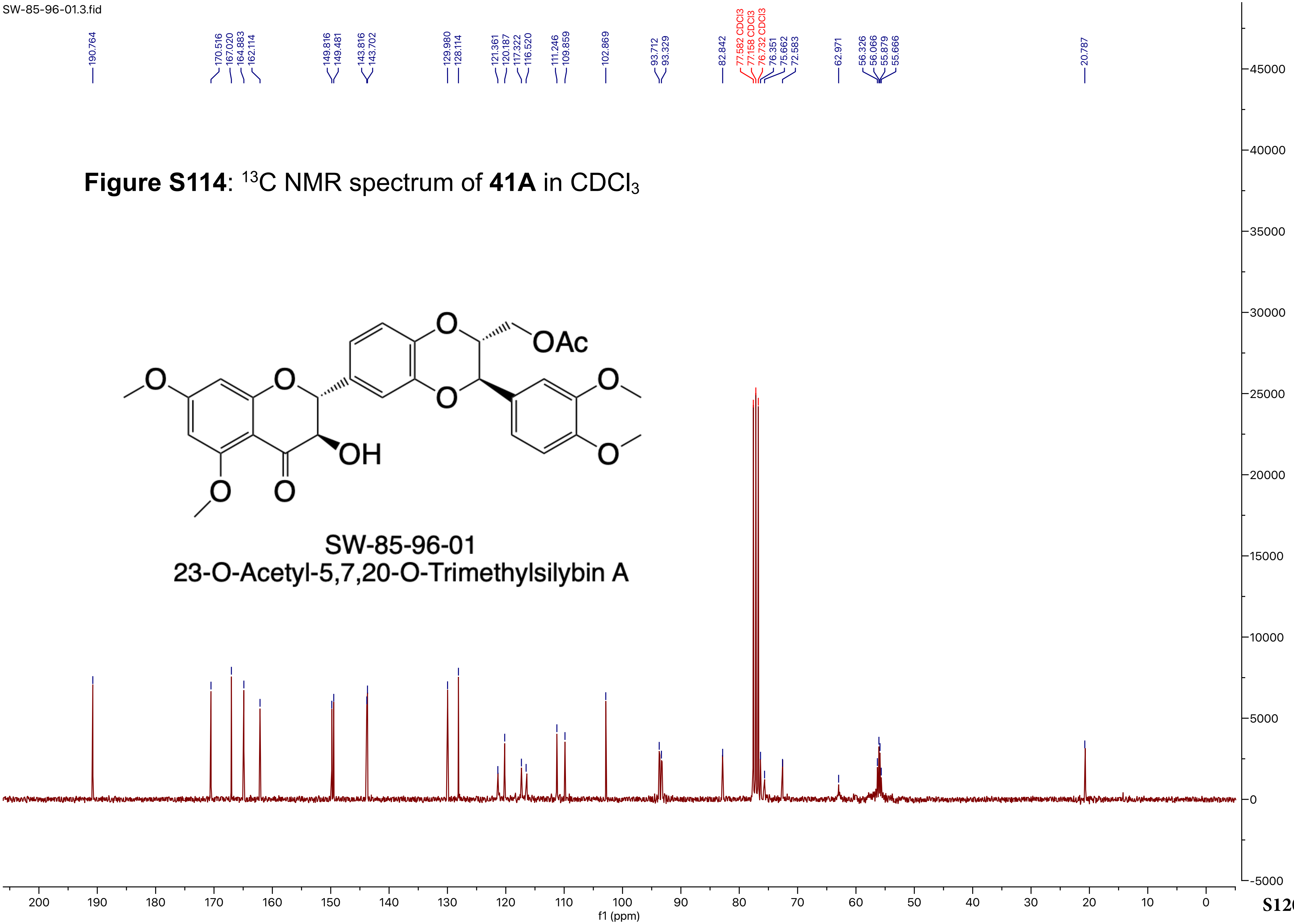

Figure S114: ^{13}C NMR spectrum of **41A** in $CDCl_3$

Sample Name	Mol Fomula	MW	M+H	observed	delta	ppm
SW-85-96-01	C30H30O11	566.1789	567.1867	567.1869	0.0002	0.41

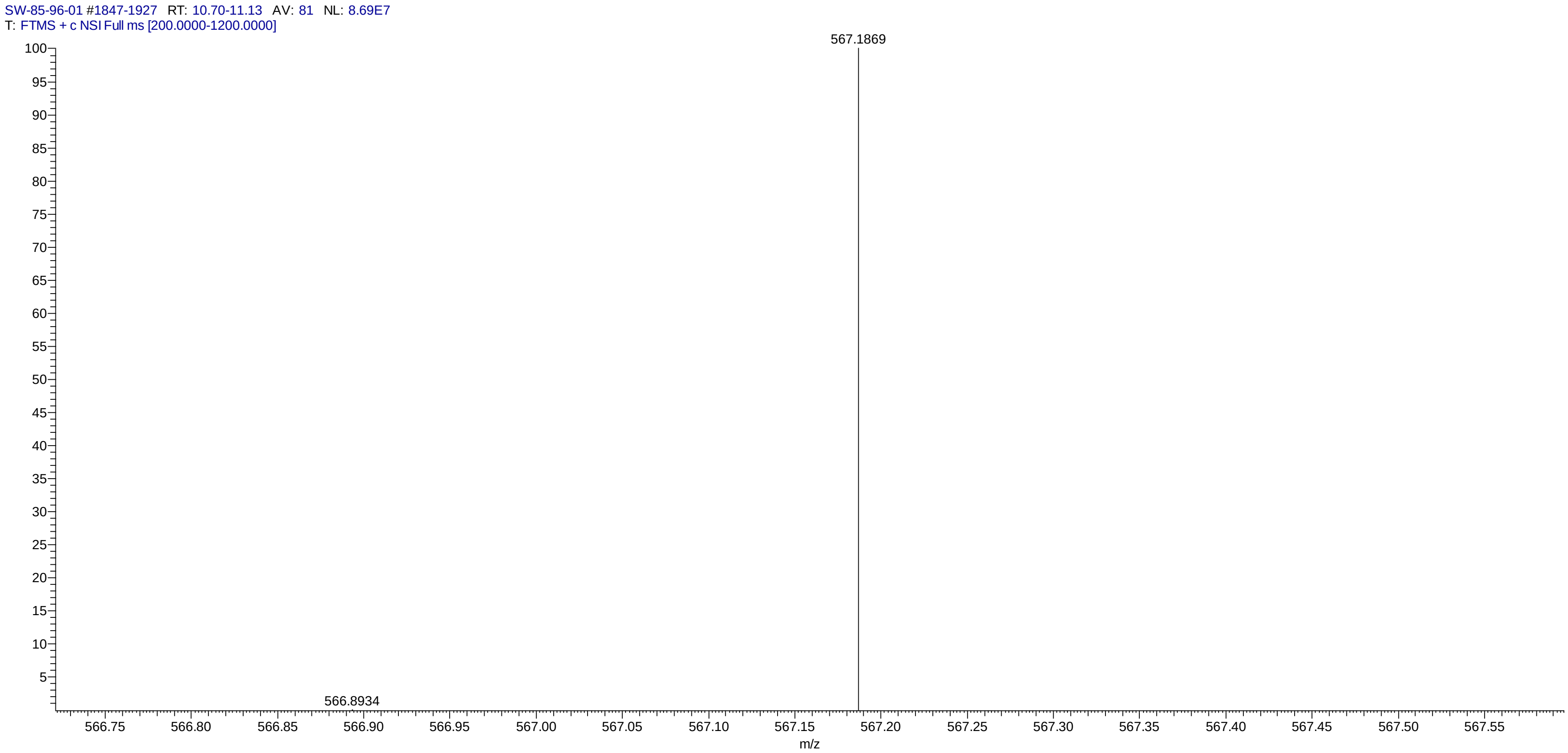

Figure S115: High resolution mass spectrum of **41A**

```
=====================================================================
Injection Date  : 5/1/2022 2:04:23 PM
Sample Name     : SW-85-96-01                    Location : Vial 1
Acq. Operator   :
Method          : C:\HPCHEM\1\METHODS\JNP2015.M
Last changed    : 4/30/2022 3:37:52 PM
                  (modified after loading)
```

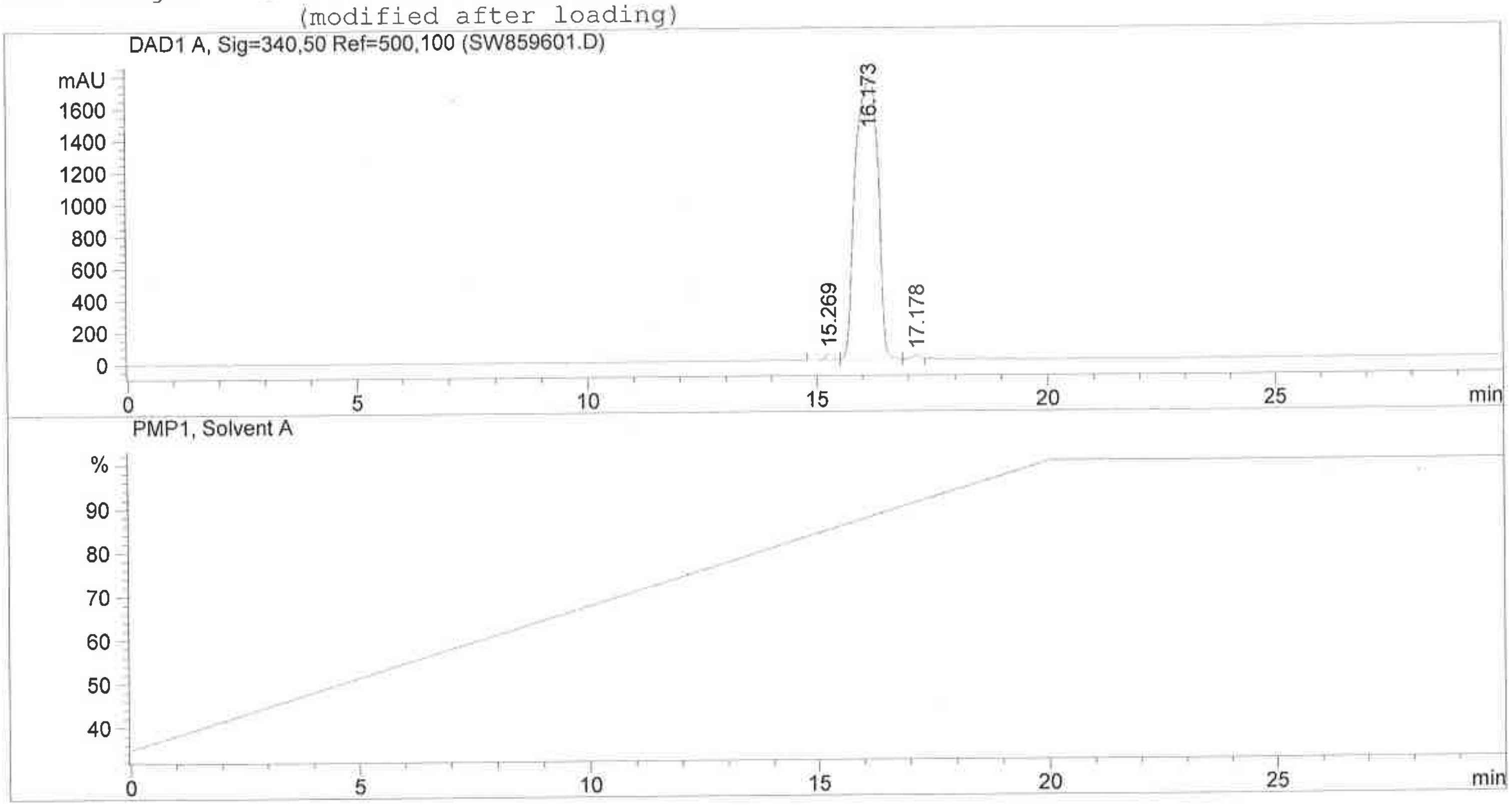


```
=====================================================================
                        Area Percent Report
=====================================================================

Sorted By             :      Signal
Multiplier            :      1.0000
Dilution              :      1.0000

Signal 1: DAD1 A, Sig=340,50 Ref=500,100
```

Peak #	RetTime [min]	Type	Width [min]	Area [mAU*s]	Height [mAU]	Area %
1	15.269	PV	0.1803	491.90530	40.90677	0.8050
2	16.173	VV	0.5799	6.01970e4	1773.94360	98.5084
3	17.178	VV	0.2139	419.57355	28.81751	0.6866

Totals : 6.11085e4 1843.66789

```
Results obtained with enhanced integrator!
=====================================================================
                        *** End of Report ***
```

Figure S116: HPLC chromatogram of **41A**

Figure S117: ^{1}H NMR spectrum of **41B** in $CDCl_3$

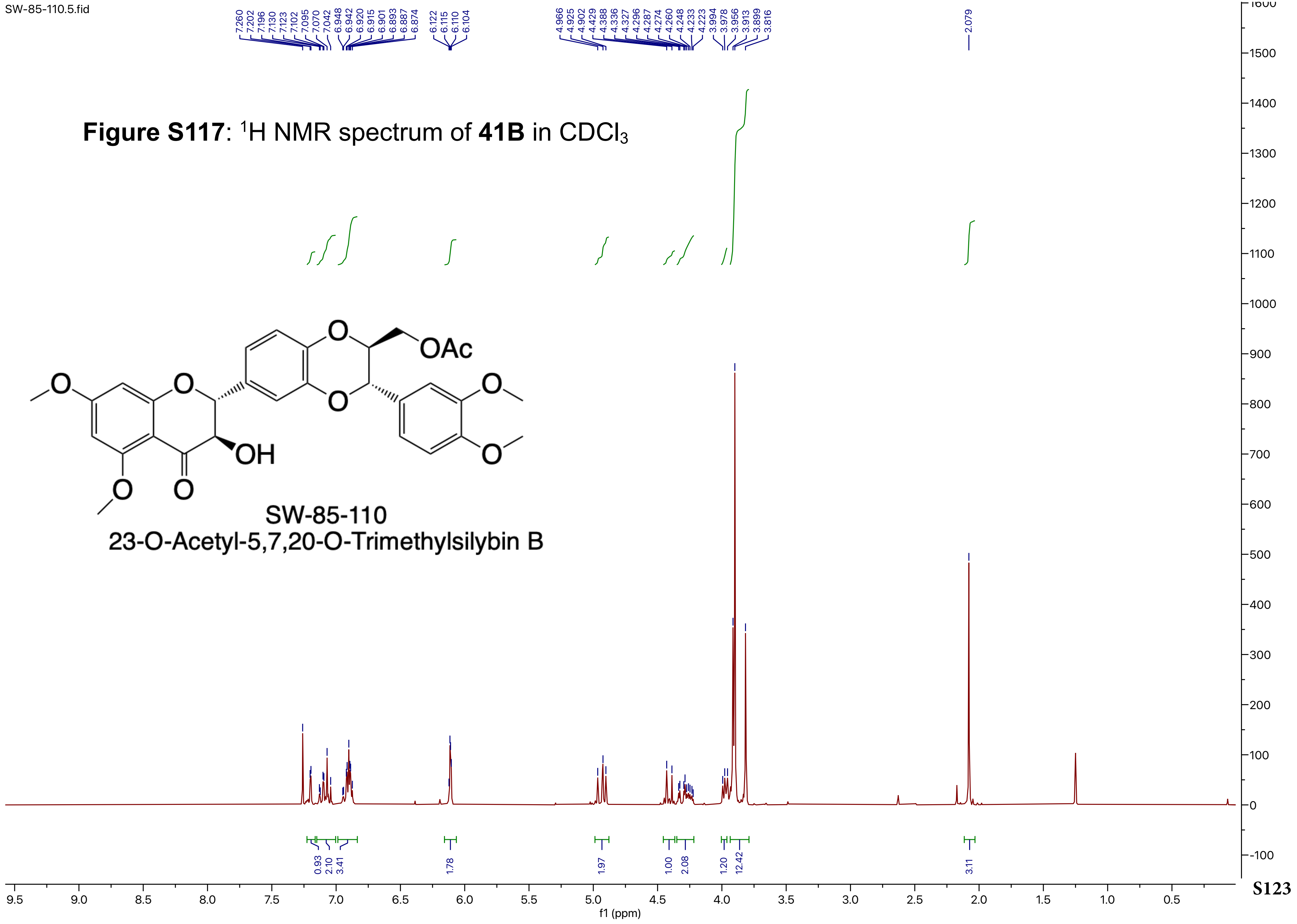

SW-85-110

23-O-Acetyl-5,7,20-O-Trimethylsilybin B

Figure S118: ^{13}C NMR spectrum of **41B** in $CDCl_3$

SW-85-110
23-O-Acetyl-5,7,20-O-Trimethylsilybin B

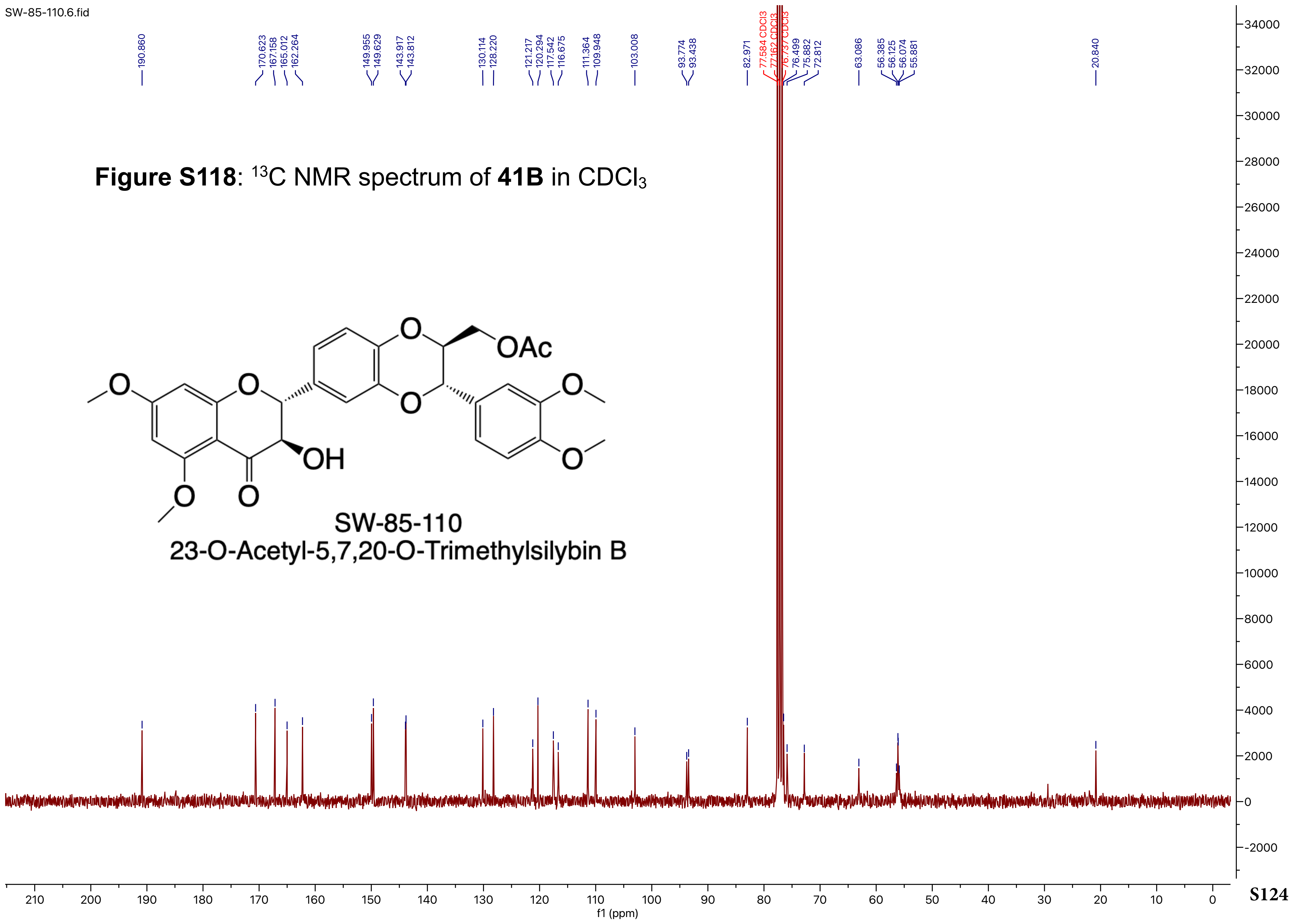

Sample Name	Mol Fomula	MW	M+H	observed	delta	ppm
SW-85-110	C30H30O11	566.1789	567.1867	567.1871	0.0004	0.76

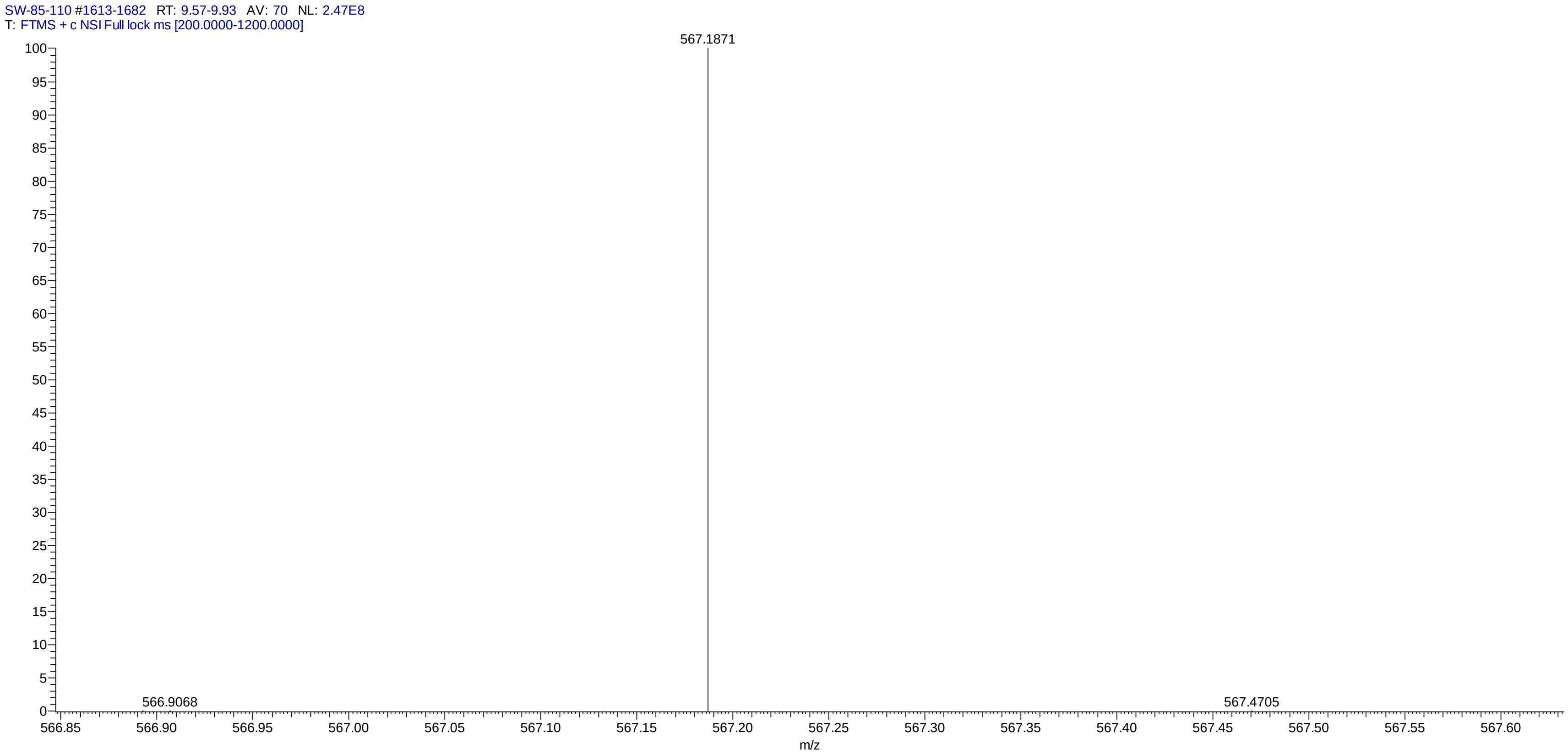

Figure S119: High resolution mass spectrum of **41B**

```
=====================================================================
Injection Date  : 5/1/2022 2:57:07 PM
Sample Name     : SW-85-110                     Location : Vial 1
Acq. Operator   :
Method          : C:\HPCHEM\1\METHODS\JNP2015.M
Last changed    : 4/30/2022 3:37:52 PM
                  (modified after loading)
```

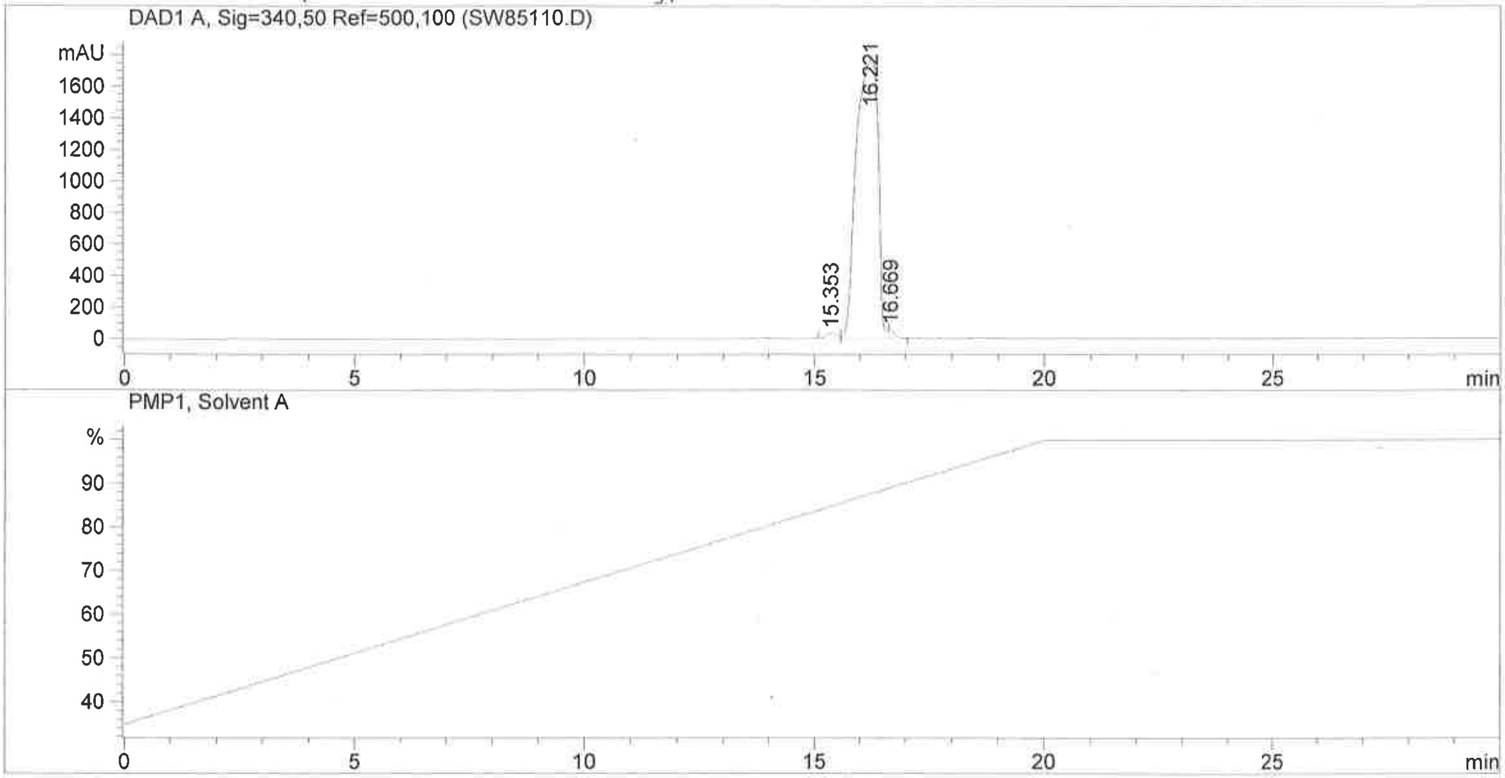


```
=====================================================================
                         Area Percent Report
=====================================================================

Sorted By             :      Signal
Multiplier            :      1.0000
Dilution              :      1.0000
```

Signal 1: DAD1 A, Sig=340,50 Ref=500,100

Peak #	RetTime [min]	Type	Width [min]	Area [mAU*s]	Height [mAU]	Area %
1	15.353	VV	0.2655	538.65302	31.18570	0.9020
2	16.221	VV	0.5660	5.86821e4	1792.36096	98.2707
3	16.669	VV	0.1432	494.01041	51.81585	0.8273

Totals : 5.97147e4 1875.36251

Results obtained with enhanced integrator!

```
=====================================================================
                         *** End of Report ***
```

Figure S120: HPLC chromatogram of **41B**

Figure S121: ^{1}H NMR spectrum of **10A** in $CDCl_3$

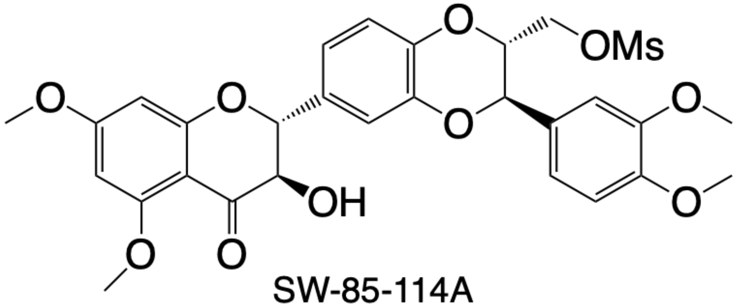

SW-85-114A
23-O-Mesylate-5,7,20-O-Trimethylsilybin A

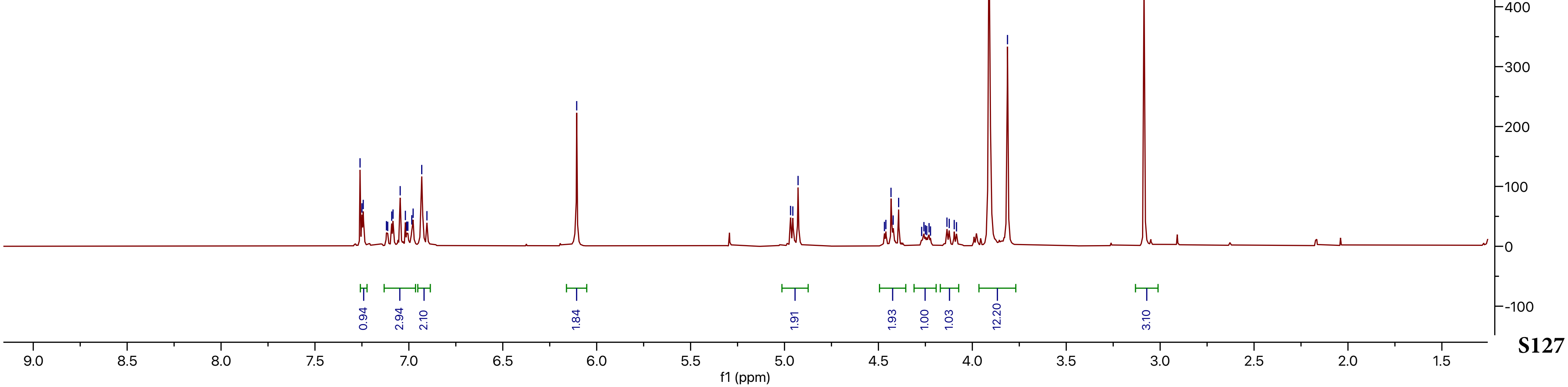

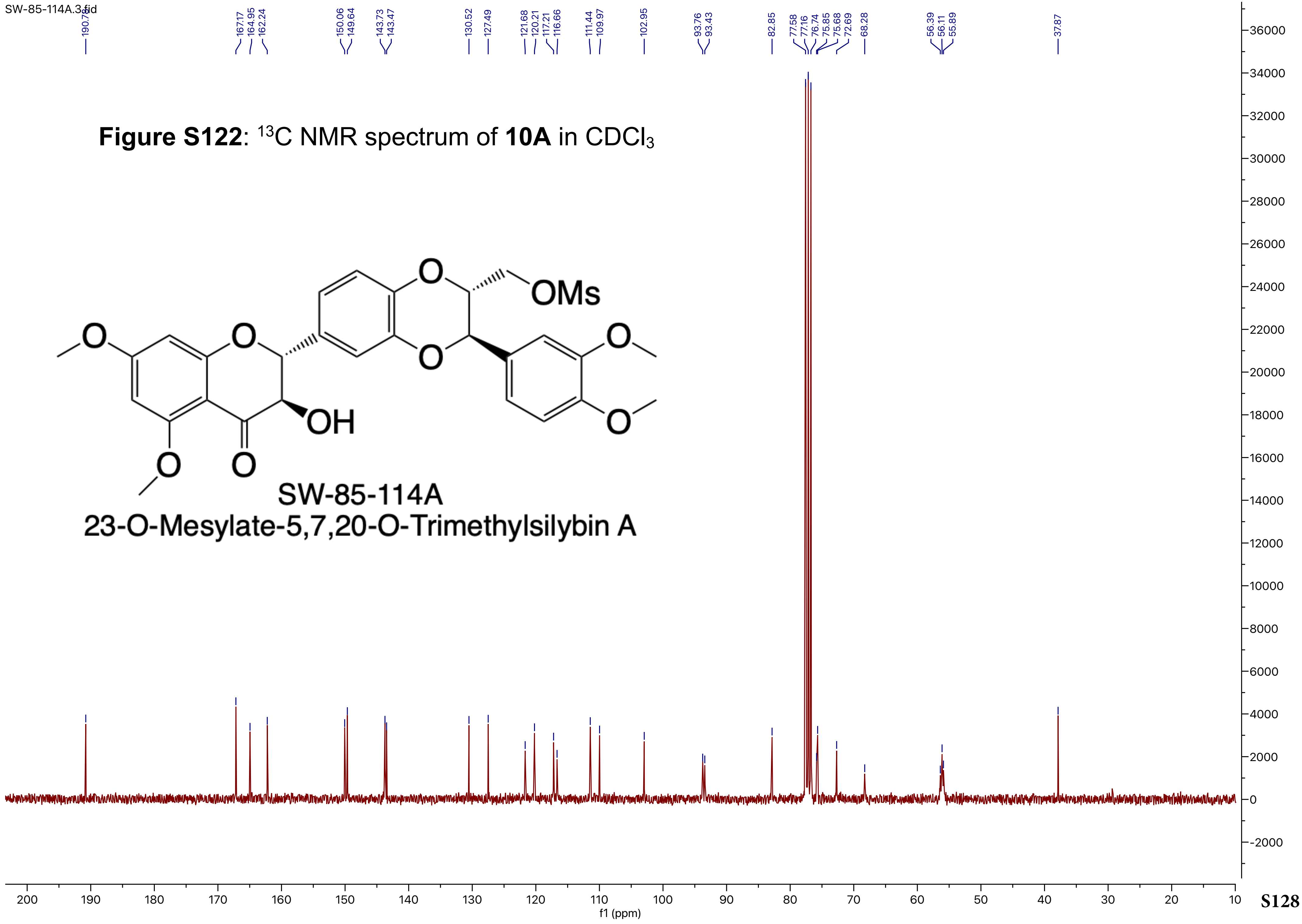

Figure S122: ^{13}C NMR spectrum of **10A** in $CDCl_3$

Sample Name	Mol Fomula	MW	M+H	observed	delta	ppm
SW-85-114A	C29H30O12S	602.1459	603.1537	603.1534	-0.0003	-0.43

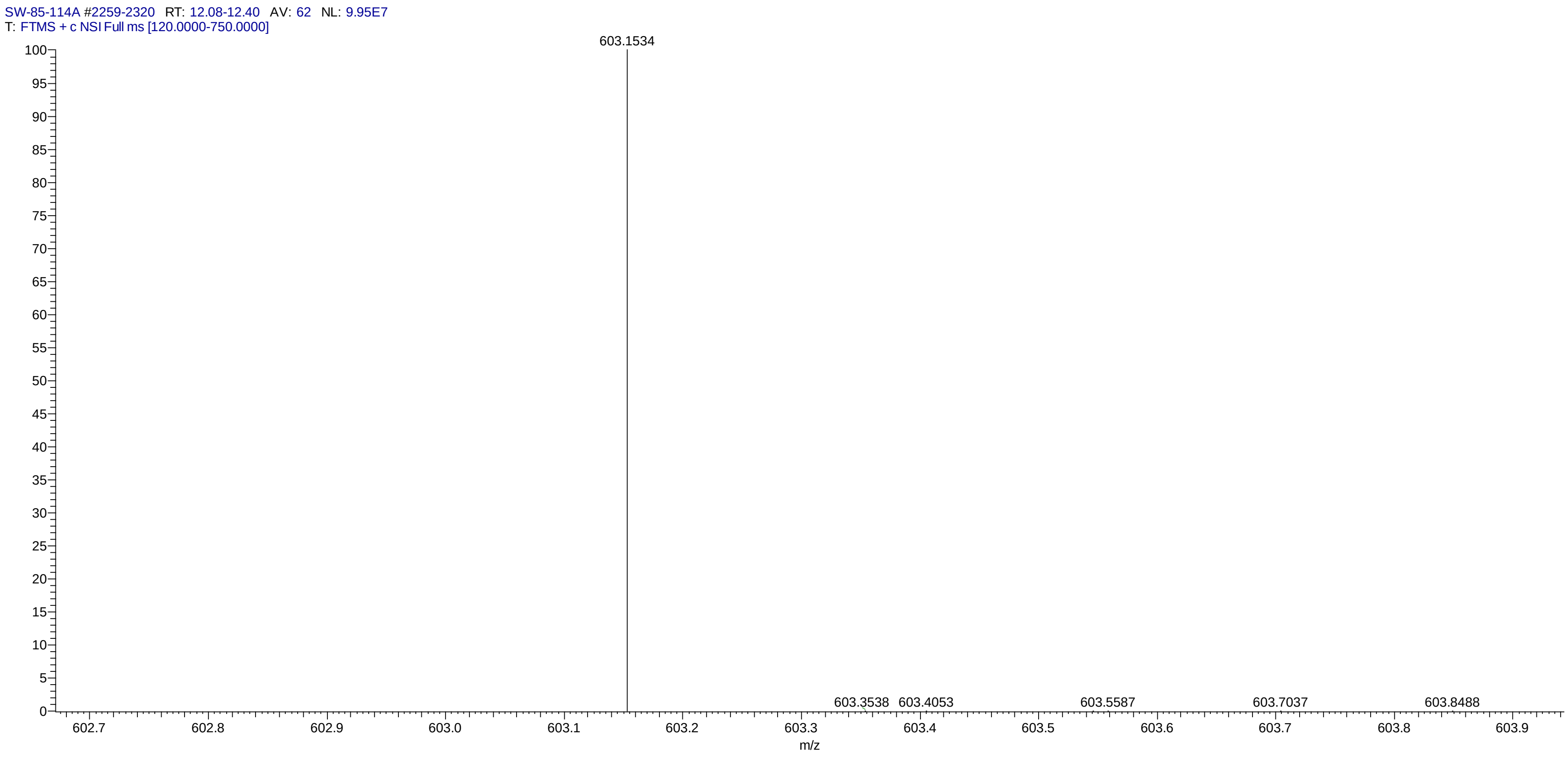

Figure S123: High resolution mass spectrum of **10A**

```
=====================================================================
Injection Date  : 4/20/2022 5:04:17 PM
Sample Name     : SW-85-114A                       Location : Vial 1
Acq. Operator   :
Acq. Method     : C:\HPCHEM\1\METHODS\JNP2015.M
Last changed    : 4/20/2022 5:00:25 PM
                  (modified after loading)
Analysis Method : C:\HPCHEM\1\METHODS\JNP2015.M
Last changed    : 4/20/2022 5:42:54 PM
                  (modified after loading)
```

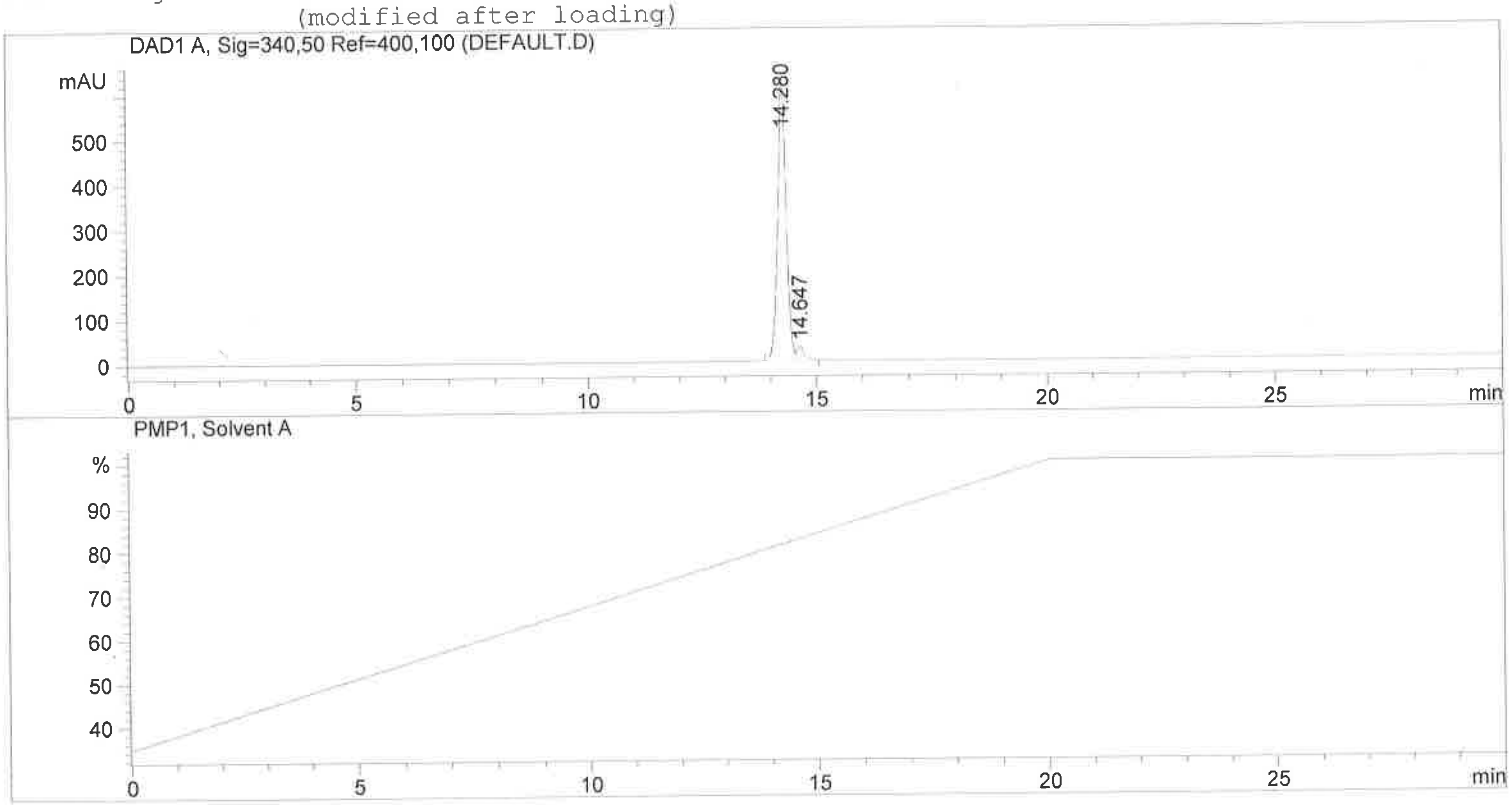

===
Area Percent Report
===

Sorted By : Signal
Multiplier : 1.0000
Dilution : 1.0000

Signal 1: DAD1 A, Sig=340,50 Ref=400,100

Peak #	RetTime [min]	Type	Width [min]	Area [mAU*s]	Height [mAU]	Area %
1	14.280	VV	0.2142	8131.07080	632.57202	95.2458
2	14.647	VV	0.2041	405.85938	33.14649	4.7542

Totals : 8536.93018 665.71851

Results obtained with enhanced integrator!

===

*** End of Report ***

Figure S124: HPLC chromatogram of **10A**

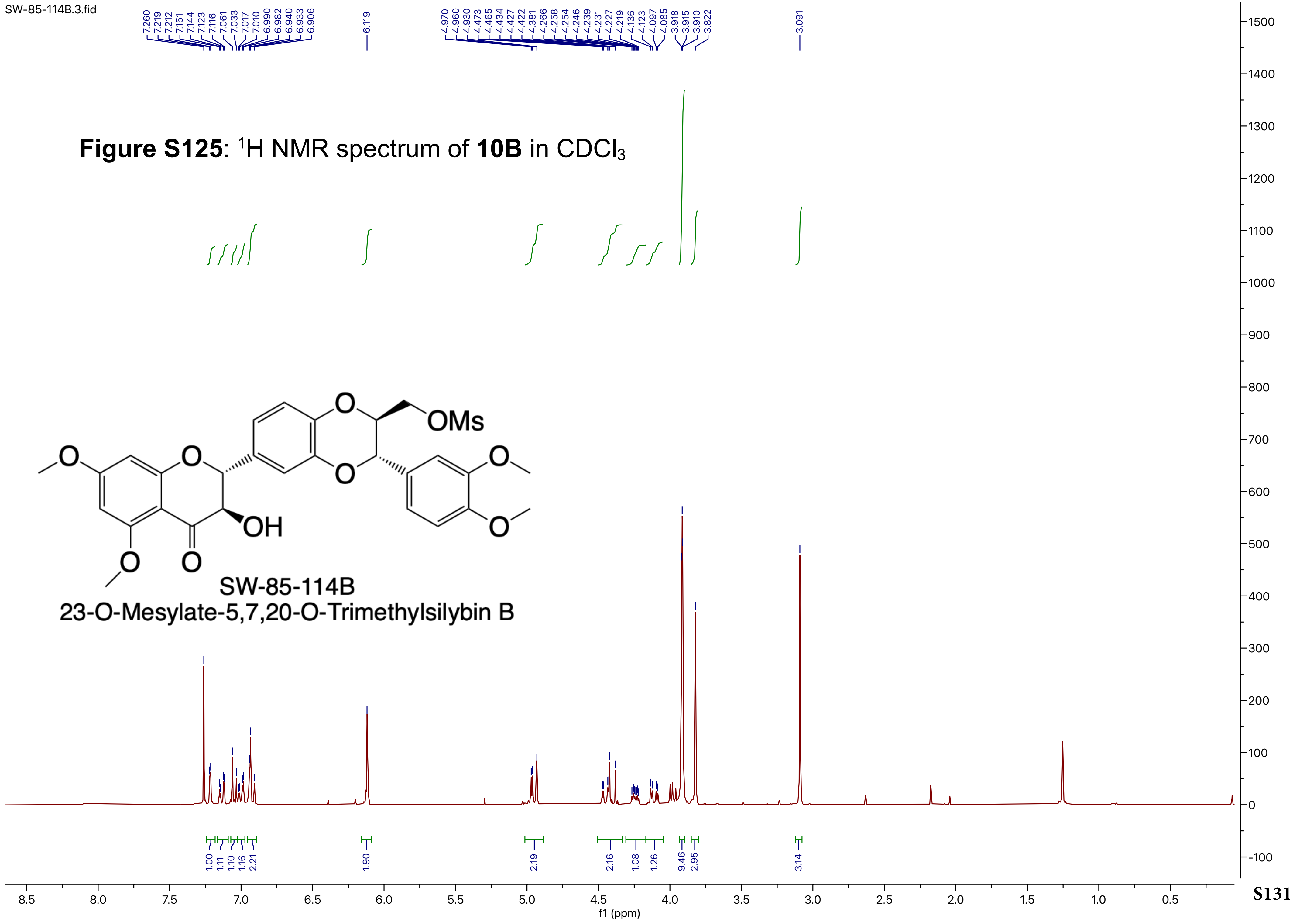

Figure S125: ^{1}H NMR spectrum of **10B** in $CDCl_3$

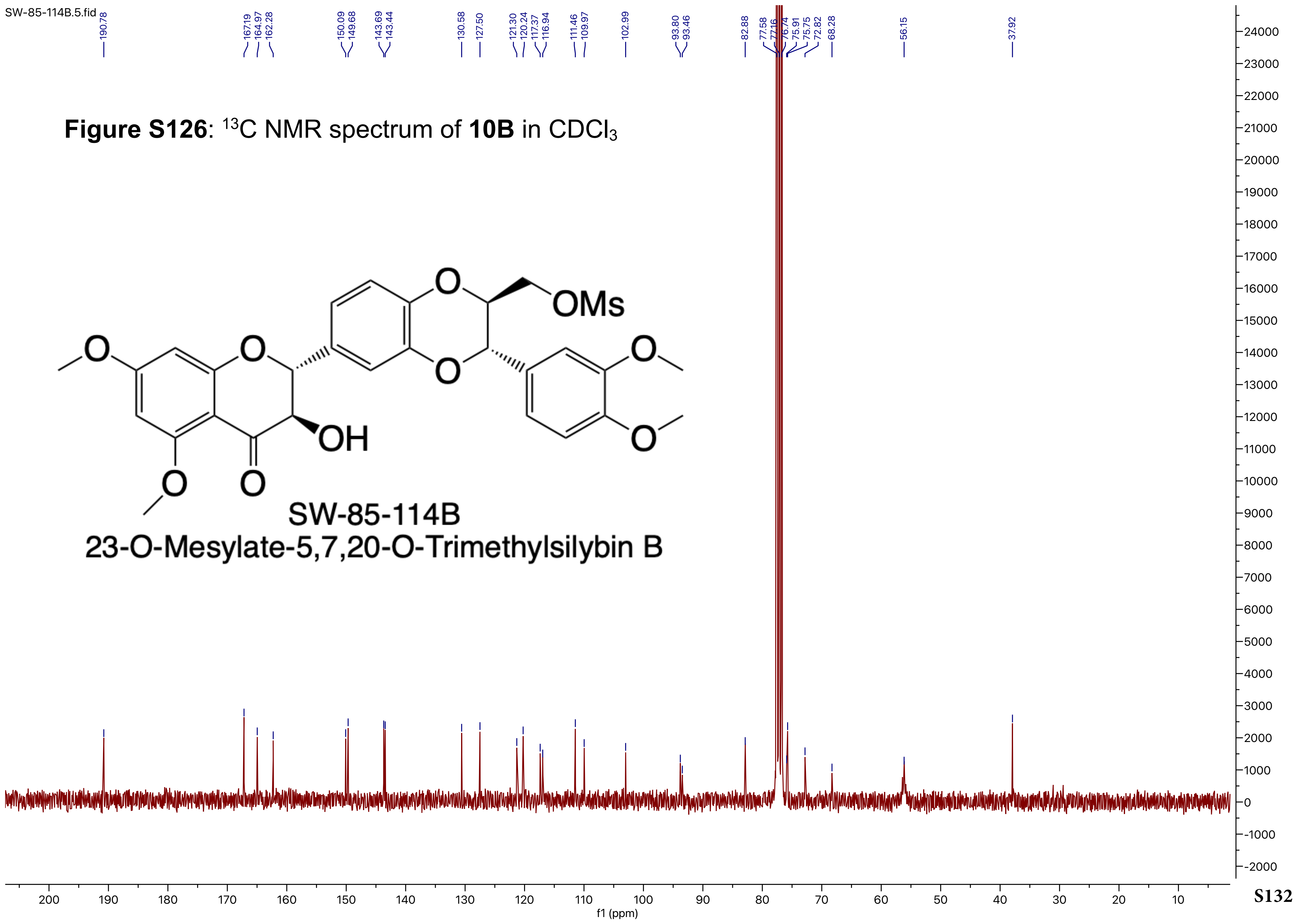

Figure S126: ^{13}C NMR spectrum of **10B** in $CDCl_3$

Sample Name	Mol Fomula	MW	M+H	observed	delta	ppm
SW-85-114B	C29H30O12S	602.1459	603.1537	603.1537	0.0000	0.00

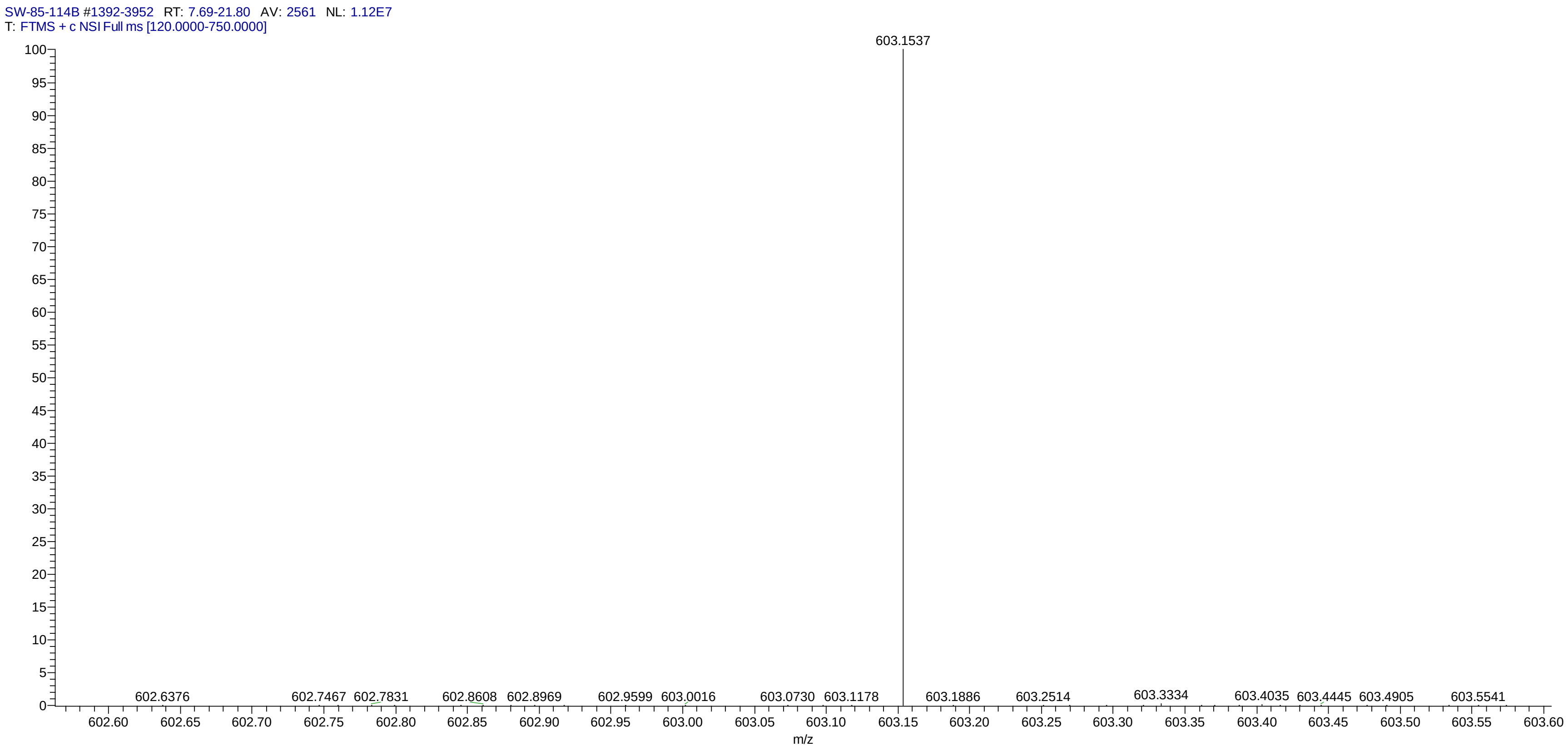

Figure S127: High resolution mass spectrum of **10B**

Injection Date : 4/19/2022 1:48:14 PM
Sample Name : SW-85-114B Location : Vial 1
Acq. Operator :
Method : C:\HPCHEM\1\METHODS\JNP2015.M
Last changed : 4/19/2022 12:01:10 PM
(modified after loading)

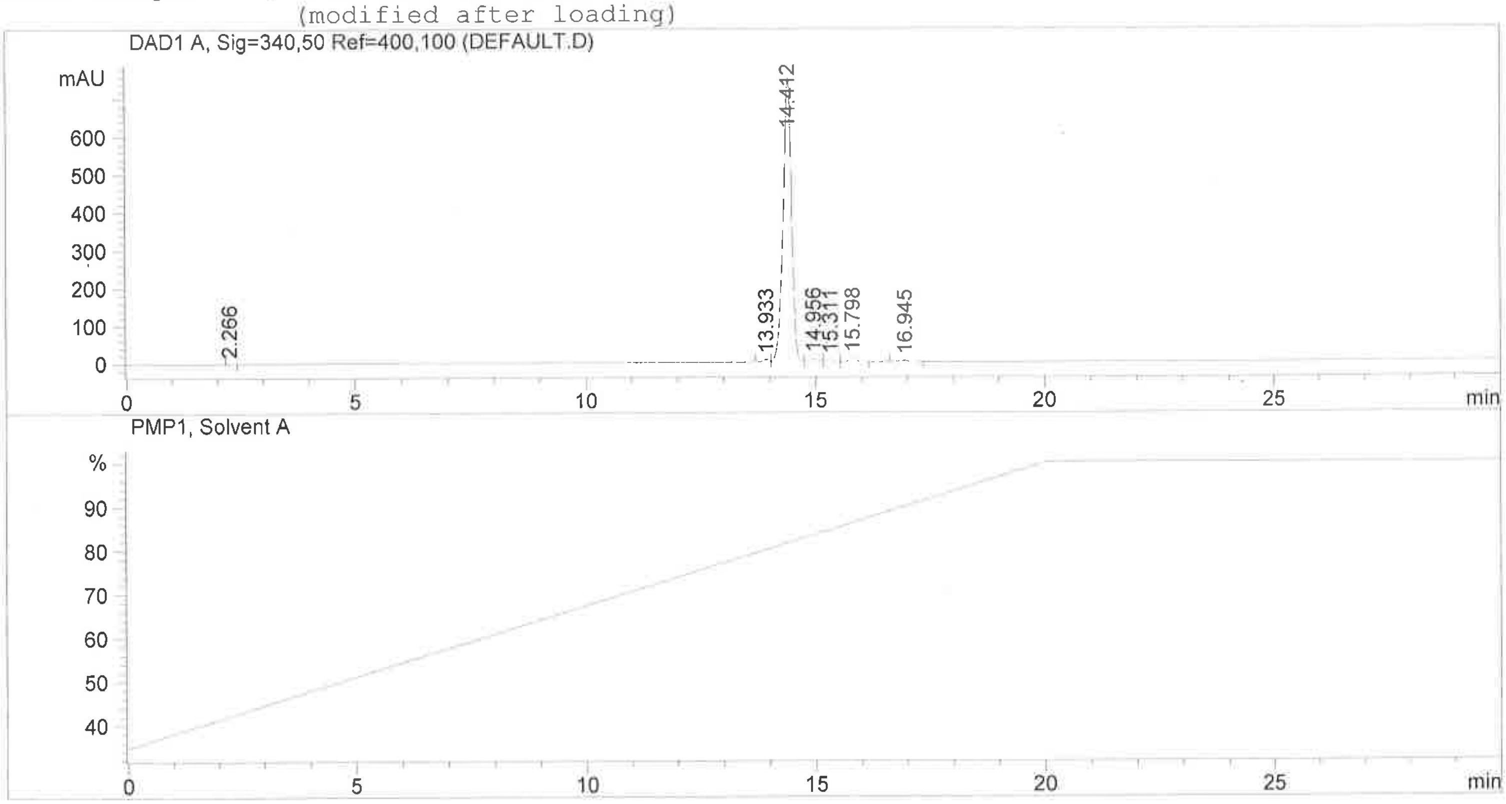

Area Percent Report

Sorted By : Signal
Multiplier : 1.0000
Dilution : 1.0000

Signal 1: DAD1 A, Sig=340,50 Ref=400,100

Peak #	RetTime [min]	Type	Width [min]	Area [mAU*s]	Height [mAU]	Area %
1	2.266	PB	0.1160	12.26225	1.45977	0.1233
2	13.933	BV	0.1487	72.28875	7.22981	0.7268
3	14.412	VV	0.1853	9503.08984	752.57904	95.5456
4	14.956	VV	0.1775	120.82584	9.55987	1.2148
5	15.311	VV	0.1562	69.23528	6.60800	0.6961
6	15.798	VB	0.1742	113.87707	9.90321	1.1449
7	16.945	PP	0.2162	54.55200	3.38083	0.5485
Totals :				9946.13103	790.72053	

Results obtained with enhanced integrator!

*** End of Report ***

Figure S128: HPLC chromatogram of **10B**

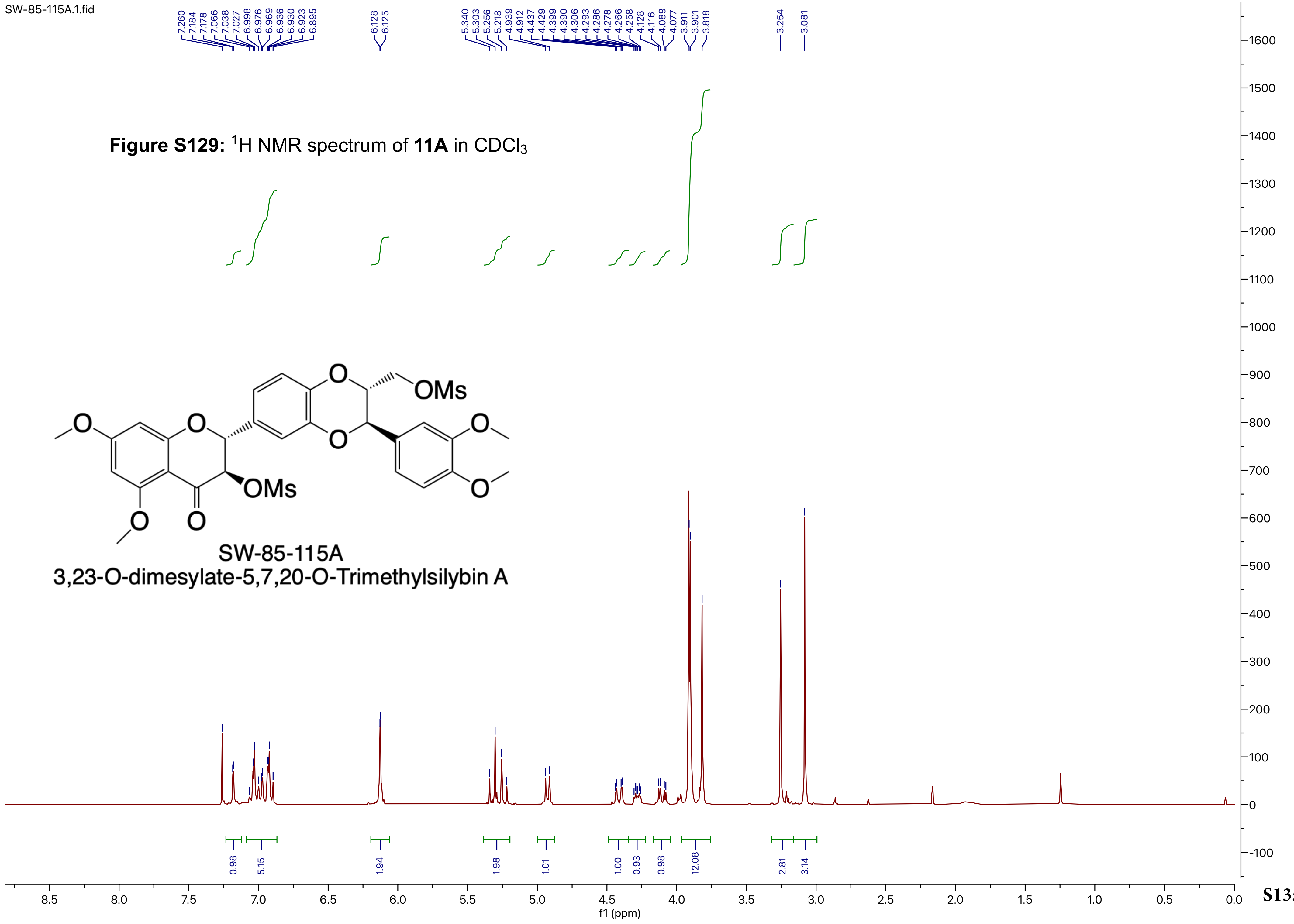

Figure S129: ^{1}H NMR spectrum of **11A** in $CDCl_3$

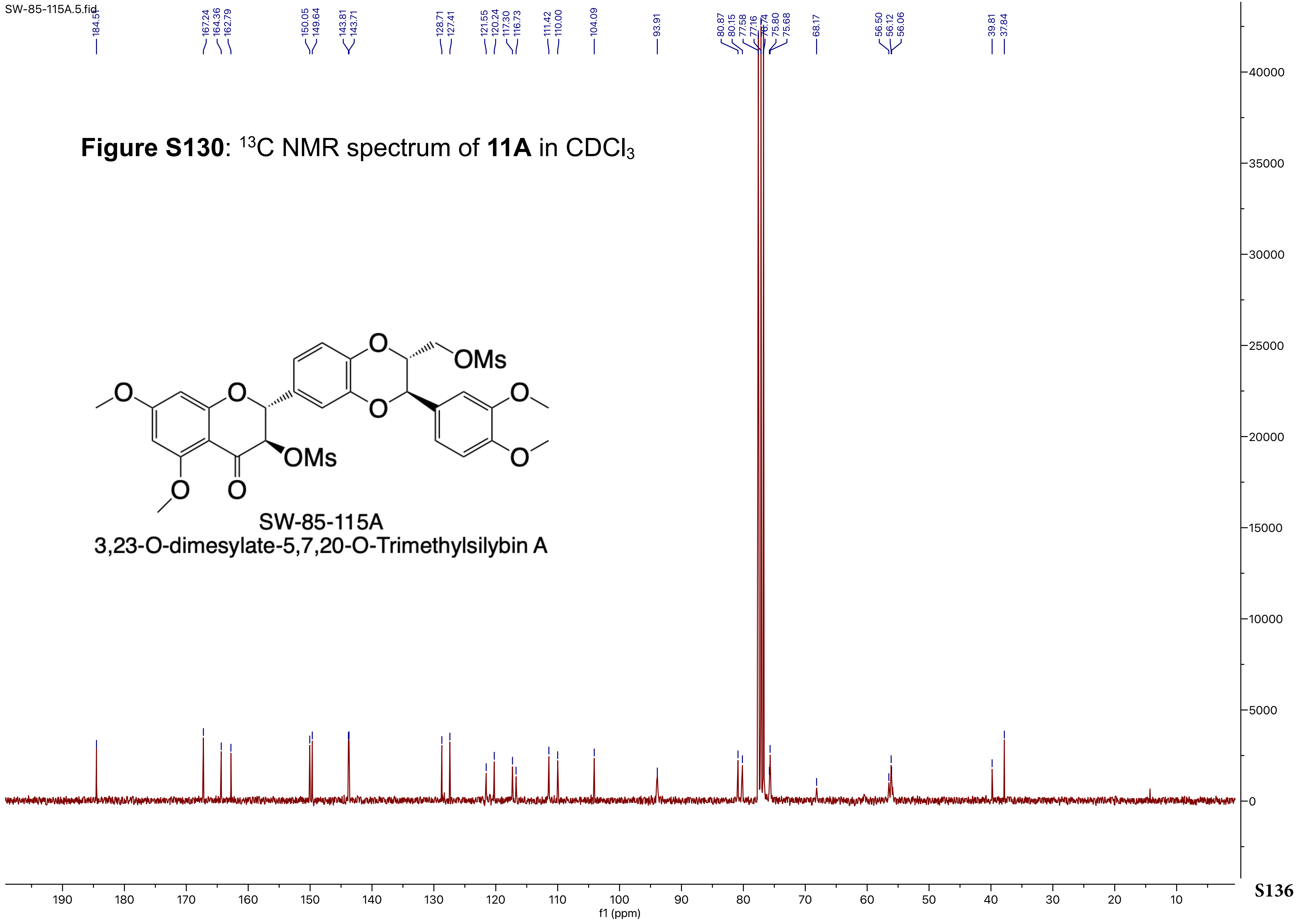

Figure S130: ^{13}C NMR spectrum of **11A** in $CDCl_3$

SW-85-115A
3,23-O-dimesylate-5,7,20-O-Trimethylsilybin A

Sample Name	Mol Fomula	MW	M+H	observed	delta	ppm
SW-85-115A	C30H32O14S2	680.1234	681.1312	681.1306	-0.0006	-0.91

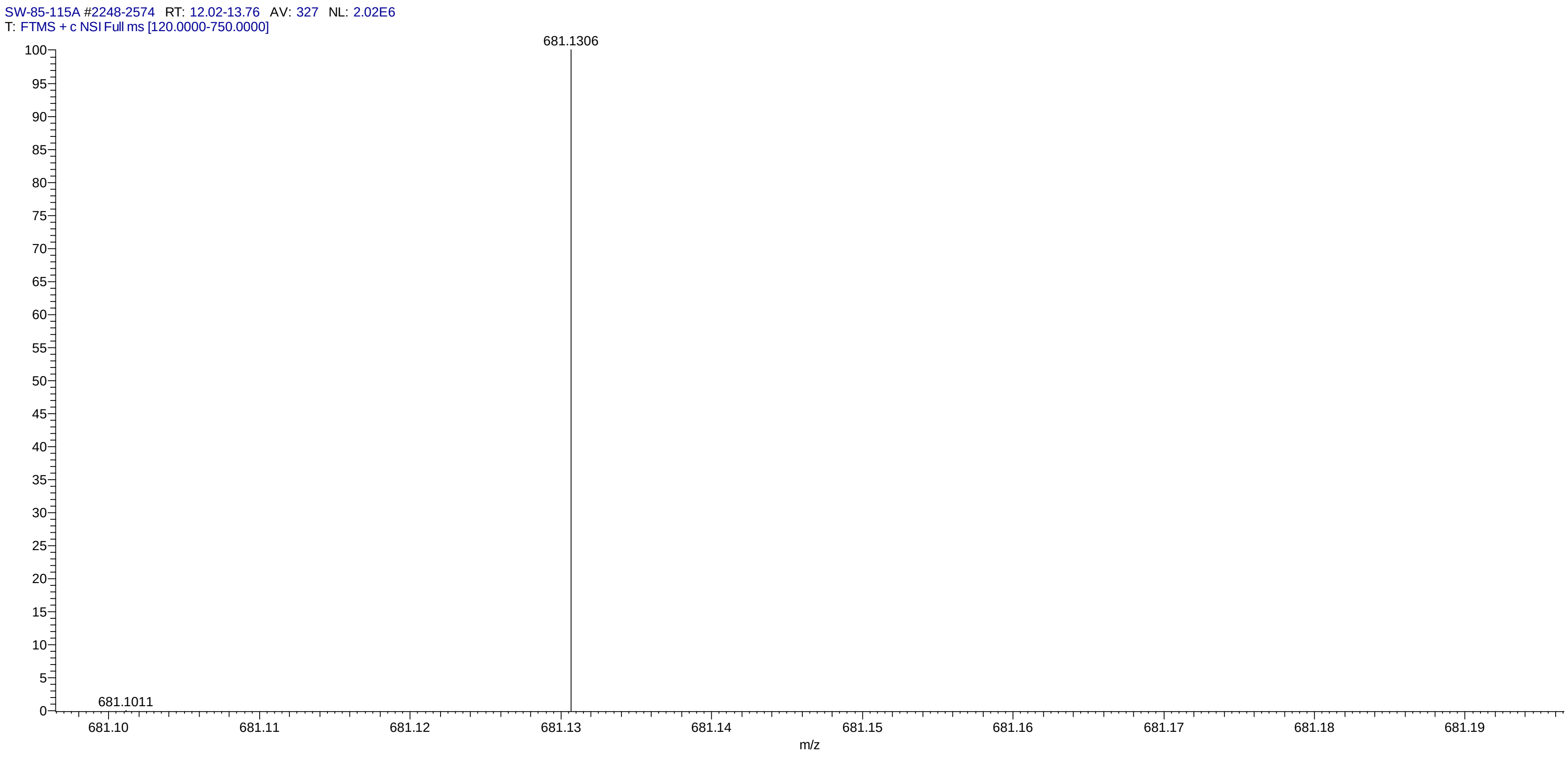

Figure S131: High resolution mass spectrum of **11A**

===

Injection Date : 4/23/2022 4:42:53 PM
Sample Name : SW-85-115A Location : Vial 1
Acq. Operator :
Method : C:\HPCHEM\1\METHODS\JNP2015.M
Last changed : 4/22/2022 5:19:36 PM
(modified after loading)

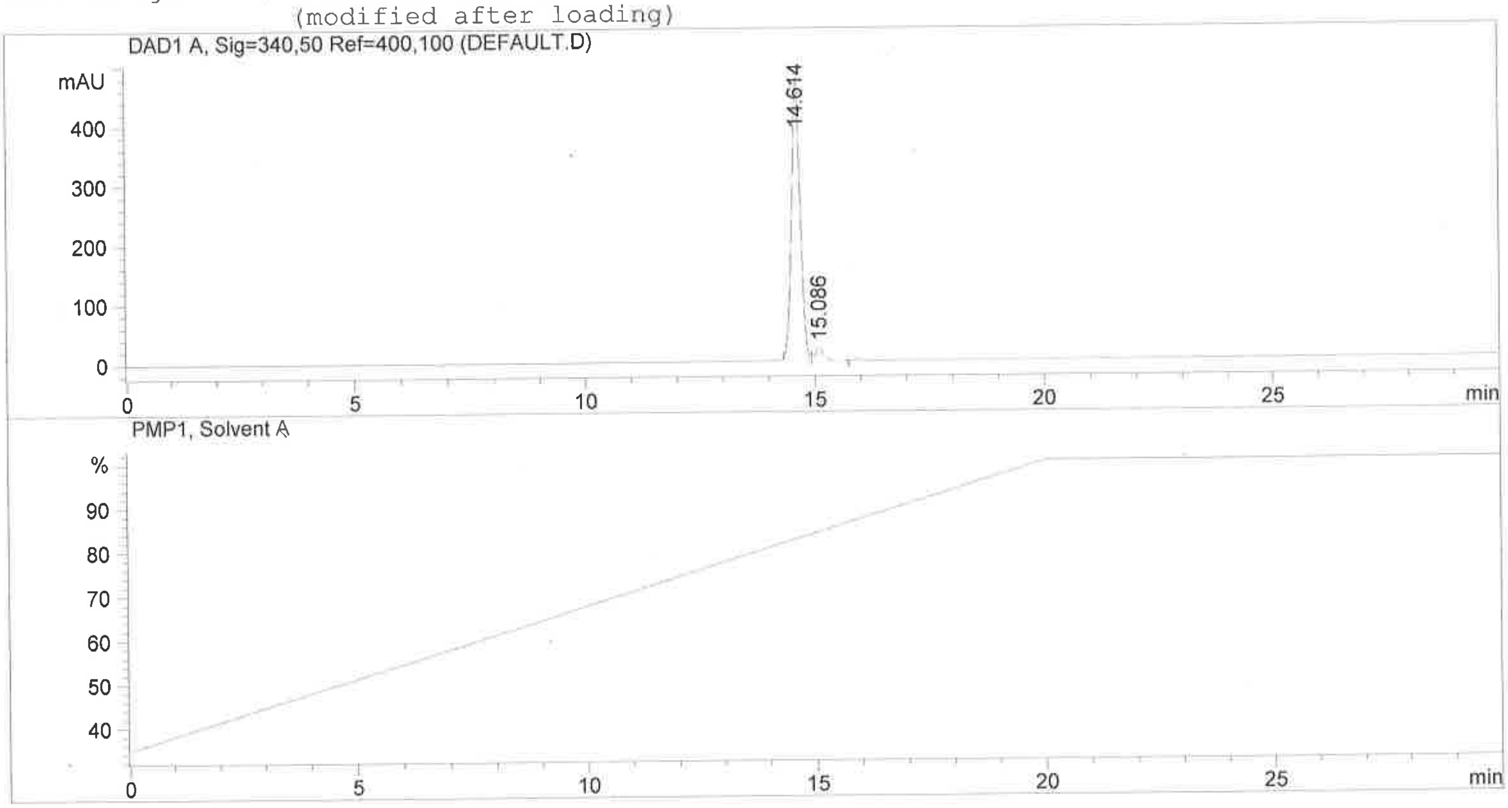

===

Area Percent Report

===

Sorted By : Signal
Multiplier : 1.0000
Dilution : 1.0000

Signal 1: DAD1 A, Sig=340,50 Ref=400,100

Peak #	RetTime [min]	Type	Width [min]	Area [mAU*s]	Height [mAU]	Area %
1	14.614	BV	0.1877	6104.35303	475.69272	95.9774
2	15.086	VP	0.1632	255.84680	23.11047	4.0226

Totals : 6360.19983 498.80318

Results obtained with enhanced integrator!

===

*** End of Report ***

Figure S132: HPLC chromatogram of **11A**

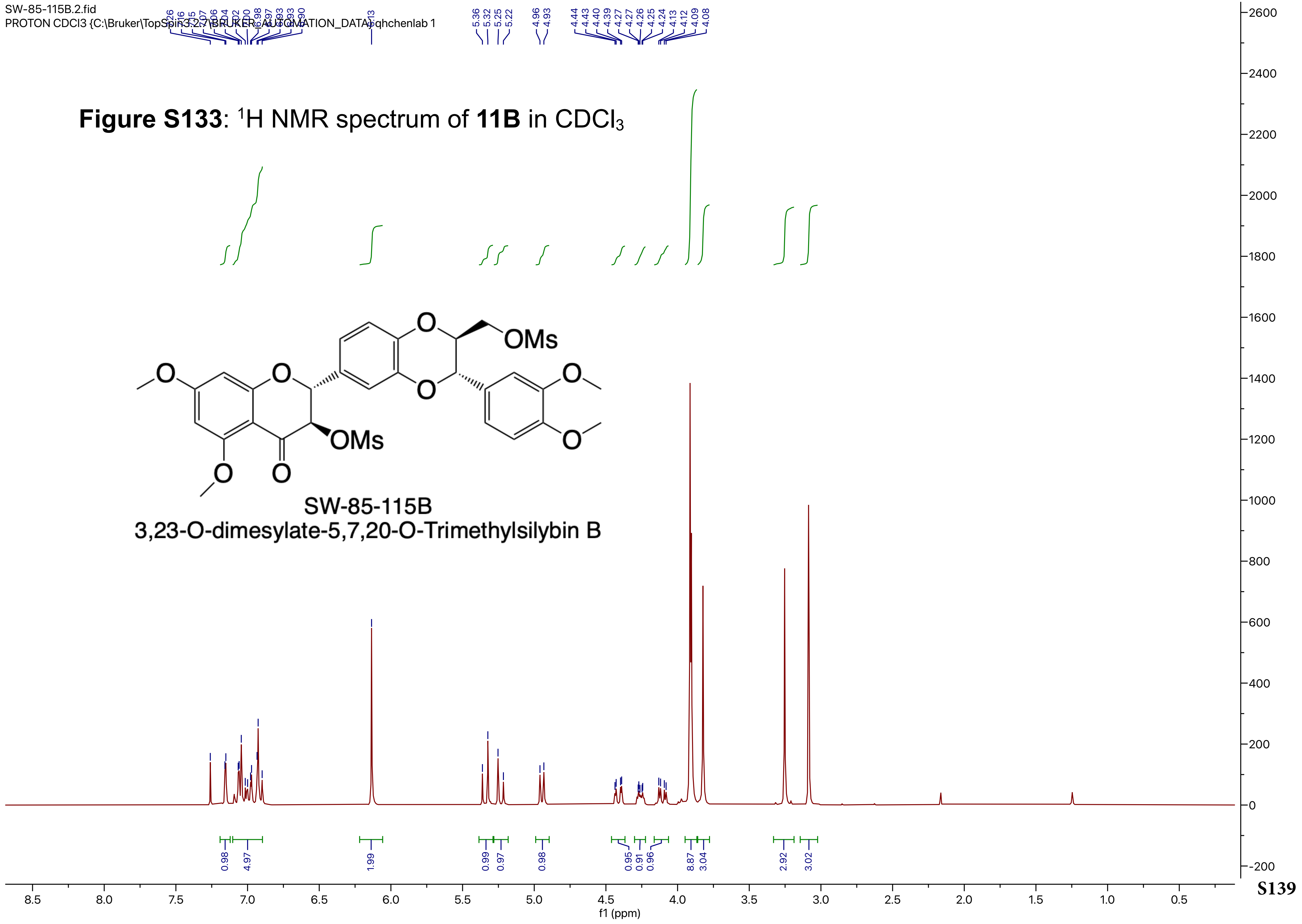

Figure S133: ^{1}H NMR spectrum of **11B** in $CDCl_3$

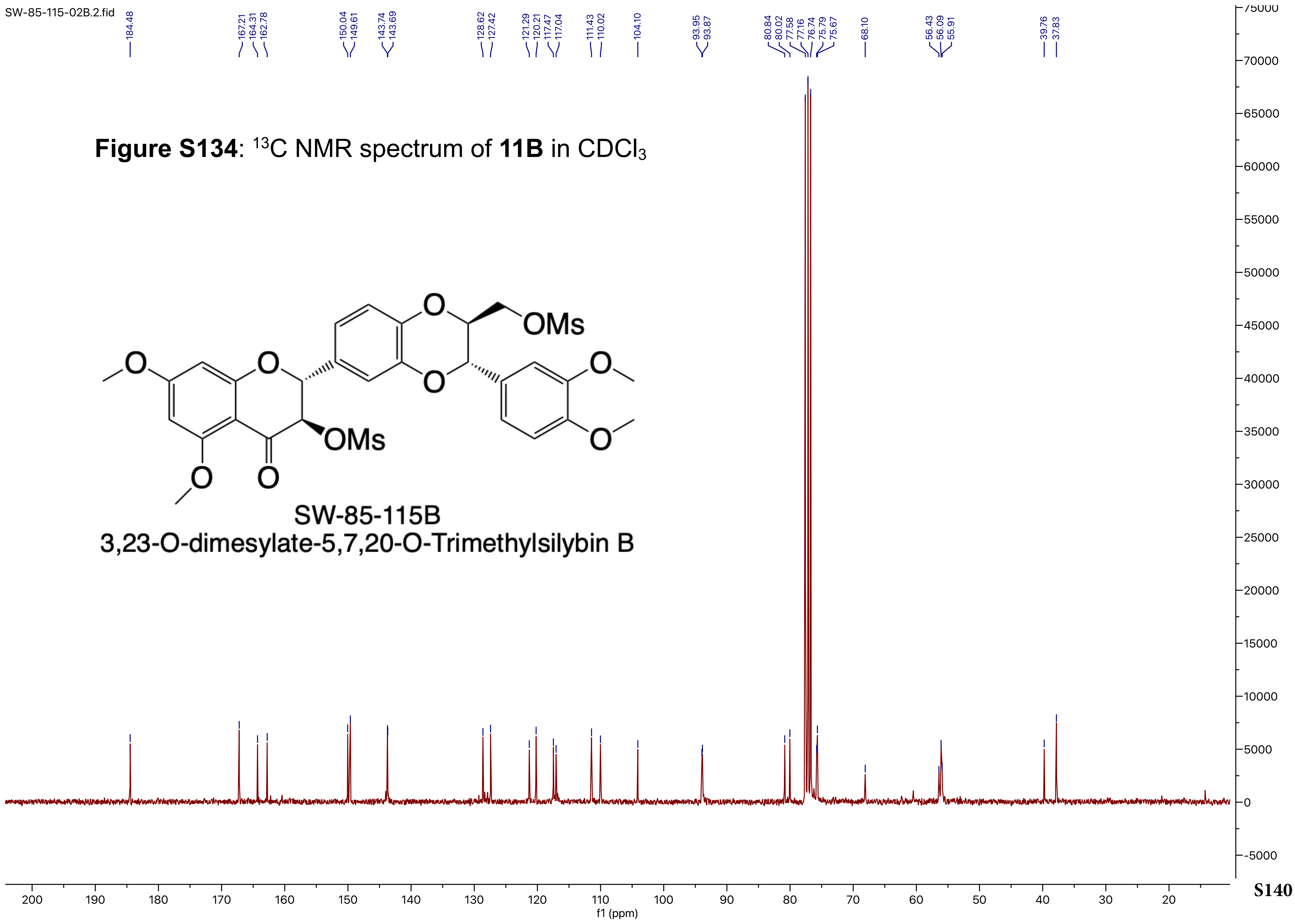

Figure S134: ^{13}C NMR spectrum of **11B** in $CDCl_3$

SW-85-115B

3,23-O-dimesylate-5,7,20-O-Trimethylsilybin B

Sample Name	Mol Fomula	MW	M+H	observed	delta	ppm
SW-85-115B	C30H32O14S2	680.1234	681.1312	681.1310	-0.0002	-0.32

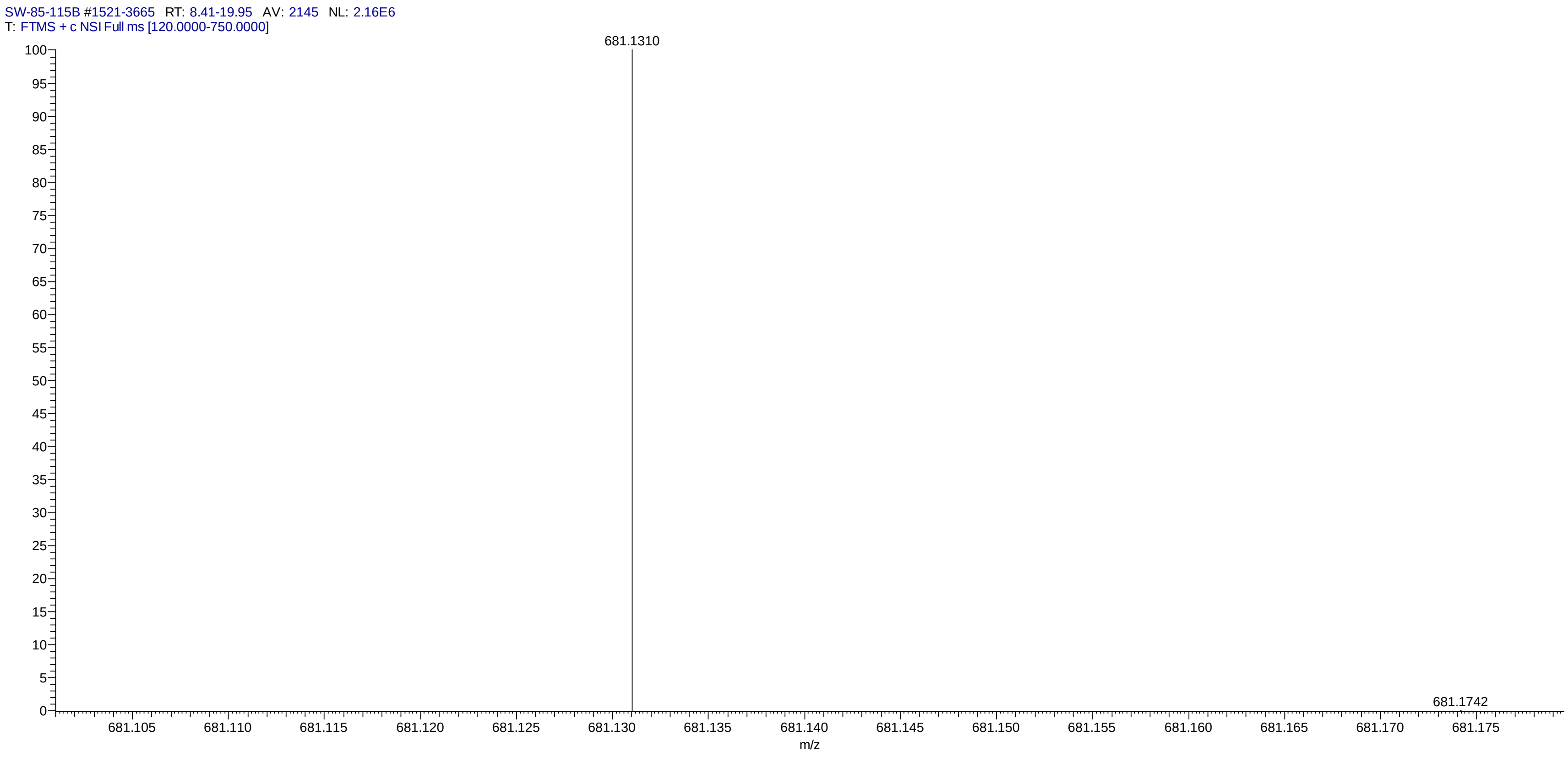

Figure S135: High resolution mass spectrum of **11B**

```
=====================================================================
Injection Date  : 4/23/2022 5:28:28 PM
Sample Name     : SW-85-115B                    Location : Vial 1
Acq. Operator   :
Method          : C:\HPCHEM\1\METHODS\JNP2015.M
Last changed    : 4/22/2022 5:19:36 PM
                  (modified after loading)
```

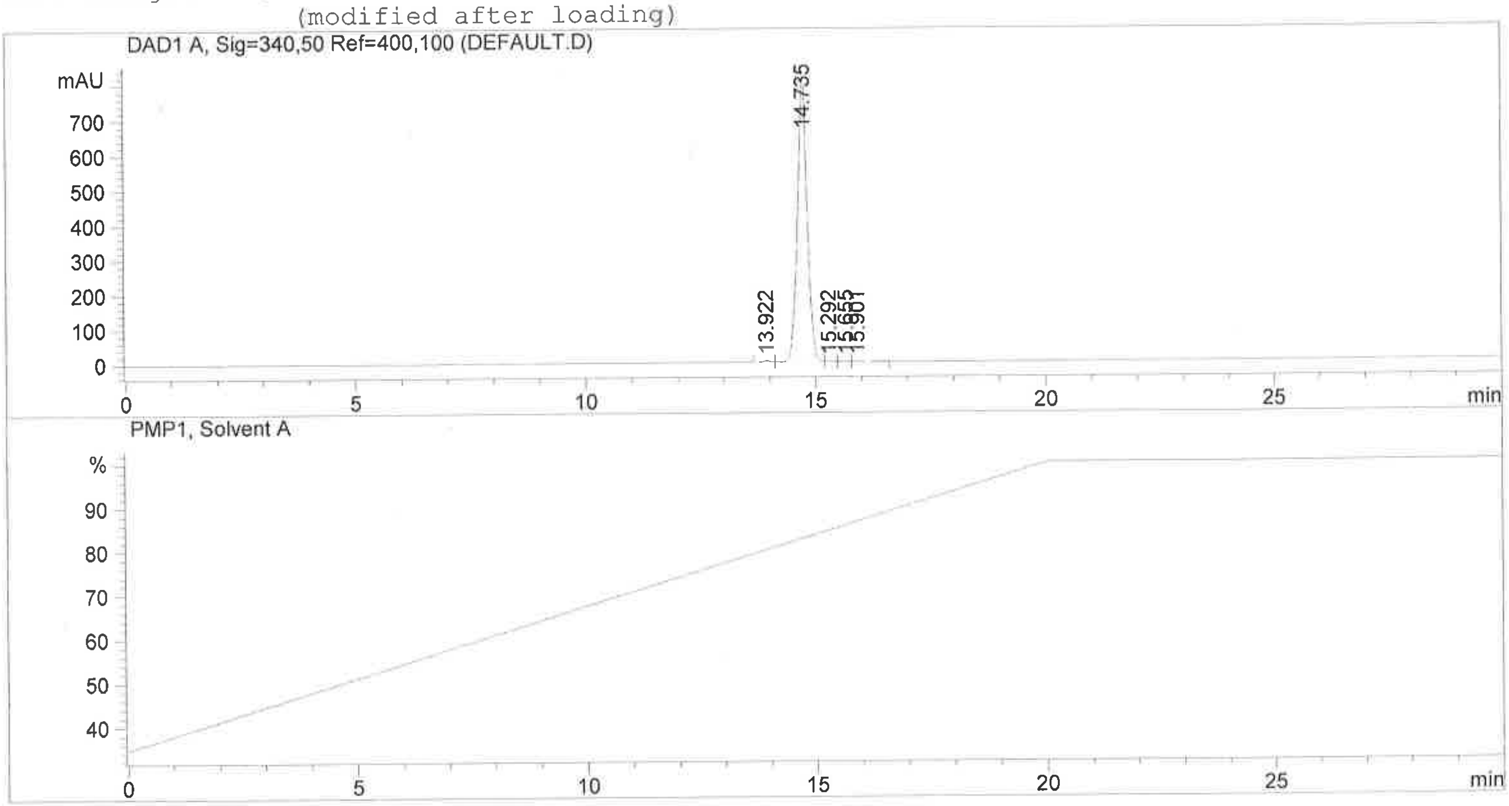


```
=====================================================================
                      Area Percent Report
=====================================================================

Sorted By             :      Signal
Multiplier            :      1.0000
Dilution              :      1.0000
```

Signal 1: DAD1 A, Sig=340,50 Ref=400,100

Peak #	RetTime [min]	Type	Width [min]	Area [mAU*s]	Height [mAU]	Area %
1	13.922	BV	0.1707	47.03403	4.01530	0.3891
2	14.735	VV	0.2151	1.19343e4	813.72095	98.7236
3	15.292	VV	0.1652	36.68073	3.21229	0.3034
4	15.655	VV	0.1810	34.45500	2.85137	0.2850
5	15.901	VP	0.1856	36.12410	2.81770	0.2988
Totals :				1.20886e4	826.61761	

Results obtained with enhanced integrator!

```
=====================================================================
                      *** End of Report ***
```

Figure S136: HPLC chromatogram of **11B**

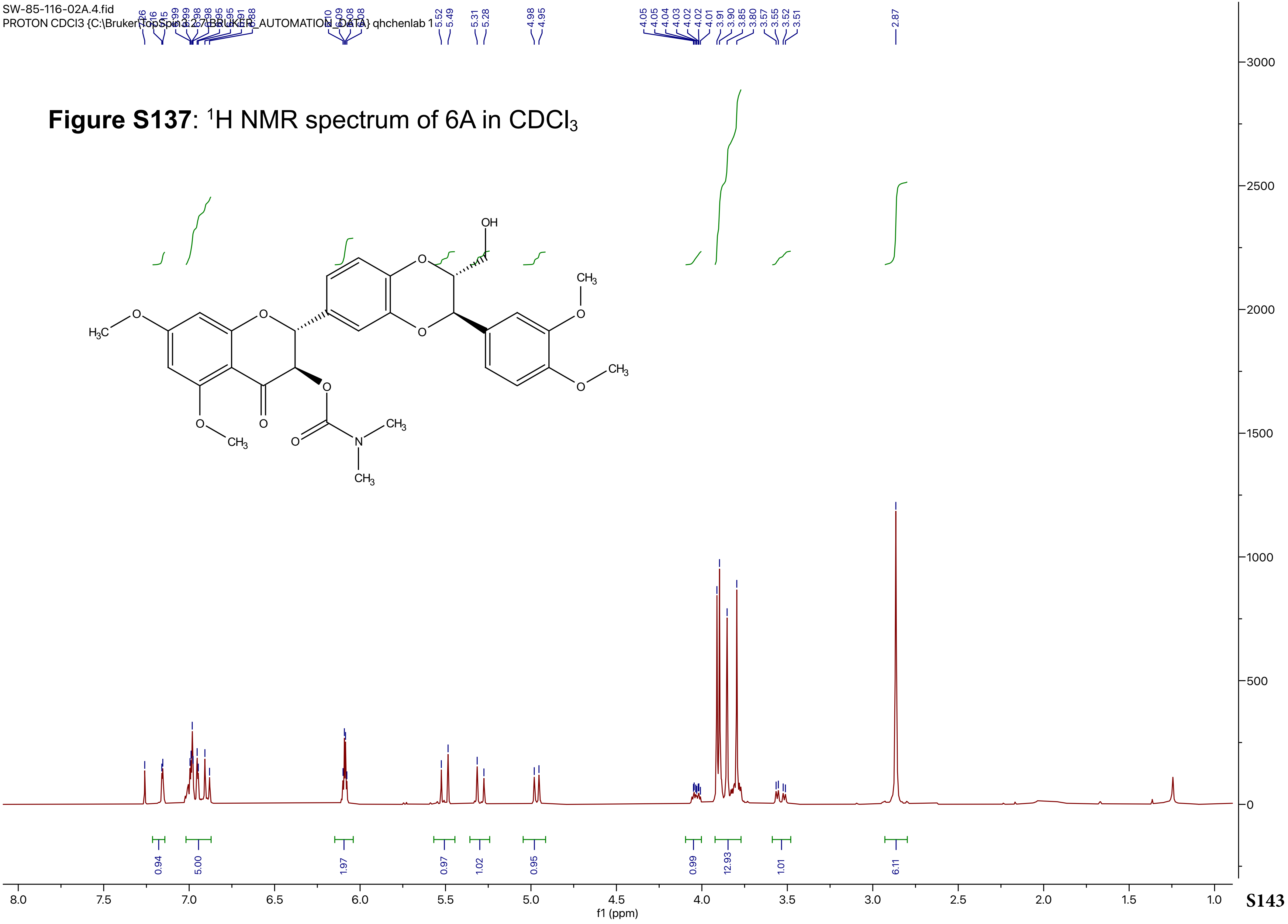

Figure S137: ^{1}H NMR spectrum of 6A in $CDCl_3$

Figure S138: ^{13}C NMR spectrum of **6A** in $CDCl_3$

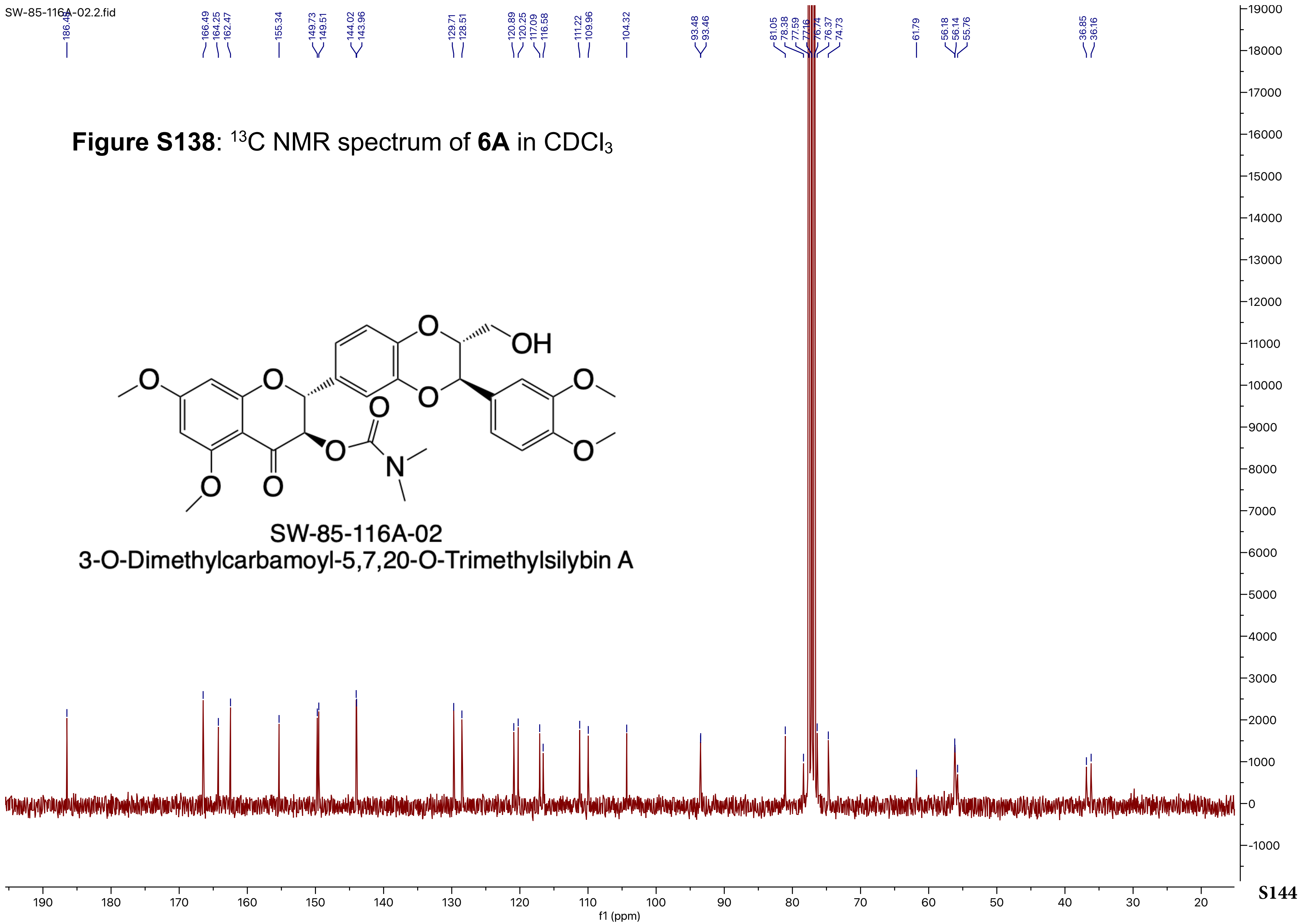

SW-85-116A-02

3-O-Dimethylcarbamoyl-5,7,20-O-Trimethylsilybin A

Sample Name	Mol Fomula	MW	M+H	observed	delta	ppm
SW-85-116A-02	C31H33NO11	595.2054	596.2132	596.2129	-0.0003	-0.52

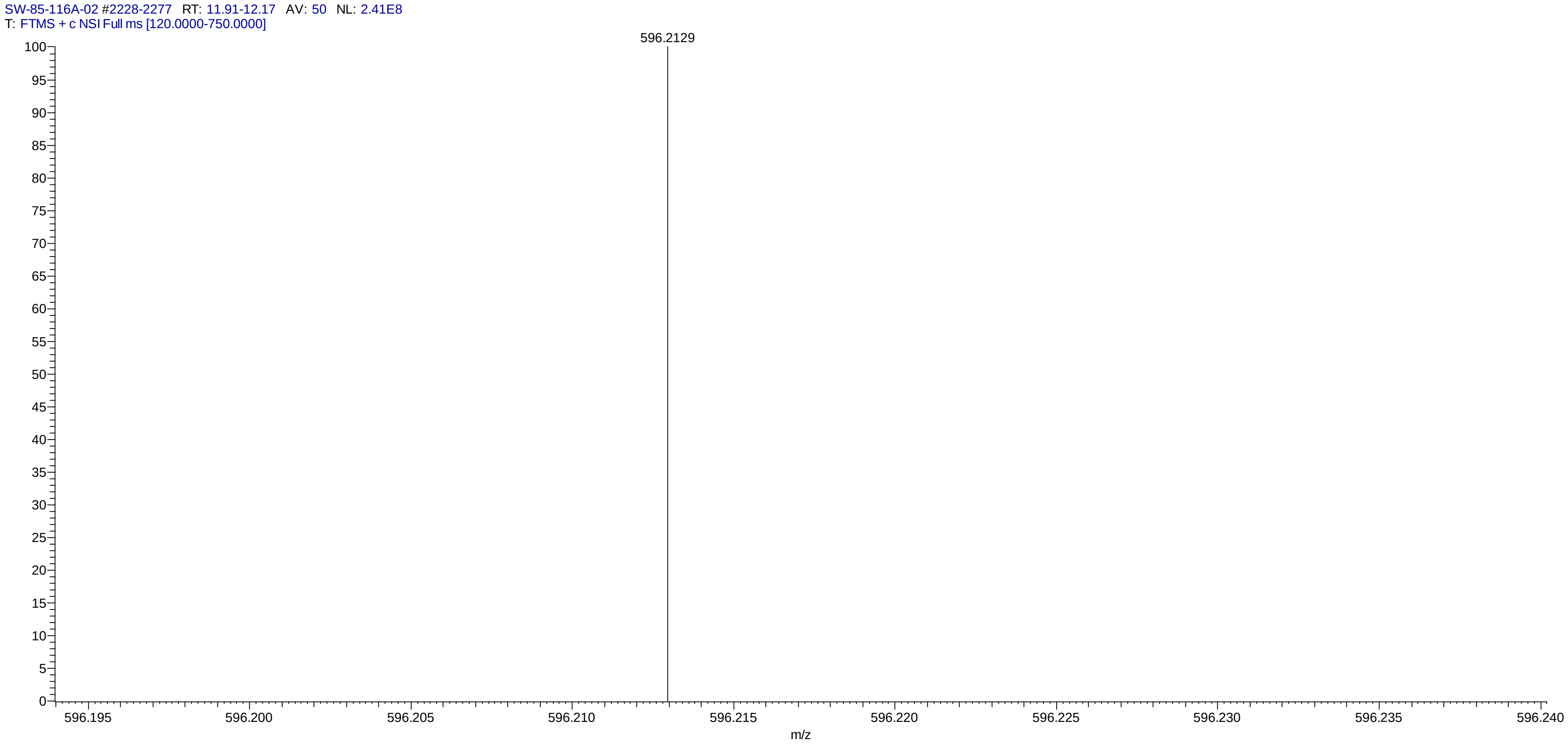

Figure S139: High resolution mass spectrum of **6A**

Data File C:\HPCHEM\1\DATA\DEFAULT.D Sample Name: SW-85-116A-02

===

Injection Date : 4/18/2022 3:52:21 PM
Sample Name : SW-85-116A-02 Location : Vial 1
Acq. Operator :
Method : C:\HPCHEM\1\METHODS\JNP2015.M
Last changed : 4/18/2022 3:51:49 PM
(modified after loading)

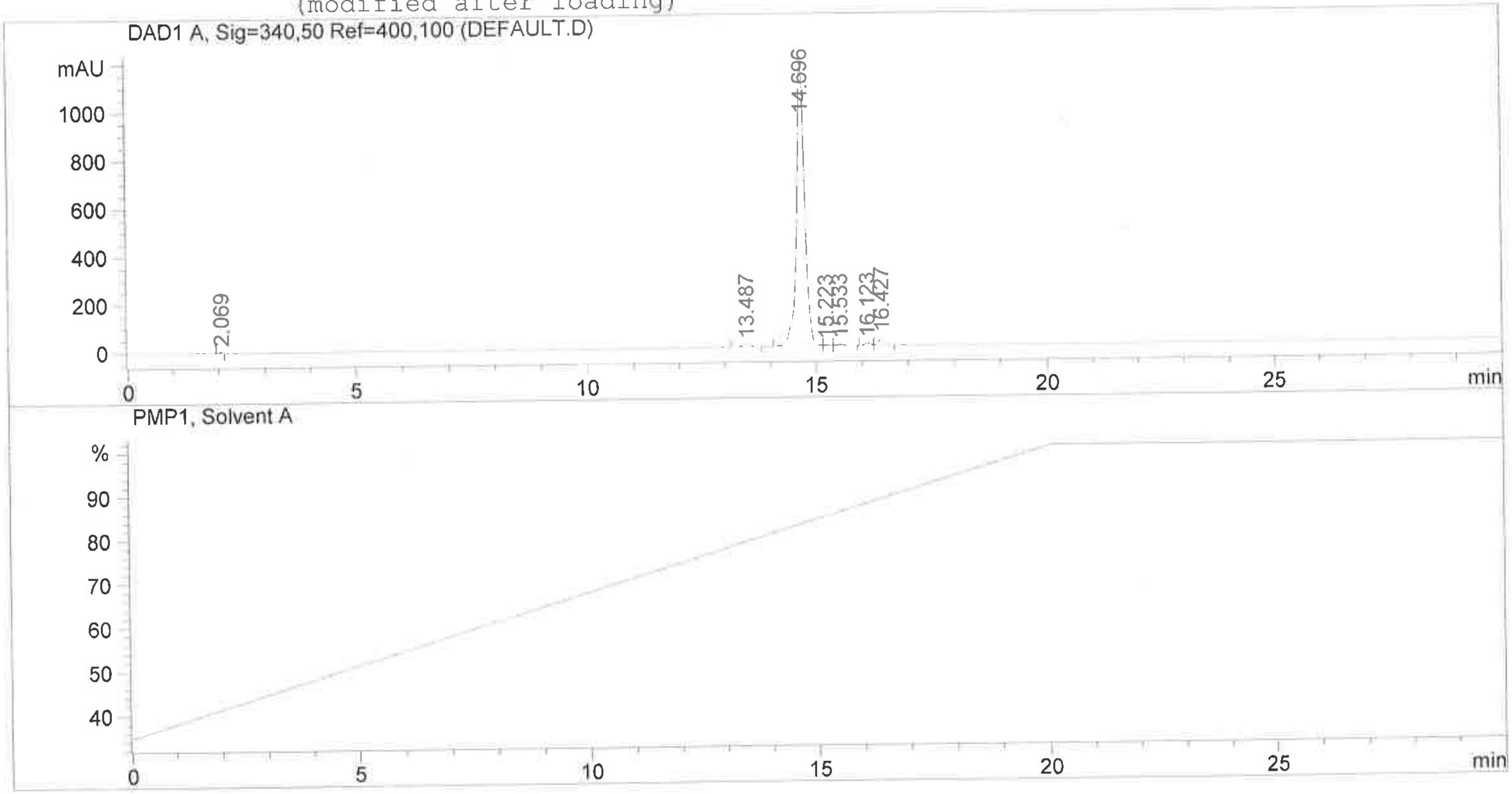

===

Area Percent Report

===

Sorted By : Signal
Multiplier : 1.0000
Dilution : 1.0000

Signal 1: DAD1 A, Sig=340,50 Ref=400,100

Peak #	RetTime [min]	Type	Width [min]	Area [mAU*s]	Height [mAU]	Area %
1	2.069	BP	0.0887	45.51932	7.11014	0.2993
2	13.487	BB	0.1824	119.77469	9.67877	0.7875
3	14.696	BB	0.1824	1.45948e4	1179.54858	95.9623
4	15.223	BV	0.1444	29.75826	2.98306	0.1957
5	15.533	VP	0.1661	54.19590	4.78533	0.3563
6	16.123	BV	0.1288	77.73266	9.17496	0.5111
7	16.427	VB	0.1378	287.10831	31.65473	1.8878
Totals :				1.52089e4	1244.93559	

Results obtained with enhanced integrator!

===

*** End of Report ***

Figure S140: HPLC chromatogram of **6A**

Figure S141: ^{1}H NMR spectrum of **6B** in $CDCl_3$

SW-85-116B-02
3-O-Dimethylcarbamoyl-5,7,20-O-Trimethylsilybin B

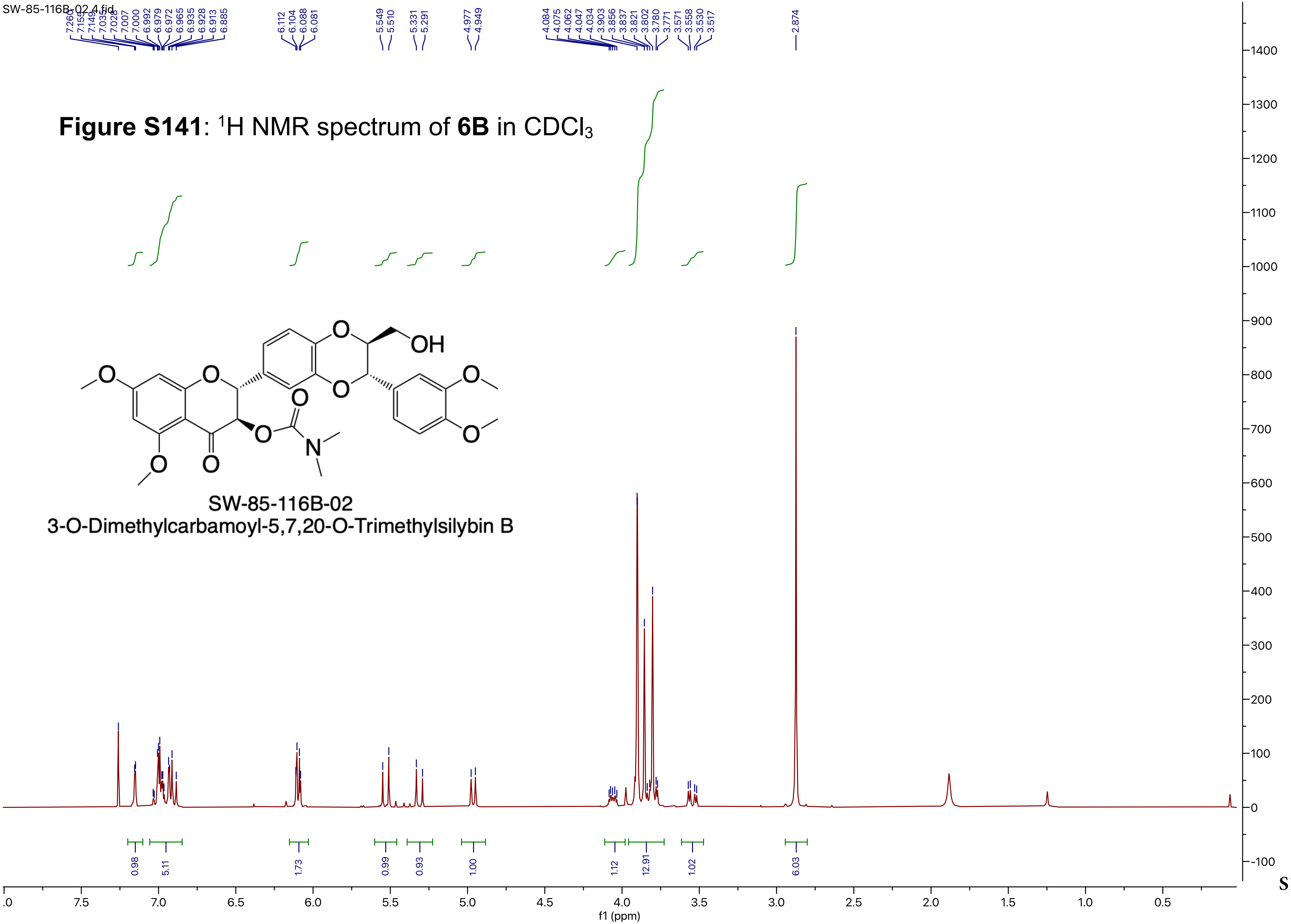

Figure S142: ^{13}C NMR spectrum of **6B** in $CDCl_3$

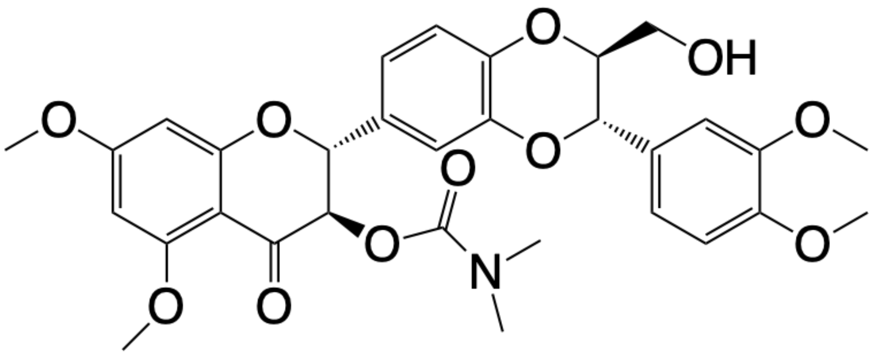

SW-85-116B-02
3-O-Dimethylcarbamoyl-5,7,20-O-Trimethylsilybin B

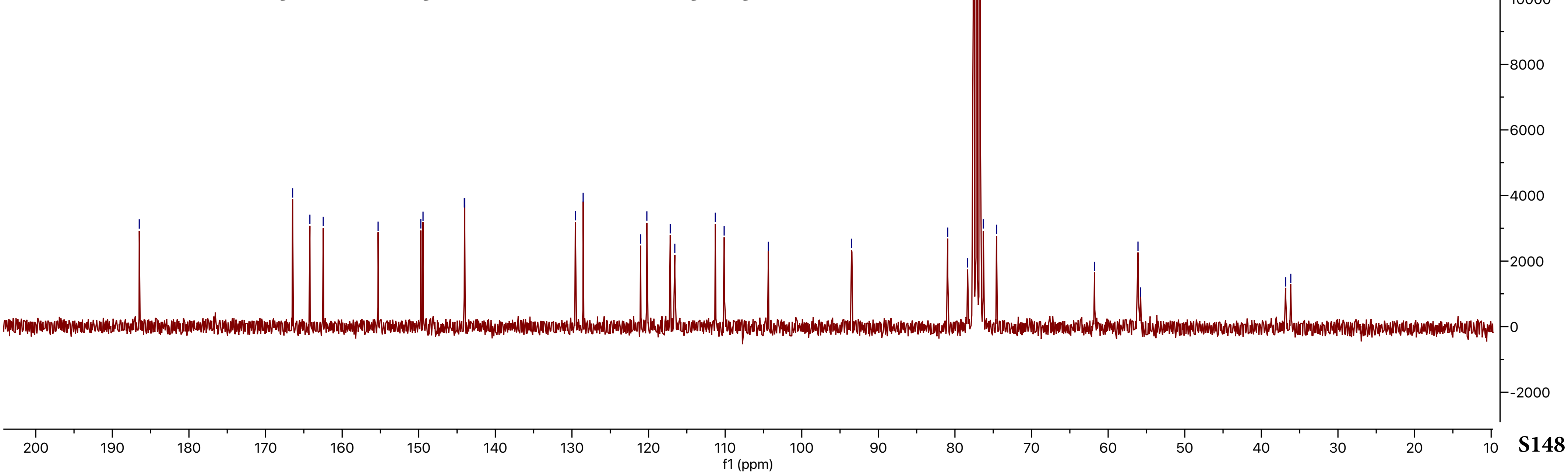

Sample Name	Mol Fomula	MW	M+H	observed	delta	ppm
SW-85-116B-02	C31H33NO11	595.2054	596.2132	596.2133	0.0001	0.15

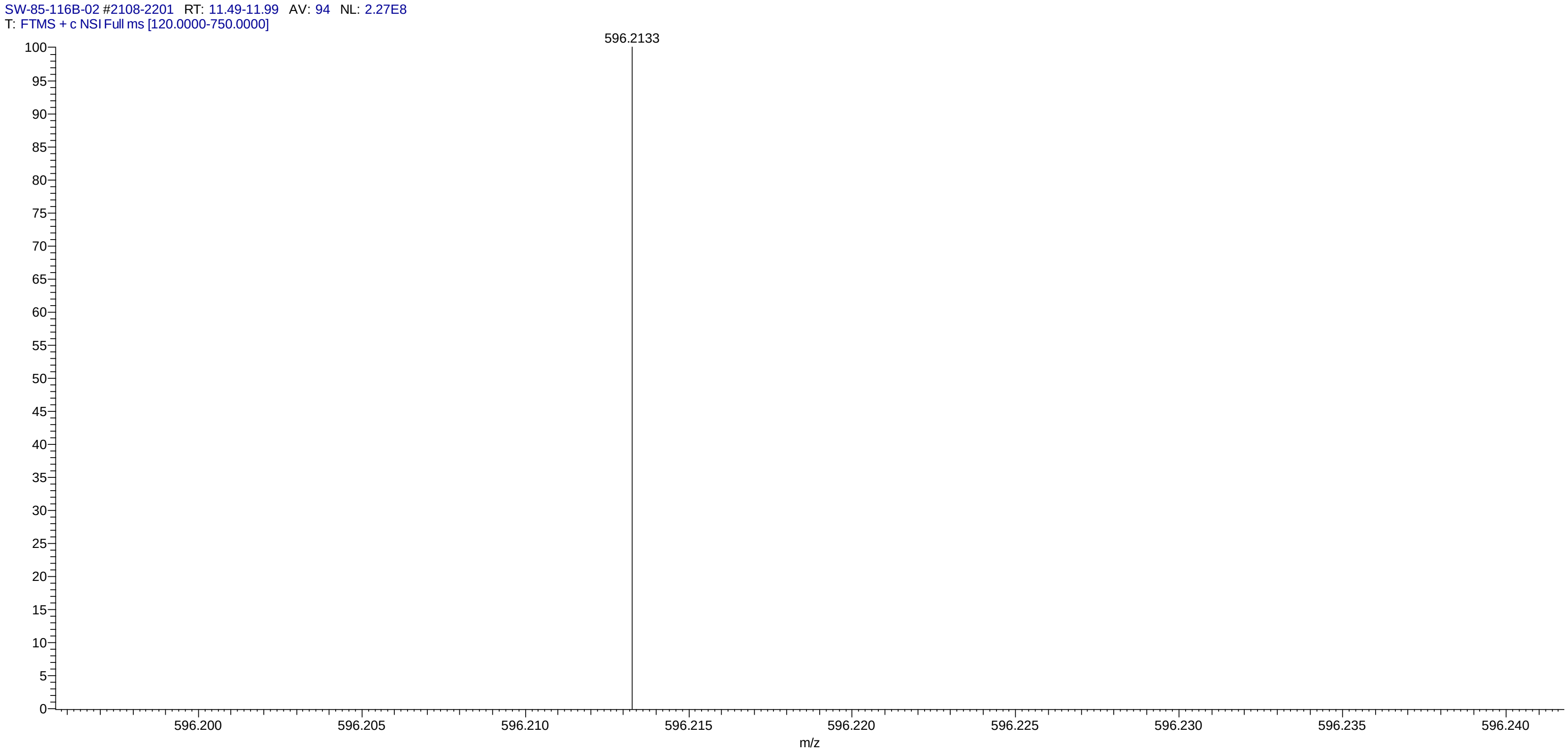

Figure S143: High resolution mass spectrum of **6B**

Injection Date : 4/19/2022 4:52:04 PM
Sample Name' : SW-85-116B-02 Location : Vial 1
Acq. Operator :
Method : C:\HPCHEM\1\METHODS\JNP2015.M
Last changed : 4/19/2022 12:01:10 PM
(modified after loading)

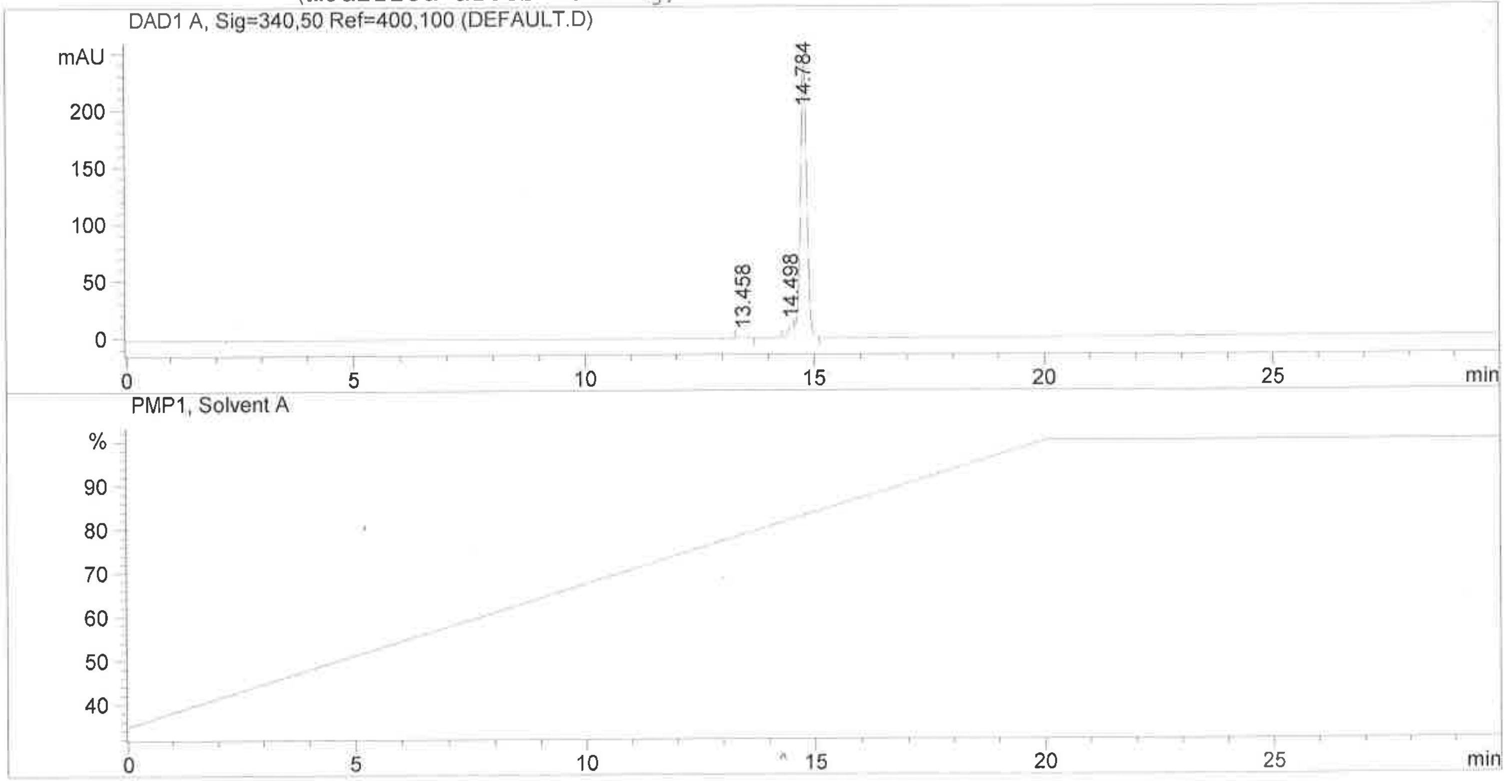

Area Percent Report

Sorted By : Signal
Multiplier : 1.0000
Dilution : 1.0000

Signal 1: DAD1 A, Sig=340,50 Ref=400,100

Peak #	RetTime [min]	Type	Width [min]	Area [mAU*s]	Height [mAU]	Area %
1	13.458	BB	0.1464	25.09278	2.55986	1.0332
2	14.498	BV	0.1194	91.06142	11.37198	3.7495
3	14.784	VB	0.1411	2312.44775	247.18042	95.2172

Totals : 2428.60195 261.11226

Results obtained with enhanced integrator!

*** End of Report ***

Figure S144: HPLC chromatogram of **6B**

SW-85-116A-01.3.fid

Figure S145: ^{1}H NMR spectrum of **7A** in $CDCl_3$

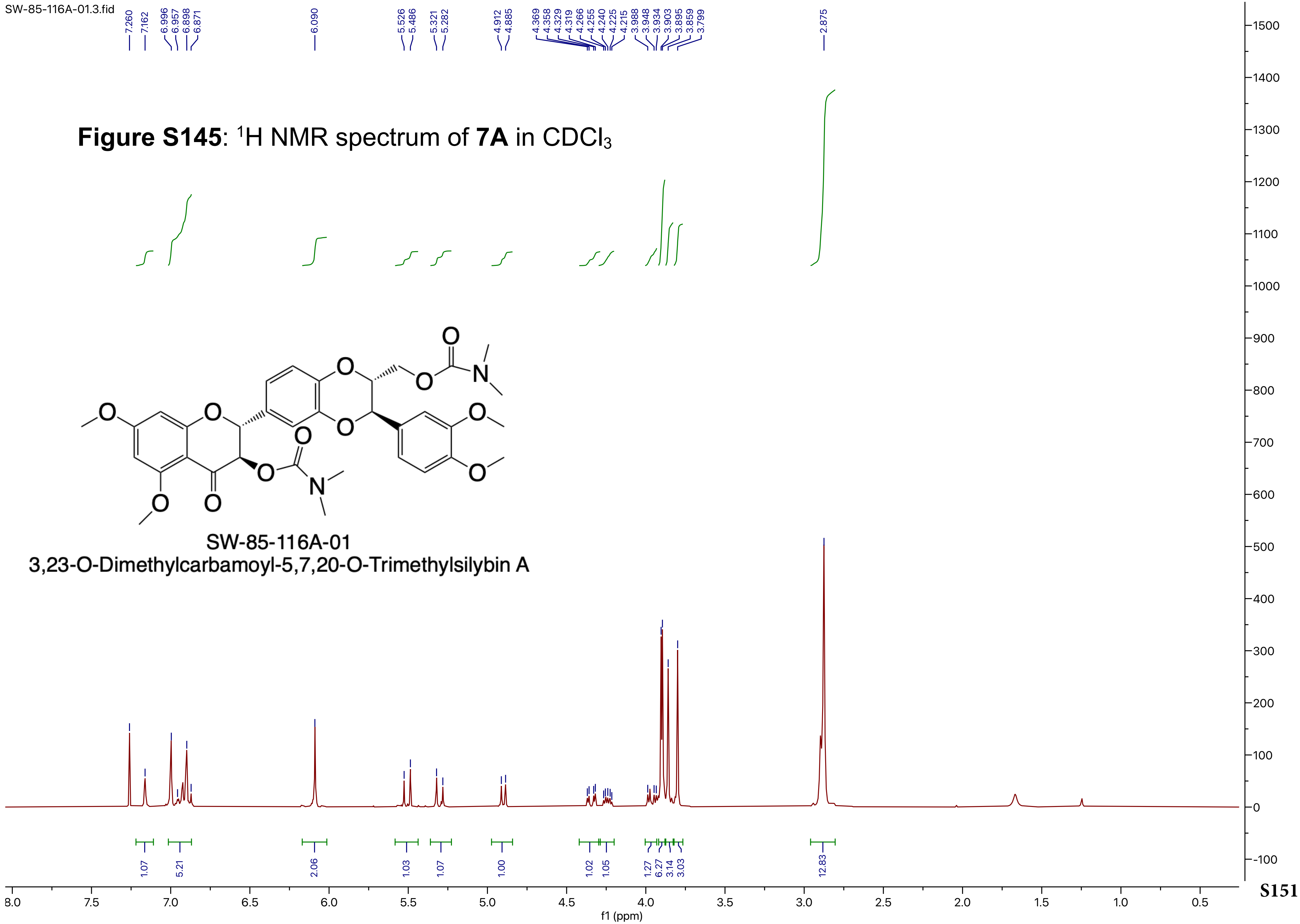

SW-85-116A-014.fid

Figure S146: ^{13}C NMR spectrum of **7A** in $CDCl_3$

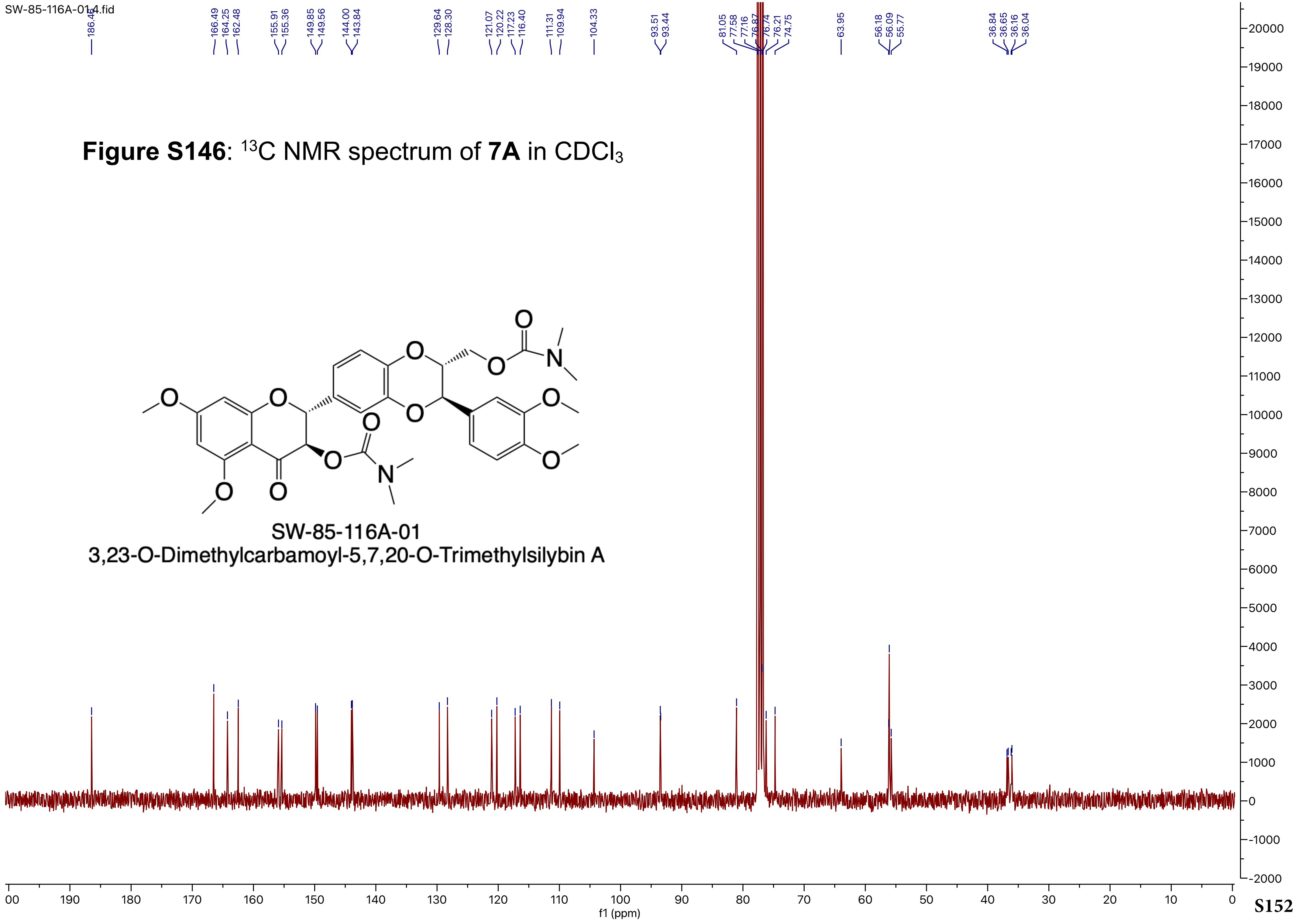

Sample Name	Mol Fomula	MW	M+H	observed	delta	ppm
SW-85-116A-01	C34H38N2O12	666.2425	667.2503	667.2499	-0.0004	-0.66

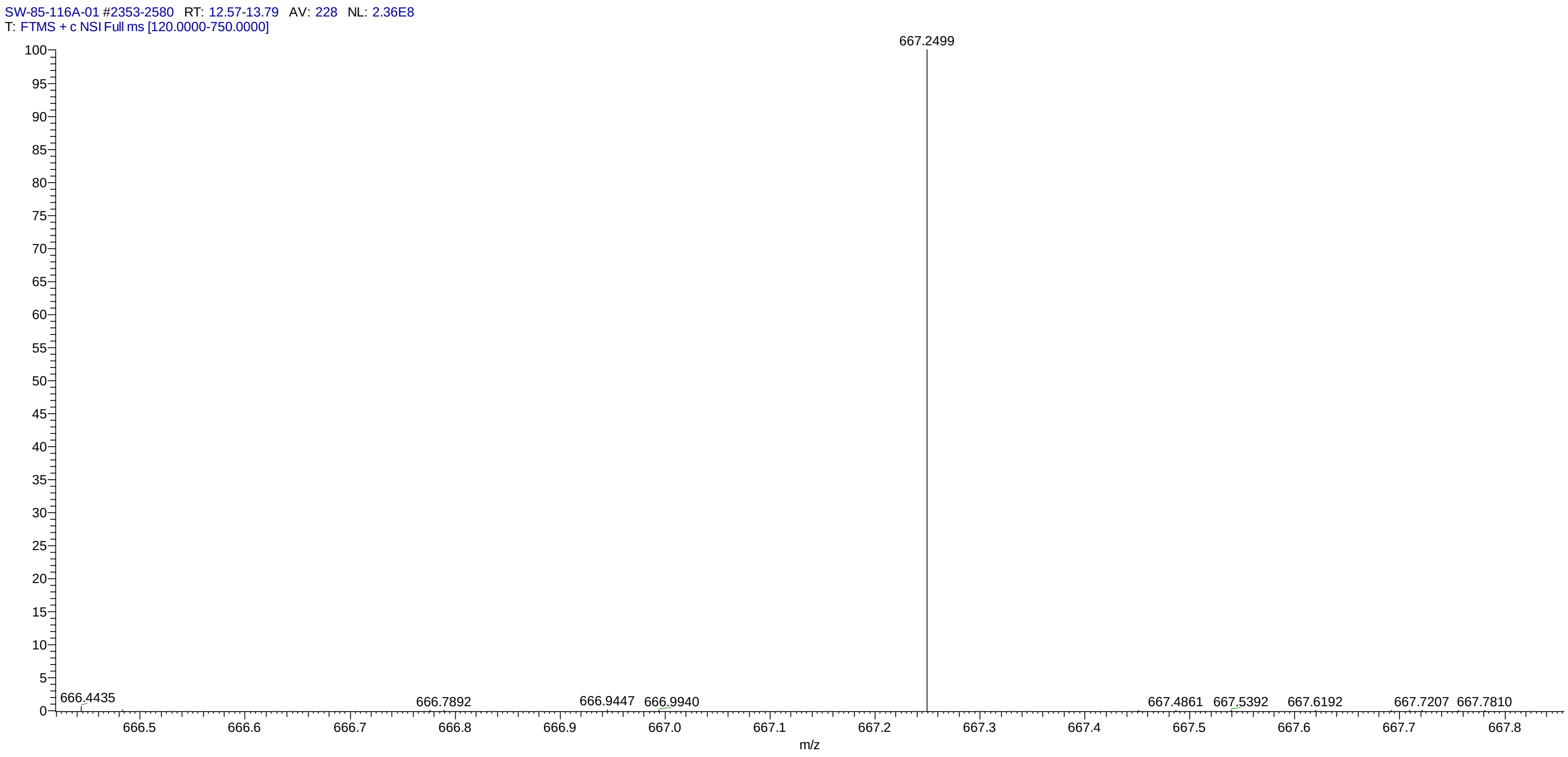

Figure S147: High resolution mass spectrum of **7A**

===

Injection Date : 4/20/2022 4:23:32 PM
Sample Name : SW-85-116A-01 Location : Vial 1
Acq. Operator :
Acq. Method : C:\HPCHEM\1\METHODS\JNP2015.M
Last changed : 4/20/2022 3:39:29 PM
Analysis Method : C:\HPCHEM\1\METHODS\JNP2015.M
Last changed : 4/20/2022 5:00:25 PM
(modified after loading)

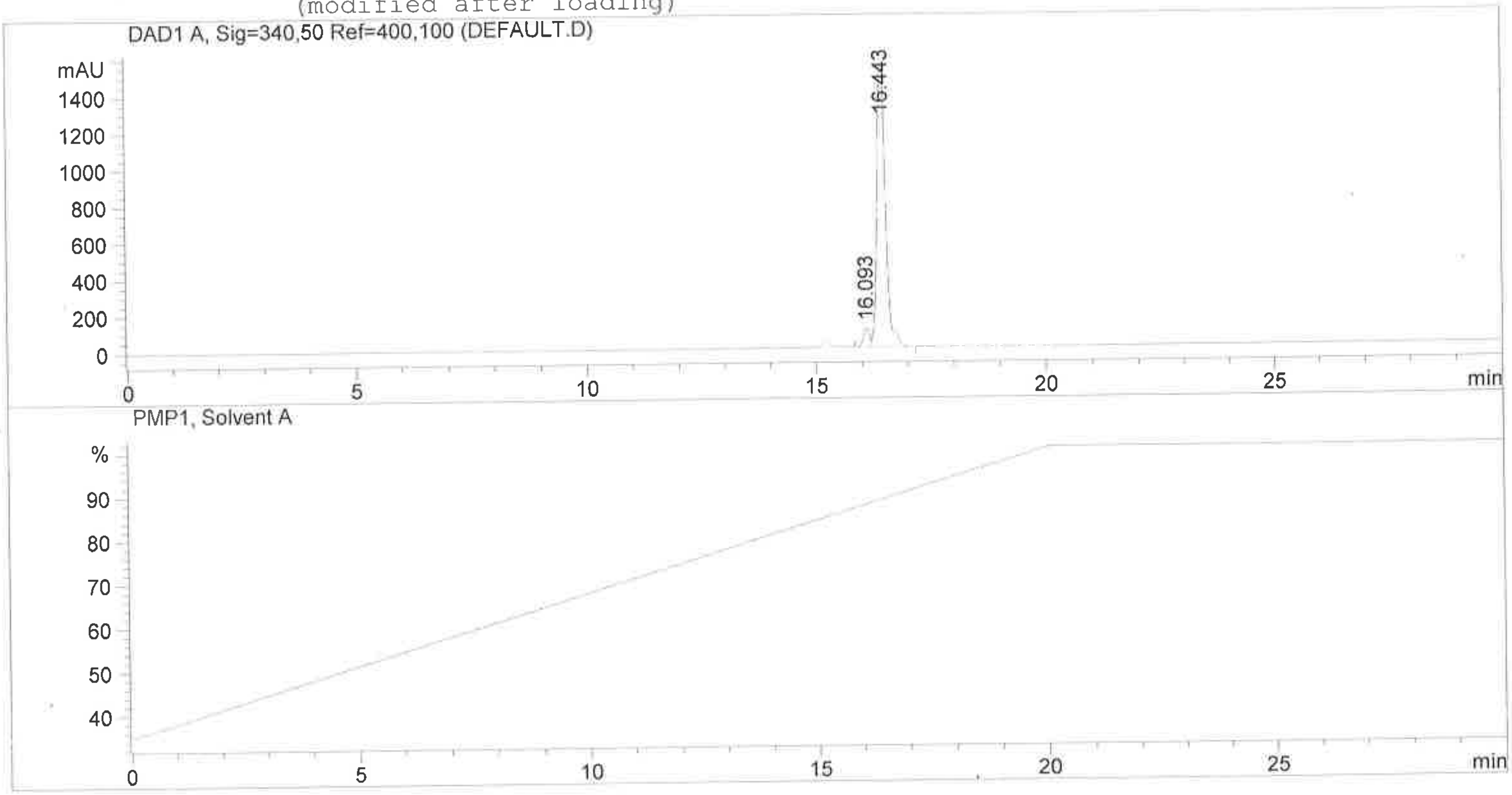

===

Area Percent Report

===

Sorted By : Signal
Multiplier : 1.0000
Dilution : 1.0000

Signal 1: DAD1 A, Sig=340,50 Ref=400,100

Peak #	RetTime [min]	Type	Width [min]	Area [mAU*s]	Height [mAU]	Area %
1	16.093	VV	0.1436	952.72302	110.56577	4.5209
2	16.443	VV	0.2165	2.01212e4	1548.75269	95.4791

Totals : 2.10739e4 1659.31846

Results obtained with enhanced integrator!

===

*** End of Report ***

Figure S148: HPLC chromatogram of **7A**

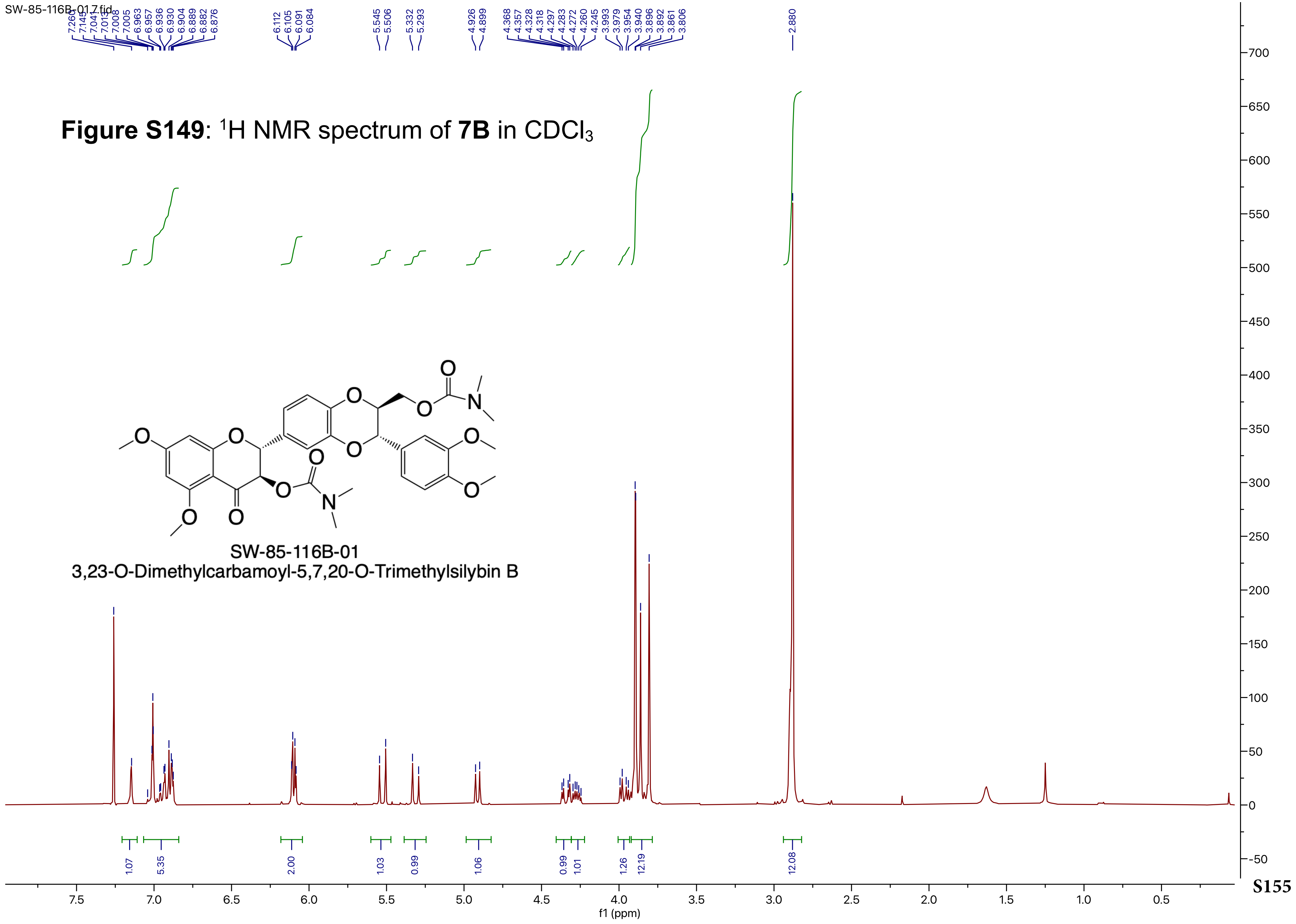

Figure S149: ^{1}H NMR spectrum of **7B** in $CDCl_3$

SW-85-116B-01
3,23-O-Dimethylcarbamoyl-5,7,20-O-Trimethylsilybin B

Figure S150: ^{13}C NMR spectrum of **7B** in $CDCl_3$

SW-85-116B-01
3,23-O-Dimethylcarbamoyl-5,7,20-O-Trimethylsilybin B

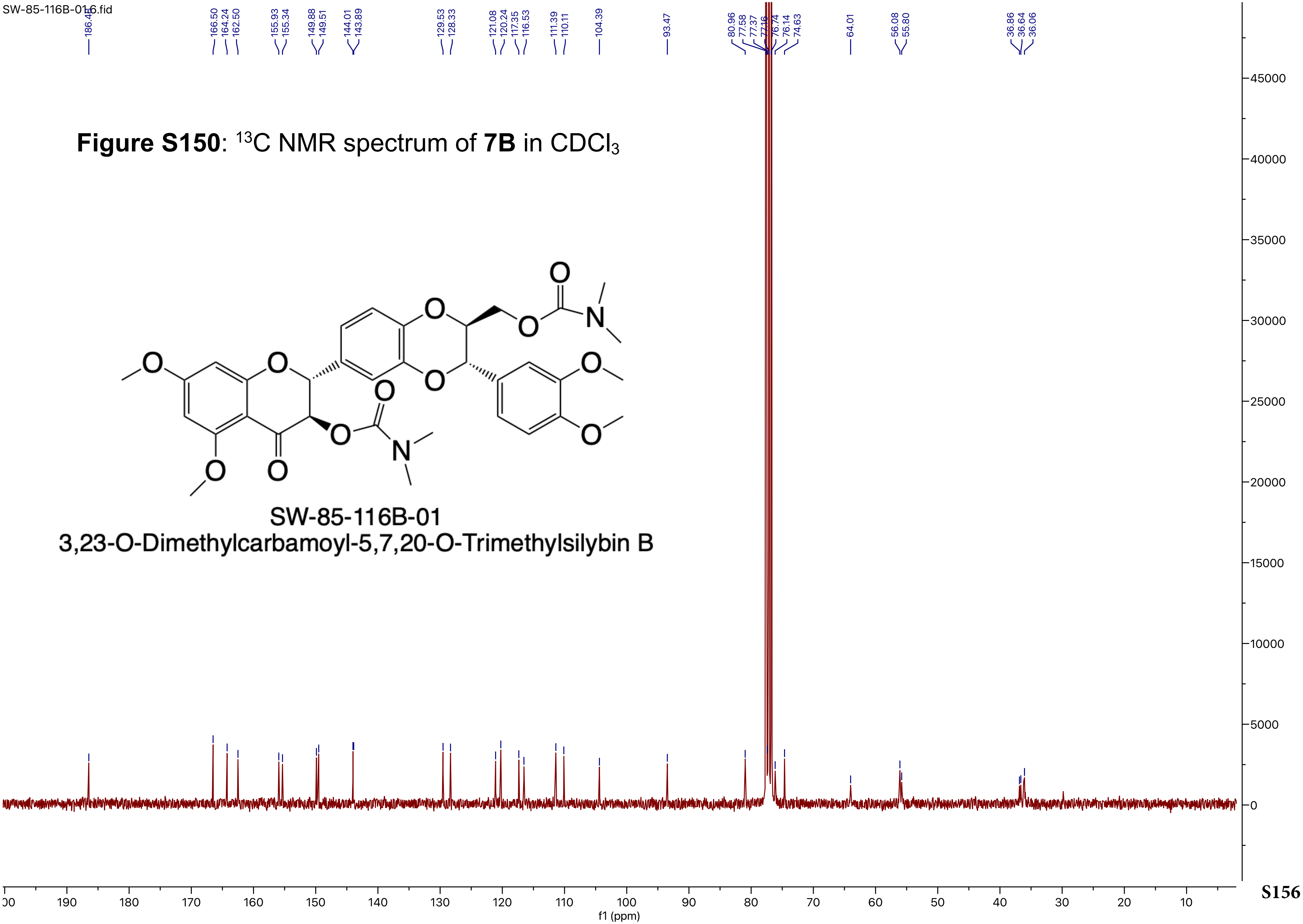

Sample Name	Mol Fomula	MW	M+H	observed	delta	ppm
SW-85-116B-01	C34H38N2O12	666.2425	667.2503	667.2501	-0.0002	-0.36

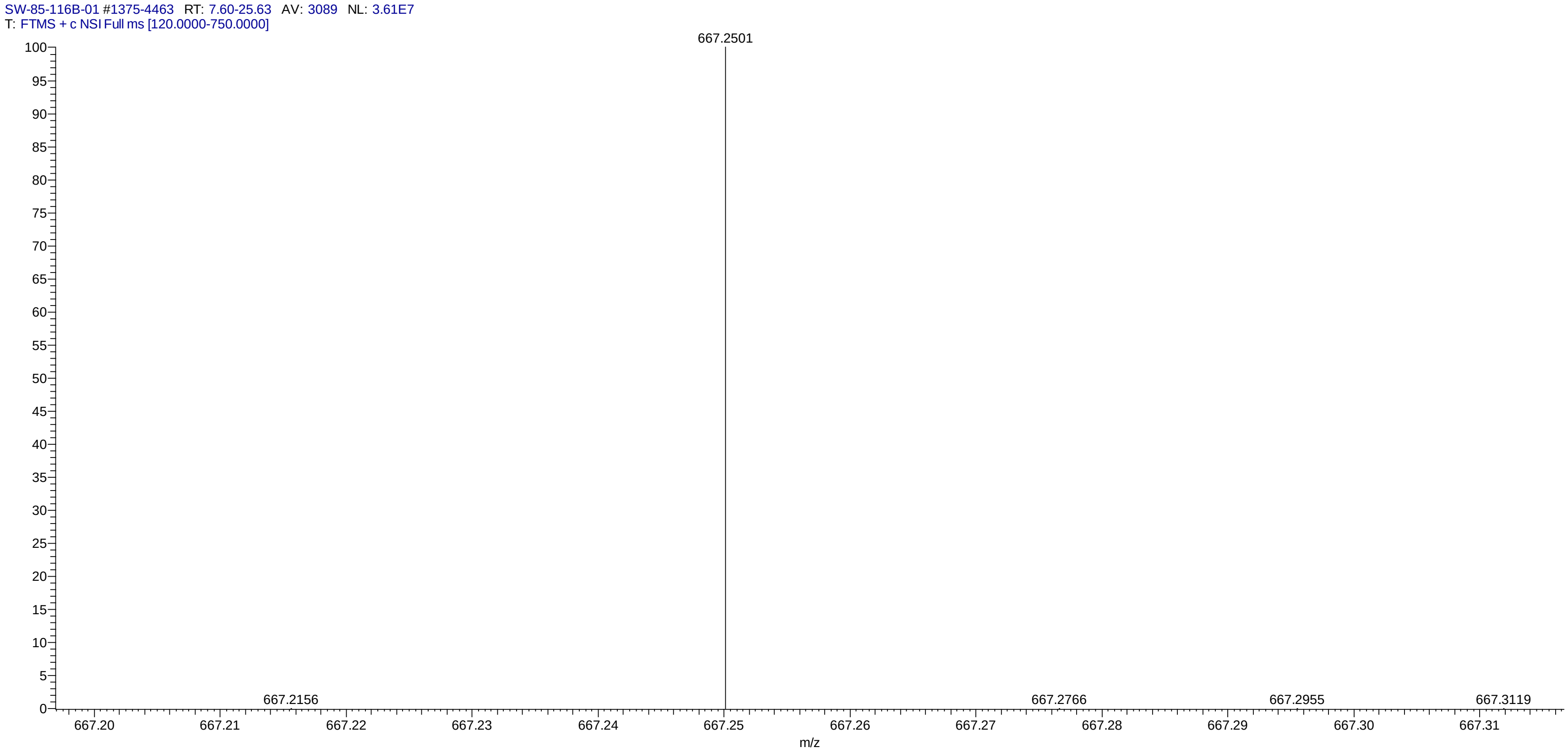

Figure S151: High resolution mass spectrum of **7B**

===

Injection Date : 4/17/2022 2:04:16 PM
Sample Name : SW-85-116B-01 Location : Vial 1
Acq. Operator :
Method : C:\HPCHEM\1\METHODS\JNP2015.M
Last changed : 4/16/2022 9:32:45 AM
(modified after loading)

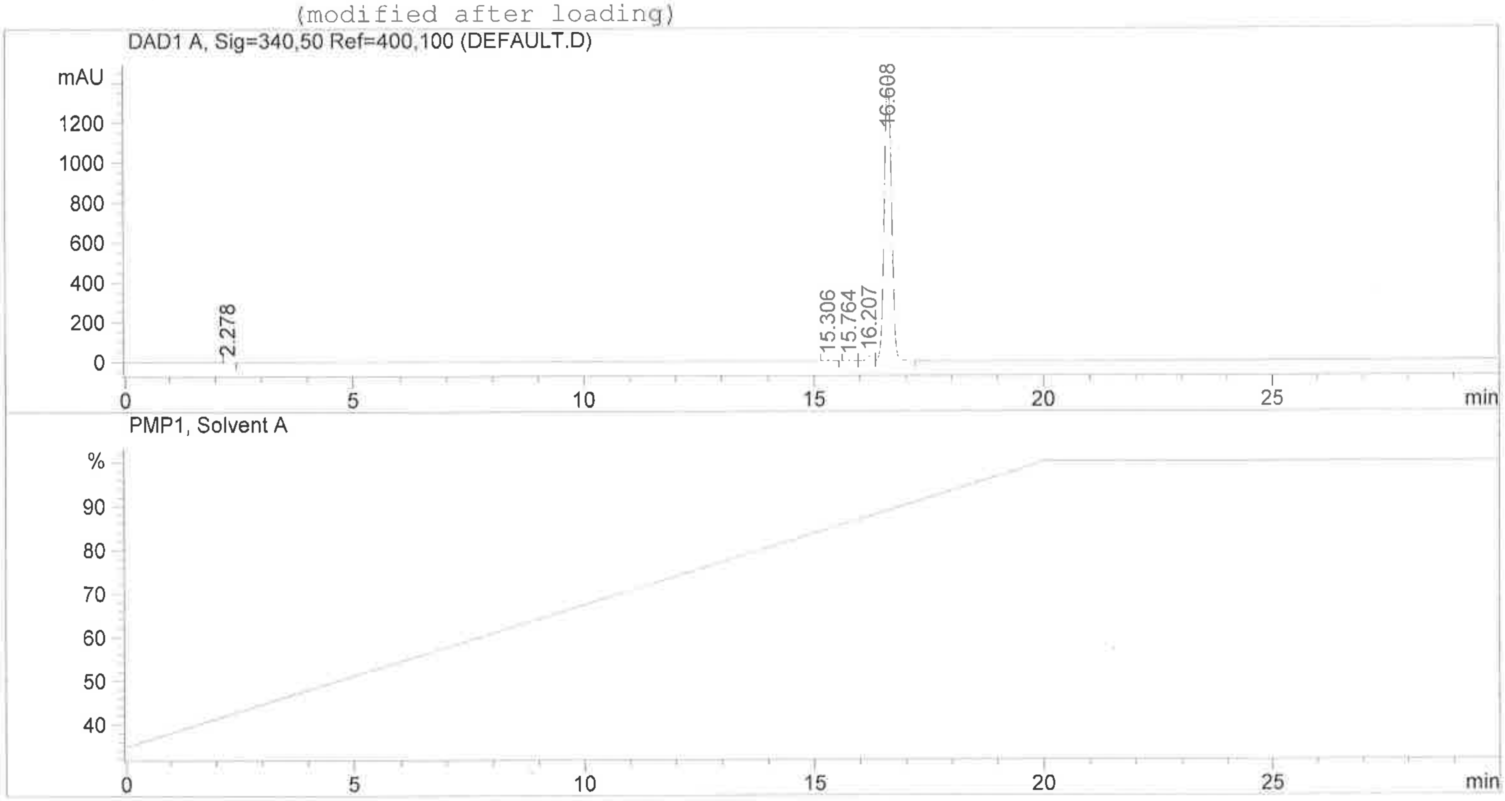

===

Area Percent Report

===

Sorted By : Signal
Multiplier : 1.0000
Dilution : 1.0000

Signal 1: DAD1 A, Sig=340,50 Ref=400,100

Peak #	RetTime [min]	Type	Width [min]	Area [mAU*s]	Height [mAU]	Area %
1	2.278	PB	0.1081	8.38715	1.19145	0.0538
2	15.306	BB	0.1342	19.13877	2.18386	0.1227
3	15.764	BP	0.1302	17.89757	2.08451	0.1148
4	16.207	VV	0.1309	195.76437	23.08830	1.2556
5	16.608	VB	0.1700	1.53507e4	1421.47668	98.4531
Totals :				1.55919e4	1450.02481	

Results obtained with enhanced integrator!

===

*** End of Report ***

Figure S152: HPLC chromatogram of 7B

Figure S153: ^{1}H NMR spectrum in $CDCl_3$

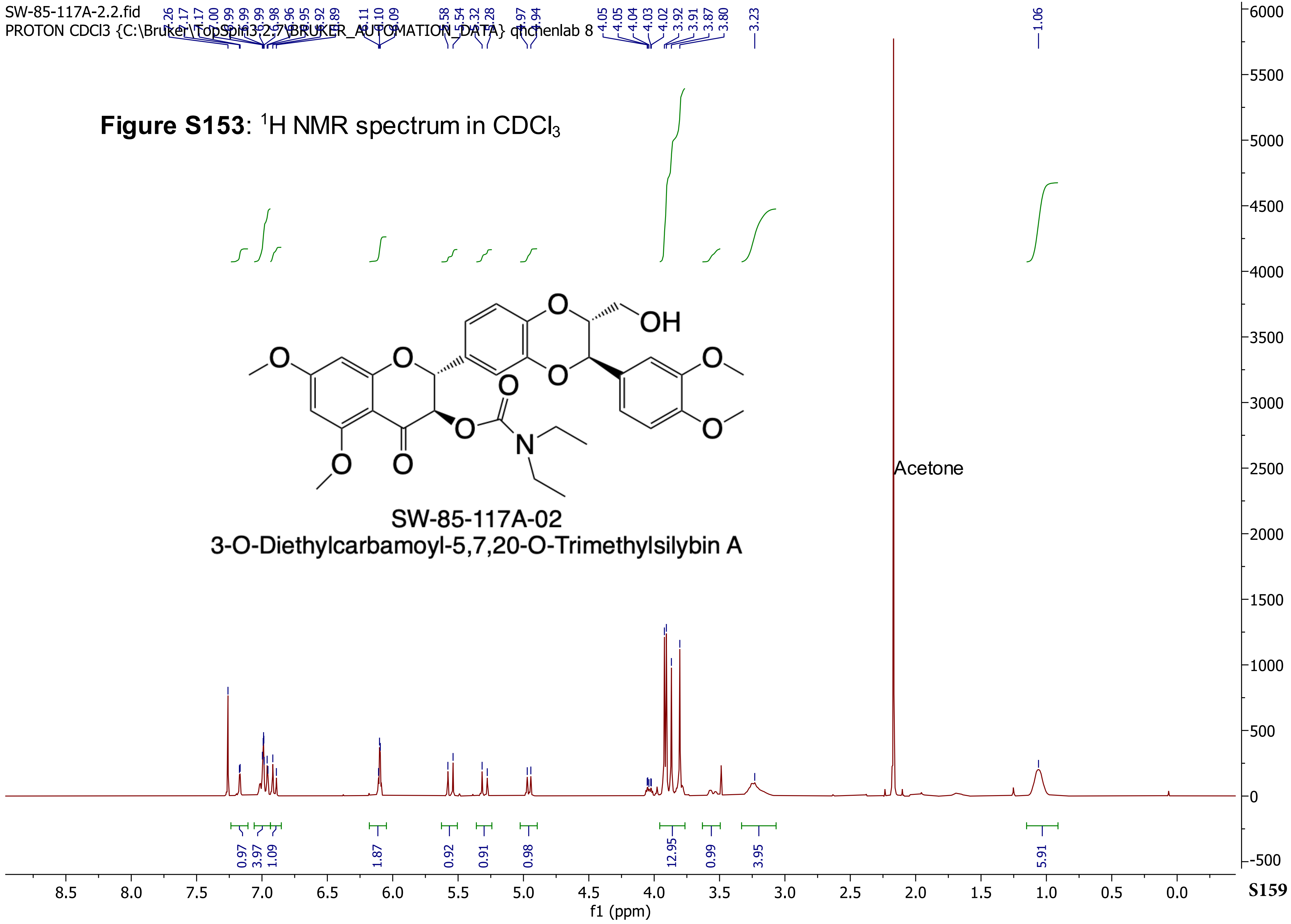

Figure S154: ^{13}C NMR spectrum of **8A** in $CDCl_3$

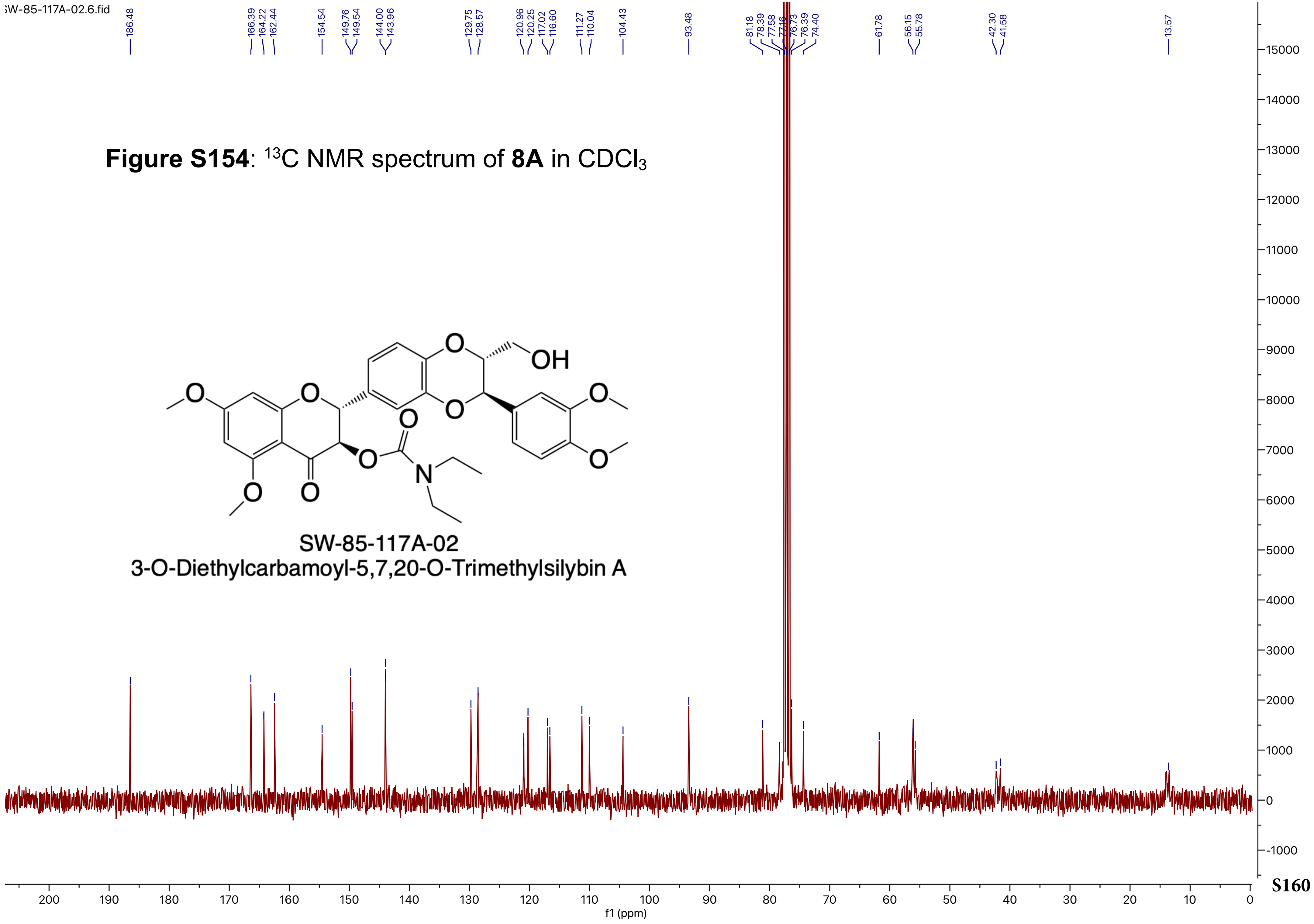

Sample Name	Mol Fomula	MW	M+H	observed	delta	ppm
SW-85-117A-02	C33H37NO11	623.2367	624.2445	624.2440	-0.0005	-0.82

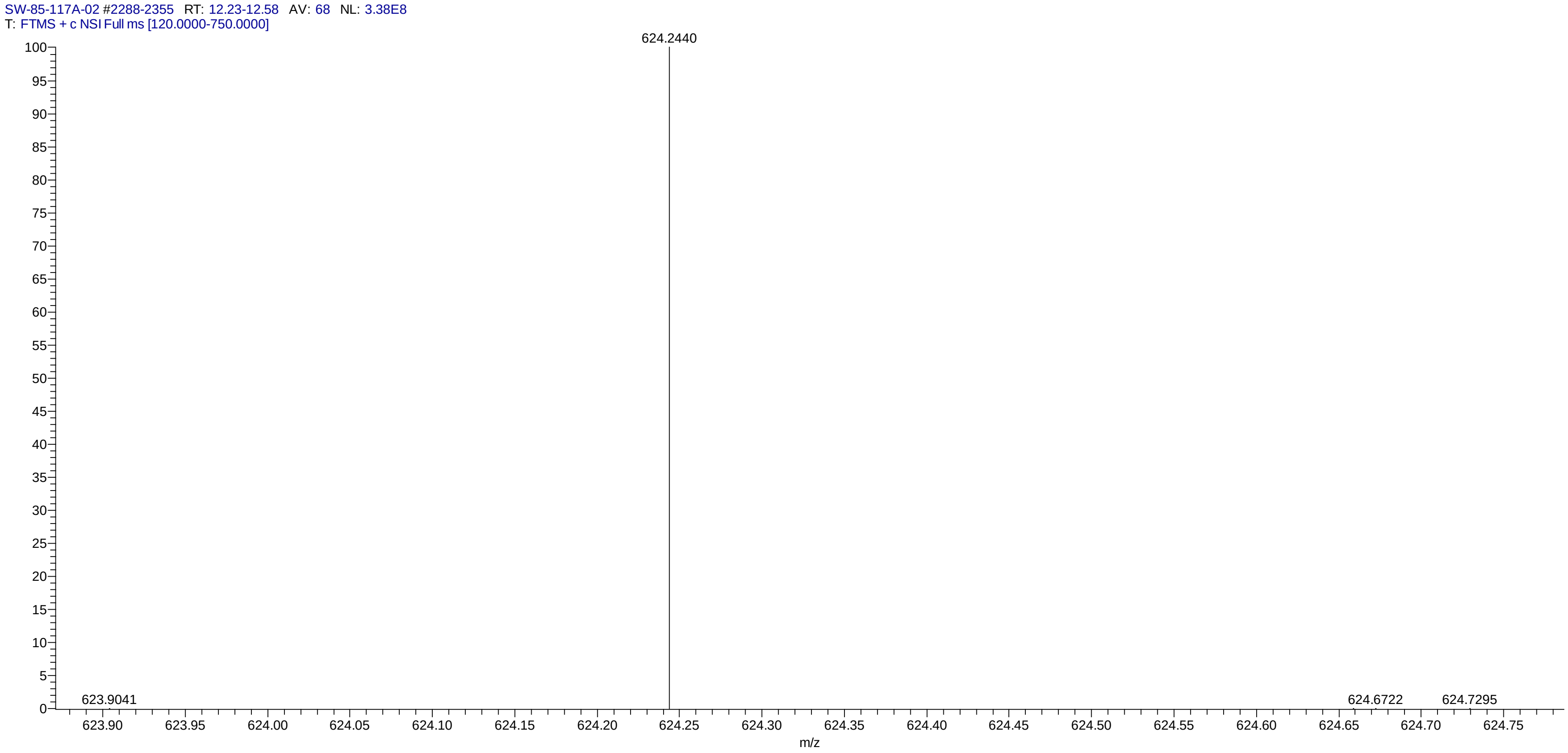

Figure S155: High resolution mass spectrum of **8A**

===

Injection Date : 2/19/2023 12:58:40 PM
Sample Name : SW-85-117A-02 Location : Vial 1
Acq. Operator :
Method : C:\HPCHEM\1\METHODS\JNP2015.M
Last changed : 2/9/2023 3:27:35 PM

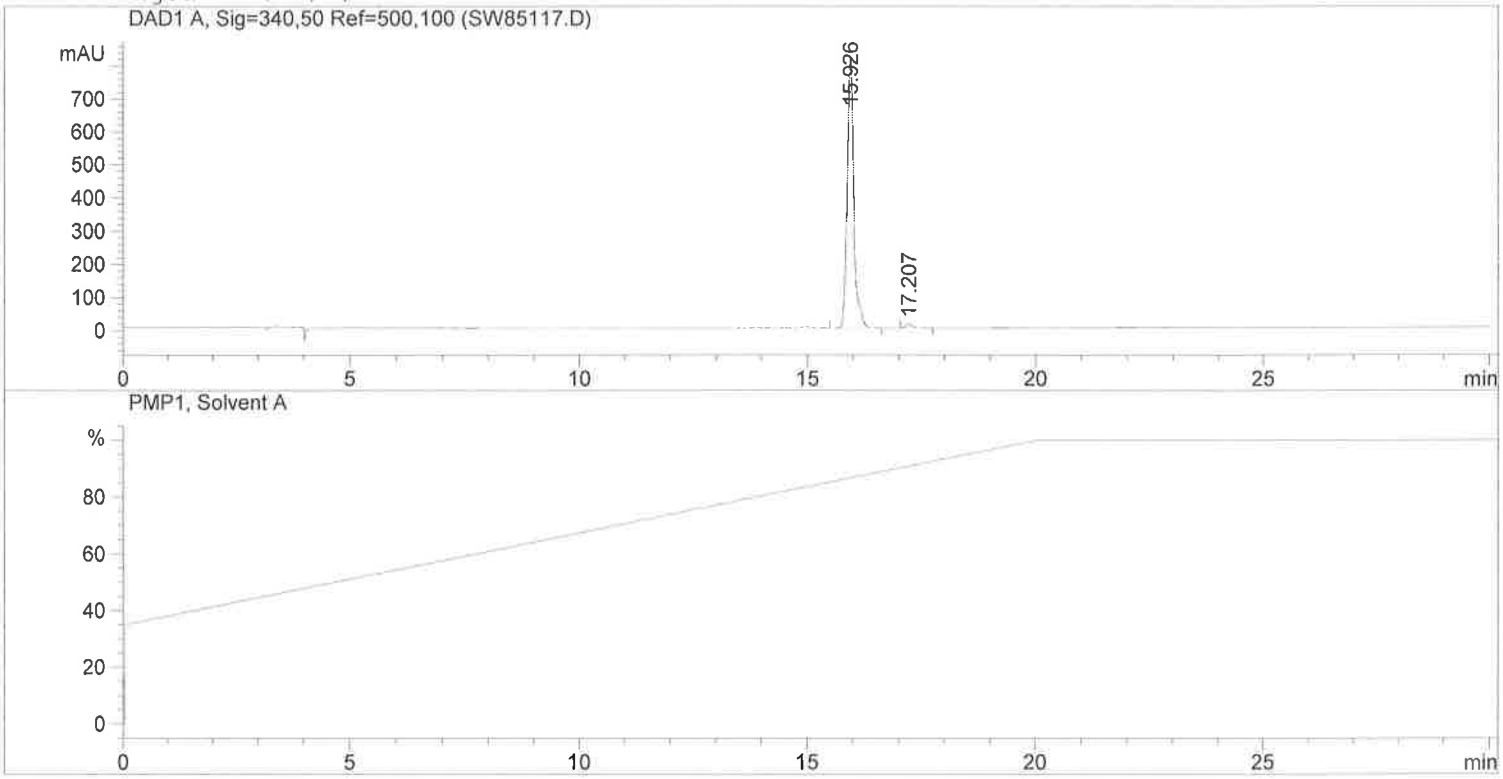

===
Area Percent Report
===

Sorted By : Signal
Multiplier : 1.0000
Dilution : 1.0000

Signal 1: DAD1 A, Sig=340,50 Ref=500,100

Peak #	RetTime [min]	Type	Width [min]	Area [mAU*s]	Height [mAU]	Area %
1	15.926	VP	0.1535	8262.76660	820.12885	98.5428
2	17.207	PP	0.1377	122.18180	12.98965	1.4572
Totals :				8384.94840	833.11850	

Results obtained with enhanced integrator!

===
*** End of Report ***

Figure S156: HPLC chromatogram of **8A**

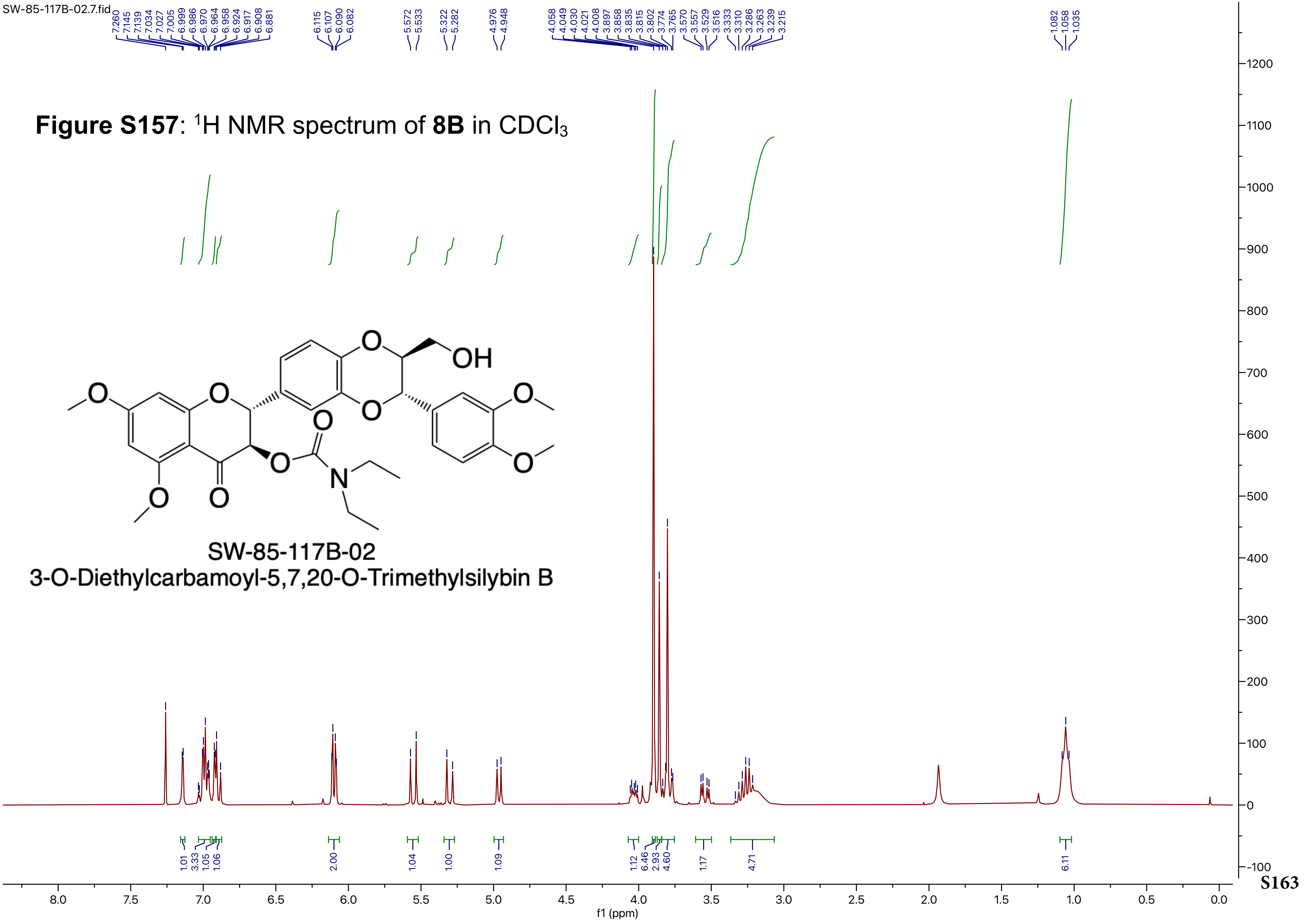

Figure S157: ^{1}H NMR spectrum of **8B** in $CDCl_3$

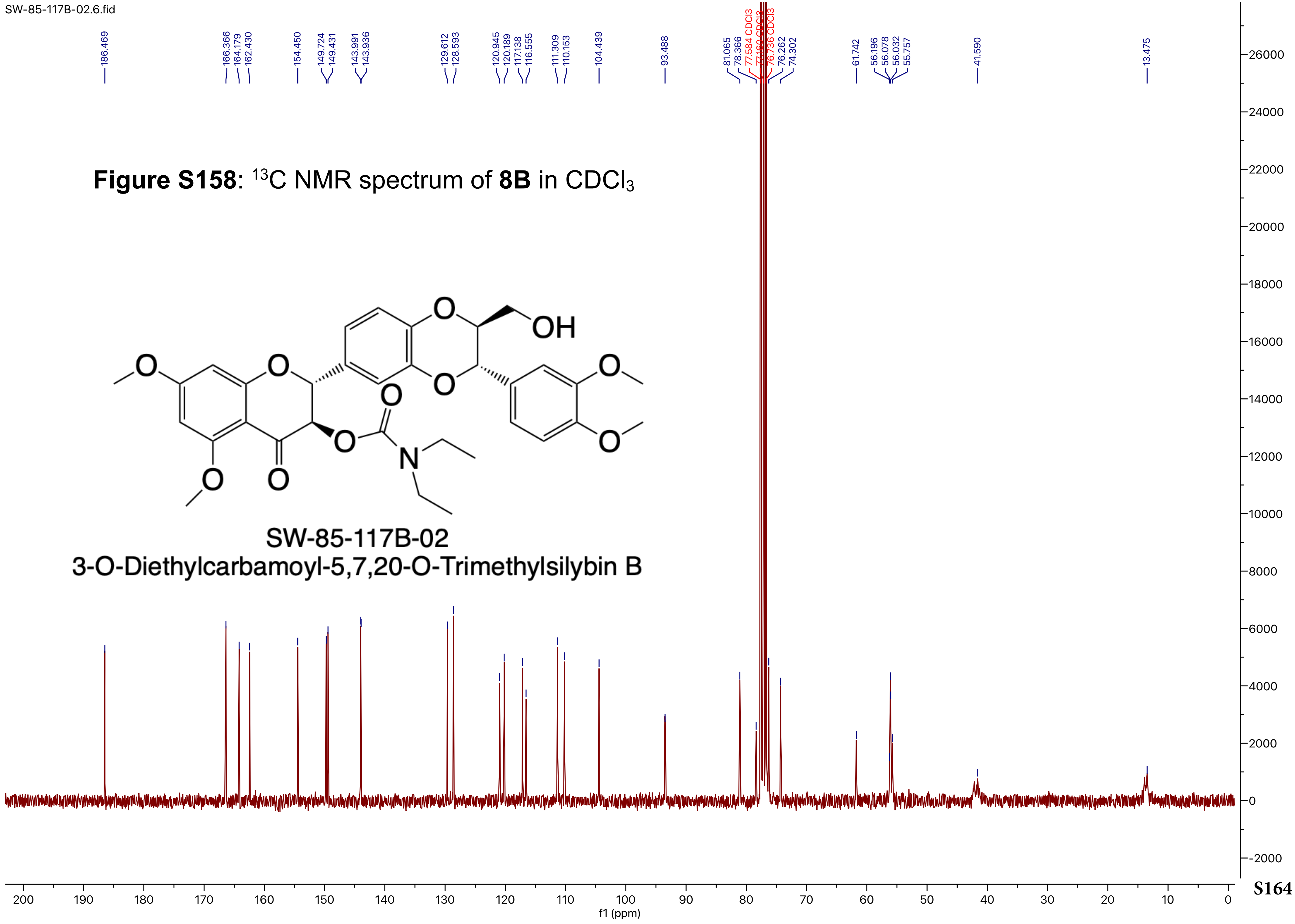

Figure S158: ^{13}C NMR spectrum of **8B** in $CDCl_3$

Sample Name	Mol Fomula	MW	M+H	observed	delta	ppm
SW-85-117B-02	C33H37NO11	623.2367	624.2445	624.2441	-0.0004	-0.66

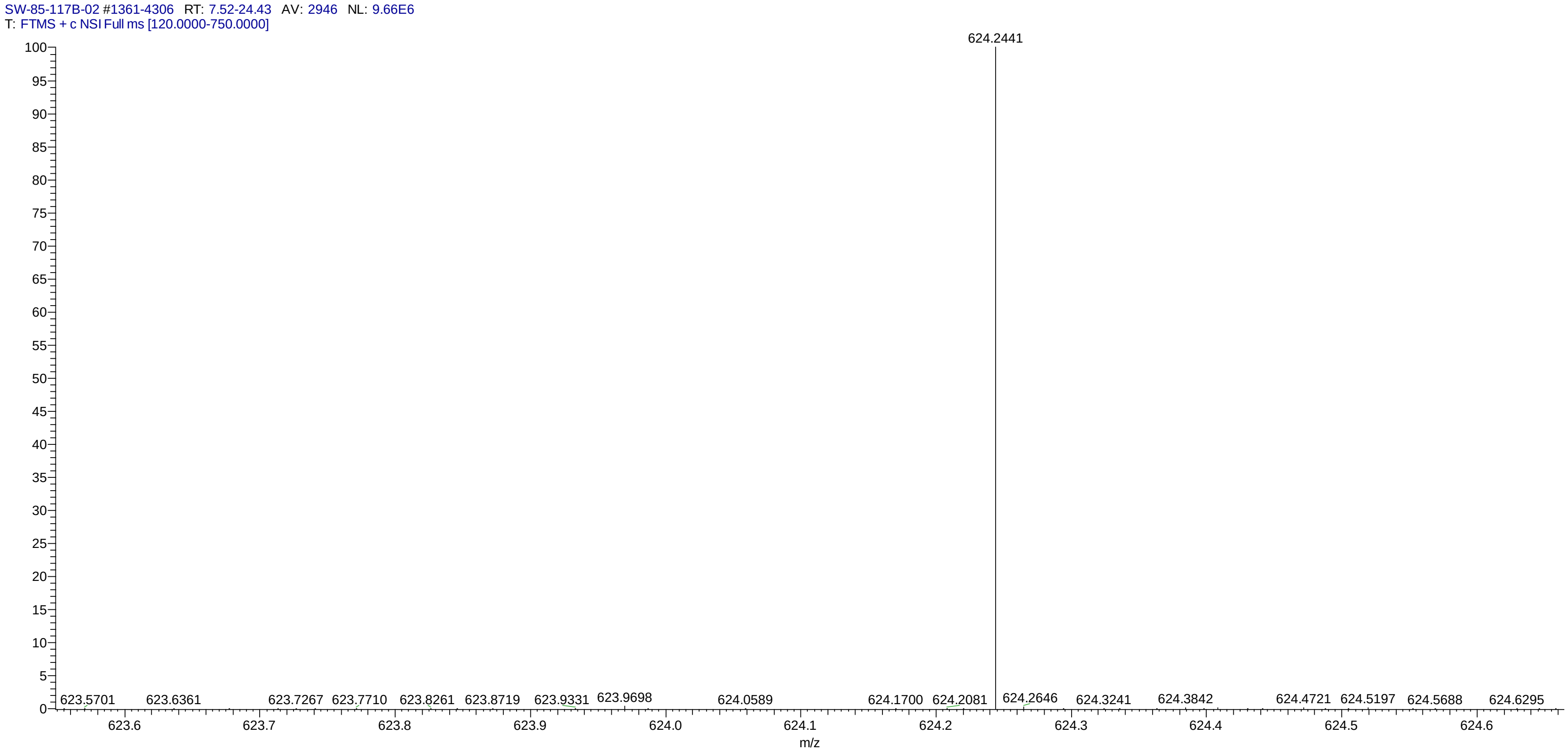

Figure S159: High resolution mass spectrum of **8B**

Data File C:\HPCHEM\1\DATA\DEFAULT.D Sample Name: SW-85-117B-02

Injection Date : 4/17/2022 10:57:16 AM
Sample Name : SW-85-117B-02 Location : Vial 1
Acq. Operator :
Method : C:\HPCHEM\1\METHODS\JNP2015.M
Last changed : 4/16/2022 9:32:45 AM
(modified after loading)

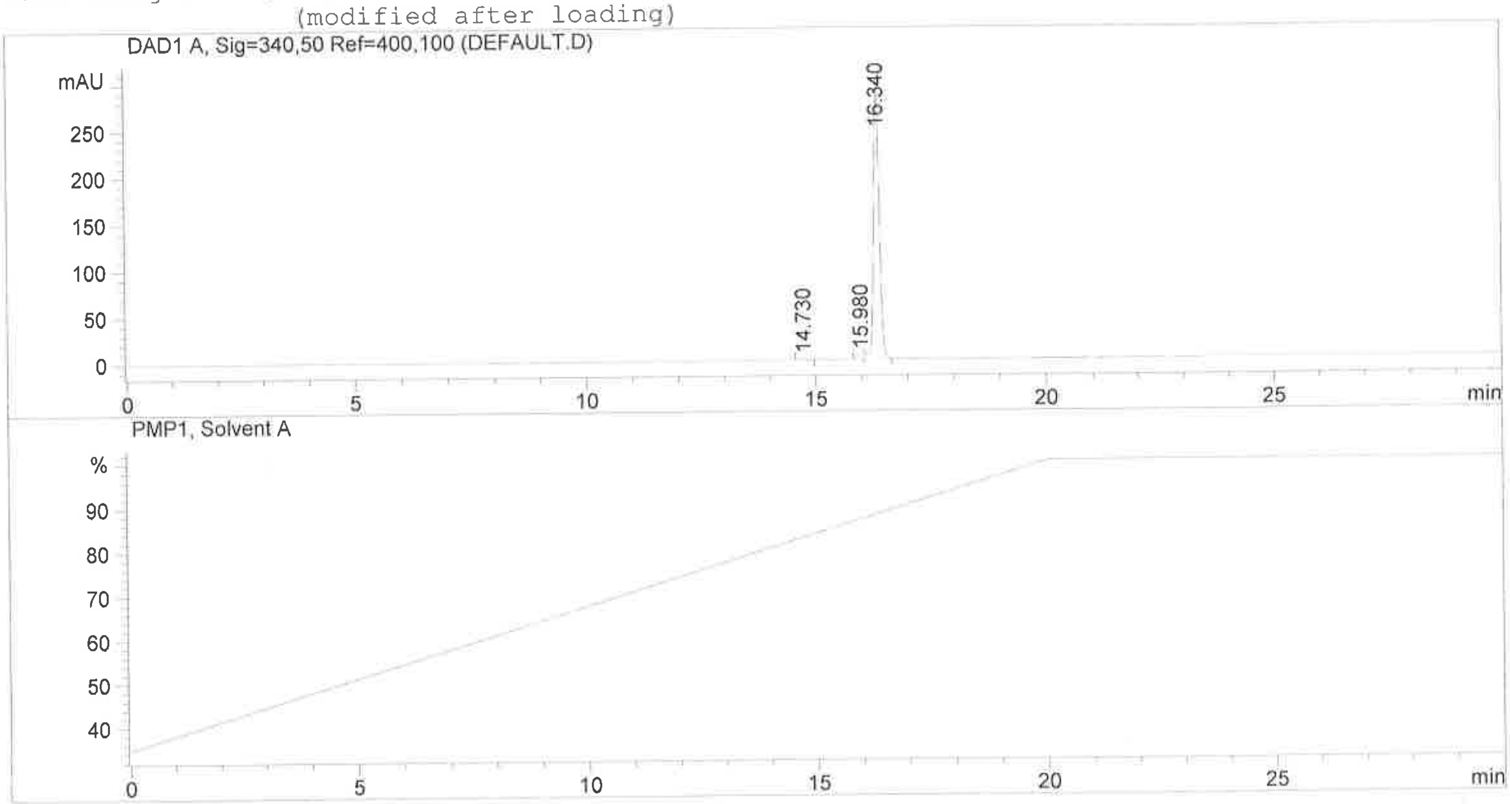

Area Percent Report

Sorted By : Signal
Multiplier : 1.0000
Dilution : 1.0000

Signal 1: DAD1 A, Sig=340,50 Ref=400,100

Peak #	RetTime [min]	Type	Width [min]	Area [mAU*s]	Height [mAU]	Area %
1	14.730	BP	0.1469	11.30698	1.18976	0.4261
2	15.980	BV	0.1140	37.02183	4.90998	1.3952
3	16.340	VB	0.1320	2605.26831	303.84845	98.1787

Totals : 2653.59712 309.94818

Results obtained with enhanced integrator!

*** End of Report ***

Figure S160: HPLC chromatogram of **8B**

SW-85-118A.3.fid

Figure S161: ^{1}H NMR spectrum of **9A** in $CDCl_3$

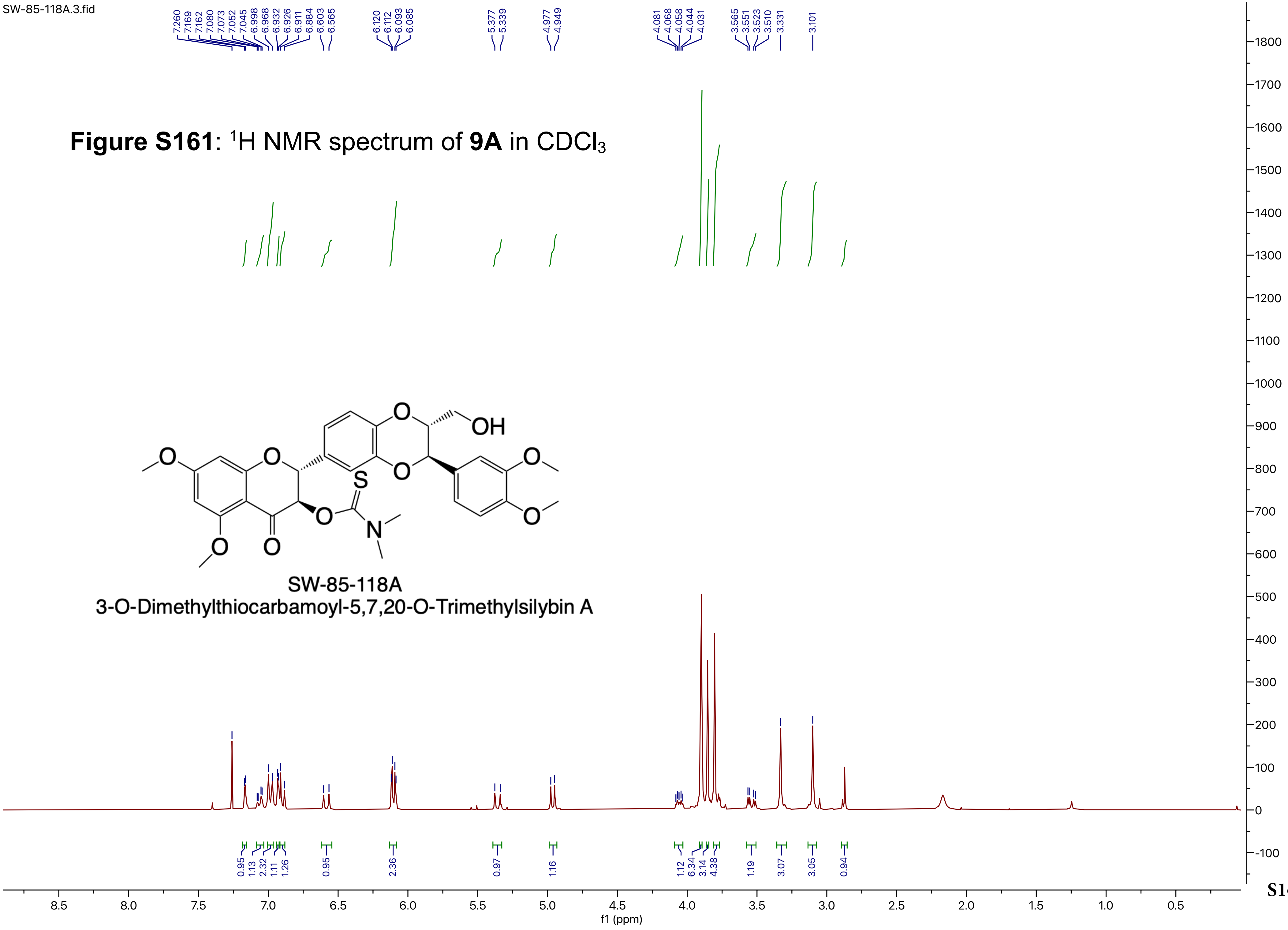

SW-85-118A

3-O-Dimethylthiocarbamoyl-5,7,20-O-Trimethylsilybin A

Figure S162: ^{13}C NMR spectrum of **9A** in $CDCl_3$

SW-85-118A
3-O-Dimethylthiocarbamoyl-5,7,20-O-Trimethylsilybin A

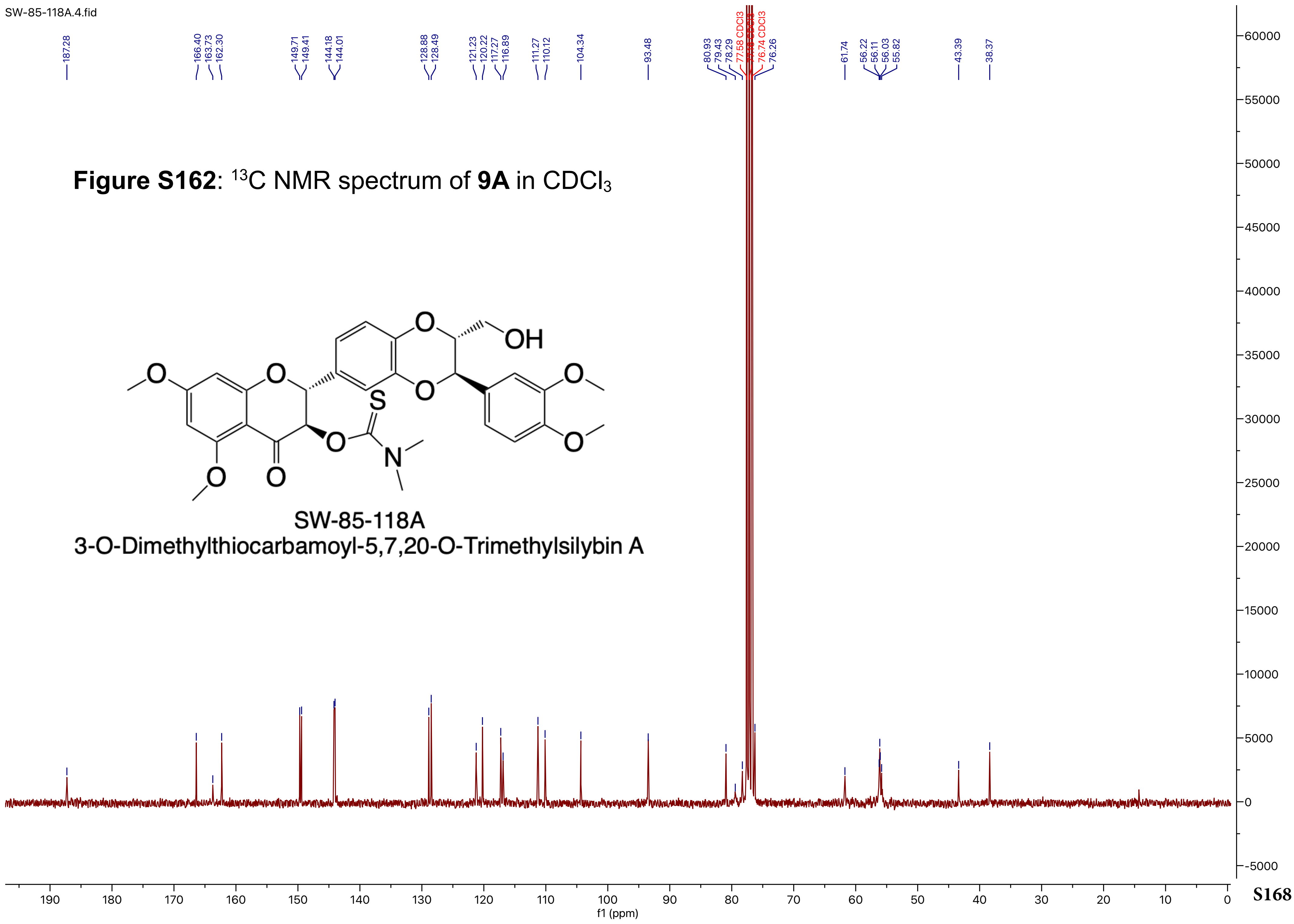

Sample Name	Mol Fomula	MW	M+H	observed	delta	ppm
SW-85-118A	C31H33NO10S	611.1826	612.1904	612.1902	-0.0002	-0.26

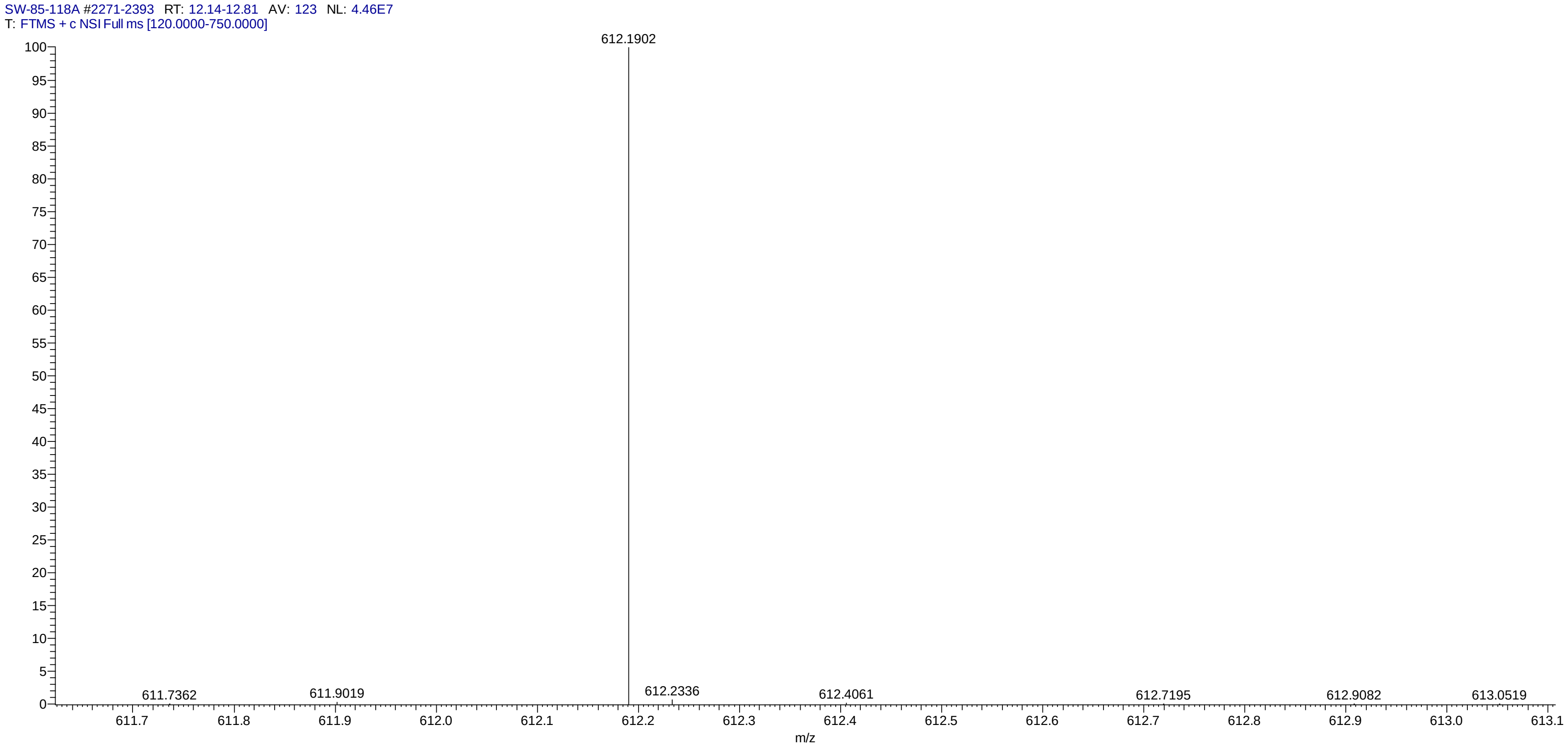

Figure S163: High resolution mass spectrum of **9A**

Injection Date : 4/20/2022 11:22:58 AM
Sample Name : SW-85-118A Location : Vial 1
Acq. Operator :
Method : C:\HPCHEM\1\METHODS\JNP2015.M
Last changed : 4/19/2022 12:01:10 PM
(modified after loading)

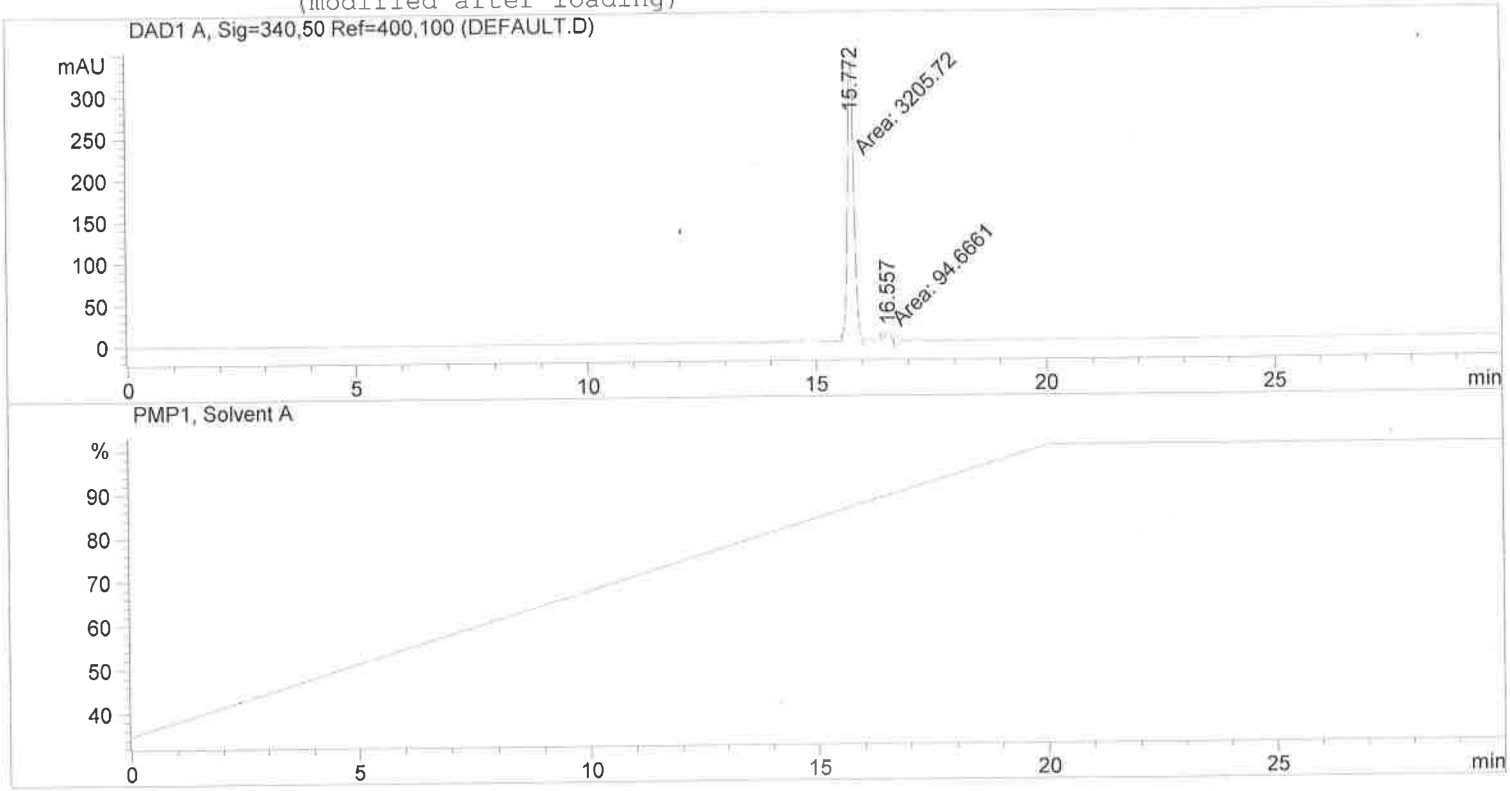

Area Percent Report

Sorted By : Signal
Multiplier : 1.0000
Dilution : 1.0000

Signal 1: DAD1 A, Sig=340,50 Ref=400,100

Peak #	RetTime [min]	Type	Width [min]	Area [mAU*s]	Height [mAU]	Area %
1	15.772	MM	0.1593	3205.72046	335.43436	97.1317
2	16.557	MM	0.1436	94.66611	10.98537	2.8683

Totals : 3300.38657 346.41973

Results obtained with enhanced integrator!

*** End of Report ***

Figure S164: HPLC chromatogram of **9A**

Figure S165: ^{1}H NMR spectrum of **9B** in $CDCl_3$

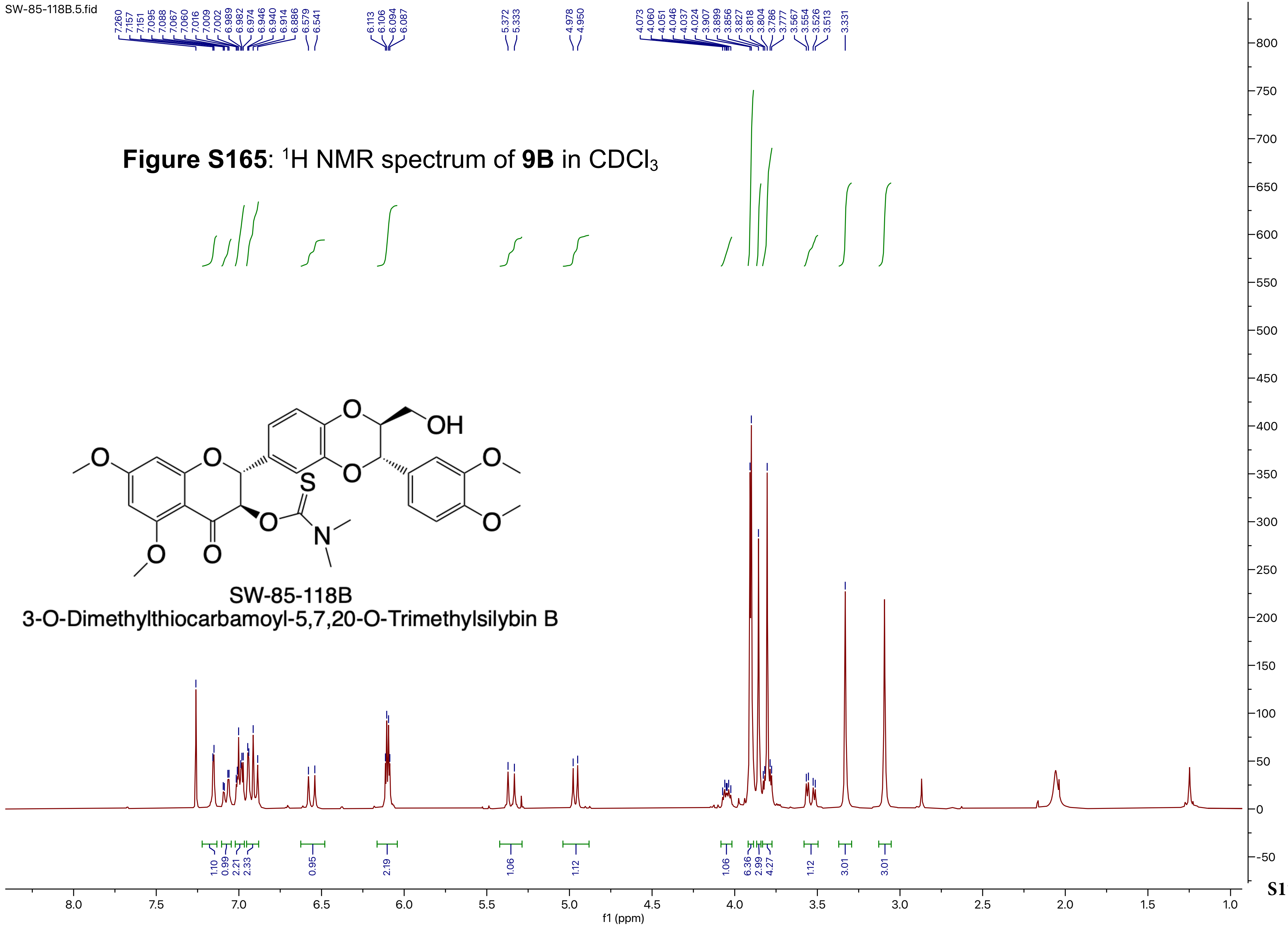

SW-85-118B

3-O-Dimethylthiocarbamoyl-5,7,20-O-Trimethylsilybin B

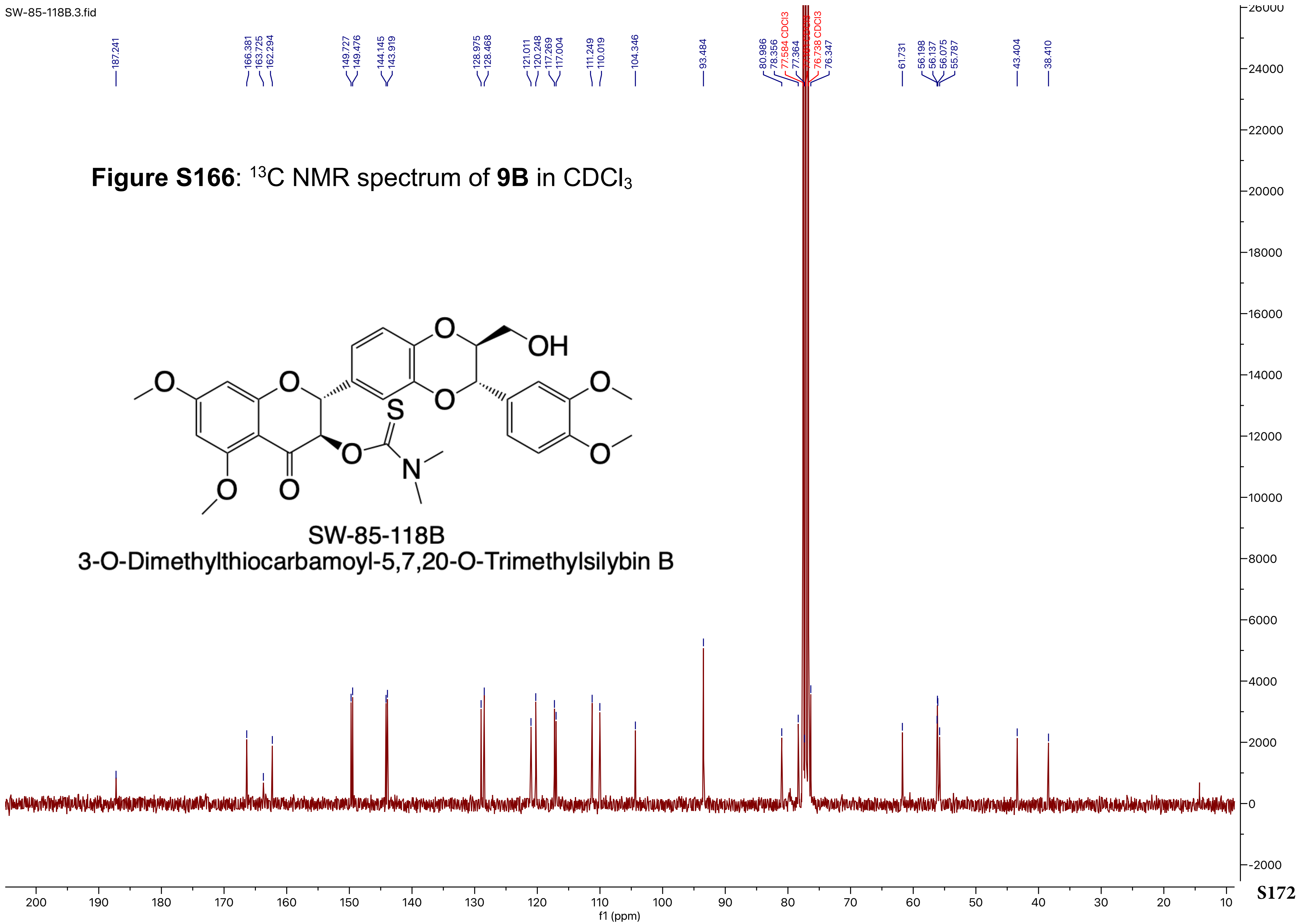

Figure S166: ^{13}C NMR spectrum of **9B** in $CDCl_3$

SW-85-118B

3-O-Dimethylthiocarbamoyl-5,7,20-O-Trimethylsilybin B

Sample Name	Mol Fomula	MW	M+H	observed	delta	ppm
SW-85-118B	C31H33NO10S	611.1826	612.1904	612.1904	0.0000	0.00

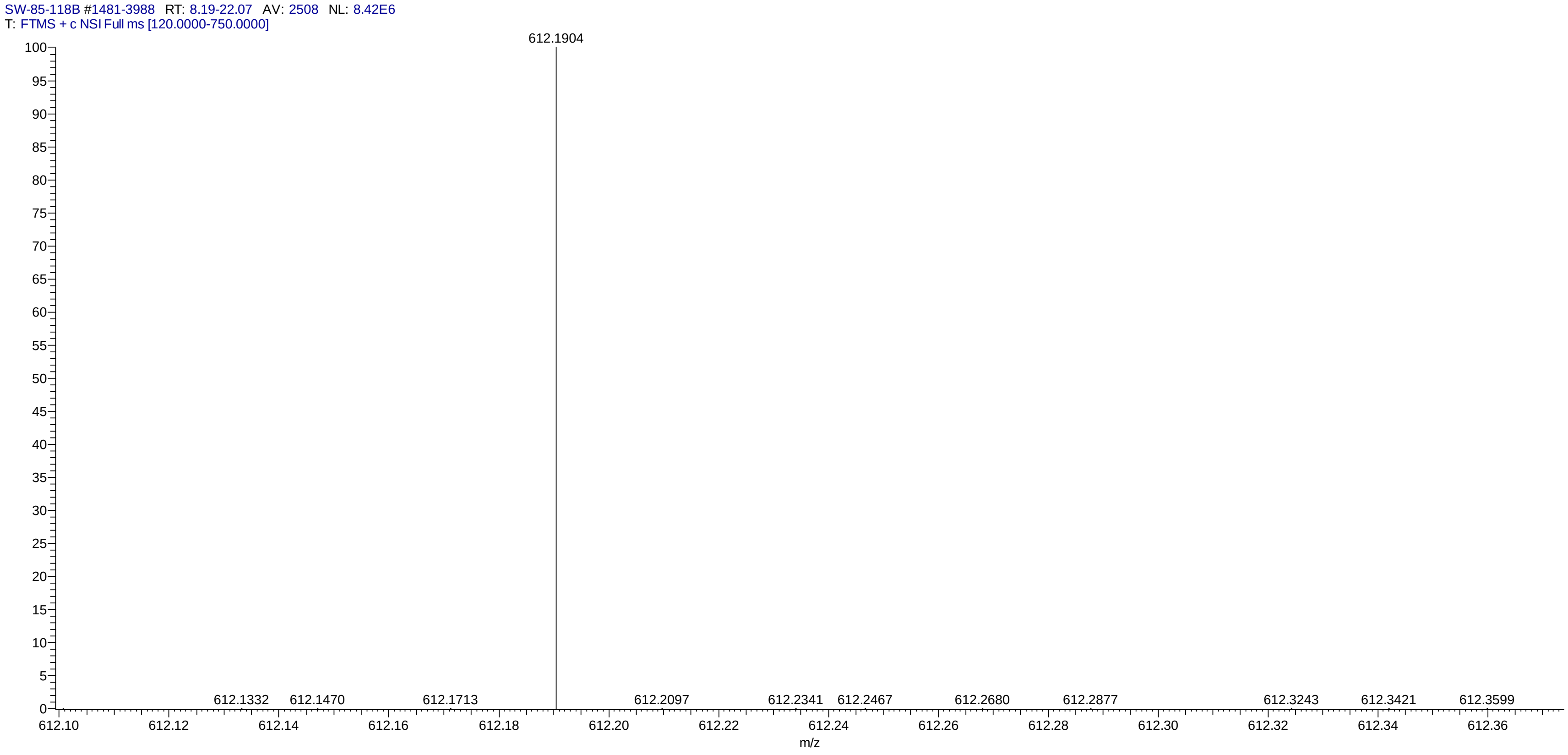

Figure S167: High resolution mass spectrum of **9B**

Injection Date : 4/19/2022 3:34:01 PM
Sample Name : SW-85-118B Location : Vial 1
Acq. Operator :
Method : C:\HPCHEM\1\METHODS\JNP2015.M
Last changed : 4/19/2022 12:01:10 PM
(modified after loading)

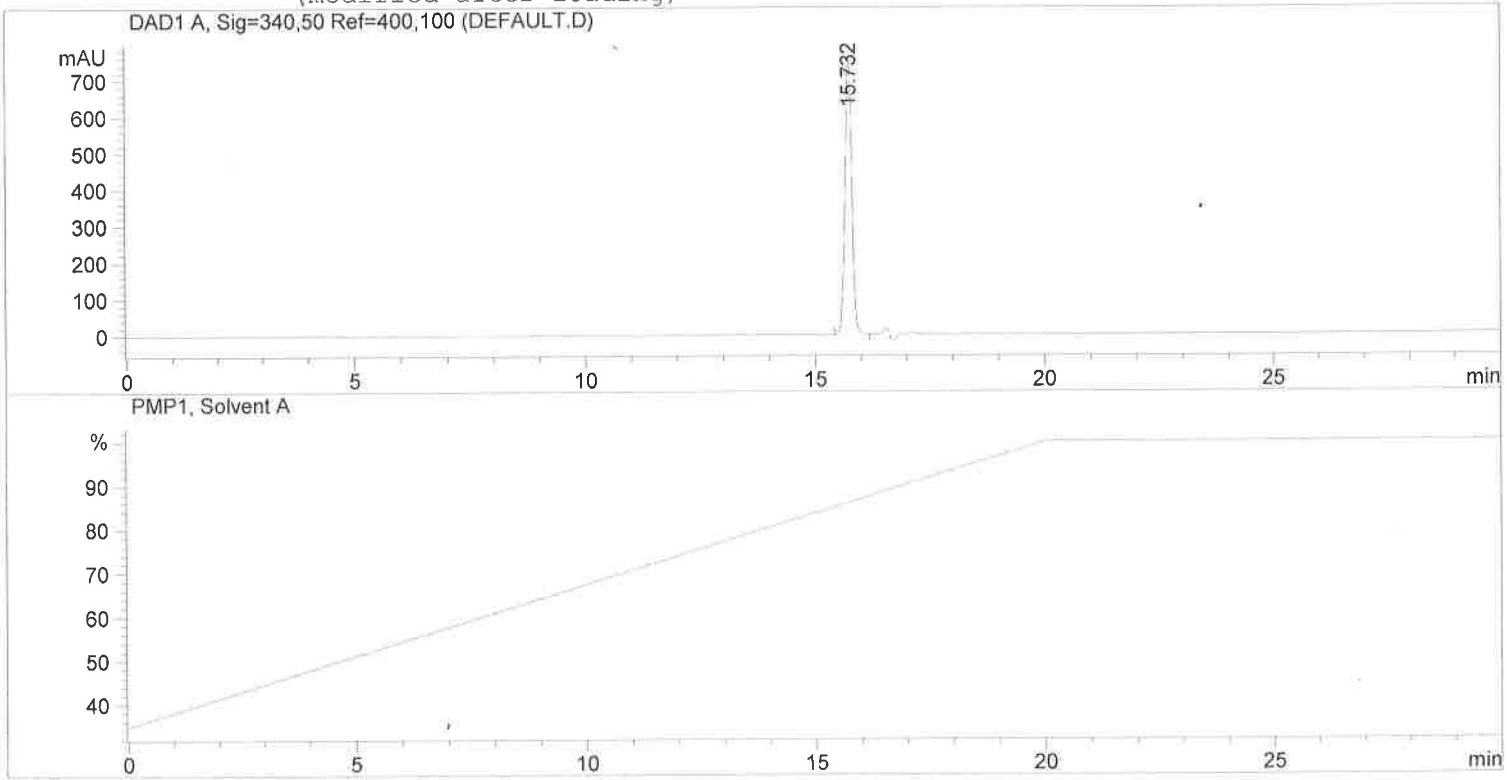

Area Percent Report

Sorted By : Signal
Multiplier : 1.0000
Dilution : 1.0000

Signal 1: DAD1 A, Sig=340,50 Ref=400,100

Peak #	RetTime [min]	Type	Width [min]	Area [mAU*s]	Height [mAU]	Area %
1	15.732	BB	0.1616	8117.97949	766.30164	100.0000

Totals : 8117.97949 766.30164

Results obtained with enhanced integrator!

*** End of Report ***

Figure S168: HPLC Chromatogram of **9B**

Figure S169: COSY spectrum of **5** in $CDCl_3$

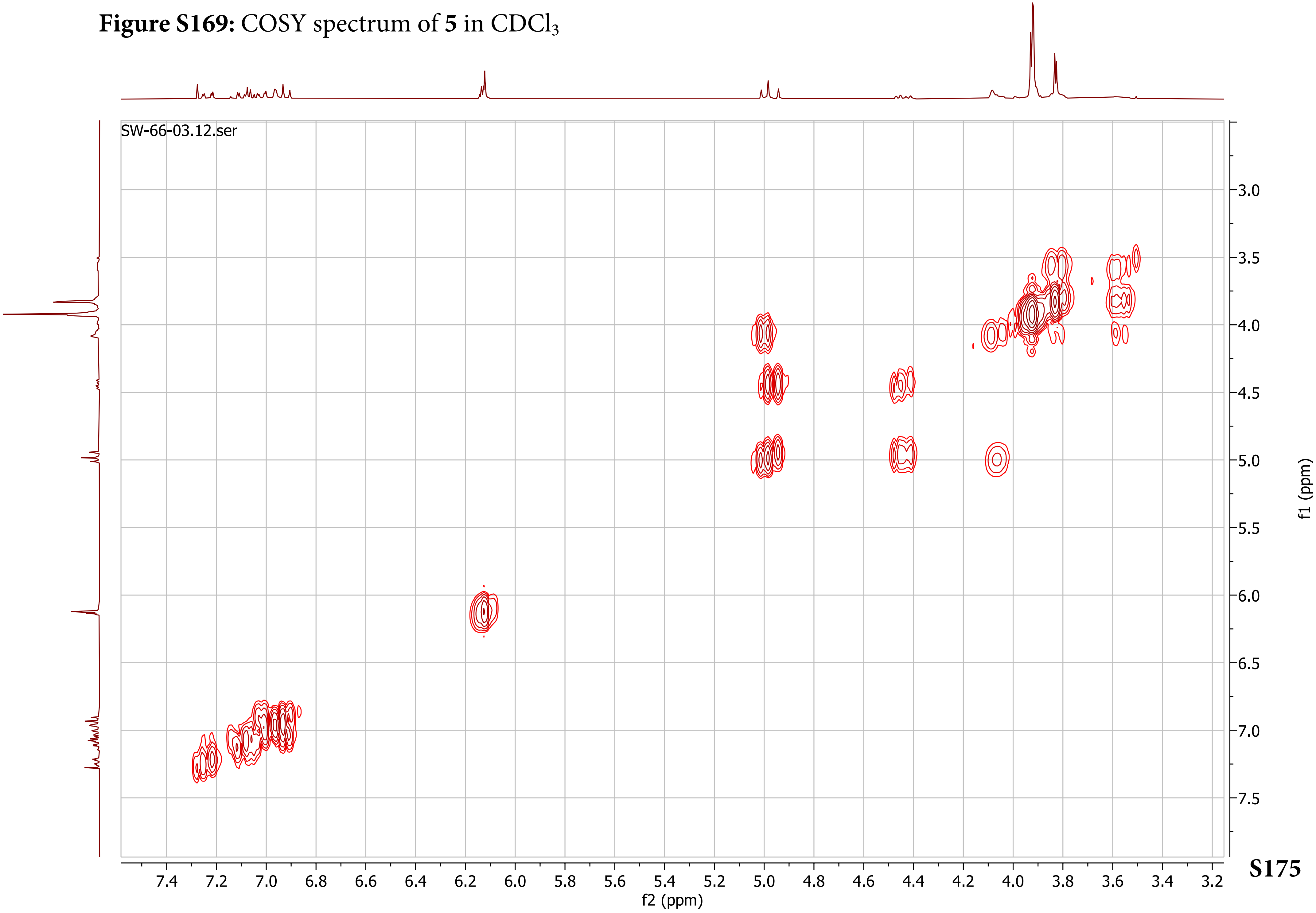

Figure S170: HMQC spectrum of **5** in $CDCl_3$

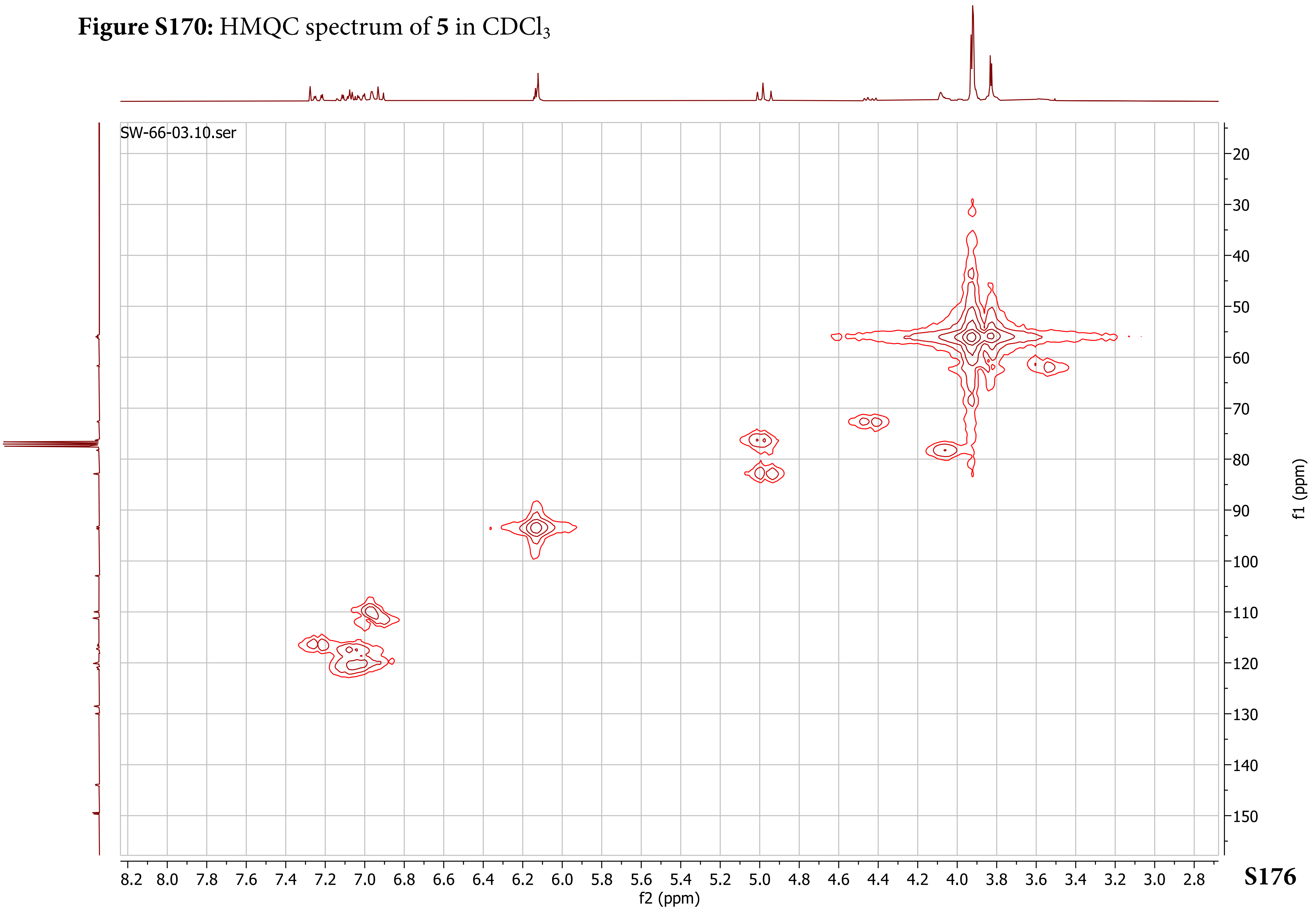

Figure S171: HMBC spectrum of **5** in $CDCl_3$

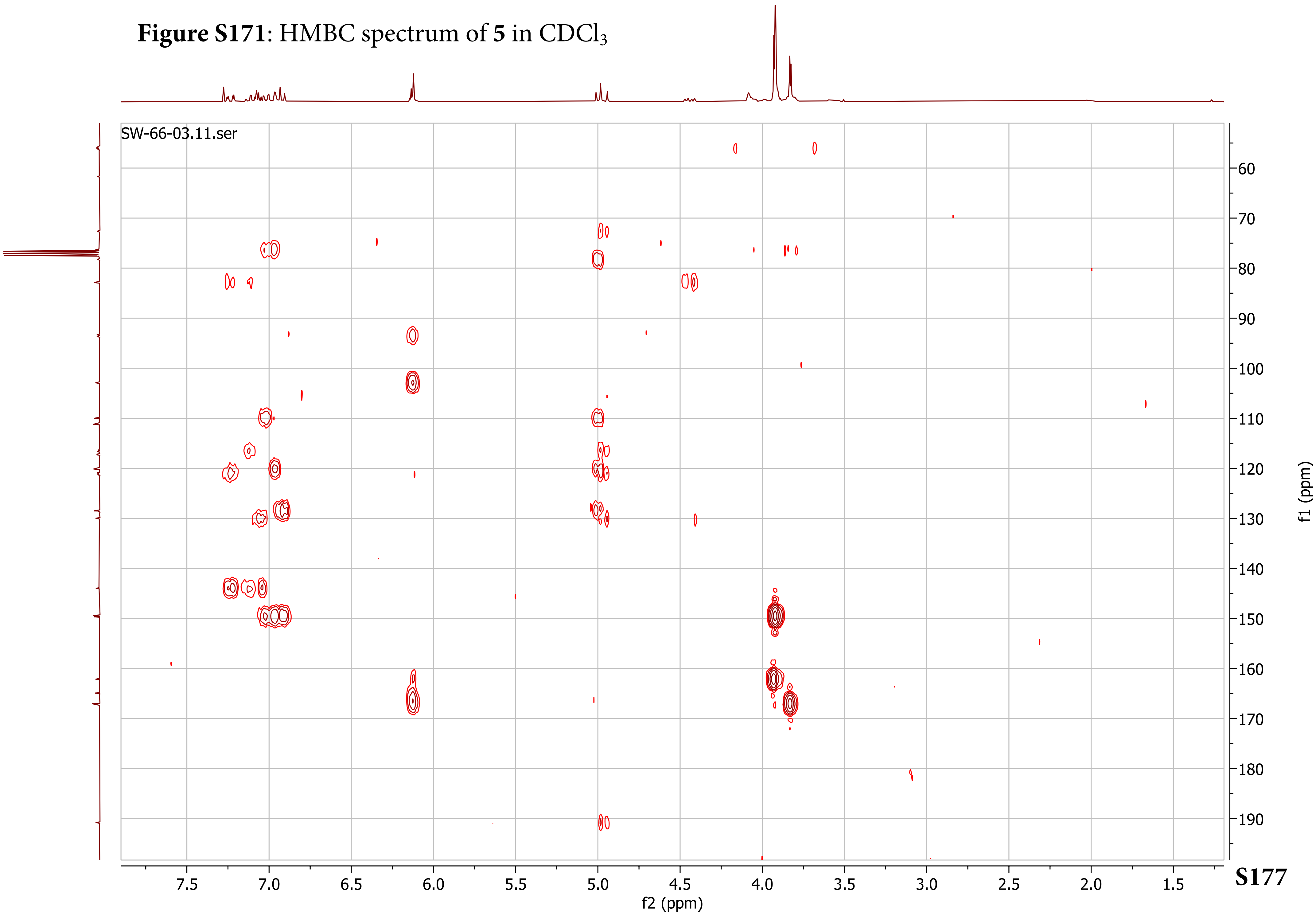

Supplement: Supplementary file 1 [file pharmaceuticals-16-00531-s001.zip › pharmaceuticals-2288043-supplementary.pdf]
